# A computational investigation towards substitution effects on 8π electrocyclisation of conjugated 1,3,5,7-octatetraenes†

**DOI:** 10.1039/d3ra05127g

**Published:** 2023-10-20

**Authors:** Nur Hazimah B. Z. Arfan, Malai Haniti S. A. Hamid, Nadeem S. Sheikh

**Affiliations:** a Chemical Sciences, Faculty of Science, Universiti Brunei Darussalam Jalan Tungku Link, Gadong BE1413 Brunei Darussalam nadeem.sheikh@ubd.edu.bn

## Abstract

A computational investigation using M06-2X/6-31+G(d) method is reported for the substitution effects on 8π electrocyclisation of conjugated octatetraene. This systematic study describes the mono- and di-substitution effect across the 1,3,5,7-octatetraene skeleton. A general preference of the outward substitution over the inward, at C1 position of the monosubstituted system is observed. However, mesomerically electron donating group (–NH_2_ and –OH) display an opposite effect with respect to secondary orbital interaction (SOI) between the lone pair on the substituent and the 
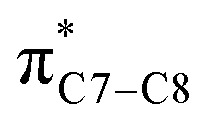
 orbital. A comparative evaluation on the computed activation energies for the 1-, 2-, 3-, and 4-monosubstituted system showed an insignificant impact on the rate of the reaction, in contrast to the electrocyclic ring closure of the unsubstituted compound. Computations of disubstituted system are more pronounced, where a remarkable acceleration is observed for 2-NO_2_–7-NO_2_ substituted octatetraene at 4.9 kcal mol^−1^, and a noticeable deceleration for 4-CH_3_–5-CH_3_ substituted octatetraene at 25.4 kcal mol^−1^ from the parent molecule, 17.0 kcal mol^−1^. A visible accelerated effects are commonly exhibited by the substitution on the terminal double bonds (C1, C2, C7, and C8), that are 1,2-, 1,7-, 1,8-, and 2,7-patterns, in regard to the greater orbital interaction for the new σ-bond formation. Despite the unfavourable steric clashes of the substituents in the 1,8-system, an apparent reduction in the energy barrier up to 7.4 kcal mol^−1^ is computed for 1-NH_2_–8-NO_2_ system from 17.0 kcal mol^−1^. This is due to the synergistic effect of the electron donor and electron acceptor, enhancing the stability of the transition structure. The electrocyclic ring closure involving vicinal substitution patterns, such as 1,2-, 2,3-, 3,4-, and 4,5-systems are critically dominated by steric crowding between the adjacent functional groups. In certain cases of the 1,2-substituted system, a noticeable accelerated effects are found for 1-NH_2_–2-NH_2_-substituted compound (9.7 kcal mol^−1^) due to an increased in electronic density on the substituted terminal double bond (C1–C2), hence favouring the formation of the new σ-bond.

## Abstract 2

Significance of quantum chemical calculations as a theoretical tool to identify the optimum substitution pattern for a rapid 8π-electrocyclisation of conjugated 1,3,5,7-octatetraene is presented.

## Graphical abstract

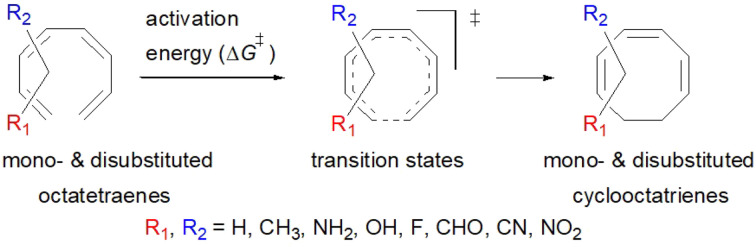

## 1. Introduction

**Fig. 1:**
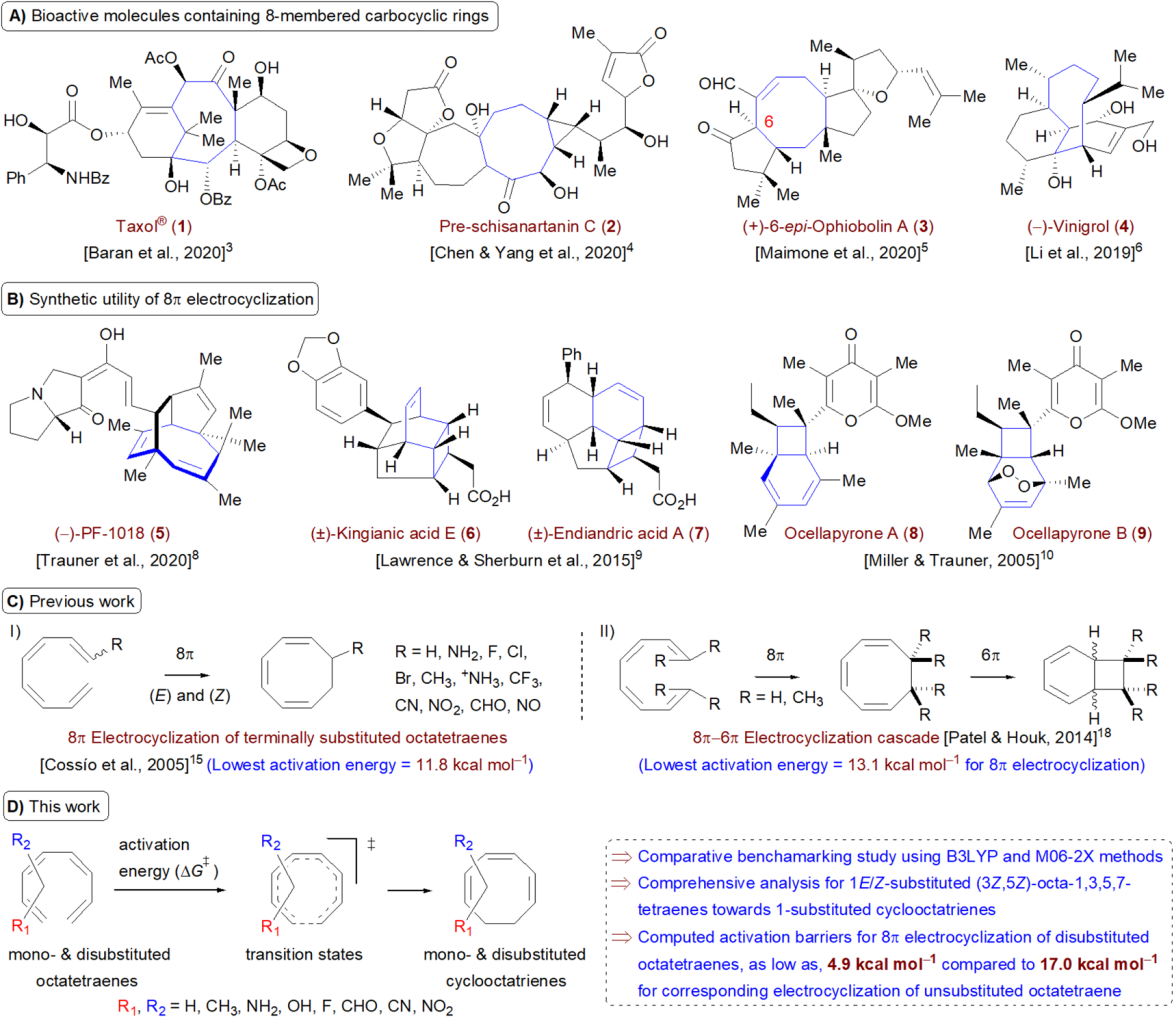
(A) Recent examples of bioactive natural products containing 8-membered carbocyclic rings; (B) representative biologically relevant natural products synthesized by employing 8π electrocyclisation protocol (in cascade); (C) previous work: DFT studies towards 8π electrocyclisation; (D) this work: detailed computational investigation for mono- and disubstituted octatetraenes towards corresponding cyclooctatrienes.

Carbocycles^1^ are structurally diversified motifs, which are ubiquitously present in bioactive natural products and cytotoxic molecules. Amongst them, 8-membered carbocyclic compounds^2^ such as taxol® (1),^3^ pre-schisanartanin C (2),^4^ (+)-6-*epi*-ophiobolin A (3)^5^ and (−)-vinigrol (4,^6^Fig. 1A) are of great synthetic interest due to presence of synthetically challenging architectural features and substantial medicinal relevance. One of the elegant approaches to access these carbocycles is 8π electrocyclisation^7^ of conjugated tetraenes, which has been elegantly applied to synthesize several structurally complex potent compounds including (−)-PF-1018 (5),^8^ (±)-kingianic acid E (6),^9^ (±)-endiandric acid A (7),^9^ ocellapyrone A (8)^10^ and ocellapyrone B (9, Fig. 1B).

In general, electrocyclisation^11^ is a powerful methodology to construct polycyclic scaffolds with highly efficient atom economy.^12^ It proceeds with an excellent and predictable regio- and stereocontrol, governed by Woodward–Hoffmann's rules.^13^ According to these rules, electrocyclic ring closure of conjugated octatetraenes with 8π system is a thermally induced conrotatory process, which involves spontaneous rotation of the terminal olefin bonds of a tetraene. Particularly, the steric effect, electronic properties and torquoselectivity^14^ (inward/outward rotation) of the substituents located at the C-atoms subjected to electrocyclic transformation (ring-opening or closure) play a vital role in orchestrating the observed stereoselectivities. For the conrotatory electrocyclisation of 1-substituted 1,3,5,7-octatetraenes, a pronounced steric effect of the substituents influences the stereochemical outcomes as reported by Houk and colleagues using *ab initio* molecular orbital theory.^14*b*^ In addition, electronic properties of the substituents renders an insignificant effect on stereoselectivities for the octatetraene cyclisation due to dearth of rotational preference caused by the inward and outward substituents located at the terminal C-atoms involved in the bond formation. These findings are in sharp contrast to the conrotatory ring-opening of 3-substituted cyclobutenes, in which, the stereoselectivities are primarily mediated by the electronic nature of the substituents.

Building on the pioneer work championed by Houk *et al.*,^14^ Cossío and co-workers exploited the application of density functional theory (DFT) to further elaborate the electrocyclic ring closure for variously 1-substituted octatetraenes (Fig. 1C(I)).^15^ A detailed investigation suggests complete periselective electrocyclic ring closure of octatetraenes *via* Möbius aromatic transition structure. With a couple of exceptions (–NH_2_ and –NH_3_^+^ substituents), lower barriers have been found for the transition structures which have substituents at the outward positions and this is independent of the electronic nature of the substituents.

The lowest activation energy (11.8 kcal mol^−1^) is computed for inward NH_3_^+^-substituted octatetraene, which is considerably lower than the measured activation energy for the unsubstituted octatetraene *i.e.*, 17.0 kcal mol^−1^.^16^ In addition, rate of reactions for 1,8-dimethyl substituted octatetraene isomers have also been computed, which require substantially higher barrier compared to that of the parent compound. Later, the groups of Schreiner and Suffert applied a computational approach to explore the origin of high torquoselectivities and complete diastereoselectivities observed during the competing synthesis of cyclooctatrienes *vs.* fenestradienes *via* 8π electrocyclic transformations.^17^ As expected, Möbius aromatic transition structures having different *P*- and *M*-helical topologies led to origin of observed selectivities in the cyclooctatrienes, formed as a result of 8π conrotatory electrocyclisation of tetraenes. However, the corresponding fenestradienes are the products of the 8π–6π electrocyclic cascade reactions.

Owing to synthetic utilities and increasing research interests towards 8π–6π electrocyclisation cascade, an inclusive and efficient computational investigation dealing with terminally CH_3_-substituted 1,3,5,7-octatetraenes has been elegantly reported by Patel and Houk (Fig. 1C(II)).^18^ The influence of steric crowding at terminal positions of the tetraene is directly linked with the reaction thermodynamics. Formation of corresponding 1,3,5-cyclooctatrienes *via* 8π electrocyclisation from the un- and monosubstituted tetraenes proceeds rapidly however, 6π electrocyclization leading to highly diastereoselective bicyclic product is relatively slow and reversible. In addition, initial 8π electrocyclisation for di- and trisubstituted reactants is comparatively less exergonic due to destabilization rendered by the steric effect. This leads to an endergonic 8π electrocyclic product for tetrasubstituted tetraene however, the overall reaction involving this cascade still remains exergonic and yields a bicyclic product containing two vicinal quaternary centers.

Here, a systematic and succinct DFT study is reported which delineates the mono- and disubstitution effect across the skeleton of 1,3,5,7-octatetraenes (Fig. 1D). Such an effect has already been reported for thermal conrotatory 4π electrocyclic ring-opening of substituted cyclobutenes^19^ and thermal disrotatory 6π electrocyclic ring-closure of 1,3,5-hexatrienes.^20^

To start with, a selection of commonly used substituents ranging from electron donating to withdrawing substituents have been subjected to this study. A remarkable accelerated substitution effect has been noticed that lowers the activation free energy for the 8π electrocyclisation up to 4.9 kcal mol^−1^ compared to 17.0 kcal mol^−1^, experimentally measured for the unsubstituted 1,3,5,7-octatetraenes.^16^ At the same time, certain disubstituted patterns produce negative effect, leading to a noticeable increase in the activation barriers; the maximum computed to be 23.6 kcal mol^−1^.

## 2. Results and discussion

### 2.1 8π electrocyclisation of unsubstituted 1,3,5,7-octatetraenes

At the outset, a detailed comparative benchmarking study was envisaged as both B3LYP and M06-2X methods have been reported for the electrocyclisation of octatetraenes (Table 1).^15,18^ For an extensive and systematic study of a particular transformation, as it is, computational expense plays a crucial role, as well as, evaluation of calculations with the experimental values. Several basis sets were selected for both methods to find an appropriate computational approach for further investigation. It is evident from this study that B3LYP method overestimates the barrier for tetraene by ∼2 kcal mol^−1^ compared to M06-2X method. With the same time, a significant difference in reaction energies have been observed for both methods. Apparently, B3LYP method fails to accurately compute the reaction thermodynamics by showing approximate thermoneutral behaviour of electrocyclisation while M06-2X method, as expected, describes the exergonic nature of the 8π electrocyclisation of unsubstituted 1,3,5,7-octatetraenes.

**Table 1:** Comparative benchmarking study using B3LYP and M06-2X methods to compute the activation energies (Δ*G*^‡^, kcal mol^−1^) and reaction energies (Δ*G*, kcal mol^−1^) for conrotatory 8π electrocyclic ring-closure of 1,3*Z*,5*Z*,7-octatetraene and disrotatory 8π electrocyclic ring-closure of 1,3*Z*,5*E*,7-octatetraene (in parentheses)

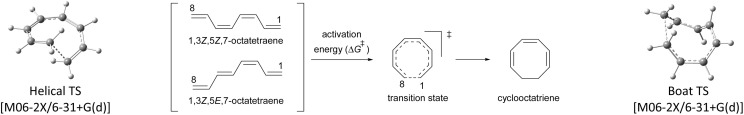									
Entry	Basis set	B3LYP				M06-2X			
		Δ*G*^‡^	ΔΔ*G*^‡^^a^	Δ*G*	C1–C8^b^	Δ*G*^‡^	ΔΔ*G*^‡^^a^	Δ*G*	C1–C8^b^
1	6-31G(d)	18.3 (48.3)	30.0	−3.8 (−1.3)	2.36301 (2.37609)	16.3 (45.7)	29.4	−11.3 (−9.1)	2.34510 (2.30942)
2	**6-31+G(d)**	19.1 (48.4)	29.3	−2.2 (0.3)	2.36304 (2.39441)	**17.0** (45.8)	28.8	−9.9 (−7.8)	2.34147 (2.32612)
3	6-31G(d,p)	18.3 (48.2)	29.9	−3.4 (−0.9)	2.35405 (2.37660)	16.4 (45.6)	29.2	−10.9 (−8.7)	2.33886 (2.31052)
4	6-31+G(d,p)	19.2 (48.4)	29.2	−1.6 (0.9)	2.33727 (2.39580)	17.3 (45.9)	28.6	−9.2 (−7.2)	2.33415 (2.32772)
5	Experimental^16^	**17.0**							

a Activation energy difference between the disrotatory and conrotatory electrocyclic ring closures.

b Bond distance values are for the conrotatory and disrotatory (in parentheses) electrocyclic transition states and in Å.

Compared to experimental result, M06-2X/6-31+G(d) is the most appropriate basis set for the unsubstituted 1,3,5,7-octatetraenes (entry 2, Table 1). It is worth noticing that an absence of diffused function (entry 1, Table 1) and addition of polarization function to the hydrogen atoms (entry 3, Table 1) result in lower activation barriers compared to experimental value. At the same time, basis set containing both diffused and polarizations functions for heavy and hydrogen atoms offer slightly higher barrier (entry 4, Table 1). The reaction preferably proceeds through the helical shaped TS,^21^ which is associated with the conrotatory electrocyclisation of tetraene. In addition, disrotatory movement of molecular orbitals would undergo *via* deformed boat-shaped TS of the 1,3*Z*,5*E*,7-octatetraenes and has significantly higher activation barrier, as reported by Cossío *et al.*^15^

For the optimum method concluded from this benchmarking study [M0-62X/6-31+G(d)], the activation energy difference between the conrotatory and disrotatory TSs is 28.8 kcal mol^−1^, which is substantially higher than the measured activation energy *i.e.*, 17.0 kcal mol^−1^.^16^ More or less, similar difference in the activation barriers is observed for all the basis sets considered for this study.

**Fig. 2:**
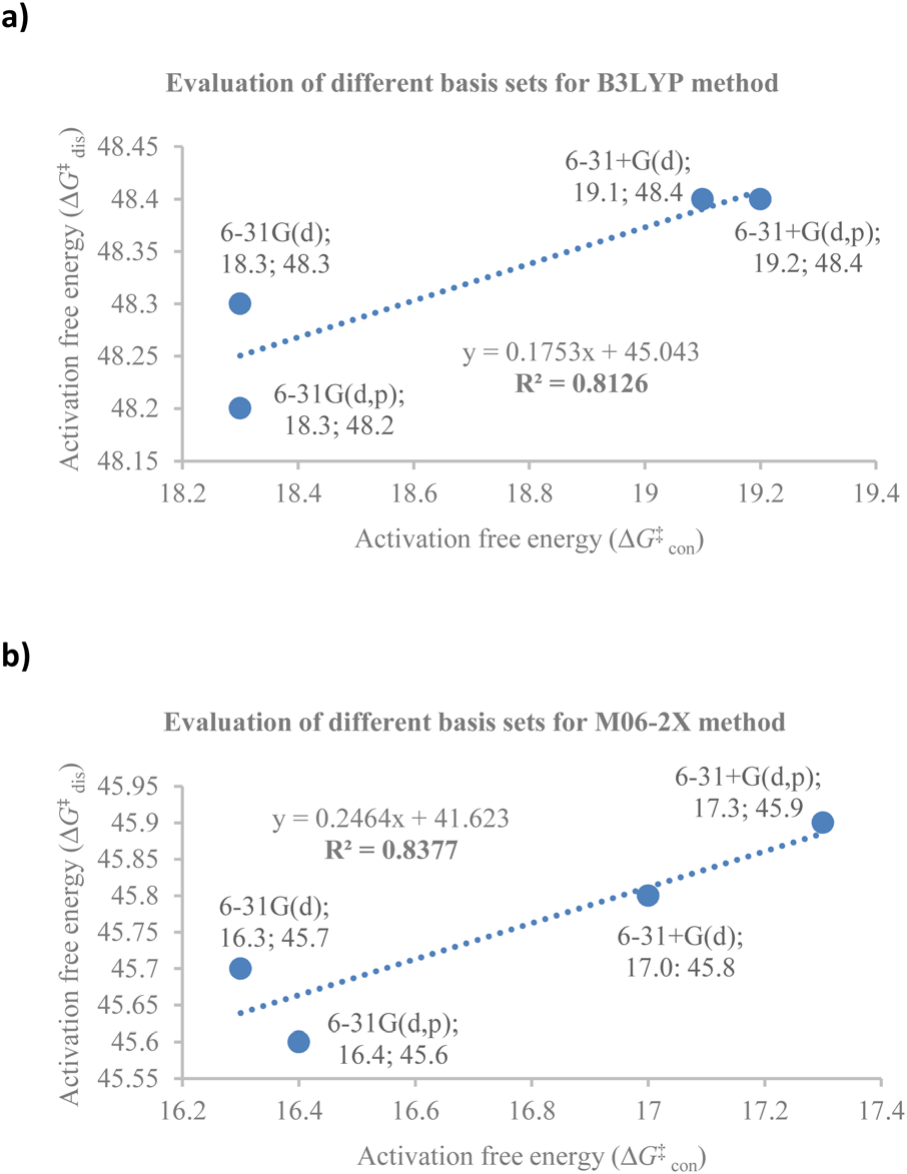
The correlation between activation free energies for conrotatory (con) and disrotatory (dis) electrocyclisation of unsubstituted 1,3*Z*,5*Z*,7-octatetraene and 1,3*Z*,5*E*,7-octatetraene respectively, using different basis sets for (a) B3LYP and (b) M0-62X methods.

Further comparative analysis between B3LYP and M06-2X methods reveals a good correlation between the activation barriers for the conrotatory and disrotatory electrocyclisation using different basis sets for B3LYP method (Fig. 2a) and the corresponding M06-2X method (Fig. 2b). There is a strong correlation between the activation and reaction energies for the conrotatory electrocyclisation, however poor correlation was found for the disrotatory process.

### 2.2 8π electrocyclisation of monosubstituted 1,3,5,7-octatetraenes

The computational results of thermally allowed conrotatory 8π electrocyclic ring closure of terminally monosubstituted system using M06-2X/6-31+G(d) method are shown in Table 2. The rate of these octatetraene cyclisation is determined by the outward or inward arrangement of the substituent on the system. Cossío *et al.* has identified a general preference of the outward attachment over the inward, as a consequence of lower steric crowding experienced by the substituent in the transition state.^15^ Such interaction renders slight stabilization to the outward transition structure, which was reflected by the lower computed activation barrier regardless of its electron donating or electron accepting character. Our computational findings have also shown similar trends. A minor preference of 1.5 to 2.6 kcal mol^−1^ lower than the computed activation energies for the substituent to be in the inside position are recorded. Consequently, the stereochemical outcomes of these electrocyclic reactions are directed by the steric effects.

**Table 2:** Comprehensive study for conrotatory electrocyclisation of (1*E*)- and (1*Z*)-substituted octatetraenes using M06-2X/6-31+G(d)^a^

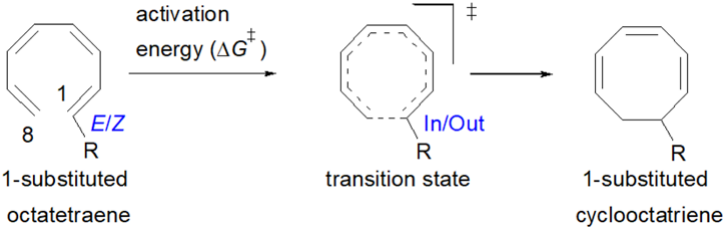										
Entry	R	Δ*G*^‡^_(out TS)_^b^	Δ*G*^‡^_(In TS)_^c^	Δ*G*^‡^_(in–out)_^d^	Δ*G*_(1*E*)_^e^	Δ*G*_(1*Z*)_^e^	C1–C8_(out TS)_^f^	C1–C8_(In TS)_^f^	R–C8_(out TS)_^f^	R–C8_(In TS)_^f^
1	CH_3_	19.2	20.7	1.5	−5.9	−7.4	2.30891	2.32493	3.09747	3.03689
2	NH_2_	**19.2**	**16.0**	−3.2	−2.8	−3.4	2.19929	2.18498	2.94978	2.95553
3	OH	**17.3**	**14.9**	−2.2	−7.9	−7.6	2.24732	2.20575	2.96520	2.87002
4	F	16.5	19.1	2.6	−12.2	−11.0	2.30316	2.27767	2.97892	2.80513
5	CHO	17.0	17.9	0.9	−5.0	−7.5	2.29083	2.33002	2.97881	2.88939
6	CN	18.2	19.9	1.7	−5.1	−5.9	2.30282	2.31697	3.00091	2.89120
7	NO_2_	16.9	18.5	1.6	−9.6	−12.3	2.32672	2.38439	2.99101	2.84308

a For computed values using M06-2X/6-31+G(d,p), see ESI.

b Activation barriers (kcal mol^−1^) for conrotatory electrocyclisation of (1*E*)-substituted octatetraenes (outward-substituted transition structures).

c Activation barriers (kcal mol^−1^) for conrotatory electrocyclisation of (1*Z*)-substituted octatetraenes (inward-substituted transition structures).

d Activation energy difference (kcal mol^−1^) between the inward- and outward-substituted transition structures.

e Reaction energies (kcal mol^−1^) for the electrocyclisation of (1*E*)- and (1*Z*)-substituted octatetraenes.

f Bond distances are from the corresponding transition states and the values are in Å (for optimized geometries, see ESI).

**Fig. 3:**
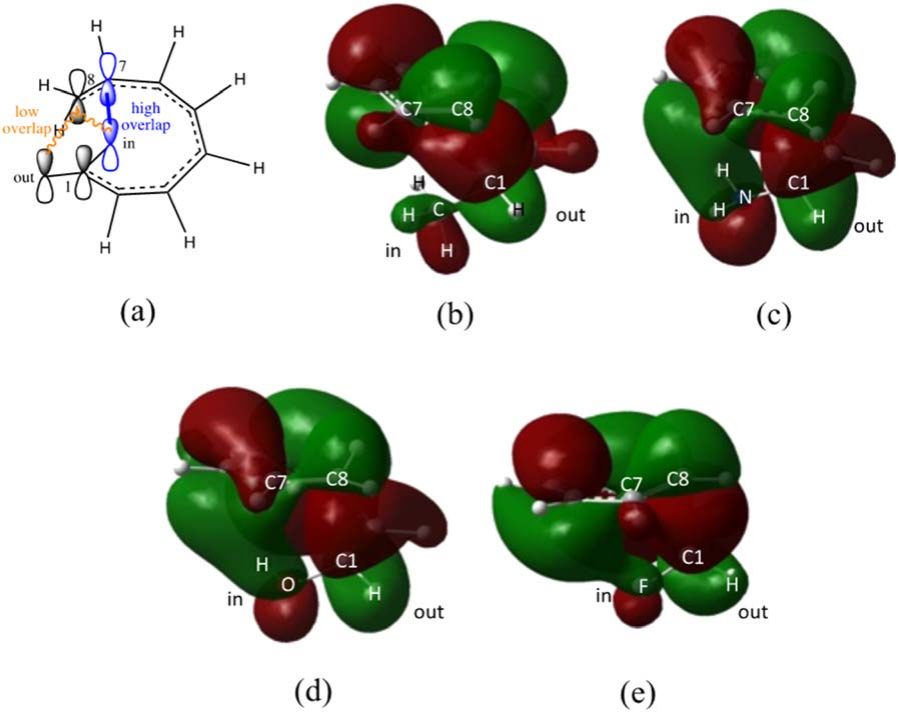
(a) General (generic) atomic orbital interaction in eight-electron conrotatory electrocyclic reactions. Secondary orbital interaction between the lone pair of the inward substituent with the atomic orbital on C7 of the tetraene compound shown in blue. The atomic orbitals interaction of the substituents, (b) –CH_3_, (c) –NH_2_, (d) –OH, and (e) –F, with that of the C7 position are represented in red and green.

On the other hand, installation of mesomerically electron donating group, –NH_2_ and –OH group, shows an opposite preference of the torquoselectivity in the electrocyclisation process. Presumably, a secondary orbital interaction (SOI)^15^ between the lone pair on the functional group and the localized 
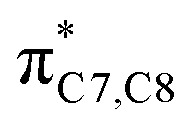
 orbital, as shown in Fig. 3, provide extra stabilization to the transition structure. This interaction is favoured by the excellent overlap of the atomic orbital of the inward substituent with the atomic orbital on C7 (Fig. 3a). Moreover, it depends on the capacity of the electron donor character. Introduction of other mesomerically electron donating groups, –CH_3_ and –F, at the terminal position with an inward substitution does not offer a stabilizing SOI to the transition structures (Fig. 3b and e), particularly due to the weaker electron donating effect. Despite the presence of lone pairs on –F, the large electronegativity demonstrates an inductive effect that lowers the π-donor capability towards the 
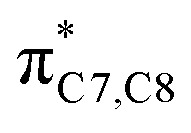
 orbital of the terminal double bond (Fig. 3e).

It is noteworthy that the carbonyl (–CHO) and hydroxyl (–OH) functional groups can be oriented in two possible conformations on the tetraene molecule, as was reported in the computational investigation of substituted 4π system paper.^19^ Hence, lowest energy conformation is used to study the reaction energies. In contrast to the unfunctionalised octatetraene (17.0 kcal mol^−1^), electronic properties of the substituent in a 1-substituted system hardly affect the rate of the transformation, ranging from 14.9 to 20.7 kcal mol^−1^. The mild steric effects do bring about the minute preference for the outward mode of rotation of the terminal substituent.

We have also computed the activation and reaction energies of the transition structure with functionality located at 3 other possible positions (C2-, C3-, and C4-position) to extensively study the mono-substituent effect on the reactivity of electrocyclization of the tetraene molecule (Table 3). Based on the computations, electronic properties of the functionality at C2-position are maximized, while the steric effects are minimized. A much larger reduction in the activation energies is observed, contrary to the other monosubstituted systems. At C3- and C4-monosubstituted octatetraenes, the energies are practically indistinguishable from the terminally monosubstituted system. The reasons may be attributed to the inconsequential electronic interaction of the substituent due to an indirect interaction with the terminal double bonds that are involved in the formation of the new σ-bond.

**Table 3:** Activation energies (Δ*G*^‡^, kcal mol^−1^) and reaction energies (Δ*G*, kcal mol^−1^) given in parentheses for conrotatory electrocyclization of 2-substituted (entries 1–7), (3*Z*)-methyl substituted (entry 8), (3*E*)-substituted (entries 9–14), (4*Z*)-methyl substituted (entry 15) and (4*E*)-substituted (entries 16–21) octatetraenes

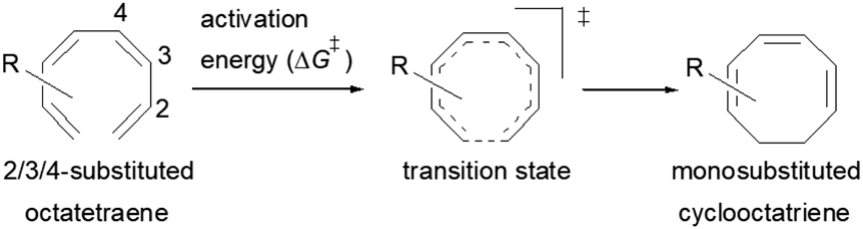											
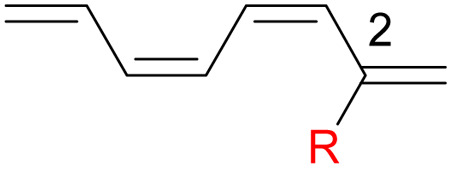				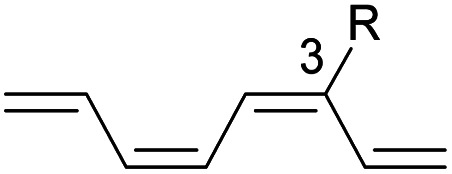				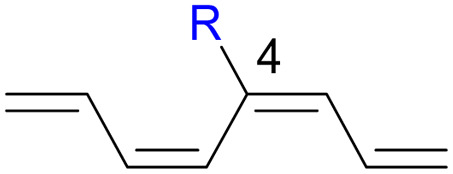			
Entry	R	Δ*G*^‡^^a^	Δ*G*^‡^^b^	Entry	R	Δ*G*^‡^^a^	Δ*G*^‡^^b^	Entry	R	Δ*G*^‡^^a^	Δ*G*^‡^^b^
1	CH_3_	14.5 (−12.4)	14.7 (−11.9)	8	CH_3_	19.1 (−8.3)	19.2 (−7.7)	15	CH_3_	18.0 (−12.0)	18.0 (−11.5)
2	NH_2_	15.7 (−12.5)	15.7 (−12.1)	9	NH_2_	19.4 (−6.3)	19.5 (−5.7)	16	NH_2_	18.5 (−11.1)	18.6 (−10.5)
3	OH	17.8 (−11.6)	18.3 (−10.7)	10	OH	19.1 (−7.6)	18.2 (−8.0)	17	OH	17.5 (−11.3)	18.0 (−10.6)
4	F	15.8 (−13.5)	16.0 (−12.9)	11	F	17.3 (−9.8)	17.5 (−9.2)	18	F	15.1 (−12.2)	15.3 (−11.6)
5	**CHO**	**11.4 (−16.8)**	**11.6 (−16.3)**	12	CHO	14.9 (−12.5)	14.1 (−10.7)	19	CHO	14.1 (−13.7)	14.2 (−13.2)
6	CN	14.2 (−13.3)	14.4 (−12.6)	13	CN	17.9 (−9.1)	18.0 (−8.5)	20	CN	15.2 (−12.7)	15.4 (−12.1)
7	**NO**_**2**_	**10.2 (−17.2)**	**10.5 (−16.6)**	14	NO_2_	12.1 (−15.2)	12.3 (−14.7)	21	NO_2_	14.8 (−13.4)	14.8 (−12.9)

a For computed values using M06-2X/6-31+G(d).

b For computed values using M06-2X/6-31+G(d,p).

Particularly, incorporating a strong electron-withdrawing group (–CHO and –NO_2_) at these positions (C2, C3 or C4) reveals an increase in the rate of the tetraene ring closure. A noticeable reduction is shown especially by the substitution at position C2, where the electronic properties are maximized and steric congestion are minimized.

The reaction of 2-CHO (Table 3, entry 5) and 2-NO_2_ (Table 3, entry 7) system are accelerated to 11.4 kcal mol^−1^ and 10.2 kcal mol^−1^, respectively, from 17.0 kcal mol^−1^ as reported for the unsubstituted 1,3,5,7-tetraene. It is worth mentioning that the strength of the electron withdrawing character is directly linked with facilitating the re-arrangement of the π-bonds and generation of the new σ-bond, to form a ring structure. Overall, a comparative evaluation of the results extracted for C1- to C4-substituted tetraenes with the unsubstituted parent 1,3,5,7-octatetraenes substrate shows a trivial impact on the acceleration of the 8π electrocyclisation reaction.

With regard to further verify the suitability of the selected DFT method for this investigation, calculations using the M06-2X/6-31+G(d,p) level of theory are conducted for all the monosubstituted patterns. The computed values (refer to ESI†) express only minor variation by up to 0.5 kcal mol^−1^ and 0.9 kcal mol^−1^ for the activation energies and the reaction energies, respectively when compared with M06-2X/6-31+G(d) level of theory. Henceforth, we conclude that the use of a lower basis set which is less computationally expensive is suitable to generate a reliable information for this organic transformation.

### 2.3 8π electrocyclisation of disubstituted 1,3,5,7-octatetraene

We have also extensively explored the disubstituted 1,3,5,7-octatetraenes using the M06-2X/6-31+G(d) method (Table 4). All possible substitutional patterns of different combinations at different site on the tetraene backbone are computed. Such investigation aims to understand the activation barrier requirements for the 8π electrocyclisation of the conjugated difunctionalised system.

**Table 4:** Activation energies (Δ*G*^‡^, kcal mol^−1^) using M062X/6-31+G(d) for electrocyclic ring-closure of variously functionalised 1,*X*-disubstituted octatetraenes (*X* = 2–8)

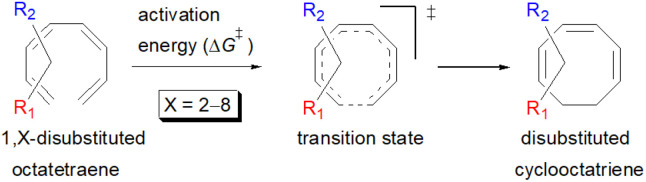									
			R_2_						
			CH_3_	NH_2_	OH	F	CHO	CN	NO_2_
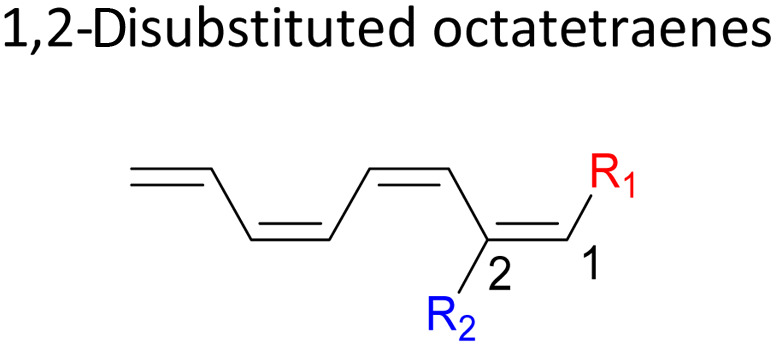	R_1_	CH_3_	14.6	13.1	14.5	15.5	14.4	13.1	14.2
		NH_2_	13.6	**9.7**	10.8	12.3	20.9	15.7	14.7
		OH	14.4	10.4	10.7	11.8	19.0	14.2	14.0
		F	14.2	12.7	15.5	17.0	14.3	15.6	15.4
		CHO	13.1	13.0	*21.0*	19.2	13.2	14.5	11.2
		CN	14.0	15.4	19.4	17.6	13.4	14.7	13.8
		NO_2_	12.2	12.4	19.2	18.9	12.4	13.2	13.7
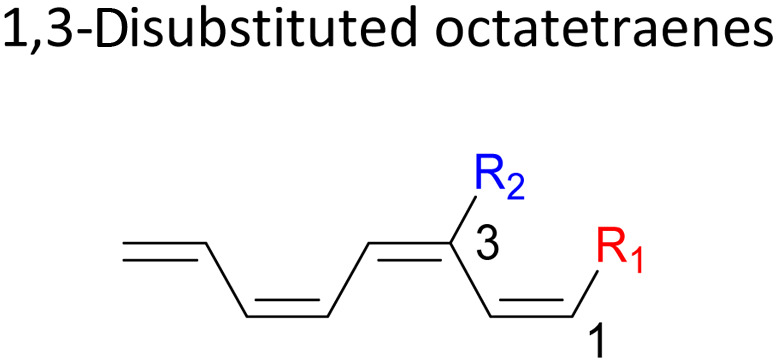	R_1_	CH_3_	14.7	17.5	16.9	15.6	14.0	15.3	13.5
		NH_2_	16.6	*21.2*	*21.9*	20.2	14.9	19.3	16.0
		OH	16.7	19.9	16.6	15.2	14.8	15.8	13.4
		F	17.7	18.4	16.8	15.7	14.0	15.5	13.4
		CHO	12.4	15.3	15.9	14.4	12.8	13.5	11.7
		CN	18.0	18.2	16.8	14.7	13.5	14.3	11.8
		NO_2_	11.0	12.0	10.6	**8.5**	10.7	10.5	**9.6**
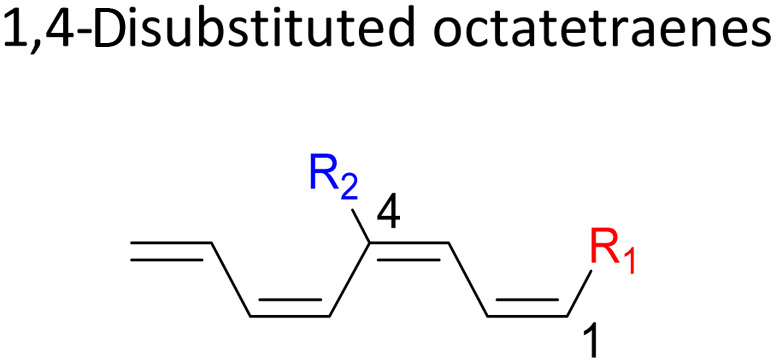	R_1_	CH_3_	16.4	17.8	18.5	16.0	13.9	16.1	14.2
		NH_2_	16.8	18.8	18.3	16.4	15.7	17.0	15.6
		OH	17.4	19.3	18.7	16.4	15.0	16.2	14.5
		F	17.8	19.4	18.3	15.8	13.8	15.2	14.8
		CHO	14.6	17.3	16.2	14.2	11.8	14.0	12.0
		CN	17.1	20.0	18.8	16.0	13.9	16.4	13.8
		NO_2_	13.6	17.3	16.4	13.1	**9.8**	12.8	13.5
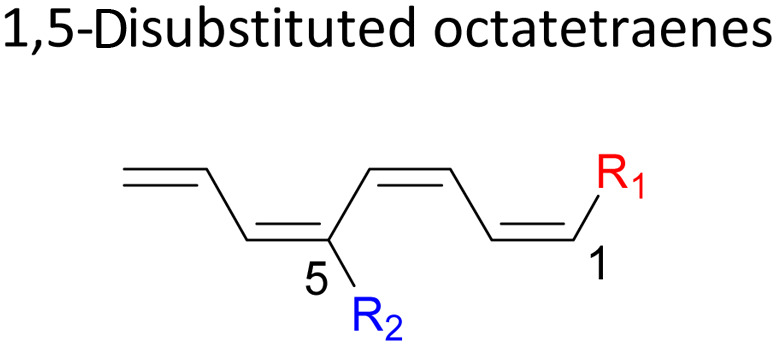	R_1_	CH_3_	15.7	18.2	16.2	14.6	13.9	15.8	13.2
		NH_2_	15.7	18.3	17.7	15.8	14.6	16.3	14.3
		OH	17.2	18.7	18.1	16.1	14.9	16.4	14.2
		F	16.8	18.8	17.7	16.1	15.0	17.3	15.3
		CHO	14.5	16.7	16.3	14.5	12.2	14.2	12.3
		CN	17.0	18.2	17.8	18.0	15.2	17.7	14.9
		NO_2_	12.8	14.8	15.6	13.9	11.8	14.0	11.8
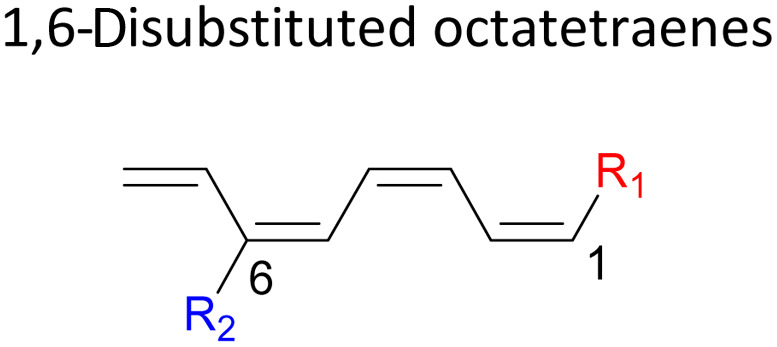	R_1_	CH_3_	18.5	19.2	18.6	17.5	14.0	16.2	13.3
		NH_2_	18.9	19.9	19.3	17.7	14.4	17.1	14.2
		OH	19.1	20.6	19.9	18.1	14.1	17.7	13.9
		F	19.5	19.8	18.9	17.4	14.2	18.1	12.9
		CHO	16.8	16.8	17.0	15.3	12.8	15.8	**9.9**
		CN	19.5	19.2	18.4	17.1	15.6	19.6	15.2
		NO_2_	14.3	15.3	15.0	14.7	11.9	15.4	11.7
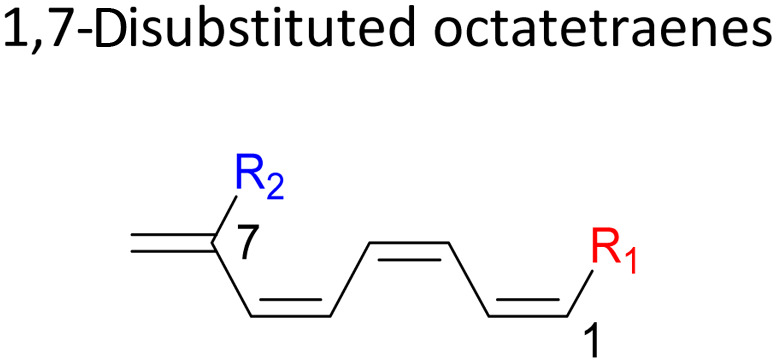	R_1_	CH_3_	13.0	14.8	15.7	16.2	10.2	11.9	**8.6**
		NH_2_	14.5	14.1	16.0	15.9	11.9	13.1	**8.0**
		OH	14.7	15.2	17.4	16.5	12.0	14.3	10.7
		F	15.1	15.2	17.1	16.1	12.5	15.5	11.3
		CHO	12.6	14.0	16.0	14.9	**9.4**	13.2	**8.9**
		CN	15.1	15.3	18.8	16.9	12.0	16.7	12.4
		NO_2_	10.3	11.0	15.7	15.3	**8.6**	13.9	**9.6**
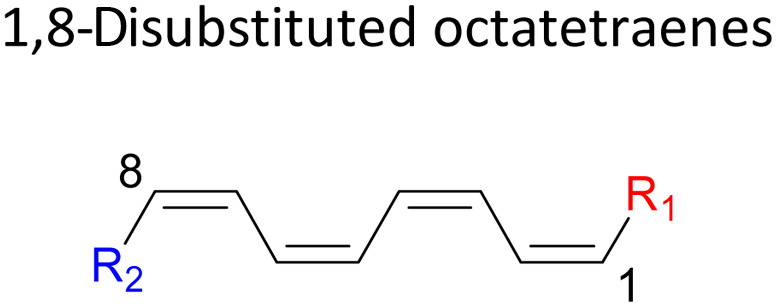	R_1_	CH_3_	17.0	16.5	16.4	15.9	14.6	15.5	12.0
		NH_2_		16.5	17.0	15.6	12.1	13.2	**7.4**
		OH			15.6	17.2	16.0	16.2	11.3
		F				19.0	17.4	18.9	16.0
		CHO					14.3	18.3	13.4
		CN						*21.4*	17.9
		NO_2_							14.8

#### 2.3.1 1,*X*-Disubstituted 1,3,5,7-octatetraenes (*X* = 2–8)

The activation energies for 1,2-disubstituted octatetraene are influenced by both the electronic and steric effects of the substituent on the molecular backbone and, according to their electronic nature. Inclusion of strong electron donating group (–NH_2_) at both positions (C1 and C2) on the tetraene skeleton demonstrate a noticeable accelerated effect to the tetraene cyclization, reflected by the reduced activation energy to 9.7 kcal mol^−1^ from 17.0 kcal mol^−1^ (Table 4). This is related to the capacity of the electron donating character of the substituents on one of the terminal carbon double bonds (C1–C2), in which they enhanced the orbital interaction between the two termini (C1 and C8) to form a ring structure.

In the case of dinitro functionalized system (1-NO_2_–2-NO_2_), it is not surprising that only minor reduction in the activation barrier by 3.3 kcal mol^−1^ from 17.0 kcal mol^−1^ is recorded, in spite of their strong electron withdrawing effect. It arises from the steric congestion between the vicinal nitro groups that slightly destabilizes the transition structure. Greater destabilization leading to a higher energy barrier is presented by 1-CHO–2-OH substituted compound at 21.0 kcal mol^−1^ due to the unfavourable adjacent side groups clashes.

In 1,3-disubstituted model systems, the strain experience between the motifs becomes less eminent as they no longer feature in vicinal clashing. The reaction energies are seen to slightly be driven by the electronic properties of the substituents due to their minor electronic contribution at C3 position to the terminal double bonds to facilitate the formation of the new σ-bond. Hence, a decrease in reaction reactivity is recorded by the 1,3-diamino substituted system (1-NH_2_–3-NH_2_) to 21.2 kcal mol^−1^, in contrast to the corresponding 1,2-disubstituted (9.7 kcal mol^−1^) and also the unsubstituted system (17.0 kcal mol^−1^). Similar trends are also observed for some other 1,3-difunctionalized tetraenes. However, reverse trend is observed when functionalities with high electron withdrawing character are incorporated. An obvious increased in the activation barrier up to 8.5 kcal mol^−1^ and 9.6 kcal mol^−1^ from 17.0 kcal mol^−1^, are shown by the 1-NO_2_–3-F and 1-NO_2_–3-NO_2_ system, respectively. The effect of the functional groups installed at positions C4, C5 and C6 on the tetraene skeleton of the 1,*X*-disubstituted octatetraene become less significant during the cyclisation step. The location of these installation inconsequentially delegates effective electronic contribution with the molecular orbital for the newly formed C1–C8 bond. The energy trends observed for 1,7-disubstituted octatetraene are more pronounced than the other 1,*X*-systems (Table 4). It is the electronic effect of both substituents at these positions that are responsible for the observed accelerated transformation. Direct attachment of these groups on the terminal carbon double bonds promotes the orbital interactions between the forming C1–C8 σ-bond. A measurable decrease in the activation energies of 1-R_1_–7-CHO and 1-R_1_–7-NO_2_ systems are observed, particularly as a result of the R_2_ side group's strong electronic nature and their position on the tetraene skeleton where the electronic contributions are maximized and steric effects are minimized. The most accelerated transformation is observed by the 1-NH_2_–7-NO_2_ system, in which the activation energy is 8.0 kcal mol^−1^ compared to 17.0 kcal mol^−1^ for unsubstituted system. The synergistic effect between the two substituents with opposite electronic characteristics (–NH_2_ having electron donating effect, and –NO_2_ having electron withdrawing effect) and the magnitude of these properties effectively promotes the rate of ring closure. Greater orbital interaction with the terminus carbon atoms renders better stabilization to the transition structure, hence favouring the cyclooctatriene formation. Similar reactivity trends of such effects is also observed for 1-CH_3_–7-NO_2_ and 1-OH–7-NO_2_ substituted systems at 8.6 kcal mol^−1^ and 10.7 kcal mol^−1^, respectively.

In addition, a slightly more enhanced reactivity resulted by this synergistic effect is shown by 1-NH_2_–8-NO_2_ functionalized octatetraene. The computed activation free energy for this combination is 7.4 kcal mol^−1^. Despite the slight destabilization by the steric crowding between the vicinal functional groups at the transition state, the capacity of the electron donating and electron withdrawing characters, together with the direct attachment to the terminus position leads to a more stabilized and favourable orbital interaction. On the contrary, the highest activation barrier is shown by 1-CN–8-CN system at 21.4 kcal mol^−1^, that is governed by the greater steric clashes between the adjacent nitrile groups on the transition structure. The clashing of these substituents outweighs the electronic properties, that lead to the destabilization of the transition state by 4.4 kcal mol^−1^ from the unsubstituted tetraene.

#### 2.3.2 2,*Y*-Disubstituted 1,3,5,7-octatetraenes (*Y* = 3–7)

Similar energy trends are observed for the 2,3-disubstituted with the 1,2-disubstituted system, where they are driven by the clashing of the vicinal moieties. Mild destabilization of the transition structure caused by the steric congestion of the adjacent nitro groups in 2-NO_2_–3-NO_2_ compound are recorded at 19.0 kcal mol^−1^, that is 2.0 kcal mol^−1^ higher than the parent molecule (Table 5). As discussed for the monosubstitution systems, location of substituents on the tetraene skeleton are shown to exhibit an essential electronic effect on the rate of the reaction. Therefore, an insignificant variation between the computed results is shown between the studied difunctionalised 2,4-, 2,5- and 2,6 compounds. As opposed to the unsubstituted compound, a measurable decrease in the activation energies of these difunctionalised system are shown to effectively influenced by the presence of substituents with strong electron withdrawing characters as represented by the bold values in Table 5.

**Table 5:** Activation energies (Δ*G*^‡^, kcal mol^−1^) using M06-2X/6-31+G(d) for electrocyclic ring-closure of variously functionalised 2,*Y*-disubstituted octatetraenes (*Y* = 3–7)

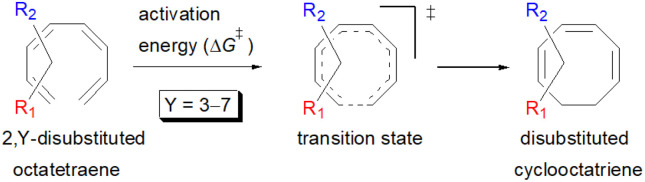									
			R_2_						
			CH_3_	NH_2_	OH	F	C(O)H	CN	NO_2_
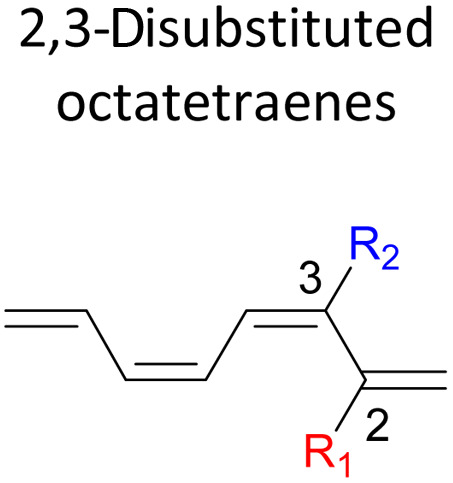	R_1_	CH_3_	14.4	16.4	14.9	14.3	17.0	12.7	17.3
		NH_2_	17.8	18.1	15.2	14.7	16.9	13.9	16.9
		OH	17.4	17.1	19.4	18.3	15.4	18.3	11.9
		F	17.0	17.1	18.8	17.8	15.4	18.6	15.2
		C(O)H	16.1	15.6	13.6	12.3	16.3	13.2	15.0
		CN	14.0	12.9	15.0	13.8	14.5	15.9	11.5
		NO_2_	17.9	13.3	16.4	14.8	15.5	16.3	19.0
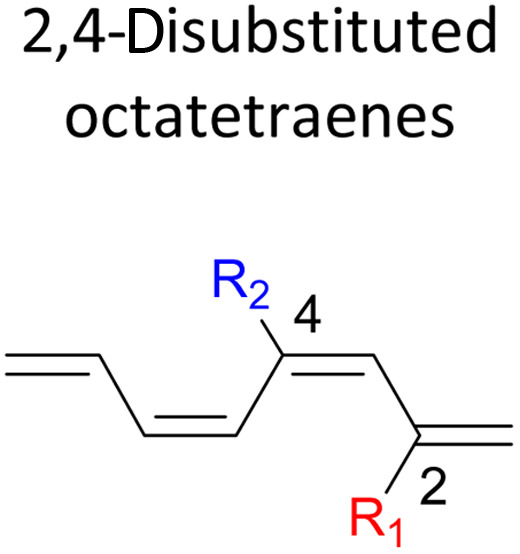	R_1_	CH_3_	13.2	15.6	14.2	12.9	12.2	13.3	13.5
		NH_2_	15.2	17.0	15.9	13.8	13.3	14.7	13.6
		OH	15.3	17.2	16.8	16.4	13.1	14.2	13.0
		F	13.7	16.8	16.1	14.1	12.1	13.7	12.4
		C(O)H	11.4	13.5	12.3	**9.9**	**8.1**	10.6	**8.5**
		CN	12.6	15.2	14.9	13.4	**9.8**	12.6	11.1
		NO_2_	12.5	12.7	12.1	**9.4**	**5.7**	**9.5**	**9.2**
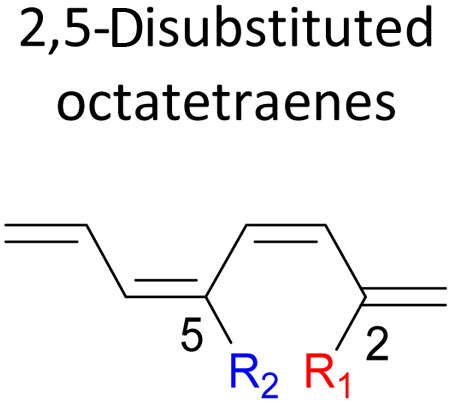	R_1_	CH_3_	15.7	16.2	14.3	11.1	11.9	12.8	13.1
		NH_2_	17.1	17.5	15.2	12.5	13.7	12.0	12.5
		OH	15.7	17.4	14.0	11.7	13.9	14.2	13.9
		F	14.4	15.5	13.8	11.3	12.6	13.2	12.9
		C(O)H	11.8	13.1	11.8	**9.2**	**9.3**	10.4	**8.5**
		CN	14.7	12.3	12.7	**10.0**	10.7	12.0	12.3
		NO_2_	**9.7**	**9.2**	11.2	**8.5**	**7.2**	10.5	**6.3**
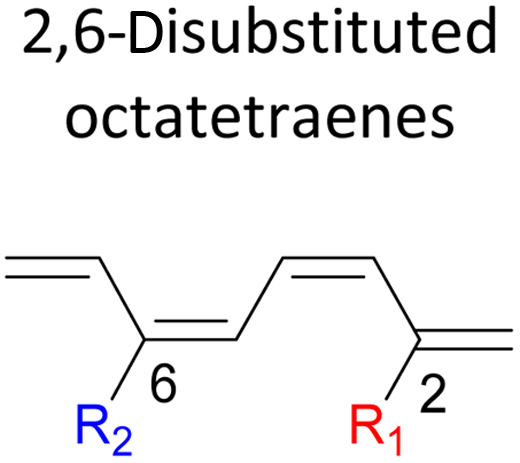	R_1_	CH_3_	15.8	15.6	14.7	14.5	11.7	15.4	11.2
		NH_2_	16.5	16.5	17.0	15.1	12.8	15.5	11.3
		OH	15.6	18.3	19.9	18.0	13.3	18.0	13.9
		F	17.0	17.9	16.3	15.9	13.1	18.7	12.5
		C(O)H	13.2	13.8	13.5	11.6	**8.4**	11.7	**7.3**
		CN	15.9	16.3	12.6	13.1	11.5	14.9	**9.5**
		NO_2_	10.9	12.4	12.7	10.6	**7.5**	10.9	**6.6**
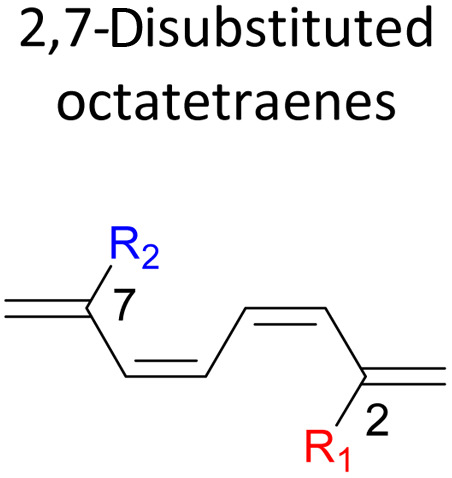	R_1_	CH_3_	11.0	13.1	13.5	13.2	**8.4**	10.4	**6.9**
		NH_2_		15.3	16.1	15.0	**9.3**	11.4	**7.7**
		OH			17.8	15.6	10.2	13.2	**8.6**
		F				15.7	10.2	12.1	**8.9**
		C(O)H					**7.5**	9.4	**5.8**
		CN						11.4	**9.0**
		NO_2_							**4.9**

A remarkably lower activation energy of 6.3 kcal mol^−1^ is computed by the 2-NO_2_–5-NO_2_ octatetraene (Table 5). In the case of 2,7-disubstitution pattern, a much more prominent decrease in the reaction energies is observed which is the most accelerated transformation in this investigation. The lowest activation barrier is computed for 2-NO_2_–7-NO_2_ at 4.9 kcal mol^−1^ compared to the unsubstituted parent molecule (17.0 kcal mol^−1^). In general, any substituent attached at these positions (C2 and C7) offer maximized electronic contribution towards the formation of the new terminal σ-bond. Thus, a decrease in activation barriers of the difunctionalised octatetraene from 17.0 kcal mol^−1^ are depicted. In addition, an increase in the reactivity *via* lower barrier of certain substitutional pattern with the involvement of opposite electronic nature, presumably arises as a result of the synergistic effect between the attached substituents. For example, the 2-CH_3_–7-NO_2_, 2-NH_2_–7-NO_2_ and 2-OH–7-NO_2_ reveals an apparent decrease in the energy barriers to 6.9 kcal mol^−1^, 7.7 kcal mol^−1^, and 8.6 kcal mol^−1^, respectively, contrary to 17.0 kcal mol^−1^ for unsubstituted tetraene.

#### 2.3.3 3,*Z*-Disubstituted 1,3,5,7-octatetraenes (*Z* = 4–6)

The 3,4-disubstitution systems (Table 6) display similar outcomes as exhibited by the 1,2- (Table 4) and 2,3-substrates (Table 5), whereby the mild steric interaction between the adjacent side groups govern the overall reaction. In regards to the position of the attachment on the tetraene backbone, minimal electronic effect is involved in the organic transformation to the corresponding substituted cyclooctatriene molecule. Therefore, an even higher activation barrier is noted for 3-NO_2_–4-NH_2_ compound at 22.8 kcal mol^−1^ than its corresponding 1,2-disubstituted (12.4 kcal mol^−1^), and 2,3-disubstituted compound (13.3 kcal mol^−1^).

**Table 6:** Activation energies (Δ*G*^‡^, kcal mol^−1^) using M06-2X/6-31+G(d) for electrocyclic ring-closure of variously functionalised 3,*Z*-disubstituted (*Z* = 4–6) octatetraenes

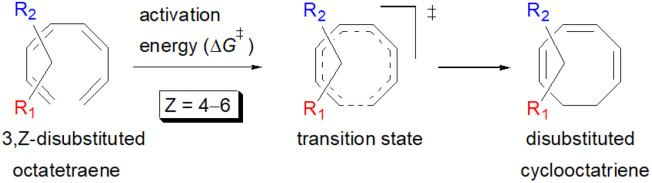									
			R_2_						
			CH_3_	NH_2_	OH	F	C(O)H	CN	NO_2_
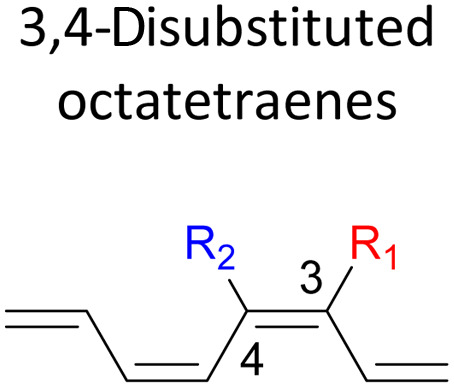	R_1_	CH_3_	19.2	20.0	18.7	16.6	17.2	18.5	16.4
		NH_2_	19.3	17.3	18.5	17.1	17.5	19.7	*22.1*
		OH	17.6	19.3	18.8	16.6	16.4	17.0	15.4
		F	16.8	18.4	17.6	16.0	15.1	15.9	14.7
		C(O)H	16.0	17.8	16.3	13.9	11.8	13.9	11.9
		CN	18.7	*21.1*	18.9	17.1	14.3	16.5	14.9
		NO_2_	16.3	*22.8*	10.1	15.0	13.0	15.2	12.1
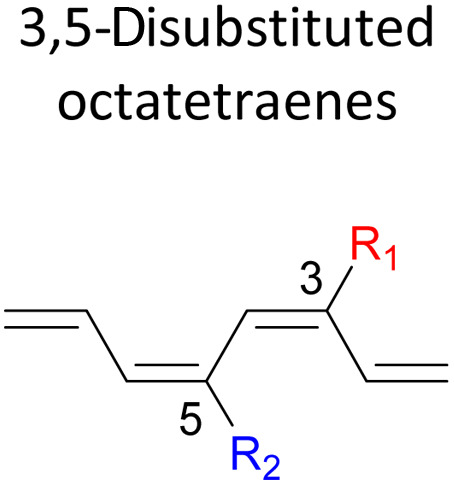	R_1_	CH_3_	17.2	19.2	19.1	16.8	16.2	17.1	16.2
		NH_2_	17.5	20.8	19.0	16.7	16.6	18.0	16.5
		OH	16.8	17.6	18.5	16.4	15.3	17.6	16.2
		F	16.6	17.8	17.2	15.7	14.4	17.1	15.2
		C(O)H	14.5	16.1	13.7	11.8	11.9	12.8	10.6
		CN	16.3	18.0	18.3	16.3	14.7	15.5	14.8
		NO_2_	12.8	13.1	14.3	**9.8**	10.7	12.8	10.4
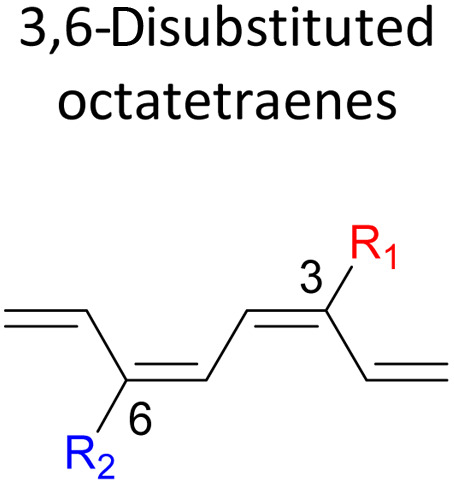	R_1_	CH_3_	18.4	19.9	20.0	18.7	16.1	18.4	15.5
		NH_2_		20.8	19.7	19.1	15.9	19.5	16.5
		OH			19.4	18.0	14.8	18.1	14.5
		F				17.6	14.3	17.1	12.5
		C(O)H					12.0	15.0	**9.3**
		CN						17.5	14.0
		NO_2_							**9.5**

In the event of 3,5- and 3,6- patterns, only few combinations reveal a noticeable decrease in the reaction rate, as shown when nitro group is/are incorporated (Table 6). This could be explained in terms of the high electron accepting capacity that is strong enough to facilitate the 8π electrocyclic ring closure. On the other hand, the reaction reactivity for the other attachments is shown to be insignificantly affected by the electronic properties, as shown by the trivial variation from the unfunctionalised octatetraene.

#### 2.3.4 4,5-Disubstituted 1,3,5,7-octatetraenes

A decreased in the stabilizing effect on the transition structure of the 4,5-disubstituted octatetraene (Table 7) is as a result of similar steric effect between the neighbouring functionalities, as experienced by the 1,2- (Table 4), 2,3- (Table 5) and 3,4-cases (Table 6). However, a much severe steric congestion is experience by this substitution, as shown by the greater increased in the activation barrier to 25.4 kcal mol^−1^ of the 4-CH_3_–5-CH_3_ substituted octatetraene from the other corresponding vicinal dimethyl substitution (Table 7).

**Table 7:** Activation energies (Δ*G*^‡^, kcal mol^−1^) using M06-2X/6-31+G(d) for electrocyclic ring-closure of variously functionalised 4,5-disubstituted octatetraenes

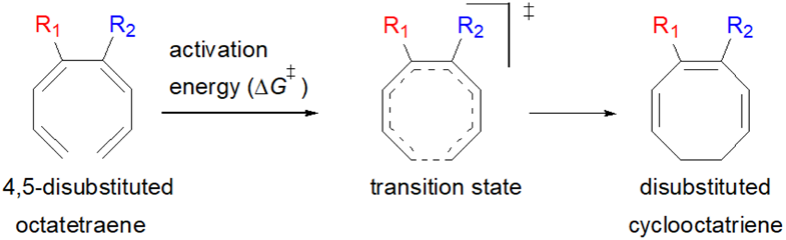								
		R_2_						
		CH_3_	NH_2_	OH	F	CHO	CN	NO_2_
R_1_	CH_3_	*25.4*	*23.5*	*21.8*	15.5	*22.9*	20.4	*23.6*
	NH_2_		*21.0*	20.5	17.6	19.8	18.1	*22.5*
	OH			*21.0*	18.8	18.2	17.6	18.1
	F				15.8	16.1	16.3	18.7
	CHO					*21.1*	18.9	*21.0*
	CN						20.1	*22.3*
	NO_2_							*23.5*

**Fig. 4:**
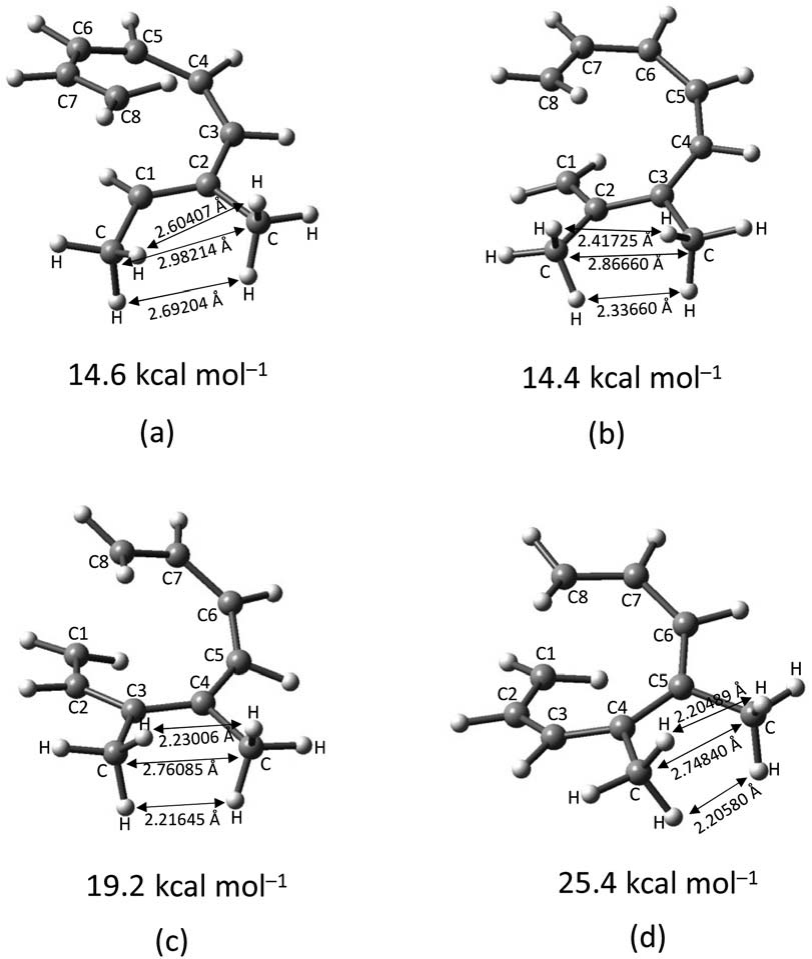
Optimized transition structures of (a) 1-CH_3_–2-CH_3_-, (b) 2-CH_3_–3-CH_3_-, (c) 3-CH_3_–4-CH_3_-, and (d) 4-CH_3_–5-CH_3_-disubstituted octatetraene. Interatomic bond distance between vicinal methyl groups.

The transition structure of the 4,5-dimethyl substitution features relatively shorter interatomic distances between the adjacent functional groups than the 1,2-dimethyl substitution (Fig. 4). In accordance to the position of the attached functional groups, the rate of the electrocyclisation reaction is dominantly controlled by steric effect and considerably unaffected by the electronic characters of the substituents, as demonstrated by the increased in the activation energies from 17.0 kcal mol^−1^ (unsubstituted molecule).

In the event of the vicinal attachment involving strongest electron donating group (–NH_2_), similar trend of increasing activation barriers is observed (from 1,2- to 4,5-disubstitution). However, it is as a result of the decreasing electronic effect on the 8π electrocyclic ring closure in regards to their position on the tetraene skeleton.

Moreover, reactivity trend involving –NO_2_ substitution (the strongest electron withdrawing group) also shows a general increase in the activation energies. However, an anomaly is identified whereby the 3-NO_2_–4-NO_2_-substituted octatetraene display an opposite effect (decrease in activation energy to 12.1 kcal mol^−1^). Presumably, extra stabilization of the 3,4-disubstitution favoring the electrocyclisation arise from the greater resonance structures at the transition states, compared to the 2,3- and 4,5-disubstituted octatetraene transition structures. Further investigation is underway to explore the crucial aspects pertinent to 8π electrocyclization of 1,3,5,7-octatetraene motifs.

## 3. Conclusions

Salient conclusions drawn from this computational study towards 8π electrocyclic ring closure of conjugated 1,3,5,7-octatetraenes include:

(a) An extensive benchmarking studies reveal that M06-2X/6-31+G(d) method is one of the appropriate DFT methods for this investigation. This was supported by the accurate computed value with the measured activation energy (17.0 kcal mol^−1^) of the 8π electrocyclisation of 1,3,5,7-octatetraenes.

(b) Monosubstituted 1,3,5,7-octatetraenes shown to selectively prefer the transition structure with an outward substituent over the inward. The lower steric clashes stabilize the transition structure towards the 8π ring closure process. However, the opposite effects are also observed for some of the mesomerically electron donating groups (–NH_2_ and –OH). The SOI between the substituent lone pair and 
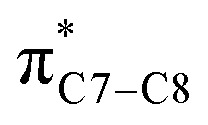
 orbital results in the extra stabilization to the inward transition structure. Therefore, this exceptional case depends strongly on the capacity of the electron donating characters. Amidst the four possible monosubstitution patterns, attachment at C2 position displayed a more pronounced effect on the acceleration of the electrocyclic process due to greater electronic contributions with the terminal carbon double bonds. Therefore, they enhanced the orbital interaction for the generation of new σ-bond between the C1 and C8 atom. Contrary to the unfunctionalized 1,3,5,7-octatetraenes molecule, a single substitution does not result much impact on the efficiency of the 8π electrocyclic ring closure of studied tetraenes.

(c) In the case of disubstitution systems, attachments on the terminal double bonds renders a general reduction in the activation energies. This is due to the greater electronic interaction from both substituents that stabilizes the transition structure. Particularly, the involvement of strong electron withdrawing group (–NO_2_) often proceeds with reduced energy barriers. A remarkable decrease in the activation barrier up to 4.9 kcal mol^−1^ from 17.0 kcal mol^−1^ (parent molecule) is observed for 2-NO_2_–7-NO_2_ system. Moreover, the synergistic effect between the attached substituents of opposite electronic nature in a disubstituted environment also reveals an apparent accelerated effect on the reactivity of the functionalised tetraene molecule. For example, a decrease in the activation barrier to 7.4 kcal mol^−1^ is observed in the 1-NH_2_–8-NO_2_-disubstituted octatetraene.

(d) In the event of vicinal substitution (1,2-, 2,3-, 3,4-, and 4,5-systems), the eight electron conrotatory electrocyclisation reaction is disfavoured by the destabilization of the transition state, as a result of the sterically congested vicinal functional group arrangements. Greatest decelerated effect is observed by the 4-CH_3_–5-CH_3_ substituted octatetraene at 25.4 kcal mol^−1^, which is 8.4 kcal mol^−1^ higher than the unsubstituted compound (17.0 kcal mol^−1^). A much severe steric clashes are observed in the 4,5-pattern over the other vicinal disubstituted patterns because of the less important electronic effect of the substituents, and the greater steric clashes experienced between the adjacent side groups.

## 4. Computational methods

Density functional theory (DFT)^22^ calculations were performed using Gaussian 16 (revision C.01)^23^ and the GaussView (Version 6)^24^ was used to generate input geometries and visualize output structures. For the calculations related to the comparative benchmarking study for the electrocyclisation of unsubstituted octatetraene, hybrid three-parameter functional commonly known as B3LYP^25^ and metahybrid functional M0-62X^26^ levels of theory with different basis sets were used to locate all the stationary points involved. Both B3LYP/6-31G(d)^15^ and M0-62X/6-31+G(d,p)^18^ levels of theory have already been reported for the 8π electrocyclisation of tetraenes. Geometry optimization for mono- and disubstituted octatetraenes were performed using the M0-62X functional with the 6-31+G(d) basis set, followed by the frequency calculations at the same level of theory. All stationary points were characterized as minima or transition structures based on normal vibrational mode analysis. Thermal corrections were computed from unscaled frequencies, assuming a standard state of 298.15 K and 1 atm. Representative transition states were also linked to their corresponding minima through intrinsic reaction coordinate (IRC) calculations.^27^ All-*s*-transoid conformations of the octatetraenes have been used to calculate the energies of the reactants. For the substituents (–OH and –CHO) exhibiting more than one conformation, lowest energy conformers have been provided here.

## Author contributions

Nur Hazimah B. Z. Arfan: data curation, formal analysis, investigation, methodology, validation, visualization, writing – original draft, writing – review & editing. Malai Haniti S. A. Hamid: funding acquisition, resources, software, co-supervision, writing – review & editing. Nadeem S. Sheikh: conceptualization, methodology, funding acquisition, project administration, resources, software, supervision, writing – original draft, writing – review & editing.

## Conflicts of interest

There are no conflicts of interest to declare.

## Supplementary Material

RA-013-D3RA05127G-s001
