# Supplementary material for: A computational investigation towards substitution effects on 8π electrocyclisation of conjugated 1,3,5,7-octatetraenes
Source: RSC Adv. 2023 Oct 20;13(44):30787–97. doi: 10.1039/d3ra05127g (PMC10587816; doi:10.1039/d3ra05127g)
Supplement: RA-013-D3RA05127G-s001 [file RA-013-D3RA05127G-s001.pdf]

Electronic Supplementary Material (ESI) for RSC Advances.
This journal is © The Royal Society of Chemistry 2023

Electronic Supplementary Information

A computational investigation towards substitution effects on 8π electrocyclisation of conjugated 1,3,5,7-octatetraenes

Nur Hazimah B. Z. Arfan[a], *Malai Haniti S. A. Hamid*[a], *Nadeem S. Sheikh*[a]*

[a]*Chemical Sciences, Faculty of Science, Universiti Brunei Darussalam, Jalan Tungku Link Gadong BE 1410, Brunei Darussalam*
*Email: nadeem.sheikh@ubd.edu.bn

Table of Contents

Electronic Supplementary Information 1
1. Complete citation for reference 23 3
2. Energies for unsubstituted system 3
 2.1 Octatetraene (B3LYP) 3
 2.2 Octatetraene (M06-2X) 4
3. Energies for monosubstituted system 4
 3.1 1-substituted (outward) 4
 3.2 1-substituted (inward) 5
 3.3 2-substituted 6
 3.4 3-substituted 7
 3.5 4-substituted 8
4. Energies for disubstituted system 9
 4.1 1,2-substituted 10
 4.2 1,3-substituted 12
 4.3 1,4-substituted 15
 4.4 1,5-substituted 18
 4.5 1,6-substituted 21
 4.6 1,7-substituted 24
 4.7 1,8-substituted 27
 4.8 2,3-substituted 29
 4.9 2,4-substituted 32
 4.10 2,5-substituted 35
 4.11 2,6-substituted 38
 4.12 2,7-substituted 41
 4.13 3,4-substituted 43
 4.14 3,5-substituted 46
 4.15 3,6-substituted 48
 4.16 4,5-substituted 50
5. Cartesian coordinates of transition structure 52
 5.1 Octatetraene 52

5.1.1 Helical-shaped transition structure....52
5.1.2 Boat-shaped transition structure....53

5.2 Monosubstituted....53
5.2.1 1-substituted (outward)....53
5.2.2 1-substituted (inward)....56
5.2.3 2-substituted....59
5.2.4 3-substituted....63
5.2.5 4-substituted....66

5.3 Disubstituted....69
5.3.1 1,2-substituted....69
5.3.2 1,3-substituted....94
5.3.4 1,4-substituted....118
5.3.5 1,5-substituted....143
5.3.6 1,6-substituted....167
5.3.7 1,7-substituted....191
5.3.8 1,8-substituted....215
5.3.9 2,3-substituted....228
5.3.10 2,4-substituted....251
5.3.11 2,5-substituted....276
5.3.12 2,6-substituted....299
5.3.13 2,7-substituted....323
5.3.14 3,4-substituted....336
5.3.15 3,5-substituted....360
5.2.16 3,6-substituted....384
5.3.17 4,5-substituted....398

1. **Complete citation for reference 23**

Gaussian 16, Revision C.01, M. J. Frisch, G. W. Trucks, H. B. Schlegel, G. E. Scuseria, M. A. Robb, J. R. Cheeseman, G. Scalmani, V. Barone, G. A. Petersson, H. Nakatsuji, X. Li, M. Caricato, A. V. Marenich, J. Bloino, B. G. Janesko, R. Gomperts, B. Mennucci, H. P. Hratchian, J. V. Ortiz, A. F. Izmaylov, J. L. Sonnenberg, D. Williams-Young, F. Ding, F. Lipparini, F. Egidi, J. Goings, B. Peng, A. Petrone, T. Henderson, D. Ranasinghe, V. G. Zakrzewski, J. Gao, N. Rega, G. Zheng, W. Liang, M. Hada, M. Ehara, K. Toyota, R. Fukuda, J. Hasegawa, M. Ishida, T. Nakajima, Y. Honda, O. Kitao, H. Nakai, T. Vreven, K. Throssell, J. A. Montgomery, Jr., J. E. Peralta, F. Ogliaro, M. J. Bearpark, J. J. Heyd, E. N. Brothers, K. N. Kudin, V. N. Staroverov, T. A. Keith, R. Kobayashi, J. Normand, K. Raghavachari, A. P. Rendell, J. C. Burant, S. S. Iyengar, J. Tomasi, M. Cossi, J. M. Millam, M. Klene, C. Adamo, R. Cammi, J. W. Ochterski, R. L. Martin, K. Morokuma, O. Farkas, J. B. Foresman, and D. J. Fox, Gaussian, Inc., Wallingford CT, 2019.

2. **Energies for unsubstituted system**

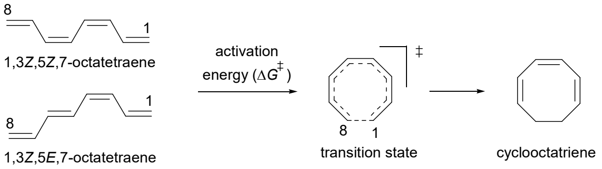

2.1 Octatetraene (B3LYP)

Octatetraene [Starting Material], (Hartree)					
Entry	Basis Set	Total Electronic Energy	Sum of Electronic and Zero-Point Energies	Sum of Electronic and Thermal Enthalpies	Gibbs Free Energy
1	6-31G(d)	-310.7992541 (-310.802614)	-310.6460140 (-310.649570)	-310.6362600 (-310.639816)	-310.6792760 (-310.683320)
2	6-31G+(d)	-310.8143824 (-310.8175926)	-310.6615370 (-310.665035)	-310.6517570 (-310.655236)	-310.6948610 (-310.698864)
3	6-31G(d,p)	-310.8151320 (-310.8184987)	-310.6622150 (-310.665771)	-310.6524460 (-310.656010)	-310.6955120 (-310.699526)
4	6-31G+(d,p)	-310.8303415 (-310.8335417)	-310.6778310 (-310.681309)	-310.6680320 (-310.671495)	-310.7111870 (-310.715155)

Transition State, (Hartree)					
Entry	Basis Set	Total Electronic Energy	Sum of Electronic and Zero-Point Energies	Sum of Electronic and Thermal Enthalpies	Gibbs Free Energy
1	6-31G(d)	-310.7736697 (-310.7275109)	-310.619182 (-310.575506)	-310.611142 (-310.567369)	-310.650172 (-310.606328)
2	6-31G+(d)	-310.7877014 (-310.742398)	-310.63349 (-310.590800)	-310.625449 (-310.582615)	-310.664473 (-310.621676)
3	6-31G(d,p)	-310.7894664 (-310.7435959)	-310.635337 (-310.591949)	-310.627291 (-310.583798)	-310.666327 (-310.622778)
4	6-31G+(d,p)	-310.8033777 (-310.7583815)	-310.649528 (-310.607149)	-310.641479 (-310.598944)	-310.680511 (-310.638036)

Cyclooctatetriene [Product], (Hartree)					
Entry	Basis Set	Total Electronic Energy	Sum of Electronic and Zero-Point Energies	Sum of Electronic and Thermal Enthalpies	Gibbs Free Energy
1	6-31G(d)	-310.8118501	-310.6543740	-310.6464540	-310.6853160
2	6-31G+(d)	-310.8244492	-310.6673760	-310.6594350	-310.6983340
3	6-31G(d,p)	-310.8270269	-310.6699730	-310.6620390	-310.7009240
4	6-31G+(d,p)	-310.8393865	-310.6827480	-310.6747900	-310.7137150

Note: Computed energies (Hartree) for conrotatory and disrotatory 8π electrocyclic ring-closure of 1,3Z,5Z,7-octatetraene *via* helical-shaped TS and for 1,3*Z*,5*E*,7-octatetraene *via* boat-shaped TS (in parentheses) respectively, using B3LYP functional.

2.2 Octatetraene (M06-2X)

Octatetraene [Starting Material], (Hartree)					
Entry	Basis Set	Total Electronic Energy	Sum of Electronic and Zero-Point Energies	Sum of Electronic and Thermal Enthalpies	Gibbs Free Energy
1	6-31G(d)	-310.6347999 (-310.6376588)	-310.47992 (-310.483068)	-310.47021 (-310.473314)	-310.513284 (-310.516854)
2	6-31G+(d)	-310.6466442 (-310.6493868)	-310.492255 (-310.495336)	-310.482447 (-310.485503)	-310.526022 (-310.529303)
3	6-31G(d,p)	-310.647605 (-310.650484)	-310.493169 (-310.496341)	-310.483452 (-310.486569)	-310.526545 (-310.530164)
4	6-31G+(d,p)	-310.6595008 (-310.6622439)	-310.505579 (-310.508656)	-310.495747 (-310.498790)	-310.539465 (-310.542698)

Transition State, (Hartree)					
Entry	Basis Set	Total Electronic Energy	Sum of Electronic and Zero-Point Energies	Sum of Electronic and Thermal Enthalpies	Gibbs Free Energy
1	6-31G(d)	-310.6121088 (-310.567316)	-310.456417 (-310.413495)	-310.448417 (-310.405542)	-310.487321 (-310.444105)
2	6-31G+(d)	-310.6232859 (-310.5790775)	-310.46797 (-310.425711)	-310.459938 (-310.417710)	-310.498913 (-310.456369)
3	6-31G(d,p)	-310.624771 (-310.5801686)	-310.469543 (-310.426830)	-310.461545 (-310.418868)	-310.500438 (-31.457443)
4	6-31G+(d,p)	-310.6358264 (-310.5918)	-310.480965 (-310.438924)	-310.472935 (-310.430908)	-310.511893 (-310.460589)

Cyclooctatetriene [Product], (Hartree)					
Entry	Basis Set	Total Electronic Energy	Sum of Electronic and Zero-Point Energies	Sum of Electronic and Thermal Enthalpies	Gibbs Free Energy
1	6-31G(d)	-310.659532	-310.500516	-310.492655	-310.531342
2	6-31G+(d)	-310.6695176	-310.510921	-310.503037	-310.541761
3	6-31G(d,p)	-310.6715965	-310.513147	-310.505275	-310.543973
4	6-31G+(d,p)	-310.6813312	-310.523301	-310.515402	-310.554146

Note: Computed energies (Hartree) for conrotatory and disrotatory 8π electrocyclic ring-closure of 1,3*Z*,5*Z*,7-octatetraene *via* helical-shaped TS and for 1,3*Z*,5*E*,7-octatetraene *via* boat-shaped TS (in parentheses) respectively, using M06-2X functional.

3. Energies for monosubstituted system

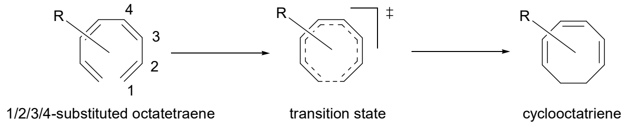

3.1 1-substituted (outward)

Octatetraene [Starting Material], (Hartree)					
Entry	R	Total Electronic Energy	Sum of Electronic and Zero-Point Energies	Sum of Electronic and Thermal Enthalpies	Gibbs Free Energy
1	CH_3	-349.9451704 (-349.9599514)	-349.762745 (-349.778185)	-349.751272 (-349.766689)	-349.800777 (-349.816613)
2	NH_2	-365.9853984 (-366.0032139)	-365.813549 (-365.831912)	-365.802451 (-365.820753)	-365.8497100 (-365.868049)
3	OH	-385.8448401 (-385.8600895)	-385.685698 (-385.701170)	-385.675672 (-385.690270)	-385.7199600 (-385.737015)
4	F	-409.8578234 (-409.8695132)	-409.711304 (-409.723437)	-409.701514 (-409.713627)	-409.7456260 (-409.757804)
5	CHO	-423.935885	-423.771711	-423.760869	-423.8075750

		(-423.9485292)	(-423.784877)	(-423.774006)	(-423.820810)
6	CN	-402.8671404<br>(-402.8787128)	-402.713782<br>(-402.725820)	-402.702282<br>(-402.714288)	-402.7516770<br>(-402.763871)
7	NO_2	-515.0833488<br>(-515.0949929)	-514.925328<br>(-514.937408)	-514.913232<br>(-514.925299)	-514.9642870<br>(-514.976360)

Transition State, (Hartree)					
Entry	R	Total Electronic Energy	Sum of Electronic and Zero-Point Energies	Sum of Electronic and Thermal Enthalpies	Gibbs Free Energy
1	CH_3	-349.921311<br>(-349.9358089)	-349.73729<br>(-349.752425)	-349.727769<br>(-349.742898)	-349.770133<br>(-349.785262)
2	NH_2	-365.9594694<br>(-365.976968)	-365.786617<br>(-365.804791)	-365.777297<br>(-365.795348)	-365.819071<br>(-365.837332)
3	OH	-385.8205407<br>(-385.837376)	-385.660058<br>(-385.677181)	-385.651071<br>(-385.668193)	-385.692383<br>(-385.709499)
4	F	-409.8348242<br>(-409.8462151)	-409.687028<br>(-409.698873)	-409.678243<br>(-409.690081)	-409.719367<br>(-409.731213)
5	CHO	-423.9119541<br>(-423.923224)	-423.746413<br>(-423.758160)	-423.736513<br>(-423.748153)	-423.780432<br>(-423.792344)
6	CN	-402.8436267<br>(-402.8548999)	-402.688991<br>(-402.700714)	-402.679333<br>(-402.691050)	-402.72264<br>(-402.734362)
7	NO_2	-515.0611812<br>(-515.0725215)	-514.902119<br>(-514.913907)	-514.891687<br>(-514.903472)	-514.937376<br>(-514.949154)

Cyclooctatriene [Product], (Hartree)					
Entry	R	Total Electronic Energy	Sum of Electronic and Zero-Point Energies	Sum of Electronic and Thermal Enthalpies	Gibbs Free Energy
1	CH_3	-349.9647373<br>(-349.9786163)	-349.777731<br>(-349.792308)	-349.768543<br>(-349.783102)	-349.8101930<br>(-349.824782)
2	NH_2	-365.9980677<br>(-366.0144252)	-365.821834<br>(-365.838767)	-365.812832<br>(-365.829738)	-365.8541080<br>(-365.871054)
3	OH	-385.8635893<br>(-385.8796379)	-385.700225<br>(-385.716704)	-385.691319<br>(-385.707767)	-385.7325360<br>(-385.749042)
4	F	-409.8840559<br>(-409.8948483)	-409.732963<br>(-409.744280)	-409.724417<br>(-409.735715)	-409.7650450<br>(-409.776377)
5	CHO	-423.9488377<br>(-423.9604803)	-423.781317<br>(-423.792894)	-423.771435<br>(-423.782996)	-423.815605<br>(-423.827348)
6	CN	-402.8839501<br>(-402.8947723)	-402.726409<br>(-402.737723)	-402.716890<br>(-402.728191)	-402.7598750<br>(-402.771196)
7	NO_2	-515.1064451<br>(-515.1173435)	-514.944362<br>(-514.955774)	-514.934203<br>(-514.945601)	-514.9795140<br>(-514.990929)

Note: Computed energies (Hartree) for 8π electrocyclic ring-closure of 1,3,5,7-octatetraene using M06-2X/6-31+G(d) and M06-2X/6-31+G(d,p) (in parentheses).

3.2 1-substituted (inward)

Octatetraene [Starting Material], (Hartree)					
Entry	R	Total Electronic Energy	Sum of Electronic and Zero-Point Energies	Sum of Electronic and Thermal Enthalpies	Gibbs Free Energy
1	CH_3	-349.9428887<br>(-349.9576975)	-349.760592<br>(-349.776017)	-349.749047<br>(-349.764478)	-349.7984050<br>(-349.813688)
2	NH_2	-365.98451<br>(-366.0023442)	-365.812373<br>(-365.830713)	-365.801297<br>(-365.819599)	-365.8486450<br>(-365.867005)
3	OH	-385.8441906<br>(-385.8607759)	-385.684434<br>(-385.701947)	-385.673722<br>(-385.690965)	-385.7204080<br>(-385.738404)
4	F	-409.8586081<br>(-409.8703154)	-409.712234<br>(-409.724363)	-409.702432<br>(-409.714555)	-409.747448<br>(-409.759524)
5	CHO	-423.9310747	-423.766996	-423.756116	423.803684

		(-423.9437075)	(-423.779958)	(-423.768208)	(-423.818024)
6	CN	-402.8674395<br>(-402.8790409)	-402.714196<br>(-402.726224)	-402.703590<br>(-402.715611)	-402.7504780<br>(-402.762435)
7	NO_2	-515.079366<br>(-515.0910253)	-514.921728<br>(-514.933819)	-514.910375<br>(-514.922461)	-514.9598880<br>(-514.971973)

Transition State, (Hartree)					
Entry	R	Total Electronic Energy	Sum of Electronic and Zero-Point Energies	Sum of Electronic and Thermal Enthalpies	Gibbs Free Energy
1	CH_3	-349.9175407<br>(-349.9320751)	-349.733086<br>(-349.748285)	-349.723824<br>(-349.739005)	-349.765383<br>(-349.780596)
2	NH_2	-365.9651042<br>(-365.9821745)	-365.79139<br>(-365.808920)	-365.782524<br>(-365.800052)	-365.823116<br>(-365.840633)
3	OH	-385.8259145<br>(-385.842474)	-385.665<br>(-385.681874)	-385.656302<br>(-385.673175)	-385.69666<br>(-385.713532)
4	F	-409.8334281<br>(-409.8447524)	-409.685255<br>(-409.697024)	-409.676711<br>(-409.688472)	-409.71699<br>(-409.728760)
5	CHO	-423.9069521<br>(-423.9179264)	-423.741341<br>(-423.752827)	-423.731472<br>(-423.743069)	-423.775227<br>(-423.786057)
6	CN	-402.8401109<br>(-402.8513614)	-402.685662<br>(-402.697367)	-402.676107<br>(-402.687806)	-402.718722<br>(-402.730425)
7	NO_2	-515.0552824<br>(-515.0665621)	-514.896222<br>(-514.907941)	-514.886015<br>(-514.897731)	-514.930468<br>(-514.942177)

Cyclooctatriene [Product], (Hartree)					
Entry	R	Total Electronic Energy	Sum of Electronic and Zero-Point Energies	Sum of Electronic and Thermal Enthalpies	Gibbs Free Energy
1	CH_3	-349.9647373<br>(-349.9786163)	-349.777731<br>(-349.792308)	-349.768543<br>(-349.783102)	-349.8101930<br>(-349.824782)
2	NH_2	-365.9980677<br>(-366.0144252)	-365.821834<br>(-365.838767)	-365.812832<br>(-365.829738)	-365.8541080<br>(-365.871054)
3	OH	-385.8635893<br>(-385.8796379)	-385.700225<br>(-385.716704)	-385.691319<br>(-385.707767)	-385.7325360<br>(-385.749042)
4	F	-409.8840559<br>(-409.8948483)	-409.732963<br>(-409.744280)	-409.724417<br>(-409.735715)	-409.7650450<br>(-409.776377)
5	CHO	-423.9488377<br>(-423.9604803)	-423.781317<br>(-423.792894)	-423.771435<br>(-423.782996)	-423.815605<br>(-423.827348)
6	CN	-402.8839501<br>(-402.8947723)	-402.726409<br>(-402.737723)	-402.716890<br>(-402.728191)	-402.7598750<br>(-402.771196)
7	NO_2	-515.1064451<br>(-515.1173435)	-514.944362<br>(-514.955774)	-514.934203<br>(-514.945601)	-514.9795140<br>(-514.990929)

Note: Computed energies (Hartree) for 8π electrocyclic ring-closure of 1,3,5,7-octatetraene using M06-2X/6-31+G(d) and M06-2X/6-31+G(d,p) (in parentheses).

3.3 2-substituted

Octatetraene [Starting Material], (Hartree)					
Entry	R	Total Electronic Energy	Sum of Electronic and Zero-Point Energies	Sum of Electronic and Thermal Enthalpies	Gibbs Free Energy
1	CH_3	-349.9406453<br>(-349.95541)	-349.75733<br>(-349.772746)	-349.746327<br>(-349.761734)	-349.7940980<br>(-349.809358)
2	NH_2	-365.9818596<br>(-365.9993809)	-365.809765<br>(-365.827731)	-365.799032<br>(-365.816987)	-365.8451610<br>(-365.863129)
3	OH	-385.8449307<br>(-385.8620599)	-385.686065<br>(-385.703590)	-385.675216<br>(-385.692669)	-385.7226010<br>(-385.740532)
4	F	-409.8602516<br>(-409.8719872)	-409.713975<br>(-409.726116)	-409.704324<br>(-409.716461)	-409.7482250<br>(-409.760356)

5	CHO	-423.9264695 (-423.9391896)	-423.762433 (-423.775616)	-423.750919 (-423.764100)	-423.7997550 (-423.812916)
6	CN	-402.8604556 (-402.8720991)	-402.707042 (-402.719108)	-402.695663 (-402.707718)	-402.7446380 (-402.756692)
7	NO_2	-515.0728411 (-515.0845738)	-514.915484 (-514.927672)	-514.903445 (-514.915611)	-514.9542880 (-514.966543)

Transition State, (Hartree)					
Entry	R	Total Electronic Energy	Sum of Electronic and Zero-Point Energies	Sum of Electronic and Thermal Enthalpies	Gibbs Free Energy
1	CH_3	-349.9205965 (-349.9350441)	-349.737246 (-349.752340)	-349.727498 (-349.742594)	-349.770963 (-349.785984)
2	NH_2	-365.9598301 (-365.9772379)	-365.787469 (-365.805414)	-365.778049 (-365.795963)	-365.820149 (-365.838098)
3	OH	-385.8218248 (-385.8386702)	-385.661863 (-385.679027)	-385.661863 (-385.669963)	-385.694214 (-385.711379)
4	F	-409.8383446 (-409.8496605)	-409.690817 (-409.702584)	-409.682069 (-409.693832)	-409.723068 (-409.734829)
5	CHO	-423.9128654 (-423.9252484)	-423.747702 (-423.760586)	-423.737783 (-423.750664)	-423.781623 (-423.794494)
6	CN	-402.8427917 (-402.8540637)	-402.688363 (-402.700095)	-402.678621 (-402.690346)	-402.722064 (-402.733797)
7	NO_2	-515.0616633 (-515.0730874)	-514.902697 (-514.914561)	-514.892294 (-514.904160)	-514.938023 (-514.949862)

Cyclooctatetriene [Product], (Hartree)					
Entry	R	Total Electronic Energy	Sum of Electronic and Zero-Point Energies	Sum of Electronic and Thermal Enthalpies	Gibbs Free Energy
1	CH_3	-349.9675489 (-349.9813001)	-349.78102 (-349.795508)	-349.771603 (-349.786073)	-349.8138120 (-349.828305)
2	NH_2	-366.0084349 (-366.0250531)	-365.832706 (-365.849968)	-365.823532 (-365.840743)	-365.8651260 (-365.882414)
3	OH	-385.8720389 (-385.8881079)	-385.708835 (-385.725345)	-385.699945 (-385.716427)	-385.7410460 (-385.757572)
4	F	-409.8883096 (-409.8989117)	-409.737671 (-409.748822)	-409.729994 (-409.740183)	-409.7697740 (-409.780936)
5	CHO	-423.9613637 (-423.973059)	-423.792781 (-423.805077)	-423.783065 (-423.795343)	-423.8265050 (-423.838821)
6	CN	-402.8896555 (-402.9002001)	-402.732230 (-402.743292)	-402.722625 (-402.733675)	-402.7657840 (-402.776851)
7	NO_2	-515.1087095 (-515.1193953)	-514.946686 (-514.957915)	-514.936468 (-514.947681)	-514.9817350 (-514.992971)

Note: Computed energies (Hartree) for 8π electrocyclic ring-closure of 1,3,5,7-octatetraene using M06-2X/6-31+G(d) and M06-2X/6-31+G(d,p) (in parentheses).

3.4 3-substituted

Octatetraene [Starting Material], (Hartree)					
Entry	R	Total Electronic Energy	Sum of Electronic and Zero-Point Energies	Sum of Electronic and Thermal Enthalpies	Gibbs Free Energy
1	CH_3	-349.944538 (-349.9593215)	-349.762154 (-349.777547)	-349.75086 (-349.766263)	-349.7985320 (-349.813838)
2	NH_2	-365.9842884 (-366.0019283)	-365.812628 (-365.830745)	-365.801698 (-365.819796)	-365.8481930 (-365.866321)
3	OH	-385.8461517 (-385.8613832)	-385.687394 (-385.703015)	-385.676554 (-385.692202)	-385.723111 (-385.738652)

4	F	-409.8615425 (-409.8732058)	-409.726527 (-409.728031)	-409.706003 (-409.718126)	-409.7506980 (-409.762763)
5	CHO	-423.9328526 (-423.9433634)	-423.768903 (-423.779664)	-423.757970 (-423.768009)	-423.805244 (-423.816823)
6	CN	-402.8656483 (-402.8772657)	-402.712479 (-402.724470)	-402.700994 (-402.712983)	-402.7496790 (-402.761674)
7	NO_2	-515.0772119 (-515.0889297)	-514.919741 (-514.931904)	-514.909200 (-514.921351)	-514.9560780 (-514.968252)

Transition State, (Hartree)					
Entry	R	Total Electronic Energy	Sum of Electronic and Zero-Point Energies	Sum of Electronic and Thermal Enthalpies	Gibbs Free Energy
1	CH_3	-349.9181166 (-349.9325615)	-349.734735 (-349.749842)	-349.725024 (-349.740114)	-349.768122 (-349.783273)
2	NH_2	-365.9573047 (-365.9746758)	-365.784819 (-365.802715)	-365.775445 (-365.793313)	-365.817295 (-365.835188)
3	OH	-385.8197025 (-385.8372372)	-385.660182 (-385.677407)	-385.650963 (-385.668402)	-385.692612 (-385.709595)
4	F	-409.8383007 (-409.8495934)	-409.690928 (-409.702689)	-409.682186 (-409.693939)	-409.723058 (-409.734818)
5	CHO	-423.913238 (-423.9256419)	-423.747826 (-423.760712)	-423.737967 (-423.750859)	-423.781537 (-423.794402)
6	CN	-402.8418975 (-402.8531729)	-402.687633 (-402.699355)	-402.677878 (-402.689597)	-402.721229 (-402.732943)
7	NO_2	-515.0598415 (-515.0712428)	-514.901116 (-514.912971)	-514.890660 (-514.902508)	-514.936822 (-514.948708)

Cyclooctatriene [Product], (Hartree)					
Entry	R	Total Electronic Energy	Sum of Electronic and Zero-Point Energies	Sum of Electronic and Thermal Enthalpies	Gibbs Free Energy
1	CH_3	-349.965701 (-349.9794009)	-349.778922 (-349.793349)	-349.769526 (-349.783935)	-349.8116880 (-349.826123)
2	NH_2	-366.0008621 (-366.0174442)	-365.825483 (-365.842632)	-365.81608 (-365.833218)	-365.8582540 (-365.875368)
3	OH	-385.8662872 (-385.8817801)	-385.702976 (-385.719150)	-385.694063 (-385.710123)	-385.735222 (-385.751473)
4	F	-409.8852026 (-409.8957562)	-409.734297 (-409.745384)	-409.725707 (-409.736780)	-409.7663700 (-409.777464)
5	CHO	-423.9596747 (-423.9679286)	-423.791346 (-423.800129)	-423.781590 (-423.790331)	-423.825161 (-423.833921)
6	CN	-402.8878316 (-402.8983854)	-402.730507 (-402.741575)	-402.720869 (-402.731926)	-402.7641980 (-402.775267)
7	NO_2	-515.1065207 (-515.1172205)	-514.944904 (-514.956133)	-514.934588 (-514.945803)	-514.9803280 (-514.991565)

Note: Computed energies (Hartree) for 8π electrocyclic ring-closure of 1,3,5,7-octatetraene using M06-2X/6-31+G(d) and M06-2X/6-31+G(d,p) (in parentheses).

3.5 4-substituted

Octatetraene [Starting Material], (Hartree)					
Entry	R	Total Electronic Energy	Sum of Electronic and Zero-Point Energies	Sum of Electronic and Thermal Enthalpies	Gibbs Free Energy
1	CH_3	-349.9396727 (-349.9544471)	-349.756949 (-349.772362)	-349.745696 (-349.761122)	-349.7943360 (-349.809598)
2	NH_2	-365.9821376 (-365.9996359)	-365.81034 (-365.828381)	-365.799506 (-365.817518)	-365.8456570 (-365.863738)
3	OH	-385.8440233 (-385.8610996)	-385.68565 (-385.703106)	-385.675509 (-385.692916)	-385.7200390 (-385.737522)

4	F	-409.859808<br>(-409.8714826)	-409.713842<br>(-409.725928)	-409.704089<br>(-409.716173)	-409.7478080<br>(-409.759875)
5	CHO	-423.9298162<br>(-423.9425228)	-423.765864<br>(-423.779054)	-423.754330<br>(-423.767508)	-423.8027360<br>(-423.815935)
6	CN	-402.8617762<br>(-402.8734067)	-402.708817<br>(-402.720871)	-402.698156<br>(-402.710204)	-402.7440630<br>(-402.756115)
7	NO_2	-515.0757224<br>(-515.0874166)	-514.919037<br>(-514.931092)	-514.906760<br>(-514.918838)	-514.9581310<br>(-514.970010)

Transition State, (Hartree)					
Entry	R	Total Electronic Energy	Sum of Electronic and Zero-Point Energies	Sum of Electronic and Thermal Enthalpies	Gibbs Free Energy
1	CH_3	-349.9164569<br>(-349.9308785)	-349.732847<br>(-349.747943)	-349.72329<br>(-349.738371)	-349.765725<br>(-349.780838)
2	NH_2	-365.9567591<br>(-365.9740143)	-365.78383<br>(-365.801723)	-365.774639<br>(-365.792477)	-365.816123<br>(-365.834046)
3	OH	-385.8194026<br>(-385.8360294)	-385.659771<br>(-385.676570)	-385.650615<br>(-385.667466)	-385.692136<br>(-385.708851)
4	F	-409.8387496<br>(-409.8500792)	-409.691518<br>(-409.703312)	-409.682762<br>(-409.694550)	-409.723667<br>(-409.735458)
5	CHO	-423.9121905<br>(-423.924619)	-423.746621<br>(-423.759552)	-423.736812<br>(-423.749740)	-423.780303<br>(-423.793228)
6	CN	-402.8405985<br>(-402.8518725)	-402.686347<br>(-402.698085)	-402.676612<br>(-402.688346)	-402.719892<br>(-402.731626)
7	NO_2	-515.0581848<br>(-515.0696141)	-514.899497<br>(-514.911363)	-514.889102<br>(-514.900967)	-514.934539<br>(-514.946396)

Cyclooctatriene [Product], (Hartree)					
Entry	R	Total Electronic Energy	Sum of Electronic and Zero-Point Energies	Sum of Electronic and Thermal Enthalpies	Gibbs Free Energy
1	CH_3	-349.9670239<br>(-349.9807713)	-349.780699<br>(-349.795164)	-349.77122<br>(-349.785672)	-349.8135200<br>(-349.827983)
2	NH_2	-366.0064205<br>(-366.0229569)	-365.830901<br>(-365.848077)	-365.821705<br>(-365.838836)	-365.8632930<br>(-365.880488)
3	OH	-385.8688178<br>(-385.8848906)	-385.70576<br>(-385.722254)	-385.696845<br>(-385.713317)	-385.7379890<br>(-385.754491)
4	F	-409.8857765<br>(-409.896365)	-409.735162<br>(-409.746283)	-409.726527<br>(-409.737632)	-409.7672960<br>(-409.778425)
5	CHO	-423.9592584<br>(-423.9709801)	-423.790822<br>(-423.803105)	-423.781053<br>(-423.793328)	-423.8246160<br>(-423.836892)
6	CN	-402.8881301<br>(-402.8987009)	-402.730788<br>(-402.741871)	-402.721162<br>(-402.732234)	-402.7643620<br>(-402.775448)
7	NO_2	-515.1063256<br>(-515.1170332)	-514.944418<br>(-514.955617)	-514.934177<br>(-514.945369)	-514.9794280<br>(-514.990624)

Note: Computed energies (Hartree) for 8π electrocyclic ring-closure of 1,3,5,7-octatetraene using M06-2X/6-31+G(d) and M06-2X/6-31+G(d,p) (in parentheses).

4. Energies for disubstituted system

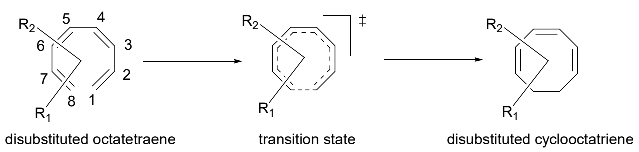

4.1 1,2-substituted

Octatetraene [Starting Material], (Hartree)

Entry	R_1	R_2	Total Electronic Energy	Sum of Electronic and Zero-Point Energies	Sum of Electronic and Thermal Enthalpies	Gibbs Free Energy
1	CH_3	CH_3	-389.2352107	-389.023883	-389.011059	-389.063954
2		NH_2	-405.2757525	-405.075535	-405.063859	-405.112318
3		OH	-425.1375282	-424.950011	-424.937677	-424.98829
4		F	-449.1558291	-448.980879	-448.968743	-449.019446
5		CHO	-463.2264627	-463.033926	-463.020802	-463.073345
6		CN	-442.1583449	-441.976463	-441.963409	-442.016395
7		NO_2	-554.3732404	-554.187551	-554.173764	-554.22913

Transition State, (Hartree)						
Entry	R_1	R_2	Total Electronic Energy	Sum of Electronic and Zero-Point Energies	Sum of Electronic and Thermal Enthalpies	Gibbs Free Energy
1	CH_3	CH_3	-389.2614012	-389.04573	-389.035187	-389.079734
2		NH_2	-405.2582544	-405.057117	-405.046252	-405.0915
3		OH	-425.1192573	-424.93086	-424.92017	-424.965172
4		F	-449.1363309	-448.960448	-448.950031	-448.994792
5		CHO	-463.2088771	-463.014875	-463.003494	-463.050426
6		CN	-442.1426925	-441.959947	-441.948606	-441.995561
7		NO_2	-554.3567336	-554.169253	-554.15733	-554.206482

Octatetraene [Starting Material], (Hartree)						
Entry	R_1	R_2	Total Electronic Energy	Sum of Electronic and Zero-Point Energies	Sum of Electronic and Thermal Enthalpies	Gibbs Free Energy
1	NH_2	CH_3	-405.2769095	-405.07623	-405.063891	-405.114034
2		NH_2	-421.3108688	-421.121482	-421.109284	-421.158817
3		OH	-441.1711367	-440.994792	-440.982525	-441.032473
4		F	-465.1907195	-465.026321	-465.014528	-465.063665
5		CHO	-479.2770895	-479.095166	-479.082265	-479.133946
6		CN	-458.2053699	-458.034434	-458.02166	-458.073252
7		NO_2	-570.4218756	-570.246575	-570.233234	-570.286335

Transition State, (Hartree)						
Entry	R_1	R_2	Total Electronic Energy	Sum of Electronic and Zero-Point Energies	Sum of Electronic and Thermal Enthalpies	Gibbs Free Energy
1	NH_2	CH_3	-405.259854	-405.058275	-405.047593	-405.09237
2		NH_2	-421.3000099	-421.10938	-421.098885	-421.143302
3		OH	-441.1590748	-440.981319	-440.97098	-441.015239
4		F	-465.1755584	-465.010413	-465.000406	-465.044053
5		CHO	-479.2490054	-479.065481	-479.054421	-479.10062
6		CN	-458.1860429	-458.013441	-458.00264	-458.048212
7		NO_2	-570.4044105	-570.227143	-570.215878	-570.262955

Octatetraene [Starting Material], (Hartree)						
Entry	R_1	R_2	Total Electronic Energy	Sum of Electronic and Zero-Point Energies	Sum of Electronic and Thermal Enthalpies	Gibbs Free Energy
1	OH	CH_3	-425.1367693	-424.948437	-424.936419	-424.98608
2		NH_2	-441.1711007	-440.994179	-440.982213	-441.031424
3		OH	-461.0324575	-460.868504	-460.85672	-460.905641
4		F	-485.0480902	-484.89631	-484.884816	-484.933392
5		CHO	-499.1317062	-498.96193	-498.949505	-499.000542
6		CN	-478.0608659	-477.902452	-477.890095	-477.941193
7		NO_2	-590.2826765	-590.119468	-590.106697	-590.158687

Transition State, (Hartree)						
Entry	R_1	R_2	Total Electronic Energy	Sum of Electronic and Zero-Point Energies	Sum of Electronic and Thermal Enthalpies	Gibbs Free Energy
1	OH	CH_3	-425.1177352	-424.928932	-424.91836	-424.963133
2		NH_2	-441.1594085	-440.981014	-440.970841	-441.014783
3		OH	-461.0199623	-460.854855	-460.844725	-460.888597
4		F	-485.0338622	-484.881105	-484.871363	-484.914643
5		CHO	-499.105773	-498.935177	-498.924282	-498.970299
6		CN	-478.0438354	-477.883874	-477.873282	-477.918616
7		NO_2	-590.2655725	-590.100835	-590.0899	-590.136425

Octatetraene [Starting Material], (Hartree)						
Entry	R_1	R_2	Total Electronic Energy	Sum of Electronic and Zero-Point Energies	Sum of Electronic and Thermal Enthalpies	Gibbs Free Energy
1	F	CH_3	-449.1519636	-448.975982	-448.964236	-449.013653
2		NH_2	-465.1867883	-465.022271	-465.010676	-465.059295
3		OH	-485.0466643	-484.895877	-484.884959	-484.93229
4		F	-509.0627785	-508.923995	-508.912661	-508.962023
5		CHO	-523.1394985	-522.982336	-522.970136	-523.020751
6		CN	-502.0704834	-501.924418	-501.912273	-501.963083
7		NO_2	-614.2808155	-614.130881	-614.118042	-614.170926

Transition State, (Hartree)						
Entry	R_1	R_2	Total Electronic Energy	Sum of Electronic and Zero-Point Energies	Sum of Electronic and Thermal Enthalpies	Gibbs Free Energy
1	F	CH_3	-449.1324456	-448.956478	-448.946014	-448.990951
2		NH_2	-465.169763	-465.004988	-464.994734	-465.039014
3		OH	-485.0257258	-484.873672	-484.863582	-484.907622
4		F	-509.0410342	-508.90119	-508.891539	-508.934893
5		CHO	-523.1207546	-522.962807	-522.952067	-522.997993
6		CN	-502.050223	-501.903338	-501.892785	-501.938299
7		NO_2	-614.2611345	-614.109966	-614.098723	-614.146329

Octatetraene [Starting Material], (Hartree)						
Entry	R_1	R_2	Total Electronic Energy	Sum of Electronic and Zero-Point Energies	Sum of Electronic and Thermal Enthalpies	Gibbs Free Energy
1	CHO	CH_3	-463.2248737	-463.031303	-463.018392	-463.070568
2		NH_2	-479.2736621	-479.091729	-479.078995	-479.130629
3		OH	-499.1332044	-498.96355	-498.951005	-499.002639
4		F	-523.1451448	-522.988484	-522.976089	-523.02776
5		CHO	-537.2091287	-537.034867	-537.021385	-537.075446
6		CN	-516.1405894	-515.977316	-515.963946	-516.018147
7		NO_2	-628.3534597	-628.186071	-628.17205	-628.227975

Transition State, (Hartree)						
Entry	R_1	R_2	Total Electronic Energy	Sum of Electronic and Zero-Point Energies	Sum of Electronic and Thermal Enthalpies	Gibbs Free Energy
1	CHO	CH_3	-463.2076301	-463.013834	-463.002326	-463.049744
2		NH_2	-479.2579163	-479.074812	-479.063718	-479.109928
3		OH	-499.1038918	-498.933658	-498.922616	-498.969095
4		F	-523.1190266	-522.96155	-522.950687	-522.997132
5		CHO	-537.1928556	-537.017402	-537.00544	-537.054392
6		CN	-516.1223184	-515.958228	-515.946394	-515.995075
7		NO_2	-628.3404034	-628.171921	-628.159374	-628.210168

Octatetraene [Starting Material], (Hartree)						
Entry	R_1	R_2	Total Electronic Energy	Sum of Electronic and Zero-Point Energies	Sum of Electronic and Thermal Enthalpies	Gibbs Free Energy
1	CN	CH_3	-442.1622408	-441.980124	-441.968255	-442.01789
2		NH_2	-458.2073091	-458.035858	-458.023489	-458.074114
3		OH	-478.0670359	-477.908606	-477.896231	-477.94795
4		F	-502.0793332	-501.933768	-501.922464	-501.971125
5		CHO	-516.1447388	-515.981633	-515.968411	-516.02187
6		CN	-495.0762673	-494.924169	-494.911899	-494.962823
7		NO_2	-607.2867167	-607.130465	-607.11665	-607.172095

Transition State, (Hartree)						
Entry	R_1	R_2	Total Electronic Energy	Sum of Electronic and Zero-Point Energies	Sum of Electronic and Thermal Enthalpies	Gibbs Free Energy
1	CN	CH_3	-442.1424612	-441.959883	-441.948547	-441.995585
2		NH_2	-458.1858403	-458.014308	-458.003193	-458.049533
3		OH	-478.0409541	-477.881926	-477.871083	-477.917074
4		F	-502.0546977	-501.908037	-501.897539	-501.943036
5		CHO	-516.1284533	-515.96403	-515.952395	-516.000506
6		CN	-495.0562032	-494.903015	-494.891472	-494.939442
7		NO_2	-607.2699248	-607.112348	-607.100157	-607.15008

Octatetraene [Starting Material], (Hartree)						
Entry	R_1	R_2	Total Electronic Energy	Sum of Electronic and Zero-Point Energies	Sum of Electronic and Thermal Enthalpies	Gibbs Free Energy
1	NO_2	CH_3	-554.3721443	-554.185132	-554.171812	-554.225624
2		NH_2	-570.4200721	-570.244561	-570.231396	-570.284493
3		OH	-590.276836	-590.113625	-590.100588	-590.154541
4		F	-614.2887373	-614.138381	-614.125551	-614.179357
5		CHO	-628.3550642	-628.186998	-628.173171	-628.22854
6		CN	-607.2843236	-607.127167	-607.113398	-607.169133
7		NO_2	-719.4954428	-719.334043	-719.31969	-719.376373

Transition State, (Hartree)						
Entry	R_1	R_2	Total Electronic Energy	Sum of Electronic and Zero-Point Energies	Sum of Electronic and Thermal Enthalpies	Gibbs Free Energy
1	NO_2	CH_3	-554.3571585	-554.16943	-554.157593	-554.206175
2		NH_2	-570.4048106	-570.228408	-570.2167	-570.264805
3		OH	-590.250765	-590.087288	-590.075699	-590.124017
4		F	-614.2640576	-614.112846	-614.10164	-614.149267
5		CHO	-628.3395793	-628.170566	-628.158127	-628.208801
6		CN	-607.2678682	-607.110194	-607.097904	-607.148141
7		NO_2	-719.4770227	-719.315076	-719.302019	-719.354545

4.2 1,3-substituted

Octatetraene [Starting Material], (Hartree)						
Entry	R_1	R_2	Total Electronic Energy	Sum of Electronic and Zero-Point Energies	Sum of Electronic and Thermal Enthalpies	Gibbs Free Energy
1	CH_3	CH_3	-389.2355486	-389.024493	-389.012507	-389.062437
2		NH_2	-405.2790524	-405.078721	-405.06636	-405.116509
3		OH	-425.1400999	-424.952352	-424.940023	-424.99069
4		F	-449.1560331	-448.981089	-448.968993	-449.019452

5		CHO	-463.2276081	-463.035013	-463.021918	-463.074502
6		CN	-442.1599451	-441.978123	-441.965176	-442.017368
7		NO_2	-554.3757609	-554.189737	-554.176273	-554.229946

Transition State, (Hartree)						
Entry	R_1	R_2	Total Electronic Energy	Sum of Electronic and Zero-Point Energies	Sum of Electronic and Thermal Enthalpies	Gibbs Free Energy
1	CH_3	CH_3	-389.2158996	-389.004122	-388.992925	-389.039068
2		NH_2	-405.2550247	-405.054306	-405.043405	-405.088623
3		OH	-425.1175397	-424.929533	-424.918831	-424.963777
4		F	-449.1363261	-448.960507	-448.950224	-448.994536
5		CHO	-463.2101452	-463.016495	-463.005018	-463.052192
6		CN	-442.1404375	-441.957577	-441.946327	-441.992962
7		NO_2	-554.3583927	-554.171078	-554.159121	-554.208477

Octatetraene [Starting Material], (Hartree)						
Entry	R_1	R_2	Total Electronic Energy	Sum of Electronic and Zero-Point Energies	Sum of Electronic and Thermal Enthalpies	Gibbs Free Energy
1	NH_2	CH_3	-405.2787739	-405.078013	-405.065649	-405.116063
2		NH_2	-421.3264768	-421.136023	-421.124203	-421.17267
3		OH	-441.1886666	-441.011969	-440.999932	-441.049262
4		F	-465.2031788	-465.039332	-465.027451	-465.076775
5		CHO	-479.2701386	-479.087975	-479.075165	-479.126647
6		CN	-458.2065576	-458.035703	-458.022925	-458.074634
7		NO_2	-570.4210912	-570.245342	-570.232205	-570.2851

Transition State, (Hartree)						
Entry	R_1	R_2	Total Electronic Energy	Sum of Electronic and Zero-Point Energies	Sum of Electronic and Thermal Enthalpies	Gibbs Free Energy
1	NH_2	CH_3	-405.256043	-405.054939	-405.044039	-405.089678
2		NH_2	-421.2951583	-421.104973	-421.094427	-421.138833
3		OH	-441.1581162	-440.980644	-440.970261	-441.014426
4		F	-465.1764042	-465.011124	-465.001179	-465.044653
5		CHO	-479.2510639	-479.067883	-479.0568	-479.102965
6		CN	-458.1811087	-458.009023	-457.998091	-458.043947
7		NO_2	-570.3992986	-570.222858	-570.211223	-570.25958

Octatetraene [Starting Material], (Hartree)						
Entry	R_1	R_2	Total Electronic Energy	Sum of Electronic and Zero-Point Energies	Sum of Electronic and Thermal Enthalpies	Gibbs Free Energy
1	OH	CH_3	-425.1388873	-424.950967	-424.938733	-424.988852
2		NH_2	-441.180773	-441.00398	-440.991847	-441.04137
3		OH	-461.0376772	-460.873484	-460.861659	-460.910788
4		F	-485.054105	-484.902646	-484.891046	-484.939874
5		CHO	-499.1277781	-498.958437	-498.945722	-498.997265
6		CN	-478.0587525	-477.900702	-477.88809	-477.93966
7		NO_2	-590.2746799	-590.112171	-590.09899	-590.151956

Transition State, (Hartree)						
Entry	R_1	R_2	Total Electronic Energy	Sum of Electronic and Zero-Point Energies	Sum of Electronic and Thermal Enthalpies	Gibbs Free Energy
1	OH	CH_3	-425.1148327	-424.926292	-424.915485	-424.962286
2		NH_2	-441.1531742	-440.975796	-440.965353	-441.009631
3		OH	-461.0152403	-460.850557	-460.840344	-460.8843

4		F	-485.0344943	-484.882131	-484.872346	-484.915683
5		CHO	-499.1091153	-498.938587	-498.927722	-498.973658
6		CN	-478.0389044	-477.879549	-477.868799	-477.914454
7		NO_2	-590.2572694	-590.093577	-590.082119	-590.130541

Octatetraene [Starting Material], (Hartree)						
Entry	R_1	R_2	Total Electronic Energy	Sum of Electronic and Zero-Point Energies	Sum of Electronic and Thermal Enthalpies	Gibbs Free Energy
1	F	CH_3	-449.1543572	-448.978896	-448.967041	-449.016729
2		NH_2	-465.1941644	-465.030295	-465.019313	-465.066467
3		OH	-485.0527382	-484.901068	-484.889585	-484.938421
4		F	-509.0682614	-508.929312	-508.918067	-508.966713
5		CHO	-523.1404059	-522.983922	-522.972204	-523.022239
6		CN	-502.0719394	-501.926506	-501.91504	-501.964223
7		NO_2	-614.2869525	-614.136754	-614.123975	-614.176516

Transition State, (Hartree)						
Entry	R_1	R_2	Total Electronic Energy	Sum of Electronic and Zero-Point Energies	Sum of Electronic and Thermal Enthalpies	Gibbs Free Energy
1	F	CH_3	-449.1295123	-448.953681	-448.943152	-448.988536
2		NH_2	-465.1681022	-465.003332	-464.993118	-465.03718
3		OH	-485.0297792	-484.877804	-484.867791	-484.911597
4		F	-509.0478773	-508.908218	-508.89865	-508.941754
5		CHO	-523.1224628	-522.964803	-522.954069	-522.999992
6		CN	-502.0513508	-501.904616	-501.894071	-501.93955
7		NO_2	-614.269202	-614.118209	-614.106942	-614.155222

Octatetraene [Starting Material], (Hartree)						
Entry	R_1	R_2	Total Electronic Energy	Sum of Electronic and Zero-Point Energies	Sum of Electronic and Thermal Enthalpies	Gibbs Free Energy
1	CHO	CH_3	-463.2244745	-463.030964	-463.018038	-463.070294
2		NH_2	-479.2664958	-479.084466	-479.071697	-479.123538
3		OH	-499.1282681	-498.959045	-498.946374	-498.997985
4		F	-523.1431046	-522.986612	-522.974208	-523.025512
5		CHO	-537.2140209	-537.039939	-537.026499	-537.080484
6		CN	-516.1437954	-515.980304	-515.966971	-516.020702
7		NO_2	-628.3585995	-628.190773	-628.176868	-628.232562

Transition State, (Hartree)						
Entry	R_1	R_2	Total Electronic Energy	Sum of Electronic and Zero-Point Energies	Sum of Electronic and Thermal Enthalpies	Gibbs Free Energy
1	CHO	CH_3	-463.205795	-463.012644	-463.000774	-463.050593
2		NH_2	-479.245771	-479.063474	-479.052079	-479.099121
3		OH	-499.1069606	-498.937238	-498.926123	-498.972711
4		F	-523.1243902	-522.967104	-522.956303	-523.002497
5		CHO	-537.1982113	-537.022923	-537.010932	-537.060024
6		CN	-516.1265664	-515.962466	-515.950694	-515.999216
7		NO_2	-628.3440133	-628.175335	-628.162843	-628.213868

Octatetraene [Starting Material], (Hartree)						
Entry	R_1	R_2	Total Electronic Energy	Sum of Electronic and Zero-Point Energies	Sum of Electronic and Thermal Enthalpies	Gibbs Free Energy
1	CN	CH_3	-442.162508	-441.980789	-441.967893	-442.0203
2		NH_2	-458.2014903	-458.030931	-458.018266	-458.069841
3		OH	-478.0611139	-477.903163	-477.890764	-477.941765

4		F	-502.0756087	-501.930371	-501.919017	-501.967565
5		CHO	-516.1476672	-515.984013	-515.970895	-516.023772
6		CN	-495.0762533	-494.923931	-494.910857	-494.964039
7		NO_2	-607.2900362	-607.133517	-607.119765	-607.174863

Transition State, (Hartree)						
Entry	R_1	R_2	Total Electronic Energy	Sum of Electronic and Zero-Point Energies	Sum of Electronic and Thermal Enthalpies	Gibbs Free Energy
1	CN	CH_3	-442.1383739	-441.95556	-441.944184	-441.991596
2		NH_2	-458.1771576	-458.005609	-457.994517	-458.040787
3		OH	-478.0385785	-477.879843	-477.868976	-477.914961
4		F	-502.0558802	-501.909223	-501.898783	-501.944069
5		CHO	-516.1302095	-515.965764	-515.954146	-516.002267
6		CN	-495.0581848	-494.90489	-494.893414	-494.941225
7		NO_2	-607.2756955	-607.117987	-607.105811	-607.156021

Octatetraene [Starting Material], (Hartree)						
Entry	R_1	R_2	Total Electronic Energy	Sum of Electronic and Zero-Point Energies	Sum of Electronic and Thermal Enthalpies	Gibbs Free Energy
1	NO_2	CH_3	-554.3707335	-554.183913	-554.170573	-554.224114
2		NH_2	-570.4116012	-570.235587	-570.22254	-570.275421
3		OH	-590.268686	-590.106584	-590.093451	-590.146764
4		F	-614.2824405	-614.132385	-614.119661	-614.17213
5		CHO	-628.3603082	-628.192529	-628.17869	-628.234104
6		CN	-607.2865439	-607.130016	-607.116242	-607.171648
7		NO_2	-719.5047206	-719.343354	-719.329082	-719.385753

Transition State, (Hartree)						
Entry	R_1	R_2	Total Electronic Energy	Sum of Electronic and Zero-Point Energies	Sum of Electronic and Thermal Enthalpies	Gibbs Free Energy
1	NO_2	CH_3	-554.3562864	-554.169233	-554.157127	-554.206547
2		NH_2	-570.3956408	-570.219691	-570.207888	-570.256325
3		OH	-590.2561716	-590.093133	-590.081451	-590.12984
4		F	-614.2727468	-614.122085	-614.110798	-614.15864
5		CHO	-628.3473774	-628.178852	-628.166385	-628.217093
6		CN	-607.274689	-607.117018	-607.104757	-607.154945
7		NO_2	-719.4921903	-719.330285	-719.317271	-719.370414

4.3 1,4-substituted

Octatetraene [Starting Material], (Hartree)						
Entry	R_1	R_2	Total Electronic Energy	Sum of Electronic and Zero-Point Energies	Sum of Electronic and Thermal Enthalpies	Gibbs Free Energy
1	CH_3	CH_3	-389.2358214	-389.024309	-389.011582	-389.063398
2		NH_2	-405.2781141	-405.077587	-405.065154	-405.115664
3		OH	-425.1400845	-424.952894	-424.94036	-424.992373
4		F	-449.1564948	-448.981942	-448.969743	-449.020638
5		CHO	-463.2273711	-463.034774	-463.021651	-463.074317
6		CN	-442.1592497	-441.977595	-441.964465	-442.017877
7		NO_2	-554.3737372	-554.188178	-554.174397	-554.229601

Transition State, (Hartree)						
Entry	R_1	R_2	Total Electronic Energy	Sum of Electronic and Zero-Point	Sum of Electronic and Thermal	Gibbs Free Energy

				Energies	Enthalpies	
1		CH_3	-389.2142031	-389.002266	-388.991113	-389.037255
2		NH_2	-405.2540856	-405.052958	-405.042183	-405.087229
3		OH	-425.1168909	-424.928696	-424.918055	-424.962945
4	CH_3	F	-449.136585	-448.961027	-448.950687	-448.995196
5		CHO	-463.209985	-463.016353	-463.004872	-463.05213
6		CN	-442.1394506	-441.956809	-441.945539	-441.992265
7		NO_2	-554.357098	-554.170035	-554.158106	-554.207016

Octatetraene [Starting Material], (Hartree)						
Entry	R_1	R_2	Total Electronic Energy	Sum of Electronic and Zero-Point Energies	Sum of Electronic and Thermal Enthalpies	Gibbs Free Energy
1		CH_3	-405.2775534	-405.076755	-405.064438	-405.114502
2		NH_2	-421.3180394	-421.128452	-421.116347	-421.165695
3		OH	-441.1802687	-441.003526	-440.991516	-441.040942
4	NH_2	F	-465.1975871	-465.03344	-465.021628	-465.070904
5		CHO	-479.2728253	-479.091057	-479.078151	-479.129812
6		CN	-458.2038911	-458.033062	-458.020206	-458.071943
7		NO_2	-570.4193326	-570.244769	-570.231151	-570.285242

Transition State, (Hartree)						
Entry	R_1	R_2	Total Electronic Energy	Sum of Electronic and Zero-Point Energies	Sum of Electronic and Thermal Enthalpies	Gibbs Free Energy
1		CH_3	-405.2539037	-405.052729	-405.041814	-405.087807
2		NH_2	-421.2923668	-421.101973	-421.091505	-421.135797
3		OH	-441.1554028	-440.977967	-440.967591	-441.011793
4	NH_2	F	-465.1761593	-465.011153	-465.001182	-465.044763
5		CHO	-479.253028	-479.069782	-479.058799	-479.104838
6		CN	-458.181817	-458.009821	-457.998913	-458.044776
7		NO_2	-570.4000214	-570.223589	-570.211994	-570.260404

Octatetraene [Starting Material], (Hartree)						
Entry	R_1	R_2	Total Electronic Energy	Sum of Electronic and Zero-Point Energies	Sum of Electronic and Thermal Enthalpies	Gibbs Free Energy
1		CH_3	-425.1373797	-424.94913	-424.937018	-424.987054
2		NH_2	-441.1785881	-441.001484	-440.989652	-441.038679
3		OH	-461.0400336	-460.875783	-460.864054	-460.913222
4	OH	F	-485.0569295	-484.905028	-484.893601	-484.942148
5		CHO	-499.1303493	-498.96061	-498.948198	-498.999157
6		CN	-478.0608931	-477.902108	-477.88976	-477.940696
7		NO_2	-590.2758552	-590.113331	-590.100258	-590.153604

Transition State, (Hartree)						
Entry	R_1	R_2	Total Electronic Energy	Sum of Electronic and Zero-Point Energies	Sum of Electronic and Thermal Enthalpies	Gibbs Free Energy
1		CH_3	-425.1130325	-424.924645	-424.913956	-424.959267
2		NH_2	-441.1516475	-440.974127	-440.963815	-441.007917
3		OH	-461.0143369	-460.849708	-460.839519	-460.883495
4	OH	F	-485.034775	-484.88246	-484.872706	-484.916008
5		CHO	-499.1108153	-498.940205	-498.929414	-498.975217
6		CN	-478.0391745	-477.879947	-477.869196	-477.91494
7		NO_2	-590.2572514	-590.093345	-590.081938	-590.130427

Octatetraene [Starting Material], (Hartree)						
Entry	R_1	R_2	Total Electronic	Sum of Electronic	Sum of Electronic	Gibbs Free Energy

			Energy	and Zero-Point Energies	and Thermal Enthalpies	
1	F	CH_3	-449.1518447	-448.976128	-448.976128	-449.014941
2		NH_2	-465.1944834	-465.030009	-465.018429	-465.067215
3		OH	-485.0562157	-484.904965	-484.894273	-484.940637
4		F	-509.0714042	-508.932724	-508.922314	-508.968318
5		CHO	-523.1413152	-522.984371	-522.972167	-523.022867
6		CN	-502.0732067	-501.9273	-501.916044	-501.963913
7		NO_2	-614.2870747	-614.137541	-614.124609	-614.17804

Transition State, (Hartree)						
Entry	R_1	R_2	Total Electronic Energy	Sum of Electronic and Zero-Point Energies	Sum of Electronic and Thermal Enthalpies	Gibbs Free Energy
1	F	CH_3	-449.1278747	-448.952094	-448.941664	-448.98657
2		NH_2	-465.1674715	-465.002525	-464.992465	-465.036304
3		OH	-485.0296745	-484.87763	-484.867709	-484.911401
4		F	-509.048994	-508.909478	-508.899892	-508.943115
5		CHO	-523.1235887	-522.965765	-522.955146	-523.000821
6		CN	-502.0513709	-501.904731	-501.894177	-501.939757
7		NO_2	-614.2690172	-614.117897	-614.106673	-614.154511

Octatetraene [Starting Material], (Hartree)						
Entry	R_1	R_2	Total Electronic Energy	Sum of Electronic and Zero-Point Energies	Sum of Electronic and Thermal Enthalpies	Gibbs Free Energy
1	CHO	CH_3	-463.2246636	-463.031277	-463.018322	-463.070794
2		NH_2	-479.2707853	-479.088808	-479.07611	-479.127587
3		OH	-499.13073	-498.961406	-498.948808	-499.000225
4		F	-523.1445929	-522.988012	-522.975637	-523.026876
5		CHO	-537.21206	-537.037731	-537.0243	-537.078065
6		CN	-516.1432076	-515.979847	-515.966488	-516.020371
7		NO_2	-628.356768	-628.189335	-628.175327	-628.231191

Transition State, (Hartree)						
Entry	R_1	R_2	Total Electronic Energy	Sum of Electronic and Zero-Point Energies	Sum of Electronic and Thermal Enthalpies	Gibbs Free Energy
1	CHO	CH_3	-463.2044547	-463.011106	-462.999431	-463.047599
2		NH_2	-479.2472573	-479.064544	-479.053324	-479.100005
3		OH	-499.1088224	-498.938977	-498.92795	-498.974384
4		F	-523.1262745	-522.96898	-522.958239	-523.004315
5		CHO	-537.1975249	-537.02233	-537.010459	-537.059206
6		CN	-516.1254283	-515.961216	-515.949425	-515.998017
7		NO_2	-628.3420885	-628.173522	-628.161025	-628.211991

Octatetraene [Starting Material], (Hartree)						
Entry	R_1	R_2	Total Electronic Energy	Sum of Electronic and Zero-Point Energies	Sum of Electronic and Thermal Enthalpies	Gibbs Free Energy
1	CN	CH_3	-442.1613406	-441.978564	-441.965968	-442.017454
2		NH_2	-458.2078256	-458.036454	-458.024092	-458.074681
3		OH	-478.0680834	-477.909886	-477.898424	-477.946833
4		F	-502.0804498	-501.935096	-501.923792	-501.972326
5		CHO	-516.1466017	-515.983533	-515.970348	-516.023623
6		CN	-495.0785229	-494.925953	-494.912911	-494.966225
7		NO_2	-607.2912637	-607.134687	-607.120983	-607.176282

Transition State, (Hartree)						
Entry	R_1	R_2	Total Electronic	Sum of Electronic	Sum of Electronic	Gibbs Free Energy

			Energy	and Zero-Point Energies	and Thermal Enthalpies	
1	CN	CH_3	-442.1372253	-441.954277	-441.942969	-441.990128
2		NH_2	-458.1795672	-458.007615	-457.996671	-458.04273
3		OH	-478.0408065	-477.881742	-477.870979	-477.916827
4		F	-502.0582486	-501.911874	-501.901405	-501.946853
5		CHO	-516.1296596	-515.96505	-515.953497	-516.001519
6		CN	-495.0571945	-494.903819	-494.892384	-494.940083
7		NO_2	-607.2738581	-607.11627	-607.104124	-607.154241

Octatetraene [Starting Material], (Hartree)						
Entry	R_1	R_2	Total Electronic Energy	Sum of Electronic and Zero-Point Energies	Sum of Electronic and Thermal Enthalpies	Gibbs Free Energy
1	NO_2	CH_3	-554.3734839	-554.186293	-554.172948	-554.227518
2		NH_2	-570.4230236	-570.247055	-570.234052	-570.286675
3		OH	-590.28227	-590.118962	-590.106099	-590.15871
4		F	-614.2931256	-614.142622	-614.129947	-614.182476
5		CHO	-628.3574394	-628.189397	-628.175554	-628.231268
6		CN	-607.2895669	-607.132425	-607.118725	-607.174221
7		NO_2	-719.5074434	-719.346034	-719.331762	-719.389296

Transition State, (Hartree)						
Entry	R_1	R_2	Total Electronic Energy	Sum of Electronic and Zero-Point Energies	Sum of Electronic and Thermal Enthalpies	Gibbs Free Energy
1	NO_2	CH_3	-554.3552926	-554.168176	-554.156048	-554.156048
2		NH_2	-570.3986914	-570.222416	-570.210687	-570.259155
3		OH	-590.2592615	-590.09577	-590.084259	-590.132524
4		F	-614.275832	-614.125041	-614.113806	-614.16167
5		CHO	-628.3463948	-628.177408	-628.165044	-628.215573
6		CN	-607.2737053	-607.115995	-607.103766	-607.153896
7		NO_2	-719.4905777	-719.328561	-719.31565	-719.367836

4.4 1,5-substituted

Octatetraene [Starting Material], (Hartree)						
Entry	R_1	R_2	Total Electronic Energy	Sum of Electronic and Zero-Point Energies	Sum of Electronic and Thermal Enthalpies	Gibbs Free Energy
1	CH_3	CH_3	-389.2356769	-389.023791	-389.011223	-389.062188
2		NH_2	-405.2783685	-405.07814	-405.065633	-405.116511
3		OH	-425.1399517	-424.952615	-424.94107	-424.989334
4		F	-449.1560013	-448.981472	-448.970107	-449.018641
5		CHO	-463.2266073	-463.033952	-463.02076	-463.073857
6		CN	-442.1582717	-441.976721	-441.963644	-442.016909
7		NO_2	-554.3726161	-554.186983	-554.173306	-554.228005

Transition State, (Hartree)						
Entry	R_1	R_2	Total Electronic Energy	Sum of Electronic and Zero-Point Energies	Sum of Electronic and Thermal Enthalpies	Gibbs Free Energy
1	CH_3	CH_3	-389.2144435	-389.002411	-388.991309	-389.037243
2		NH_2	-405.2540154	-405.053208	-405.042344	-405.087531
3		OH	-425.1175154	-424.929328	-424.918718	-424.963504
4		F	-449.137043	-448.961354	-448.951079	-448.995408
5		CHO	-463.2094906	-463.01592	-463.004469	-463.05168
6		CN	-442.1392344	-441.95644	-441.945228	-441.991809
7		NO_2	-554.3569165	-554.169787	-554.157789	-554.206998

Octatetraene [Starting Material], (Hartree)						
Entry	R_1	R_2	Total Electronic Energy	Sum of Electronic and Zero-Point Energies	Sum of Electronic and Thermal Enthalpies	Gibbs Free Energy
1	NH_2	CH_3	-405.2774865	-405.076686	-405.065181	-405.112915
2		NH_2	-421.3203465	-421.13094	-421.118794	-421.168314
3		OH	-441.1819769	-441.00541	-440.993277	-441.043318
4		F	-465.1984053	-465.034388	-465.022534	-465.07208
5		CHO	-479.2686642	-479.0873	-479.074337	-479.126412
6		CN	-458.2013161	-458.030526	-458.017715	-458.069667
7		NO_2	-570.4148085	-570.240377	-570.226764	-570.281119

Transition State, (Hartree)						
Entry	R_1	R_2	Total Electronic Energy	Sum of Electronic and Zero-Point Energies	Sum of Electronic and Thermal Enthalpies	Gibbs Free Energy
1	NH_2	CH_3	-405.2549104	-405.053454	-405.042682	-405.087867
2		NH_2	-421.2956576	-421.105414	-421.094921	-421.139226
3		OH	-441.159	-440.98143	-440.971176	-441.015108
4		F	-465.1782394	-465.01328	-465.003345	-465.046845
5		CHO	-479.2508929	-479.067889	-479.056805	-479.103133
6		CN	-458.1807117	-458.008677	-457.99776	-458.043632
7		NO_2	-570.3982473	-570.221804	-570.210218	-570.258374

Octatetraene [Starting Material], (Hartree)						
Entry	R_1	R_2	Total Electronic Energy	Sum of Electronic and Zero-Point Energies	Sum of Electronic and Thermal Enthalpies	Gibbs Free Energy
1	OH	CH_3	-425.1374746	-424.949188	-424.937136	-424.987002
2		NH_2	-441.1800921	-441.003167	-440.991296	-441.040559
3		OH	-461.0416604	-460.877501	-460.865705	-460.915073
4		F	-485.0574634	-484.905909	-484.894383	-484.943449
5		CHO	-499.1277939	-498.958546	-498.945982	-498.997395
6		CN	-478.0598382	-477.901244	-477.888822	-477.939921
7		NO_2	-590.2737133	-590.111232	-590.09809	-590.1515

Transition State, (Hartree)						
Entry	R_1	R_2	Total Electronic Energy	Sum of Electronic and Zero-Point Energies	Sum of Electronic and Thermal Enthalpies	Gibbs Free Energy
1	OH	CH_3	-425.1139619	-424.92528	-424.914676	-424.959661
2		NH_2	-441.1544211	-440.976953	-440.966628	-441.010731
3		OH	-461.0174732	-460.852609	-460.84254	-460.886242
4		F	-485.0365214	-484.884196	-484.874452	-484.917718
5		CHO	-499.1087301	-498.93847	-498.927554	-498.973681
6		CN	-478.0384432	-477.878944	-477.868267	-477.913801
7		NO_2	-590.2561217	-590.092193	-590.080807	-590.128803

Octatetraene [Starting Material], (Hartree)						
Entry	R_1	R_2	Total Electronic Energy	Sum of Electronic and Zero-Point Energies	Sum of Electronic and Thermal Enthalpies	Gibbs Free Energy
1	F	CH_3	-449.1517985	-448.97575	-448.964024	-449.013392
2		NH_2	-465.1946367	-465.030137	-465.018575	-465.067437
3		OH	-485.0566978	-484.905239	-484.894585	-484.940854
4		F	-509.0722825	-508.9336	-508.923146	-508.969464
5		CHO	-523.1418472	-522.98483	-522.972634	-523.023292
6		CN	-502.0744813	-501.928273	-501.916132	-501.966999

7		NO_2	-614.2877947	-614.138001	-614.125136	-614.178558

Transition State, (Hartree)						
Entry	R_1	R_2	Total Electronic Energy	Sum of Electronic and Zero-Point Energies	Sum of Electronic and Thermal Enthalpies	Gibbs Free Energy
1	F	CH_3	-449.1283524	-448.952243	-448.941889	-448.986573
2		NH_2	-465.16842	-465.003632	-464.993493	-465.037469
3		OH	-485.0312061	-484.878997	-484.869134	-484.912674
4		F	-509.0499655	-508.910282	-508.90075	-508.94384
5		CHO	-523.1219666	-522.964222	-522.95356	-522.999359
6		CN	-502.0512304	-501.904477	-501.893966	-501.93943
7		NO_2	-614.2687373	-614.117522	-614.106297	-614.15416

Octatetraene [Starting Material], (Hartree)						
Entry	R_1	R_2	Total Electronic Energy	Sum of Electronic and Zero-Point Energies	Sum of Electronic and Thermal Enthalpies	Gibbs Free Energy
1	CHO	CH_3	-463.2239354	-463.03022	-463.017343	-463.069376
2		NH_2	-479.2662908	-479.084189	-479.071454	-479.123229
3		OH	-499.128142	-498.958841	-498.946178	-498.997942
4		F	-523.1430766	-522.986681	-522.974156	-523.026503
5		CHO	-537.2132898	-537.038595	-537.02526	-537.078659
6		CN	-516.1444416	-515.980736	-515.967449	-516.020948
7		NO_2	-628.3582667	-628.190612	-628.176644	-628.232402

Transition State, (Hartree)						
Entry	R_1	R_2	Total Electronic Energy	Sum of Electronic and Zero-Point Energies	Sum of Electronic and Thermal Enthalpies	Gibbs Free Energy
1	CHO	CH_3	-463.2037487	-463.009988	-462.998384	-463.046205
2		NH_2	-479.2433002	-479.060963	-479.049578	-479.096661
3		OH	-499.1061027	-498.936373	-498.925256	-498.971917
4		F	-523.1251243	-522.967925	-522.957143	-523.003344
5		CHO	-537.1972652	-537.022142	-537.010179	-537.059258
6		CN	-516.1257407	-515.96157	-515.949802	-515.998361
7		NO_2	-628.343177	-628.174544	-628.162095	-628.21288

Octatetraene [Starting Material], (Hartree)						
Entry	R_1	R_2	Total Electronic Energy	Sum of Electronic and Zero-Point Energies	Sum of Electronic and Thermal Enthalpies	Gibbs Free Energy
1	CN	CH_3	-442.1606815	-441.97815	-441.965529	-442.017273
2		NH_2	-458.203054	-458.031649	-458.01926	-458.069997
3		OH	-478.065344	-477.907033	-477.89557	-477.943774
4		F	-502.0802008	-501.934567	-501.9224	-501.974825
5		CHO	-516.1494667	-515.985951	-515.972833	-516.025787
6		CN	-495.0817051	-494.928824	-494.915866	-494.968858
7		NO_2	-607.2945602	-607.137812	-607.124163	-607.178928

Transition State, (Hartree)						
Entry	R_1	R_2	Total Electronic Energy	Sum of Electronic and Zero-Point Energies	Sum of Electronic and Thermal Enthalpies	Gibbs Free Energy
1	CN	CH_3	-442.1367005	-441.95417	-441.942821	-441.990109
2		NH_2	-458.1773945	-458.005786	-457.994794	-458.040988
3		OH	-478.0388896	-477.880266	-477.8694	-477.915451
4		F	-502.0574413	-501.911192	-501.900713	-501.946177
5		CHO	-516.1293461	-515.96501	-515.953383	-516.001631
6		CN	-495.0575551	-494.90431	-494.89286	-494.940648

7		NO_2	-607.2748942	-607.117179	-607.105037	-607.155143

Octatetraene [Starting Material], (Hartree)						
Entry	R_1	R_2	Total Electronic Energy	Sum of Electronic and Zero-Point Energies	Sum of Electronic and Thermal Enthalpies	Gibbs Free Energy
1	NO_2	CH_3	-554.3722094	-554.184664	-554.17141	-554.22526
2		NH_2	-570.4150755	-570.239125	-570.22603	-570.279323
3		OH	-590.2769897	-590.113912	-590.10088	-590.154482
4		F	-614.2915698	-614.14141	-614.128538	-614.18248
5		CHO	-628.3613438	-628.193162	-628.179376	-628.234678
6		CN	-607.2928242	-607.13525	-607.121618	-607.176665
7		NO_2	-719.5059662	-719.344309	-719.32999	-719.387418

Transition State, (Hartree)						
Entry	R_1	R_2	Total Electronic Energy	Sum of Electronic and Zero-Point Energies	Sum of Electronic and Thermal Enthalpies	Gibbs Free Energy
1	NO_2	CH_3	-554.3544424	-554.167477	-554.155381	-554.204891
2		NH_2	-570.3951566	-570.219031	-570.20729	-570.255786
3		OH	-590.2561161	-590.09286	-590.081293	-590.12956
4		F	-614.2744839	-614.12379	-614.112539	-614.16035
5		CHO	-628.3462137	-628.177537	-628.165109	-628.215853
6		CN	-607.2742437	-607.11646	-607.104234	-607.154427
7		NO_2	-719.4914979	-719.329248	-719.316372	-719.368647

4.5 1,6-substituted

Octatetraene [Starting Material], (Hartree)						
Entry	R_1	R_2	Total Electronic Energy	Sum of Electronic and Zero-Point Energies	Sum of Electronic and Thermal Enthalpies	Gibbs Free Energy
1	CH_3	CH_3	-389.2408404	-389.02963	-389.016851	-389.068515
2		NH_2	-405.2803586	-405.080223	-405.067641	-405.11887
3		OH	-425.1423727	-424.95509	-424.942661	-424.993623
4		F	-449.1580061	-448.983303	-448.971118	-449.0221
5		CHO	-463.227414	-463.03467	-463.021396	-463.074853
6		CN	-442.1625183	-441.980886	-441.968631	-442.0193
7		NO_2	-554.3743169	-554.188071	-554.17423	-554.230667

Transition State, (Hartree)						
Entry	R_1	R_2	Total Electronic Energy	Sum of Electronic and Zero-Point Energies	Sum of Electronic and Thermal Enthalpies	Gibbs Free Energy
1	CH_3	CH_3	-389.2160227	-389.003965	-388.992799	-389.039086
2		NH_2	-405.2548367	-405.053925	-405.043049	-405.088301
3		OH	-425.1181186	-424.929823	-424.919233	-424.963974
4		F	-449.1362236	-448.960272	-448.950047	-448.994249
5		CHO	-463.2105168	-463.016898	-463.005445	-463.05261
6		CN	-442.1407762	-441.958077	-441.946826	-441.993536
7		NO_2	-554.3590175	-554.171888	-554.15991	-554.209404

Octatetraene [Starting Material], (Hartree)						
Entry	R_1	R_2	Total Electronic Energy	Sum of Electronic and Zero-Point Energies	Sum of Electronic and Thermal Enthalpies	Gibbs Free Energy
1	NH_2	CH_3	-405.2823173	-405.081937	-405.069453	-405.120101
2		NH_2	-421.3212962	-421.131703	-421.119527	-421.169257

3		OH	-441.1835075	-441.006786	-440.994764	-441.044319
4		F	-465.1996087	-465.035375	-465.023618	-465.072911
5		CHO	-479.2705002	-479.088709	-479.075667	-479.128406
6		CN	-458.2058274	-458.03472	-458.021983	-458.073471
7		NO_2	-570.4183945	-570.243251	-570.22954	-570.285265

Transition State, (Hartree)						
Entry	R_1	R_2	Total Electronic Energy	Sum of Electronic and Zero-Point Energies	Sum of Electronic and Thermal Enthalpies	Gibbs Free Energy
1	NH_2	CH_3	-405.255848	-405.054744	-405.043769	-405.089994
2		NH_2	-421.2934879	-421.10348	-421.092856	-421.137534
3		OH	-441.1569906	-440.979714	-440.969364	-441.013557
4		F	-465.1759424	-465.011096	-465.001094	-465.044771
5		CHO	-479.2534883	-479.070324	-479.059263	-479.105473
6		CN	-458.183413	-458.011324	-458.000423	-458.04629
7		NO_2	-570.4024349	-570.22591	-570.214325	-570.262558

Octatetraene [Starting Material], (Hartree)						
Entry	R_1	R_2	Total Electronic Energy	Sum of Electronic and Zero-Point Energies	Sum of Electronic and Thermal Enthalpies	Gibbs Free Energy
1	OH	CH_3	-425.1421367	-424.954232	-424.942035	-424.992325
2		NH_2	-441.1817224	-441.004789	-440.9928	-441.042684
3		OH	-461.0435969	-460.879433	-460.867651	-460.917061
4		F	-485.0590662	-484.907343	-484.895861	-484.944795
5		CHO	-499.1280236	-498.958914	-498.946846	-498.997708
6		CN	-478.0636477	-477.905039	-477.892606	-477.944029
7		NO_2	-590.2755895	-590.112459	-590.099246	-590.153915

Transition State, (Hartree)						
Entry	R_1	R_2	Total Electronic Energy	Sum of Electronic and Zero-Point Energies	Sum of Electronic and Thermal Enthalpies	Gibbs Free Energy
1	OH	CH_3	-425.1150052	-424.926653	-424.915854	-424.961957
2		NH_2	-441.1533913	-440.975893	-440.965515	-441.009816
3		OH	-461.0162939	-460.85154	-460.841419	-460.88527
4		F	-485.0347254	-484.882431	-484.872666	-484.916005
5		CHO	-499.1106195	-498.940124	-498.929277	-498.975186
6		CN	-478.0403328	-477.880873	-477.870179	-477.915754
7		NO_2	-590.2587434	-590.094933	-590.083527	-590.131687

Octatetraene [Starting Material], (Hartree)						
Entry	R_1	R_2	Total Electronic Energy	Sum of Electronic and Zero-Point Energies	Sum of Electronic and Thermal Enthalpies	Gibbs Free Energy
1	F	CH_3	-449.1566224	-448.981351	-448.969393	-449.020208
2		NH_2	-465.1964431	-465.031998	-465.020359	-465.069458
3		OH	-485.0580917	-484.906961	-484.896156	-484.94309
4		F	-509.0735884	-508.934751	-508.924312	-508.970788
5		CHO	-523.1419879	-522.985164	-522.97348	-523.023312
6		CN	-502.0774502	-501.931232	-501.919143	-501.969775
7		NO_2	-614.2890642	-614.138791	-614.12755	-614.17694

Transition State, (Hartree)						
Entry	R_1	R_2	Total Electronic Energy	Sum of Electronic and Zero-Point Energies	Sum of Electronic and Thermal Enthalpies	Gibbs Free Energy
1	F	CH_3	-449.129715	-448.953991	-448.943441	-448.989146
2		NH_2	-465.1687823	-465.003989	-464.993804	-465.03793

3		OH	-485.0315055	-484.879217	-484.869355	-484.912909
4		F	-509.0490981	-508.909415	-508.899861	-508.943001
5		CHO	-523.1229935	-522.965417	-522.954698	-523.00061
6		CN	-502.0526748	-501.905957	-501.89543	-501.940924
7		NO_2	-614.2706447	-614.119512	-614.108285	-614.156326

Octatetraene [Starting Material], (Hartree)						
Entry	R_1	R_2	Total Electronic Energy	Sum of Electronic and Zero-Point Energies	Sum of Electronic and Thermal Enthalpies	Gibbs Free Energy
1	CHO	CH_3	-463.2294132	-463.036299	-463.023297	-463.075587
2		NH_2	-479.2709089	-479.088902	-479.076118	-479.127842
3		OH	-499.1318061	-498.962604	-498.949973	-499.001521
4		F	-523.1459447	-522.98929	-522.976918	-523.028265
5		CHO	-537.2132524	-537.038768	-537.025201	-537.079601
6		CN	-516.1476468	-515.984145	-515.970776	-516.024896
7		NO_2	-628.3583456	-628.190621	-628.178161	-628.230509

Transition State, (Hartree)						
Entry	R_1	R_2	Total Electronic Energy	Sum of Electronic and Zero-Point Energies	Sum of Electronic and Thermal Enthalpies	Gibbs Free Energy
1	CHO	CH_3	-463.2060703	-463.012451	-463.000805	-463.048802
2		NH_2	-479.2480144	-479.065404	-479.054095	-479.101031
3		OH	-499.1091205	-498.93897	-498.927983	-498.974387
4		F	-523.1256347	-522.968358	-522.957557	-523.003832
5		CHO	-537.1975084	-537.02226	-537.010333	-537.059229
6		CN	-516.1269176	-515.96291	-515.951121	-515.999743
7		NO_2	-628.3443632	-628.17593	-628.163415	-628.214723

Octatetraene [Starting Material], (Hartree)						
Entry	R_1	R_2	Total Electronic Energy	Sum of Electronic and Zero-Point Energies	Sum of Electronic and Thermal Enthalpies	Gibbs Free Energy
1	CN	CH_3	-442.1657477	-441.983582	-441.970817	-442.023336
2		NH_2	-458.2072877	-458.036192	-458.023734	-458.074628
3		OH	-478.0681165	-477.909903	-477.898377	-477.94689
4		F	-502.0823592	-501.936558	-501.925348	-501.973411
5		CHO	-516.1496482	-515.986233	-515.972965	-516.026707
6		CN	-495.0839072	-494.93177	-494.918575	-494.972864
7		NO_2	-607.2950574	-607.138197	-607.124377	-607.1805

Transition State, (Hartree)						
Entry	R_1	R_2	Total Electronic Energy	Sum of Electronic and Zero-Point Energies	Sum of Electronic and Thermal Enthalpies	Gibbs Free Energy
1	CN	CH_3	-442.1390825	-441.956179	-441.944843	-441.99222
2		NH_2	-458.1806619	-458.008877	-457.997861	-458.044055
3		OH	-478.0416668	-477.882523	-477.871751	-477.917621
4		F	-502.0578569	-501.911205	-501.900767	-501.946144
5		CHO	-516.1296643	-515.965252	-515.953621	-516.001872
6		CN	-495.0585496	-494.905249	-494.893789	-494.941619
7		NO_2	-607.2757291	-607.117974	-607.105825	-607.156256

Octatetraene [Starting Material], (Hartree)						
Entry	R_1	R_2	Total Electronic Energy	Sum of Electronic and Zero-Point Energies	Sum of Electronic and Thermal Enthalpies	Gibbs Free Energy
1	NO_2	CH_3	-554.3778669	-554.191221	-554.178646	-554.230293

2		NH_2	-570.4204856	-570.244919	-570.231722	-570.285208
3		OH	-590.2806856	-590.118093	-590.105828	-590.156897
4		F	-614.294356	-614.144008	-614.131211	-614.184687
5		CHO	-628.3609808	-628.192613	-628.178657	-628.234946
6		CN	-607.2950379	-607.137712	-607.123942	-607.180136
7		NO_2	-719.5059086	-719.344186	-719.329647	-719.388551

Transition State, (Hartree)						
Entry	R_1	R_2	Total Electronic Energy	Sum of Electronic and Zero-Point Energies	Sum of Electronic and Thermal Enthalpies	Gibbs Free Energy
1	NO_2	CH_3	-554.3570465	-554.170092	-554.157982	-554.20757
2		NH_2	-570.4000183	-570.224054	-570.212255	-570.260806
3		OH	-590.2599159	-590.096396	-590.084927	-590.13297
4		F	-614.2753918	-614.124629	-614.113391	-614.161206
5		CHO	-628.3464345	-628.177761	-628.165343	-628.215924
6		CN	-607.2749957	-607.117475	-607.105205	-607.155534
7		NO_2	-719.4919699	-719.329977	-719.317008	-719.369831

4.6 1,7-substituted

Octatetraene [Starting Material], (Hartree)						
Entry	R_1	R_2	Total Electronic Energy	Sum of Electronic and Zero-Point Energies	Sum of Electronic and Thermal Enthalpies	Gibbs Free Energy
1	CH_3	CH_3	-389.2368689	-389.025408	-389.013626	-389.062927
2		NH_2	-405.2782016	-405.07759	-405.065282	-405.11573
3		OH	-425.1411699	-424.953964	-424.942435	-424.991145
4		F	-449.1566851	-448.981835	-448.969753	-449.020831
5		CHO	-463.2230715	-463.03044	-463.017382	-463.070264
6		CN	-442.1570332	-441.975081	-441.962992	-442.013436
7		NO_2	-554.3695142	-554.183684	-554.170033	-554.225376

Transition State, (Hartree)						
Entry	R_1	R_2	Total Electronic Energy	Sum of Electronic and Zero-Point Energies	Sum of Electronic and Thermal Enthalpies	Gibbs Free Energy
1	CH_3	CH_3	-389.2188947	-389.007127	-388.995985	-389.042157
2		NH_2	-405.258788	-405.057722	-405.046846	-405.092212
3		OH	-425.1200782	-424.931893	-424.921319	-424.96613
4		F	-449.13677	-448.960799	-448.950508	-448.994995
5		CHO	-463.2119075	-463.018144	-463.006677	-463.053945
6		CN	-442.1420331	-441.958924	-441.947698	-441.994462
7		NO_2	-554.3615735	-554.174374	-554.162453	-554.211593

Octatetraene [Starting Material], (Hartree)						
Entry	R_1	R_2	Total Electronic Energy	Sum of Electronic and Zero-Point Energies	Sum of Electronic and Thermal Enthalpies	Gibbs Free Energy
1	NH_2	CH_3	-405.2785313	-405.077677	-405.065413	-405.115859
2		NH_2	-421.3197657	-421.129962	-421.117881	-421.167738
3		OH	-441.1827687	-441.00601	-440.993976	-441.044066
4		F	-465.1987287	-465.034609	-465.022844	-465.072588
5		CHO	-479.2652593	-479.083617	-479.070758	-479.123091
6		CN	-458.1995052	-458.028446	-458.015737	-458.068173
7		NO_2	-570.4121426	-570.237141	-570.223725	-570.278164

Transition State, (Hartree)

Entry	R_1	R_2	Total Electronic Energy	Sum of Electronic and Zero-Point Energies	Sum of Electronic and Thermal Enthalpies	Gibbs Free Energy
1	NH_2	CH_3	-405.2591117	-405.058082	-405.047237	-405.092688
2		NH_2	-421.3015489	-421.111249	-421.100756	-421.145233
3		OH	-441.1621748	-440.984651	-440.974354	-441.018536
4		F	-465.1787369	-465.013566	-465.003592	-465.047284
5		CHO	-479.2521579	-479.068937	-479.05789	-479.104172
6		CN	-458.1846342	-458.0123	-458.001437	-458.047327
7		NO_2	-570.4059441	-570.229212	-570.217743	-570.265408

Octatetraene [Starting Material], (Hartree)						
Entry	R_1	R_2	Total Electronic Energy	Sum of Electronic and Zero-Point Energies	Sum of Electronic and Thermal Enthalpies	Gibbs Free Energy
1	OH	CH_3	-425.1382624	-424.949565	-424.937667	-424.987429
2		NH_2	-441.1797336	-441.002188	-440.990478	-441.039585
3		OH	-461.042696	-460.878246	-460.866592	-460.915825
4		F	-485.0581411	-484.906245	-484.894894	-484.943676
5		CHO	-499.1243808	-498.954924	-498.942464	-498.993993
6		CN	-478.0585291	-477.89976	-477.887438	-477.938843
7		NO_2	-590.2709406	-590.108155	-590.09514	-590.148737

Transition State, (Hartree)						
Entry	R_1	R_2	Total Electronic Energy	Sum of Electronic and Zero-Point Energies	Sum of Electronic and Thermal Enthalpies	Gibbs Free Energy
1	OH	CH_3	-425.1183636	-424.929594	-424.918995	-424.964019
2		NH_2	-441.1587068	-440.981348	-440.97094	-441.015431
3		OH	-461.0193485	-460.854316	-460.844214	-460.888139
4		F	-485.035996	-484.883582	-484.873783	-484.917343
5		CHO	-499.1100895	-498.939683	-498.928805	-498.974876
6		CN	-478.0400999	-477.880896	-477.870132	-477.915996
7		NO_2	-590.2586601	-590.09498	-590.083572	-590.131648

Octatetraene [Starting Material], (Hartree)						
Entry	R_1	R_2	Total Electronic Energy	Sum of Electronic and Zero-Point Energies	Sum of Electronic and Thermal Enthalpies	Gibbs Free Energy
1	F	CH_3	-449.1527261	-448.9768	-448.965086	-449.015663
2		NH_2	-465.1939325	-465.028973	-465.01754	-465.066287
3		OH	-485.0569954	-484.905332	-484.893887	-484.943156
4		F	-509.072149	-508.933168	-508.922853	-508.969148
5		CHO	-523.1382615	-522.98127	-522.969137	-523.020086
6		CN	-502.0721548	-501.925965	-501.913879	-501.965982
7		NO_2	-614.284553	-614.134233	-614.12153	-614.174806

Transition State, (Hartree)						
Entry	R_1	R_2	Total Electronic Energy	Sum of Electronic and Zero-Point Energies	Sum of Electronic and Thermal Enthalpies	Gibbs Free Energy
1	F	CH_3	-449.1324741	-448.956512	-448.945988	-448.991602
2		NH_2	-465.172752	-465.007902	-464.997697	-465.042023
3		OH	-485.0342287	-484.882084	-484.87218	-484.915939
4		F	-509.0494867	-508.909794	-508.900203	-508.943545
5		CHO	-523.1222599	-522.964722	-522.953971	-523.000125
6		CN	-502.0528739	-501.906224	-501.895686	-501.941346
7		NO_2	-614.2710983	-614.120025	-614.108817	-614.156858

Octatetraene [Starting Material], (Hartree)

Entry	R_1	R_2	Total Electronic Energy	Sum of Electronic and Zero-Point Energies	Sum of Electronic and Thermal Enthalpies	Gibbs Free Energy
1		CH_3	-463.2251862	-463.03174	-463.018888	-463.071456
2		NH_2	-479.2664311	-479.084323	-479.0716	-479.12361
3		OH	-499.1297011	-498.960423	-498.947832	-498.999807
4	CHO	F	-523.1442219	-522.98747	-522.975176	-523.026708
5		CHO	-537.209885	-537.035432	-537.022076	-537.076068
6		CN	-516.1436975	-515.980204	-515.966855	-516.021959
7		NO_2	-628.3558051	-628.188397	-628.174388	-628.231417

Transition State, (Hartree)						
Entry	R_1	R_2	Total Electronic Energy	Sum of Electronic and Zero-Point Energies	Sum of Electronic and Thermal Enthalpies	Gibbs Free Energy
1		CH_3	-463.2083063	-463.014857	-463.003146	-463.051439
2		NH_2	-479.2478859	-479.065459	-479.054071	-479.101339
3		OH	-499.1080466	-498.938451	-498.927246	-498.97427
4	CHO	F	-523.1246256	-522.967425	-522.956607	-523.002988
5		CHO	-537.1990928	-537.023884	-537.011947	-537.06106
6		CN	-516.1281056	-515.963851	-515.952055	-516.000882
7		NO_2	-628.3468901	-628.178348	-628.165831	-628.217208

Octatetraene [Starting Material], (Hartree)						
Entry	R_1	R_2	Total Electronic Energy	Sum of Electronic and Zero-Point Energies	Sum of Electronic and Thermal Enthalpies	Gibbs Free Energy
1		CH_3	-442.1615644	-441.979035	-441.966465	-442.018721
2		NH_2	-458.2026952	-458.031354	-458.019006	-458.069932
3		OH	-478.0660033	-477.907596	-477.895341	-477.946765
4	CN	F	-502.0803656	-501.934719	-501.923557	-501.971892
5		CHO	-516.1458671	-515.982056	-515.969051	-516.02215
6		CN	-495.079483	-494.92699	-494.913964	-494.968389
7		NO_2	-607.2916737	-607.134853	-607.121251	-607.17663

Transition State, (Hartree)						
Entry	R_1	R_2	Total Electronic Energy	Sum of Electronic and Zero-Point Energies	Sum of Electronic and Thermal Enthalpies	Gibbs Free Energy
1		CH_3	-442.1413612	-441.958645	-441.947278	-441.994717
2		NH_2	-458.1820312	-458.010246	-457.999229	-458.045557
3		OH	-478.0404036	-477.881469	-477.870615	-477.916774
4	CN	F	-502.0564291	-501.909914	-501.899438	-501.944979
5		CHO	-516.1310763	-515.966472	-515.954899	-516.003071
6		CN	-495.0588466	-494.905442	-494.894024	-494.941849
7		NO_2	-607.2766925	-607.118659	-607.106542	-607.156895

Octatetraene [Starting Material], (Hartree)						
Entry	R_1	R_2	Total Electronic Energy	Sum of Electronic and Zero-Point Energies	Sum of Electronic and Thermal Enthalpies	Gibbs Free Energy
1		CH_3	-554.3735638	-554.186531	-554.174114	-554.226024
2		NH_2	-570.4147983	-570.238723	-570.225697	-570.27909
3		OH	-590.2778754	-590.114852	-590.101874	-590.15601
4	NO_2	F	-614.2919282	-614.141601	-614.128841	-614.183202
5		CHO	-628.357532	-628.189392	-628.175625	-628.2314
6		CN	-607.2908619	-607.133628	-607.119897	-607.177209
7		NO_2	-719.5031421	-719.341664	-719.327364	-719.385292

Transition State, (Hartree)						
Entry	R_1	R_2	Total Electronic Energy	Sum of Electronic and Zero-Point Energies	Sum of Electronic and Thermal Enthalpies	Gibbs Free Energy
1	NO_2	CH_3	-554.3596279	-554.172274	-554.16025	-554.209559
2		NH_2	-570.4006013	-570.224482	-570.212646	-570.26164
3		OH	-590.2577871	-590.094236	-590.082634	-590.130956
4		F	-614.2733023	-614.122207	-614.110968	-614.158842
5		CHO	-628.3484451	-628.179545	-628.167194	-628.217726
6		CN	-607.2750634	-607.117034	-607.104824	-607.155083
7		NO_2	-719.4929734	-719.330632	-719.317806	-719.370064

4.7 1,8-substituted

Octatetraene [Starting Material], (Hartree)						
Entry	R_1	R_2	Total Electronic Energy	Sum of Electronic and Zero-Point Energies	Sum of Electronic and Thermal Enthalpies	Gibbs Free Energy
1	CH_3	CH_3	-389.2391428	-389.028401	-389.015199	-389.069414
2		NH_2	-405.2806251	-405.080388	-405.067563	-405.120257
3		OH	-425.1405015	-424.952598	-424.940141	-424.991987
4		F	-449.154962	-448.980096	-448.968697	-449.017984
5		CHO	-463.2277779	-463.034998	-463.021653	-463.075892
6		CN	-442.1641436	-441.982267	-441.97007	-442.021331
7		NO_2	-554.3763249	-554.189664	-554.176004	-554.231618

Transition State, (Hartree)						
Entry	R_1	R_2	Total Electronic Energy	Sum of Electronic and Zero-Point Energies	Sum of Electronic and Thermal Enthalpies	Gibbs Free Energy
1	CH_3	CH_3	-389.2204067	-389.007976	-388.997102	-389.042325
2		NH_2	-405.2614831	-405.059961	-405.04932	-405.094041
3		OH	-425.1201271	-424.931508	-424.920911	-424.965776
4		F	-449.134492	-448.958386	-448.948032	-448.992626
5		CHO	-463.2111896	-463.017011	-463.00566	-463.052573
6		CN	-442.1443071	-441.961126	-441.94993	-441.996601
7		NO_2	-554.3631783	-554.175654	-554.163776	-554.212427

Octatetraene [Starting Material], (Hartree)						
Entry	R_1	R_2	Total Electronic Energy	Sum of Electronic and Zero-Point Energies	Sum of Electronic and Thermal Enthalpies	Gibbs Free Energy
1	NH_2	NH_2	-421.3216644	-421.131899	-421.119545	-421.170248
2		OH	-441.1819402	-441.004763	-440.99256	-441.043894
3		F	-465.1966764	-465.031933	-465.02011	-465.070283
4		CHO	-479.2704901	-479.088392	-479.075373	-479.128354
5		CN	-458.2069055	-458.035376	-458.022765	-458.074549
6		NO_2	-570.419636	-570.24376	-570.230367	-570.284979

Transition State, (Hartree)						
Entry	R_1	R_2	Total Electronic Energy	Sum of Electronic and Zero-Point Energies	Sum of Electronic and Thermal Enthalpies	Gibbs Free Energy
1	NH_2	NH_2	-421.3008184	-421.110073	-421.099619	-421.143884
2		OH	-441.1608563	-440.983073	-440.972737	-441.016851
3		F	-465.1770782	-465.011667	-465.00165	-465.045385
4		CHO	-479.2581905	-479.074314	-479.063453	-479.109114
5		CN	-458.1911482	-458.018695	-458.007912	-458.053528

6		NO_2	-570.4141847	-570.237174	-570.225705	-570.273258

Octatetraene [Starting Material], (Hartree)						
Entry	R_1	R_2	Total Electronic Energy	Sum of Electronic and Zero-Point Energies	Sum of Electronic and Thermal Enthalpies	Gibbs Free Energy
1	OH	OH	-461.0417674	-460.876777	-460.865026	-460.915025
2		F	-485.0561952	-484.90383	-484.892364	-484.941805
3		CHO	-499.1289612	-498.959232	-498.946539	-498.999069
4		CN	-478.0652515	-477.906394	-477.894007	-477.946038
5		NO_2	-590.2774682	-590.114038	-590.100959	-590.155253

Transition State, (Hartree)						
Entry	R_1	R_2	Total Electronic Energy	Sum of Electronic and Zero-Point Energies	Sum of Electronic and Thermal Enthalpies	Gibbs Free Energy
1	OH	OH	-461.0217077	-460.856596	-460.846457	-460.890231
2		F	-485.0333862	-484.880645	-484.870804	-484.914321
3		CHO	-499.1089634	-498.938298	-498.927384	-498.97365
4		CN	-478.0448018	-477.88533	-477.874645	-477.920258
5		NO_2	-590.2652723	-590.101101	-590.089765	-590.1372

Octatetraene [Starting Material], (Hartree)						
Entry	R_1	R_2	Total Electronic Energy	Sum of Electronic and Zero-Point Energies	Sum of Electronic and Thermal Enthalpies	Gibbs Free Energy
1	F	F	-509.0706281	-508.93128	-508.920019	-508.969505
2		CHO	-523.1428111	-522.986101	-522.973541	-523.02668
3		CN	-502.07929	-501.932679	-501.920685	-501.971659
4		NO_2	-614.2911335	-614.14054	-614.127682	-614.182048

Transition State, (Hartree)						
Entry	R_1	R_2	Total Electronic Energy	Sum of Electronic and Zero-Point Energies	Sum of Electronic and Thermal Enthalpies	Gibbs Free Energy
1	F	F	-509.0455751	-508.905454	-508.895847	-508.939176
2		CHO	-523.1213939	-522.963449	-522.952666	-522.998931
3		CN	-502.0538252	-501.906628	-501.896172	-501.941611
4		NO_2	-614.2713922	-614.119922	-614.108648	-614.15662

Octatetraene [Starting Material], (Hartree)						
Entry	R_1	R_2	Total Electronic Energy	Sum of Electronic and Zero-Point Energies	Sum of Electronic and Thermal Enthalpies	Gibbs Free Energy
1	CHO	CHO	-537.2138395	-537.039202	-537.025611	-537.080526
2		CN	-516.1500607	-515.986329	-515.972983	-516.028353
3		NO_2	-628.3613137	-628.19347	-628.180179	-628.235263

Transition State, (Hartree)						
Entry	R_1	R_2	Total Electronic Energy	Sum of Electronic and Zero-Point Energies	Sum of Electronic and Thermal Enthalpies	Gibbs Free Energy
1	CHO	CHO	-537.1963266	-537.020628	-537.008697	-537.057686
2		CN	-516.1269269	-515.96245	-515.950749	-515.999268
3		NO_2	-628.3443478	-628.175569	-628.163096	-628.213922

Octatetraene [Starting Material], (Hartree)						
Entry	R_1	R_2	Total Electronic Energy	Sum of Electronic and Zero-Point Energies	Sum of Electronic and Thermal Enthalpies	Gibbs Free Energy

1	CN	CN	-495.0864125	-494.933421	-494.920442	-494.974598
2		NO_2	-607.2977085	-607.140089	-607.126395	-607.183414

Transition State, (Hartree)						
Entry	R_1	R_2	Total Electronic Energy	Sum of Electronic and Zero-Point Energies	Sum of Electronic and Thermal Enthalpies	Gibbs Free Energy
1	CN	CN	-495.0582366	-494.904282	-494.892951	-494.940473
2		NO_2	-607.2751045	-607.116994	-607.104847	-607.154858

Octatetraene [Starting Material], (Hartree)						
Entry	R_1	R_2	Total Electronic Energy	Sum of Electronic and Zero-Point Energies	Sum of Electronic and Thermal Enthalpies	Gibbs Free Energy
1	NO_2	NO_2	-719.5086531	-719.346981	-719.333326	-719.390498

Transition State, (Hartree)						
Entry	R_1	R_2	Total Electronic Energy	Sum of Electronic and Zero-Point Energies	Sum of Electronic and Thermal Enthalpies	Gibbs Free Energy
1	NO_2	NO_2	-719.4903689	-719.3278	-719.314946	-719.366917

4.8 2,3-substituted

Octatetraene [Starting Material], (Hartree)						
Entry	R_1	R_2	Total Electronic Energy	Sum of Electronic and Zero-Point Energies	Sum of Electronic and Thermal Enthalpies	Gibbs Free Energy
1	CH_3	CH_3	-389.2350549	-389.023724	-389.012196	-389.060199
2		NH_2	-405.2775788	-405.077457	-405.065185	-405.114824
3		OH	-425.1380986	-424.950415	-424.938355	-424.988257
4		F	-449.1554138	-448.980004	-448.968352	-448.968352
5		CHO	-463.2264231	-463.034485	-463.021214	-463.074265
6		CN	-442.1583523	-441.97632	-441.964511	-442.013519
7		NO_2	-554.3772419	-554.191802	-554.178976	-554.230542

Transition State, (Hartree)						
Entry	R_1	R_2	Total Electronic Energy	Sum of Electronic and Zero-Point Energies	Sum of Electronic and Thermal Enthalpies	Gibbs Free Energy
1	CH_3	CH_3	-389.2146814	-389.002744	-388.991778	-389.03726
2		NH_2	-405.2555387	-405.054523	-405.043811	-405.088639
3		OH	-425.1186077	-424.930387	-424.919819	-424.964507
4		F	-449.1362287	-448.96049	-448.950207	-448.994488
5		CHO	-463.2048606	-463.01145	-462.99997	-463.04725
6		CN	-442.140207	-441.957856	-441.946552	-441.993324
7		NO_2	-554.3532653	-554.166327	-554.15446	-554.202972

Octatetraene [Starting Material], (Hartree)						
Entry	R_1	R_2	Total Electronic Energy	Sum of Electronic and Zero-Point Energies	Sum of Electronic and Thermal Enthalpies	Gibbs Free Energy
1	NH_2	CH_3	-405.2784417	-405.078378	-405.066066	-405.116067
2		NH_2	-421.3203803	-421.131195	-421.119219	-421.1679
3		OH	-441.1792527	-441.002868	-440.99104	-441.039727
4		F	-465.1948148	-465.031049	-465.019455	-465.067964
5		CHO	-479.2686277	-479.087075	-479.074336	-479.125477
6		CN	-458.1997586	-458.028868	-458.016413	-458.06698

7		NO_2	-570.4169017	-570.241983	-570.228727	-570.281554

Transition State, (Hartree)						
Entry	R_1	R_2	Total Electronic Energy	Sum of Electronic and Zero-Point Energies	Sum of Electronic and Thermal Enthalpies	Gibbs Free Energy
1	NH_2	CH_3	-405.2539651	-405.053297	-405.042406	-405.087694
2		NH_2	-421.2950469	-421.105097	-421.094498	-421.139053
3		OH	-441.158761	-440.981681	-440.971258	-441.015518
4		F	-465.175244	-465.010805	-465.000661	-465.044578
5		CHO	-479.2445503	-479.062924	-479.051437	-479.098585
6		CN	-458.1808369	-458.009609	-457.998453	-458.044833
7		NO_2	-570.393341	-570.217672	-570.205865	-570.254669

Octatetraene [Starting Material], (Hartree)						
Entry	R_1	R_2	Total Electronic Energy	Sum of Electronic and Zero-Point Energies	Sum of Electronic and Thermal Enthalpies	Gibbs Free Energy
1	OH	CH_3	-425.1406862	-424.953265	-424.941144	-424.991151
2		NH_2	-441.1799997	-441.004008	-440.992059	-441.041173
3		OH	-461.0405927	-460.877453	-460.866454	-460.913509
4		F	-485.0567741	-484.90623	-484.895503	-484.941913
5		CHO	-499.1290989	-498.960116	-498.947593	-498.998423
6		CN	-478.0622716	-477.904509	-477.892058	-477.943767
7		NO_2	-590.2718906	-590.11001	-590.096846	-590.150396

Transition State, (Hartree)						
Entry	R_1	R_2	Total Electronic Energy	Sum of Electronic and Zero-Point Energies	Sum of Electronic and Thermal Enthalpies	Gibbs Free Energy
1	OH	CH_3	-425.1174972	-424.929341	-424.918778	-424.963454
2		NH_2	-441.1573654	-440.980148	-440.969798	-441.013868
3		OH	-461.0131736	-460.848906	-460.83873	-460.882584
4		F	-485.030923	-484.879166	-484.869283	-484.912735
5		CHO	-498.998423	-498.938575	-498.927514	-498.973946
6		CN	-478.0380296	-477.879552	-477.868637	-477.914619
7		NO_2	-590.2575962	-590.094566	-590.083072	-590.131504

Octatetraene [Starting Material], (Hartree)						
Entry	R_1	R_2	Total Electronic Energy	Sum of Electronic and Zero-Point Energies	Sum of Electronic and Thermal Enthalpies	Gibbs Free Energy
1	F	CH_3	-449.1571923	-448.982198	-448.970369	-449.019781
2		NH_2	-465.1952424	-465.031521	-465.019889	-465.068455
3		OH	-485.055641	-484.905106	-484.894364	-484.941172
4		F	-509.0707199	-508.9328	-508.922326	-508.968577
5		CHO	-523.1439215	-522.98754	-522.97527	-523.025874
6		CN	-502.0762461	-501.931076	-501.91883	-501.970704
7		NO_2	-614.2860064	-614.136317	-614.125259	-614.17333

Transition State, (Hartree)						
Entry	R_1	R_2	Total Electronic Energy	Sum of Electronic and Zero-Point Energies	Sum of Electronic and Thermal Enthalpies	Gibbs Free Energy
1	F	CH_3	-449.1340309	-448.958525	-448.948165	-448.992673
2		NH_2	-465.1719487	-465.007531	-464.997442	-465.041154
3		OH	-485.0294761	-484.877696	-484.86781	-484.911289
4		F	-509.0460192	-508.90673	-508.897145	-508.940225
5		CHO	-523.1232912	-522.966149	-522.955395	-523.001319

6		CN	-502.052339	-501.906237	-501.895678	-501.941105
7		NO_2	-614.2634776	-614.11297	-614.101751	-614.149163

Octatetraene [Starting Material], (Hartree)						
Entry	R_1	R_2	Total Electronic Energy	Sum of Electronic and Zero-Point Energies	Sum of Electronic and Thermal Enthalpies	Gibbs Free Energy
1	CHO	CH_3	-463.2247273	-463.032537	-463.019498	-463.071688
2		NH_2	-479.2662692	-479.08494	-479.072217	-479.123396
3		OH	-499.1257181	-498.956967	-498.94449	-498.995254
4		F	-523.1397389	-522.983551	-522.971313	-523.021781
5		CHO	-537.2136472	-537.040158	-537.026633	-537.080218
6		CN	-516.144265	-515.981011	-515.967874	-516.020505
7		NO_2	-628.3595023	-628.192342	-628.178343	-628.233824

Transition State, (Hartree)						
Entry	R_1	R_2	Total Electronic Energy	Sum of Electronic and Zero-Point Energies	Sum of Electronic and Thermal Enthalpies	Gibbs Free Energy
1	CHO	CH_3	-463.203549	-463.010056	-462.998554	-463.045987
2		NH_2	-479.2453738	-479.062925	-479.05165	-479.098486
3		OH	-499.1079325	-498.938164	-498.927054	-498.973584
4		F	-523.1239775	-522.96686	-522.956041	-523.002201
5		CHO	-537.1921636	-537.017108	-537.00519	-537.054191
6		CN	-516.1265611	-515.962748	-515.95093	-515.99949
7		NO_2	-628.3407468	-628.172087	-628.159779	-628.209932

Octatetraene [Starting Material], (Hartree)						
Entry	R_1	R_2	Total Electronic Energy	Sum of Electronic and Zero-Point Energies	Sum of Electronic and Thermal Enthalpies	Gibbs Free Energy
1	CN	CH_3	-442.1568224	-441.975341	-441.96253	-442.014852
2		NH_2	-458.1970352	-458.026158	-458.01371	-458.064139
3		OH	-478.0576038	-477.89966	-477.887298	-477.93827
4		F	-502.0725609	-501.927395	-501.916125	-501.964128
5		CHO	-516.1448822	-515.982133	-515.968765	-516.022321
6		CN	-495.0763465	-494.924512	-494.912236	-494.962943
7		NO_2	-607.2847553	-607.128063	-607.116118	-607.166307

Transition State, (Hartree)						
Entry	R_1	R_2	Total Electronic Energy	Sum of Electronic and Zero-Point Energies	Sum of Electronic and Thermal Enthalpies	Gibbs Free Energy
1	CN	CH_3	-442.1387527	-441.956597	-441.945143	-441.992564
2		NH_2	-458.1793179	-458.00832	-457.99711	-458.043611
3		OH	-478.0377382	-477.879296	-477.868407	-477.914396
4		F	-502.0530948	-501.907201	-501.896607	-501.942178
5		CHO	-516.1266091	-515.962656	-515.950927	-515.999202
6		CN	-495.0541933	-494.901271	-494.889715	-494.937591
7		NO_2	-607.2676781	-607.110379	-607.098177	-607.148056

Octatetraene [Starting Material], (Hartree)						
Entry	R_1	R_2	Total Electronic Energy	Sum of Electronic and Zero-Point Energies	Sum of Electronic and Thermal Enthalpies	Gibbs Free Energy
1	NO_2	CH_3	-554.3742134	-554.188942	-554.175214	-554.229802
2		NH_2	-570.4100398	-570.235972	-570.222432	-570.276519
3		OH	-590.2690416	-590.107125	-590.094022	-590.146927
4		F	-614.2824354	-614.133206	-614.120346	-614.173124

5		CHO	-628.3605982	-628.193496	-628.1795	-628.234822
6		CN	-607.2890622	-607.132643	-607.118945	-607.173511
7		NO_2	-719.50851	-719.347775	-719.333363	-719.389842

Transition State, (Hartree)						
Entry	R_1	R_2	Total Electronic Energy	Sum of Electronic and Zero-Point Energies	Sum of Electronic and Thermal Enthalpies	Gibbs Free Energy
1	NO_2	CH_3	-554.3511949	-554.164299	-554.152407	-554.201355
2		NH_2	-570.3938794	-570.21847	-570.206647	-570.255382
3		OH	-590.2468249	-590.08416	-590.072511	-590.12086
4		F	-614.2640731	-614.113299	-614.102126	-614.149606
5		CHO	-628.3407512	-628.172114	-628.159837	-628.210043
6		CN	-607.2672027	-607.109773	-607.097612	-607.147507
7		NO_2	-719.4829198	-719.320929	-719.30818	-719.359544

4.9 2,4-substituted

Octatetraene [Starting Material], (Hartree)						
Entry	R_1	R_2	Total Electronic Energy	Sum of Electronic and Zero-Point Energies	Sum of Electronic and Thermal Enthalpies	Gibbs Free Energy
1	CH_3	CH_3	-389.2321629	-389.020372	-389.008074	-389.058241
2		NH_2	-405.2753359	-405.074894	-405.062814	-405.112213
3		OH	-425.1364215	-424.948746	-424.936851	-424.985894
4		F	-449.1539935	-448.978494	-448.966826	-449.016076
5		CHO	-463.2250783	-463.032562	-463.019709	-463.071198
6		CN	-442.1556937	-441.973814	-441.961129	-442.012633
7		NO_2	-554.3710699	-554.185672	-554.172137	-554.227707

Transition State, (Hartree)						
Entry	R_1	R_2	Total Electronic Energy	Sum of Electronic and Zero-Point Energies	Sum of Electronic and Thermal Enthalpies	Gibbs Free Energy
1	CH_3	CH_3	-389.2136652	-389.002044	-388.990797	-389.037235
2		NH_2	-405.2540164	-405.053699	-405.0435	-405.087428
3		OH	-425.1166587	-424.928733	-424.918026	-424.963224
4		F	-449.1363775	-448.961133	-448.950732	-448.995447
5		CHO	-463.2094603	-463.015922	-463.004441	-463.051741
6		CN	-442.1380985	-441.955769	-441.944408	-441.991394
7		NO_2	-554.3555395	-554.168983	-554.156887	-554.206248

Octatetraene [Starting Material], (Hartree)						
Entry	R_1	R_2	Total Electronic Energy	Sum of Electronic and Zero-Point Energies	Sum of Electronic and Thermal Enthalpies	Gibbs Free Energy
1	NH_2	CH_3	-405.2745509	-405.074083	-405.0619	-405.111627
2		NH_2	-421.317702	-421.12835	-421.116443	-421.16523
3		OH	-441.1783481	-441.002159	-440.990343	-441.03899
4		F	-465.1951703	-465.031215	-465.01973	-465.067789
5		CHO	-479.2664126	-479.084719	-479.072151	-479.122652
6		CN	-458.1970158	-458.026431	-458.013901	-458.064932
7		NO_2	-570.4119302	-570.237402	-570.224151	-570.277454

Transition State, (Hartree)						
Entry	R_1	R_2	Total Electronic Energy	Sum of Electronic and Zero-Point Energies	Sum of Electronic and Thermal Enthalpies	Gibbs Free Energy

1	NH_2	CH_3	-405.253357	-405.052867	-405.041891	-405.087417
2		NH_2	-421.2937623	-421.104138	-421.093467	-421.138205
3		OH	-441.1566106	-440.979743	-440.969321	-441.013638
4		F	-465.1759551	-465.011821	-465.001607	-465.04577
5		CHO	-479.2483437	-479.065963	-479.054701	-479.101411
6		CN	-458.1770482	-458.00613	-457.99487	-458.041529
7		NO_2	-570.39418	-570.218956	-570.207084	-570.255733

Octatetraene [Starting Material], (Hartree)						
Entry	R_1	R_2	Total Electronic Energy	Sum of Electronic and Zero-Point Energies	Sum of Electronic and Thermal Enthalpies	Gibbs Free Energy
1	OH	CH_3	-425.136544	-424.948509	-424.936467	-424.986313
2		NH_2	-441.1799468	-441.00378	-440.991896	-441.040835
3		OH	-461.041068	-460.8774	-460.865777	-460.914308
4		F	-485.0584672	-484.907359	-484.895817	-484.945164
5		CHO	-499.1258816	-498.95725	-498.944788	-498.9954
6		CN	-478.0585248	-477.900587	-477.888995	-477.937192
7		NO_2	-590.2723021	-590.110283	-590.0973	-590.149895

Transition State, (Hartree)						
Entry	R_1	R_2	Total Electronic Energy	Sum of Electronic and Zero-Point Energies	Sum of Electronic and Thermal Enthalpies	Gibbs Free Energy
1	OH	CH_3	-425.1154204	-424.927432	-424.916752	-424.961936
2		NH_2	-441.1565189	-440.979566	-440.969184	-441.013442
3		OH	-461.0183089	-460.853909	-460.843817	-460.887562
4		F	-485.0372026	-484.885437	-484.875574	-484.919066
5		CHO	-499.108539	-498.939161	-498.928034	-498.974579
6		CN	-478.2495747	-478.092676	-478.08171	-478.127863
7		NO_2	-590.5083601	-590.347589	-590.335849	-590.384967

Octatetraene [Starting Material], (Hartree)						
Entry	R_1	R_2	Total Electronic Energy	Sum of Electronic and Zero-Point Energies	Sum of Electronic and Thermal Enthalpies	Gibbs Free Energy
1	F	CH_3	-449.1522121	-448.977165	-448.966255	-449.012953
2		NH_2	-465.1960202	-465.032206	-465.020666	-465.069178
3		OH	-485.0565892	-484.905481	-484.894154	-484.942275
4		F	-509.0730209	-508.934464	-508.924135	-508.969802
5		CHO	-523.14038	-522.984291	-522.972038	-523.022633
6		CN	-502.0725203	-501.927131	-501.915825	-501.963991
7		NO_2	-614.2861426	-614.13676	-614.124031	-614.176182

Transition State, (Hartree)						
Entry	R_1	R_2	Total Electronic Energy	Sum of Electronic and Zero-Point Energies	Sum of Electronic and Thermal Enthalpies	Gibbs Free Energy
1	F	CH_3	-449.1321609	-448.956831	-448.946424	-448.991149
2		NH_2	-465.173227	-465.008686	-464.998649	-465.04238
3		OH	-485.0346944	-484.883043	-484.873172	-484.916689
4		F	-509.0528934	-508.913794	-508.904221	-508.947346
5		CHO	-523.1254822	-522.968309	-522.957632	-523.003416
6		CN	-502.0530788	-501.907156	-501.896581	-501.942114
7		NO_2	-614.2702924	-614.119928	-614.108663	-614.156486

Octatetraene [Starting Material], (Hartree)						
Entry	R_1	R_2	Total Electronic Energy	Sum of Electronic and Zero-Point	Sum of Electronic and Thermal	Gibbs Free Energy

				Energies	Enthalpies	
1	CHO	CH_3	-463.2205283	-463.027921	-463.015048	-463.06687
2		NH_2	-479.2637675	-479.082327	-479.069711	-479.120669
3		OH	-499.1235452	-498.955038	-498.94252	-498.993487
4		F	-523.1393165	-522.982817	-522.97064	-523.021006
5		CHO	-537.2089437	-537.035084	-537.021762	-537.074913
6		CN	-516.1403559	-515.977438	-515.964216	-516.017315
7		NO_2	-628.3542111	-628.187347	-628.173366	-628.22912

Transition State, (Hartree)						
Entry	R_1	R_2	Total Electronic Energy	Sum of Electronic and Zero-Point Energies	Sum of Electronic and Thermal Enthalpies	Gibbs Free Energy
1	CHO	CH_3	-463.2061496	-463.012832	-463.001312	-463.048694
2		NH_2	-479.2462167	-479.063797	-479.052577	-479.099203
3		OH	-499.1079077	-498.938501	-498.927422	-498.973867
4		F	-523.1265923	-522.969941	-522.959135	-523.005264
5		CHO	-537.2006919	-537.025307	-537.01353	-537.061939
6		CN	-516.1274087	-515.963758	-515.951946	-516.000482
7		NO_2	-628.345073	-628.17712	-628.16458	-628.215562

Octatetraene [Starting Material], (Hartree)						
Entry	R_1	R_2	Total Electronic Energy	Sum of Electronic and Zero-Point Energies	Sum of Electronic and Thermal Enthalpies	Gibbs Free Energy
1	CN	CH_3	-442.1528398	-441.970887	-441.958216	-442.009905
2		NH_2	-458.1975529	-458.1975529	-458.014159	-458.064834
3		OH	-478.0578464	-477.899667	-477.88744	-477.937951
4		F	-502.0731231	-501.927649	-501.915533	-501.966546
5		CHO	-516.1407068	-515.977799	-515.964637	-516.017366
6		CN	-495.0720452	-494.919669	-494.906679	-494.959471
7		NO_2	-607.2862211	-607.130149	-607.116405	-607.171378

Transition State, (Hartree)						
Entry	R_1	R_2	Total Electronic Energy	Sum of Electronic and Zero-Point Energies	Sum of Electronic and Thermal Enthalpies	Gibbs Free Energy
1	CN	CH_3	-442.1366393	-441.954139	-441.942818	-441.989777
2		NH_2	-458.1771946	-458.005578	-457.994604	-458.040636
3		OH	-478.0379888	-477.879271	-477.868463	-477.914255
4		F	-502.056166	-501.910234	-501.899684	-501.945198
5		CHO	-516.129365	-515.965299	-515.953648	-516.001803
6		CN	-495.0559009	-494.902973	-494.891432	-494.939318
7		NO_2	-607.2729118	-607.115609	-607.103352	-607.153621

Octatetraene [Starting Material], (Hartree)						
Entry	R_1	R_2	Total Electronic Energy	Sum of Electronic and Zero-Point Energies	Sum of Electronic and Thermal Enthalpies	Gibbs Free Energy
1	NO_2	CH_3	-554.3718312	-554.185707	-554.172401	-554.225773
2		NH_2	-570.4129283	-570.238645	-570.225401	-570.277246
3		OH	-590.2726577	-590.110837	-590.097864	-590.149407
4		F	-614.285565	-614.136019	-614.123244	-614.176065
5		CHO	-628.3525475	-628.185571	-628.171652	-628.226798
6		CN	-607.2860545	-607.130108	-607.116484	-607.170011
7		NO_2	-719.5034278	-719.342885	-719.328597	-719.384222

Transition State, (Hartree)						
Entry	R_1	R_2	Total Electronic	Sum of Electronic	Sum of Electronic	Gibbs Free Energy

			Energy	and Zero-Point Energies	and Thermal Enthalpies	
1	NO_2	CH_3	-554.3558261	-554.168748	-554.156779	-554.205882
2		NH_2	-570.3966187	-570.220389	-570.208774	-570.256967
3		OH	-590.256647	-590.093434	-590.08195	-590.130067
4		F	-614.274788	-614.124396	-614.113177	-614.161012
5		CHO	-628.3483422	-628.179643	-628.167311	-628.217726
6		CN	-607.2742674	-607.116869	-607.104626	-607.154869
7		NO_2	-719.4909427	-719.329373	-719.316377	-719.369637

4.10 2,5-substituted

Octatetraene [Starting Material], (Hartree)						
Entry	R_1	R_2	Total Electronic Energy	Sum of Electronic and Zero-Point Energies	Sum of Electronic and Thermal Enthalpies	Gibbs Free Energy
1	CH_3	CH_3	-389.2367219	-389.026055	-389.01364	-389.063191
2		NH_2	-405.2755608	-405.07585	-405.06354	-405.113482
3		OH	-425.1350504	-424.947968	-424.935753	-424.986383
4		F	-449.1510415	-448.976125	-448.964407	-449.01341
5		CHO	-463.2242289	-463.031923	-463.018942	-463.071197
6		CN	-442.1548053	-441.973293	-441.960513	-442.012717
7		NO_2	-554.3751312	-554.188827	-554.175594	-554.175594

Transition State, (Hartree)						
Entry	R_1	R_2	Total Electronic Energy	Sum of Electronic and Zero-Point Energies	Sum of Electronic and Thermal Enthalpies	Gibbs Free Energy
1	CH_3	CH_3	-389.2135529	-389.0024	-388.991003	-389.038104
2		NH_2	-405.25344	-405.052808	-405.041822	-405.087664
3		OH	-425.1164102	-424.928789	-424.917976	-424.96362
4		F	-449.1360084	-448.960985	-448.950468	-448.995724
5		CHO	-463.2087776	-463.015736	-463.004052	-463.052198
6		CN	-442.1384317	-441.956197	-441.944729	-441.99226
7		NO_2	-554.3560483	-554.169485	-554.157323	-554.207359

Octatetraene [Starting Material], (Hartree)						
Entry	R_1	R_2	Total Electronic Energy	Sum of Electronic and Zero-Point Energies	Sum of Electronic and Thermal Enthalpies	Gibbs Free Energy
1	NH_2	CH_3	-405.2766556	-405.077104	-405.064779	-405.114526
2		NH_2	-421.316369	-421.127009	-421.11501	-421.164092
3		OH	-441.1769128	-440.999959	-440.988357	-441.036623
4		F	-465.191344	-465.027393	-465.015823	-465.064844
5		CHO	-479.2684444	-479.086608	-479.074146	-479.12428
6		CN	-458.1945885	-458.02393	-458.011453	-458.062193
7		NO_2	-570.4135075	-570.238602	-570.225456	-570.278262

Transition State, (Hartree)						
Entry	R_1	R_2	Total Electronic Energy	Sum of Electronic and Zero-Point Energies	Sum of Electronic and Thermal Enthalpies	Gibbs Free Energy
1	NH_2	CH_3	-405.2528088	-405.052617	-405.041606	-405.087333
2		NH_2	-421.2916653	-421.2916653	-421.091476	-421.136244
3		OH	-441.1548292	-440.978311	-440.967822	-441.012328
4		F	-465.1749284	-465.011002	-465.000803	-465.044951
5		CHO	-479.2487811	-479.06682	-479.055397	-479.102494
6		CN	-458.1786268	-458.007625	-457.99638	-458.043059

7		NO_2	-570.3965982	-570.221314	-570.209373	-570.258364

Octatetraene [Starting Material], (Hartree)						
Entry	R_1	R_2	Total Electronic Energy	Sum of Electronic and Zero-Point Energies	Sum of Electronic and Thermal Enthalpies	Gibbs Free Energy
1	OH	CH_3	-425.1360654	-424.949036	-424.936887	-424.986471
2		NH_2	-441.176742	-441.000281	-440.988475	-441.037612
3		OH	-461.0352351	-460.871867	-460.860168	-460.908832
4		F	-485.0516688	-484.900832	-484.889437	-484.937563
5		CHO	-499.129142	-498.960326	-498.947958	-498.99833
6		CN	-478.0582089	-477.900591	-477.888277	-477.938563
7		NO_2	-590.2766401	-590.114506	-590.101615	-590.153197

Transition State, (Hartree)						
Entry	R_1	R_2	Total Electronic Energy	Sum of Electronic and Zero-Point Energies	Sum of Electronic and Thermal Enthalpies	Gibbs Free Energy
1	OH	CH_3	-425.1145674	-424.92683	-424.916075	-424.961459
2		NH_2	-441.15257	-440.975914	-440.96541	-441.009908
3		OH	-461.016576	-460.85266	-460.842427	-460.886484
4		F	-485.036361	-484.885125	-484.875183	-484.918877
5		CHO	-499.1103091	-498.940792	-498.929749	-498.976146
6		CN	-478.039556	-477.880889	-477.870042	-477.916
7		NO_2	-590.2573007	-590.094277	-590.082773	-590.130997

Octatetraene [Starting Material], (Hartree)						
Entry	R_1	R_2	Total Electronic Energy	Sum of Electronic and Zero-Point Energies	Sum of Electronic and Thermal Enthalpies	Gibbs Free Energy
1	F	CH_3	-449.1509469	-448.976252	-448.964428	-449.013696
2		NH_2	-465.1912501	-465.027603	-465.015986	-465.064769
3		OH	-485.0517462	-484.900836	-484.889487	-484.937573
4		F	-509.0672222	-508.928611	-508.917558	-508.965219
5		CHO	-523.1426253	-522.986295	-522.974199	-523.024146
6		CN	-502.0713907	-501.926244	-501.91418	-501.964293
7		NO_2	-614.2893831	-614.139922	-614.127255	-614.178843

Transition State, (Hartree)						
Entry	R_1	R_2	Total Electronic Energy	Sum of Electronic and Zero-Point Energies	Sum of Electronic and Thermal Enthalpies	Gibbs Free Energy
1	F	CH_3	-449.131287	-448.956067	-448.945571	-448.990731
2		NH_2	-465.170854	-465.006346	-464.996276	-465.040095
3		OH	-485.0334083	-484.881908	-484.872014	-484.915566
4		F	-509.0525073	-508.91364	-508.904068	-508.947175
5		CHO	-523.1257369	-522.968785	-522.958024	-523.004026
6		CN	-502.0545506	-501.908366	-501.897822	-501.943319
7		NO_2	-614.2721526	-614.121659	-614.110423	-614.158338

Octatetraene [Starting Material], (Hartree)						
Entry	R_1	R_2	Total Electronic Energy	Sum of Electronic and Zero-Point Energies	Sum of Electronic and Thermal Enthalpies	Gibbs Free Energy
1	CHO	CH_3	-463.220493	-463.028677	-463.01555	-463.068083
2		NH_2	-479.265698	-479.083955	-479.071508	-479.121709
3		OH	-499.1252316	-498.956347	-498.943995	-498.994327
4		F	-523.138752	-522.982457	-522.970327	-523.020434
5		CHO	-537.2092264	-537.035579	-537.022196	-537.075501

6		CN	-516.1404016	-515.977506	-515.964345	-516.016903
7		NO_2	-628.3551348	-628.187692	-628.173948	-628.228374

Transition State, (Hartree)						
Entry	R_1	R_2	Total Electronic Energy	Sum of Electronic and Zero-Point Energies	Sum of Electronic and Thermal Enthalpies	Gibbs Free Energy
1	CHO	CH_3	-463.2068606	-463.013424	-463.001931	-463.049286
2		NH_2	-479.2475803	-479.065407	-479.054108	-479.100797
3		OH	-499.109999	-498.940224	-498.929251	-498.975461
4		F	-523.1278829	-522.970669	-522.960007	-523.005766
5		CHO	-537.1989298	-537.023909	-537.012044	-537.060755
6		CN	-516.1277671	-515.963865	-515.952177	-516.000409
7		NO_2	-628.3446567	-628.176438	-628.164034	-628.214773

Octatetraene [Starting Material], (Hartree)						
Entry	R_1	R_2	Total Electronic Energy	Sum of Electronic and Zero-Point Energies	Sum of Electronic and Thermal Enthalpies	Gibbs Free Energy
1	CN	CH_3	-442.1557912	-441.974867	-441.962008	-442.013238
2		NH_2	-458.1927466	-458.022759	-458.01018	-458.060997
3		OH	-478.0557585	-477.898141	-477.885802	-477.936016
4		F	-502.0684421	-501.923551	-501.911462	-501.961741
5		CHO	-516.1419908	-515.978762	-515.965685	-516.01798
6		CN	-495.071905	-494.920149	-494.90714	-494.959428
7		NO_2	-607.2897077	-607.133747	-607.120066	-607.174133

Transition State, (Hartree)						
Entry	R_1	R_2	Total Electronic Energy	Sum of Electronic and Zero-Point Energies	Sum of Electronic and Thermal Enthalpies	Gibbs Free Energy
1	CN	CH_3	-442.1366816	-441.954207	-441.942886	-441.98985
2		NH_2	-458.1778861	-458.006301	-457.995282	-458.041432
3		OH	-478.0393975	-477.880695	-477.869846	-477.91578
4		F	-502.0569993	-501.910869	-501.900335	-501.945829
5		CHO	-516.1282226	-515.964356	-515.952615	-516.001069
6		CN	-495.0567822	-494.903844	-494.892299	-494.940233
7		NO_2	-607.273967	-607.116564	-607.104355	-607.154503

Octatetraene [Starting Material], (Hartree)						
Entry	R_1	R_2	Total Electronic Energy	Sum of Electronic and Zero-Point Energies	Sum of Electronic and Thermal Enthalpies	Gibbs Free Energy
1	NO_2	CH_3	-554.3679416	-554.182068	-554.168755	-554.22162
2		NH_2	-570.6611418	-570.488396	-570.475226	-570.527903
3		OH	-590.2734254	-590.111355	-590.098495	-590.150068
4		F	-614.2861657	-614.13661	-614.123999	-614.175493
5		CHO	-628.3549426	-628.187519	-628.173743	-628.173743
6		CN	-607.2882734	-607.132081	-607.118438	-607.17265
7		NO_2	-719.7949706	-719.636609	-719.622321	-719.677958

Transition State, (Hartree)						
Entry	R_1	R_2	Total Electronic Energy	Sum of Electronic and Zero-Point Energies	Sum of Electronic and Thermal Enthalpies	Gibbs Free Energy
1	NO_2	CH_3	-554.3557458	-554.168944	-554.156944	-554.206156
2		NH_2	-570.650087	-570.476384	-570.464537	-570.513199
3		OH	-590.2584374	-590.095422	-590.083887	-590.132179
4		F	-614.2756839	-614.125313	-614.114051	-614.162016

5		CHO	-628.3466249	-628.178544	-628.16609	-628.216944
6		CN	-607.2750458	-607.117865	-607.105627	-607.155841
7		NO_2	-719.786951	-719.627834	-719.614752	-719.667901

4.11 2,6-substituted

Octatetraene [Starting Material], (Hartree)						
Entry	R_1	R_2	Total Electronic Energy	Sum of Electronic and Zero-Point Energies	Sum of Electronic and Thermal Enthalpies	Gibbs Free Energy
1	CH_3	CH_3	-389.238398	-389.02677	-389.014398	-389.064884
2		NH_2	-405.2782698	-405.077708	-405.065585	-405.115154
3		OH	-425.1383162	-424.950581	-424.93863	-424.987994
4		F	-449.1558274	-448.980685	-448.96893	-449.018328
5		CHO	-463.2251858	-463.03194	-463.019131	-463.070875
6		CN	-442.1599633	-441.977924	-441.965228	-442.017446
7		NO_2	-554.371987	-554.185529	-554.172137	-554.226439

Transition State, (Hartree)						
Entry	R_1	R_2	Total Electronic Energy	Sum of Electronic and Zero-Point Energies	Sum of Electronic and Thermal Enthalpies	Gibbs Free Energy
1	CH_3	CH_3	-389.2154541	-389.004102	-388.992718	-389.039772
2		NH_2	-405.2555169	-405.055177	-405.044116	-405.090291
3		OH	-425.1172417	-424.929749	-424.918872	-424.964558
4		F	-449.1358572	-448.960601	-448.950138	-448.995222
5		CHO	-463.2091777	-463.01604	-463.004402	-463.052287
6		CN	-442.1395336	-441.957141	-441.945742	-441.992974
7		NO_2	-554.3575506	-554.170693	-554.158586	-554.208551

Octatetraene [Starting Material], (Hartree)						
Entry	R_1	R_2	Total Electronic Energy	Sum of Electronic and Zero-Point Energies	Sum of Electronic and Thermal Enthalpies	Gibbs Free Energy
1	NH_2	CH_3	-405.2799182	-405.079238	-405.067151	-405.116231
2		NH_2	-421.3200398	-421.130414	-421.118529	-421.167192
3		OH	-441.1823659	-441.005818	-440.994031	-441.042709
4		F	-465.197095	-465.033051	-465.021546	-465.069863
5		CHO	-479.2650688	-479.08339	-479.070635	-479.122218
6		CN	-458.2010574	-458.030024	-458.017605	-458.068047
7		NO_2	-570.4127879	-570.237332	-570.224166	-570.27717

Transition State, (Hartree)						
Entry	R_1	R_2	Total Electronic Energy	Sum of Electronic and Zero-Point Energies	Sum of Electronic and Thermal Enthalpies	Gibbs Free Energy
1	NH_2	CH_3	-405.2551347	-405.054921	-405.04379	-405.089965
2		NH_2	-421.2961495	-421.106694	-421.09598	-421.140849
3		OH	-441.158421	-440.981643	-440.971138	-441.015669
4		F	-465.1761344	-465.011874	-465.001688	-465.045794
5		CHO	-479.2483154	-479.066242	-479.054859	-479.101865
6		CN	-458.179063	-458.007989	-457.996776	-458.043367
7		NO_2	-570.3969899	-570.221451	-570.209514	-570.259139

Octatetraene [Starting Material], (Hartree)						
Entry	R_1	R_2	Total Electronic Energy	Sum of Electronic and Zero-Point Energies	Sum of Electronic and Thermal Enthalpies	Gibbs Free Energy

1	OH	CH_3	-425.142827	-424.955687	-424.944329	-424.991684
2		NH_2	-441.1833737	-441.007019	-440.995164	-441.044036
3		OH	-461.045738	-460.882477	-460.870541	-460.921383
4		F	-485.0604117	-484.909689	-484.8981	-484.948017
5		CHO	-499.1272706	-498.958627	-498.946717	-498.996781
6		CN	-478.0643621	-477.90647	-477.894099	-477.945246
7		NO_2	-590.2755509	-590.113272	-590.100148	-590.153992

Transition State, (Hartree)						
Entry	R_1	R_2	Total Electronic Energy	Sum of Electronic and Zero-Point Energies	Sum of Electronic and Thermal Enthalpies	Gibbs Free Energy
1	OH	CH_3	-425.1169105	-424.92935	-424.918398	-424.966773
2		NH_2	-441.1579165	-440.98102	-440.970621	-441.01493
3		OH	-461.0200707	-460.855846	-460.845659	-460.889604
4		F	-485.0375874	-484.88579	-484.875961	-484.919404
5		CHO	-499.109724	-498.94021	-498.929161	-498.975544
6		CN	-478.0401652	-477.881488	-477.870661	-477.916509
7		NO_2	-590.2579352	-590.09479	-590.083245	-590.131907

Octatetraene [Starting Material], (Hartree)						
Entry	R_1	R_2	Total Electronic Energy	Sum of Electronic and Zero-Point Energies	Sum of Electronic and Thermal Enthalpies	Gibbs Free Energy
1	F	CH_3	-449.1583427	-448.983831	-448.972699	-449.020075
2		NH_2	-465.1994677	-465.03561	-465.024069	-465.072468
3		OH	-485.058016	-484.907074	-484.895669	-484.944043
4		F	-509.0753101	-508.93726	-508.926826	-508.972965
5		CHO	-523.1424048	-522.985858	-522.973608	-523.024315
6		CN	-502.0781644	-501.932922	-501.92072	-501.974022
7		NO_2	-614.2889086	-614.139193	-614.126322	-614.179754

Transition State, (Hartree)						
Entry	R_1	R_2	Total Electronic Energy	Sum of Electronic and Zero-Point Energies	Sum of Electronic and Thermal Enthalpies	Gibbs Free Energy
1	F	CH_3	-449.1334174	-448.958198	-448.947673	-448.993046
2		NH_2	-465.1745868	-465.010111	-465.000029	-465.043887
3		OH	-485.0357801	-484.884248	-484.874292	-484.918005
4		F	-509.0534845	-508.914163	-508.904634	-508.947676
5		CHO	-523.1255028	-522.968291	-522.957591	-523.003448
6		CN	-502.0553259	-501.909276	-501.898734	-501.944216
7		NO_2	-614.2728837	-614.122449	-614.111148	-614.159826

Octatetraene [Starting Material], (Hartree)						
Entry	R_1	R_2	Total Electronic Energy	Sum of Electronic and Zero-Point Energies	Sum of Electronic and Thermal Enthalpies	Gibbs Free Energy
1	CHO	CH_3	-463.2248604	-463.032357	-463.019434	-463.071349
2		NH_2	-479.2652354	-479.083863	-479.071172	-479.122525
3		OH	-499.1271134	-498.958322	-498.945822	-498.996835
4		F	-523.1408648	-522.984773	-522.972557	-523.02321
5		CHO	-537.2082997	-537.034236	-537.020797	-537.074538
6		CN	-516.1432295	-515.980294	-515.967076	-516.020207
7		NO_2	-628.35435	-628.186935	-628.173012	-628.228621

Transition State, (Hartree)						
Entry	R_1	R_2	Total Electronic Energy	Sum of Electronic and Zero-Point	Sum of Electronic and Thermal	Gibbs Free Energy

				Energies	Enthalpies	
1	CHO	CH_3	-463.2076058	-463.014294	-463.002682	-463.05039
2		NH_2	-479.2468696	-479.064861	-479.053451	-479.100523
3		OH	-499.1095064	-498.939915	-498.928862	-498.975326
4		F	-523.1264395	-522.969432	-522.958657	-523.004767
5		CHO	-537.1989817	-537.024179	-537.012194	-537.0612
6		CN	-516.1286815	-515.964771	-515.952974	-516.001546
7		NO_2	-628.3463673	-628.17811	-628.165581	-628.217064

Octatetraene [Starting Material], (Hartree)						
Entry	R_1	R_2	Total Electronic Energy	Sum of Electronic and Zero-Point Energies	Sum of Electronic and Thermal Enthalpies	Gibbs Free Energy
1	CN	CH_3	-442.1587942	-441.977035	-441.964195	-442.016821
2		NH_2	-458.2009584	-458.030128	-458.017654	-458.068537
3		OH	-478.0569842	-477.899747	-477.888688	-477.935963
4		F	-502.0750456	-501.929669	-501.918469	-501.966196
5		CHO	-516.1450194	-515.981783	-515.969289	-516.021206
6		CN	-495.0764673	-494.924504	-494.911338	-494.965208
7		NO_2	-607.2869748	-607.130098	-607.116394	-607.171478

Transition State, (Hartree)						
Entry	R_1	R_2	Total Electronic Energy	Sum of Electronic and Zero-Point Energies	Sum of Electronic and Thermal Enthalpies	Gibbs Free Energy
1	CN	CH_3	-442.1381758	-441.955778	-441.944391	-441.991552
2		NH_2	-458.1788138	-458.007417	-457.99637	-458.042574
3		OH	-478.0393076	-477.880825	-477.86991	-477.915959
4		F	-502.0566695	-501.910481	-501.899971	-501.945391
5		CHO	-516.1305439	-515.966355	-515.954726	-516.002857
6		CN	-495.0581626	-494.905071	-494.893555	-494.941389
7		NO_2	-607.2756773	-607.118184	-607.105964	-607.156375

Octatetraene [Starting Material], (Hartree)						
Entry	R_1	R_2	Total Electronic Energy	Sum of Electronic and Zero-Point Energies	Sum of Electronic and Thermal Enthalpies	Gibbs Free Energy
1	NO_2	CH_3	-554.3715277	-554.185275	-554.171919	-554.225269
2		NH_2	-570.4135689	-570.238411	-570.225273	-570.278261
3		OH	-590.2746267	-590.112444	-590.099417	-590.152383
4		F	-614.2873465	-614.137908	-614.125152	-614.177814
5		CHO	-628.3537813	-628.186919	-628.172882	-628.228989
6		CN	-607.2886065	-607.132623	-607.118806	-607.174341
7		NO_2	-719.4996285	-719.339109	-719.324616	-719.382356

Transition State, (Hartree)						
Entry	R_1	R_2	Total Electronic Energy	Sum of Electronic and Zero-Point Energies	Sum of Electronic and Thermal Enthalpies	Gibbs Free Energy
1	NO_2	CH_3	-554.3571213	-554.170333	-554.158227	-554.207866
2		NH_2	-570.3978483	-570.221889	-570.210206	-570.258491
3		OH	-590.2589492	-590.095671	-590.084201	-590.132167
4		F	-614.2752962	-614.124552	-614.113391	-614.160987
5		CHO	-628.3473282	-628.178875	-628.166482	-628.217076
6		CN	-607.2765131	-607.119067	-607.106866	-607.156937
7		NO_2	-719.4939556	-719.332147	-719.319241	-719.371851

4.12 2,7-substituted

Octatetraene [Starting Material], (Hartree)						
Entry	R_1	R_2	Total Electronic Energy	Sum of Electronic and Zero-Point Energies	Sum of Electronic and Thermal Enthalpies	Gibbs Free Energy
1	CH_3	CH_3	-389.2342196	-389.021484	-389.009456	-389.059112
2		NH_2	-405.2758008	-405.07488	-405.062925	-405.112317
3		OH	-425.136623	-424.94877	-424.936931	-424.986392
4		F	-449.1539698	-448.978102	-448.966587	-449.01538
5		CHO	-463.2208177	-463.027699	-463.015011	-463.066748
6		CN	-442.1541591	-441.971793	-441.959251	-442.01113
7		NO_2	-554.3671972	-554.180972	-554.167709	-554.221958

Transition State, (Hartree)						
Entry	R_1	R_2	Total Electronic Energy	Sum of Electronic and Zero-Point Energies	Sum of Electronic and Thermal Enthalpies	Gibbs Free Energy
1	CH_3	CH_3	-389.2176847	-389.006387	-388.995116	-389.04164
2		NH_2	-405.256599	-405.056485	-405.045378	-405.09139
3		OH	-425.1179615	-424.930294	-424.919508	-424.96489
4		F	-449.1353857	-448.959952	-448.949574	-448.994309
5		CHO	-463.2102024	-463.01709	-463.005444	-463.053397
6		CN	-442.1407035	-441.958384	-441.946931	-441.994525
7		NO_2	-554.3597846	-554.173094	-554.160963	-554.210891

Octatetraene [Starting Material], (Hartree)						
Entry	R_1	R_2	Total Electronic Energy	Sum of Electronic and Zero-Point Energies	Sum of Electronic and Thermal Enthalpies	Gibbs Free Energy
1	NH2	NH_2	-421.3173175	-421.127598	-421.115865	-421.16438
2		OH	-441.1800874	-441.003023	-440.991374	-441.039984
3		F	-465.1955336	-465.031219	-465.019804	-465.068197
4		CHO	-479.2622396	-479.080284	-479.067796	-479.11878
5		CN	-458.1958511	-458.024892	-458.01254	-458.06355
6		NO_2	-570.4094729	-570.234302	-570.221294	-570.274315

Transition State, (Hartree)						
Entry	R_1	R_2	Total Electronic Energy	Sum of Electronic and Zero-Point Energies	Sum of Electronic and Thermal Enthalpies	Gibbs Free Energy
1	NH2	NH_2	-421.2946542	-421.105559	-421.094687	-421.140041
2		OH	-441.1568545	-440.980253	-440.96975	-441.014358
3		F	-465.1743051	-465.010233	-464.999987	-465.044316
4		CHO	-479.2499435	-479.068018	-479.056543	-479.103928
5		CN	-458.1810126	-458.009809	-457.998577	-458.045397
6		NO_2	-570.4004215	-570.224846	-570.21295	-570.262043

Octatetraene [Starting Material], (Hartree)						
Entry	R_1	R_2	Total Electronic Energy	Sum of Electronic and Zero-Point Energies	Sum of Electronic and Thermal Enthalpies	Gibbs Free Energy
1	OH	OH	-461.0431417	-460.879279	-460.867515	-460.91718
2		F	-485.0587003	-484.907369	-484.89682	-484.943028
3		CHO	-499.1251538	-498.955782	-498.943473	-498.994195
4		CN	-478.059018	-477.900694	-477.888425	-477.939902
5		NO_2	-590.2716818	-590.109356	-590.096455	-590.149348

Transition State, (Hartree)

Entry	R_1	R_2	Total Electronic Energy	Sum of Electronic and Zero-Point Energies	Sum of Electronic and Thermal Enthalpies	Gibbs Free Energy
1	OH	OH	-461.0188259	-460.85485	-460.844615	-460.88875
2		F	-485.0359971	-484.884442	-484.874539	-484.91822
3		CHO	-499.1120324	-498.942451	-498.93139	-498.978005
4		CN	-478.0423173	-477.88361	-477.872735	-477.918883
5		NO_2	-590.2616717	-590.098663	-590.087103	-590.135594

Octatetraene [Starting Material], (Hartree)						
Entry	R_1	R_2	Total Electronic Energy	Sum of Electronic and Zero-Point Energies	Sum of Electronic and Thermal Enthalpies	Gibbs Free Energy
1	F	F	-509.0739018	-508.934888	-508.923869	-508.971937
2		CHO	-523.1400455	-522.983283	522.971223	-523.021701
3		CN	-502.0739674	-501.928425	-501.917288	-501.965304
4		NO_2	-614.2863914	-614.136589	-614.123967	-614.17649

Transition State, (Hartree)						
Entry	R_1	R_2	Total Electronic Energy	Sum of Electronic and Zero-Point Energies	Sum of Electronic and Thermal Enthalpies	Gibbs Free Energy
1	F	F	-509.0523128	-508.913161	-508.903568	-508.946848
2		CHO	-523.126919	-522.969963	-522.959182	-523.005404
3		CN	-502.0570214	-501.910835	-501.900288	-501.945945
4		NO_2	-614.2757938	-614.125402	-614.114133	-614.16232

Octatetraene [Starting Material], (Hartree)						
Entry	R_1	R_2	Total Electronic Energy	Sum of Electronic and Zero-Point Energies	Sum of Electronic and Thermal Enthalpies	Gibbs Free Energy
1	CHO	CHO	-537.2061597	-537.032358	-537.019076	-537.072868
2		CN	-516.1399205	-515.976807	-515.963729	-516.017062
3		NO_2	-628.3528704	-628.185758	-628.171994	-628.227441

Transition State, (Hartree)						
Entry	R_1	R_2	Total Electronic Energy	Sum of Electronic and Zero-Point Energies	Sum of Electronic and Thermal Enthalpies	Gibbs Free Energy
1	CHO	CHO	-537.1981954	-537.023506	-537.011473	-537.060869
2		CN	-516.1288838	-515.965083	-515.95329	-516.002046
3		NO_2	-628.3469263	-628.178908	-628.166377	-628.218128

Octatetraene [Starting Material], (Hartree)						
Entry	R_1	R_2	Total Electronic Energy	Sum of Electronic and Zero-Point Energies	Sum of Electronic and Thermal Enthalpies	Gibbs Free Energy
1	CN	CN	-495.0738787	-494.921816	-494.909689	-494.960481
2		NO_2	-607.2860807	-607.129708	-607.116054	-607.17192

Transition State, (Hartree)						
Entry	R_1	R_2	Total Electronic Energy	Sum of Electronic and Zero-Point Energies	Sum of Electronic and Thermal Enthalpies	Gibbs Free Energy
1	CN	CN	-495.0588604	-494.905841	-494.894322	-494.942332
2		NO_2	-607.276998	-607.119494	-607.107305	-607.157544

Octatetraene [Starting Material], (Hartree)						
Entry	R_1	R_2	Total Electronic Energy	Sum of Electronic and Zero-Point	Sum of Electronic and Thermal	Gibbs Free Energy

				Energies	Enthalpies	
1	NO_2	NO_2	-719.49726	-719.336909	-719.322469	-719.380978

Transition State, (Hartree)						
Entry	R_1	R_2	Total Electronic Energy	Sum of Electronic and Zero-Point Energies	Sum of Electronic and Thermal Enthalpies	Gibbs Free Energy
1	NO_2	NO_2	-719.4947965	-719.333272	-719.320324	-719.373183

4.13 3,4-substituted

Octatetraene [Starting Material], (Hartree)						
Entry	R_1	R_2	Total Electronic Energy	Sum of Electronic and Zero-Point Energies	Sum of Electronic and Thermal Enthalpies	Gibbs Free Energy
1	CH_3	CH_3	-389.2338848	-389.023087	-389.010401	-389.061089
2		NH_2	-405.2774039	-405.077036	-405.064686	-405.114371
3		OH	-425.14047	-424.952781	-424.940697	-424.989702
4		F	-449.1573952	-448.982837	-448.971617	-449.018566
5		CHO	-463.2211275	-463.029208	-463.015923	-463.068453
6		CN	-442.1601859	-441.97883	-441.965864	-442.018207
7		NO_2	-554.36988	-554.183927	-554.170328	-554.223994

Transition State, (Hartree)						
Entry	R_1	R_2	Total Electronic Energy	Sum of Electronic and Zero-Point Energies	Sum of Electronic and Thermal Enthalpies	Gibbs Free Energy
1	CH_3	CH_3	-389.2077263	-388.995794	-388.984807	-389.030489
2		NH_2	-405.2495541	-405.048476	-405.037819	-405.082464
3		OH	-425.1140695	-424.926002	-424.915444	-424.959954
4		F	-449.1337623	-448.958203	-448.947887	-448.992174
5		CHO	-463.198808	-463.005177	-462.993713	-463.041082
6		CN	-442.1358521	-441.953373	-441.942119	-441.988649
7		NO_2	-554.3485199	-554.161451	-554.14957	-554.197935

Octatetraene [Starting Material], (Hartree)						
Entry	R_1	R_2	Total Electronic Energy	Sum of Electronic and Zero-Point Energies	Sum of Electronic and Thermal Enthalpies	Gibbs Free Energy
1	NH_2	CH_3	-405.2759789	-405.075316	-405.063155	-405.112069
2		NH_2	-421.3164309	-421.126841	-421.114853	-421.163463
3		OH	-441.1805893	-441.004049	-440.992214	-441.040604
4		F	-465.1969622	-465.032936	-465.021287	-465.069533
5		CHO	-479.2663968	-479.084978	-479.072214	-479.122992
6		CN	-458.2070126	-458.036051	-458.023525	-458.07401
7		NO_2	-570.422265	-570.247359	-570.234274	-570.286305

Transition State, (Hartree)						
Entry	R_1	R_2	Total Electronic Energy	Sum of Electronic and Zero-Point Energies	Sum of Electronic and Thermal Enthalpies	Gibbs Free Energy
1	NH_2	CH_3	-405.2483887	-405.047249	-405.036554	-405.081336
2		NH_2	-421.2928043	-421.102332	-421.092092	-421.135832
3		OH	-441.154632	-440.977538	-440.967178	-441.011191
4		F	-465.1732959	-465.008697	-464.998639	-465.042262
5		CHO	-479.2428656	-479.060063	-479.048997	-479.095079
6		CN	-458.1796263	-458.007839	-457.996925	-458.042668
7		NO_2	-570.390836	-570.21492	-570.203302	-570.251115

Octatetraene [Starting Material], (Hartree)						
Entry	R_1	R_2	Total Electronic Energy	Sum of Electronic and Zero-Point Energies	Sum of Electronic and Thermal Enthalpies	Gibbs Free Energy

1	OH	CH_3	-425.1368797	-424.949078	-424.937018	-424.986143
2		NH_2	-441.180528	-441.004206	-440.992315	-441.040795
3		OH	-461.0342281	-460.870903	-460.859058	-460.907634
4		F	-485.0510541	-484.899952	-484.888447	-484.936465
5		CHO	-499.1265333	-498.958144	-498.945469	-498.996282
6		CN	-478.0593775	-477.900954	-477.88867	-477.938541
7		NO_2	-478.035171	-477.876565	-477.865702	-477.911458

Transition State, (Hartree)						
Entry	R_1	R_2	Total Electronic Energy	Sum of Electronic and Zero-Point Energies	Sum of Electronic and Thermal Enthalpies	Gibbs Free Energy
1	OH	CH_3	-425.1122656	-424.924064	-424.913511	-424.958057
2		NH_2	-441.1534817	-440.976343	-440.965957	-441.010048
3		OH	-461.0079252	-460.843902	-460.833608	-460.877608
4		F	-485.0277619	-484.876322	-484.866327	-484.909946
5		CHO	-499.1047091	-498.934943	-498.923932	-498.970161
6		CN	-478.035171	-477.876565	-477.865702	-477.911458
7		NO_2	-590.2666753	-590.10492	-590.091571	-590.144498

Octatetraene [Starting Material], (Hartree)						
Entry	R_1	R_2	Total Electronic Energy	Sum of Electronic and Zero-Point Energies	Sum of Electronic and Thermal Enthalpies	Gibbs Free Energy
1	F	CH_3	-449.1545685	-448.97932	-448.967519	-449.016271
2		NH_2	-465.1965587	-465.03254	-465.020924	-465.06911
3		OH	-485.0510703	-484.899996	-484.888496	-484.936517
4		F	-509.0664767	-508.927982	-508.916662	-508.964739
5		CHO	-523.1412768	-522.985435	-522.973026	-523.023581
6		CN	-502.0731139	-501.927922	-501.916475	-501.964687
7		NO_2	-614.2801382	-614.130522	-614.117648	-614.169795

Transition State, (Hartree)						
Entry	R_1	R_2	Total Electronic Energy	Sum of Electronic and Zero-Point Energies	Sum of Electronic and Thermal Enthalpies	Gibbs Free Energy
1	F	CH_3	-449.1311174	-448.95552	-448.945214	-448.989499
2		NH_2	-465.1713192	-465.006399	-464.996429	-465.039853
3		OH	-485.0268749	-484.875064	-484.865206	-484.908497
4		F	-509.0448733	-508.905781	-508.896183	-508.939177
5		CHO	-523.1214903	-522.96434	-522.953609	-522.999446
6		CN	-502.0508517	-501.904661	-501.894142	-501.939379
7		NO_2	-614.25997	-614.109974	-614.098602	-614.146342

Octatetraene [Starting Material], (Hartree)						
Entry	R_1	R_2	Total Electronic Energy	Sum of Electronic and Zero-Point Energies	Sum of Electronic and Thermal Enthalpies	Gibbs Free Energy
1	CHO	CH_3	-463.2191027	-463.026752	-463.013674	-463.065592
2		NH_2	-479.2673903	-479.085881	-479.073097	-479.124286
3		OH	-499.1279843	-498.958772	-498.946224	-498.996988
4		F	-523.1418104	-522.985091	-522.972682	-523.023621
5		CHO	-537.2008563	-537.027113	-537.013505	-537.067257
6		CN	-516.1408622	-515.977918	-515.964582	-516.017483
7		NO_2	-628.3498049	-628.182466	-628.168446	-628.223602

Transition State, (Hartree)						
Entry	R_1	R_2	Total Electronic Energy	Sum of Electronic and Zero-Point Energies	Sum of Electronic and Thermal Enthalpies	Gibbs Free Energy
1	CHO	CH_3	-463.1978628	-463.004224	-462.992743	-463.040077
2		NH_2	-479.2433606	-479.060814	-479.049685	-479.095885
3		OH	-499.1057686	-498.935981	-498.925025	-498.971085
4		F	-523.1234749	-522.966441	-522.955693	-523.001525

5		CHO	-537.1868115	-537.011705	-536.999867	-537.0484
6		CN	-516.1229115	-515.958974	-515.947245	-515.99541
7		NO_2	-628.3348912	-628.166792	-628.154366	-628.204624

Octatetraene [Starting Material], (Hartree)						
Entry	R_1	R_2	Total Electronic Energy	Sum of Electronic and Zero-Point Energies	Sum of Electronic and Thermal Enthalpies	Gibbs Free Energy
1	CN	CH_3	-442.159266	-441.97799	-441.96508	-442.017023
2		NH_2	-458.2086292	-458.038138	-458.025567	-458.076131
3		OH	-478.0620223	-477.904225	-477.89182	-477.942293
4		F	-502.0756244	-501.930221	-501.918002	-501.96842
5		CHO	-516.1421829	-515.979282	-515.966047	-516.018346
6		CN	-495.0729397	-494.921113	-494.907956	-494.96033
7		NO_2	-607.2817635	-607.125548	-607.11175	-607.165904

Transition State, (Hartree)						
Entry	R_1	R_2	Total Electronic Energy	Sum of Electronic and Zero-Point Energies	Sum of Electronic and Thermal Enthalpies	Gibbs Free Energy
1	CN	CH_3	-442.1345906	-441.951978	-441.940761	-441.987196
2		NH_2	-458.1792917	-458.007733	-457.996774	-458.042584
3		OH	-478.0360046	-477.877313	-477.866486	-477.912125
4		F	-502.0526651	-501.906533	-501.896029	-501.941225
5		CHO	-516.1229192	-515.959016	-515.94727	-515.995525
6		CN	-495.050406	-494.897922	-494.886347	-494.934083
7		NO_2	-607.2616115	-607.104628	-607.092334	-607.142169

Octatetraene [Starting Material], (Hartree)						
Entry	R_1	R_2	Total Electronic Energy	Sum of Electronic and Zero-Point Energies	Sum of Electronic and Thermal Enthalpies	Gibbs Free Energy
1	NO_2	CH_3	-554.3681235	-554.182059	-554.168491	-554.222066
2		NH_2	-570.4236022	-570.248192	-570.235245	-570.287156
3		OH	-590.2675278	-590.105486	-590.092198	-590.145542
4		F	-614.2801598	-614.130862	-614.1177	-614.171034
5		CHO	-628.3492227	-628.182734	-628.16843	-628.2242
6		CN	-607.2793668	-607.12367	-607.109535	-607.165644
7		NO_2	-719.4896692	-719.329465	-719.314854	-719.371162

Transition State, (Hartree)						
Entry	R_1	R_2	Total Electronic Energy	Sum of Electronic and Zero-Point Energies	Sum of Electronic and Thermal Enthalpies	Gibbs Free Energy
1	NO_2	CH_3	-554.3462917	-554.159386	-554.147433	-554.196086
2		NH_2	-570.3907048	-570.214549	-570.202988	-570.250847
3		OH	-590.2567405	-590.093249	-590.082117	-590.129368
4		F	-614.2611463	-614.11096	-614.099683	-614.147128
5		CHO	-628.3341441	-628.165855	-628.153515	-628.203501
6		CN	-607.2607331	-607.103885	-607.091591	-607.141472
7		NO_2	-719.4743171	-719.313258	-719.300276	-719.351828

4.14 3,5-substituted

Octatetraene [Starting Material], (Hartree)						
Entry	R_1	R_2	Total Electronic Energy	Sum of Electronic and Zero-Point Energies	Sum of Electronic and Thermal Enthalpies	Gibbs Free Energy
1	CH_3	CH_3	-389.2362773	-389.025124	-389.012617	-389.062977
2		NH_2	-405.2797064	-405.079459	-405.067212	-405.116523
3		OH	-425.1408093	-424.953255	-424.941123	-424.990694
4		F	-449.1568673	-448.982191	-448.970214	-449.020022
5		CHO	-463.2282553	-463.035798	-463.022857	-463.074224

6		CN	-442.1594662	-441.977724	-441.964932	-442.016261
7		NO_2	-554.3748695	-554.189414	-554.175788	-554.22969

Transition State, (Hartree)						
Entry	R_1	R_2	Total Electronic Energy	Sum of Electronic and Zero-Point Energies	Sum of Electronic and Thermal Enthalpies	Gibbs Free Energy
1	CH_3	CH_3	-389.062977	-388.999623	-388.988187	-389.035534
2		NH_2	-405.25134	-405.050843	-405.039821	-405.085885
3		OH	-425.1144017	-424.926682	-424.916709	-424.960194
4		F	-449.020022	-448.958791	-448.948328	-448.993314
5		CHO	-463.2058908	-463.012622	-463.001072	-463.048458
6		CN	-442.016261	-441.953321	-441.941914	-441.98907
7		NO_2	-554.3530896	-554.166352	-554.15422	-554.203882

Octatetraene [Starting Material], (Hartree)						
Entry	R_1	R_2	Total Electronic Energy	Sum of Electronic and Zero-Point Energies	Sum of Electronic and Thermal Enthalpies	Gibbs Free Energy
1	NH_2	CH_3	-405.2764512	-405.076248	-405.064013	-405.113406
2		NH_2	-421.3203128	-421.13155	-421.119436	-421.16851
3		OH	-441.1813836	-441.005075	-440.993135	-441.042282
4		F	-465.1975185	-465.033576	-465.021959	-465.0704
5		CHO	-479.2685493	-479.086883	-479.074213	-479.124894
6		CN	-458.2004774	-458.030155	-458.017543	-458.068381
7		NO_2	-570.4155634	-570.241009	-570.227681	-570.280661

Transition State, (Hartree)						
Entry	R_1	R_2	Total Electronic Energy	Sum of Electronic and Zero-Point Energies	Sum of Electronic and Thermal Enthalpies	Gibbs Free Energy
1	NH_2	CH_3	-405.2506957	-405.050399	-405.039348	-405.085442
2		NH_2	-421.2913714	-421.101564	-421.091009	-421.135361
3		OH	-441.1552192	-440.978212	-440.967834	-441.011963
4		F	-465.1745974	-465.010249	-465.000187	-465.043849
5		CHO	-479.2456213	-479.063163	-479.051947	-479.098434
6		CN	-458.1759278	-458.004602	-457.993544	-458.039652
7		NO_2	-570.3934699	-570.217692	-570.205955	-570.254386

Octatetraene [Starting Material], (Hartree)						
Entry	R_1	R_2	Total Electronic Energy	Sum of Electronic and Zero-Point Energies	Sum of Electronic and Thermal Enthalpies	Gibbs Free Energy
1	OH	CH_3	-425.1368071	-424.949403	-424.937322	-424.986618
2		NH_2	-441.1765296	-441.001448	-440.989196	-441.038972
3		OH	-461.0418695	-460.878326	-460.86658	-460.915193
4		F	-485.0574961	-484.906598	-484.895092	-484.943584
5		CHO	-499.1280299	-498.959123	-498.946628	-498.997028
6		CN	-478.0601772	-477.902474	-477.890041	-477.940665
7		NO_2	-590.2746213	-590.113089	-590.099858	-590.152959

Transition State, (Hartree)						
Entry	R_1	R_2	Total Electronic Energy	Sum of Electronic and Zero-Point Energies	Sum of Electronic and Thermal Enthalpies	Gibbs Free Energy
1	OH	CH_3	-425.1129303	-424.925327	-424.914463	-424.95992
2		NH_2	-441.1537835	-440.977096	-440.966687	-441.010865
3		OH	-461.0163158	-460.852152	-460.84198	-460.885787
4		F	-485.0352472	-484.883833	-484.873919	-484.917404
5		CHO	-499.1068692	-498.937391	-498.92632	-498.972649
6		CN	-478.0360191	-477.877603	-477.866687	-477.912617
7		NO_2	-590.2533186	-590.090534	-590.078935	-590.127143

Octatetraene [Starting Material], (Hartree)

Entry	R_1	R_2	Total Electronic Energy	Sum of Electronic and Zero-Point Energies	Sum of Electronic and Thermal Enthalpies	Gibbs Free Energy
1	F	CH_3	-449.1550042	-448.979683	-448.967942	-449.016716
2		NH_2	-465.1971975	-465.033361	-465.021793	-465.069908
3		OH	-485.0592993	-484.908728	-484.897986	-484.943951
4		F	-509.0742144	-508.936382	-508.925828	-508.971932
5		CHO	-523.1443502	-522.988137	-522.975896	-523.026052
6		CN	-502.0764173	-501.931075	-501.918895	-501.969386
7		NO_2	-614.2903628	-614.141323	-614.128402	-614.181049

Transition State, (Hartree)						
Entry	R_1	R_2	Total Electronic Energy	Sum of Electronic and Zero-Point Energies	Sum of Electronic and Thermal Enthalpies	Gibbs Free Energy
1	F	CH_3	-449.1318618	-448.956284	-448.945969	-448.990326
2		NH_2	-465.1724077	-465.008003	-464.997956	-465.04158
3		OH	-485.0344064	-484.882988	-484.873092	-484.916553
4		F	-509.0526317	-508.913492	-508.903957	-508.94688
5		CHO	-523.12487	-522.967951	-522.957197	-523.003096
6		CN	-502.0534381	-501.907384	-501.896873	-501.942152
7		NO_2	-614.2708742	-614.120418	-614.109221	-614.156789

Octatetraene [Starting Material], (Hartree)						
Entry	R_1	R_2	Total Electronic Energy	Sum of Electronic and Zero-Point Energies	Sum of Electronic and Thermal Enthalpies	Gibbs Free Energy
1	CHO	CH_3	-463.2252902	-463.032681	-463.019634	-463.071868
2		NH_2	-479.267101	-479.085741	-479.07293	-479.124223
3		OH	-479.098577	-498.958495	-498.946629	-498.995625
4		F	-523.142685	-522.986378	-522.974757	-523.023571
5		CHO	-537.2145804	-537.040683	-537.027205	-537.08058
6		CN	-516.1444614	-515.981105	-515.96784	-516.020823
7		NO_2	-628.3583319	-628.191061	-628.17704	-628.232276

Transition State, (Hartree)						
Entry	R_1	R_2	Total Electronic Energy	Sum of Electronic and Zero-Point Energies	Sum of Electronic and Thermal Enthalpies	Gibbs Free Energy
1	CHO	CH_3	-463.2062078	-463.012861	-463.001316	-463.04876
2		NH_2	-479.2459332	-479.063417	-479.052266	-479.098577
3		OH	-499.1082573	-498.938658	-498.927651	-498.973768
4		F	-523.126967	-522.969845	-522.959182	-523.004796
5		CHO	-537.1997629	-537.024925	-537.013059	-537.061551
6		CN	-516.1281377	-515.964062	-515.952394	-516.000441
7		NO_2	-628.3459022	-628.17751	-628.165152	-628.21544

Octatetraene [Starting Material], (Hartree)						
Entry	R_1	R_2	Total Electronic Energy	Sum of Electronic and Zero-Point Energies	Sum of Electronic and Thermal Enthalpies	Gibbs Free Energy
1	CN	CH_3	-442.1581609	-441.975981	-441.963261	-442.014572
2		NH_2	-458.1997982	-458.029239	-458.01668	-458.067203
3		OH	-478.0614445	-477.903669	-477.891175	-477.942326
4		F	-502.076177	-501.930975	-501.918733	-501.969626
5		CHO	516.1466511	-515.983904	-515.970597	-516.023454
6		CN	-495.077481	-494.92552	-494.913223	-494.963119
7		NO_2	-607.2908224	-607.135013	-607.121122	-607.175965

Transition State, (Hartree)						
Entry	R_1	R_2	Total Electronic Energy	Sum of Electronic and Zero-Point Energies	Sum of Electronic and Thermal Enthalpies	Gibbs Free Energy
1	CN	CH_3	-442.1350792	-441.9528	-441.941398	-441.988549
2		NH_2	-458.1749472	-458.003584	-457.992577	-458.038521

3		OH	-478.0365116	-477.878137	-477.867254	-477.9131
4		F	-502.0546841	-501.90888	-501.89832	-501.943716
5		CHO	-516.1274613	-515.963574	-515.951862	-516.000008
6		CN	-495.0548077	-494.902111	-494.890547	-494.938364
7		NO_2	-607.2720824	-607.114739	-607.102543	-607.152447

Octatetraene [Starting Material], (Hartree)						
Entry	R_1	R_2	Total Electronic Energy	Sum of Electronic and Zero-Point Energies	Sum of Electronic and Thermal Enthalpies	Gibbs Free Energy
1	NO_2	CH_3	-554.3697695	-554.183733	-554.170206	-554.224666
2		NH_2	-570.4113745	-570.236065	-570.22288	-570.275476
3		OH	-590.2725277	-590.110371	-590.09715	-590.150325
4		F	-614.2863036	-614.137002	-614.125658	-614.174692
5		CHO	-628.358535	-628.191275	-628.177293	-628.232409
6		CN	-607.2885706	-607.132165	-607.118266	-607.173634
7		NO_2	-719.5018264	-719.34159	-719.326952	-719.384689

Transition State, (Hartree)						
Entry	R_1	R_2	Total Electronic Energy	Sum of Electronic and Zero-Point Energies	Sum of Electronic and Thermal Enthalpies	Gibbs Free Energy
1	NO_2	CH_3	-554.3530673	-554.166456	-554.154315	-554.204301
2		NH_2	-570.3921636	-570.216713	-570.204792	-570.254554
3		OH	-590.2539615	-590.091842	-590.08085	-590.127539
4		F	-614.2721489	-614.122113	-614.110799	-614.158999
5		CHO	-628.3454561	-628.177222	-628.164796	-628.215423
6		CN	-607.2721246	-607.115162	-607.102877	-607.15322
7		NO_2	-719.489381	-719.32812	-719.315091	-719.368169

4.15 3,6-substituted

Octatetraene [Starting Material], (Hartree)						
Entry	R_1	R_2	Total Electronic Energy	Sum of Electronic and Zero-Point Energies	Sum of Electronic and Thermal Enthalpies	Gibbs Free Energy
1	CH_3	CH_3	-389.2424341	-389.031313	-389.019627	-389.067162
2		NH_2	-405.2819463	-405.081908	-405.06957	-405.119198
3		OH	-425.1420007	-424.954589	-424.942462	-424.991752
4		F	-449.1595989	-448.984745	-448.97284	-449.022318
5		CHO	-463.2291932	-463.036729	-463.023599	-463.075907
6		CN	-442.1641087	-441.982913	-441.970721	-442.020438
7		NO_2	-441.943901	-441.943901	-554.176406	-554.231507

Transition State, (Hartree)						
Entry	R_1	R_2	Total Electronic Energy	Sum of Electronic and Zero-Point Energies	Sum of Electronic and Thermal Enthalpies	Gibbs Free Energy
1	CH_3	CH_3	-389.21259	-389.0013	-388.989768	-389.037771
2		NH_2	-405.2519219	-405.051443	-405.040286	-405.087436
3		OH	-425.1139195	-424.926349	-424.916287	-424.959953
4		F	-449.1328828	-448.957612	-448.947082	-448.947082
5		CHO	-463.2074393	-463.014399	-463.002799	-463.050325
6		CN	-442.020438	-441.95535	-441.943901	-441.943901
7		NO_2	-554.3557316	-554.169175	-554.15703	-554.206881

Octatetraene [Starting Material], (Hartree)						
Entry	R_1	R_2	Total Electronic Energy	Sum of Electronic and Zero-Point Energies	Sum of Electronic and Thermal Enthalpies	Gibbs Free Energy
1	NH2	NH_2	-421.3195316	-421.3195316	-421.118251	-421.16726
2		OH	-441.180455	-441.004039	-440.992162	-441.040803
3		F	-465.1986577	-465.034848	-465.023192	-465.0716

4		CHO	-479.2722957	-479.090384	-479.077677	-479.128469
5		CN	-458.2066601	-458.2066601	-458.023439	-458.074106
6		NO_2	-570.4203038	-570.245065	-570.231691	-570.285717

Transition State, (Hartree)						
Entry	R_1	R_2	Total Electronic Energy	Sum of Electronic and Zero-Point Energies	Sum of Electronic and Thermal Enthalpies	Gibbs Free Energy
1	NH2	NH_2	-421.2897991	-421.100097	-421.089423	-421.134077
2		OH	-441.1523724	-440.975592	-440.965084	-441.009482
3		F	-465.1720494	-465.007577	-464.997489	-464.997489
4		CHO	-479.2501352	-479.06791	-479.056697	-479.103098
5		CN	-458.1794341	-458.1794341	-457.996973	-458.043023
6		NO_2	-570.3984288	-570.222644	-570.2109	-570.259389

Octatetraene [Starting Material], (Hartree)						
Entry	R_1	R_2	Total Electronic Energy	Sum of Electronic and Zero-Point Energies	Sum of Electronic and Thermal Enthalpies	Gibbs Free Energy
1	OH	OH	-461.0423575	-460.878711	-460.866991	-460.915391
2		F	-485.0587299	-484.907577	-484.896124	-484.944225
3		CHO	-499.1304208	-498.961168	-498.948655	-498.999221
4		CN	-478.0648435	-477.906777	-477.894412	-477.94472
5		NO_2	-590.2775136	-590.114837	-590.101741	-590.154922

Transition State, (Hartree)						
Entry	R_1	R_2	Total Electronic Energy	Sum of Electronic and Zero-Point Energies	Sum of Electronic and Thermal Enthalpies	Gibbs Free Energy
1	OH	OH	-461.0148188	-460.850728	-460.84046	-460.884426
2		F	-485.0335727	-484.881979	-484.872033	-484.915576
3		CHO	-499.1099748	-498.940541	-498.929511	-498.975681
4		CN	-478.0395339	-477.88097	-477.87014	-477.915883
5		NO_2	-590.2580004	-590.095106	-590.083547	-590.131894

Octatetraene [Starting Material], (Hartree)						
Entry	R_1	R_2	Total Electronic Energy	Sum of Electronic and Zero-Point Energies	Sum of Electronic and Thermal Enthalpies	Gibbs Free Energy
1	F	F	-509.0758922	-508.937341	-508.926103	-508.974205
2		CHO	523.1454558	-522.989525	-522.977752	-523.027403
3		CN	-523.004682	-501.935359	-501.923933	-501.972004
4		NO_2	-614.292233	-614.14278	-614.131467	-614.180245

Transition State, (Hartree)						
Entry	R_1	R_2	Total Electronic Energy	Sum of Electronic and Zero-Point Energies	Sum of Electronic and Thermal Enthalpies	Gibbs Free Energy
1	F	F	-509.0520463	-508.912745	-508.903218	-508.946107
2		CHO	-523.1267016	-522.969711	-522.959024	-523.004682
3		CN	-502.0560783	-501.910013	-501.899502	-501.944764
4		NO_2	-614.2741091	-614.123623	-614.112393	-614.16035

Octatetraene [Starting Material], (Hartree)						
Entry	R_1	R_2	Total Electronic Energy	Sum of Electronic and Zero-Point Energies	Sum of Electronic and Thermal Enthalpies	Gibbs Free Energy
1	CHO	CHO	-537.2140259	-537.040079	-537.026441	-537.080621
2		CN	-516.148552	-515.985994	-515.973224	-516.025688
3		NO_2	-628.3588034	-628.191547	-628.178947	-628.231574

Transition State, (Hartree)						
Entry	R_1	R_2	Total Electronic Energy	Sum of Electronic and Zero-Point Energies	Sum of Electronic and Thermal Enthalpies	Gibbs Free Energy
1	CHO	CHO	-537.1995623	-537.024783	-537.012868	-537.06156

2		CN	-516.129173	-515.965281	-515.953581	-516.001721
3		NO_2	-628.3464943	-628.178324	-628.165881	-628.216734

Octatetraene [Starting Material], (Hartree)						
Entry	R_1	R_2	Total Electronic Energy	Sum of Electronic and Zero-Point Energies	Sum of Electronic and Thermal Enthalpies	Gibbs Free Energy
1	CN	CN	-495.0811098	-494.929653	-494.929653	-494.968263
2		NO_2	-607.2918608	-607.135481	-607.121521	-607.177169

Transition State, (Hartree)						
Entry	R_1	R_2	Total Electronic Energy	Sum of Electronic and Zero-Point Energies	Sum of Electronic and Thermal Enthalpies	Gibbs Free Energy
1	CN	CN	-495.0568517	-494.904078	-494.892496	-494.940363
2		NO_2	-607.2741732	-607.116968	-607.104698	-607.15492

Octatetraene [Starting Material], (Hartree)						
Entry	R_1	R_2	Total Electronic Energy	Sum of Electronic and Zero-Point Energies	Sum of Electronic and Thermal Enthalpies	Gibbs Free Energy
1	NO_2	NO_2	-719.502376	-719.341433	-719.326855	-719.384247

Transition State, (Hartree)						
Entry	R_1	R_2	Total Electronic Energy	Sum of Electronic and Zero-Point Energies	Sum of Electronic and Thermal Enthalpies	Gibbs Free Energy
1	NO_2	NO_2	-719.4911568	-719.329491	-719.316527	-719.369035

4.16 4,5-substituted

Octatetraene [Starting Material], (Hartree)						
Entry	R_1	R_2	Total Electronic Energy	Sum of Electronic and Zero-Point Energies	Sum of Electronic and Thermal Enthalpies	Gibbs Free Energy
1	CH_3	CH_3	-389.2399373	-389.029967	-389.01716	-389.067496
2		NH_2	-405.2787205	-405.079607	-405.066947	-405.117126
3		OH	-425.141553	-424.954569	-424.942405	-424.991267
4		F	-449.1517077	-448.976784	-448.96493	-449.013953
5		CHO	-463.2271263	-463.035302	-463.022071	-463.074303
6		CN	-442.1614981	-441.980078	-441.967278	-442.01796
7		NO_2	-554.3780859	-554.192788	-554.17914	-554.232457

Transition State, (Hartree)						
Entry	R_1	R_2	Total Electronic Energy	Sum of Electronic and Zero-Point Energies	Sum of Electronic and Thermal Enthalpies	Gibbs Free Energy
1	CH_3	CH_3	-389.2052727	-388.992666	-388.981884	-389.026939
2		NH_2	-405.2472557	-405.045793	-405.035256	-405.079627
3		OH	-425.1111933	-424.92272	-424.912305	-424.956529
4		F	-449.1312451	-448.955476	-448.945298	-448.989242
5		CHO	-463.1963652	-463.002455	-462.991207	-463.03779
6		CN	-442.1328457	-441.950321	-441.939165	-441.985434
7		NO_2	-554.3457745	-554.158504	-554.146733	-554.194815

Octatetraene [Starting Material], (Hartree)						
Entry	R_1	R_2	Total Electronic Energy	Sum of Electronic and Zero-Point Energies	Sum of Electronic and Thermal Enthalpies	Gibbs Free Energy
1	NH2	NH_2	-421.3187757	-421.130363	-421.118083	-421.167227
2		OH	-441.1774558	-441.001877	-440.989819	-441.038921
3		F	-465.192871	-465.029477	-465.017785	-465.066065
4		CHO	-479.2687517	-479.08748	-479.074661	-479.125555
5		CN	-458.1982587	-458.027995	-458.015288	-458.066232
6		NO_2	-570.4171248	-570.242511	-570.229171	-570.281645

Transition State, (Hartree)						
Entry	R_1	R_2	Total Electronic Energy	Sum of Electronic and Zero-Point Energies	Sum of Electronic and Thermal Enthalpies	Gibbs Free Energy
1	NH2	NH_2	-421.2913128	-421.100309	-421.090132	-421.133766
2		OH	-441.1496878	-440.972534	-440.962214	-441.006202
3		F	-465.1688496	-465.004499	-464.994422	-465.038095
4		CHO	-479.2416248	-479.058988	-479.047934	-479.09402
5		CN	-458.1734714	-458.002376	-457.991274	-458.037331
6		NO_2	-570.3843906	-570.209134	-570.197262	-570.245823

Octatetraene [Starting Material], (Hartree)						
Entry	R_1	R_2	Total Electronic Energy	Sum of Electronic and Zero-Point Energies	Sum of Electronic and Thermal Enthalpies	Gibbs Free Energy
1	OH	OH	-461.0364719	-461.0364719	-460.861115	-460.909432
2		F	-485.0526024	-484.901617	-484.890108	-484.938359
3		CHO	-499.1279077	-498.9592	-498.946624	-498.997275
4		CN	-478.0572434	-477.899501	-477.887074	-477.937479
5		NO_2	-590.2747836	-590.113189	-590.100012	-590.15209

Transition State, (Hartree)						
Entry	R_1	R_2	Total Electronic Energy	Sum of Electronic and Zero-Point Energies	Sum of Electronic and Thermal Enthalpies	Gibbs Free Energy
1	OH	OH	-461.0063536	-460.84226	-460.832001	-460.875972
2		F	-485.0263932	-484.874787	-484.864875	-484.908336
3		CHO	-499.1029856	-498.933181	-498.922245	-498.968262
4		CN	-477.937479	-477.874533	-477.863689	-477.909361
5		NO_2	-590.250183	-590.087131	-590.075767	-590.123287

Octatetraene [Starting Material], (Hartree)						
Entry	R_1	R_2	Total Electronic Energy	Sum of Electronic and Zero-Point Energies	Sum of Electronic and Thermal Enthalpies	Gibbs Free Energy
1	F	F	-509.0678593	-508.929716	-508.919322	-508.964322
2		CHO	-523.1424684	-522.986285	-522.973918	-523.024334
3		CN	-502.0719345	-501.926826	-501.914611	-501.965098
4		NO_2	-614.2855167	-614.136466	-614.123471	-614.176113

Transition State, (Hartree)						
Entry	R_1	R_2	Total Electronic Energy	Sum of Electronic and Zero-Point Energies	Sum of Electronic and Thermal Enthalpies	Gibbs Free Energy
1	F	F	-509.0448482	-508.90576	-508.896217	-508.939123
2		CHO	-523.1207892	-522.963601	-522.952919	-522.998613
3		CN	-502.0500514	-501.904283	-501.893723	-501.939061
4		NO_2	-614.2610273	-614.110395	-614.09926	-614.14629

Octatetraene [Starting Material], (Hartree)						
Entry	R_1	R_2	Total Electronic Energy	Sum of Electronic and Zero-Point Energies	Sum of Electronic and Thermal Enthalpies	Gibbs Free Energy
1	CHO	CHO	-537.2165937	-537.04265	-537.029211	-537.082036
2		CN	-516.1469532	-515.98406	-515.970764	-516.023375
3		NO_2	-628.3631508	-628.196541	-628.182376	-628.237768

Transition State, (Hartree)						
Entry	R_1	R_2	Total Electronic Energy	Sum of Electronic and Zero-Point Energies	Sum of Electronic and Thermal Enthalpies	Gibbs Free Energy
1	CHO	CHO	-537.1872469	-537.012052	-537.000414	-537.048407
2		CN	-516.1209691	-515.956943	-515.945276	-515.993335
3		NO_2	-628.3356146	-628.166912	-628.154769	-628.204223

Octatetraene [Starting Material], (Hartree)						
Entry	R_1	R_2	Total Electronic Energy	Sum of Electronic and Zero-Point Energies	Sum of Electronic and Thermal Enthalpies	Gibbs Free Energy
1	CN	CN	-495.0762411	-494.924658	-494.91144	-494.964011
2		NO_2	-607.2919237	-607.136031	-607.122142	-607.176773

Transition State, (Hartree)						
Entry	R_1	R_2	Total Electronic Energy	Sum of Electronic and Zero-Point Energies	Sum of Electronic and Thermal Enthalpies	Gibbs Free Energy
1	CN	CN	-495.0487601	-494.895916	-494.884412	-494.931966
2		NO_2	-607.2613104	-607.104013	-607.091874	-607.141298

Octatetraene [Starting Material], (Hartree)						
Entry	R_1	R_2	Total Electronic Energy	Sum of Electronic and Zero-Point Energies	Sum of Electronic and Thermal Enthalpies	Gibbs Free Energy
1	NO_2	NO_2	-719.5090484	-719.348708	-719.334053	-719.391457

Transition State, (Hartree)						
Entry	R_1	R_2	Total Electronic Energy	Sum of Electronic and Zero-Point Energies	Sum of Electronic and Thermal Enthalpies	Gibbs Free Energy
1	NO_2	NO_2	-719.4777951	-719.316	-719.303326	-719.354013

5. Cartesian coordinates of transition structure

5.1 Octatetraene

5.1.1 Helical-shaped transition structure

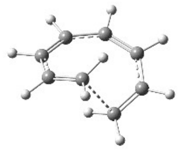

C	-0.83559000	-1.37784400	0.82000500
C	0.83559600	-1.37784300	-0.82000400
C	-1.78726700	-0.71310400	0.09239200
C	-1.72497800	0.65723400	-0.25826200
C	-0.69478900	1.57852500	-0.16776800
C	0.69478200	1.57852800	0.16776800
C	1.72497500	0.65724000	0.25826100
C	1.78727100	-0.71309700	-0.09239300
H	-0.13724200	-0.82116900	1.43098600
H	-0.96179200	-2.42976200	1.06058700
H	0.13724500	-0.82117300	-1.43098500
H	0.96180200	-2.42976100	-1.06058400
H	-2.63006900	-1.27760300	-0.30416600
H	-2.64951600	1.06723700	-0.66219700
H	-1.03654300	2.59237100	-0.37775800
H	1.03653300	2.59237400	0.37775800
H	2.64951100	1.06724700	0.66219800
H	2.63007600	-1.27759200	0.30416500

5.1.2 Boat-shaped transition structure

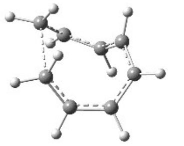

C	-0.14741900	1.64434700	-0.45688300
C	-1.73137400	-0.55895700	0.59864700
C	-0.49967300	-1.20241800	0.60895400
C	0.34554000	-1.37215400	-0.51860200
C	1.63329600	-0.85266700	-0.44329600
C	1.81231200	0.30048000	0.34541800
C	0.85915300	1.34378800	0.45689400
C	-2.04364100	0.29707800	-0.44910700
H	-0.03660300	1.24566600	-1.45945400
H	-0.66195900	2.59517100	-0.36550600
H	-2.26274100	-0.44831100	1.54152400
H	-0.03300300	-1.35534000	1.58135000
H	-0.02161100	-1.88367900	-1.41034100
H	2.47381400	-1.28437700	-0.98751400
H	2.78696200	0.49218600	0.79829400
H	0.99302600	2.03236700	1.29172500
H	-1.68440600	0.07503500	-1.44780400
H	-2.92263800	0.93429700	-0.39443000

5.2 Monosubstituted

5.2.1 1-substituted (outward)

1-CH_3-substituted

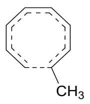

C	0.28515000	-1.20853900	1.11350100
C	-0.55571000	-1.84744900	0.23635600
C	-1.67035100	-1.23661200	-0.38094200
C	-2.06480300	0.09311200	-0.42137600
C	-1.53154400	1.35711500	-0.02940900
C	-0.28874000	1.89000400	0.28494600
C	1.01007300	1.34348600	0.20235600
C	1.33027600	0.16067100	-0.42404900
H	-0.05861900	-0.32246700	1.63080800
H	1.12004000	-1.73970500	1.56371800
H	-0.32439900	-2.86795500	-0.06601400
H	-2.34181700	-1.91670300	-0.90315100
H	-3.06649300	0.20994700	-0.83584700
H	-2.31599000	2.11399300	-0.00396100
H	-0.32919400	2.91485800	0.65130000
H	1.80271700	1.87950400	0.72621700
H	0.61881900	-0.22309300	-1.14739000
C	2.74454500	-0.33130500	-0.55268200
H	3.36339300	0.01459800	0.28118500
H	2.78567900	-1.42437400	-0.58531700
H	3.19249000	0.03849800	-1.48376300

1-NH_2-substituted

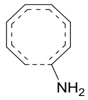

C	0.28233400	-1.13724500	1.10359500
C	-0.59184700	-1.81367400	0.26802700
C	-1.68634900	-1.21017300	-0.36339900
C	-2.06083700	0.13814500	-0.40431100
C	-1.48787300	1.37741100	-0.04345700
C	-0.21071000	1.86709800	0.26608800
C	1.05743800	1.29840600	0.16988800
C	1.34532700	0.05643700	-0.40705400
N	2.64817500	-0.41785300	-0.50466300
H	-0.08302700	-0.26671600	1.63360500
H	1.11648200	-1.66456500	1.55810400
H	-0.37268500	-2.84772200	0.00344900
H	-2.36452500	-1.88189800	-0.88729000
H	-3.06958700	0.27129200	-0.79682600
H	-2.23319700	2.17263800	-0.04592400
H	-0.21306600	2.89579500	0.62274400
H	1.88551100	1.84582700	0.62180200
H	0.65642200	-0.32994000	-1.14750400
H	2.87323900	-0.95254800	-1.33132400
H	3.38231300	0.22437200	-0.23445800

1-OH-substituted

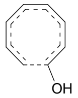

C	0.26951600	-1.20154400	1.08227300
C	-0.62940200	-1.81222800	0.23354600
C	-1.72002100	-1.15538700	-0.36364800
C	-2.05157600	0.19773600	-0.37756400
C	-1.43592000	1.41750700	0.00359200
C	-0.14492500	1.86633600	0.28098900
C	1.11158700	1.26165200	0.14582500
C	1.34989400	0.03368100	-0.45312800
H	-0.03797900	-0.31747500	1.62635300
H	1.08945800	-1.77502300	1.50710400
H	-0.44599400	-2.84204100	-0.06992300
H	-2.42853900	-1.79741400	-0.88430100
H	-3.05879500	0.37042200	-0.75797600
H	-2.16294900	2.22807400	0.04701200
H	-0.10647700	2.88950100	0.65052800
H	1.96254900	1.77547900	0.59594400
H	0.67199500	-0.35856300	-1.19809300
O	2.60043100	-0.47651200	-0.60105100
H	3.21835700	-0.00738000	-0.01954200

1-F-substituted

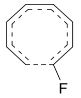

C	0.23963700	-1.26478700	1.08133300

C	-0.66985900	-1.81893700	0.21601800
C	-1.74096300	-1.11122500	-0.37408400
C	-2.02893800	0.24584700	-0.38358700
C	-1.38704800	1.45213700	0.02376800
C	-0.09478000	1.87536800	0.29814500
C	1.15713800	1.24187100	0.15639700
C	1.35096600	0.03621500	-0.46038900
H	-0.01674100	-0.36228100	1.62146600
H	1.04865600	-1.86494100	1.48926900
H	-0.52480900	-2.84835400	-0.10829100
H	-2.47085500	-1.72588300	-0.89840600
H	-3.02445300	0.45145300	-0.77769000
H	-2.10402200	2.27016100	0.08988800
H	-0.03418800	2.89406300	0.67576500
H	2.02686800	1.70389800	0.61818900
H	0.69830200	-0.37606900	-1.21698200
F	2.60492500	-0.45344200	-0.53764600

1-CHO-substituted

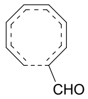

C	0.20481300	-1.02997900	1.25575300
C	-0.41881300	-1.86875800	0.36250300
C	-1.59120700	-1.54751800	-0.35030700
C	-2.27413000	-0.34621100	-0.51002200
C	-2.07880500	1.02626000	-0.19301200
C	-1.02683300	1.86008700	0.17669900
C	0.35454400	1.61651800	0.23706800
C	0.98398200	0.49480100	-0.26603700
H	-0.35013800	-0.21852000	1.70977200
H	1.09957000	-1.35913300	1.77773300
H	0.04890700	-2.82397900	0.13244500
H	-2.05550800	-2.38565000	-0.86763000
H	-3.24229800	-0.49322800	-0.98822000
H	-3.01405100	1.57778800	-0.29099200
H	-1.33453600	2.86537700	0.45821000
H	0.96224600	2.34501200	0.77625100
H	0.51141600	-0.09324800	-1.04343400
C	2.44559500	0.35497600	-0.13496900
H	2.92811000	1.07391000	0.56192100
O	3.10642500	-0.48117400	-0.71151400

1-CN-substituted

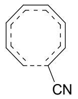

C	0.15496400	-1.17862800	1.13047100
C	-0.61079800	-1.89215500	0.24010400
C	-1.76119600	-1.38646800	-0.39878900
C	-2.28735800	-0.10056500	-0.45795100
C	-1.90110200	1.21335400	-0.07209300
C	-0.73412800	1.88892300	0.27190600
C	0.61344800	1.49376100	0.22791600
C	1.05900900	0.32568600	-0.36041400

H	-0.27306100	-0.32158100	1.63559400
H	1.03172200	-1.62784900	1.58863600
H	-0.28540000	-2.88914100	-0.05072400
H	-2.35260500	-2.12761300	-0.93406200
H	-3.28681700	-0.09248500	-0.89242600
H	-2.76230300	1.88130500	-0.07973400
H	-0.89812400	2.90580300	0.62252700
H	1.33870800	2.12328400	0.73894800
H	0.45445300	-0.15547600	-1.11844000
C	2.45905800	0.00727200	-0.38289300
N	3.58315100	-0.27476000	-0.38569600

1-NO_2-substituted

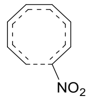

C	-0.18811400	-1.07741400	1.28288900
C	-0.93971000	-1.84892400	0.43720300
C	-2.07987000	-1.39000300	-0.26289500
C	-2.59380700	-0.11536900	-0.45032000
C	-2.20707800	1.23071400	-0.17755700
C	-1.04243400	1.93033400	0.10624800
C	0.30436200	1.51768800	0.12177700
C	0.72693900	0.30553500	-0.34921600
N	2.14078400	-0.03557800	-0.27819000
H	-0.60257300	-0.16837100	1.70119600
H	0.68498500	-1.49071000	1.78174800
H	-0.61444900	-2.86592200	0.22756100
H	-2.66804900	-2.17307800	-0.73872200
H	-3.58365500	-0.14121700	-0.90538500
H	-3.06742400	1.89606800	-0.24907800
H	-1.20022200	2.97808800	0.35164100
H	1.04320100	2.16412400	0.58908900
H	0.20863000	-0.27514400	-1.09609100
O	2.51266800	-0.95111500	-0.99610200
O	2.85387500	0.57734500	0.50067600

5.2.2 1-substituted (inward)

1-CH_3-substituted

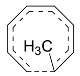

C	0.34023300	-1.23781800	-1.30301600
C	1.38404500	-1.23638300	-0.41187200
C	1.92321300	-0.08511000	0.20611100
C	1.43464400	1.20866900	0.30489000
C	0.20695900	1.87479200	0.02712300
C	-1.09756800	1.53205000	-0.30794400
C	-1.77411800	0.29679800	-0.42128300
C	-1.44308500	-0.91316800	0.15289300
H	0.07982800	-0.32522700	-1.82204700
H	0.02793900	-2.16579000	-1.77453100
H	1.81949200	-2.19132900	-0.11975100
H	2.89257900	-0.23208700	0.68145100

H	2.17494700	1.90300200	0.70196000
H	0.33473500	2.95577700	0.08791900
H	-1.69977100	2.38975100	-0.60675100
H	-2.62456600	0.29934500	-1.10435800
H	-2.05335000	-1.76678000	-0.14287800
C	-0.80212300	-1.04353700	1.50411000
H	-0.19544300	-0.17576300	1.76354300
H	-1.60659600	-1.12692100	2.24645000
H	-0.18298000	-1.94174200	1.58292200

1-NH_2-substituted

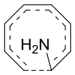

C	0.54330200	-1.15542700	-1.25535200
C	1.62928900	-0.82022300	-0.45240200
C	1.82175200	0.42347200	0.15012000
C	0.98296900	1.55642400	0.20237800
C	-0.37052300	1.82789400	-0.04613900
C	-1.52858300	1.07251100	-0.34170000
C	-1.77139400	-0.28960400	-0.30964400
C	-0.96423300	-1.26212900	0.32265100
N	-0.38752700	-0.99666400	1.54527300
H	0.05906100	-0.36725000	-1.81632900
H	0.51557300	-2.13976500	-1.71725900
H	2.34721500	-1.60093300	-0.19899600
H	2.79178600	0.56906400	0.62428500
H	1.51841100	2.44869500	0.52759000
H	-0.57851400	2.89701200	-0.01758400
H	-2.36064700	1.67668800	-0.70032100
H	-2.63305500	-0.66065300	-0.86097600
H	-1.22824100	-2.30799600	0.18873900
H	-0.12797600	-0.02924600	1.70535200
H	0.35359600	-1.62647800	1.82912600

1-OH-substituted

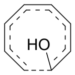

C	0.63765300	-1.18927200	-1.20856900
C	1.67317900	-0.74590700	-0.40211600
C	1.78247100	0.54324000	0.13377900
C	0.87262000	1.61135700	0.16295800
C	-0.50262000	1.78922900	-0.09760300
C	-1.59938600	0.95356500	-0.36990300
C	-1.73855500	-0.42783400	-0.28440000
C	-0.87574300	-1.29732500	0.39245500
H	0.09704200	-0.46878200	-1.80826800
H	0.67099800	-2.20083100	-1.60550200
H	2.41937100	-1.46750500	-0.07372100
H	2.73726900	0.76683600	0.60748200
H	1.34067700	2.54248700	0.48172700
H	-0.77825900	2.84314200	-0.09502900
H	-2.47763000	1.48251500	-0.73599800
H	-2.55263900	-0.89648600	-0.83330400
H	-1.06295200	-2.36615000	0.36986500

O	-0.22268800	-0.95806100	1.51968600
H	-0.11008300	0.00694700	1.57565900

1-F-substituted

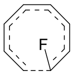

C	0.71156700	-1.18794900	-1.20251100
C	1.69581400	-0.73187800	-0.35955300
C	1.77878600	0.57758900	0.16129400
C	0.86168900	1.61839300	0.17584100
C	-0.51906500	1.78012100	-0.11374900
C	-1.61220200	0.95395000	-0.34977200
C	-1.76742000	-0.44009600	-0.25765300
C	-0.91906400	-1.30772700	0.38320000
H	0.13088600	-0.47956400	-1.77983800
H	0.75780500	-2.20143400	-1.59242000
H	2.44778200	-1.43757300	-0.00962600
H	2.73330400	0.82247100	0.62585600
H	1.30872500	2.56053400	0.49317700
H	-0.78976800	2.83499900	-0.15978200
H	-2.49960800	1.48165900	-0.69518500
H	-2.58563300	-0.90118200	-0.80707400
H	-1.07621000	-2.38041300	0.41853300
F	-0.20087900	-0.87487900	1.43153000

1-CHO-substituted

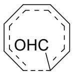

C	-0.38650400	0.70010400	1.65087700
C	-0.22280500	1.71775000	0.74538700
C	0.85679800	1.81529600	-0.16165600
C	1.77223300	0.86714100	-0.58576100
C	1.94308200	-0.54451300	-0.47168800
C	1.24788600	-1.66085200	-0.01632600
C	-0.05582400	-1.86864300	0.48673400
C	-1.16503100	-1.06088900	0.33871400
H	0.45658800	0.07113300	1.90752900
H	-1.21373400	0.72168000	2.35416200
H	-0.98505800	2.49183800	0.68464000
H	0.98975400	2.79672700	-0.61440000
H	2.57295600	1.30339500	-1.18161700
H	2.92291000	-0.83085600	-0.85408700
H	1.86485500	-2.55803200	0.00421600
H	-0.16943100	-2.75168700	1.11543700
H	-2.08330000	-1.31529000	0.86476000
C	-1.37769700	-0.26145700	-0.88713100
H	-0.51811700	-0.19667900	-1.57828300
O	-2.43878200	0.25551900	-1.16215700

1-CN-substituted

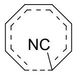

C	-0.64332100	-0.66594600	1.67762300
C	-1.69534400	-0.30955700	0.86738400
C	-1.71116800	0.81830200	0.02620300
C	-0.70054000	1.69766400	-0.35479000
C	0.70929800	1.76634200	-0.25744000
C	1.77682400	0.93731000	0.10025100
C	1.86010800	-0.43023500	0.38252900
C	0.90373700	-1.40403500	0.11747000
H	0.08926500	0.07928100	1.96006800
H	-0.72139300	-1.53616700	2.32346400
H	-2.55815200	-0.97001600	0.80951000
H	-2.68329800	1.05588600	-0.40241700
H	-1.09532400	2.57136400	-0.87189100
H	1.06256300	2.75760000	-0.54168000
H	2.72071800	1.46982100	0.20323600
H	2.73821000	-0.75603700	0.93772600
H	1.08497700	-2.41957800	0.45897500
C	0.07755300	-1.31827100	-1.04537400
N	-0.58577800	-1.25736900	-1.99430400

1-NO_2-substituted

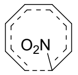

C	-0.22954800	0.74367200	1.76131400
C	-0.09159900	1.71170800	0.80570900
C	0.96388300	1.78249000	-0.13622800
C	1.92056200	0.85460200	-0.51500400
C	2.10205700	-0.54088100	-0.35604000
C	1.37020300	-1.63991300	0.08380900
C	0.05751200	-1.84302700	0.54825400
C	-1.08709700	-1.08050500	0.48761500
N	-1.43945400	-0.22650300	-0.62153800
H	0.60375200	0.09486900	2.00352300
H	-1.03907800	0.79949300	2.48334400
H	-0.85747400	2.48086800	0.73050000
H	1.06024900	2.74661000	-0.63377300
H	2.72312600	1.29519200	-1.10523400
H	3.10058800	-0.83863800	-0.67604000
H	1.97426100	-2.54343900	0.14750500
H	-0.06472400	-2.73665600	1.16055600
H	-1.98055300	-1.36743300	1.02773600
O	-2.55690300	0.27865500	-0.59127100
O	-0.62807300	-0.06293200	-1.51672000

5.2.3 2-substituted

2-CH_3-substituted

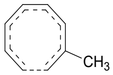

C	0.59483900	-1.56564900	-0.74602900
C	-0.77613100	-0.86250300	0.99322700
C	1.79568300	-1.29892100	-0.14142100
C	2.28780600	0.00002900	0.13088300
C	1.67310100	1.23987000	0.07733800
C	0.35877000	1.75947400	-0.14679600
C	-0.94521000	1.29730500	-0.12619900
C	-1.50198400	0.05242600	0.27555200
H	0.11273900	-0.80413200	-1.34607900
H	0.28614000	-2.59166900	-0.92722000
H	0.11758800	-0.54973300	1.51752500
H	-1.23140300	-1.79465800	1.31953000
H	2.39533500	-2.13286800	0.22029500
H	3.33311000	0.03885100	0.43440000
H	2.38697600	2.05305900	0.20942400
H	0.40556100	2.82567300	-0.37057200
H	-1.67463000	2.02471300	-0.48472500
C	-2.88539700	-0.28404300	-0.22881600
H	-3.29604100	-1.16068000	0.27901200
H	-3.57277300	0.55444900	-0.07130500
H	-2.87146200	-0.49093300	-1.30672100

2-NH_2-substituted

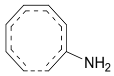

C	0.63486500	-1.56906100	-0.76896800
C	-0.82540200	-0.88568400	1.01083500
C	1.81524600	-1.27673700	-0.14948600
C	2.27460400	0.03720600	0.13916400
C	1.63748100	1.26043200	0.10024600
C	0.31016600	1.75615200	-0.13992400
C	-0.98143900	1.28258900	-0.12778300
C	-1.52371600	0.01398400	0.25502800
H	0.11074500	-0.81738100	-1.34559700
H	0.34244900	-2.60044900	-0.94643100
H	0.04906100	-0.55636900	1.55406100
H	-1.26879600	-1.83586100	1.29736800
H	2.43393900	-2.09617300	0.21279000
H	3.31831800	0.09611800	0.44609500
H	2.32799400	2.08844000	0.25953600
H	0.34080600	2.82378300	-0.35975900
H	-1.72391900	2.00474400	-0.46984800
N	-2.76384100	-0.33813700	-0.30391900
H	-3.36767700	-0.87806300	0.30365100
H	-3.26686100	0.42487900	-0.73910600

2-OH-substituted

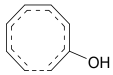

C	0.62634700	-1.55860000	-0.76892800
C	-0.82969800	-0.92027200	0.94388400
C	1.80729800	-1.26925000	-0.13681300
C	2.25434000	0.03854100	0.16971800

C	1.61532100	1.26583500	0.11545000
C	0.29800800	1.76019200	-0.14776100
C	-0.99565300	1.28077200	-0.15961300
C	-1.52032600	0.02233300	0.23204300
H	0.13262200	-0.80591400	-1.37080300
H	0.35067700	-2.59032700	-0.97058500
H	0.03754000	-0.61023300	1.50902300
H	-1.30047600	-1.85715500	1.23348500
H	2.42297400	-2.09121800	0.22452400
H	3.28933000	0.09906100	0.50411100
H	2.30570500	2.09242800	0.28220300
H	0.32912100	2.82764000	-0.36692500
H	-1.75187900	1.97317400	-0.52330400
O	-2.77342300	-0.22112800	-0.27299900
H	-3.16205000	-0.98573100	0.17439900

2-F-substituted

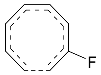

C	0.63874100	-1.55666600	-0.78133600
C	-0.83623100	-0.96881800	0.93284900
C	1.80518500	-1.24563400	-0.13138200
C	2.23225600	0.06614200	0.18241800
C	1.58381300	1.28966000	0.11879100
C	0.26641100	1.76892000	-0.15324400
C	-1.01830400	1.25998500	-0.17031200
C	-1.49646800	0.00016400	0.24103800
H	0.13967100	-0.81084600	-1.38695300
H	0.38508800	-2.59244900	-0.98913400
H	0.03351400	-0.68773500	1.50814800
H	-1.35005500	-1.88958400	1.18816900
H	2.42903300	-2.05853300	0.23652100
H	3.26187000	0.13633100	0.53070900
H	2.26654900	2.12206900	0.28756200
H	0.28013700	2.83614800	-0.37328700
H	-1.79276200	1.92859000	-0.53923600
F	-2.74505200	-0.29627800	-0.21060300

2-CHO-substituted

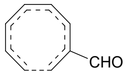

C	0.99943200	-1.52150300	-0.82912300
C	-0.34761700	-1.02222500	1.01336500
C	2.18505500	-1.18281000	-0.23595400
C	2.59009500	0.14020300	0.08203500
C	1.89151000	1.33204500	0.08807400
C	0.53559800	1.76100600	-0.09584300
C	-0.72152800	1.20104300	-0.02122000
C	-1.14348200	-0.09190600	0.39047700
H	0.42769300	-0.77258000	-1.36256900
H	0.77422100	-2.56004200	-1.05479600
H	0.55401900	-0.71784300	1.52651900
H	-0.76358600	-1.98913300	1.29251100
H	2.85831300	-1.98069200	0.07424500

H	3.63766300	0.23944700	0.36183700
H	2.54772000	2.19014700	0.23481000
H	0.49752500	2.82726900	-0.31858100
H	-1.53988100	1.85179400	-0.32423300
C	-2.53276700	-0.49963400	0.06261800
H	-2.78062600	-1.54986500	0.32507000
O	-3.36885500	0.22052300	-0.43517400

2-CN-substituted

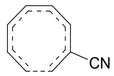

C	0.74325500	-1.59075000	-0.76914500
C	-0.55036700	-0.81010300	1.05456300
C	1.95086200	-1.37891200	-0.16673200
C	2.52256800	-0.10412600	0.09455400
C	1.98465300	1.16574700	0.04345600
C	0.69725000	1.75991500	-0.17814500
C	-0.62471300	1.38021100	-0.10793200
C	-1.21474400	0.16744100	0.35957000
H	0.27150300	-0.80161800	-1.34136100
H	0.37944100	-2.59979000	-0.94054600
H	0.38258400	-0.56641400	1.54375000
H	-1.07049700	-1.70550500	1.37968800
H	2.51140300	-2.24087400	0.19197300
H	3.57259000	-0.12800000	0.38137800
H	2.74395200	1.93778800	0.16518800
H	0.80127200	2.81220900	-0.44142300
H	-1.33528800	2.12776600	-0.45351100
C	-2.58590500	-0.07663700	-0.02326300
N	-3.68487400	-0.27318400	-0.33238500

2-NO_2-substituted

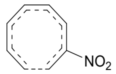

C	1.07140500	-1.54606400	-0.84717400
C	-0.21291500	-0.84552800	1.02483300
C	2.27262000	-1.36175000	-0.22640600
C	2.85083000	-0.10044700	0.09159900
C	2.32088800	1.17235500	0.09850500
C	1.03596600	1.78469500	-0.09644400
C	-0.28600600	1.40976400	-0.03167200
C	-0.84660400	0.17592000	0.38686100
H	0.60165100	-0.73081200	-1.38401100
H	0.70145400	-2.54528100	-1.05788400
H	0.71856800	-0.63082000	1.52912400
H	-0.74926000	-1.75142100	1.28115100
H	2.82757300	-2.23984600	0.10056600
H	3.90074700	-0.14352200	0.37642800
H	3.08430500	1.93375800	0.25605100
H	1.14566100	2.84605600	-0.31610700
H	-1.00838400	2.16300100	-0.32887700
N	-2.25335100	-0.06650200	-0.01797200
O	-2.91040000	0.88697700	-0.39755300

O	-2.67534500	-1.20813700	0.05614700

5.2.4 3-substituted

3-CH_3-substituted

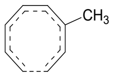

C	-1.53812100	-1.09695100	0.90077800
C	-0.16611100	-1.55664700	-0.89033800
C	-2.33642900	-0.17846500	0.26655400
C	-1.90476100	1.10015900	-0.15291100
C	-0.63677500	1.65771900	-0.22659300
C	0.72408800	1.25818200	-0.05063300
C	1.46871100	0.08437800	-0.01285600
C	1.03247000	-1.23263800	-0.30962300
H	-0.64816200	-0.76456700	1.41873300
H	-1.93894400	-2.06197600	1.19878600
H	-0.71380600	-0.80639400	-1.44565100
H	-0.40439300	-2.59366200	-1.10939700
H	-3.35191700	-0.46431800	-0.00402600
H	-2.70015600	1.77204200	-0.47188000
H	-0.68341700	2.72374700	-0.45144300
H	1.35562700	2.14143800	0.05107900
H	1.68843700	-2.04312800	0.01016700
C	2.93251400	0.20222200	0.37168200
H	3.20994700	1.23198500	0.60881900
H	3.14857300	-0.41265700	1.25358500
H	3.58469300	-0.15026400	-0.43513200

3-NH_2-substituted

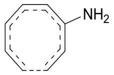

C	-1.55505800	-1.11385200	0.89066800
C	-0.13780600	-1.54123200	-0.88854900
C	-2.32416700	-0.17225500	0.25344300
C	-1.87718500	1.10479300	-0.15266400
C	-0.60286300	1.65784700	-0.21306500
C	0.75301400	1.26264000	-0.06171600
C	1.49271000	0.07864800	-0.00201000
C	1.05944100	-1.24000500	-0.29737200
H	-0.66200900	-0.81082900	1.42041000
H	-1.98488500	-2.07018800	1.17648800
H	-0.67252000	-0.78011900	-1.44224800
H	-0.39124800	-2.57371600	-1.10912400
H	-3.34274000	-0.43874700	-0.02724500
H	-2.66384300	1.78432900	-0.47561800
H	-0.64833900	2.72483000	-0.43384600
H	1.40821000	2.13350800	-0.01479500
N	2.85584300	0.22697700	0.31326900
H	3.06466100	0.91548200	1.02476100
H	3.35649700	-0.63765900	0.47084000
H	1.69679700	-2.05522600	0.04508000

3-OH-substituted

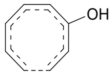

C	-1.52904600	-1.12101800	0.88441800
C	-0.08520600	-1.56402000	-0.87693400
C	-2.30487000	-0.20247400	0.22490500
C	-1.87704900	1.08410400	-0.17841100
C	-0.61687100	1.66378500	-0.20870700
C	0.74100000	1.28297100	-0.02092100
C	1.47924000	0.10905200	0.01598600
C	1.09699400	-1.22288600	-0.27731200
H	-0.64718600	-0.79583500	1.42061600
H	-1.94367800	-2.08300500	1.17318100
H	-0.63260100	-0.82553200	-1.44796400
H	-0.31048500	-2.60578100	-1.08306100
H	-3.31229600	-0.49172900	-0.07222400
H	-2.67283100	1.74613700	-0.51518700
H	-0.67323000	2.73087500	-0.42447300
H	1.39769000	2.14469600	0.08939800
H	1.75257000	-2.01207900	0.09247100
O	2.78675100	0.30898200	0.40398900
H	3.32288700	-0.45668900	0.15718700

3-F-substituted

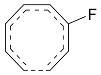

C	-1.49910800	-1.10977400	0.89007200
C	-0.06534000	-1.60326000	-0.86302800
C	-2.27965200	-0.20138800	0.22171100
C	-1.86165500	1.08687100	-0.18833200
C	-0.60819400	1.67659900	-0.21938400
C	0.74532400	1.28459700	-0.01364800
C	1.45129900	0.10084700	0.00654600
C	1.11766300	-1.23693200	-0.27747300
H	-0.62116000	-0.77606000	1.42753600
H	-1.90943700	-2.07105200	1.18663700
H	-0.62539100	-0.88378200	-1.44510400
H	-0.27434700	-2.65121700	-1.05432200
H	-3.28345500	-0.50049700	-0.07680400
H	-2.66279500	1.73983000	-0.53023400
H	-0.66234500	2.74250500	-0.43896300
H	1.41434100	2.13352200	0.11944200
F	2.75595400	0.25202500	0.37698400
H	1.81898400	-1.98683200	0.08017500

3-CHO-substituted

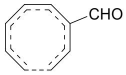

C	-1.66256900	-1.18555000	0.93792700

C	-0.35248700	-1.57254200	-0.94660200
C	-2.58200600	-0.36472200	0.33558300
C	-2.31674400	0.95814900	-0.08241400
C	-1.12282500	1.65398500	-0.20498000
C	0.26808500	1.36500700	-0.11137100
C	1.09915100	0.24931800	-0.12987200
C	0.81469100	-1.11430000	-0.40096300
H	-0.78956200	-0.75802100	1.41377900
H	-1.93609500	-2.19170400	1.24236600
H	-1.00840000	-0.89699700	-1.48017500
H	-0.48588200	-2.63310400	-1.14094400
H	-3.56820300	-0.76337400	0.10395600
H	-3.19646100	1.54103800	-0.35079600
H	-1.28452300	2.71447800	-0.39589100
H	0.84026400	2.29135700	-0.02304600
H	1.58366900	-1.82134200	-0.09521000
C	2.52076600	0.54584300	0.20769300
H	2.75778500	1.62137700	0.34570700
O	3.38638000	-0.28935300	0.34378000

3-CN-substituted

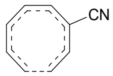

C	-1.75007800	-1.02614600	0.98912600
C	-0.48994600	-1.61576100	-0.90979400
C	-2.55200500	-0.11663900	0.35245500
C	-2.11644300	1.14625000	-0.11313400
C	-0.84492400	1.67809300	-0.25620300
C	0.50427900	1.23419100	-0.13896700
C	1.18216500	0.01807200	-0.12625600
C	0.72778100	-1.30930900	-0.37279400
H	-0.82099800	-0.70367100	1.44184900
H	-2.15098300	-1.97679200	1.32874700
H	-1.05369400	-0.86449600	-1.44657000
H	-0.76423600	-2.65185900	-1.08321000
H	-3.58314000	-0.38850200	0.13359600
H	-2.91387600	1.82769200	-0.40500700
H	-0.87112000	2.74168900	-0.49034500
H	1.18649600	2.08033000	-0.07223300
H	1.38715500	-2.11322400	-0.05300200
C	2.59340300	0.11909200	0.18066300
N	3.72271300	0.18311000	0.43080100

3-NO_2-substituted

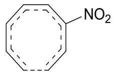

C	2.08947400	1.01515200	1.00027700
C	0.85817900	1.62684100	-0.90979900
C	2.89424000	0.10789600	0.36416100
C	2.45519100	-1.14779800	-0.11665300
C	1.18124300	-1.66668800	-0.28158500
C	-0.16463400	-1.21023100	-0.17855900
C	-0.81406900	0.00817900	-0.16682600

C	-0.36773000	1.33423800	-0.38463600
H	1.15604500	0.69246500	1.44377200
H	2.48895100	1.96325300	1.34845200
H	1.41850800	0.87767300	-1.45321100
H	1.14969500	2.66173800	-1.06010200
H	3.92864300	0.37650800	0.15761600
H	3.25041100	-1.83372100	-0.40412700
H	1.19992400	-2.72754700	-0.52679800
H	-0.86587300	-2.03848700	-0.13074200
N	-2.28214400	-0.08945600	0.12605600
H	-1.02042500	2.13386100	-0.05290300
O	-2.90846000	0.95418500	0.19983800
O	-2.78182000	-1.18982200	0.27983300

5.2.5 4-substituted

4-CH_3-substituted

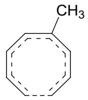

C	1.96565600	-0.06125400	0.90035300
C	1.34804400	1.38844800	-0.77077600
C	1.85365700	-1.21736100	0.16858900
C	0.63353700	-1.76059800	-0.29269600
C	-0.66057500	-1.26257400	-0.30786500
C	-1.35606300	-0.05078200	0.01626300
C	-0.95652500	1.27422000	0.14535200
C	0.26541800	1.93183200	-0.12561500
H	1.11453100	0.30009200	1.46241900
H	2.94176500	0.29319800	1.22023400
H	1.21759800	0.52768800	-1.41319700
H	2.23719900	1.98892500	-0.94278800
H	2.76272100	-1.71906700	-0.15959800
H	0.71678500	-2.76004200	-0.71772400
H	-1.37095500	-2.03519600	-0.60931000
H	-1.73606400	1.93825900	0.51575900
H	0.36499200	2.93408300	0.28946000
C	-2.84959200	-0.30029700	0.20358300
H	-3.03312100	-1.05677300	0.97403700
H	-3.38330200	0.60994000	0.48589900
H	-3.29349700	-0.67091000	-0.72831000

4-NH_2-substituted

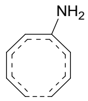

C	1.93182200	-0.02297700	0.90584600
C	1.30538700	1.41368900	-0.77845100
C	1.84985900	-1.19605100	0.19616900
C	0.64653200	-1.75397400	-0.29121900
C	-0.65234000	-1.27865100	-0.34725300
C	-1.36854200	-0.08546100	0.01194900
C	-0.99250800	1.24841500	0.16568900

C	0.20298400	1.92977000	-0.14118500
H	1.06695300	0.33416200	1.44990500
H	2.89765100	0.34902900	1.23707400
H	1.20781400	0.54599300	-1.41733000
H	2.17445000	2.04304300	-0.94793200
H	2.77024800	-1.68942700	-0.11086900
H	0.75520000	-2.74413000	-0.73188500
H	-1.35126100	-2.02846300	-0.71846600
H	-1.77551800	1.88391800	0.57879100
N	-2.75346500	-0.34753300	0.13618200
H	-2.98135600	-1.17208800	0.67862000
H	-3.30866200	0.44621100	0.42971400
H	0.27957100	2.93591700	0.26983900

4-OH-substituted

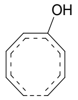

C	1.93515000	0.01575400	0.88744100
C	1.23675800	1.46417400	-0.78351100
C	1.87826700	-1.14494900	0.15912900
C	0.68103200	-1.75260100	-0.28424600
C	-0.63310000	-1.32281000	-0.29614000
C	-1.35912200	-0.13284900	0.02548500
C	-1.04634600	1.21264100	0.16563500
C	0.13127600	1.93618200	-0.12345600
H	1.06788600	0.33830000	1.44930100
H	2.89316100	0.42228300	1.19962800
H	1.15972100	0.59893300	-1.42855700
H	2.08861500	2.11731800	-0.94906000
H	2.80781700	-1.60092900	-0.17679500
H	0.80701900	-2.74902200	-0.70501700
H	-1.33783600	-2.10090500	-0.58422600
H	-1.85971200	1.81614700	0.57060500
H	0.17907200	2.93697000	0.30390800
O	-2.68917100	-0.46995900	0.20412600
H	-3.23586300	0.32732700	0.18518800

4-F-substituted

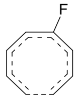

C	1.92403300	0.00365300	0.88741800
C	1.20569600	1.49280700	-0.77121200
C	1.87106300	-1.13938500	0.13477400
C	0.67448200	-1.76223400	-0.29398500
C	-0.63878100	-1.33342000	-0.27779300
C	-1.33066300	-0.12706100	0.02460900
C	-1.07680000	1.22069700	0.14954000
C	0.10259300	1.95759300	-0.10609200
H	1.05663900	0.31629200	1.45480000
H	2.88009900	0.41705300	1.19564100
H	1.12838800	0.63141800	-1.42169700
H	2.06363400	2.14089900	-0.92489300

H	2.80192600	-1.57986800	-0.21775400
H	0.79935700	-2.75972900	-0.71144500
H	-1.35895700	-2.10700700	-0.53921600
H	-1.93706300	1.78186200	0.50659800
H	0.14109400	2.95461400	0.32951200
F	-2.66276200	-0.40793700	0.20498900

4-CHO-substituted

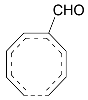

C	2.22444000	-0.38950700	0.90607100
C	1.87430900	1.16921600	-0.77742200
C	1.89509300	-1.48620900	0.15392600
C	0.58591200	-1.80967200	-0.28221700
C	-0.58876700	-1.08251100	-0.28576200
C	-1.00788000	0.25370400	0.01644700
C	-0.39026700	1.49454900	0.13856600
C	0.92922100	1.91904100	-0.12283300
H	1.46037300	0.12436400	1.47401500
H	3.25314000	-0.22020900	1.21194100
H	1.56671800	0.34726600	-1.40970100
H	2.86715900	1.57356600	-0.95384100
H	2.69549300	-2.13777700	-0.19380400
H	0.47850800	-2.81470100	-0.68705700
H	-1.45560800	-1.68628100	-0.55208000
H	-1.05149000	2.28404600	0.49807700
H	1.21857600	2.88633800	0.28382100
C	-2.49604100	0.35859300	0.19405800
H	-2.85317100	1.36193800	0.50431100
O	-3.29197800	-0.53522100	0.02241400

4-CN-substituted

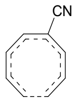

C	2.17941700	-0.16567500	0.95579300
C	1.65421700	1.35366700	-0.75734000
C	2.03390600	-1.28449300	0.18163400
C	0.80016400	-1.78718200	-0.30332700
C	-0.46795600	-1.24284400	-0.34398500
C	-1.08076500	0.02499000	-0.04287700
C	-0.65885300	1.34304000	0.10967300
C	0.59914900	1.94474100	-0.11345000
H	1.33286400	0.23722200	1.49599800
H	3.16356700	0.14499100	1.29488600
H	1.48890700	0.49869500	-1.39961500
H	2.58362400	1.89706500	-0.90159500
H	2.92921800	-1.81202800	-0.14338600
H	0.85292200	-2.79021400	-0.72276200
H	-1.21806400	-1.96742700	-0.65655500
H	-1.43670500	2.02001200	0.45622700
H	0.73699300	2.93530200	0.31546400

C	-2.52018900	-0.11280700	0.10205400
N	-3.66683800	-0.22917800	0.21889700

4-NO_2-substituted

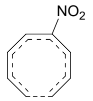

C	2.51497400	-0.08762300	0.97792500
C	2.01021200	1.27042600	-0.87588500
C	2.35939600	-1.26836000	0.30372700
C	1.12511500	-1.79819600	-0.14923800
C	-0.13801500	-1.25046100	-0.25815800
C	-0.71849500	0.03707100	-0.06590100
C	-0.30923400	1.35522000	-0.02244800
C	0.95413400	1.92347000	-0.29730600
H	1.67212600	0.37494000	1.47399000
H	3.50153200	0.23789600	1.29559300
H	1.84584800	0.35914200	-1.43532800
H	2.94251100	1.79519900	-1.06364800
H	3.25047300	-1.83348500	0.03554200
H	1.17232000	-2.83379900	-0.48038300
H	-0.89512200	-1.97861200	-0.53149200
H	-1.08643900	2.05167700	0.27457300
N	-2.22524100	-0.06972300	0.08025300
H	1.09476400	2.94863700	0.03883700
O	-2.79939700	0.79621800	0.71497100
O	-2.78933400	-1.01156800	-0.44569000

5.3 Disubstituted

5.3.1 1,2-substituted

1-CH_3-2-CH_3-substituted

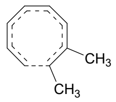

C	-0.47136900	-1.23737400	1.16758300
C	-1.59725600	-1.50375700	0.43592200
C	-2.40449900	-0.51055600	-0.17182700
C	-2.17853200	0.84217200	-0.35890200
C	-1.10443700	1.76767000	-0.16225200
C	0.27003300	1.73061900	0.00437600
C	1.21433100	0.67146800	-0.08207400
C	0.87473800	-0.54632700	-0.62450400
H	-0.32443800	-0.25116200	1.58962000
H	0.10733000	-2.04689100	1.60587000
H	-1.86682500	-2.54121100	0.24278300
H	-3.36383900	-0.86040400	-0.55013000
H	-3.06648400	1.35911400	-0.72396200
H	-1.47764500	2.79188300	-0.18452000
H	0.70505200	2.70333700	0.23515000
H	-0.02598700	-0.57820400	-1.22776600
C	1.84708700	-1.67732100	-0.81211000

H	2.49643400	-1.80750000	0.05934100
H	1.31879200	-2.61857500	-0.98795300
H	2.49330700	-1.49362100	-1.68170500
C	2.58144100	0.88430400	0.52653900
H	3.38250300	0.51051900	-0.11971400
H	2.76441500	1.94539400	0.71699700
H	2.66816500	0.36191900	1.48948200

1-CH_3-2-NH_2-substituted

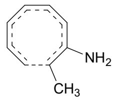

C	-0.46208700	-1.27389500	1.15381500
C	-1.57491500	-1.54395800	0.41093100
C	-2.39167800	-0.54975100	-0.19173800
C	-2.18545300	0.80350200	-0.36947700
C	-1.12476700	1.74723500	-0.15168100
C	0.24340600	1.73909200	0.01195200
C	1.20747600	0.68684300	-0.06433400
C	0.93440300	-0.52918000	-0.63875500
H	-0.31318500	-0.29010300	1.58104200
H	0.13422500	-2.07898400	1.57594300
H	-1.83023000	-2.58123700	0.19963200
H	-3.34545400	-0.90817300	-0.57699800
H	-3.07376500	1.31051000	-0.74641200
H	-1.51643100	2.76475600	-0.16534900
H	0.66831600	2.71917200	0.23338000
H	0.05549500	-0.57995800	-1.26961700
C	1.96063000	-1.61814300	-0.76085600
H	2.52775100	-1.72979000	0.17040900
H	1.48504700	-2.57632000	-0.98806000
H	2.67969700	-1.41243300	-1.56762600
N	2.43608400	0.88752000	0.59157500
H	3.24909400	0.54675300	0.09090000
H	2.58476300	1.83270300	0.92258800

1-CH_3-2-OH-substituted

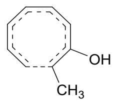

C	-0.42433000	-1.28752500	1.13231200
C	-1.53348700	-1.57731200	0.38176600
C	-2.35938200	-0.59859700	-0.22145000
C	-2.18672500	0.76714000	-0.37327500
C	-1.15858900	1.73231100	-0.12843600
C	0.21034700	1.75342400	0.05478200
C	1.18028200	0.72539000	-0.04772200
C	0.94853600	-0.51553700	-0.58848300
H	-0.32038200	-0.31069800	1.58810100
H	0.17275400	-2.08696500	1.56472600
H	-1.76671200	-2.61837900	0.16409100
H	-3.29649600	-0.97433200	-0.63011700
H	-3.08662300	1.25476500	-0.74811200

H	-1.57435900	2.74008000	-0.12981300
H	0.63367700	2.72129500	0.31578100
H	0.08054800	-0.59025300	-1.23125800
C	2.01744900	-1.56413500	-0.73350500
H	2.60597000	-1.67864900	0.18481600
H	1.56921000	-2.53678000	-0.95308100
H	2.70931600	-1.33329100	-1.55597000
O	2.37907900	1.04293900	0.54507000
H	3.07585500	0.45874100	0.21433800

1-CH_3-2-F-substituted

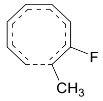

C	-0.37943300	-1.30375500	1.12454500
C	-1.47971900	-1.60925100	0.36324100
C	-2.32935300	-0.65007700	-0.23271300
C	-2.20073800	0.72465200	-0.37059300
C	-1.20196800	1.71291700	-0.12377200
C	0.16850900	1.75138600	0.06869600
C	1.13219000	0.73542800	-0.05806700
C	0.97806500	-0.51894900	-0.58194000
H	-0.30611900	-0.33225400	1.59708400
H	0.22988700	-2.09518800	1.55389800
H	-1.68592000	-2.65363400	0.13440600
H	-3.25282100	-1.05004300	-0.64899000
H	-3.11748200	1.18706000	-0.73599900
H	-1.63609500	2.71251600	-0.12261400
H	0.58321600	2.71810700	0.34610200
H	0.11988100	-0.64202400	-1.23092800
C	2.11266800	-1.49328300	-0.71396000
H	2.78253300	-1.44345500	0.14775400
H	1.73210500	-2.51384700	-0.80833000
H	2.70590100	-1.27379900	-1.61026000
F	2.33817600	1.03246100	0.50280700

1-CH_3-2-CHO-substituted

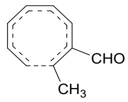

C	-0.71969000	-1.22234000	1.19651100
C	-1.84916500	-1.48086800	0.47438400
C	-2.67296400	-0.48590200	-0.12071500
C	-2.44918500	0.86096900	-0.31573300
C	-1.36245700	1.78202400	-0.13427000
C	0.01136800	1.72250200	-0.03647100
C	0.94004800	0.64716000	-0.16659800
C	0.59085700	-0.59022200	-0.67348400
H	-0.54400300	-0.22984500	1.59231000
H	-0.13665900	-2.03265600	1.62538400
H	-2.12482700	-2.51763400	0.28592100
H	-3.64052300	-0.83796900	-0.47404300
H	-3.34025200	1.38292600	-0.66528000

H	-1.72891900	2.80816300	-0.11759500
H	0.47579100	2.68707400	0.17220900
H	-0.33900800	-0.61286300	-1.23174000
C	1.51374900	-1.75489800	-0.88605400
H	2.09596400	-1.98866000	0.00600100
H	0.94099800	-2.63320100	-1.19501600
H	2.24000700	-1.52023000	-1.67537900
C	2.31110100	0.93291600	0.31220600
H	2.48614800	1.99076800	0.60204300
O	3.21666500	0.12951100	0.39706600

1-CH_3-2-CN-substituted

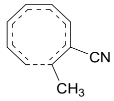

C	0.72212200	1.18309600	1.20150800
C	1.90174900	1.35346400	0.52751100
C	2.64499500	0.30235100	-0.06704600
C	2.30854600	-1.01816800	-0.30057200
C	1.14429800	-1.84335500	-0.17664700
C	-0.22299500	-1.67261500	-0.09194300
C	-1.02275200	-0.50236300	-0.22252000
C	-0.56006000	0.71591600	-0.67938300
H	0.45823200	0.20651800	1.58819900
H	0.20586000	2.03428500	1.63766100
H	2.27967100	2.36368300	0.37699800
H	3.65008100	0.57132200	-0.38721100
H	3.16240100	-1.60783400	-0.63385600
H	1.41747400	-2.89805000	-0.18813100
H	-0.78422800	-2.58577900	0.09405200
H	0.36762900	0.68118300	-1.23917100
C	-1.43161100	1.91924300	-0.88230900
H	-0.83626500	2.83586100	-0.86632200
H	-1.92389600	1.86060700	-1.86162600
H	-2.21352700	1.98841700	-0.12106800
C	-2.38550200	-0.58966900	0.24310200
N	-3.48231200	-0.64823000	0.61289500

1-CH_3-2-NO_2-substituted

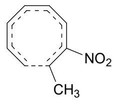

C	1.11109600	1.15930500	1.19986500
C	2.30648400	1.20817000	0.53679700
C	2.94661500	0.08456600	-0.04886400
C	2.47843100	-1.19320900	-0.28631300
C	1.23438100	-1.89775500	-0.17163500
C	-0.11084500	-1.59742700	-0.11663200
C	-0.77541900	-0.35402700	-0.25974800
C	-0.18883700	0.81187100	-0.69580200
H	0.74480800	0.21449000	1.58295900
H	0.67434000	2.05944400	1.62327300
H	2.78447600	2.17519600	0.38741500

H	3.97686800	0.24925900	-0.35930300
H	3.26930500	-1.86724800	-0.61511400
H	1.40278200	-2.97393700	-0.17470800
H	-0.77117400	-2.44350500	0.04473000
H	0.74076500	0.61892000	-1.21974000
C	-0.82174700	2.14859600	-0.96797800
H	-1.63171800	2.04606400	-1.70037700
H	-1.26013600	2.58722200	-0.07222400
H	-0.06968800	2.82762800	-1.37657900
N	-2.18202600	-0.34208100	0.17722200
O	-2.66768000	0.73336800	0.49357400
O	-2.79074600	-1.39830700	0.19404900

1- NH_2-2-CH_3-substituted

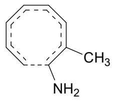

C	-0.46346000	-1.18413600	1.15433300
C	-1.61282900	-1.46497700	0.44189000
C	-2.39956900	-0.48495900	-0.18583000
C	-2.15523000	0.87955800	-0.35098500
C	-1.07275000	1.77038900	-0.14483700
C	0.31095700	1.68875300	0.04402200
C	1.22321100	0.63070700	-0.06348800
C	0.84313900	-0.60735900	-0.58899700
H	-0.35042100	-0.20458000	1.60131000
H	0.09934200	-1.99200100	1.61732500
H	-1.89085400	-2.50558800	0.27635500
H	-3.35173000	-0.82611000	-0.58850200
H	-3.03752000	1.41376200	-0.70541900
H	-1.40823000	2.80669300	-0.18138500
H	0.76750800	2.64386600	0.30382800
H	-0.03811600	-0.64307500	-1.21776700
N	1.77658800	-1.59857300	-0.86237100
H	2.53350200	-1.68974300	-0.19517800
H	1.38111000	-2.49898400	-1.10100000
C	2.63372300	0.79837000	0.44162300
H	3.37080200	0.50037000	-0.31495000
H	2.82884700	1.83797800	0.71616300
H	2.81649900	0.18934500	1.33943700

1- NH_2-2-NH_2-substituted

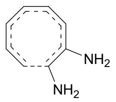

C	-0.48904700	-1.22623800	1.15276100
C	-1.60219000	-1.50274900	0.39937900
C	-2.39519800	-0.51352300	-0.22402600
C	-2.16708700	0.84866500	-0.36400400
C	-1.09370500	1.75243800	-0.11720100
C	0.28413100	1.69277200	0.05645200
C	1.21622500	0.64130800	-0.05470800
C	0.91637100	-0.59643400	-0.60771300

H	-0.36745700	-0.24462600	1.59253400
H	0.08913000	-2.03098900	1.60075600
H	-1.86027300	-2.54222300	0.19919200
H	-3.33894500	-0.85990000	-0.64247700
H	-3.04458900	1.38037800	-0.73311700
H	-1.44567700	2.78378400	-0.12683700
H	0.73708500	2.65311100	0.30607900
H	0.05777100	-0.68111200	-1.26088600
N	1.92133700	-1.53913400	-0.78950900
H	2.58206900	-1.57032500	-0.01778700
H	1.58699200	-2.46315500	-1.03341900
N	2.51944700	0.77815900	0.48941200
H	3.24911600	0.67198400	-0.21385400
H	2.65227900	1.65247000	0.98485100

1- NH_2-2-OH-substituted

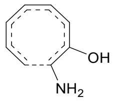

C	-0.47521600	-1.27481500	1.12269700
C	-1.57634300	-1.54667300	0.34956400
C	-2.37795100	-0.55695900	-0.26066500
C	-2.18081900	0.81319000	-0.36502000
C	-1.13443500	1.73679200	-0.08118200
C	0.24160500	1.70626400	0.10276000
C	1.19099600	0.68177500	-0.04135700
C	0.96131300	-0.57473600	-0.57637700
H	-0.37115900	-0.30255500	1.58779600
H	0.09718300	-2.08650300	1.56609000
H	-1.81908100	-2.58471300	0.12495000
H	-3.30871700	-0.91284100	-0.69971200
H	-3.06701200	1.33183800	-0.73161400
H	-1.51306700	2.75831800	-0.06172700
H	0.69239000	2.65483400	0.38791200
H	0.13095300	-0.70455200	-1.25669600
N	2.03748400	-1.45930200	-0.72952100
H	1.76842900	-2.35870700	-1.10952000
H	2.57444700	-1.59033900	0.12315500
O	2.44404900	0.94067800	0.47618600
H	3.10596100	0.57587900	-0.13598400

1- NH_2-2-F-substituted

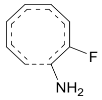

C	-0.37693200	-1.26213800	1.12178300
C	-1.49455900	-1.57378500	0.36986800
C	-2.32482300	-0.62509900	-0.24599800
C	-2.18698900	0.76159300	-0.35997700
C	-1.17945300	1.71937900	-0.10782400
C	0.20299900	1.71162300	0.09572700
C	1.13497000	0.69622400	-0.05234300
C	0.96358800	-0.58490600	-0.56539100

H	-0.32402200	-0.29843000	1.61263800
H	0.21215400	-2.05836200	1.57137200
H	-1.70878300	-2.62156400	0.16161900
H	-3.23963900	-1.01911700	-0.68563600
H	-3.10204800	1.23744800	-0.71299000
H	-1.57757200	2.73305800	-0.11754000
H	0.64346100	2.66037800	0.39481000
H	0.12279900	-0.73581700	-1.22930800
F	2.38482700	0.94713800	0.43999500
N	2.05754300	-1.40417600	-0.77404100
H	2.82857600	-1.27130000	-0.13106700
H	1.84602900	-2.37865200	-0.94064000

1- NH_2-2-CHO-substituted

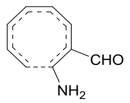

C	0.94180400	1.03019700	1.18911500
C	2.17818600	1.01644100	0.56343500
C	2.70413400	-0.10801300	-0.08887700
C	2.10174100	-1.34104100	-0.34521000
C	0.80493700	-1.90378900	-0.23168200
C	-0.50615200	-1.44299600	-0.10903600
C	-1.07191300	-0.16293500	-0.19982100
C	-0.31442300	0.97068700	-0.57617800
H	0.55261500	0.09730900	1.57852100
H	0.60680000	1.93358300	1.69418800
H	2.74529400	1.94409700	0.49038600
H	3.73417500	-0.02544400	-0.42977500
H	2.81961200	-2.08281600	-0.69635100
H	0.84326100	-2.99002800	-0.30475500
H	-1.24620200	-2.22266100	0.06090000
H	0.55068700	0.76826300	-1.19731900
N	-0.87251000	2.21810900	-0.81672100
H	-1.56624100	2.54735100	-0.15799100
H	-0.19852800	2.94110300	-1.03262200
C	-2.50530900	-0.03250600	0.11897300
H	-2.94877500	0.97395200	-0.01309500
O	-3.22289400	-0.94596800	0.47508100

1-NH_2-2-CN-substituted

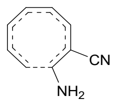

C	0.65381300	1.14756500	1.14103200
C	1.87136100	1.36232000	0.51089000
C	2.62867500	0.34818400	-0.08812500
C	2.31560900	-1.00090700	-0.28401800
C	1.17565500	-1.82649600	-0.14997400
C	-0.20618200	-1.65200700	-0.03614800
C	-1.00794700	-0.51547700	-0.18880700
C	-0.53724000	0.74737200	-0.62204100
H	0.47040500	0.17716900	1.58609700

H	0.13046600	1.98402700	1.59836300
H	2.22836000	2.38436200	0.38798900
H	3.61843200	0.63644400	-0.43645200
H	3.18464100	-1.58196000	-0.59300000
H	1.44381300	-2.88156700	-0.17797700
H	-0.75792100	-2.56264100	0.18602200
H	0.35440400	0.73429600	-1.23690800
N	-1.41464200	1.76893900	-0.90619900
H	-2.26755500	1.81321300	-0.35894300
H	-1.00747000	2.67396500	-1.09647600
C	-2.38651300	-0.58290500	0.20346400
N	-3.50549700	-0.56082600	0.50957600

1-NH_2-2-NO_2-substituted

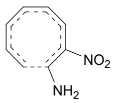

C	0.98917700	1.10102600	1.13644300
C	2.25431900	1.20365700	0.56662000
C	2.92764200	0.12801500	-0.02139300
C	2.47774500	-1.17519800	-0.26472700
C	1.25316800	-1.87814000	-0.18959900
C	-0.10726000	-1.56750700	-0.10639200
C	-0.77019500	-0.35143800	-0.24697900
C	-0.13014400	0.85672700	-0.63528900
H	0.70971900	0.14984000	1.57482000
H	0.53174700	1.97775900	1.58857000
H	2.71799800	2.18587800	0.48106800
H	3.95538100	0.31497200	-0.32552400
H	3.29017500	-1.83874800	-0.56122500
H	1.41141700	-2.95409000	-0.23977000
H	-0.76405400	-2.41217700	0.07761600
H	0.76222800	0.65963500	-1.22016800
N	-0.77591200	2.00877900	-1.00516400
H	-1.59884900	2.26497000	-0.47622800
H	-0.17993700	2.77590600	-1.28230400
N	-2.16717600	-0.32571800	0.14829500
O	-2.66203700	0.77131300	0.40731300
O	-2.78807800	-1.37234100	0.21132700

1-OH-2-CH_3-substituted

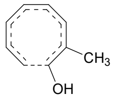

C	-0.48369900	-1.21994300	1.12952800
C	-1.62976200	-1.46241700	0.40770000
C	-2.41069600	-0.44972400	-0.18391400
C	-2.14488200	0.90759100	-0.32772700
C	-1.04141200	1.77871100	-0.11013600
C	0.33717200	1.67319600	0.05424600
C	1.23785000	0.59844900	-0.08013100
C	0.83567400	-0.61505300	-0.61917300
H	-0.34614900	-0.24984100	1.59133500

H	0.07980600	-2.04825400	1.55197200
H	-1.91305300	-2.49324200	0.20046900
H	-3.37279300	-0.76689100	-0.58257300
H	-3.01763400	1.46485000	-0.66933800
H	-1.36306800	2.81997800	-0.11343800
H	0.81465800	2.61657500	0.31832600
H	-0.03453400	-0.67055100	-1.25870200
O	1.68900800	-1.64605200	-0.84961600
H	2.50496200	-1.52570800	-0.34086400
C	2.64602500	0.73556500	0.44873500
H	2.80372700	0.13390400	1.35571700
H	3.40091500	0.43588100	-0.29050900
H	2.85348000	1.77346500	0.71976600

1-OH-2-NH_2-substituted

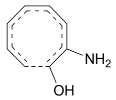

C	-0.47405100	-1.25552000	1.09641400
C	-1.60249900	-1.50074600	0.35091300
C	-2.40240800	-0.48944200	-0.21941100
C	-2.16885300	0.87630000	-0.32052200
C	-1.08146300	1.76025400	-0.07658800
C	0.29935000	1.67358200	0.07110700
C	1.21066400	0.61324900	-0.07864300
C	0.89676500	-0.63032500	-0.61892100
H	-0.35817100	-0.29315400	1.57948800
H	0.10712000	-2.08014200	1.50048500
H	-1.85921200	-2.53131400	0.11007900
H	-3.35423700	-0.81768200	-0.63380200
H	-3.05119400	1.42587200	-0.64930400
H	-1.41857000	2.79620100	-0.05343500
H	0.77505800	2.61826900	0.33627900
H	0.06469500	-0.75782700	-1.29664200
O	1.84597000	-1.58099700	-0.74267700
H	2.58260800	-1.30363400	-0.16156400
N	2.55173400	0.70666600	0.42866800
H	3.20112300	1.06495900	-0.26978200
H	2.61585100	1.29565200	1.25285000

1-OH-2-OH-substituted

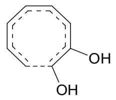

C	-0.45237200	-1.28164100	1.10715000
C	-1.56802200	-1.53690500	0.34936400
C	-2.38097900	-0.53534200	-0.22362200
C	-2.17716300	0.83404700	-0.32787300
C	-1.10846600	1.74294100	-0.08881600
C	0.27015500	1.68351800	0.06434400
C	1.18963600	0.63071400	-0.07088900
C	0.95010700	-0.61376200	-0.62275000
H	-0.34777400	-0.31844500	1.59157100

H	0.14083500	-2.09912200	1.50816200
H	-1.80774900	-2.56959900	0.10088100
H	-3.32562700	-0.88123300	-0.64047300
H	-3.06906400	1.36326400	-0.66321100
H	-1.46440100	2.77248100	-0.07809500
H	0.73886000	2.63802000	0.30646800
H	0.11868800	-0.77676200	-1.29213900
O	1.94737500	-1.51518800	-0.75581800
H	2.72251100	-1.18141200	-0.27079600
O	2.48459100	0.78065400	0.40112000
H	2.50061900	1.38765500	1.15377200

1-OH-2-F-substituted

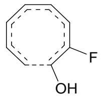

C	-0.41295000	-1.30624500	1.08796600
C	-1.52663400	-1.56596500	0.32182900
C	-2.34851000	-0.57676800	-0.25183000
C	-2.18237800	0.80265900	-0.33304700
C	-1.14645700	1.73374700	-0.06345200
C	0.23254200	1.69412100	0.11015700
C	1.14872600	0.66109400	-0.07093200
C	0.97109500	-0.60708300	-0.59139000
H	-0.32739700	-0.35273300	1.59500100
H	0.17467900	-2.12654600	1.49185900
H	-1.75322300	-2.60006400	0.06659300
H	-3.27715600	-0.94009300	-0.68906700
H	-3.08710100	1.31001600	-0.66775600
H	-1.52577400	2.75401800	-0.02934900
H	0.70012700	2.62884300	0.41209900
H	0.15850200	-0.80197000	-1.27455600
F	2.40819500	0.88087700	0.42589800
O	2.00958200	-1.46285600	-0.71979100
H	2.77432500	-1.10987500	-0.23538300

1-OH-2-CHO-substituted

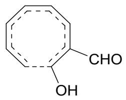

C	0.95533400	1.06885800	1.18305500
C	2.17359200	1.04377200	0.53768900
C	2.70421900	-0.10207700	-0.08657000
C	2.10594400	-1.33252300	-0.32745400
C	0.80336900	-1.89766000	-0.20093700
C	-0.50232900	-1.43968400	-0.10188300
C	-1.07189900	-0.15331000	-0.21655700
C	-0.31491500	0.95955300	-0.61381700
H	0.55480300	0.14346300	1.57959500
H	0.61020500	1.98856500	1.64869500
H	2.72628200	1.97461700	0.41990100
H	3.73536600	-0.02354900	-0.42507800
H	2.82100900	-2.07865700	-0.67458300

H	0.84700200	-2.98547300	-0.23900800
H	-1.24532000	-2.21466700	0.07525200
H	0.55865000	0.78959300	-1.22985800
O	-0.80491300	2.20496500	-0.81875200
H	-1.62758100	2.34921900	-0.32918700
C	-2.49604600	-0.01424700	0.12910600
H	-2.94129300	0.99868100	0.02619100
O	-3.21793000	-0.92220000	0.48528900

1-OH-2-CN-substituted

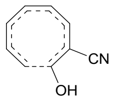

C	-0.67324700	-1.19804800	1.12722200
C	-1.86543100	-1.39000800	0.46246400
C	-2.63026300	-0.35044500	-0.10130500
C	-2.32157900	0.99487100	-0.26529700
C	-1.17722600	1.82397000	-0.09938400
C	0.19723400	1.65434400	-0.00891300
C	1.00903800	0.51725600	-0.19676900
C	0.55517200	-0.72546600	-0.66029900
H	-0.47491900	-0.23479400	1.58213400
H	-0.13614300	-2.04959400	1.53647100
H	-2.20605800	-2.40817100	0.28051100
H	-3.61977600	-0.63279300	-0.45530700
H	-3.18660400	1.58304600	-0.57129000
H	-1.45334600	2.87720800	-0.07978600
H	0.75172900	2.56018500	0.22467600
H	-0.32965900	-0.75780700	-1.28068100
O	1.37517400	-1.76764400	-0.88530400
H	2.24475300	-1.62157900	-0.47046000
C	2.38532700	0.56293500	0.20600300
N	3.50492600	0.49842900	0.50483200

1-OH-2-NO_2-substituted

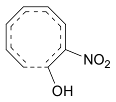

C	1.01299000	1.13328900	1.13979200
C	2.24933800	1.23741100	0.53110500
C	2.93296100	0.14961400	-0.04173700
C	2.49332400	-1.15027700	-0.26858800
C	1.26645300	-1.86458400	-0.16676600
C	-0.08761600	-1.56621300	-0.09890500
C	-0.76663500	-0.34906400	-0.25598500
C	-0.14198100	0.85213200	-0.66147200
H	0.72864600	0.18086900	1.57278300
H	0.54185000	2.01655400	1.56252800
H	2.68891900	2.22467800	0.39893700
H	3.95715800	0.34300300	-0.35344700
H	3.30321200	-1.81253600	-0.57423500
H	1.43669500	-2.93970200	-0.18384400
H	-0.74051100	-2.41364000	0.08562100

H	0.75205600	0.71045200	-1.25709500
O	-0.75675900	2.00492100	-0.93020600
H	-1.64465200	1.98008600	-0.51375400
N	-2.15896400	-0.32598900	0.13638700
O	-2.70049300	0.78266500	0.26276600
O	-2.75070200	-1.37029600	0.32283200

1-F-2-CH_3-substituted

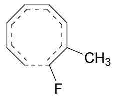

C	-0.51367900	-1.26092700	1.11904600
C	-1.65964800	-1.45267000	0.39205100
C	-2.41470000	-0.40276000	-0.18140100
C	-2.11707200	0.94338500	-0.31881000
C	-0.99420100	1.79780700	-0.08908900
C	0.37757800	1.67400100	0.05778800
C	1.26529100	0.57781500	-0.08809700
C	0.83192100	-0.59731600	-0.63977200
H	-0.32788700	-0.29784100	1.57919800
H	0.03732000	-2.10900100	1.51736500
H	-1.97680200	-2.46949000	0.16624900
H	-3.38921400	-0.68881000	-0.57381600
H	-2.97551600	1.52154200	-0.66113600
H	-1.30597500	2.84194600	-0.06655900
H	0.87579700	2.60601800	0.32291900
H	-0.02355000	-0.67937500	-1.29629600
F	1.71152300	-1.60843200	-0.80356500
C	2.66162300	0.66666800	0.47448900
H	2.69957600	0.25621100	1.49168400
H	3.37786400	0.10796800	-0.13272400
H	2.98200600	1.71070400	0.52796900

1-F-2-NH_2-substituted

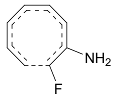

C	-0.53219200	-1.29898500	1.12911400
C	-1.65786900	-1.48174300	0.37915500
C	-2.40796000	-0.42472700	-0.20322900
C	-2.11708300	0.91711900	-0.34536400
C	-1.00075500	1.78448300	-0.09247700
C	0.36283100	1.68152200	0.06641200
C	1.26640400	0.58396300	-0.05624900
C	0.89417400	-0.58761000	-0.65054000
H	-0.32594800	-0.33684800	1.58233200
H	0.02194900	-2.14831100	1.52008500
H	-1.97228200	-2.49578200	0.13823700
H	-3.37864700	-0.71372500	-0.60401300
H	-2.96847200	1.48945800	-0.71325700
H	-1.32493000	2.82484400	-0.06857600
H	0.85429800	2.61924000	0.32649200
H	0.05799500	-0.69027000	-1.32574000

F	1.83579400	-1.55194700	-0.79029700
N	2.51550200	0.66232700	0.56941000
H	3.25006200	0.13581000	0.11032400
H	2.81001100	1.60268200	0.79999200

1-F-OH-2-OH-substituted

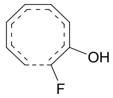

C	-0.51639800	-1.28949200	1.12407100
C	-1.63659900	-1.48349100	0.36505200
C	-2.39209700	-0.43148100	-0.21126600
C	-2.10831400	0.91612100	-0.33758100
C	-0.99365100	1.78088000	-0.09226700
C	0.37417500	1.67561500	0.06264400
C	1.26930500	0.58143400	-0.05979300
C	0.91906400	-0.60997800	-0.63178300
H	-0.34132500	-0.32645600	1.58808500
H	0.04367100	-2.13204000	1.52070500
H	-1.93740500	-2.49989100	0.11737600
H	-3.35934900	-0.72301100	-0.61798100
H	-2.96497800	1.48888700	-0.69173500
H	-1.31307800	2.82254300	-0.07735300
H	0.87424300	2.61581800	0.29849600
H	0.07914900	-0.70771300	-1.30441300
F	1.84035900	-1.57662600	-0.75578700
O	2.53544700	0.67590300	0.44691500
H	2.57935600	1.40663100	1.07912700

1-F-2-F-substituted

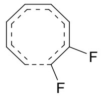

C	-0.47869200	-1.31975500	1.10060400
C	-1.59247600	-1.52142500	0.32725800
C	-2.36118500	-0.48394400	-0.24914000
C	-2.12213300	0.87855700	-0.34243500
C	-1.04542900	1.77007400	-0.05792800
C	0.32472000	1.68875000	0.11567000
C	1.22009500	0.62093300	-0.05997600
C	0.94404400	-0.60720600	-0.59785600
H	-0.33050600	-0.36464200	1.58980300
H	0.07712000	-2.16325000	1.50161900
H	-1.87338800	-2.54088100	0.06846800
H	-3.30908000	-0.79835000	-0.68315000
H	-2.99485200	1.42795000	-0.69457400
H	-1.39319800	2.80133900	-0.01291500
H	0.82206600	2.60864500	0.41507700
H	0.12597600	-0.76178700	-1.28590500
F	2.46062300	0.79758000	0.44449400
F	1.93295500	-1.50479400	-0.70178300

1-F-2-CHO-substituted

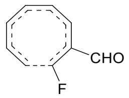

C	0.99915500	1.12803800	1.19033800
C	2.19160100	1.07163700	0.51637400
C	2.70485000	-0.09642600	-0.09870800
C	2.10904900	-1.32451500	-0.32497900
C	0.80685500	-1.90055100	-0.17406500
C	-0.49619100	-1.45256900	-0.09348300
C	-1.06935400	-0.16283200	-0.23270900
C	-0.34551200	0.93033900	-0.65242000
H	0.56025800	0.21580200	1.57639700
H	0.65789800	2.06144800	1.62997800
H	2.75557100	1.99149000	0.36922000
H	3.73550100	-0.02691800	-0.44160600
H	2.82191400	-2.07058300	-0.67630400
H	0.86356900	-2.98858600	-0.17244400
H	-1.24063700	-2.22220200	0.10016000
H	0.54258100	0.85849200	-1.26525000
F	-0.95722200	2.12251500	-0.77692000
C	-2.49364400	0.01337000	0.15070900
H	-2.89541300	1.03787600	0.07197500
O	-3.20338700	-0.89979900	0.51422600

1-F-2-CN-substituted

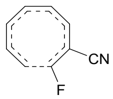

C	-0.76196100	-1.22414600	1.15482000
C	-1.94495400	-1.33175600	0.47311400
C	-2.64254900	-0.23633300	-0.09430200
C	-2.25495600	1.07937000	-0.27106300
C	-1.06415600	1.85361000	-0.09014500
C	0.29482800	1.62699100	-0.01392300
C	1.06042600	0.44487700	-0.21274100
C	0.52772900	-0.72779400	-0.69531000
H	-0.47004000	-0.27150000	1.58084200
H	-0.26851700	-2.10795900	1.54997900
H	-2.35348300	-2.32316300	0.28423500
H	-3.64977900	-0.45728600	-0.44247200
H	-3.07971100	1.71325500	-0.59593500
H	-1.29602900	2.91706600	-0.04833300
H	0.89338200	2.50429400	0.21791300
H	-0.36676800	-0.75170700	-1.30189200
F	1.32150700	-1.78677200	-0.87026700
C	2.43590600	0.44564900	0.20751300
N	3.54221500	0.46787700	0.54861400

1-F-2-NO_2-substituted

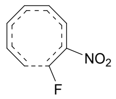

C	-1.08512800	-1.24177300	1.11707600
C	-2.28730900	-1.24525000	0.46402000
C	-2.92828500	-0.08590200	-0.04673700
C	-2.45909700	1.20334900	-0.21067500
C	-1.21208000	1.89501900	-0.05941800
C	0.12853500	1.57707000	-0.03733700
C	0.78525300	0.34574500	-0.27068500
C	0.16827300	-0.77322300	-0.77110700
H	-0.71489600	-0.32511800	1.56089300
H	-0.63397000	-2.16776000	1.46269800
H	-2.76617100	-2.20012500	0.25313100
H	-3.96016000	-0.22937000	-0.36175300
H	-3.24967000	1.89781300	-0.49406600
H	-1.37064100	2.96998900	0.01233800
H	0.80815000	2.39639100	0.17859600
H	-0.74800000	-0.66194300	-1.33638900
F	0.82398000	-1.89495400	-1.04586900
N	2.17685400	0.25438300	0.18061900
O	2.86268800	1.25864400	0.09533300
O	2.55238500	-0.81566700	0.62494300

1-CHO-2-CH_3-substituted

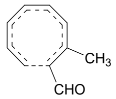

C	-0.41090400	-1.28171500	1.13369300
C	-1.49130500	-1.75099800	0.42733400
C	-2.47999000	-0.92848200	-0.15274800
C	-2.53878900	0.44727600	-0.34177200
C	-1.68873000	1.57936000	-0.17520800
C	-0.33692600	1.85920500	-0.00629600
C	0.81273300	1.03820200	-0.07510500
C	0.70764600	-0.24012700	-0.60102100
H	-0.44141000	-0.29071600	1.56957000
H	0.31538800	-1.97115300	1.55632200
H	-1.57248200	-2.82058700	0.24176400
H	-3.35937000	-1.45732300	-0.51753300
H	-3.52385100	0.76290000	-0.68469300
H	-2.27735600	2.49641800	-0.21092200
H	-0.13513000	2.90558900	0.22025600
H	-0.15833700	-0.45783500	-1.21574500
C	1.82385200	-1.19254100	-0.73174100
H	1.59747100	-2.08229200	-1.35395200
O	2.91317000	-1.08582800	-0.20277000
C	2.09635600	1.56111900	0.51799000
H	2.33481500	1.02511000	1.44451600
H	2.94761100	1.40324100	-0.14854600
H	2.00362800	2.62547700	0.75036100

1-CHO-2-NH_2-substituted

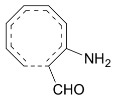

C	-0.35176600	-1.29538400	1.07928100
C	-1.46250900	-1.78479500	0.42069600
C	-2.46163600	-0.96940700	-0.13913200
C	-2.53976600	0.40662000	-0.34162800
C	-1.70310300	1.55276600	-0.19568100
C	-0.36482700	1.86375200	-0.02015600
C	0.78855600	1.03501800	-0.03455900
C	0.72444300	-0.26069500	-0.57451300
H	-0.40140900	-0.32831500	1.56643400
H	0.38850600	-1.98115300	1.48481400
H	-1.53899800	-2.85506600	0.24104800
H	-3.34332400	-1.50517000	-0.48956000
H	-3.52959300	0.70627200	-0.68301100
H	-2.30386200	2.46086700	-0.25714500
H	-0.16634500	2.91645500	0.17950800
H	-0.10671100	-0.46157300	-1.23779600
C	1.88928600	-1.12272800	-0.70446500
H	1.71477200	-2.06714500	-1.25482800
O	2.99939000	-0.89792500	-0.23578700
N	1.92271500	1.48625500	0.56104000
H	2.77768000	0.94970000	0.47112100
H	1.94309000	2.39385700	0.99936800

1-CHO-2-OH-substituted

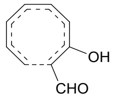

C	-0.33602800	-1.30315100	1.11319100
C	-1.39506300	-1.81357600	0.40062100
C	-2.41593200	-1.02682100	-0.17042200
C	-2.53941800	0.34696300	-0.34664600
C	-1.74046100	1.51432000	-0.17097800
C	-0.40705200	1.85117800	0.00395500
C	0.76522200	1.06053200	-0.04914100
C	0.77038800	-0.21674500	-0.58374100
H	-0.42119200	-0.32896600	1.57879700
H	0.42481600	-1.96125600	1.52458700
H	-1.42458900	-2.88190300	0.19564500
H	-3.27009900	-1.58932500	-0.54537400
H	-3.53541200	0.62130300	-0.69181400
H	-2.36449200	2.40709300	-0.21311200
H	-0.23394400	2.90781700	0.21330600
H	-0.06619500	-0.45655900	-1.22768800
C	1.95158600	-1.08811900	-0.70486700
H	1.77884800	-1.98042200	-1.34237100
O	3.02401200	-0.92828600	-0.16293400
O	1.90867200	1.54480600	0.48971700
H	1.73134000	2.38257200	0.94191800

1-CHO-2-F-substituted

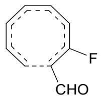

C	-0.32186000	-1.33471700	1.08600400
C	-1.37952200	-1.82845800	0.35605400
C	-2.40089700	-1.03645200	-0.20194000
C	-2.54275500	0.34293500	-0.33664800
C	-1.76198200	1.51104500	-0.12228900
C	-0.42554800	1.84492300	0.06143600
C	0.72326900	1.05319800	-0.05642300
C	0.77831200	-0.22214100	-0.57863400
H	-0.40980000	-0.37230200	1.57488100
H	0.43078500	-2.00641700	1.49049000
H	-1.40706400	-2.89267900	0.12981100
H	-3.24520200	-1.59759700	-0.60008500
H	-3.54344700	0.61228700	-0.67235100
H	-2.39239200	2.39970700	-0.13046000
H	-0.22504700	2.87964400	0.32930200
H	-0.04117200	-0.48754700	-1.23443500
F	1.83812100	1.59309100	0.46373200
C	2.00758200	-1.03352800	-0.69622100
H	1.91239900	-1.89604600	-1.38671000
O	3.03978100	-0.84471200	-0.09275800

1-CHO-2-CHO-substituted

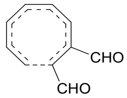

C	-0.91770000	-1.18124000	1.20453500
C	-2.10414000	-1.35578500	0.54202200
C	-2.83981300	-0.31454900	-0.07269300
C	-2.51189600	1.00677800	-0.33210900
C	-1.36505500	1.85398200	-0.23294400
C	0.01215800	1.72733700	-0.15194600
C	0.85672900	0.59519000	-0.24746900
C	0.38390300	-0.63876600	-0.66301800
H	-0.63023300	-0.19581800	1.55054800
H	-0.40430800	-2.02367700	1.65985200
H	-2.49793300	-2.36426800	0.42735700
H	-3.84527500	-0.58912700	-0.38714600
H	-3.37646300	1.57804500	-0.66970700
H	-1.66711000	2.90059100	-0.26874300
H	0.54642100	2.66138000	0.00671700
H	-0.53939500	-0.64570400	-1.23212600
C	1.16656700	-1.89265600	-0.74077900
H	0.75686900	-2.64589400	-1.44222500
O	2.15460800	-2.14838600	-0.08439300
C	2.29586300	0.81113600	0.11346200
H	2.98816200	-0.01762300	-0.09148700
O	2.69658900	1.85757800	0.57596700

1-CHO-2-CN-substituted

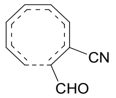

C	-0.78022400	-1.22757200	1.19944100
C	-1.93960300	-1.50760800	0.52663600
C	-2.78423500	-0.53675300	-0.06481900
C	-2.60222400	0.81484600	-0.30754800
C	-1.55332000	1.78041500	-0.20101700
C	-0.17258700	1.80393200	-0.11200000
C	0.77893400	0.75449400	-0.22095500
C	0.45080400	-0.50874100	-0.66926700
H	-0.59504300	-0.22789700	1.57333300
H	-0.18216100	-2.02424600	1.63374700
H	-2.22354000	-2.54908200	0.38542100
H	-3.75577800	-0.91157000	-0.38225500
H	-3.52332900	1.29356700	-0.63867600
H	-1.96348700	2.78951200	-0.23048700
H	0.25527100	2.78891900	0.05945600
H	-0.46776400	-0.60221100	-1.23538900
C	1.41272300	-1.63018700	-0.77439400
H	1.06198300	-2.48991700	-1.37799100
O	2.49464000	-1.65314300	-0.23449200
C	2.10803000	1.08033700	0.24245800
N	3.13241900	1.45987100	0.62536700

1-CHO-2-NO_2-substituted

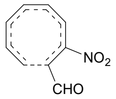

C	1.08641300	0.81951800	1.43298600
C	2.27837200	0.93252100	0.77537900
C	2.92437900	-0.11791900	0.06947000
C	2.47512300	-1.35331400	-0.35682000
C	1.23692600	-2.07254000	-0.39845100
C	-0.11269600	-1.79177300	-0.32778200
C	-0.76423700	-0.53877900	-0.28125700
C	-0.19052600	0.67713400	-0.54984700
H	0.68577100	-0.15603900	1.68352800
H	0.65795000	1.66997200	1.95614600
H	2.76183400	1.90680300	0.73967300
H	3.96338700	0.08305600	-0.18594200
H	3.28444200	-1.97086700	-0.74474400
H	1.41549200	-3.13461000	-0.56190500
H	-0.77586800	-2.65144800	-0.30811500
H	0.74191900	0.63261600	-1.10103600
C	-0.82793100	2.02288300	-0.59976800
H	-1.86663400	2.13003300	-0.25562500
O	-0.20071000	2.97199300	-1.01702700
N	-2.15152400	-0.58681400	0.22293500
O	-2.50968900	0.32408200	0.95358100
O	-2.84492100	-1.52960000	-0.10730400

1-CN-2-CH_3-substituted

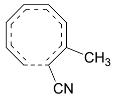

C	-0.33062100	-1.20305800	1.21839000
C	-1.33257200	-1.78693800	0.48187800
C	-2.34269200	-1.06539800	-0.18753400
C	-2.48310200	0.29519700	-0.43395500
C	-1.71462300	1.48302400	-0.26402700
C	-0.39015600	1.84517600	-0.04736600
C	0.80475600	1.09205000	-0.05129800
C	0.80620100	-0.21242900	-0.51628900
H	-0.46767000	-0.20212600	1.61026600
H	0.42689700	-1.81489000	1.70094600
H	-1.31846900	-2.86543500	0.33754800
H	-3.15884500	-1.67002700	-0.57992200
H	-3.46555500	0.53282800	-0.84123900
H	-2.35307800	2.36172600	-0.35803900
H	-0.25335500	2.90741800	0.14963500
H	0.01082200	-0.54179900	-1.17280700
C	2.02171300	-0.97279900	-0.57699200
N	2.99933500	-1.59536500	-0.60468000
C	2.04386300	1.68487700	0.56969900
H	2.18687100	1.28673200	1.58298300
H	2.94348300	1.44370700	-0.00311700
H	1.95695700	2.77120500	0.65146100

1-CN-2-NH_2-substituted

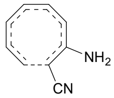

C	-0.30150200	-1.25467200	1.14960500
C	-1.31945100	-1.83030200	0.42487300
C	-2.34357400	-1.09027100	-0.19810400
C	-2.49391200	0.27609500	-0.40277800
C	-1.72458900	1.46452200	-0.22104300
C	-0.40723000	1.84530200	-0.02832300
C	0.79528300	1.09336700	-0.02854000
C	0.83028200	-0.21293800	-0.51537600
H	-0.45182800	-0.27768200	1.59462500
H	0.47270800	-1.87179400	1.59814800
H	-1.29930500	-2.90274400	0.24414000
H	-3.16714400	-1.68369900	-0.59326500
H	-3.48255000	0.52242700	-0.78799500
H	-2.36980400	2.34028700	-0.29475400
H	-0.26384300	2.91054100	0.14550600
H	0.07188200	-0.51682400	-1.22407400
C	2.07879300	-0.90810300	-0.55329300
N	3.10737200	-1.44520600	-0.52425400
N	1.92421700	1.68649700	0.49245400
H	2.77387000	1.13807200	0.53672800
H	1.81028800	2.35438400	1.24140100

1-CN-2-OH-substituted

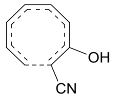

C	-0.26537000	-1.24717300	1.18922100
C	-1.23790900	-1.85520000	0.43278800
C	-2.27588400	-1.15880400	-0.21931600
C	-2.47996600	0.19952400	-0.43334300
C	-1.76425400	1.41615600	-0.24049400
C	-0.46068200	1.83407600	-0.01996500
C	0.75535300	1.11705300	-0.01986900
C	0.87285000	-0.17529500	-0.49662900
H	-0.45251400	-0.26915400	1.61663700
H	0.52234700	-1.83313300	1.65480500
H	-1.17306800	-2.92640700	0.25443100
H	-3.06374900	-1.78800100	-0.63102800
H	-3.47048500	0.40253100	-0.83837000
H	-2.43684200	2.26931500	-0.32840100
H	-0.35390800	2.90293200	0.16597000
H	0.11968900	-0.52729100	-1.18814800
C	2.14117000	-0.83712400	-0.53052000
N	3.14622600	-1.41353500	-0.55217700
O	1.86690600	1.67758300	0.52266900
H	1.63785900	2.48400900	1.00675200

1-CN-2-F-substituted

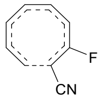

C	-0.24629200	-1.27557800	1.16800300
C	-1.21481200	-1.87185300	0.39231300
C	-2.25337100	-1.17561800	-0.25387600
C	-2.48330200	0.18649200	-0.43454600
C	-1.79394800	1.40655700	-0.20394500
C	-0.49114700	1.82729600	0.03605900
C	0.70834500	1.11268500	-0.01823700
C	0.88351000	-0.17587700	-0.47847100
H	-0.44091500	-0.30997000	1.61946700
H	0.53325400	-1.87446200	1.63113700
H	-1.14389800	-2.93987700	0.19703800
H	-3.02576300	-1.80778600	-0.68928000
H	-3.47717000	0.37883900	-0.83671200
H	-2.47681400	2.25337500	-0.26438900
H	-0.36543400	2.87785200	0.28557400
H	0.15235700	-0.55513300	-1.17928500
C	2.18267700	-0.77685600	-0.50880000
F	1.77662000	1.72746200	0.52456400
N	3.21497700	-1.30192400	-0.52509000

1-CN-2-CHO-substituted

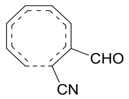

C	0.90080900	1.08416000	1.29859300
C	2.06038100	1.31893700	0.60424400
C	2.78003900	0.33189200	-0.10807200
C	2.45336900	-0.97434900	-0.43953200
C	1.31690300	-1.83399300	-0.34237000
C	-0.05434800	-1.71084300	-0.19439400
C	-0.89567600	-0.57631300	-0.20007100
C	-0.44821900	0.69031600	-0.53883200
H	0.65119900	0.07514000	1.60431600
H	0.39835600	1.89305300	1.82159900
H	2.43172200	2.33966700	0.53502900
H	3.76536300	0.64166200	-0.45160200
H	3.30504200	-1.51327100	-0.85415000
H	1.61699800	-2.87571900	-0.45360100
H	-0.59129700	-2.64789000	-0.06251600
H	0.44673300	0.78162900	-1.14130800
C	-1.31329600	1.83798300	-0.56400700
N	-1.97518100	2.78907300	-0.57233700
C	-2.30870800	-0.76763700	0.23911800
H	-2.92908800	0.14686100	0.27962500
O	-2.77703600	-1.84569400	0.52511400

1-CN-2-CN-substituted

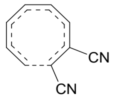

C	0.70619500	1.12661700	1.29437000
C	1.82084300	1.51724000	0.59963900
C	2.68791400	0.63414000	-0.08638100
C	2.56187600	-0.70976000	-0.40034900
C	1.56366000	-1.72675200	-0.30006000
C	0.18939200	-1.81218200	-0.16111300
C	-0.80643800	-0.80382300	-0.19622700
C	-0.55125700	0.50355500	-0.56953300
H	0.60153200	0.09779600	1.61815600
H	0.07745800	1.86073600	1.79042900
H	2.03539200	2.58040000	0.50991800
H	3.62154400	1.07923800	-0.42567400
H	3.48770900	-1.12373400	-0.79807700
H	2.01352700	-2.71420200	-0.39714300
H	-0.19455700	-2.81958000	-0.02196400
H	0.33300800	0.69873400	-1.16224700
C	-1.57835500	1.50128300	-0.62592200
N	-2.37966600	2.33666100	-0.66568400
C	-2.12007100	-1.16809700	0.27228000
N	-3.16578700	-1.48419100	0.65516600

1-CN-2-NO_2-substituted

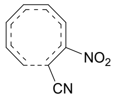

C	1.06169000	1.10405500	1.30360100
C	2.22435800	1.33403600	0.62032600
C	2.98160700	0.33798900	-0.04771000
C	2.68437600	-0.97351400	-0.37364900
C	1.55368100	-1.84730700	-0.30710600
C	0.17920500	-1.74157800	-0.21333500
C	-0.64313700	-0.59712900	-0.25813800
C	-0.23072700	0.66431600	-0.61210000
H	0.80438800	0.09953300	1.61936500
H	0.52638800	1.91832000	1.78352500
H	2.58236000	2.35785200	0.52979300
H	3.97574200	0.65411400	-0.35850100
H	3.55542300	-1.50793900	-0.75131700
H	1.86702600	-2.88706100	-0.39160400
H	-0.36084100	-2.67656100	-0.10141200
H	0.68777800	0.70781100	-1.18531800
C	-1.05215600	1.83327500	-0.71714300
N	-1.62126200	2.83313600	-0.84698500
N	-2.02308600	-0.78534700	0.23350000
O	-2.53236000	-1.88019100	0.07794300
O	-2.55279100	0.16950900	0.76973100

1-NO_2-2-CH_3-substituted

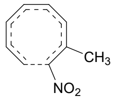

C	-0.45844100	-0.99539000	1.40637900
C	-1.34642900	-1.76446500	0.70293600
C	-2.41746900	-1.23480400	-0.05452400
C	-2.70553400	0.06575200	-0.43811700
C	-2.08554400	1.35249200	-0.37343500
C	-0.82286600	1.89672900	-0.19185700
C	0.46397700	1.30497300	-0.09030500
C	0.57589200	-0.02526100	-0.42224300
H	-0.72383000	0.01776200	1.68562800
H	0.36379700	-1.45154700	1.95220200
H	-1.19114200	-2.84006400	0.65369700
H	-3.14444400	-1.96720300	-0.40148900
H	-3.69428900	0.14658800	-0.88867900
H	-2.82493800	2.13240300	-0.55572500
H	-0.82184500	2.98267400	-0.11683500
H	-0.13669000	-0.50723600	-1.07465100
N	1.83136400	-0.74840800	-0.40148800
O	1.90899600	-1.71655900	-1.14350700
O	2.71288200	-0.38935600	0.36721500
C	1.58874100	2.14778200	0.45328400
H	1.81042000	1.86848900	1.48914800
H	2.51494700	2.01603900	-0.10967400
H	1.29949100	3.20141100	0.43442900

1-NO_2-2-NH_2-substituted

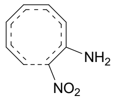

C	-0.38577300	-0.99754200	1.34540300
C	-1.29687900	-1.79693500	0.69229100
C	-2.38570700	-1.28004400	-0.03668800
C	-2.69797900	0.01657300	-0.42989500
C	-2.09822700	1.31409800	-0.39237500
C	-0.85877000	1.89896500	-0.20514000
C	0.43526500	1.32491500	-0.04924500
C	0.58831100	-0.02372300	-0.39040100
H	-0.68684600	-0.01203600	1.68431900
H	0.45913500	-1.44057900	1.86838500
H	-1.12358500	-2.86889100	0.64220300
H	-3.11238100	-2.02007800	-0.36934700
H	-3.69196300	0.07786200	-0.87040500
H	-2.85046500	2.07494500	-0.60216300
H	-0.87382100	2.98664400	-0.15294500
H	-0.10601200	-0.46478100	-1.08789600
N	1.85268200	-0.68708800	-0.40726300
O	1.90406000	-1.77055600	-0.96995100
O	2.81312200	-0.17521400	0.17811800
N	1.41480600	2.08032700	0.50179200
H	2.35607900	1.71678800	0.56567300
H	1.21854800	3.02576100	0.79143700

1-NO_2-2-OH-substituted

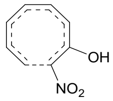

C	-0.39408400	-1.05041200	1.37183500
C	-1.24927500	-1.84493600	0.65446700
C	-2.34633000	-1.34564900	-0.08366900
C	-2.70100400	-0.05251700	-0.43898600
C	-2.14391900	1.26102200	-0.35467700
C	-0.91381100	1.86475900	-0.16359100
C	0.39188800	1.32060000	-0.05539100
C	0.62277700	0.00754400	-0.40545100
H	-0.71400700	-0.06593700	1.69308400
H	0.45658700	-1.47580300	1.89857000
H	-1.04081900	-2.90921100	0.57276600
H	-3.04022600	-2.10242900	-0.44634300
H	-3.69303200	-0.01061200	-0.88648600
H	-2.91647000	2.00828200	-0.53496300
H	-0.95124300	2.95236700	-0.09531800
H	-0.04895100	-0.48122300	-1.09315700
N	1.92518900	-0.61459200	-0.37725000
O	2.74949000	-0.23697000	0.43829700
O	2.09063100	-1.53798900	-1.16492300
O	1.38991700	2.08627300	0.43253500
H	1.03408100	2.93372800	0.73810200

1-NO_2-2-F-substituted

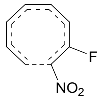

C	-0.40509200	-1.09910800	1.34896800
C	-1.26408800	-1.86292200	0.60141100
C	-2.35305800	-1.34170600	-0.13194700
C	-2.71545800	-0.03825400	-0.44608500
C	-2.16782500	1.27153000	-0.31334800
C	-0.93072200	1.86038700	-0.10437000
C	0.35166400	1.29050000	-0.05699900
C	0.62966900	-0.01061000	-0.39803700
H	-0.71315800	-0.11842100	1.69226400
H	0.43010200	-1.55413700	1.87558800
H	-1.06598200	-2.92734400	0.49615300
H	-3.04060700	-2.08962800	-0.52344300
H	-3.70801000	0.01077800	-0.89195300
H	-2.94090500	2.02527500	-0.45815800
H	-0.93047000	2.93898100	0.03150500
H	-0.01589400	-0.52606000	-1.09146300
F	1.31584900	2.08687100	0.41675500
N	1.96450100	-0.56825100	-0.35590500
O	2.19358000	-1.46194100	-1.15867200
O	2.74645100	-0.16086300	0.48523400

1-NO_2-2-CHO-substituted

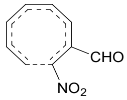

C	-0.90239700	-0.89508900	1.46751400
C	-1.99070400	-1.36753200	0.79200600
C	-2.84530300	-0.56378200	-0.00906900
C	-2.71023300	0.73661700	-0.45639200
C	-1.71150900	1.76516800	-0.43215000
C	-0.33942600	1.86532300	-0.30974000
C	0.67461800	0.87254700	-0.23579100
C	0.37058700	-0.44404300	-0.47432900
H	-0.79451400	0.16695200	1.65619800
H	-0.27272200	-1.56139200	2.05131100
H	-2.19940000	-2.43536700	0.80964200
H	-3.77122200	-1.04538100	-0.31828600
H	-3.62480600	1.10734900	-0.91850900
H	-2.16803200	2.73846800	-0.61051500
H	0.04645500	2.88095700	-0.27346300
H	-0.47942000	-0.74187800	-1.07019200
N	1.34871300	-1.51533900	-0.37486900
O	1.18234400	-2.46765700	-1.11848000
O	2.23790400	-1.42098500	0.45932900
C	2.05710400	1.36700700	0.09361700
H	2.89719900	0.68615700	-0.10048300
O	2.24338300	2.48792000	0.50719900

1-NO_2-2-CN-substituted

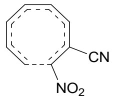

C	-0.81436800	-0.89603100	1.47611800
C	-1.86950800	-1.43242600	0.79641600
C	-2.79383100	-0.67961900	0.02313300
C	-2.76087500	0.62859700	-0.41892500
C	-1.83939500	1.72740500	-0.41156300
C	-0.48003700	1.93326400	-0.29574700
C	0.59866700	1.00304700	-0.22230300
C	0.39828700	-0.32757500	-0.47991400
H	-0.78298100	0.16597600	1.69217300
H	-0.13148500	-1.52859400	2.03754400
H	-1.99565000	-2.51306900	0.79035800
H	-3.68863400	-1.22331200	-0.27424900
H	-3.70669800	0.93388900	-0.86509300
H	-2.36520700	2.66456800	-0.59120600
H	-0.17357600	2.97547500	-0.26541200
H	-0.44090100	-0.66280100	-1.06941800
N	1.45895400	-1.31837100	-0.42414100
O	1.25522100	-2.34690600	-1.05041200
O	2.44279100	-1.08251800	0.25315500
C	1.86017800	1.55146400	0.21042500
N	2.81337900	2.11472800	0.54664400

1-NO_2-2-NO_2-substituted

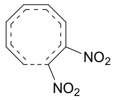

C	1.15677500	0.88981100	1.43513900
C	2.27172900	1.30137900	0.75858900
C	3.08118800	0.46059900	-0.04570600
C	2.88935800	-0.83285000	-0.50166500
C	1.84636600	-1.80800400	-0.48565700
C	0.47111900	-1.83554100	-0.34106900
C	-0.45195300	-0.77541300	-0.27769200
C	-0.15536700	0.54016700	-0.48816000
H	0.99046600	-0.16255100	1.63670800
H	0.56886500	1.59224900	2.02041300
H	2.53800700	2.35616900	0.78069500
H	4.02809600	0.89893900	-0.35590300
H	3.78797600	-1.24316700	-0.96055500
H	2.24504000	-2.80246500	-0.68243100
H	0.02599600	-2.82588300	-0.29116700
H	0.68343200	0.82837500	-1.10323000
N	-1.13385600	1.60491500	-0.33031600
O	-1.00242100	2.56299400	-1.07235200
O	-1.96547800	1.49337100	0.55203300
N	-1.82044800	-1.18140100	0.12453600
O	-1.91535900	-1.79049600	1.17251700
O	-2.72212300	-0.92176400	-0.64304200

5.3.2 1,3-substituted

1-CH_3-3-CH_3-substituted

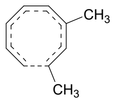

C	-1.49289800	-0.01405300	1.20283200
C	-2.04293700	0.97296800	0.41887500
C	-1.28509400	1.93883700	-0.27526200
C	0.08537600	2.02896100	-0.48757800
C	1.25296600	1.24259400	-0.25806000
C	1.57825100	-0.08623200	0.00971500
C	0.72325800	-1.21267700	-0.00424500
C	-0.57049400	-1.23241600	-0.47585700
H	-0.50532200	0.13166700	1.62020400
H	-2.13184800	-0.73031000	1.71381300
H	-3.11868400	0.97368100	0.24788100
H	-1.85757300	2.75376200	-0.71551500
H	0.36110800	2.98926800	-0.92495700
H	2.14328200	1.86508700	-0.35308200
H	-0.86000300	-0.43732000	-1.15497900
C	-1.37644800	-2.49986100	-0.55396600
H	-1.09345300	-3.19980900	0.23860600
H	-2.44857800	-2.29740200	-0.47146900
H	-1.21157900	-2.99592800	-1.51874700
H	1.11275700	-2.12618700	0.45129700
C	3.02805800	-0.38828400	0.34729600
H	3.46440000	-1.09383200	-0.36823100
H	3.64212200	0.51528900	0.35217700
H	3.10314300	-0.84699300	1.34049800

1-CH_3-3-NH_2-substituted

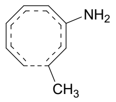

C	-1.51580400	0.03602000	1.20144400
C	-1.99180600	1.05383100	0.40809800
C	-1.18039100	1.98236100	-0.27499400
C	0.19804500	2.01132100	-0.47582000
C	1.32702600	1.17889600	-0.27154800
C	1.59182900	-0.16497500	0.02320300
C	0.69520500	-1.25851100	0.01105300
C	-0.59075800	-1.20718500	-0.47472700
H	-0.52794700	0.11809100	1.63441400
H	-2.20705400	-0.63797700	1.70181800
H	-3.06415400	1.11552800	0.22398700
H	-1.70856600	2.82267200	-0.72175200
H	0.51449900	2.95950300	-0.91210200
H	2.25459400	1.73247900	-0.42304200
H	-0.83136300	-0.39526600	-1.15358600
C	-1.45861900	-2.43178000	-0.56589700
H	-1.24478300	-3.13307600	0.24678800
H	-2.52030700	-2.17174200	-0.52980800

H	-1.27956500	-2.95035100	-1.51640800
H	1.03084500	-2.18023500	0.49055600
N	2.94198400	-0.46823300	0.28684600
H	3.11081500	-1.41219300	0.60943600
H	3.43073000	0.21032300	0.85691000

1-CH_3-3-OH-substituted

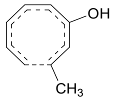

C	-1.50870500	0.07509900	1.18233500
C	-1.95898700	1.09236900	0.37522400
C	-1.12294500	2.00991600	-0.29633800
C	0.25739600	2.02380000	-0.46305000
C	1.36439700	1.16780000	-0.22384000
C	1.57826100	-0.18137400	0.03865800
C	0.68272700	-1.27224000	0.01957100
C	-0.60082500	-1.21273100	-0.47208300
H	-0.52334000	0.14416600	1.62414700
H	-2.21565700	-0.58520200	1.67872500
H	-3.02715400	1.16741900	0.17363400
H	-1.63280000	2.85672900	-0.75204000
H	0.60231100	2.96775500	-0.88560900
H	2.31612000	1.69107600	-0.30184300
H	-0.82811900	-0.40635800	-1.16161000
C	-1.48268200	-2.42791500	-0.55137100
H	-1.27677600	-3.12384100	0.26778400
H	-2.54103800	-2.15466700	-0.51697800
H	-1.31145000	-2.95713600	-1.49734900
H	1.01055500	-2.18794700	0.51695500
O	2.88959200	-0.45707400	0.36639300
H	3.05877600	-1.40375400	0.26840200

1-CH_3-3-F-substituted

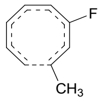

C	-1.46239500	0.28599300	1.17273400
C	-1.75890100	1.35847000	0.36514600
C	-0.80072400	2.15238200	-0.30137800
C	0.56683000	1.97626000	-0.46526500
C	1.52828600	0.96179200	-0.20988800
C	1.50548000	-0.39892500	0.03169300
C	0.49671600	-1.37299400	0.01223300
C	-0.77282900	-1.14026100	-0.46813700
H	-0.48161600	0.21089500	1.62370700
H	-2.26062100	-0.26414200	1.66455300
H	-2.80467600	1.58278600	0.15858400
H	-1.18802100	3.06338300	-0.75403800
H	1.04825100	2.85967600	-0.88405500
H	2.55291100	1.32700800	-0.25878400
H	-0.89484000	-0.31530800	-1.16170700
C	-1.81006200	-2.22625000	-0.53455600

H	-1.68173400	-2.94965600	0.27619900
H	-2.82195000	-1.81517600	-0.47754000
H	-1.72879700	-2.76575900	-1.48648500
H	0.74024200	-2.32351700	0.48441900
F	2.72960400	-0.90988700	0.35551800

1-CH_3-3-CHO-substituted

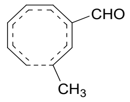

C	-1.72534200	0.19632000	1.24924800
C	-2.18198500	1.20990000	0.44661400
C	-1.34130100	2.08155000	-0.28123400
C	0.02421300	2.02335400	-0.52530100
C	1.07955200	1.09540800	-0.31089100
C	1.21585200	-0.26415900	-0.04739200
C	0.25616300	-1.30923300	-0.02868400
C	-1.03213800	-1.22422000	-0.49026600
H	-0.70614300	0.21934100	1.61303700
H	-2.42118700	-0.44159300	1.78797900
H	-3.25506800	1.32723800	0.30389200
H	-1.83505900	2.95088600	-0.71274600
H	0.40280900	2.94465000	-0.96763900
H	2.05703900	1.56910400	-0.40725000
H	-1.27335300	-0.39972000	-1.15240800
C	-1.97034600	-2.39660600	-0.50742400
H	-1.70041700	-3.13423800	0.25453000
H	-3.00465100	-2.08254700	-0.33623400
H	-1.94079400	-2.89194400	-1.48600000
H	0.57031100	-2.24632900	0.43717700
C	2.60859400	-0.71631600	0.24657900
H	2.69506500	-1.78854000	0.52120000
O	3.60148300	-0.02528600	0.20462100

1-CH_3-3-CN-substituted

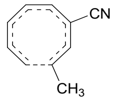

C	1.63458600	0.10583300	1.26130100
C	2.30386900	-0.81070000	0.48898900
C	1.67076000	-1.84671100	-0.23115700
C	0.32737800	-2.06579800	-0.50404700
C	-0.90343800	-1.37337000	-0.33337700
C	-1.31171300	-0.06092700	-0.09084700
C	-0.58043000	1.15609000	-0.06071500
C	0.71266300	1.29538500	-0.49938700
H	0.64182400	-0.12477400	1.62593700
H	2.17718400	0.88195100	1.79460700
H	3.38040500	-0.70813700	0.36224000
H	2.33690200	-2.60637800	-0.63641900
H	0.15220600	-3.04891800	-0.93961800
H	-1.75050600	-2.04672100	-0.45475400
H	1.10079600	0.52478500	-1.15684100

C	1.41741400	2.62118900	-0.52893000
H	1.02152500	3.30079900	0.23141900
H	2.49310800	2.50390600	-0.36714300
H	1.28601700	3.09443100	-1.50973100
H	-1.07816800	2.01326400	0.39147700
C	-2.72845600	0.08854200	0.16677600
N	-3.85958300	0.22265700	0.37816900

1-CH_3-3-NO_2-substituted

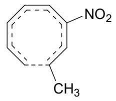

C	1.94553600	0.13074600	1.25598300
C	2.62477000	-0.80923300	0.52152100
C	1.99869700	-1.86897800	-0.16899500
C	0.65834200	-2.09330400	-0.45138900
C	-0.57125000	-1.39033900	-0.32206300
C	-0.96028800	-0.07657200	-0.12700500
C	-0.24788900	1.14214900	-0.11611900
C	1.04674700	1.25880200	-0.55604900
H	0.95133300	-0.09136500	1.62204900
H	2.47986900	0.92838700	1.76527600
H	3.70216500	-0.70870900	0.40157400
H	2.66739600	-2.64367900	-0.54021000
H	0.48459300	-3.08896000	-0.85717000
H	-1.42708500	-2.04898000	-0.43795800
H	1.43810900	0.46999600	-1.18978300
C	1.76026900	2.57913400	-0.61201900
H	1.36603600	3.27695900	0.13227000
H	2.83496100	2.45869200	-0.44576200
H	1.63336700	3.03164300	-1.60314800
H	-0.74659500	2.00359200	0.31759900
N	-2.43081900	0.08855100	0.11861100
O	-2.81917100	1.18651200	0.48076200
O	-3.16808000	-0.86674400	-0.04803700

1- NH_2-3-CH_3-substituted

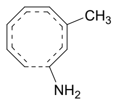

C	-1.43644900	-0.09278600	1.22164500
C	-2.06529800	0.87200500	0.45664900
C	-1.38152700	1.85358500	-0.27540400
C	-0.00785600	2.01049800	-0.49165900
C	1.17775700	1.27516500	-0.26768800
C	1.55044000	-0.05205700	0.01361300
C	0.76278600	-1.20552500	-0.02380200
C	-0.55583900	-1.26711200	-0.47726600
H	-0.45272300	0.11602600	1.62129200
H	-2.02855200	-0.82586100	1.76528100
H	-3.14737400	0.82646100	0.33423600
H	-1.99919000	2.62809900	-0.72633600
H	0.22117400	2.98345800	-0.92870300

H	2.05389200	1.91580100	-0.38478000
H	-0.90404500	-0.48818600	-1.14526000
N	-1.19804400	-2.48638400	-0.64412000
H	-0.94868400	-3.20298800	0.02754700
H	-2.19967300	-2.42982000	-0.77650100
H	1.19988100	-2.12843200	0.35899600
C	3.01940100	-0.24601600	0.35333300
H	3.31102600	0.35333600	1.22215700
H	3.24714900	-1.29288300	0.57015600
H	3.65293800	0.06314200	-0.48577300

1- NH_2-3-NH_2-substituted

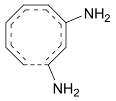

C	-1.44622800	-0.10047400	1.17301900
C	-2.03162000	0.91969300	0.43337000
C	-1.30732500	1.89151000	-0.26068500
C	0.07948900	2.01048500	-0.45551800
C	1.24115900	1.24423300	-0.26283600
C	1.57090600	-0.10177900	0.02750600
C	0.75065500	-1.22802700	-0.01485800
C	-0.57437100	-1.22478600	-0.46346800
H	-0.48242500	0.08394300	1.62995900
H	-2.08135900	-0.81627600	1.69138100
H	-3.11268600	0.91591700	0.29302000
H	-1.88986400	2.69273500	-0.71087600
H	0.33830100	2.98444800	-0.87301100
H	2.13704500	1.84765000	-0.41509300
H	-0.86784300	-0.44698800	-1.16028700
N	-1.25134700	-2.43036400	-0.65035900
H	-1.04315500	-3.14100700	0.04230800
H	-2.25119600	-2.33085100	-0.77617600
H	1.14930300	-2.15094000	0.40713800
N	2.90384200	-0.34109900	0.43031300
H	3.32451400	-1.16349400	0.01445600
H	3.51590500	0.45997000	0.33832400

1- NH_2-3-OH-substituted

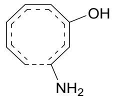

C	-1.43413300	-0.00184800	1.16887700
C	-1.94516900	1.05526500	0.42633200
C	-1.15656200	1.97735500	-0.26617100
C	0.23445100	2.00466900	-0.45747700
C	1.33391500	1.15431900	-0.25395200
C	1.54895900	-0.21224200	0.02439100
C	0.67839600	-1.29091300	-0.02348200
C	-0.64937700	-1.20363500	-0.46080700
H	-0.46004600	0.11491600	1.62573200
H	-2.11898200	-0.66841300	1.68925500
H	-3.02359900	1.12516500	0.28377800

H	-1.68386600	2.81549600	-0.71692300
H	0.56443800	2.95735400	-0.87284800
H	2.27883100	1.68016400	-0.40462500
H	-0.89806900	-0.41244300	-1.15952000
N	-1.40005600	-2.36274700	-0.63355000
H	-1.22613900	-3.09048000	0.05013600
H	-2.39178600	-2.21040800	-0.76603900
H	1.05315000	-2.23663200	0.36314300
O	2.83602300	-0.58014100	0.35404200
H	3.35539200	0.20782000	0.56416600

1- NH_2-3-F-substituted

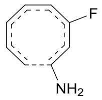

C	-1.43413300	-0.00184800	1.16887700
C	-1.94516900	1.05526500	0.42633200
C	-1.15656200	1.97735500	-0.26617100
C	0.23445100	2.00466900	-0.45747700
C	1.33391500	1.15431900	-0.25395200
C	1.54895900	-0.21224200	0.02439100
C	0.67839600	-1.29091300	-0.02348200
C	-0.64937700	-1.20363500	-0.46080700
H	-0.46004600	0.11491600	1.62573200
H	-2.11898200	-0.66841300	1.68925500
H	-3.02359900	1.12516500	0.28377800
H	-1.68386600	2.81549600	-0.71692300
H	0.56443800	2.95735400	-0.87284800
H	2.27883100	1.68016400	-0.40462500
H	-0.89806900	-0.41244300	-1.15952000
N	-1.40005600	-2.36274700	-0.63355000
H	-1.22613900	-3.09048000	0.05013600
H	-2.39178600	-2.21040800	-0.76603900
H	1.05315000	-2.23663200	0.36314300
O	2.83602300	-0.58014100	0.35404200
H	3.35539200	0.20782000	0.56416600

1- NH_2-3-CHO-substituted

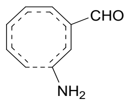

C	-1.65919800	0.09599400	1.23910900
C	-2.19013100	1.12523300	0.48255400
C	-1.41424400	2.02548300	-0.25939100
C	-0.03642900	2.02118400	-0.50598600
C	1.03495800	1.12657700	-0.31639600
C	1.19656900	-0.24372000	-0.05714300
C	0.27940500	-1.30334900	-0.05707700
C	-1.04445100	-1.23136400	-0.48265100
H	-0.64814900	0.19139000	1.61393800
H	-2.31855300	-0.56446800	1.79748500
H	-3.27247200	1.20452400	0.38626200
H	-1.94757100	2.87068200	-0.69080600

H	0.30890800	2.96462700	-0.92910700
H	2.00341000	1.61167400	-0.43457900
H	-1.32685900	-0.42748400	-1.15097500
N	-1.83366600	-2.36335500	-0.58767200
H	-1.65254800	-3.09670400	0.08717700
H	-2.82287700	-2.19956600	-0.71989500
H	0.62869500	-2.26197400	0.33172700
C	2.59685000	-0.67110100	0.23505200
H	2.70514900	-1.74594900	0.49292400
O	3.57481900	0.04088900	0.20764000

1-NH_2-3-CN-substituted

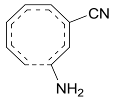

C	1.54696200	0.18588400	1.24537100
C	2.29284600	-0.73762400	0.52910300
C	1.72789200	-1.78425400	-0.20652200
C	0.38146800	-2.05215400	-0.48583100
C	-0.85354600	-1.39085600	-0.34310200
C	-1.28526700	-0.06976400	-0.10269700
C	-0.59549700	1.14820600	-0.09352600
C	0.73571800	1.30328400	-0.48651100
H	0.57589200	-0.11168300	1.62021400
H	2.05111600	0.97295000	1.80122500
H	3.37142100	-0.60289400	0.45664500
H	2.42511900	-2.51573900	-0.61005500
H	0.23942200	-3.04890700	-0.90265400
H	-1.69221600	-2.06827400	-0.49179000
H	1.15837100	0.55888600	-1.15036800
N	1.29959300	2.56023000	-0.60925400
H	0.97474300	3.26242700	0.04452300
H	2.30284600	2.58473000	-0.73393500
H	-1.12946000	2.02398600	0.27161200
C	-2.70358200	0.05148300	0.16164900
N	-3.83662400	0.15681200	0.37882200

1-NH_2-3-NO_2-substituted

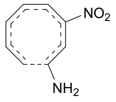

C	1.85306800	0.21960300	1.23461500
C	2.60697500	-0.73478400	0.56630700
C	2.04799100	-1.80776200	-0.13253100
C	0.70394500	-2.08021400	-0.42246900
C	-0.52896000	-1.40816600	-0.32836400
C	-0.93633100	-0.08369100	-0.13859400
C	-0.25935600	1.13139900	-0.15393400
C	1.07339500	1.26314700	-0.54857100
H	0.88195000	-0.06477100	1.61963600
H	2.35198900	1.03047900	1.76002200
H	3.68646000	-0.60440900	0.50156300
H	2.74700100	-2.55724600	-0.49828000

H	0.56334400	-3.09103800	-0.80351000
H	-1.37571400	-2.07130400	-0.47401600
H	1.50056500	0.49572500	-1.18264200
N	1.64149600	2.51331800	-0.71060900
H	1.30517200	3.24215800	-0.09285600
H	2.64619800	2.53336900	-0.82343700
H	-0.79549400	2.01016300	0.19126700
N	-2.40652600	0.06454800	0.11716300
O	-3.13521200	-0.88946100	-0.08668600
O	-2.80486500	1.14378900	0.52388800

1-OH-3-CH_3-substituted

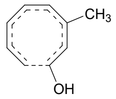

C	1.44305600	0.23429700	1.18849000
C	2.13959500	-0.69181900	0.43814400
C	1.52680500	-1.74874900	-0.25444200
C	0.17319300	-2.01397600	-0.46388700
C	-1.07236200	-1.36938500	-0.24861300
C	-1.54572300	-0.07395000	-0.00419300
C	-0.84259500	1.13883000	-0.06528100
C	0.46575600	1.29495200	-0.49987200
H	0.48739500	-0.05058200	1.61037800
H	1.97572600	1.03775900	1.69079000
H	3.20854200	-0.54998400	0.28539400
H	2.20095300	-2.48421900	-0.68955300
H	0.01803200	-3.00794300	-0.88500100
H	-1.88902500	-2.08482900	-0.34362400
H	0.90328700	0.58179900	-1.18526000
O	1.05387900	2.51630000	-0.60954500
H	0.54570500	3.17185700	-0.10749400
H	-1.35085100	2.02872900	0.31403400
C	-3.01286200	0.05580900	0.36965700
H	-3.12016600	0.25009700	1.44405900
H	-3.49432500	0.88103600	-0.16405800
H	-3.56548900	-0.86017000	0.14667100

1-OH-3-NH_2-substituted

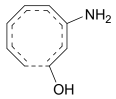

C	1.47154700	0.22207300	1.17961100
C	2.12165100	-0.73066700	0.41996800
C	1.47166400	-1.77329100	-0.25802600
C	0.10591000	-2.00826000	-0.44766400
C	-1.12162500	-1.34186600	-0.24893500
C	-1.56676700	-0.03425700	0.02129600
C	-0.84572100	1.16803200	-0.03881600
C	0.45956000	1.27868000	-0.49551500
H	0.51667700	-0.02705700	1.62485100
H	2.04248600	1.00742100	1.66848500
H	3.19221200	-0.62294900	0.24896600

H	2.11920300	-2.52564400	-0.70361100
H	-0.07246100	-2.99949500	-0.86610500
H	-1.96785500	-2.01304200	-0.39654500
H	0.86201700	0.54911300	-1.18599100
O	1.07966100	2.48194900	-0.62254300
H	0.60192800	3.15408500	-0.11307900
H	-1.31673000	2.06296900	0.37208200
N	-2.94639900	0.07481300	0.29332900
H	-3.25291600	0.99515400	0.58096700
H	-3.31436100	-0.64250800	0.90550900

1-OH-3-OH-substituted

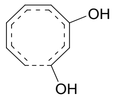

C	1.46520800	0.23711700	1.15527100
C	2.12113600	-0.69885300	0.38165700
C	1.48063600	-1.75991700	-0.27976100
C	0.11935900	-2.02909400	-0.42924600
C	-1.11285500	-1.37754900	-0.19694400
C	-1.55369100	-0.07286000	0.03801300
C	-0.87692800	1.15237600	-0.03227200
C	0.42342600	1.30239700	-0.49305300
H	0.52136100	-0.03238600	1.61294900
H	2.02832800	1.03198000	1.63759300
H	3.18523700	-0.56717400	0.18942300
H	2.13640900	-2.50015100	-0.73363600
H	-0.05365600	-3.02837600	-0.82944000
H	-1.96388300	-2.05240100	-0.26668200
H	0.83700600	0.59491500	-1.19907200
O	1.01440600	2.52107800	-0.60341000
H	0.53695800	3.17151400	-0.06629600
H	-1.37131000	2.02461500	0.40016300
O	-2.89090500	-0.02402900	0.37725700
H	-3.24221400	0.85935700	0.20223000

1-OH-3-F-substituted

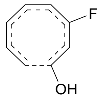

C	-1.46786300	-0.11652800	1.13452600
C	-2.03301700	0.87668500	0.35907900
C	-1.30576700	1.89095500	-0.28573100
C	0.07177900	2.05834000	-0.42051800
C	1.23364300	1.29281800	-0.17413900
C	1.52024200	-0.05064700	0.02864400
C	0.78387900	-1.22971400	-0.04558100
C	-0.53060400	-1.30083800	-0.48952700
H	-0.51145300	0.06825100	1.60762500
H	-2.10562200	-0.85918900	1.60679600
H	-3.10181100	0.83266200	0.15403700
H	-1.89926900	2.68324600	-0.73761200
H	0.33446300	3.04452800	-0.80274600

H	2.15055600	1.87829500	-0.20705400
H	-0.90968100	-0.58557100	-1.20569100
O	-1.19547800	-2.48165100	-0.56869100
H	-0.74563500	-3.15382600	-0.03404500
H	1.26601900	-2.12339700	0.34777100
F	2.82805700	-0.27313500	0.35332500

1-OH-3-CHO-substituted

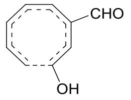

C	-1.70893100	0.05395000	1.21715000
C	-2.23912500	1.06840700	0.44970800
C	-1.46249200	2.00065100	-0.25915600
C	-0.08713500	2.02725300	-0.48560800
C	1.00285800	1.14911300	-0.28741400
C	1.19353600	-0.21590700	-0.05600100
C	0.29871800	-1.30341000	-0.07884300
C	-1.01501900	-1.26530700	-0.50469500
H	-0.70411000	0.15798700	1.60755300
H	-2.36705700	-0.63801700	1.73603300
H	-3.31833600	1.11398200	0.31323300
H	-2.00605000	2.84173300	-0.68600900
H	0.24689100	2.97977400	-0.89668200
H	1.96155500	1.66000400	-0.37505500
H	-1.36269000	-0.49835900	-1.18212200
O	-1.80885800	-2.36330600	-0.54915400
H	-1.40884500	-3.08745000	-0.04286200
H	0.66938600	-2.25314300	0.31483200
C	2.60332200	-0.61742900	0.23188400
H	2.73612600	-1.69451100	0.46843500
O	3.56370000	0.11756500	0.22171800

1-OH-3-CN-substituted

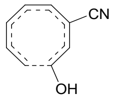

C	1.59053300	0.24517800	1.22370300
C	2.33805300	-0.65590700	0.49241600
C	1.78444000	-1.73881700	-0.20774900
C	0.44832100	-2.04783800	-0.46190300
C	-0.80778300	-1.41701800	-0.30671300
C	-1.27521800	-0.11078600	-0.09732700
C	-0.62102800	1.13578100	-0.11518800
C	0.69074900	1.33295200	-0.51332700
H	0.62938500	-0.06561000	1.61437200
H	2.08178100	1.06670700	1.73814700
H	3.40586700	-0.47976300	0.37450600
H	2.49568500	-2.45830900	-0.60852600
H	0.32602800	-3.05264500	-0.86487400
H	-1.62636500	-2.12542300	-0.41832500
H	1.17563100	0.63817300	-1.18497500
O	1.25930300	2.56091700	-0.57119400

H	0.71875200	3.20681700	-0.08998400
H	-1.17956500	1.99398600	0.25778800
C	-2.69593700	-0.02072400	0.16527100
N	-3.83062700	0.06454200	0.38233200

1-OH-3-NO_2-substituted

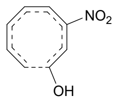

C	1.89738100	0.25839800	1.21486700
C	2.64680400	-0.67394500	0.52371700
C	2.08962200	-1.77083200	-0.14877100
C	0.75255100	-2.06898500	-0.41498400
C	-0.49558200	-1.41599300	-0.30173000
C	-0.92833500	-0.10409700	-0.13460600
C	-0.27641000	1.13309200	-0.16812100
C	1.03990700	1.29643400	-0.56395100
H	0.93257900	-0.03416600	1.61068300
H	2.38901500	1.09386400	1.70589700
H	3.71767000	-0.51187300	0.41455500
H	2.79630900	-2.51152100	-0.51784100
H	0.62209600	-3.08317000	-0.79003000
H	-1.33026800	-2.10106700	-0.41287500
H	1.52214800	0.57879300	-1.21300800
O	1.62641200	2.51440800	-0.64658900
H	1.08151300	3.18180200	-0.20062300
H	-0.83107300	1.99701400	0.18809400
N	-2.40194400	0.02047600	0.11877800
O	-3.10531200	-0.96396200	-0.01284300
O	-2.82635100	1.11487400	0.45257900

1-F-3-CH_3-substituted

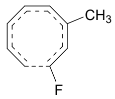

C	1.49357800	0.28736000	1.18556800
C	2.18918400	-0.61314800	0.41462600
C	1.58624900	-1.69039800	-0.26752100
C	0.24440400	-1.99408400	-0.46240400
C	-1.02739600	-1.39608100	-0.22017400
C	-1.54957600	-0.12619600	0.01454600
C	-0.88932100	1.12082000	-0.05607300
C	0.38852000	1.30762100	-0.51042600
H	0.53950400	-0.00385300	1.60660000
H	2.00795800	1.11315800	1.66979500
H	3.25039800	-0.44642500	0.23636800
H	2.27447800	-2.40964500	-0.70795000
H	0.11514600	-2.98870200	-0.89012300
H	-1.81251400	-2.14938900	-0.28565900
H	0.88637800	0.64824200	-1.20833800
H	-1.39955400	1.99936000	0.33672700
C	-3.02345400	-0.03563700	0.36847100
H	-3.56729100	0.58869000	-0.34855600

H	-3.49535300	-1.02057600	0.39036700
H	-3.15273700	0.41760200	1.35831600
F	0.87560600	2.56555500	-0.55080300

1-F-3-NH_2-substituted

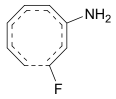

C	1.51937700	0.27777000	1.18630300
C	2.16977000	-0.64809400	0.40573300
C	1.53300800	-1.70997200	-0.26851400
C	0.17990200	-1.98613000	-0.45174300
C	-1.07775500	-1.37001600	-0.23196500
C	-1.57447400	-0.08762300	0.03065700
C	-0.89479700	1.14952500	-0.03953400
C	0.38144000	1.28901600	-0.51148800
H	0.56127300	0.02652800	1.62307600
H	2.07250600	1.08359900	1.66138900
H	3.23433500	-0.51619300	0.21577200
H	2.19873600	-2.44481600	-0.71669300
H	0.03295500	-2.97811900	-0.88002800
H	-1.89241700	-2.08424300	-0.35361300
H	0.84810000	0.60351200	-1.20673000
H	-1.36722300	2.03591300	0.37908200
N	-2.95038100	-0.01572100	0.30956500
H	-3.28493100	0.89017000	0.61004900
H	-3.31287500	-0.76084200	0.89008800
F	0.90482000	2.52974400	-0.56733800

1-F-3-OH-substituted

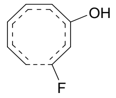

C	1.50970600	0.30347300	1.16565200
C	2.17321300	-0.59812300	0.37024200
C	1.55678500	-1.68320300	-0.28990600
C	0.21273700	-2.00377400	-0.43877200
C	-1.05383100	-1.41354900	-0.18500100
C	-1.55772000	-0.13952200	0.04607500
C	-0.93437200	1.12422200	-0.02951700
C	0.33218500	1.31710500	-0.50929200
H	0.56378600	0.02391200	1.61262300
H	2.04594200	1.12392600	1.63487200
H	3.22916500	-0.43297000	0.16022800
H	2.23751100	-2.39916300	-0.74621000
H	0.08007100	-3.00285800	-0.85400000
H	-1.86680400	-2.13578000	-0.23433100
H	0.81842700	0.66037900	-1.21800200
H	-1.43603500	1.98316100	0.41325500
O	-2.89073600	-0.14603700	0.39225300
H	-3.27525600	0.72956700	0.24969400
F	0.81076400	2.57537100	-0.54811500

1-F-3-F-substituted

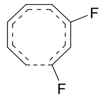

C	-1.50485700	-0.23542400	1.14531200
C	-2.12334000	0.69706200	0.34843200
C	-1.46160000	1.76478500	-0.29639300
C	-0.10742000	2.03733900	-0.43124900
C	1.12133500	1.37660300	-0.16177600
C	1.52861700	0.07263100	0.03810900
C	0.89370400	-1.17520400	-0.03765200
C	-0.38393400	-1.33908900	-0.50340100
H	-0.55457600	0.00175000	1.60719000
H	-2.08090100	-1.03393600	1.60520800
H	-3.18234300	0.57346500	0.12650400
H	-2.11266600	2.50864000	-0.75160400
H	0.07597300	3.03421500	-0.83099100
H	1.97840900	2.04752000	-0.17459600
H	-0.85703900	-0.68507500	-1.22243400
H	1.41594100	-2.03261100	0.37758200
F	2.84644100	-0.04050200	0.36652800
F	-0.89731100	-2.58240900	-0.51598800

1-F-3-CHO-substituted

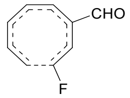

C	-1.76290900	0.04223800	1.22865200
C	-2.28161200	1.03556400	0.43843200
C	-1.49853400	1.97010200	-0.27427900
C	-0.12937200	2.00939100	-0.50009700
C	0.98087200	1.15112400	-0.27964700
C	1.20212500	-0.20306200	-0.05300700
C	0.32668100	-1.31638800	-0.08026100
C	-0.96521200	-1.29490400	-0.51563500
H	-0.75068800	0.12873000	1.60404400
H	-2.41795800	-0.65769300	1.74030300
H	-3.35887700	1.07790100	0.28716300
H	-2.04717300	2.80605600	-0.70503000
H	0.19289100	2.95884800	-0.92700900
H	1.92743100	1.68816000	-0.34580700
H	-1.37924000	-0.56614300	-1.19758600
H	0.68818500	-2.26172500	0.32369700
C	2.62116700	-0.57378900	0.24110400
H	2.77821900	-1.64748400	0.47397500
O	3.56068800	0.18703900	0.23813300
F	-1.67528100	-2.43829000	-0.48782000

1-F-3-CN-substituted

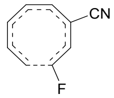

C	1.64728300	0.25788800	1.23734300
C	2.37174900	-0.62941500	0.48071600
C	1.80339500	-1.71331700	-0.22111600
C	0.47389600	-2.02378300	-0.47369600
C	-0.79536100	-1.40869500	-0.29521500
C	-1.28234900	-0.11707400	-0.09011700
C	-0.64171300	1.14698200	-0.11400700
C	0.64428000	1.34326300	-0.53003000
H	0.67362900	-0.03023800	1.61410100
H	2.13878600	1.08629400	1.74004500
H	3.43786500	-0.45701600	0.34425900
H	2.51324700	-2.43200400	-0.62618500
H	0.35595200	-3.02239400	-0.89259800
H	-1.60082800	-2.13527200	-0.38552100
H	1.17907100	0.68864600	-1.20396600
H	-1.18121100	2.00725500	0.27406500
C	-2.70336200	-0.04761900	0.17618300
N	-3.83867700	0.01632100	0.39592700
F	1.13859100	2.59234500	-0.51733900

1-F-3-NO_2-substituted

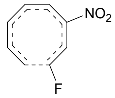

C	1.95499300	0.26859500	1.22798400
C	2.68008300	-0.65054500	0.51013700
C	2.10643900	-1.74841500	-0.16375200
C	0.77585100	-2.04570900	-0.42682000
C	-0.48433900	-1.40355600	-0.28810500
C	-0.93446300	-0.10478100	-0.13056800
C	-0.29675300	1.14995300	-0.17135900
C	0.99531400	1.31073500	-0.58149500
H	0.97619300	0.00057800	1.60635700
H	2.44778800	1.10974900	1.70773600
H	3.74956100	-0.49402700	0.38174900
H	2.81051900	-2.49028400	-0.53584300
H	0.64686800	-3.05432600	-0.81658000
H	-1.30993600	-2.10414100	-0.37207400
H	1.52625900	0.63299200	-1.23530100
H	-0.83253900	2.01793000	0.19743200
N	-2.41011400	0.00073900	0.12587500
O	-2.84461500	1.09158300	0.45187900
O	-3.09851900	-0.99556500	0.00354200
F	1.51315700	2.55006200	-0.59045600

1-CHO-3-CH_3-substituted

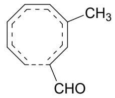

C	0.64650000	-1.40280700	1.12407300
C	0.00274400	-2.36307100	0.37894300
C	-1.25614800	-2.19098300	-0.22965400
C	-2.05502600	-1.06461900	-0.40684900
C	-1.99214800	0.34312900	-0.20651300
C	-1.03075500	1.33407700	0.00820500
C	0.36957600	1.19875100	-0.06585300
C	1.03669600	0.09964300	-0.56211600
H	0.07748800	-0.60023800	1.57476100
H	1.61854900	-1.60668000	1.56610100
H	0.51641600	-3.30243600	0.18077900
H	-1.69445300	-3.10146800	-0.63519800
H	-3.04118500	-1.33948000	-0.78152000
H	-2.99563600	0.76598800	-0.26384100
H	0.52283800	-0.58199200	-1.22941800
C	2.51643700	0.06776300	-0.57727800
H	2.97145300	-0.74460800	-1.17806900
O	3.22217500	0.84274400	0.03347300
H	0.98067500	2.00321800	0.34545700
C	-1.53317800	2.72651900	0.34447900
H	-1.25255200	3.44551500	-0.43286800
H	-2.61933800	2.75341300	0.45787800
H	-1.08984100	3.07640500	1.28353300

1-CHO-3-NH_2-substituted

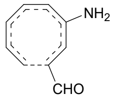

C	0.64116200	-1.44028900	1.12067900
C	-0.05773000	-2.35384500	0.36650300
C	-1.31451800	-2.13191900	-0.23008700
C	-2.08386500	-0.97951500	-0.39567000
C	-1.98842700	0.42135100	-0.21978600
C	-0.99718800	1.38598100	0.02117200
C	0.40168700	1.21989700	-0.05219500
C	1.01952100	0.10568100	-0.56719700
H	0.12607100	-0.60990100	1.58435100
H	1.60423200	-1.70608100	1.54941000
H	0.41125700	-3.31436400	0.15631700
H	-1.78421400	-3.02360700	-0.64113500
H	-3.07672800	-1.22953000	-0.77025400
H	-2.96632900	0.89222500	-0.32773000
H	0.47443800	-0.55889400	-1.22700000
C	2.49565800	0.00574100	-0.57182000
H	2.91455800	-0.84539600	-1.14372900
O	3.23264000	0.76987000	0.01590100
H	1.03850400	1.98906200	0.38514400
N	-1.46498600	2.68025600	0.29099200
H	-0.75662900	3.34784700	0.56608800
H	-2.28917700	2.73938500	0.87479900

1-CHO-3-OH-substituted

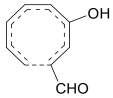

C	0.58094200	-1.42848100	1.10712600
C	-0.14064100	-2.32888000	0.35568600
C	-1.39152900	-2.07322300	-0.23798000
C	-2.12557200	-0.89881600	-0.39570600
C	-1.97432800	0.49622200	-0.19957900
C	-0.94005000	1.40901600	0.02677600
C	0.44919900	1.22175500	-0.04991700
C	1.02959300	0.07944900	-0.55305000
H	0.08075600	-0.59582200	1.58307600
H	1.53708500	-1.71757700	1.53624800
H	0.30595700	-3.29907100	0.14238200
H	-1.88719600	-2.94937300	-0.65182300
H	-3.12786500	-1.10916600	-0.76859000
H	-2.93927600	1.00169700	-0.27980400
H	0.46485300	-0.55783600	-1.22308900
C	2.50426800	-0.06313400	-0.57191800
H	2.89317100	-0.91425400	-1.16507300
O	3.26532000	0.66556300	0.02685600
H	1.08287600	1.99656200	0.37663200
O	-1.28248300	2.69784800	0.34814900
H	-2.22433600	2.75410500	0.56136000

1-CHO-3-F-substituted

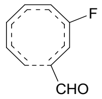

C	0.41147000	-1.48273200	1.10106800
C	-0.38414100	-2.29657900	0.33151600
C	-1.61067200	-1.91971400	-0.25613000
C	-2.23847400	-0.68612900	-0.38695600
C	-1.93841600	0.68148400	-0.15732500
C	-0.80153400	1.45038500	0.02540000
C	0.56672400	1.17443900	-0.06015900
C	1.06040700	-0.00630900	-0.56636200
H	-0.00388900	-0.60068500	1.57077000
H	1.33463700	-1.86395600	1.52970200
H	-0.02958700	-3.30051300	0.10325900
H	-2.18447600	-2.74408000	-0.67598100
H	-3.26188400	-0.78993000	-0.74554200
H	-2.82688800	1.31110600	-0.16140400
H	0.45330800	-0.60079100	-1.23773000
C	2.51955600	-0.26134000	-0.56605800
H	2.85394800	-1.12990900	-1.16601800
O	3.32133700	0.40111800	0.05583800
H	1.25555800	1.90184700	0.36463000
F	-1.07455000	2.74299200	0.35351600

1-CHO-3-CHO-substituted

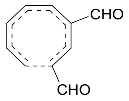

C	-1.47468200	0.97031400	1.18195300
C	-1.45728600	2.10887700	0.41880100
C	-0.31440200	2.61984200	-0.23407100
C	0.94794100	2.07811800	-0.45019400
C	1.60068000	0.83164300	-0.26746800
C	1.25187800	-0.50323500	-0.06535500
C	-0.00709800	-1.14006000	-0.11336600
C	-1.16709400	-0.57749700	-0.58631000
H	-0.55020600	0.56122000	1.56956600
H	-2.39110300	0.63474100	1.66018100
H	-2.39179900	2.64234900	0.25492000
H	-0.43020900	3.62695500	-0.63166200
H	1.64562900	2.82148400	-0.83434700
H	2.68489300	0.93812700	-0.32389600
H	-1.12627400	0.29080200	-1.23208300
C	-2.43550300	-1.34042900	-0.55121600
H	-3.27436500	-0.90694900	-1.12984400
O	-2.59150700	-2.36597800	0.07671900
H	-0.08031200	-2.14791400	0.29923000
C	2.39572700	-1.42047600	0.23379800
H	2.10133100	-2.46469600	0.46260500
O	3.56293700	-1.10385900	0.23651900

1-CHO-3-CN-substituted

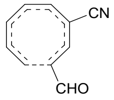

C	-1.55434700	0.64466000	1.19263300
C	-1.79273000	1.77424000	0.45130100
C	-0.79366400	2.53520800	-0.19168500
C	0.55290900	2.28285400	-0.42747700
C	1.47282400	1.21109100	-0.27866800
C	1.42311200	-0.17410500	-0.09376100
C	0.32890800	-1.06979800	-0.13151800
C	-0.92043400	-0.73504600	-0.59207600
H	-0.56395300	0.45011400	1.58473800
H	-2.37229600	0.10659500	1.66427300
H	-2.82257500	2.09108700	0.29694100
H	-1.12746900	3.50072700	-0.56787100
H	1.06722100	3.16590700	-0.80453700
H	2.49819400	1.56974700	-0.35543600
H	-1.04563900	0.12726800	-1.23594000
C	-2.01564700	-1.73509300	-0.57088000
H	-2.91552600	-1.47497200	-1.16095600
O	-1.97117700	-2.76906400	0.05832000
H	0.47325200	-2.06978500	0.27431900
C	2.70715900	-0.78926400	0.17007400
N	3.73280900	-1.28323700	0.38146500

1-CHO-3-NO_2-substituted

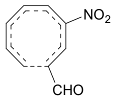

C	-1.91615200	0.48484600	1.19670200
C	-2.29297400	1.59758200	0.48790400
C	-1.39612500	2.48776800	-0.13924800
C	-0.03207100	2.40128600	-0.39152800
C	1.00475400	1.43774300	-0.27941800
C	1.09722300	0.06282000	-0.13058100
C	0.13905600	-0.96411200	-0.17484600
C	-1.14308800	-0.76527700	-0.62465700
H	-0.90832500	0.39987300	1.58309800
H	-2.66097100	-0.16126700	1.65384500
H	-3.35421300	1.79310000	0.34627200
H	-1.84329900	3.41737800	-0.48747800
H	0.37318300	3.34658000	-0.74884600
H	1.98732900	1.89459600	-0.35963000
H	-1.37748700	0.08898400	-1.24804100
C	-2.11114300	-1.88997000	-0.62159200
H	-3.03887100	-1.72597100	-1.20343600
O	-1.93985000	-2.92449100	-0.01663300
H	0.41073900	-1.94192300	0.21305600
N	2.49521600	-0.42496400	0.12415200
O	3.42420600	0.33930100	-0.06474900
O	2.62171000	-1.57140000	0.51209300

1-CN-3-CH_3-substituted

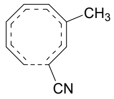

C	-0.93107100	1.08878900	1.22155900
C	-0.69197700	2.17957000	0.41712700
C	0.50041200	2.37651900	-0.30410400
C	1.58493200	1.53239700	-0.52887900
C	1.95682900	0.17986900	-0.28956400
C	1.35041700	-1.04136500	0.01437700
C	-0.02618600	-1.33909200	0.03113600
C	-1.01964400	-0.48618900	-0.41166400
H	-0.09362900	0.53359800	1.62542300
H	-1.87906900	0.98612600	1.74261200
H	-1.49065200	2.90139300	0.25731500
H	0.60543300	3.35638900	-0.76655300
H	2.41267300	2.06711400	-0.99470000
H	3.03438400	0.06853800	-0.41313200
H	-0.77513000	0.29399900	-1.12170500
C	-2.39328700	-0.90828200	-0.39926200
N	-3.50664700	-1.22884300	-0.37351200
H	-0.32801400	-2.28649900	0.47613800
C	2.26502100	-2.20968400	0.33756800
H	3.31349500	-1.90620400	0.37565400
H	2.00709200	-2.64122300	1.31126700
H	2.16727600	-3.00651800	-0.40749700

1-CN-3-NH_2-substituted

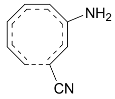

C	-0.89671900	1.14654700	1.22812300
C	-0.57831600	2.20325000	0.40784900
C	0.62847000	2.32701700	-0.30743900
C	1.67074900	1.42541600	-0.52203900
C	1.97378600	0.05950600	-0.30487500
C	1.30388500	-1.12733600	0.02644800
C	-0.08798800	-1.36121400	0.04573500
C	-1.02235300	-0.45960300	-0.41713300
H	-0.10709600	0.53009200	1.63812300
H	-1.85310700	1.11730700	1.74292500
H	-1.32928900	2.97233400	0.23474300
H	0.78769400	3.29531600	-0.77776600
H	2.52276700	1.91719400	-0.99137900
H	3.03239500	-0.13532500	-0.47983200
H	-0.72855700	0.30895100	-1.12163200
C	-2.41749800	-0.80019700	-0.40430000
N	-3.54585300	-1.06333900	-0.38224200
H	-0.44229200	-2.27736400	0.51490300
N	2.12653600	-2.23613700	0.28156500
H	1.64866200	-3.06600900	0.60728600
H	2.95993500	-2.04647500	0.82314800

1-CN-3-OH-substituted

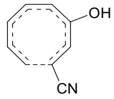

C	0.79924700	-1.21530900	1.21038100
C	0.40554900	-2.23261400	0.37529300
C	-0.81615300	-2.26517700	-0.32798200
C	-1.79891200	-1.29614600	-0.50828100
C	-1.99362700	0.08527000	-0.25573200
C	-1.21771700	1.20382400	0.03990200
C	0.18162400	1.35976700	0.05112000
C	1.05607200	0.40261300	-0.41768400
H	0.05768800	-0.54789100	1.63135600
H	1.75858700	-1.26042300	1.71842600
H	1.10138800	-3.04759800	0.18326700
H	-1.04633700	-3.21509400	-0.80658800
H	-2.69630600	-1.71803300	-0.96007000
H	-3.03864100	0.37575500	-0.35297600
H	0.71170100	-0.33769000	-1.12881600
C	2.47049700	0.64687400	-0.39770000
N	3.61434200	0.82992500	-0.36932500
H	0.58844100	2.24810900	0.53238400
O	-1.97610100	2.30584100	0.35874500
H	-1.44759300	3.11205800	0.28243700

1-CN-3-F-substituted

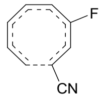

C	-0.71327500	1.24113700	1.18983800
C	-0.25145100	2.23530800	0.35977400
C	0.97860000	2.20007500	-0.32789100
C	1.90624200	1.17811500	-0.49905300
C	1.99730300	-0.21213900	-0.23434300
C	1.12209300	-1.25095600	0.03356700
C	-0.27253300	-1.36099900	0.03966800
C	-1.09742500	-0.35199800	-0.41565500
H	-0.01477500	0.53772300	1.62585200
H	-1.67264300	1.34538500	1.68890800
H	-0.89746900	3.08801700	0.15873700
H	1.26811700	3.13652700	-0.80104000
H	2.83596200	1.54050500	-0.93576200
H	3.01803300	-0.58610000	-0.29974300
H	-0.72022500	0.36410700	-1.13428500
C	-2.52356600	-0.51646900	-0.38748000
N	-3.67627200	-0.62557100	-0.34935700
H	-0.69279300	-2.25302900	0.49582500
F	1.74819600	-2.41850900	0.34405100

1-CN-3-CHO-substituted

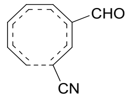

C	-1.43537900	0.86580200	1.24851900
C	-1.53401300	1.96807500	0.43550600
C	-0.46054200	2.50727400	-0.30104300
C	0.81996300	2.01906500	-0.54665900
C	1.53793600	0.81747900	-0.32832000
C	1.26492300	-0.52149400	-0.04294100
C	0.04424000	-1.22686400	-0.01012200
C	-1.16483300	-0.72521800	-0.43854600
H	-0.46602800	0.55209000	1.61616000
H	-2.30479100	0.49390100	1.78368500
H	-2.50906400	2.43017700	0.29524600
H	-0.65209400	3.48098800	-0.74895500
H	1.46259700	2.77334100	-0.99942900
H	2.61249900	0.96832900	-0.43930500
H	-1.19022600	0.10931200	-1.12683400
C	-2.35245500	-1.52958900	-0.38111600
N	-3.32248100	-2.16004900	-0.31667200
H	0.05759100	-2.22271900	0.43279100
C	2.46545900	-1.36565600	0.24938200
H	2.23666600	-2.41366600	0.53127800
O	3.61230200	-0.98808100	0.19551500

1-CN-3-CN-substituted

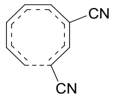

C	-1.44585300	0.65074400	1.26537000
C	-1.72460700	1.74104500	0.47649600
C	-0.75633500	2.45524600	-0.25510800
C	0.57839400	2.17185300	-0.52952200
C	1.48511200	1.09561500	-0.35341000
C	1.42194800	-0.27698700	-0.08589500
C	0.32116600	-1.16181700	-0.03684100
C	-0.94805100	-0.81728100	-0.44666800
H	-0.43856800	0.49465000	1.63221300
H	-2.24148800	0.13595800	1.79664800
H	-2.76157700	2.04574800	0.35059200
H	-1.10033800	3.39693700	-0.67903500
H	1.08856000	3.02244700	-0.97928700
H	2.51256000	1.42801000	-0.49319900
H	-1.08628600	0.00752100	-1.13412400
C	-2.01664000	-1.77488500	-0.39312200
N	-2.89586300	-2.52646000	-0.33236500
H	0.48619200	-2.14385800	0.39924900
C	2.70437900	-0.89950000	0.16577000
N	3.72784400	-1.40091400	0.37015500

1-CN-3-NO_2-substituted

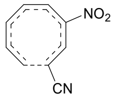

C	-1.80960200	0.51712300	1.26874500
C	-2.21723000	1.59652200	0.52049600
C	-1.33994900	2.43646200	-0.19025000
C	0.01596300	2.30959000	-0.47965100
C	1.03273200	1.33109400	-0.34499800
C	1.10278600	-0.03746900	-0.12434300
C	0.12863100	-1.04752500	-0.09123000
C	-1.17198700	-0.82545700	-0.48803000
H	-0.78939200	0.46760300	1.62970500
H	-2.53693200	-0.10306000	1.78532900
H	-3.28271700	1.78705200	0.41034700
H	-1.78668600	3.34869500	-0.58152000
H	0.42883500	3.22526600	-0.89959100
H	2.02230100	1.75881400	-0.47956100
H	-1.40365600	-0.00109200	-1.15016100
C	-2.13315700	-1.89244800	-0.45876500
N	-2.93045200	-2.73155600	-0.41706300
H	0.41250600	-2.01097000	0.31817100
N	2.49523400	-0.54384800	0.11683600
O	3.43295700	0.19486500	-0.11835000
O	2.60868600	-1.67884400	0.54297900

1-NO_2-3-CH_3-substituted

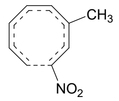

C	-1.80960200	0.51712300	1.26874500
C	-2.21723000	1.59652200	0.52049600
C	-1.33994900	2.43646200	-0.19025000
C	0.01596300	2.30959000	-0.47965100
C	1.03273200	1.33109400	-0.34499800
C	1.10278600	-0.03746900	-0.12434300
C	0.12863100	-1.04752500	-0.09123000
C	-1.17198700	-0.82545700	-0.48803000
H	-0.78939200	0.46760300	1.62970500
H	-2.53693200	-0.10306000	1.78532900
H	-3.28271700	1.78705200	0.41034700
H	-1.78668600	3.34869500	-0.58152000
H	0.42883500	3.22526600	-0.89959100
H	2.02230100	1.75881400	-0.47956100
H	-1.40365600	-0.00109200	-1.15016100
C	-2.13315700	-1.89244800	-0.45876500
N	-2.93045200	-2.73155600	-0.41706300
H	0.41250600	-2.01097000	0.31817100
N	2.49523400	-0.54384800	0.11683600
O	3.43295700	0.19486500	-0.11835000
O	2.60868600	-1.67884400	0.54297900

1-NO_2-3-NH_2-substituted

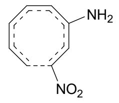

C	0.44790800	-1.25510200	1.32615600
C	0.01663500	-2.28586500	0.52840200
C	-1.19225100	-2.29081400	-0.19921500
C	-2.11534500	-1.28526800	-0.47184500
C	-2.26202300	0.11867800	-0.32102200
C	-1.45915400	1.22768900	-0.03182000
C	-0.04779200	1.28039000	0.03330700
C	0.75851000	0.25558300	-0.37247100
H	-0.26249100	-0.53702600	1.71625200
H	1.40263700	-1.32027100	1.84238600
H	0.67766100	-3.13758500	0.37744500
H	-1.45906300	-3.25155800	-0.63540000
H	-3.01120800	-1.69398300	-0.93910700
H	-3.29488600	0.41449300	-0.50961000
H	0.48095000	-0.49424900	-1.09790400
N	2.20480800	0.42456200	-0.29051400
O	2.87651300	-0.32025800	-0.98669200
O	2.65480200	1.26645300	0.47054200
H	0.41699000	2.13835500	0.51339700
N	-2.10515000	2.43430200	0.27314700
H	-1.67030800	3.26909200	-0.09953300
H	-3.10734200	2.43937900	0.13389200

1-NO_2-3-OH-substituted

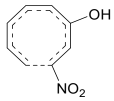

C	0.34882100	-1.30172000	1.33000700
C	-0.13114300	-2.28554100	0.50855500
C	-1.34128700	-2.21429400	-0.22289200
C	-2.20966600	-1.16157800	-0.47392400
C	-2.26962800	0.24661700	-0.28402400
C	-1.38861900	1.28872700	-0.02821200
C	0.02239300	1.28989700	0.02276600
C	0.78274400	0.23490500	-0.38776000
H	-0.31401100	-0.53206600	1.70627300
H	1.29456800	-1.42851600	1.85083700
H	0.47976500	-3.17188000	0.34673500
H	-1.66056300	-3.15559000	-0.66630300
H	-3.13264300	-1.50787100	-0.93818000
H	-3.27873200	0.63525000	-0.41242400
H	0.46974100	-0.51163100	-1.10110300
N	2.23219400	0.32844100	-0.28514600
O	2.87386700	-0.46453900	-0.95526300
O	2.71321300	1.16199600	0.46695200
H	0.53151100	2.12840500	0.49482200
O	-2.02374100	2.48288400	0.21319200
H	-1.40339200	3.22000500	0.12921600

1-NO_2-3-F-substituted

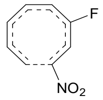

C	-0.28527500	1.29112000	1.31087300
C	0.23358100	2.26274200	0.49685100
C	1.44771800	2.15506600	-0.22265100
C	2.28271800	1.07579700	-0.46750500
C	2.27148600	-0.33278700	-0.26618700
C	1.32344400	-1.30895200	-0.03629900
C	-0.07924100	-1.30007000	0.00535500
C	-0.80919100	-0.21460000	-0.38590700
H	0.35096200	0.50349400	1.69599700
H	-1.22890300	1.44943400	1.82690700
H	-0.34747500	3.16802800	0.33168800
H	1.80181800	3.08707100	-0.65924700
H	3.22504600	1.38238200	-0.91956700
H	3.26021600	-0.77852600	-0.36469400
H	-0.47981800	0.52182100	-1.10188700
N	-2.26178500	-0.26428300	-0.27658600
O	-2.87950300	0.55768400	-0.93424200
O	-2.76232600	-1.08765700	0.47148300
H	-0.58232600	-2.15661600	0.44579400
F	1.85068400	-2.53969200	0.19733500

1-NO_2-3-CHO-substituted

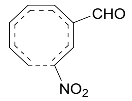

C	-0.98101700	1.18640000	1.35168200
C	-0.92723900	2.29126400	0.54566600
C	0.20498000	2.67310400	-0.21059800
C	1.38605200	2.00321000	-0.50229100
C	1.92186700	0.69938800	-0.32609000
C	1.45545300	-0.58799900	-0.07302200
C	0.13733800	-1.09443400	-0.01402200
C	-0.96917400	-0.39722400	-0.39809400
H	-0.06717300	0.70980500	1.68637700
H	-1.88905800	0.93772800	1.89504600
H	-1.82464100	2.89412900	0.42309500
H	0.15290500	3.67439400	-0.63513900
H	2.12287400	2.66031300	-0.96277200
H	3.00511700	0.69188100	-0.45481200
H	-0.97168500	0.42877600	-1.09111600
N	-2.28325000	-1.01448200	-0.27678100
O	-3.18722500	-0.48084300	-0.89997500
O	-2.40895400	-1.98808100	0.44869400
H	-0.01859800	-2.08258900	0.41726400
C	2.51619900	-1.61858200	0.16010600
H	2.13218200	-2.62562400	0.42081600
O	3.70543700	-1.42085200	0.08111700

1-NO_2-3-CN-substituted

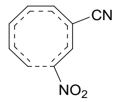

C	-0.97111200	1.08945600	1.36802800
C	-1.02525800	2.21049300	0.58313700
C	0.06112100	2.70301300	-0.17442100
C	1.28892800	2.14011900	-0.49501200
C	1.94781100	0.88716900	-0.35596500
C	1.59411700	-0.44213100	-0.11765500
C	0.32157200	-1.05978300	-0.04011400
C	-0.83055700	-0.42850700	-0.40327400
H	-0.01535700	0.69833900	1.69617400
H	-1.84822400	0.75063900	1.91383800
H	-1.97441500	2.73141000	0.47736700
H	-0.07927900	3.70287200	-0.58113000
H	1.95915900	2.86415800	-0.95601800
H	3.02007900	0.99477200	-0.51241600
H	-0.89068600	0.39710800	-1.09479000
N	-2.10011400	-1.13667300	-0.28055000
O	-3.03874500	-0.66326200	-0.89971200
O	-2.15334000	-2.11812600	0.44109400
H	0.25058300	-2.05762000	0.38536400
C	2.71194000	-1.34040000	0.07677300
N	3.60346500	-2.06149300	0.23648900

1-NO_2-3 -NO_2-substituted

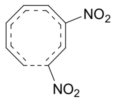

C	-1.41898200	0.99508300	1.37119000
C	-1.64799100	2.11079100	0.60984200
C	-0.65144000	2.78088800	-0.13390600
C	0.64825900	2.42028000	-0.46282300
C	1.48846000	1.27961300	-0.34541000
C	1.32696600	-0.07880200	-0.13402000
C	0.18697600	-0.90155000	-0.06420800
C	-1.04652800	-0.44551300	-0.42166000
H	-0.41408700	0.75550000	1.69816800
H	-2.23184700	0.50856000	1.90448600
H	-2.66637700	2.48013800	0.51132300
H	-0.94515100	3.75535100	-0.52013800
H	1.19875000	3.24591500	-0.91066600
H	2.53422400	1.53337300	-0.49753000
H	-1.23428600	0.36691400	-1.10605300
N	-2.19440300	-1.34023000	-0.30921300
O	-3.18623900	-1.01611500	-0.94209700
O	-2.10660800	-2.31525500	0.41634200
H	0.28304500	-1.89973400	0.35079100
N	2.61416300	-0.82809700	0.05375900
O	2.53269300	-2.01089900	0.32830000
O	3.66304000	-0.22428900	-0.07207300

5.3.4 1,4-substituted

1-CH_3-4-CH_3-substituted

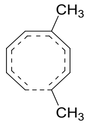

C	-1.25212300	1.00438200	1.10973900
C	-0.79994900	1.97626700	0.24774700
C	0.46558800	1.95743900	-0.37428500
C	1.45158200	0.98055300	-0.43108400
C	1.63947800	-0.37991000	-0.02755500
C	0.75947500	-1.41275900	0.28784300
C	-0.64207600	-1.53505000	0.19026200
C	-1.49703700	-0.64770900	-0.42680700
H	-0.53571900	0.39425200	1.64361300
H	-2.24275800	1.08285500	1.55138900
H	-1.48188100	2.76934200	-0.05576800
H	0.72672000	2.87049200	-0.90760900
H	2.36982800	1.37721200	-0.86922200
H	1.24701200	-2.30600800	0.67574900
H	-1.06554200	0.02623100	-1.15869600
C	-2.97220000	-0.91115500	-0.55336400
H	-3.34047700	-1.51343000	0.28328300
H	-3.54320700	0.02180600	-0.58604800
H	-3.18527200	-1.45523600	-1.48219700
H	-1.08518500	-2.38236100	0.71639500
C	3.11807200	-0.75578300	0.00606100
H	3.26932400	-1.78573400	0.33734100

H	3.55924600	-0.66047000	-0.99349600
H	3.68304700	-0.09660400	0.67393000

1-CH_3-4-NH_2-substituted

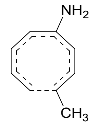

C	-1.19968000	1.01851300	1.11780000
C	-0.72671800	1.99759500	0.27500800
C	0.52585500	1.94601200	-0.37248200
C	1.47985500	0.94521000	-0.47356200
C	1.64394600	-0.41245900	-0.03956300
C	0.74577100	-1.42554700	0.30129000
C	-0.65262500	-1.52625300	0.17411600
C	-1.49776900	-0.62247500	-0.43290600
H	-0.49656400	0.37695200	1.63300700
H	-2.18365600	1.11719100	1.57049300
H	-1.38697100	2.81575400	-0.00735900
H	0.79505400	2.84955700	-0.91777700
H	2.38489100	1.28350300	-0.97890000
H	1.21855300	-2.31051300	0.72670700
H	-1.06305200	0.05316800	-1.16151100
C	-2.97809000	-0.86180500	-0.54815500
H	-3.34515700	-1.48024700	0.27744400
H	-3.53560800	0.08033300	-0.54628800
H	-3.21544900	-1.37649600	-1.48794700
H	-1.11026200	-2.36516200	0.70152000
N	3.00973500	-0.79094000	-0.06539400
H	3.19021300	-1.73140600	0.26266100
H	3.63660000	-0.12880300	0.37642600

1-CH_3-4-OH-substituted

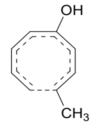

C	-1.18134200	1.04838900	1.10275800
C	-0.69994300	2.00595700	0.24325300
C	0.57080100	1.95030500	-0.36918500
C	1.52281500	0.94656800	-0.43116800
C	1.63635400	-0.41915100	-0.02947200
C	0.74445200	-1.43415800	0.29872900
C	-0.65833800	-1.52209700	0.19080700
C	-1.49192600	-0.62436400	-0.43582200
H	-0.48496200	0.41024600	1.63149000
H	-2.17028100	1.15318100	1.54235300
H	-1.35491300	2.82010800	-0.06207300
H	0.86541600	2.85526000	-0.89829500
H	2.46794700	1.27286800	-0.86178900
H	1.22048900	-2.32287100	0.71498300
H	-1.04849400	0.03974700	-1.17002400
C	-2.97495300	-0.84589500	-0.54774200

H	-3.34970300	-1.44948400	0.28518700
H	-3.52019600	0.10316200	-0.55782500
H	-3.21793400	-1.36867300	-1.48146700
H	-1.12047700	-2.34873700	0.73273100
O	2.97906000	-0.75957900	-0.00296700
H	3.07310800	-1.72149100	0.01551400

1-CH_3-4-F-substituted

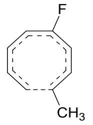

C	-1.12720400	1.08750400	1.09969400
C	-0.62919700	2.02048400	0.22506700
C	0.64669400	1.93907800	-0.37660200
C	1.56766800	0.90661500	-0.41348600
C	1.59241300	-0.46082000	-0.03050600
C	0.71069200	-1.47839100	0.27202800
C	-0.69680600	-1.52648600	0.19425000
C	-1.50273800	-0.60420500	-0.43101700
H	-0.44560800	0.43233400	1.62706700
H	-2.11255000	1.21625000	1.54013000
H	-1.26688300	2.84546800	-0.08770200
H	0.97346800	2.83625200	-0.89922000
H	2.53851800	1.19142000	-0.81553400
H	1.20947300	-2.37584500	0.63030600
H	-1.04105600	0.04431300	-1.16819400
C	-2.99316400	-0.77064400	-0.53068100
H	-3.38505100	-1.35100100	0.31051500
H	-3.50254700	0.19772900	-0.55039300
H	-3.25790600	-1.29505900	-1.45738800
H	-1.17672900	-2.34275900	0.73516000
F	2.89519200	-0.89754500	0.00919600

1-CH_3-4-CHO-substituted

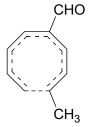

C	-1.67558800	0.81706100	1.18129300
C	-1.47546100	1.85013400	0.30364200
C	-0.25256700	2.12659000	-0.35514000
C	0.90745000	1.38104500	-0.46758700
C	1.36161900	0.06893300	-0.11740600
C	0.75614700	-1.14518400	0.19676900
C	-0.58535800	-1.56451200	0.14680700
C	-1.61914900	-0.86790600	-0.43945900
H	-0.83022100	0.34329400	1.66149100
H	-2.64489100	0.67574200	1.65248200
H	-2.32373400	2.47820000	0.03467600
H	-0.20916000	3.09155400	-0.85735400
H	1.71410900	1.96309800	-0.91773600
H	1.47016500	-1.89700100	0.53070400
H	-1.35696600	-0.08784500	-1.14606400
C	-3.01185800	-1.41890200	-0.53809800

H	-3.22276100	-2.11832600	0.27635900
H	-3.75953500	-0.62044000	-0.51805200
H	-3.13582100	-1.95540500	-1.48741800
H	-0.82127900	-2.50092100	0.65331800
C	2.86151100	-0.01193900	-0.12608100
H	3.37399500	0.89888100	-0.49901000
O	3.51820300	-0.96034500	0.23851900

1-CH_3-4-CN-substituted

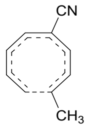

C	-1.56609800	0.92561600	1.13174400
C	-1.23307500	1.91434900	0.24153400
C	0.03297200	2.04616400	-0.37568000
C	1.11572300	1.18716600	-0.43313500
C	1.40235300	-0.16480800	-0.03990900
C	0.66364000	-1.30532200	0.28300100
C	-0.71595900	-1.56777100	0.20315300
C	-1.64525300	-0.77360400	-0.43246100
H	-0.78655900	0.39460600	1.66153900
H	-2.55963500	0.89183700	1.57150100
H	-2.00314600	2.61663200	-0.07423300
H	0.19483100	2.98679800	-0.89864600
H	1.99744100	1.66025200	-0.86223300
H	1.26654200	-2.13046500	0.65608500
H	-1.27303300	-0.06038200	-1.15996500
C	-3.08926000	-1.16507600	-0.55813400
H	-3.40712000	-1.78984600	0.28181300
H	-3.73849800	-0.28605500	-0.60630300
H	-3.24367700	-1.73429300	-1.48340200
H	-1.07035700	-2.45187600	0.73336500
C	2.83353200	-0.41084600	-0.00905400
N	3.97596600	-0.60177300	0.01630300

1-CH_3-4-NO_2-substituted

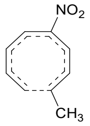

C	-1.94679600	0.90860100	1.13886400
C	-1.64295300	1.90316000	0.24470200
C	-0.38325100	2.07476600	-0.37616800
C	0.73234300	1.25960800	-0.43176600
C	1.04747000	-0.07497500	-0.04770300
C	0.36388000	-1.24055600	0.26097300
C	-1.00772900	-1.54047700	0.19183300
C	-1.97044200	-0.77915400	-0.43555700
H	-1.15372700	0.40072500	1.67099700
H	-2.93928300	0.84759700	1.57798600
H	-2.43558800	2.57891700	-0.07329000
H	-0.26009400	3.01502600	-0.90983800
H	1.59629300	1.75806200	-0.85942300
H	0.99872400	-2.04220300	0.62281100

H	-1.63119100	-0.05377400	-1.16725500
C	-3.39900400	-1.22579600	-0.55038400
H	-3.68849000	-1.85672100	0.29507700
H	-4.08147700	-0.37270300	-0.60219200
H	-3.53428300	-1.80724200	-1.47105200
H	-1.32947300	-2.43235600	0.72927200
N	2.54572200	-0.28297600	-0.00476500
O	3.26153700	0.70353600	-0.00084300
O	2.97314200	-1.42423000	0.03228100

1- NH_2-4-CH_3-substituted

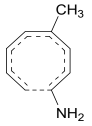

C	-1.25892900	0.92609600	1.10885000
C	-0.83377100	1.94776900	0.27430000
C	0.41254700	1.96501100	-0.36305400
C	1.42934200	1.00392600	-0.40780900
C	1.62790600	-0.34532400	-0.02086200
C	0.74346200	-1.39360700	0.28721000
C	-0.63685700	-1.54299900	0.16255700
C	-1.51412300	-0.63083200	-0.42975000
H	-0.51411400	0.35607500	1.64880700
H	-2.24256500	0.98341400	1.57079100
H	-1.53605900	2.73656100	0.00600300
H	0.65015600	2.88104000	-0.90155900
H	2.34684000	1.42525500	-0.82489400
H	1.23639300	-2.28204100	0.67853300
H	-1.11218300	0.05410000	-1.16608400
N	-2.85158400	-0.95992600	-0.63546700
H	-3.26729700	-1.53263100	0.09025200
H	-3.44883300	-0.17856900	-0.87490200
H	-1.07607800	-2.43334700	0.61384500
C	3.10539600	-0.73115000	-0.00535600
H	3.68576200	-0.07598400	0.65294300
H	3.25352400	-1.76097100	0.32874900
H	3.53569900	-0.64676500	-1.01072500

1- NH_2-4-NH_2-substituted

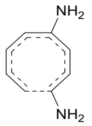

C	-1.21122300	0.94523000	1.12100300
C	-0.76803000	1.96740700	0.29816300
C	0.46822600	1.95496200	-0.36264500
C	1.45436500	0.97138000	-0.45491700
C	1.63296000	-0.37576100	-0.03612700
C	0.73608600	-1.40522400	0.29635100
C	-0.64534300	-1.53467100	0.15054200
C	-1.51078600	-0.61252700	-0.43550200
H	-0.47799800	0.34351300	1.64290000
H	-2.18948500	1.02085200	1.59208100
H	-1.45149200	2.77710700	0.04601400

H	0.71190200	2.86309700	-0.91166900
H	2.35973800	1.33484300	-0.94218100
H	1.21621100	-2.28697300	0.71954400
H	-1.10567300	0.07642500	-1.16644200
N	-2.85878600	-0.91906000	-0.63302800
H	-3.27392300	-1.48432200	0.09924200
H	-3.44118000	-0.11806200	-0.84462000
H	-1.09720100	-2.41212500	0.61468700
N	3.00410400	-0.75792500	-0.07397800
H	3.17435900	-1.71377600	0.21304700
H	3.61998000	-0.12646200	0.42523500

1- NH_2-4-OH-substituted

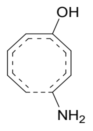

C	-1.18925600	0.97769800	1.10867700
C	-0.73510900	1.97842800	0.26898800
C	0.51733300	1.95844600	-0.36194400
C	1.49847500	0.96916600	-0.41626600
C	1.62450000	-0.38500000	-0.02491100
C	0.73114600	-1.41258800	0.29959300
C	-0.65434200	-1.52903800	0.16800500
C	-1.50738900	-0.61212900	-0.43974700
H	-0.46453600	0.37509000	1.64137700
H	-2.17187100	1.06310800	1.56804200
H	-1.41156000	2.78713200	-0.00438900
H	0.78555900	2.86600300	-0.89978300
H	2.44180900	1.31712200	-0.83412300
H	1.21350800	-2.29361200	0.72360600
H	-1.09344200	0.06721800	-1.17463100
N	-2.85726600	-0.90209800	-0.63309700
H	-3.28387800	-1.45072300	0.10498800
H	-3.42963100	-0.10074900	-0.86833900
H	-1.11270600	-2.39537500	0.64561400
O	2.96967200	-0.73687300	-0.00003200
H	3.05808400	-1.69544600	-0.08478700

1- NH_2-4-F-substituted

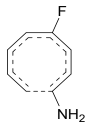

C	-1.13344100	1.01089800	1.10207100
C	-0.65730800	1.99410400	0.25426100
C	0.59895400	1.94849700	-0.36757400
C	1.54889200	0.92789100	-0.39516700
C	1.58248200	-0.42857700	-0.02627000
C	0.69264600	-1.45788700	0.26745400
C	-0.69598100	-1.53115800	0.16503200
C	-1.52316100	-0.58090600	-0.43269000
H	-0.42333000	0.39043300	1.63422700
H	-2.11149100	1.12351900	1.56435500
H	-1.31455300	2.81758600	-0.02192400

H	0.90042400	2.84870000	-0.89919600
H	2.52096000	1.23456000	-0.77836900
H	1.19383300	-2.35304300	0.62748900
H	-1.09309600	0.08163300	-1.17369800
N	-2.88053000	-0.82003300	-0.60743100
H	-3.32561300	-1.35497600	0.12914700
H	-3.43064000	-0.01144000	-0.86707200
H	-1.17459400	-2.39317200	0.62904600
F	2.88259100	-0.88230400	0.00059000

1- NH_2-4-CHO-substituted

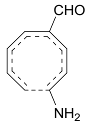

C	-1.60166300	0.90289900	1.07993800
C	-1.15000300	1.93527000	0.26929700
C	0.12623500	1.98493900	-0.30393800
C	1.14591100	1.03080500	-0.31488900
C	1.29916400	-0.32847600	0.04578000
C	0.42296200	-1.40273700	0.32081300
C	-0.94134800	-1.55949600	0.16353700
C	-1.79595900	-0.61978200	-0.44632400
H	-0.87688200	0.35089400	1.66355500
H	-2.60460200	0.95369000	1.49830700
H	-1.85178600	2.71597700	-0.02393000
H	0.38061700	2.91483600	-0.80883600
H	2.09522700	1.43196900	-0.67022700
H	0.92748500	-2.28682100	0.71176000
H	-1.35897500	0.04501400	-1.18187200
N	-3.11908400	-0.94181300	-0.69383700
H	-3.57563200	-1.51547300	0.00516800
H	-3.70725400	-0.18340200	-1.01264000
H	-1.39697900	-2.46015700	0.57377300
C	2.73032500	-0.75974700	0.08845000
H	2.88650900	-1.80285900	0.43442200
O	3.68776400	-0.08412800	-0.21857700

1-NH_2-4-CN-substituted

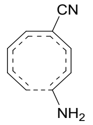

C	-1.57075400	0.81714800	1.10997800
C	-1.26303900	1.87652500	0.27040700
C	-0.02144000	2.05210300	-0.35268200
C	1.09461300	1.21309700	-0.39367700
C	1.39109900	-0.12612600	-0.02735000
C	0.64489200	-1.28722600	0.28613500
C	-0.70370700	-1.57303000	0.17092100
C	-1.66673200	-0.74185300	-0.42935300
H	-0.76985000	0.34107800	1.66002400
H	-2.55681700	0.76241300	1.56567500
H	-2.05298600	2.57574900	-0.00229500
H	0.11675600	2.99568400	-0.87606900

H	1.97486000	1.71704500	-0.79024800
H	1.25179600	-2.10540500	0.66678000
H	-1.32578400	-0.03188800	-1.17329400
N	-2.95400600	-1.19745000	-0.63270300
H	-3.33009300	-1.81949200	0.07243800
H	-3.62805300	-0.51388300	-0.94993700
H	-1.05241900	-2.50815400	0.60755400
C	2.81828500	-0.38212300	-0.01829200
N	3.95876000	-0.58886900	-0.00689000

1-NH_2-4-NO_2-substituted

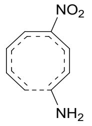

C	-1.95556600	0.79174500	1.11368900
C	-1.66607100	1.86493300	0.28389500
C	-0.42707600	2.08047300	-0.33035100
C	0.71848000	1.28203200	-0.37067200
C	1.04029700	-0.04315700	-0.02454300
C	0.34094100	-1.22823300	0.26779000
C	-0.99795800	-1.54337600	0.15138600
C	-1.99364100	-0.73797400	-0.43854600
H	-1.15088500	0.33473800	1.67391100
H	-2.94489900	0.71196100	1.55841300
H	-2.47319400	2.54315700	0.00807400
H	-0.31875500	3.02822800	-0.85277600
H	1.58516200	1.81138700	-0.75426700
H	0.97162100	-2.02744000	0.63986500
H	-1.68117400	-0.01519200	-1.18285400
N	-3.26048500	-1.24271200	-0.64170600
H	-3.61066200	-1.88903500	0.05476600
H	-3.96276600	-0.59052400	-0.96307400
H	-1.32002600	-2.48898900	0.58585300
N	2.52911900	-0.26626400	-0.01353900
O	3.26466700	0.70532200	-0.07550700
O	2.94392100	-1.41208600	0.06337300

1-OH-4-CH_3-substituted

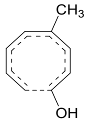

C	-1.27243900	0.95166100	1.09005100
C	-0.83017100	1.95716800	0.25289500
C	0.43077500	1.96583700	-0.36401000
C	1.43477100	0.99899800	-0.40425500
C	1.61659300	-0.35741800	-0.01440100
C	0.72273600	-1.39672100	0.26566500
C	-0.66394300	-1.53479400	0.12487800
C	-1.53215800	-0.61829800	-0.45228700
H	-0.54192800	0.36580800	1.63352900
H	-2.26857700	1.00813300	1.52203000
H	-1.52772900	2.74029900	-0.04037600
H	0.68181200	2.88193600	-0.89628200

H	2.35954900	1.40884400	-0.81600200
H	1.20041300	-2.29730100	0.64725200
H	-1.18245900	0.07560400	-1.20369200
O	-2.86327900	-0.86788100	-0.59064600
H	-3.12208300	-1.60211500	-0.01266200
H	-1.11077000	-2.42223400	0.57710600
C	3.09161500	-0.74719500	0.03446200
H	3.22956400	-1.78009600	0.36294600
H	3.54617200	-0.65326900	-0.95922100
H	3.65558900	-0.09798400	0.71255400

1-OH-4-NH_2-substituted

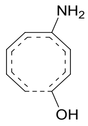

C	-1.22501400	0.96792300	1.09893200
C	-0.76187800	1.97773100	0.27851500
C	0.48818400	1.95480600	-0.36206900
C	1.46164000	0.96429700	-0.44841200
C	1.62169400	-0.38918000	-0.02710400
C	0.71238700	-1.40941500	0.27821900
C	-0.67332400	-1.52566200	0.11208200
C	-1.52881800	-0.59452700	-0.45619500
H	-0.50701600	0.35180100	1.62625000
H	-2.21594700	1.04435600	1.54012300
H	-1.43926400	2.78422300	0.00354700
H	0.74694300	2.86214300	-0.90548200
H	2.37511300	1.31647600	-0.92841600
H	1.17551800	-2.30369700	0.69314200
H	-1.17400800	0.10226300	-1.20254500
O	-2.86783500	-0.82151300	-0.58744900
H	-3.13480800	-1.55373300	-0.01108100
H	-1.13484300	-2.40103500	0.57321100
N	2.98939700	-0.77642200	-0.03622100
H	3.15407200	-1.73066800	0.25895400
H	3.60191600	-0.14090900	0.46163500

1-OH-4-OH-substituted

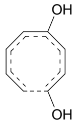

C	-1.20319400	1.00003200	1.08306000
C	-0.72832500	1.98863100	0.24681400
C	0.53975300	1.95876000	-0.35861900
C	1.50735300	0.96208400	-0.40520900
C	1.61299400	-0.39948300	-0.01601300
C	0.70680500	-1.41865300	0.27710900
C	-0.68361200	-1.52101400	0.12835600
C	-1.52593700	-0.59387400	-0.46086400
H	-0.49391300	0.38493600	1.62337500
H	-2.19900200	1.08506700	1.51076000
H	-1.39812500	2.79340100	-0.05083400
H	0.82577100	2.86642800	-0.88713000

H	2.45983700	1.29893400	-0.81084700
H	1.17146600	-2.31473000	0.68841300
H	-1.16132600	0.09185200	-1.21261600
O	-2.86681900	-0.80056900	-0.58796100
H	-3.14773300	-1.51643200	0.00219900
H	-1.15140700	-2.38494200	0.60356400
O	2.95398200	-0.75534000	0.03609800
H	3.04211000	-1.71615100	-0.01978800

1-OH-4-F-substituted

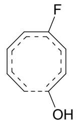

C	-1.14462800	1.04192700	1.07524400
C	-0.64568600	2.00751000	0.22770300
C	0.62841900	1.94762800	-0.36439300
C	1.56030300	0.91719600	-0.38291200
C	1.56816900	-0.44633100	-0.01742800
C	0.66467400	-1.46344800	0.24769500
C	-0.72998200	-1.52131900	0.12925500
C	-1.54234200	-0.56390900	-0.45516700
H	-0.45252900	0.40735500	1.61555700
H	-2.13664900	1.15631400	1.50397000
H	-1.29391100	2.82753500	-0.07602700
H	0.95215800	2.84692800	-0.88454400
H	2.54196400	1.20807600	-0.75331400
H	1.14828900	-2.37161700	0.59836000
H	-1.15829700	0.10707400	-1.21053500
O	-2.88815600	-0.71464400	-0.56545400
H	-3.19262000	-1.42266600	0.02291800
H	-1.21889600	-2.37723600	0.59630000
F	2.86246500	-0.90890400	0.03899500

1-OH-4-CHO-substituted

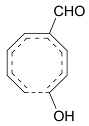

C	-1.60872800	0.93694800	1.07427600
C	-1.15386100	1.94307100	0.24522600
C	0.13468800	1.98632700	-0.31597400
C	1.14407300	1.03116700	-0.32692700
C	1.28732200	-0.33426600	0.04128100
C	0.41082400	-1.40146700	0.29391000
C	-0.96259900	-1.55457700	0.13013600
C	-1.80900400	-0.62260400	-0.47117800
H	-0.89094700	0.36695700	1.65000200
H	-2.62075700	0.98070500	1.46849000
H	-1.85632200	2.71121600	-0.07552400
H	0.39537000	2.91615300	-0.81794600
H	2.09637600	1.42284300	-0.68398300
H	0.90709700	-2.29465800	0.67425200
H	-1.42511100	0.06004800	-1.21652200
O	-3.12974900	-0.86064700	-0.65151200

H	-3.41981200	-1.59088200	-0.08258600
H	-1.41968800	-2.45014000	0.55253500
C	2.71869500	-0.76761200	0.11266300
H	2.86775900	-1.81023000	0.46282300
O	3.67944600	-0.09109300	-0.17749000

1-OH-4-CN-substituted

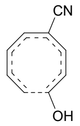

C	-1.58225400	0.84693700	1.10438100
C	-1.26741100	1.88276700	0.25030300
C	-0.01156900	2.05489700	-0.35944100
C	1.09386600	1.21415300	-0.40302800
C	1.38126800	-0.13424800	-0.03356600
C	0.63291400	-1.28924600	0.25091000
C	-0.72573300	-1.57232200	0.12962300
C	-1.68307400	-0.74770000	-0.45511000
H	-0.78819600	0.34996000	1.64680200
H	-2.57776200	0.78538700	1.53610400
H	-2.05657800	2.57119100	-0.04802200
H	0.13358800	3.00120300	-0.87619900
H	1.97859800	1.70925300	-0.80025300
H	1.23187000	-2.12003600	0.61608800
H	-1.40341400	-0.02337600	-1.20771700
O	-2.97613300	-1.12155400	-0.58592000
H	-3.16764400	-1.87905200	-0.01086000
H	-1.07356700	-2.50497700	0.57482300
C	2.80922000	-0.38772000	0.00744700
N	3.94983000	-0.58889000	0.04392500

1-OH-4-NO_2-substituted

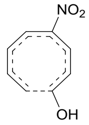

C	-1.96364000	0.74711700	1.15865800
C	-1.67775700	1.84711500	0.37712100
C	-0.43025800	2.09908300	-0.22099800
C	0.70593300	1.30533600	-0.32550100
C	1.02594900	-0.04115700	-0.03721000
C	0.33594900	-1.23674700	0.16502800
C	-1.01008600	-1.55418100	0.01254200
C	-1.99976900	-0.72563600	-0.51378000
H	-1.15857900	0.23775200	1.67212700
H	-2.95806200	0.63016400	1.58130900
H	-2.48756100	2.53031600	0.12540600
H	-0.32068200	3.08026400	-0.67755300
H	1.57091500	1.84437700	-0.69846500
H	0.96709100	-2.05712100	0.48745100
H	-1.74867000	0.06081700	-1.21191700
O	-3.27403100	-1.14272400	-0.67826200
H	-3.43445000	-1.95017000	-0.16467700
H	-1.32479200	-2.52835600	0.38751300

N	2.52237200	-0.26050700	-0.00189200
O	3.24655200	0.58209800	-0.50134700
O	2.94251000	-1.27313300	0.53172100

1-F-4-CH_3-substituted

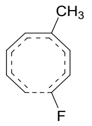

C	-1.30379300	0.97354200	1.09705000
C	-0.86603000	1.95968400	0.24505500
C	0.39873300	1.96315100	-0.37850600
C	1.40298900	1.00503200	-0.42630300
C	1.60970300	-0.34881500	-0.01246300
C	0.74070100	-1.39956700	0.26817200
C	-0.65193200	-1.55948000	0.12742000
C	-1.50643400	-0.66177200	-0.45669400
H	-0.57874900	0.36836300	1.62628800
H	-2.30248100	1.02003500	1.52369200
H	-1.56267200	2.74008900	-0.05698500
H	0.64332100	2.87833300	-0.91550000
H	2.31724500	1.41474700	-0.86036600
H	1.23174300	-2.29116700	0.65273000
H	-1.21808500	0.04787200	-1.21953900
F	-2.81792400	-0.97838800	-0.52016800
H	-1.10990400	-2.43235900	0.58814900
C	3.09298300	-0.69745500	0.05901300
H	3.25469000	-1.72301400	0.39855900
H	3.55633700	-0.59793200	-0.92993700
H	3.62834500	-0.02538400	0.73796000

1-F-4-NH_2-substituted

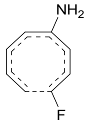

C	-1.25123800	0.99617200	1.10474000
C	-0.78771100	1.98589800	0.27057200
C	0.46483500	1.95124800	-0.37829900
C	1.43543500	0.96616100	-0.46995900
C	1.61562200	-0.38469000	-0.02254800
C	0.72372000	-1.41313100	0.28328600
C	-0.66607500	-1.54588700	0.11196800
C	-1.51009400	-0.63144000	-0.46112500
H	-0.54285700	0.35606700	1.61605000
H	-2.24368600	1.06947300	1.54233300
H	-1.45990300	2.79390400	-0.01197000
H	0.71948200	2.85545200	-0.92903500
H	2.33873300	1.31388200	-0.97151800
H	1.19537000	-2.29958600	0.70431800
H	-1.22068100	0.08128900	-1.22057600
F	-2.82888400	-0.93214500	-0.51485600
H	-1.14157400	-2.40793100	0.57576400
N	2.98586700	-0.73895600	-0.00952800
H	3.17671200	-1.67872900	0.31418900

H	3.59032300	-0.06781100	0.44904100

1-F-4-OH-substituted

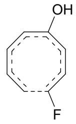

C	-1.23307800	1.02743500	1.08540600
C	-0.75801600	1.99610900	0.23612000
C	0.51542100	1.95793900	-0.37198100
C	1.48248900	0.96859200	-0.42214600
C	1.60807700	-0.39309500	-0.01194400
C	0.72011100	-1.42217200	0.27721300
C	-0.67526400	-1.54110500	0.12767200
C	-1.50770500	-0.63206300	-0.46562400
H	-0.53295000	0.39160400	1.61341000
H	-2.23206600	1.10628600	1.50622100
H	-1.42376100	2.80021800	-0.07174800
H	0.79915600	2.86475300	-0.90341500
H	2.42777900	1.30590500	-0.84355900
H	1.19347600	-2.31380900	0.68747800
H	-1.20982700	0.06920800	-1.23232100
F	-2.82927000	-0.91332100	-0.51085900
H	-1.15576400	-2.39166400	0.60665000
O	2.95252700	-0.71358700	0.05525600
H	3.06496300	-1.67375300	0.06466800

1-F-4-F-substituted

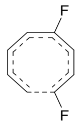

C	-1.17471000	1.07334500	1.07807800
C	-0.67716500	2.01619500	0.21479500
C	0.60376500	1.94825900	-0.37746800
C	1.53487500	0.92506900	-0.39981500
C	1.56222900	-0.44072400	-0.01325000
C	0.67930700	-1.46744600	0.25009500
C	-0.72263200	-1.54304600	0.13268800
C	-1.52258500	-0.60693500	-0.46156900
H	-0.49223900	0.41955100	1.60750300
H	-2.17115600	1.18036300	1.49792900
H	-1.32182900	2.83404900	-0.10163800
H	0.92619900	2.84729900	-0.89917900
H	2.50968300	1.21665200	-0.78699100
H	1.17385300	-2.36826400	0.60361800
H	-1.20047200	0.08071700	-1.23091800
F	-2.85270400	-0.82690300	-0.49122300
H	-1.22392000	-2.38052700	0.61232800
F	2.86396800	-0.86844700	0.06411500

1-F-4-CHO-substituted

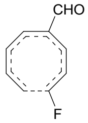

C	-1.63178100	0.97618700	1.08797000
C	-1.17768200	1.95138500	0.23507400
C	0.11611500	1.97931500	-0.33424000
C	1.11935400	1.02655300	-0.35353400
C	1.27733700	-0.33828600	0.03978600
C	0.42262200	-1.41012300	0.29479900
C	-0.96133100	-1.57877100	0.13581800
C	-1.78494700	-0.66887600	-0.47943300
H	-0.92191600	0.37827400	1.64491700
H	-2.64604200	1.01433200	1.47639300
H	-1.87469500	2.71897900	-0.09806300
H	0.37710200	2.90665000	-0.84099800
H	2.06499100	1.41372800	-0.73176800
H	0.92780000	-2.29742900	0.67649200
H	-1.45552300	0.03438900	-1.23189900
F	-3.09301200	-0.95921200	-0.58047400
H	-1.43342800	-2.45267300	0.57805800
C	2.71847800	-0.74676600	0.13208100
H	2.88235700	-1.77993800	0.50123700
O	3.66593500	-0.05589000	-0.16250300

1-F-4-CN-substituted

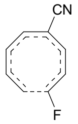

C	-1.60686300	0.88297700	1.11886400
C	-1.28971000	1.88875100	0.24200300
C	-0.02861000	2.04450400	-0.37838900
C	1.06945700	1.20489700	-0.43257000
C	1.37176100	-0.14231400	-0.03913600
C	0.64315100	-1.29834100	0.24608700
C	-0.72459700	-1.59496300	0.13097500
C	-1.66147300	-0.78897700	-0.46221000
H	-0.81892300	0.35665900	1.64236600
H	-2.60323600	0.81928500	1.54782400
H	-2.07279200	2.57945500	-0.06639700
H	0.11643400	2.98802400	-0.90068300
H	1.94656900	1.69228400	-0.85496100
H	1.24881600	-2.12426600	0.61049500
H	-1.44634800	-0.05092200	-1.22213000
F	-2.93288800	-1.21816100	-0.50692300
H	-1.08915400	-2.50913200	0.59325300
C	2.80500100	-0.36673000	0.02269900
N	3.94941900	-0.53811000	0.07465700

1-F-4-NO_2-substituted

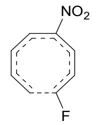

C	-1.98455700	0.76605100	1.18319400
C	-1.69986900	1.84693800	0.38831700
C	-0.44710800	2.09311900	-0.21917400
C	0.68081500	1.30149500	-0.34501700
C	1.01309500	-0.04744900	-0.04334600
C	0.35090500	-1.24801600	0.15190800
C	-1.00305000	-1.58495400	-0.00195900
C	-1.96734000	-0.76908800	-0.53516900
H	-1.18225000	0.22432900	1.66732000
H	-2.97886100	0.63857800	1.60254700
H	-2.50554100	2.53437200	0.13571000
H	-0.33825000	3.07751600	-0.66937800
H	1.53853300	1.83508300	-0.74256600
H	0.99319900	-2.06083300	0.47299500
H	-1.77510500	0.03627000	-1.23044800
F	-3.22007700	-1.24058200	-0.62545400
H	-1.33423300	-2.54613200	0.38294500
N	2.51429300	-0.24047800	0.00859400
O	2.94708800	-1.15769800	0.68249300
O	3.21613700	0.52780200	-0.62283400

1-CHO-4-CH_3-substituted

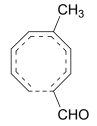

C	-0.85987900	1.33177000	1.03271400
C	-0.15097500	2.20054000	0.23427700
C	1.11886500	1.93394200	-0.31224000
C	1.90657200	0.78683100	-0.36190900
C	1.83321400	-0.59553600	-0.01195100
C	0.77383500	-1.47124300	0.24418200
C	-0.61360600	-1.33427000	0.09507700
C	-1.26179500	-0.28216000	-0.52283000
H	-0.33926900	0.55933300	1.58326400
H	-1.83920100	1.61161700	1.41313400
H	-0.62458200	3.13175800	-0.07177600
H	1.58751800	2.79067600	-0.79461500
H	2.90229400	1.02418300	-0.74102200
H	1.08474800	-2.44481500	0.61825400
H	-0.72756300	0.32634200	-1.24220800
C	-2.73911600	-0.25659200	-0.58652400
H	-3.17605400	0.48543100	-1.28401400
O	-3.46386100	-0.96287300	0.08354600
H	-1.24266900	-2.09216100	0.56101400
C	3.21761900	-1.23262300	0.04557400
H	3.68616800	-1.20784100	-0.94555900
H	3.87675700	-0.68994900	0.73102800
H	3.17433500	-2.27554100	0.36590900

1-CHO-4-NH_2-substituted

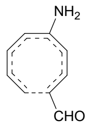

C	-0.80086800	1.33342500	1.03066100
C	-0.06029100	2.20554800	0.26359200
C	1.19319100	1.90348400	-0.30149400
C	1.94064500	0.73664700	-0.39814100
C	1.83167200	-0.64099500	-0.02194000
C	0.74229600	-1.48630000	0.26174700
C	-0.62819100	-1.31790700	0.07661200
C	-1.26192500	-0.24485200	-0.53259800
H	-0.30373700	0.53648200	1.56876000
H	-1.77188000	1.63432100	1.41632200
H	-0.50247600	3.15777200	-0.02208500
H	1.67797800	2.74779900	-0.78984500
H	2.91897800	0.91937900	-0.84270100
H	1.03038600	-2.45030800	0.67908000
H	-0.72460800	0.35692100	-1.25547800
C	-2.73336700	-0.20104700	-0.58264700
H	-3.16613200	0.55492300	-1.26822000
O	-3.46659100	-0.90320200	0.08663300
H	-1.27834100	-2.06280100	0.53509500
N	3.07990400	-1.27493300	-0.01717800
H	3.09398500	-2.23573800	0.29661000
H	3.86028100	-0.73661700	0.33489600

1-CHO-4-OH-substituted

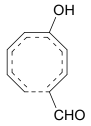

C	-0.77236100	1.36585800	1.01955800
C	-0.02650800	2.20793600	0.22864900
C	1.24030700	1.89260100	-0.30118700
C	1.97932100	0.71909700	-0.35560800
C	1.81807700	-0.65413400	-0.01253300
C	0.73730500	-1.50024400	0.25954500
C	-0.63785800	-1.31651500	0.09517900
C	-1.25359300	-0.25032100	-0.53449400
H	-0.28488500	0.57129400	1.57003400
H	-1.74392800	1.67948600	1.39279400
H	-0.46019000	3.15609100	-0.08282200
H	1.74990700	2.72945600	-0.77625600
H	2.99136400	0.87198300	-0.72554000
H	1.02631900	-2.46920700	0.66751800
H	-0.70442800	0.33867600	-1.25905100
C	-2.72624800	-0.18254500	-0.58653600
H	-3.14699300	0.56913700	-1.28350000
O	-3.46756800	-0.86610200	0.09109300
H	-1.29339800	-2.04617200	0.56963900
O	3.06047100	-1.24437600	0.03313100
H	2.97235000	-2.20731600	0.05795800

1-CHO-4-F-substituted

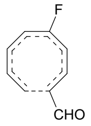

C	-0.72117300	1.38519500	1.02300900
C	0.02501100	2.20075300	0.20925200
C	1.29294600	1.86830100	-0.31319800
C	2.00521400	0.67927100	-0.34097500
C	1.77329000	-0.68046900	-0.01263900
C	0.71579100	-1.54301300	0.22525900
C	-0.66396300	-1.33340600	0.09012200
C	-1.25321100	-0.25539700	-0.53574900
H	-0.24513200	0.57972400	1.56789500
H	-1.68597900	1.71457000	1.39942000
H	-0.39824000	3.15053600	-0.11170300
H	1.82267500	2.69367700	-0.78540900
H	3.03189600	0.79468800	-0.68349400
H	1.03761200	-2.52034100	0.57557200
H	-0.68966900	0.32516200	-1.25628700
C	-2.72652800	-0.13827400	-0.57728200
H	-3.12879000	0.62154400	-1.27574100
O	-3.48003300	-0.79590200	0.10978700
H	-1.32792400	-2.05320800	0.56691200
F	2.97106200	-1.33743300	0.05752800

1-CHO-4-CHO-substituted

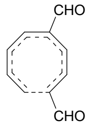

C	-1.24355600	1.32828100	1.03928200
C	-0.58401800	2.21483500	0.22354900
C	0.72124000	2.03547100	-0.28611400
C	1.57341400	0.94159800	-0.30619700
C	1.53523100	-0.44276900	0.03542300
C	0.54589000	-1.39893500	0.27255900
C	-0.84705000	-1.35421500	0.11385300
C	-1.52784300	-0.34252000	-0.52970100
H	-0.68799100	0.59051800	1.60303300
H	-2.24577700	1.55012700	1.39715100
H	-1.11497100	3.10535300	-0.10931700
H	1.14891100	2.92298700	-0.74976200
H	2.57578000	1.20582600	-0.64198200
H	0.92803600	-2.35040100	0.64298200
H	-1.00320800	0.28993000	-1.23564200
C	-3.00822900	-0.37758400	-0.62160600
H	-3.46381800	0.33784500	-1.33332300
O	-3.70714300	-1.11108300	0.04322000
H	-1.43906000	-2.14292300	0.57560600
C	2.91399500	-1.04687400	0.11888500
H	2.93638700	-2.09894600	0.46715100
O	3.94355200	-0.48317200	-0.16515700

1-CHO-4-CN-substituted

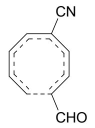

C	-1.20892500	1.29365000	1.06814600
C	-0.63990200	2.22178500	0.23438900
C	0.65640200	2.13079800	-0.32189900
C	1.58122200	1.10074400	-0.37945500
C	1.63763600	-0.29162800	-0.03833400
C	0.72470300	-1.31762400	0.22109700
C	-0.67297600	-1.35102900	0.09352100
C	-1.43561500	-0.39239100	-0.53457200
H	-0.58943900	0.58235700	1.59839600
H	-2.21106700	1.44410400	1.46098400
H	-1.23725900	3.07765200	-0.07473100
H	1.00805400	3.04461600	-0.79742300
H	2.54383300	1.44132100	-0.75630500
H	1.18075000	-2.23843100	0.57728700
H	-0.97718100	0.28429900	-1.24545100
C	-2.91258900	-0.52716900	-0.58540400
H	-3.43457800	0.15371100	-1.28508200
O	-3.54134800	-1.30521400	0.09772100
H	-1.20044800	-2.17517300	0.57117400
C	3.01373300	-0.75645400	0.02195600
N	4.11228600	-1.11954700	0.07038700

1-CHO-4-NO_2-substituted

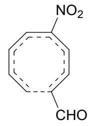

C	-1.62272400	1.30727200	1.06129600
C	-1.10454100	2.24921000	0.21052400
C	0.19272000	2.22059400	-0.35080700
C	1.17655200	1.24700400	-0.39513700
C	1.29015300	-0.12811900	-0.05416200
C	0.45484300	-1.20389800	0.19970400
C	-0.94218200	-1.30585700	0.09164900
C	-1.76391200	-0.39720300	-0.53444400
H	-0.96836300	0.63310100	1.59809800
H	-2.62992800	1.41377900	1.45551700
H	-1.74736600	3.06741000	-0.10954800
H	0.48767500	3.14313200	-0.84708900
H	2.11970800	1.63126800	-0.76993500
H	0.96884600	-2.09567700	0.54225800
H	-1.35284600	0.29921000	-1.25490400
C	-3.23277300	-0.61748000	-0.56678700
H	-3.80153800	0.02343500	-1.26741500
O	-3.80573500	-1.42297200	0.13244600
H	-1.41869100	-2.15040800	0.58653300
N	2.75224900	-0.53778100	0.02178500
O	3.58416500	0.34321300	0.13973600
O	3.02006400	-1.72398000	-0.02931000

1-CN-4-CH_3-substituted

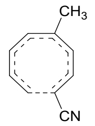

C	-0.92063700	1.19481200	1.12198100
C	-0.37241200	2.10673600	0.24689200
C	0.86991800	1.94066600	-0.39153700
C	1.75444700	0.86645800	-0.45421800
C	1.82500200	-0.49818200	-0.04251000
C	0.86427300	-1.45030400	0.31118300
C	-0.53575600	-1.45264000	0.23911100
C	-1.30715900	-0.46629400	-0.35316800
H	-0.27014300	0.51058300	1.65219100
H	-1.89446400	1.37910100	1.56790000
H	-0.96393100	2.97092100	-0.04864100
H	1.21576600	2.81306700	-0.94406200
H	2.69709100	1.17057800	-0.91278400
H	1.27974200	-2.37786800	0.69891500
H	-0.87183000	0.15870700	-1.12206100
C	-2.73726400	-0.59743500	-0.39590500
N	-3.89336300	-0.67725100	-0.41396700
H	-1.05166300	-2.26164000	0.75203300
C	3.26110800	-1.01180000	-0.04497400
H	3.66949900	-0.98648900	-1.06215000
H	3.90625300	-0.38754100	0.58145200
H	3.32809900	-2.04075700	0.31384000

1-CN-4-NH_2-substituted

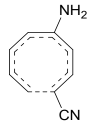

C	-0.87393500	1.19279400	1.11884300
C	-0.29767000	2.11783000	0.27462600
C	0.93238900	1.92405100	-0.37948200
C	1.78312200	0.83066100	-0.48888700
C	1.83113900	-0.53258500	-0.05376200
C	0.84701000	-1.46425500	0.32502500
C	-0.54286200	-1.44443600	0.21744500
C	-1.30573900	-0.43852200	-0.36299500
H	-0.24059000	0.48350100	1.63791800
H	-1.84332100	1.39205600	1.56830700
H	-0.86474600	3.00354600	-0.00244000
H	1.28924600	2.78952200	-0.93605800
H	2.70622200	1.08396200	-1.00997800
H	1.24883000	-2.38220700	0.75035200
H	-0.86864800	0.18322800	-1.13385200
C	-2.73512400	-0.55552700	-0.39580500
N	-3.89286400	-0.62204100	-0.40128100
H	-1.07384500	-2.24722300	0.72516500
N	3.13316100	-1.04557400	-0.10570200
H	3.25915500	-1.98937200	0.23366700
H	3.87562900	-0.42378100	0.18575000

1-CN-4-OH-substituted

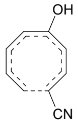

C	-0.85188700	1.22034700	1.10634100
C	-0.27210300	2.12084100	0.24143700
C	0.97525400	1.92263300	-0.37935000
C	1.82454200	0.82602400	-0.44832900
C	1.82069100	-0.54005200	-0.04679800
C	0.84630200	-1.47548800	0.31883300
C	-0.54818100	-1.44375300	0.23381300
C	-1.29904100	-0.44476000	-0.36527600
H	-0.22432300	0.51446100	1.63691700
H	-1.82435000	1.42748800	1.54497900
H	-0.83556400	3.00190500	-0.05747200
H	1.35664700	2.78641100	-0.92153100
H	2.78946900	1.05993100	-0.89401300
H	1.25170600	-2.39764800	0.73427500
H	-0.85407800	0.16778900	-1.13893800
C	-2.73013400	-0.54183800	-0.39359900
N	-3.88823400	-0.59457600	-0.39808900
H	-1.08192300	-2.23487300	0.75613000
O	3.11522000	-1.00677400	-0.05788500
H	3.12563000	-1.97297000	-0.01308100

1-CN-4-F-substituted

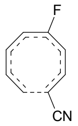

C	-0.80777900	1.23778900	1.10585900
C	-0.22643700	2.11645600	0.22305300
C	1.02620100	1.90790900	-0.38757500
C	1.85562000	0.79635300	-0.43049100
C	1.77988400	-0.56467200	-0.04590000
C	0.82846100	-1.51725900	0.28384300
C	-0.57055200	-1.46228500	0.22820600
C	-1.30062700	-0.45067400	-0.36786100
H	-0.18948600	0.52059600	1.63222400
H	-1.77657800	1.45784600	1.54572200
H	-0.78266000	2.99953700	-0.08372600
H	1.42850900	2.76587300	-0.92300700
H	2.84116700	0.99893400	-0.84566700
H	1.26513700	-2.44695800	0.63844800
H	-0.84525200	0.15768100	-1.13880800
C	-2.73485500	-0.50323100	-0.38186900
N	-3.89359400	-0.52003600	-0.37653200
H	-1.11200500	-2.24765000	0.75042200
F	3.03631600	-1.10310200	-0.03371800

1-CN-4-CHO-substituted

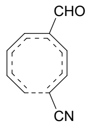

C	-1.29020000	1.19819600	1.12814800
C	-0.75847800	2.10526400	0.24236400
C	0.51951100	1.99712700	-0.34591400
C	1.44057600	0.95968600	-0.37004100
C	1.50674300	-0.40546400	0.03454000
C	0.59285700	-1.40970800	0.35989500
C	-0.80435100	-1.46928800	0.26512500
C	-1.57739500	-0.51019200	-0.36419100
H	-0.63338600	0.54036100	1.68297300
H	-2.28472100	1.34907000	1.53902300
H	-1.37958900	2.93601300	-0.08756500
H	0.85301300	2.88687000	-0.87722900
H	2.40410900	1.26836900	-0.77469200
H	1.05071400	-2.32004400	0.74657300
H	-1.12910100	0.12592300	-1.11652100
C	-3.00352500	-0.66533300	-0.44741000
N	-4.15637800	-0.76625100	-0.50067300
H	-1.30777000	-2.29175400	0.76753500
C	2.92369900	-0.92093300	0.07221900
H	3.02960100	-1.95353600	0.46106900
O	3.89939600	-0.30670500	-0.28560800

1-CN-4-CN-substituted

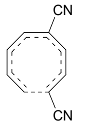

C	-1.25035200	1.16877200	1.14408700
C	-0.81451900	2.10302500	0.23721900
C	0.44594900	2.08057100	-0.39692900
C	1.43881500	1.11419900	-0.45475200
C	1.60464400	-0.24944200	-0.04660100
C	0.77517700	-1.31868200	0.30732000
C	-0.61876000	-1.46120800	0.24826600
C	-1.47321000	-0.56552000	-0.36495800
H	-0.53467400	0.54980800	1.67025700
H	-2.23912200	1.25206100	1.58674600
H	-1.50127000	2.88770700	-0.07415900
H	0.69819200	2.98665000	-0.94425500
H	2.35514800	1.49592700	-0.90116400
H	1.30826400	-2.18986300	0.67990400
H	-1.09425500	0.10564000	-1.12465800
C	-2.88701800	-0.81545600	-0.40070900
N	-4.03173000	-0.99229900	-0.41468100
H	-1.05076800	-2.31266300	0.76860000
C	3.00888400	-0.62410700	-0.03578700
N	4.12899000	-0.91744000	-0.02592000

1-CN-4-NO_2-substituted

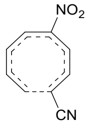

C	-1.65368100	1.17601600	1.14659000
C	-1.25354200	2.12059200	0.23413300
C	0.00743000	2.14771000	-0.39924400
C	1.04426200	1.22876900	-0.44860900
C	1.24656900	-0.11852400	-0.05007700
C	0.48104400	-1.22562500	0.28284900
C	-0.90785900	-1.41777800	0.23544900
C	-1.80470400	-0.55983000	-0.37018800
H	-0.91626600	0.58583900	1.67566300
H	-2.64536500	1.22392300	1.58803500
H	-1.97137200	2.87493900	-0.08239600
H	0.21825900	3.05885800	-0.95524600
H	1.94677900	1.64443100	-0.88497300
H	1.05663400	-2.07424000	0.63608900
H	-1.46249900	0.12683000	-1.13359000
C	-3.20665800	-0.87289100	-0.39426900
N	-4.34189000	-1.10256000	-0.39860600
H	-1.30044600	-2.28324200	0.76365100
N	2.73157100	-0.45000600	-0.02142900
O	3.52006000	0.47683700	-0.01592900
O	3.05860700	-1.62183800	0.00508000

1-NO_2-4-CH_3-substituted

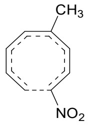

C	-0.51432100	1.21267000	1.26575100
C	0.05518400	2.14376600	0.43261400
C	1.27874500	1.95985800	-0.24551400
C	2.10609600	0.85542900	-0.40638400
C	2.12240000	-0.54021100	-0.08947900
C	1.12557200	-1.47107500	0.20357100
C	-0.27656700	-1.39464200	0.16654100
C	-0.98846700	-0.33321100	-0.33108700
H	0.10536000	0.45437700	1.72848700
H	-1.47457800	1.40687000	1.73772400
H	-0.50276300	3.04727900	0.19633600
H	1.65499600	2.85147200	-0.74495000
H	3.05044500	1.14483100	-0.87053900
H	1.49957900	-2.44556900	0.50850900
H	-0.62652000	0.33629200	-1.09521800
N	-2.44338400	-0.38365100	-0.30242100
O	-3.02652500	0.37137100	-1.06568900
O	-2.98973600	-1.13463300	0.49108400
H	-0.84228000	-2.19582300	0.63624500
C	3.53501700	-1.10949600	-0.14316800
H	3.93482900	-1.03079000	-1.16101400
H	4.20982000	-0.55378800	0.51586000
H	3.56293200	-2.16202600	0.14528000

1-NO_2-4-NH_2-substituted

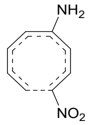

C	-0.47568100	1.19616800	1.25732500
C	0.12188900	2.14336600	0.46090400
C	1.33373100	1.93968900	-0.23143000
C	2.13075000	0.82458900	-0.43934600
C	2.12916700	-0.57132800	-0.10210600
C	1.11045300	-1.48846800	0.21531000
C	-0.27800600	-1.39223200	0.14096500
C	-0.98634100	-0.31240300	-0.34090300
H	0.12740100	0.41808500	1.71053400
H	-1.43427700	1.39940300	1.72924000
H	-0.41368700	3.06459100	0.24475000
H	1.71828100	2.82634300	-0.73353200
H	3.05156400	1.07451300	-0.96581100
H	1.47524500	-2.45435200	0.55911200
H	-0.62466100	0.35664300	-1.10557800
N	-2.43345300	-0.35327100	-0.30030800
O	-3.02146900	0.44706000	-1.01504000
O	-2.98322400	-1.13774100	0.46167100
H	-0.85820000	-2.19137100	0.59679000
N	3.40339400	-1.12652800	-0.20154100
H	3.50604700	-2.09673400	0.06141000
H	4.18447000	-0.54936800	0.07867100

1-NO_2-4-OH-substituted

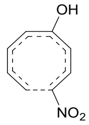

C	-0.45198500	1.22749000	1.24674900
C	0.14772800	2.14719100	0.42376900
C	1.37771300	1.93396800	-0.23500000
C	2.17311300	0.81190500	-0.40048300
C	2.11732800	-0.58196100	-0.09591100
C	1.10805400	-1.50028300	0.20745600
C	-0.28538200	-1.39249800	0.15996700
C	-0.98109400	-0.32052200	-0.34353900
H	0.14461100	0.45187600	1.71247700
H	-1.41243200	1.44224400	1.70927800
H	-0.38408700	3.06467500	0.18305800
H	1.78850200	2.81622900	-0.72317400
H	3.13810000	1.03387400	-0.85176500
H	1.47427900	-2.47116500	0.53899000
H	-0.61262600	0.33952500	-1.11275300
N	-2.43134300	-0.34181700	-0.29719400
O	-3.00937600	0.44513700	-1.03271500
O	-2.98560700	-1.10041900	0.48558100
H	-0.86720600	-2.17839300	0.63589100
O	3.38899200	-1.09426300	-0.15691700
H	3.36534400	-2.06152200	-0.15728400

1-NO_2-4-F-substituted

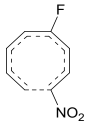

C	-0.41006600	1.24115700	1.24929900
C	0.18730100	2.13887200	0.40466700
C	1.42313800	1.91624600	-0.24495200
C	2.20005600	0.77991500	-0.38615100
C	2.07569800	-0.60541800	-0.09575000
C	1.09179900	-1.54024300	0.17173300
C	-0.30659000	-1.41529900	0.15370800
C	-0.98065900	-0.33592900	-0.35048700
H	0.17720800	0.45548000	1.71010300
H	-1.36668300	1.46768300	1.71359800
H	-0.33896900	3.05732900	0.15506200
H	1.85269100	2.79227300	-0.72729900
H	3.18369100	0.96938400	-0.81178600
H	1.49108300	-2.51357900	0.44275200
H	-0.60069000	0.32433400	-1.11401900
N	-2.43386800	-0.31461400	-0.28870500
O	-2.99367700	0.48300300	-1.02520100
O	-2.99860400	-1.05292800	0.50339800
H	-0.89375400	-2.19371300	0.63475500
F	3.30963100	-1.18592200	-0.13557900

1-NO_2-4-CHO-substituted

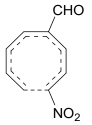

C	-0.87594400	1.24892400	1.28079000
C	-0.32786900	2.16844900	0.42612400
C	0.93555200	2.03596800	-0.19982300
C	1.80496000	0.96543100	-0.31527000
C	1.82017900	-0.42789100	0.00362300
C	0.87214100	-1.41095500	0.27149600
C	-0.53306400	-1.39603100	0.21105900
C	-1.25282700	-0.36879200	-0.33397300
H	-0.24533600	0.51729000	1.77061200
H	-1.85744900	1.41451300	1.71817300
H	-0.91892600	3.03888100	0.14840100
H	1.29813100	2.94236800	-0.68175400
H	2.77095400	1.25520700	-0.72772500
H	1.29294800	-2.36741700	0.58032000
H	-0.88047400	0.30798700	-1.08681600
N	-2.71069300	-0.44928600	-0.34650900
O	-3.28206900	0.27564900	-1.14441500
O	-3.25986400	-1.19803600	0.44469100
H	-1.08281100	-2.20788800	0.68024500
C	3.21708400	-1.00041300	-0.00831400
H	3.28577100	-2.05894400	0.31244900
O	4.21078000	-0.40075400	-0.33810400

1-NO_2-4-CN-substituted

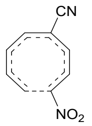

C	-0.83090300	1.24814700	1.28381200
C	-0.36307400	2.19275100	0.41088000
C	0.88509200	2.13225600	-0.25490800
C	1.81648600	1.11957700	-0.39868200
C	1.91625700	-0.27678000	-0.07350100
C	1.03720000	-1.31828900	0.22011500
C	-0.36821100	-1.37070000	0.18950900
C	-1.16124600	-0.39392700	-0.34321000
H	-0.14936000	0.54836800	1.75164700
H	-1.80775000	1.35971300	1.74776300
H	-1.00965400	3.02791600	0.14987200
H	1.17758300	3.05452900	-0.75330200
H	2.74128800	1.47424800	-0.84987100
H	1.52259500	-2.24340800	0.51980200
H	-0.85260300	0.30852200	-1.10160800
N	-2.61175100	-0.55906200	-0.31101400
O	-3.24898300	0.14439800	-1.07742200
O	-3.09051700	-1.35042300	0.48388600
H	-0.86004800	-2.20925100	0.67570300
C	3.30062700	-0.71951400	-0.09966700
N	4.40612000	-1.06287600	-0.11724400

1-NO_2-4-NO_2-substituted

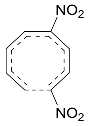

C	-1.24801200	1.18844600	1.36914700
C	-0.81691600	2.20431200	0.55988200
C	0.43807800	2.24502600	-0.09434600
C	1.41812000	1.28954400	-0.29657900
C	1.56177100	-0.10345000	-0.04862600
C	0.75953500	-1.20760100	0.16740500
C	-0.64207400	-1.32930800	0.10789600
C	-1.47929500	-0.36437100	-0.37314100
H	-0.54105000	0.48971800	1.79902100
H	-2.23348100	1.22677600	1.82677800
H	-1.50005900	3.02333700	0.34440800
H	0.68899300	3.20968700	-0.53103500
H	2.32589800	1.69767300	-0.73012200
H	1.29928700	-2.11063800	0.43107200
H	-1.20195500	0.40475800	-1.07698300
N	-2.92004500	-0.60835000	-0.37270600
O	-3.58441800	0.10494600	-1.10579400
O	-3.36430400	-1.47011000	0.36641100
H	-1.09302300	-2.22252000	0.53241400
N	3.02742300	-0.50600000	-0.09385400
O	3.37397300	-1.44133600	0.60076100
O	3.76931100	0.12475900	-0.82130900

5.3.5 1,5-substituted

1-CH_3-5-CH_3-substituted

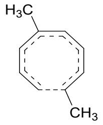

C	0.91485300	-1.18184100	1.12008100
C	-0.00231500	-1.85691800	0.34929900
C	-1.21763600	-1.32637000	-0.13387300
C	-1.73879200	-0.03509500	-0.11902600
C	-1.19477200	1.23703500	0.24706800
C	0.05010600	1.83547900	0.40367300
C	1.35995000	1.38095400	0.15456100
C	1.67942000	0.20971200	-0.49956500
H	0.59783100	-0.32773100	1.70318100
H	1.81948600	-1.68499600	1.45310700
H	0.25079300	-2.85898200	0.00435300
H	-1.87258600	-2.06692500	-0.59041700
H	-1.99607100	1.96268700	0.40018100
H	-0.00721000	2.85042400	0.79504400
H	0.92171900	-0.21009100	-1.15239100
C	3.10007400	-0.18891600	-0.78790000
H	3.78192300	0.18727600	-0.01878600
H	3.20596800	-1.27659100	-0.84361400
H	3.42180100	0.21915300	-1.75444700
H	2.17196100	1.96859800	0.58509700
C	-3.20597500	0.07636100	-0.52219600
H	-3.81246400	0.39601500	0.33396500
H	-3.34746700	0.81717900	-1.31603200
H	-3.60516700	-0.87841500	-0.87197700

1-CH_3-5-NH_2-substituted

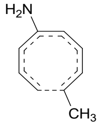

C	0.87846200	-1.20659000	1.15440000
C	-0.03909800	-1.85498500	0.36248300
C	-1.22236800	-1.31235900	-0.17473400
C	-1.74924200	-0.01963500	-0.13862100
C	-1.21274000	1.24577600	0.27351700
C	0.03578100	1.83518100	0.40834400
C	1.34540000	1.38477200	0.14476500
C	1.66231000	0.20826400	-0.49753900
H	0.58592300	-0.33583100	1.72551500
H	1.76705500	-1.73268800	1.49370300
H	0.20686000	-2.85941900	0.01831500
H	-1.85531100	-2.02208100	-0.70601800
H	-2.01395600	1.95581500	0.48296300
H	-0.01649300	2.84236600	0.82003000
H	0.89789900	-0.23232900	-1.12868200
C	3.08095900	-0.19097400	-0.78952000
H	3.76816000	0.20353100	-0.03461000
H	3.18963600	-1.27904300	-0.82420500
H	3.39195400	0.19893800	-1.76725300

H	2.15739900	1.97883300	0.56519200
N	-3.07828100	0.06387200	-0.61053600
H	-3.51229500	0.97352700	-0.52988400
H	-3.69564900	-0.67541900	-0.29988000

1-CH_3-5-OH-substituted

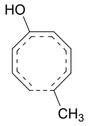

C	-0.87183100	1.21137500	1.13593000
C	0.04468200	1.87032800	0.35228500
C	1.24334900	1.33312500	-0.15813800
C	1.73673000	0.03584700	-0.13795800
C	1.22759300	-1.23773800	0.26571200
C	-0.01301600	-1.83808700	0.42124400
C	-1.32361500	-1.38942600	0.16262000
C	-1.64049400	-0.21958800	-0.49309800
H	-0.56796400	0.35149200	1.71760600
H	-1.77079800	1.72548400	1.46613800
H	-0.20390200	2.87222200	0.00413100
H	1.91738300	2.03117300	-0.64868800
H	2.04766400	-1.92856200	0.47106600
H	0.04881900	-2.84257000	0.83725900
H	-0.87708700	0.21052300	-1.13305900
C	-3.05915900	0.17620200	-0.79038300
H	-3.74548600	-0.20205400	-0.02650300
H	-3.16636900	1.26340400	-0.84710900
H	-3.37215200	-0.23310600	-1.75937900
H	-2.13466000	-1.97882100	0.59098800
O	3.05942500	-0.00321900	-0.54735400
H	3.28372900	-0.89566400	-0.84289700

1-CH_3-5-F-substituted

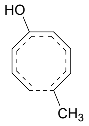

C	0.83318800	-1.23380100	1.12010000
C	-0.07775600	-1.88057900	0.32300800
C	-1.28020700	-1.32026600	-0.15845300
C	-1.71245600	-0.00995500	-0.13803700
C	-1.22063800	1.27685200	0.20458800
C	0.02132200	1.85857200	0.40041400
C	1.32961400	1.37541900	0.18937400
C	1.64209000	0.21602300	-0.48382400
H	0.52482400	-0.37992900	1.70880400
H	1.73357700	-1.74743800	1.44655800
H	0.16199400	-2.87838800	-0.04103600
H	-1.99164700	-1.99849500	-0.62301400
H	-2.05366100	1.96938700	0.31142900
H	-0.03477600	2.87463600	0.78707600
H	0.88450500	-0.19154600	-1.14543900
C	3.05917500	-0.20259100	-0.75596300
H	3.73570000	0.15102700	0.02814800

H	3.14751500	-1.29057700	-0.82796500
H	3.40016200	0.21528600	-1.71159700
H	2.14121500	1.94256500	0.64606400
F	-3.02393300	0.09504800	-0.53180800

1-CH_3-5-CHO-substituted

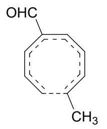

C	1.14509000	-1.18139600	1.19849200
C	0.13738500	-1.79734600	0.49865600
C	-1.04895100	-1.16455400	0.07620900
C	-1.41265700	0.17787700	0.06726700
C	-0.74787700	1.42065900	0.32273900
C	0.55042100	1.89776600	0.37435500
C	1.79184100	1.29054600	0.07012200
C	1.95877800	0.09601100	-0.58725700
H	0.94516000	-0.26437000	1.73609400
H	2.02587100	-1.74159900	1.50210400
H	0.27095200	-2.83355500	0.19213500
H	-1.83108300	-1.82159600	-0.30138600
H	-1.47223000	2.22077200	0.48562300
H	0.62424000	2.93103900	0.70961300
H	1.11578500	-0.28739000	-1.15175200
C	3.30908700	-0.45504600	-0.94536100
H	4.08338800	-0.08372100	-0.26720600
H	3.31411300	-1.54887400	-0.91454600
H	3.58040200	-0.15971200	-1.96663000
H	2.68410000	1.80308700	0.43252200
C	-2.86368700	0.39067000	-0.26326400
H	-3.22150500	1.43366100	-0.13927200
O	-3.62947100	-0.46235700	-0.64863100

1-CH_3-5-CN-substituted

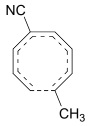

C	-1.18570700	1.19459300	1.12093700
C	-0.25551300	1.87437900	0.37355300
C	0.98512700	1.34834700	-0.03816800
C	1.47573500	0.04347300	0.01476200
C	0.95118800	-1.25290600	0.34349600
C	-0.29761900	-1.84035600	0.44904400
C	-1.59589000	-1.37207200	0.14647500
C	-1.88811200	-0.23028600	-0.56220000
H	-0.88196400	0.34182300	1.71286800
H	-2.11541000	1.67866500	1.40873800
H	-0.50080800	2.87036100	0.00954600
H	1.67979100	2.06965900	-0.46257300
H	1.75668100	-1.96244100	0.52426000
H	-0.26293400	-2.85563300	0.83998700
H	-1.09976400	0.19119100	-1.17656100
C	-3.29250400	0.16784400	-0.91587400

H	-4.01106600	-0.21438800	-0.18470200
H	-3.39711700	1.25545300	-0.97327000
H	-3.56142000	-0.23537900	-1.90011700
H	-2.42701900	-1.94635500	0.55772000
C	2.88918100	-0.02809500	-0.31441300
N	4.01510200	-0.08892600	-0.58022500

1-CH_3-5-NO_2-substituted

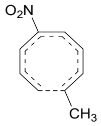

C	1.55940100	-1.06310400	1.24907200
C	0.61876100	-1.81541900	0.58949200
C	-0.62907900	-1.33899100	0.14023400
C	-1.10369200	-0.04012200	0.08242400
C	-0.60246500	1.27640700	0.27794300
C	0.64674700	1.87299300	0.28985800
C	1.94113900	1.38284200	0.00671300
C	2.23098900	0.17765300	-0.58928700
H	1.26413600	-0.15445600	1.75660200
H	2.49371700	-1.51600700	1.57088100
H	0.86093500	-2.84264800	0.32425700
H	-1.32677000	-2.08767300	-0.21955700
H	-1.41235500	1.98353200	0.42511800
H	0.61616600	2.92118000	0.58101400
H	1.43617200	-0.31168300	-1.14135700
C	3.63186600	-0.24614100	-0.92505900
H	4.36058000	0.21800800	-0.25381400
H	3.74428900	-1.33283800	-0.86517200
H	3.87868500	0.04809400	-1.95270700
H	2.77576500	2.00136300	0.33906700
N	-2.58592500	-0.01282900	-0.23352200
O	-3.04819700	-0.93535300	-0.88045900
O	-3.24578300	0.92613100	0.17320800

1- NH_2-5-CH_3-substituted

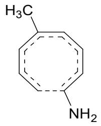

C	0.91807900	-1.12584400	1.11255700
C	-0.00664100	-1.83868900	0.36653800
C	-1.20481700	-1.32094400	-0.14146600
C	-1.72764500	-0.01827900	-0.11459400
C	-1.17057000	1.23802500	0.23025400
C	0.09903700	1.81730800	0.37854000
C	1.38738800	1.36322200	0.11460200
C	1.69902800	0.14586300	-0.50239500
H	0.57644400	-0.28297100	1.69882600
H	1.81110000	-1.62931200	1.47718200
H	0.24762900	-2.85325000	0.05914600
H	-1.85494400	-2.05832100	-0.60914500
H	-1.95295300	1.98712100	0.36364700
H	0.05892100	2.83524400	0.76340400

H	0.96356200	-0.28220700	-1.17265100
N	3.01335100	-0.16367800	-0.83516500
H	3.71101100	0.15207300	-0.17134200
H	3.17182000	-1.12408500	-1.11174500
H	2.21759300	1.97838000	0.46184100
C	-3.20569000	0.08497000	-0.48021600
H	-3.79475300	0.37300500	0.39887300
H	-3.37674200	0.84280700	-1.25148100
H	-3.60115800	-0.86653800	-0.84331900

1- NH_2-5-NH_2-substituted

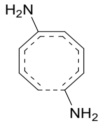

C	-0.88300800	1.14722200	1.14575000
C	0.04188500	1.83446200	0.37803600
C	1.20769400	1.30561700	-0.18333900
C	1.73853800	0.00219200	-0.13020900
C	1.18878300	-1.24941600	0.25569800
C	-0.08512500	-1.81952500	0.37581900
C	-1.37208100	-1.36672400	0.10351600
C	-1.68074000	-0.14174400	-0.49912100
H	-0.56398100	0.28749400	1.72019300
H	-1.76053500	1.67181100	1.51693500
H	-0.20709000	2.84995300	0.06823200
H	1.83568800	2.01122100	-0.72491700
H	1.97131000	-1.98483000	0.44432400
H	-0.05075800	-2.83266800	0.77389900
H	-0.93789700	0.30577900	-1.14902900
N	-2.99002300	0.17113400	-0.83258900
H	-3.69518500	-0.17099800	-0.19052200
H	-3.15350800	1.13490800	-1.09291300
H	-2.20234600	-1.98629700	0.44104000
N	3.08006700	-0.06540600	-0.56464000
H	3.51728000	-0.97343500	-0.47945800
H	3.68104100	0.67445400	-0.22408000

1- NH_2-5-OH-substituted

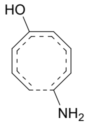

C	-0.87915800	1.14845000	1.12740100
C	0.04616200	1.84872100	0.37187500
C	1.22872700	1.32766700	-0.16197900
C	1.72750100	0.01992200	-0.12773100
C	1.20375300	-1.24034200	0.24786000
C	-0.06297800	-1.82159600	0.39017500
C	-1.35053600	-1.37209000	0.12031000
C	-1.65828100	-0.15262200	-0.49645800
H	-0.54887800	0.30070300	1.71319200
H	-1.76762700	1.66098700	1.48909700
H	-0.20458500	2.86324000	0.06205400
H	1.89719900	2.02496700	-0.66023600

H	2.00379900	-1.95949000	0.43129700
H	-0.01884400	-2.83146300	0.79458000
H	-0.91534900	0.28303600	-1.15456300
N	-2.96660900	0.15601600	-0.83682300
H	-3.67420000	-0.17375100	-0.19098500
H	-3.12898800	1.11427000	-1.11743400
H	-2.18015800	-1.98845800	0.46471200
O	3.05797300	-0.00394700	-0.50576600
H	3.30895900	-0.90323100	-0.75654600

1- NH_2-5-F-substituted

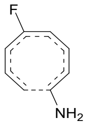

C	0.84013000	-1.16351900	1.11691400
C	-0.08135300	-1.85787700	0.35256800
C	-1.26525400	-1.31581800	-0.16022200
C	-1.70068500	0.00645700	-0.13008300
C	-1.19507700	1.27570500	0.19336400
C	0.07369400	1.83878300	0.37762300
C	1.35765000	1.35737500	0.14611000
C	1.65552200	0.14482800	-0.48750900
H	0.50420700	-0.32064800	1.70695100
H	1.72878000	-1.67517400	1.47868500
H	0.16015500	-2.87176700	0.03539900
H	-1.96714600	-1.99326300	-0.63913300
H	-2.00921000	1.99153600	0.28553800
H	0.03633400	2.85532400	0.76438500
H	0.91488600	-0.26978400	-1.16134600
N	2.96161100	-0.18382300	-0.81171300
H	3.66846900	0.12148200	-0.15342900
H	3.11202600	-1.13606300	-1.11760900
H	2.19244600	1.95328800	0.51374000
F	-3.01999900	0.10180300	-0.49819700

1- NH_2-5-CHO-substituted

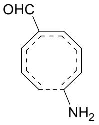

C	1.16483800	-1.11429300	1.18333300
C	0.14537500	-1.77504200	0.51741800
C	-1.03066700	-1.16769100	0.06847900
C	-1.39866700	0.18541400	0.06542200
C	-0.72908600	1.41333100	0.30006800
C	0.59073700	1.87198000	0.35060800
C	1.80868000	1.26808500	0.03570600
C	1.96414300	0.02548100	-0.58345700
H	0.93364900	-0.21443900	1.73721800
H	2.04038100	-1.66920500	1.51312700
H	0.28878900	-2.82175600	0.25131700
H	-1.80425400	-1.82790000	-0.31882600
H	-1.43699900	2.23223100	0.43679900
H	0.67938600	2.90392200	0.68524000

H	1.13875700	-0.36694200	-1.16382000
N	3.20636700	-0.42365200	-0.99243100
H	3.99407200	-0.14710100	-0.41904100
H	3.25617200	-1.39328900	-1.27530500
H	2.71610400	1.80376000	0.31497200
C	-2.85282300	0.40050600	-0.24221600
H	-3.20434600	1.44625800	-0.12194300
O	-3.62768400	-0.45332600	-0.60811200

1-NH_2-5-CN-substituted

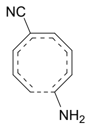

C	-1.19530600	1.12004200	1.10848800
C	-0.25633100	1.84920500	0.39480400
C	0.96906500	1.34536900	-0.04606900
C	1.46509900	0.02881000	0.01149500
C	0.93185300	-1.25236900	0.31053600
C	-0.34236300	-1.82256500	0.41323300
C	-1.61642000	-1.35490500	0.10359500
C	-1.90734500	-0.15578100	-0.55883200
H	-0.86144900	0.28680100	1.71229000
H	-2.11523900	1.60317900	1.42961300
H	-0.50952100	2.85905100	0.07519900
H	1.65615100	2.06741100	-0.48024200
H	1.71993700	-1.98712600	0.46120500
H	-0.32274300	-2.84028100	0.79772800
H	-1.14483500	0.27469600	-1.19590200
N	-3.19980200	0.14721000	-0.94459500
H	-3.93914400	-0.17318200	-0.33116200
H	-3.35658200	1.08656400	-1.28418000
H	-2.46158400	-1.96210500	0.42756600
C	2.88626900	-0.03510800	-0.28648000
N	4.01807200	-0.08309500	-0.52922300

1-NH_2-5-NO_2-substituted

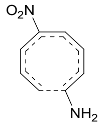

C	-1.57169300	1.13768000	1.09462000
C	-0.61464700	1.84678400	0.38375500
C	0.62409800	1.34354900	-0.01682600
C	1.10152700	0.03351600	0.07270300
C	0.57786600	-1.23711000	0.37194200
C	-0.69999600	-1.80013500	0.46118800
C	-1.96972300	-1.33913900	0.12403800
C	-2.26008200	-0.15602600	-0.56712100
H	-1.25583300	0.31955500	1.72794900
H	-2.49629900	1.63305600	1.38179400
H	-0.86560200	2.84443000	0.02660100
H	1.31965200	2.05288400	-0.45001200
H	1.36369800	-1.96319200	0.54933600
H	-0.68966100	-2.80768400	0.87137200

H	-1.49433300	0.26865000	-1.20359300
N	-3.54922500	0.13229500	-0.97129400
H	-4.29495300	-0.18280100	-0.36295700
H	-3.70875200	1.06208100	-1.33488900
H	-2.81735300	-1.94184800	0.45047100
N	2.59093500	-0.03488600	-0.20355200
O	3.09792200	-1.12790900	-0.38567400
O	3.21749900	1.01019600	-0.23656900

1-OH-5-CH_3-substituted

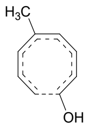

C	-0.91878300	1.17854600	1.08147500
C	0.01894600	1.85243500	0.32502100
C	1.22467700	1.30936500	-0.14968000
C	1.72812900	0.00614800	-0.10141800
C	1.14658700	-1.24106000	0.26026300
C	-0.12630200	-1.80067300	0.38608600
C	-1.41211100	-1.33306000	0.09577700
C	-1.70898400	-0.12659900	-0.52697100
H	-0.60438600	0.33551700	1.68316700
H	-1.81942200	1.69611400	1.40210100
H	-0.22558200	2.85559500	-0.02262300
H	1.89065200	2.03282800	-0.61687500
H	1.91812200	-1.99462600	0.42677700
H	-0.10918100	-2.81707300	0.77591400
H	-1.00896500	0.32265000	-1.21736900
O	-2.98552500	0.25110800	-0.80337900
H	-3.60604400	-0.27412100	-0.27492700
H	-2.25071000	-1.93299300	0.45284800
C	3.20378400	-0.13114900	-0.46146800
H	3.78158900	-0.44245700	0.41713800
H	3.35804300	-0.88693900	-1.23844400
H	3.62443500	0.81293300	-0.81517400

1-OH-5-NH_2-substituted

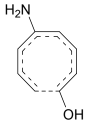

C	-0.88139500	1.20354300	1.12235500
C	0.05395500	1.84898900	0.34115300
C	1.22648800	1.29268000	-0.19125000
C	1.73687500	-0.01092200	-0.11913000
C	1.16245200	-1.25223200	0.29041900
C	-0.11311900	-1.80150700	0.39039600
C	-1.39721500	-1.33432900	0.08687100
C	-1.68753700	-0.12354800	-0.52689400
H	-0.59082100	0.34232100	1.71004800
H	-1.76646600	1.74209400	1.45003600
H	-0.18502700	2.85334900	-0.00784400
H	1.86899800	1.98361100	-0.73496600

H	1.93348700	-1.98939100	0.51579400
H	-0.10370900	-2.81051700	0.79945300
H	-0.97507100	0.34696500	-1.19057200
O	-2.95881700	0.25672800	-0.81039300
H	-3.58443500	-0.28728800	-0.30742900
H	-2.23736300	-1.93552800	0.43652600
N	3.07427800	-0.11790900	-0.55110700
H	3.49536100	-1.03158100	-0.45057800
H	3.69261400	0.62145300	-0.24310300

1-OH-5-OH-substituted

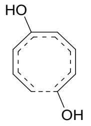

C	-0.87604700	1.20452800	1.10117100
C	0.06021300	1.86325500	0.33221900
C	1.24955900	1.31389600	-0.17071900
C	1.72546600	0.00542100	-0.11713300
C	1.17610400	-1.24515100	0.28031800
C	-0.09319400	-1.80534600	0.40190600
C	-1.37802500	-1.34017500	0.10354700
C	-1.66583800	-0.13436700	-0.52355400
H	-0.57385400	0.35517400	1.70023700
H	-1.77200300	1.73076600	1.41915700
H	-0.18050300	2.86604000	-0.01861600
H	1.93363600	1.99510100	-0.67031100
H	1.96514500	-1.96626100	0.49980500
H	-0.07396600	-2.81204400	0.81539500
H	-0.95288100	0.32144300	-1.19721400
O	-2.93513200	0.24611500	-0.81310600
H	-3.56410600	-0.28331600	-0.29886600
H	-2.21774500	-1.93707900	0.46092100
O	3.05570200	-0.05633400	-0.48839000
H	3.28228700	-0.96042300	-0.74507700

1-OH-5-F-substituted

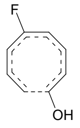

C	-0.83564600	1.21999600	1.08608300
C	0.09691100	1.87166200	0.30828300
C	1.28835800	1.30048000	-0.16812200
C	1.70010000	-0.02172700	-0.11789000
C	1.16687200	-1.28379600	0.22034700
C	-0.10549800	-1.82481300	0.38268400
C	-1.38782900	-1.32572600	0.12875900
C	-1.66589000	-0.12683900	-0.51335000
H	-0.52734500	0.37647600	1.69077900
H	-1.73242700	1.74513800	1.40298800
H	-0.13417100	2.87252100	-0.05271700
H	2.00885300	1.96139600	-0.64280300
H	1.96983400	-2.00721900	0.34482600
H	-0.09227600	-2.84083300	0.77168700

H	-0.95613200	0.30592700	-1.20518100
O	-2.93121200	0.27928500	-0.78312000
H	-3.56369300	-0.23010300	-0.25305100
H	-2.23123000	-1.90162800	0.51153800
F	3.01822400	-0.15237700	-0.47376300

1-OH-5-CHO-substituted

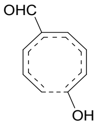

C	1.15694600	-1.18172600	1.13885500
C	0.13212300	-1.79967700	0.45268600
C	-1.04877400	-1.16307900	0.04379000
C	-1.39933600	0.18805500	0.07259300
C	-0.70816200	1.40629400	0.32664600
C	0.60951600	1.85075300	0.35708100
C	1.82802100	1.24083300	0.02063000
C	1.98386900	0.01532200	-0.60601100
H	0.94877400	-0.28601300	1.70954600
H	2.03675800	-1.75154800	1.42616500
H	0.26630900	-2.83293800	0.13672700
H	-1.83663800	-1.80612300	-0.34459800
H	-1.40559900	2.22724200	0.50013600
H	0.71396000	2.87952000	0.69614600
H	1.20098800	-0.40747700	-1.21892300
O	3.19719800	-0.48933600	-0.93687600
H	3.89897100	-0.00085900	-0.47895500
H	2.73718700	1.76632700	0.31762400
C	-2.85470800	0.42864600	-0.21514000
H	-3.19191100	1.47546000	-0.06723000
O	-3.64291900	-0.40767900	-0.59105100

1-OH-5-CN-substituted

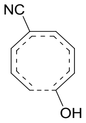

C	-1.19676300	1.17974700	1.07118200
C	-0.25060800	1.86995700	0.34045800
C	0.98364000	1.34378300	-0.06430200
C	1.46464600	0.03015900	0.01800700
C	0.91268900	-1.24586300	0.33667200
C	-0.35900800	-1.80551600	0.41887800
C	-1.63550800	-1.33380300	0.08772500
C	-1.91986600	-0.15183000	-0.58201500
H	-0.88576800	0.34730000	1.68893800
H	-2.12481800	1.67256400	1.34870300
H	-0.49721200	2.86529200	-0.02441900
H	1.68351200	2.05383700	-0.49819100
H	1.69418100	-1.98002600	0.52114300
H	-0.35333200	-2.82192000	0.80670200
H	-1.19573100	0.30206700	-1.24361000
O	-3.18172500	0.21789800	-0.90594100
H	-3.82540300	-0.30614600	-0.40401800

H	-2.48345800	-1.93050300	0.42719100
C	2.88609200	-0.05831600	-0.26971400
N	4.01856200	-0.13079400	-0.50232300

1-OH-5-NO_2-substituted

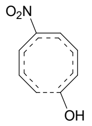

C	-1.57052600	1.05642000	1.19209100
C	-0.61660700	1.81820100	0.54733400
C	0.62580900	1.34426100	0.10420700
C	1.09379800	0.03529200	0.07669300
C	0.56840700	-1.26207800	0.26528800
C	-0.70145700	-1.83235300	0.26009200
C	-1.97545100	-1.33892000	-0.04671600
C	-2.26362800	-0.09923600	-0.60116300
H	-1.26587600	0.16799600	1.73018800
H	-2.50392600	1.51770400	1.50402700
H	-0.86377700	2.84337000	0.27872500
H	1.32555800	2.08399100	-0.26859300
H	1.35720100	-1.99180700	0.41398500
H	-0.69601900	-2.88111100	0.54854300
H	-1.53628500	0.42713700	-1.20252500
O	-3.52453400	0.28936000	-0.90371500
H	-4.16965900	-0.28822700	-0.46642500
H	-2.82291800	-1.97256500	0.21892800
N	2.58247900	-0.01292000	-0.20103400
O	3.07250900	0.90225100	-0.83741600
O	3.21906000	-0.95980700	0.22406000

1-F-5-CH_3-substituted

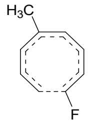

C	-0.92768500	1.23376800	1.07709500
C	0.01522100	1.87502400	0.31048000
C	1.22270500	1.30831200	-0.15121700
C	1.71456500	0.00731000	-0.09736400
C	1.13038400	-1.23949000	0.29196900
C	-0.13309100	-1.80342800	0.41028000
C	-1.42588400	-1.33863700	0.10287500
C	-1.69662000	-0.15824900	-0.53960400
H	-0.64189100	0.37752100	1.67416200
H	-1.83328300	1.75541300	1.37545400
H	-0.21251500	2.87516800	-0.05637400
H	1.89835300	2.02338500	-0.61765500
H	1.90647500	-1.98107400	0.48900600
H	-0.11777300	-2.81415000	0.81361400
H	-1.02776800	0.32043300	-1.24105800
F	-2.98723300	0.17070900	-0.75451400
H	-2.27926200	-1.90455500	0.46983100
C	3.18318100	-0.15057100	-0.47525100
H	3.76703000	-0.46917700	0.39671300

H	3.31669200	-0.91023600	-1.25231200
H	3.61238700	0.78666700	-0.83633800

1-F-5-NH_2-substituted

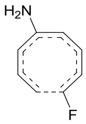

C	-0.89255800	1.25667700	1.12055300
C	0.04822100	1.87101100	0.33032400
C	1.22028800	1.29184800	-0.19346400
C	1.72158500	-0.00943400	-0.11650200
C	1.14684500	-1.24891400	0.32414200
C	-0.11863500	-1.80409100	0.41842800
C	-1.40902500	-1.34168100	0.09246200
C	-1.67136000	-0.15545700	-0.53912100
H	-0.63036700	0.38305200	1.70313200
H	-1.77921100	1.80235200	1.43067100
H	-0.17337400	2.87397900	-0.03342900
H	1.87036500	1.97517100	-0.73816400
H	1.92259200	-1.97009800	0.58288600
H	-0.11114100	-2.80496800	0.84603100
H	-0.98721100	0.34555300	-1.21022000
F	-2.95697200	0.17401800	-0.77071200
H	-2.26457700	-1.91008600	0.44865700
N	3.04946200	-0.13837600	-0.56506200
H	3.46481900	-1.05443100	-0.46581700
H	3.68245700	0.60219400	-0.29282700

1-F-5-OH-substituted

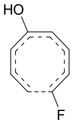

C	-0.88485400	1.26018600	1.09847400
C	0.05616700	1.88603300	0.31849800
C	1.24541800	1.31192600	-0.17410300
C	1.70959100	0.00538700	-0.11599000
C	1.15949400	-1.24355800	0.31217000
C	-0.09988600	-1.80847400	0.42886600
C	-1.39154000	-1.34634100	0.11062400
C	-1.65098300	-0.16680000	-0.53587200
H	-0.61095000	0.39740200	1.69184600
H	-1.78331800	1.79254300	1.39773600
H	-0.16709000	2.88651100	-0.04963100
H	1.93894500	1.98359700	-0.67403400
H	1.95369100	-1.94890300	0.56240100
H	-0.08289500	-2.80773100	0.85917100
H	-0.96626200	0.31843900	-1.21821900
F	-2.93453100	0.16641900	-0.76833600
H	-2.24677200	-1.90871500	0.47687900
O	3.03381400	-0.07724600	-0.50256900
H	3.24448300	-0.98309400	-0.76657300

1-F-5-F-substituted

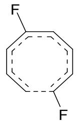

C	-0.84111000	1.28084400	1.07855500
C	0.09679400	1.89485500	0.28825200
C	1.28966600	1.29631400	-0.17029200
C	1.68714500	-0.02363000	-0.11518800
C	1.14932900	-1.28676400	0.24667300
C	-0.11448000	-1.82929400	0.40317800
C	-1.40493400	-1.32902400	0.13725700
C	-1.65740600	-0.15871500	-0.52564000
H	-0.56285900	0.42460600	1.67994200
H	-1.74165300	1.81241800	1.37255200
H	-0.11509400	2.89143400	-0.09529200
H	2.02367300	1.94649800	-0.63960400
H	1.95585400	-2.00186300	0.39552400
H	-0.10373600	-2.84161400	0.80084700
H	-0.97968800	0.30126000	-1.23199100
F	-2.93733400	0.20431600	-0.72626800
H	-2.26194600	-1.86803000	0.53395300
F	2.99904700	-0.17456300	-0.48180900

1-F-5-CHO-substituted

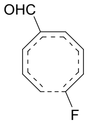

C	1.15630200	-1.25011200	1.13646300
C	0.12564600	-1.82902700	0.43885400
C	-1.05126300	-1.16104300	0.04574700
C	-1.38581700	0.18803700	0.08405600
C	-0.68709900	1.40407800	0.36536700
C	0.62283800	1.84990900	0.38691500
C	1.84527600	1.24035800	0.02634400
C	1.97255100	0.04519400	-0.62336400
H	0.98331500	-0.34008400	1.69631300
H	2.03529600	-1.83025000	1.40421100
H	0.23528100	-2.85911300	0.10422400
H	-1.85147600	-1.78996100	-0.34173100
H	-1.38526800	2.21705400	0.57125800
H	0.73226500	2.87324300	0.73935500
H	1.21570500	-0.40858200	-1.24690900
F	3.20875900	-0.40492900	-0.90462300
H	2.76916200	1.72949700	0.32777700
C	-2.83823700	0.44471000	-0.21418400
H	-3.17136100	1.48925700	-0.04511500
O	-3.62536500	-0.37866700	-0.61812000

1-F-5-CN-substituted

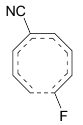

C	-1.20699100	1.24209300	1.06472100
C	-0.25582500	1.89742400	0.32231600
C	0.98154900	1.34671400	-0.06491500
C	1.45151400	0.03662400	0.02633700
C	0.89708500	-1.24037700	0.37524100
C	-0.36356100	-1.80640500	0.44849700
C	-1.64788600	-1.34140200	0.09544600
C	-1.90472300	-0.19113900	-0.59891900
H	-0.92459200	0.39439900	1.67547000
H	-2.14103100	1.73673400	1.31714000
H	-0.48434200	2.88865200	-0.06451500
H	1.69123800	2.04819000	-0.49733200
H	1.68321900	-1.96057700	0.59340500
H	-0.35850500	-2.81705700	0.85029200
H	-1.21046700	0.29213400	-1.27187100
F	-3.18341300	0.12800200	-0.86356500
H	-2.51126300	-1.90424800	0.44344600
C	2.86896900	-0.06733000	-0.27571600
N	3.99795300	-0.15535300	-0.51885700

1-F-5-NO_2-substituted

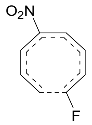

C	1.57736000	-1.12430100	1.19093700
C	0.61726900	-1.84850600	0.52869500
C	-0.62819900	-1.34614100	0.10229700
C	-1.08435000	-0.04009900	0.08410600
C	-0.55530700	1.26042500	0.30066300
C	0.70424100	1.83411900	0.28691600
C	1.98622900	1.34525800	-0.04044700
C	2.25251800	0.13554700	-0.62137200
H	1.30487700	-0.22071200	1.72087400
H	2.51459100	-1.59393600	1.47738100
H	0.84454900	-2.87186700	0.23710900
H	-1.34001700	-2.07613300	-0.26816400
H	-1.34642400	1.98079300	0.48103200
H	0.70053600	2.87892600	0.58835500
H	1.56237300	-0.42525800	-1.23536900
F	3.53377400	-0.19055600	-0.86295200
H	2.84809500	1.94799300	0.23744200
N	-2.57127800	0.02023400	-0.20526600
O	-3.20535500	0.95948200	0.23868800
O	-3.05866600	-0.87751300	-0.86693800

1-CHO-5-CH_3-substituted

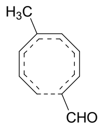

C	-0.60104300	1.28956300	1.06496400
C	0.37871500	1.90122800	0.31791400
C	1.57407200	1.30368500	-0.12963000
C	2.04718100	-0.00905200	-0.10733400
C	1.45967400	-1.26361100	0.24031000
C	0.19793800	-1.83671400	0.38580200
C	-1.08991500	-1.34527400	0.12451700
C	-1.35668200	-0.15954000	-0.53019300
H	-0.36597100	0.40907600	1.64816600
H	-1.48152600	1.84792100	1.37239700
H	0.20095600	2.91883900	-0.02716000
H	2.27430000	2.01312000	-0.56761900
H	2.23939300	-2.01133200	0.39807500
H	0.22884000	-2.85306400	0.77483400
H	-0.61366100	0.25893600	-1.19803700
C	-2.75259300	0.29950100	-0.70947100
H	-2.89556000	1.13159700	-1.42723200
O	-3.70258100	-0.15822100	-0.11009200
H	-1.93840000	-1.89619400	0.52809300
C	3.51990800	-0.16581600	-0.47779800
H	4.09790300	-0.49485000	0.39406500
H	3.65701000	-0.91593800	-1.26307300
H	3.95383700	0.77383600	-0.82626300

1-CHO-5-NH_2-substituted

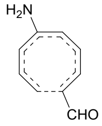

C	-0.56173000	1.31670800	1.10464300
C	0.41688500	1.89806200	0.33382900
C	1.57960000	1.28662200	-0.16940100
C	2.05622200	-0.02825500	-0.12636600
C	1.47375800	-1.27621500	0.27334900
C	0.20951800	-1.83760600	0.39568100
C	-1.07892700	-1.34743300	0.11797400
C	-1.33941800	-0.15998300	-0.52824900
H	-0.35436900	0.41946500	1.67268700
H	-1.42358800	1.89869100	1.41929500
H	0.24874400	2.91749600	-0.01150600
H	2.26021300	1.96558700	-0.68182000
H	2.25265300	-2.00831100	0.49029700
H	0.23246900	-2.84582000	0.80603800
H	-0.58587100	0.27941300	-1.17051900
C	-2.73105100	0.31256000	-0.70403000
H	-2.86204400	1.17069200	-1.39270200
O	-3.68987700	-0.16108600	-0.13147800
H	-1.92820600	-1.89939400	0.51724200
N	3.38718800	-0.15519300	-0.57378800
H	3.79399000	-1.07735800	-0.49711600
H	4.02557300	0.56781900	-0.26813400

1-CHO-5-OH-substituted

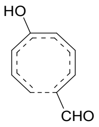

C	-0.55353400	1.32637400	1.08502800
C	0.42738600	1.91405000	0.32308200
C	1.60403400	1.30291200	-0.15007500
C	2.04212300	-0.01700000	-0.12639600
C	1.48378900	-1.27251200	0.26323800
C	0.22542100	-1.84084200	0.40968300
C	-1.06278900	-1.34840700	0.13741700
C	-1.32058200	-0.16849300	-0.52444500
H	-0.33843100	0.43826600	1.66447600
H	-1.42461700	1.90039800	1.38895200
H	0.26147400	2.93210400	-0.02598400
H	2.32863900	1.96556800	-0.61747800
H	2.27996500	-1.99023800	0.47050400
H	0.25530400	-2.84693900	0.82390900
H	-0.56719100	0.25875600	-1.17545700
C	-2.71284000	0.30143100	-0.70952400
H	-2.84444100	1.14540300	-1.41500100
O	-3.66990400	-0.16248900	-0.12715000
H	-1.91274600	-1.89328900	0.54477900
O	3.36847400	-0.09971300	-0.51080600
H	3.57543200	-0.99748600	-0.80310800

1-CHO-5-F-substituted

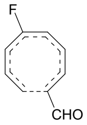

C	-0.53396000	1.34434800	1.05424700
C	0.44512300	1.92249300	0.28609500
C	1.63166000	1.29376900	-0.14399400
C	2.01620800	-0.03348400	-0.11802500
C	1.48242600	-1.30882100	0.19967100
C	0.22597900	-1.86840800	0.37738200
C	-1.06097000	-1.34778200	0.14968300
C	-1.31823400	-0.17774800	-0.52800600
H	-0.31495000	0.46614600	1.64774200
H	-1.41198200	1.91481400	1.34426200
H	0.28627200	2.93383400	-0.08355000
H	2.39230400	1.93858100	-0.57777900
H	2.29768000	-2.02161600	0.31165200
H	0.25499700	-2.88722200	0.75821100
H	-0.57252200	0.22943900	-1.20080800
C	-2.70914000	0.30498300	-0.69387500
H	-2.84825900	1.12744800	-1.42262000
O	-3.65594000	-0.12959900	-0.07402000
H	-1.90985300	-1.87548600	0.58167600
F	3.33325300	-0.17391600	-0.47396600

1-CHO-5-CHO-substituted

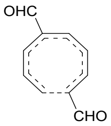

C	0.83927100	-1.27304500	1.15915100
C	-0.19810600	-1.83716400	0.46094000
C	-1.36865700	-1.16271000	0.06016700
C	-1.71779400	0.18523600	0.06408000
C	-1.05305900	1.42389200	0.32382900
C	0.24326300	1.91222900	0.38476000
C	1.48059300	1.30888800	0.08069400
C	1.64240900	0.11421800	-0.57998800
H	0.69516200	-0.34339300	1.69380800
H	1.70283800	-1.86749900	1.44551900
H	-0.11012500	-2.87580200	0.14758300
H	-2.16922200	-1.79956400	-0.31459000
H	-1.78037600	2.22073300	0.49066300
H	0.30883500	2.94260600	0.72869000
H	0.83644800	-0.29144400	-1.17885200
C	2.99876300	-0.43587400	-0.80820100
H	3.06376800	-1.27550200	-1.52714200
O	3.99485100	-0.03456600	-0.24516900
H	2.38412700	1.80422000	0.43479500
C	-3.17448900	0.40608200	-0.25464200
H	-3.53128500	1.44619900	-0.11051800
O	-3.93901800	-0.44181800	-0.64916700

1-CHO-5-CN-substituted

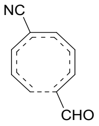

C	-0.86520800	1.31301200	1.07497400
C	0.11562500	1.93063000	0.33957000
C	1.33540300	1.34164700	-0.04622100
C	1.78447700	0.02026100	0.01239900
C	1.22928400	-1.26365200	0.33351300
C	-0.02926300	-1.83460600	0.43934300
C	-1.31200900	-1.33792900	0.13637700
C	-1.56864000	-0.18511800	-0.56922700
H	-0.63303600	0.43590100	1.66462500
H	-1.77194700	1.84863100	1.34301400
H	-0.06384300	2.93915300	-0.02737600
H	2.06571900	2.03392200	-0.45942600
H	2.02130800	-1.98813200	0.51554500
H	-0.01075700	-2.84997400	0.82973300
H	-0.80751000	0.23693600	-1.21423100
C	-2.96734400	0.25359900	-0.79818500
H	-3.10776300	1.03962500	-1.56486100
O	-3.91778200	-0.18284500	-0.18642800
H	-2.17042400	-1.87882800	0.53313900
C	3.19994500	-0.08819800	-0.30016200
N	4.32669800	-0.17890700	-0.55186000

1-CHO-5-NO_2-substituted

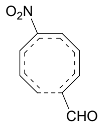

C	1.24906800	-1.20723800	1.21185000
C	0.25784000	-1.88975200	0.55196000
C	-0.96860100	-1.34472800	0.12381700
C	-1.40158500	-0.02980600	0.07681300
C	-0.87356800	1.27325200	0.27721200
C	0.38447700	1.85535300	0.30138300
C	1.66485500	1.34093300	0.02006500
C	1.92315700	0.13016300	-0.58068100
H	1.02938200	-0.27888700	1.72249200
H	2.15963000	-1.71847800	1.51305400
H	0.43491800	-2.92784800	0.27892400
H	-1.70128800	-2.06097800	-0.23363400
H	-1.67272100	1.99309300	0.42509100
H	0.36728800	2.90136700	0.59946800
H	1.15705500	-0.35946800	-1.16925800
C	3.32385700	-0.31169900	-0.79247800
H	3.46265900	-1.16362600	-1.48558100
O	4.27739400	0.19180300	-0.24001200
H	2.52501700	1.92424900	0.34658600
N	-2.88749600	0.03831600	-0.23261600
O	-3.36728900	-0.85293600	-0.90787000
O	-3.52341400	0.97657200	0.20932300

1-CN-5-CH_3-substituted

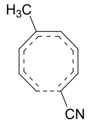

C	0.68844900	-1.13839700	1.19509000
C	-0.18189200	-1.85003300	0.40083900
C	-1.38762900	-1.36026400	-0.13694100
C	-1.95926400	-0.08707600	-0.16293100
C	-1.48546000	1.20881400	0.20344200
C	-0.27877500	1.87430500	0.40840800
C	1.05576000	1.48657600	0.21894600
C	1.43712400	0.30060600	-0.38459000
H	0.33009200	-0.27910700	1.74762200
H	1.59710700	-1.60661300	1.56375600
H	0.10907400	-2.85117200	0.08725900
H	-1.99763900	-2.12640600	-0.61246400
H	-2.32387800	1.90027000	0.30647700
H	-0.40258100	2.89015200	0.77845500
H	0.76293700	-0.17927900	-1.08237900
C	2.82932800	-0.01560400	-0.54782300
N	3.94847700	-0.29447800	-0.66041300
H	1.82884300	2.12403300	0.64194700
C	-3.41254500	-0.04206900	-0.62672500
H	-4.06422500	0.26635300	0.19941500
H	-3.55030200	0.67908200	-1.43856400
H	-3.75934100	-1.01712400	-0.97491600

1-CN-5-NH_2-substituted

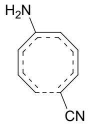

C	0.66527200	-1.13575800	1.22199300
C	-0.20514300	-1.84421800	0.42323800
C	-1.38462200	-1.35664200	-0.16329600
C	-1.96616700	-0.08285400	-0.17540900
C	-1.50722500	1.21163000	0.23447000
C	-0.30158100	1.87955000	0.40447300
C	1.03485000	1.49888900	0.19998800
C	1.41018300	0.29929300	-0.37780600
H	0.31504300	-0.26825400	1.76689400
H	1.56584000	-1.61302500	1.59759000
H	0.08731400	-2.84738400	0.11696300
H	-1.97113500	-2.11215600	-0.68483000
H	-2.36173100	1.86683800	0.40415600
H	-0.42551300	2.89055600	0.78746000
H	0.72973200	-0.19668300	-1.05894300
C	2.80117300	-0.01990200	-0.54786000
N	3.91911500	-0.30012800	-0.66860200
H	1.80801800	2.14445100	0.60888000
N	-3.31486600	-0.03977400	-0.58161700
H	-3.56240100	0.73065500	-1.18981500
H	-3.69535000	-0.91561500	-0.91556500

1-CN-5-OH-substituted

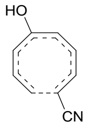

C	0.64559300	-1.17378500	1.21666900
C	-0.21986500	-1.86680200	0.40390100
C	-1.40764400	-1.37015600	-0.16332500
C	-1.95327800	-0.09070300	-0.18880600
C	-1.52043900	1.20680800	0.22129700
C	-0.32130600	1.87381600	0.43053800
C	1.01711800	1.49259100	0.23349500
C	1.39941600	0.31498200	-0.37843100
H	0.30060800	-0.30720200	1.76611400
H	1.54912300	-1.65262400	1.58313400
H	0.06768500	-2.86740500	0.08600700
H	-2.03667400	-2.09630200	-0.67257300
H	-2.38040300	1.86162000	0.37374600
H	-0.45111900	2.87861300	0.82681600
H	0.71962300	-0.17966000	-1.06102000
C	2.79111300	0.00115300	-0.54609500
N	3.91065100	-0.27292400	-0.66542200
H	1.78688300	2.12945000	0.66214900
O	-3.25061100	-0.11761100	-0.66256500
H	-3.50963600	0.75744200	-0.98135200

1-CN-5-F-substituted

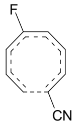

C	0.62601200	-1.18968500	1.18572000
C	-0.23577800	-1.87767200	0.36678600
C	-1.43705500	-1.36617500	-0.16122000
C	-1.92984100	-0.07411000	-0.18081100
C	-1.51842800	1.23956200	0.15797100
C	-0.32268900	1.89876000	0.40256100
C	1.01428400	1.49191500	0.25052400
C	1.39561900	0.32269200	-0.37701100
H	0.27233600	-0.33480600	1.74849900
H	1.53656100	-1.66258400	1.54227700
H	0.04820300	-2.87287200	0.03089000
H	-2.10441300	-2.07748200	-0.64193400
H	-2.39147300	1.88780000	0.20940900
H	-0.45266700	2.91511400	0.76720700
H	0.72309400	-0.14970700	-1.08235200
C	2.78602600	-0.00797200	-0.52132600
N	3.90378400	-0.29639600	-0.62068400
H	1.78356800	2.11229900	0.70411400
F	-3.22340000	-0.04076400	-0.63027700

1-CN-5-CHO-substituted

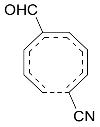

C	0.93490200	-1.08719600	1.29871500
C	-0.02685400	-1.75836400	0.58236200
C	-1.21590000	-1.18353200	0.09841500
C	-1.64482800	0.14116500	0.03382400
C	-1.06240000	1.42250100	0.27160100
C	0.20174000	1.98718000	0.36825000
C	1.48480500	1.45591300	0.13541200
C	1.73301700	0.23154500	-0.44893000
H	0.68870900	-0.15994300	1.80029000
H	1.82671500	-1.60532800	1.64109000
H	0.15365400	-2.79808100	0.31688200
H	-1.95402100	-1.88308400	-0.29141200
H	-1.83763300	2.18435400	0.37225300
H	0.19513600	3.03205700	0.67065500
H	0.97632100	-0.23981900	-1.06203200
C	3.08023100	-0.21790900	-0.65980400
N	4.16240000	-0.60311200	-0.81140900
H	2.33470900	2.03678000	0.48763600
C	-3.09599900	0.26376900	-0.35649900
H	-3.51584800	1.28717500	-0.27864800
O	-3.79210300	-0.64534400	-0.73961500

1-CN-5-CN-substituted

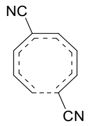

C	0.95311100	-1.14861600	1.20892500
C	0.05851600	-1.85842600	0.44320300
C	-1.17200700	-1.36240600	-0.02244200
C	-1.70178600	-0.06832600	-0.01485100
C	-1.23496000	1.24877500	0.30252800
C	-0.01629000	1.89421400	0.45463600
C	1.30655300	1.47838100	0.21801100
C	1.65396300	0.30954600	-0.42908800
H	0.61807400	-0.28658600	1.77170200
H	1.88317600	-1.60837800	1.53181200
H	0.33164400	-2.85824800	0.11304400
H	-1.83394100	-2.10593600	-0.46088400
H	-2.07251300	1.93284500	0.42775800
H	-0.11025900	2.91437600	0.81958300
H	0.94758200	-0.16791300	-1.09586400
C	3.03502800	-0.02466600	-0.64138400
N	4.14483300	-0.31864600	-0.79579400
H	2.10334100	2.09823500	0.62323200
C	-3.10427600	-0.04870700	-0.39545700
N	-4.22115000	-0.02949900	-0.70061500

1-CN-5-NO_2-substituted

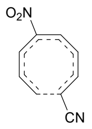

C	1.33278900	-1.01290200	1.33711500
C	0.42110100	-1.79009000	0.66257300
C	-0.81602200	-1.33883800	0.16897100
C	-1.32096900	-0.05082200	0.07121900
C	-0.87113600	1.28141400	0.25311700
C	0.35200400	1.93489200	0.30790400
C	1.66891400	1.49599400	0.08251100
C	2.00571400	0.26930500	-0.45405800
H	1.01098700	-0.09731300	1.81739000
H	2.26663700	-1.44362300	1.68772500
H	0.68345900	-2.81891600	0.42689600
H	-1.48926700	-2.10552600	-0.20035400
H	-1.71277100	1.96107900	0.34641300
H	0.26656300	2.98569900	0.57420600
H	1.28745400	-0.27322800	-1.05475500
C	3.38287800	-0.08316900	-0.66096700
N	4.49011400	-0.39049200	-0.80744200
H	2.47367500	2.15250800	0.40615600
N	-2.79615800	-0.07334700	-0.29401400
O	-3.20225700	-1.01716500	-0.94488000
O	-3.49475100	0.84860100	0.08190500

1-NO_2-5-CH_3-substituted

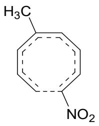

C	0.32344400	-1.01095000	1.38138000
C	-0.52508800	-1.78660800	0.63261500
C	-1.72065000	-1.34612100	0.02283500
C	-2.28603200	-0.08484800	-0.14601500
C	-1.81652600	1.24510300	0.10159300
C	-0.61455600	1.92682600	0.24719900
C	0.72373800	1.51251400	0.12472500
C	1.09111800	0.27820700	-0.34249200
H	-0.03557100	-0.09360200	1.83141900
H	1.22878800	-1.43836900	1.80524500
H	-0.22864700	-2.81049000	0.41232100
H	-2.32298000	-2.15517400	-0.38688400
H	-2.65725500	1.94063700	0.13020300
H	-0.73736900	2.97699700	0.50303700
H	0.50689600	-0.30990100	-1.03244600
N	2.50629100	-0.06615400	-0.40474300
O	2.80436700	-0.99869900	-1.13496800
O	3.29324200	0.55980800	0.28750000
H	1.50771800	2.16614400	0.49845700
C	-3.72394400	-0.08141500	-0.65605600
H	-4.39506900	0.33807600	0.10260700
H	-3.82492000	0.53222800	-1.55709700
H	-4.07552600	-1.08858600	-0.88861400

1-NO_2-5-NH_2-substituted

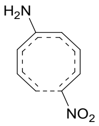

C	0.29932700	-1.00677700	1.41223800
C	-0.54646000	-1.77817700	0.65410500
C	-1.71232000	-1.34423300	-0.00724400
C	-2.28779300	-0.08082200	-0.16335600
C	-1.83507700	1.25233100	0.12867700
C	-0.63404500	1.93196400	0.24438700
C	0.70681200	1.52180700	0.10905400
C	1.06687100	0.27819000	-0.33435000
H	-0.05174400	-0.08170200	1.85261900
H	1.19455500	-1.44368700	1.84655400
H	-0.24781500	-2.80331000	0.44175500
H	-2.28785500	-2.14674400	-0.46716500
H	-2.69260300	1.91877000	0.22067400
H	-0.75707200	2.97831700	0.51537900
H	0.47339100	-0.32421500	-1.00444800
N	2.48134200	-0.07017800	-0.40530300
O	2.77102300	-1.01057300	-1.12908500
O	3.27609100	0.56220100	0.27140500
H	1.49104400	2.17963900	0.47333500
N	-3.62302500	-0.07179000	-0.61100000
H	-3.84802500	0.63306700	-1.30202000
H	-4.00290300	-0.97508900	-0.86217800

1-NO_2-5-OH-substituted

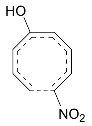

C	0.27828700	-1.04944500	1.40966500
C	-0.56292000	-1.80449400	0.63493700
C	-1.73692400	-1.35711800	-0.00780100
C	-2.27328900	-0.08845200	-0.17722300
C	-1.84504600	1.24665300	0.11616800
C	-0.65026800	1.92760700	0.27222200
C	0.69187300	1.51554200	0.14451700
C	1.05801900	0.28997200	-0.33560300
H	-0.06682200	-0.12509400	1.85617200
H	1.17464000	-1.49134600	1.83659300
H	-0.27082400	-2.82845500	0.40988400
H	-2.35712000	-2.12825400	-0.45864100
H	-2.70718200	1.91172900	0.19213200
H	-0.77786600	2.96918800	0.55799000
H	0.46557200	-0.30869900	-1.00992600
N	2.47327100	-0.05545000	-0.40446300
O	2.76661500	-0.98492100	-1.13973300
O	3.26336000	0.57050400	0.28346000
H	1.47330700	2.16513400	0.52923500
O	-3.55580700	-0.15348500	-0.68433700
H	-3.79833700	0.68557600	-1.09861800

1-NO_2-5-F-substituted

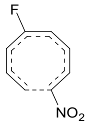

C	0.26819900	-1.07672500	1.36274900
C	-0.57253100	-1.82228400	0.58074600
C	-1.76625800	-1.35354800	-0.01235200
C	-2.25215900	-0.07200700	-0.16673600
C	-1.84270400	1.27251000	0.05243700
C	-0.65185800	1.94999500	0.24122700
C	0.68835000	1.51906400	0.15949400
C	1.05621800	0.30349700	-0.34295700
H	-0.08470900	-0.16726500	1.83393800
H	1.17538100	-1.51504600	1.77017400
H	-0.28316600	-2.83975800	0.32681700
H	-2.42935700	-2.10506300	-0.43430200
H	-2.71659000	1.92126400	0.03594000
H	-0.77983400	2.99942900	0.49506200
H	0.47520200	-0.27272900	-1.04647900
N	2.46923300	-0.05662200	-0.38262600
O	2.76928000	-0.97987300	-1.12247100
O	3.24816200	0.55154400	0.33306400
H	1.46697000	2.15406800	0.57403700
F	-3.53462200	-0.07499200	-0.64546700

1-NO_2-5-CHO-substituted

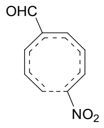

C	0.56312900	-0.92373100	1.49445800
C	-0.36506100	-1.66207800	0.80983800
C	-1.54792800	-1.14193400	0.24188200
C	-1.98317500	0.16211700	0.03438400
C	-1.41545600	1.47011000	0.15634100
C	-0.16108100	2.05313300	0.20786200
C	1.13210200	1.50574700	0.06050400
C	1.38413100	0.24048400	-0.38452100
H	0.30496400	0.05348300	1.88388000
H	1.45311700	-1.39400000	1.90547100
H	-0.16917800	-2.71838900	0.63879200
H	-2.27217600	-1.88338100	-0.09388400
H	-2.20015900	2.22897400	0.17324500
H	-0.17723400	3.12438500	0.39373500
H	0.72103200	-0.34998200	-0.99611100
N	2.76348900	-0.22317900	-0.47778700
O	2.94987400	-1.22124300	-1.15536500
O	3.62813900	0.37989100	0.13713700
H	1.98473500	2.10416800	0.37264700
C	-3.42626700	0.23195100	-0.40121400
H	-3.85578700	1.25368900	-0.43416700
O	-4.10502500	-0.71758300	-0.70881100

1-NO_2-5-CN-substituted

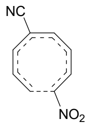

C	0.57774800	-1.01889500	1.40827700
C	-0.29232900	-1.79100100	0.68375500
C	-1.51436900	-1.33840300	0.14347700
C	-2.04054800	-0.05380700	0.00685700
C	-1.57891500	1.29358900	0.20065700
C	-0.36583200	1.95196400	0.29654400
C	0.96295000	1.50672000	0.13472300
C	1.30229200	0.28590100	-0.37468000
H	0.23792200	-0.09856400	1.86745700
H	1.50383700	-1.44068500	1.79074900
H	-0.01198300	-2.81465500	0.44630400
H	-2.16838700	-2.12250500	-0.23191300
H	-2.42008200	1.98288800	0.24935600
H	-0.46100000	3.00629900	0.54433200
H	0.68861000	-0.30091500	-1.03973800
N	2.71221000	-0.08000200	-0.47608500
O	2.97072900	-1.01858800	-1.21148100
O	3.52543700	0.53745200	0.19101900
H	1.76847700	2.14228200	0.49474400
C	-3.43315900	-0.07077800	-0.40823800
N	-4.54274700	-0.07668300	-0.73903300

1-NO_2-5-NO_2-substituted

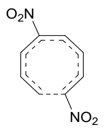

C	0.95374900	-0.87919300	1.52080600
C	0.06579600	-1.70491000	0.88299500
C	-1.16314800	-1.29652200	0.32264200
C	-1.66371300	-0.02811100	0.09253300
C	-1.21862600	1.32115900	0.15951800
C	-0.00033600	1.97779000	0.16585000
C	1.32387300	1.51548900	0.01667500
C	1.65461400	0.25380200	-0.38860700
H	0.62954900	0.08142500	1.90183400
H	1.88312200	-1.27489800	1.92260400
H	0.33517400	-2.74759000	0.73227100
H	-1.82910600	-2.09545800	0.01207100
H	-2.06364400	2.00269300	0.18006400
H	-0.08760500	3.05027400	0.32023400
H	1.02972900	-0.39064700	-0.98633100
N	3.06298300	-0.12019100	-0.48601500
O	3.30584100	-1.12781000	-1.12941500
O	3.89059800	0.56172700	0.09495300
H	2.13726100	2.17901600	0.30060200
N	-3.13003300	-0.08709700	-0.30391100
O	-3.51860900	-1.08251400	-0.88388800
O	-3.83762800	0.85974400	-0.01769300

5.3.6 1,6-substituted

1-CH_3-6-CH_3-substituted

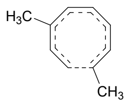

C	0.27180900	-0.97077200	1.27880700
C	-0.87733200	-1.25100100	0.58039200
C	-1.72139500	-0.28012800	-0.01120500
C	-1.46418700	1.07925600	-0.17126500
C	-0.37317100	1.98334300	-0.00343000
C	1.01045000	1.93387000	0.11963400
C	1.91665400	0.86089100	0.00216200
C	1.59191400	-0.39042900	-0.47518600
H	0.40587000	0.01309700	1.70924000
H	0.84949200	-1.77672100	1.72462800
H	-1.13578300	-2.29547600	0.40074600
H	-2.35277100	1.62615300	-0.48883500
H	-0.73610900	3.01151700	-0.00813600
H	1.46675600	2.89707500	0.34310500
H	0.70433200	-0.46163600	-1.09499900
C	2.61223700	-1.48216100	-0.64243000
H	3.41786700	-1.38668200	0.09235100
H	2.15934800	-2.47242000	-0.53260100
H	3.05953900	-1.43791900	-1.64349600
H	2.92639200	1.02341700	0.38214700
C	-3.05904600	-0.80032700	-0.50609000
H	-3.63643000	-1.25181300	0.30825700
H	-3.66376700	-0.00777100	-0.95350100
H	-2.91233000	-1.57606900	-1.26724200

1-CH_3-6-NH_2-substituted

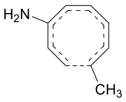

C	0.25874800	-0.98127300	1.25834300
C	-0.87336800	-1.29712300	0.55148300
C	-1.73725200	-0.33423400	-0.02632200
C	-1.52141500	1.04106800	-0.15579900
C	-0.44900000	1.95870500	-0.00345500
C	0.93678800	1.94692600	0.12896300
C	1.87740800	0.90253600	0.01487200
C	1.61374300	-0.35739100	-0.47418800
H	0.36036900	0.00350100	1.69610600
H	0.86556200	-1.77087400	1.69341100
H	-1.08857200	-2.34455000	0.33914000
H	-2.44579700	1.55926100	-0.41473100
H	-0.83618000	2.97808900	0.00036500
H	1.36171700	2.92147400	0.36297800
H	0.73594000	-0.46899700	-1.10170200
C	2.68009900	-1.40647600	-0.62833300
H	3.47707200	-1.27150800	0.10996000
H	2.26894500	-2.41444900	-0.50982900
H	3.13255800	-1.35667900	-1.62697400
H	2.87664000	1.10074200	0.40714800
N	-2.99805200	-0.79678500	-0.45390000
H	-3.10319400	-1.80307200	-0.45306100
H	-3.33320100	-0.39186300	-1.31889900

1-CH_3-6-OH-substituted

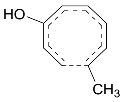

C	0.28409000	-0.99641300	1.24305300
C	-0.85051500	-1.33525800	0.54664800
C	-1.71386500	-0.38183300	-0.02912200
C	-1.55064400	0.99481400	-0.16851100
C	-0.51054600	1.94659000	0.00463200
C	0.87374700	1.96325800	0.14333600
C	1.83523100	0.94002700	0.02437900
C	1.59630800	-0.32612100	-0.46751700
H	0.36000000	-0.01605200	1.69520100
H	0.90459000	-1.77633400	1.67616800
H	-1.07931900	-2.37595300	0.32929400
H	-2.48450700	1.48548600	-0.45282500
H	-0.92593300	2.95445700	0.01308700
H	1.27664100	2.94457100	0.38752500
H	0.72876200	-0.44981200	-1.10682300
C	2.68934500	-1.34632800	-0.63141700
H	3.47436000	-1.20939300	0.11909700
H	2.30012100	-2.36544900	-0.54169400
H	3.15057700	-1.26088700	-1.62332100
H	2.82964700	1.15675600	0.41841200
O	-2.88533100	-0.94241900	-0.48569000
H	-3.37119800	-0.30045200	-1.02148300

1-CH_3-6-F-substituted

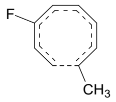

C	0.26589800	-1.04118900	1.25152100
C	-0.86358200	-1.35273700	0.53567400
C	-1.68591900	-0.37122100	-0.04143600
C	-1.55391900	0.99251600	-0.21097200
C	-0.53159400	1.95759900	0.00184100
C	0.84631900	1.96800500	0.17401400
C	1.81149800	0.94510900	0.05526000
C	1.58620600	-0.30950300	-0.46664800
H	0.35264500	-0.06740100	1.71465200
H	0.87672800	-1.83469700	1.67276300
H	-1.11225000	-2.38508900	0.30153700
H	-2.48867600	1.43299700	-0.55489900
H	-0.95264200	2.96260200	0.00495800
H	1.24781600	2.94496000	0.43783300
H	0.71684400	-0.43136300	-1.10434900
C	2.68575100	-1.31958000	-0.64371900
H	3.46913700	-1.18934800	0.10941200
H	2.30366000	-2.34263600	-0.57347200
H	3.14621500	-1.21249300	-1.63380400
H	2.80001900	1.15817700	0.46506000
F	-2.85816100	-0.87218900	-0.53032200

1-CH_3-6-CHO-substituted

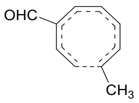

C	0.66411100	-1.03025800	1.35818500
C	-0.51172000	-1.28014000	0.70563200
C	-1.37334800	-0.28623600	0.17130300
C	-1.16826400	1.07861400	-0.00907500
C	-0.09055600	1.99774000	0.09222800
C	1.29783900	1.93975300	0.12856700
C	2.19300100	0.86794400	-0.06828600
C	1.84611800	-0.36797000	-0.56250500
H	0.85440700	-0.04853600	1.77080900
H	1.26296200	-1.84954500	1.74702400
H	-0.79663200	-2.31729900	0.52291600
H	-2.10011800	1.57802100	-0.27723300
H	-0.45700700	3.02377900	0.10982300
H	1.77313900	2.89805200	0.33314900
H	0.90221500	-0.44384600	-1.09284000
C	2.85187200	-1.45034700	-0.82672300
H	3.71630500	-1.36306700	-0.16175600
H	2.41129200	-2.44349800	-0.69955600
H	3.21080200	-1.38488700	-1.86173500
H	3.22740200	1.02854200	0.23768700
C	-2.71798400	-0.78881100	-0.23254200
H	-2.84395600	-1.88822300	-0.14263000
O	-3.63840300	-0.10890300	-0.62829600

1-CH_3-6-CN-substituted

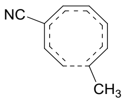

C	0.46075200	-0.93999900	1.37159100
C	-0.73156100	-1.11094300	0.72146300
C	-1.47053700	-0.04629900	0.13825200
C	-1.11332900	1.28244200	-0.09529200
C	0.06853500	2.06790700	-0.01497300
C	1.44055500	1.85502300	0.04522500
C	2.20847000	0.68078000	-0.08535000
C	1.72553600	-0.53048900	-0.52806800
H	0.73205200	0.03561500	1.75243900
H	0.97195600	-1.79425300	1.80647300
H	-1.12405600	-2.11482800	0.57390800
H	-1.96834900	1.88892300	-0.39003000
H	-0.17884400	3.12840900	-0.04247900
H	2.01733100	2.76203000	0.21913100
H	0.79518100	-0.51862100	-1.08678700
C	2.60259600	-1.73319900	-0.72207100
H	3.46483200	-1.71211400	-0.04896500
H	2.04976000	-2.66213500	-0.55406700
H	2.97626400	-1.76156900	-1.75332200
H	3.24901600	0.73412900	0.23622900
C	-2.81621200	-0.39226000	-0.26506400
N	-3.89056800	-0.68333900	-0.58668700

1-CH_3-6-NO_2-substituted

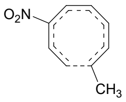

C	0.79961500	-0.95039500	1.35991500
C	-0.40887600	-1.07396100	0.72973500
C	-1.10671900	0.03715000	0.20640100
C	-0.73125100	1.35423700	0.00195400
C	0.47416600	2.10160400	0.07694100
C	1.84032900	1.84536200	0.09689500
C	2.56601300	0.65274900	-0.08756300
C	2.03046400	-0.52675900	-0.55650400
H	1.10677800	0.00313000	1.76848200
H	1.29965700	-1.83296700	1.74842900
H	-0.83323900	-2.05541600	0.54879300
H	-1.58186300	1.98104900	-0.24950900
H	0.25827900	3.16886600	0.08797100
H	2.44906800	2.72840800	0.28474100
H	1.09101200	-0.46383200	-1.09645600
C	2.85852200	-1.75383700	-0.80347800
H	3.73061300	-1.78651300	-0.14379600
H	2.27319300	-2.66637900	-0.65786000
H	3.21515200	-1.76035700	-1.84108500
H	3.61475700	0.66083800	0.21043600
N	-2.51723100	-0.26485600	-0.18968600
O	-2.89235700	-1.42173800	-0.09155800
O	-3.22468800	0.64177100	-0.59320700

1- NH_2-6-CH_3-substituted

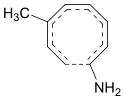

C	0.28760000	-0.94594500	1.27906200
C	-0.88541300	-1.23000600	0.60396600
C	-1.70413900	-0.26712500	-0.01289100
C	-1.42384200	1.09823000	-0.16476600
C	-0.32357900	1.97201200	0.00080700
C	1.06890400	1.87698400	0.12275800
C	1.94351100	0.80086400	-0.02409700
C	1.57125300	-0.46455000	-0.47920200
H	0.41849000	0.03680100	1.71321800
H	0.84715700	-1.75410800	1.74491700
H	-1.16236400	-2.27533100	0.46108900
H	-2.30873400	1.65811400	-0.47504100
H	-0.65075900	3.01146300	-0.01870000
H	1.55301200	2.82456100	0.35292400
H	0.68348800	-0.53891300	-1.09570100
N	2.52013800	-1.44989900	-0.72839200
H	3.31236900	-1.45503400	-0.09654400
H	2.14572400	-2.38064100	-0.86153500
H	2.98178400	0.94881300	0.27369000
C	-3.06597000	-0.73244100	-0.49912900
H	-3.83307800	-0.57201400	0.26854300
H	-3.38161200	-0.19955000	-1.40044600
H	-3.05639600	-1.80301500	-0.72671700

1- NH_2-6-NH_2-substituted

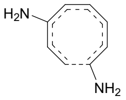

C	0.28145500	-0.95244500	1.25181400
C	-0.88235800	-1.26634300	0.57583100
C	-1.72600700	-0.30791200	-0.01365500
C	-1.48878000	1.07111700	-0.13740600
C	-0.39950800	1.95657000	0.00341000
C	0.99573200	1.89550900	0.11841700
C	1.90241900	0.84283600	-0.02253500
C	1.58824500	-0.43655000	-0.47519400
H	0.38118600	0.02750500	1.70125100
H	0.86658300	-1.74944400	1.70455900
H	-1.12203300	-2.31536700	0.39688700
H	-2.40847000	1.60557700	-0.37977100
H	-0.74977900	2.98861700	0.00274000
H	1.45386000	2.85494700	0.35112500
H	0.71666600	-0.55414200	-1.10747600
N	2.58583300	-1.38585000	-0.70613200
H	3.36400500	-1.34644400	-0.05749600
H	2.24708300	-2.33465000	-0.80735900
H	2.93172500	1.02760300	0.28790100
N	-3.00081800	-0.74145600	-0.45409000
H	-3.13160000	-1.74456900	-0.41197800
H	-3.27151400	-0.38518600	-1.36291600

1- NH_2-6-OH-substituted

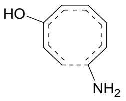

C	0.30872900	-0.96311400	1.23280800
C	-0.85933700	-1.30448200	0.57201800
C	-1.70464200	-0.35810500	-0.01595700
C	-1.52214300	1.02487100	-0.14792000
C	-0.46447100	1.94421800	0.01080700
C	0.93171200	1.91371500	0.13178900
C	1.86042800	0.88404300	-0.01557800
C	1.57390300	-0.40587000	-0.46872400
H	0.37970300	0.01196200	1.69792700
H	0.90960500	-1.74943900	1.68348000
H	-1.10954100	-2.34751500	0.38953500
H	-2.45338800	1.53267700	-0.41049400
H	-0.84330800	2.96578900	0.01250700
H	1.36671500	2.88166500	0.37300300
H	0.71549100	-0.53643400	-1.11645200
N	2.59810400	-1.32439400	-0.70480200
H	3.36870000	-1.27856000	-0.04773500
H	2.28571200	-2.27891800	-0.83268900
H	2.88596600	1.09092900	0.29258600
O	-2.88809300	-0.89662400	-0.48085700
H	-3.33271100	-0.26005800	-1.05665700

1- NH_2-6-F-substituted

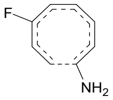

C	0.29160400	-1.00123500	1.24120800
C	-0.87449400	-1.31823400	0.56462400
C	-1.67732500	-0.34642300	-0.02785500
C	-1.52282900	1.02564100	-0.19434700
C	-0.48092500	1.95460600	0.00102600
C	0.91012100	1.91542700	0.16429700
C	1.83958300	0.88622000	0.02007400
C	1.56150600	-0.39412600	-0.46603400
H	0.37030400	-0.03300900	1.71791900
H	0.88152400	-1.80034100	1.68278000
H	-1.14753700	-2.35352600	0.37213900
H	-2.45487600	1.48597300	-0.51994700
H	-0.86084500	2.97517100	-0.01316700
H	1.34388100	2.87809700	0.42846600
H	0.69929300	-0.51606100	-1.11113300
N	2.58480000	-1.30085100	-0.71871600
H	3.36640700	-1.26445700	-0.07490500
H	2.28502600	-2.25228100	-0.88857700
H	2.86067100	1.08758500	0.34519800
F	-2.85787800	-0.82582700	-0.52508000

1- NH_2-6-CHO-substituted

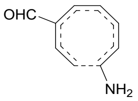

C	0.70499200	-0.98313700	1.33670000
C	-0.51118600	-1.24699300	0.74027900
C	-1.36248500	-0.27112000	0.19051600
C	-1.14023600	1.10865600	0.01676700
C	-0.05113200	1.98799400	0.08280900
C	1.35390400	1.88171200	0.11063600
C	2.20763500	0.81177800	-0.10999500
C	1.80166000	-0.45646500	-0.55453300
H	0.87868300	-0.00504800	1.76509500
H	1.29290600	-1.80089400	1.74657100
H	-0.81234500	-2.28764500	0.60496200
H	-2.07261700	1.62331000	-0.21793700
H	-0.37546800	3.02767400	0.06811500
H	1.85787700	2.82480500	0.31547400
H	0.86336400	-0.52903600	-1.09188400
N	2.72635000	-1.41909900	-0.89317500
H	3.58713400	-1.43143400	-0.36031800
H	2.36811100	-2.34423200	-1.08688900
H	3.26839100	0.95812900	0.09142300
C	-2.70162400	-0.76312800	-0.21487000
H	-2.84025400	-1.86005800	-0.10482100
O	-3.61367400	-0.08220700	-0.63342700

1-NH_2-6-CN-substituted

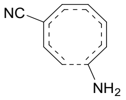

C	0.50896300	-0.90094900	1.34273800
C	-0.72847300	-1.07557400	0.75199200
C	-1.45875100	-0.03055700	0.15745400
C	-1.08182600	1.31048800	-0.07161400
C	0.10722400	2.05325800	-0.02506600
C	1.49000500	1.79018700	0.02862500
C	2.21802300	0.62121800	-0.12496900
C	1.67644000	-0.61606000	-0.51485400
H	0.76464400	0.07178300	1.74136900
H	1.00406800	-1.75335900	1.80068200
H	-1.13868500	-2.07899000	0.65294600
H	-1.93559600	1.93346800	-0.33429200
H	-0.09658400	3.12118400	-0.08514200
H	2.09402200	2.67921900	0.20074700
H	0.75676100	-0.60674800	-1.08786300
N	2.49469100	-1.69262200	-0.78268100
H	3.33116900	-1.78043400	-0.21897400
H	2.03770200	-2.57867700	-0.95033500
H	3.28490900	0.65499600	0.09363000
C	-2.80208700	-0.36645000	-0.25319700
N	-3.87748000	-0.64677900	-0.58295000

1-NH_2-6-NO_2-substituted

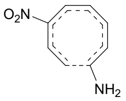

C	0.84946500	-0.91828800	1.32336100
C	-0.40471400	-1.04395300	0.75541600
C	-1.09809800	0.04723400	0.22360400
C	-0.70597300	1.38149900	0.03635800
C	0.50366000	2.08439100	0.07405400
C	1.88156500	1.77819500	0.08144000
C	2.56744700	0.59648700	-0.13075300
C	1.97307700	-0.61267600	-0.54402300
H	1.14008100	0.03041200	1.75472500
H	1.33183000	-1.80136300	1.73379600
H	-0.84753400	-2.02537400	0.61970200
H	-1.55469400	2.02637400	-0.17170200
H	0.33201700	3.15913300	0.05760700
H	2.51609200	2.64247600	0.26892700
H	1.04653900	-0.55220900	-1.10294400
N	2.74895900	-1.70412400	-0.86141000
H	3.59499900	-1.83902300	-0.32209300
H	2.26340700	-2.56748000	-1.06303900
H	3.64068700	0.58910100	0.05607100
N	-2.49575600	-0.24648600	-0.18118200
O	-3.18813400	0.66236800	-0.61026600
O	-2.89116900	-1.39800700	-0.07094000

1-OH-6-CH_3-substituted

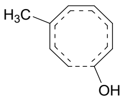

C	0.28137400	-0.98756500	1.23830700
C	-0.89945200	-1.23207600	0.56802900
C	-1.72069200	-0.23840500	-0.00064100
C	-1.42280100	1.12130600	-0.12826400
C	-0.29755900	1.97022000	0.03566500
C	1.09067200	1.85019700	0.11988500
C	1.95161300	0.76062200	-0.05346900
C	1.56703100	-0.49754400	-0.49672300
H	0.43275900	-0.01811800	1.69659700
H	0.84039100	-1.81874800	1.66057100
H	-1.18564200	-2.26858700	0.38461700
H	-2.29837000	1.70692900	-0.41101600
H	-0.60649700	3.01522300	0.05203100
H	1.59744400	2.78652300	0.34591300
H	0.70695300	-0.61307600	-1.14149000
O	2.45754600	-1.51158800	-0.67495400
H	3.28287300	-1.31012700	-0.20789200
H	2.99304600	0.89423800	0.24422100
C	-3.07482800	-0.70774800	-0.50214900
H	-3.66844900	-1.15010800	0.30546000
H	-3.65213800	0.11112200	-0.93857200
H	-2.95487800	-1.47660900	-1.27465400

1-OH-6-NH_2-substituted

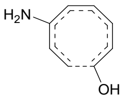

C	0.26901600	-1.00402000	1.21351600
C	-0.89483300	-1.28030600	0.52999600
C	-1.73955300	-0.29271800	-0.01648700
C	-1.48776200	1.08306300	-0.10813900
C	-0.38086200	1.94931200	0.03840000
C	1.01084600	1.86854400	0.12554400
C	1.91063400	0.80722300	-0.04332500
C	1.59902100	-0.46485400	-0.49519300
H	0.38905900	-0.03661500	1.68570600
H	0.85758700	-1.82256000	1.61874900
H	-1.13666400	-2.31946400	0.30601600
H	-2.40116200	1.63730600	-0.32820500
H	-0.71645900	2.98570200	0.07072800
H	1.48686100	2.81846700	0.35983100
H	0.75596300	-0.63477600	-1.14920800
O	2.54541900	-1.43594600	-0.65024800
H	3.35679500	-1.17884500	-0.18625200
H	2.94160600	0.98438000	0.27048100
N	-3.01961800	-0.70535300	-0.44819000
H	-3.15596800	-1.70828800	-0.45019800
H	-3.32269300	-0.29774000	-1.32419800

1-OH-6-OH-substituted

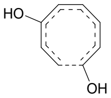

C	0.29295600	-1.01840100	1.19557200
C	-0.87547900	-1.31767400	0.52478700
C	-1.71896800	-0.33889900	-0.02025000
C	-1.51803400	1.04041600	-0.12011000
C	-0.44013000	1.93891000	0.04702700
C	0.95228100	1.88509100	0.14054000
C	1.87184300	0.84437900	-0.03546600
C	1.58484800	-0.43621600	-0.49001300
H	0.38865100	-0.05543800	1.68199100
H	0.89441400	-1.82828700	1.59912000
H	-1.13214100	-2.34981100	0.29770100
H	-2.44221400	1.57046000	-0.36169400
H	-0.80261900	2.96568200	0.08392500
H	1.40785900	2.84239000	0.38493700
H	0.75456000	-0.61560100	-1.15805000
O	2.55567400	-1.38038300	-0.65215800
H	3.35740300	-1.11220700	-0.17767200
H	2.89940300	1.04090000	0.27716200
O	-2.91259800	-0.85104000	-0.47989500
H	-3.36582800	-0.19233900	-1.02351200

1-OH-6-F-substituted

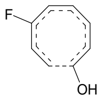

C	0.27292500	-1.05675800	1.20342900
C	-0.89204200	-1.33077800	0.51694300
C	-1.69074400	-0.32441300	-0.03195300
C	-1.51473800	1.04368200	-0.16712700
C	-0.45028200	1.95092800	0.03934100
C	0.93666200	1.88665500	0.17301700
C	1.85526800	0.84339600	-0.00053800
C	1.57068000	-0.42360700	-0.49024000
H	0.37725700	-0.09958500	1.69829500
H	0.86434200	-1.87798300	1.59788000
H	-1.17231500	-2.35418000	0.27918800
H	-2.44023900	1.52968800	-0.47279100
H	-0.81434000	2.97698900	0.06442800
H	1.39301300	2.83748100	0.44080600
H	0.73418500	-0.58803400	-1.15529700
O	2.53462400	-1.36603200	-0.67137300
H	3.34206600	-1.10919800	-0.19989400
H	2.87803900	1.02950200	0.33230800
F	-2.88504200	-0.77233900	-0.51901900

1-OH-6-CHO-substituted

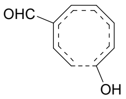

C	0.68424600	-1.04691500	1.31230700
C	-0.51920800	-1.27806400	0.69109000
C	-1.36644000	-0.27112500	0.17822100
C	-1.13210900	1.10067600	0.03259700
C	-0.02921600	1.97439600	0.12674400
C	1.36468200	1.85977500	0.13257300
C	2.21767700	0.78161300	-0.11753200
C	1.81333500	-0.46230900	-0.58398700
H	0.87620300	-0.07842900	1.75586200
H	1.27506900	-1.88230700	1.67799500
H	-0.81911400	-2.31028800	0.50464200
H	-2.05649100	1.63109700	-0.19876400
H	-0.34951200	3.01496700	0.15646700
H	1.88036600	2.79461000	0.34466100
H	0.88851100	-0.57349600	-1.13377600
O	2.68339400	-1.45651100	-0.86622400
H	3.54938700	-1.26509200	-0.47318000
H	3.27691800	0.91756300	0.10406300
C	-2.71847100	-0.73947200	-0.23058000
H	-2.86576600	-1.83771500	-0.15952200
O	-3.62621300	-0.03378400	-0.61215800

1-OH-6-CN-substituted

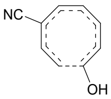

C	0.48562200	-0.96329500	1.31790300
C	-0.73564600	-1.10973500	0.70216400
C	-1.46314900	-0.03285700	0.14777000
C	-1.07796600	1.30147800	-0.05088100
C	0.12260100	2.03966200	0.02006600
C	1.49488900	1.77194100	0.04989900
C	2.22544800	0.59650600	-0.13308300
C	1.69230000	-0.61957200	-0.54685500
H	0.75762700	-0.00147100	1.73324300
H	0.98585100	-1.83566700	1.72863300
H	-1.14243300	-2.10740200	0.55131700
H	-1.92373100	1.93836800	-0.30551300
H	-0.07941100	3.10944800	0.00510700
H	2.10683200	2.65360000	0.23036000
H	0.78276100	-0.65283600	-1.13123200
O	2.45286500	-1.71737300	-0.75515000
H	3.32110900	-1.61040800	-0.33576000
H	3.28906900	0.62452900	0.10656000
C	-2.81590700	-0.34397000	-0.25619400
N	-3.89853500	-0.60287500	-0.57803700

1-OH-6-NO_2-substituted

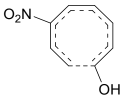

C	0.82400300	-0.98213900	1.29944600
C	-0.41112700	-1.08144900	0.70283300
C	-1.10122700	0.03899600	0.21155000
C	-0.70497800	1.36430900	0.04924000
C	0.51413200	2.06808400	0.11475500
C	1.88008000	1.76323900	0.10264800
C	2.57412500	0.57748900	-0.13550300
C	1.99969400	-0.61144600	-0.57733000
H	1.12933100	-0.04507400	1.74686300
H	1.31237200	-1.88241100	1.66093700
H	-0.84821500	-2.05631800	0.51503800
H	-1.54777800	2.01770600	-0.15680700
H	0.34064700	3.14224800	0.14204900
H	2.51917900	2.62213200	0.29816900
H	1.08218400	-0.60280700	-1.14998000
O	2.72680300	-1.72014700	-0.83136300
H	3.60203000	-1.65345900	-0.41791300
H	3.64326200	0.56970100	0.07912300
N	-2.51248600	-0.23361500	-0.18377100
O	-2.91259000	-1.38293800	-0.09098400
O	-3.20094100	0.69072100	-0.58226700

1-F-6-CH_3-substituted

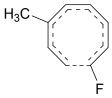

C	0.24069500	-1.04348600	1.24301700
C	-0.92829400	-1.24062700	0.55123400
C	-1.72231800	-0.21068200	-0.00946700
C	-1.39217800	1.13618900	-0.12969900
C	-0.24900100	1.96781600	0.05800300
C	1.13065800	1.83151700	0.14000600
C	1.98223100	0.72489700	-0.04269100
C	1.57471400	-0.49623600	-0.51057800
H	0.43783900	-0.07878700	1.69378200
H	0.78696900	-1.89205700	1.64649000
H	-1.24318600	-2.26455000	0.34635000
H	-2.24987400	1.74249300	-0.42240600
H	-0.54756500	3.01523100	0.09970400
H	1.65107400	2.75707300	0.37708600
H	0.72762300	-0.64662700	-1.16508300
F	2.49471200	-1.47763800	-0.62971600
H	3.01978000	0.80978900	0.27309200
C	-3.08844700	-0.64359600	-0.50930900
H	-3.69079900	-1.06867700	0.30108400
H	-3.64455000	0.18865700	-0.94730000
H	-2.98807800	-1.41856200	-1.27845100

1-F-6-NH_2-substituted

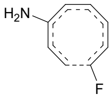

C	0.22606000	-1.05367700	1.21976200
C	-0.92725700	-1.28622500	0.51709900
C	-1.74458400	-0.26369200	-0.02588600
C	-1.45580200	1.10130100	-0.11282200
C	-0.33001400	1.94707900	0.05611400
C	1.05471200	1.84860900	0.14736400
C	1.94444200	0.77053400	-0.02969400
C	1.60597200	-0.46732800	-0.50547100
H	0.39039800	-0.08698600	1.67976100
H	0.80171900	-1.88741200	1.61204300
H	-1.19817300	-2.31450200	0.27744900
H	-2.35090400	1.67970200	-0.34478900
H	-0.65255100	2.98710700	0.10507700
H	1.54418600	2.78846000	0.39215900
H	0.77705100	-0.66869300	-1.16848300
F	2.57989900	-1.40215100	-0.60885600
H	2.97473100	0.89850400	0.29728200
N	-3.02771200	-0.64685100	-0.45547600
H	-3.18914700	-1.64474000	-0.49118300
H	-3.36358600	-0.18373100	-1.29006500

1-F-6-OH-substituted

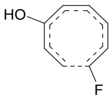

C	0.24897700	-1.07064500	1.20400900
C	-0.90956700	-1.32212600	0.51259500
C	-1.72326500	-0.30583000	-0.02832000
C	-1.48281600	1.06246100	-0.12459200
C	-0.38400800	1.94037500	0.06583000
C	1.00084100	1.86569800	0.16142100
C	1.90796600	0.80474500	-0.02240900
C	1.58808500	-0.43892900	-0.50262000
H	0.39188100	-0.10718200	1.67734000
H	0.83770500	-1.89693500	1.59250700
H	-1.19744700	-2.34176400	0.26764700
H	-2.38779800	1.61759500	-0.38101600
H	-0.73234400	2.97132100	0.12149500
H	1.47280800	2.81173700	0.41648200
H	0.77035100	-0.64509900	-1.17840400
F	2.57897700	-1.35286900	-0.60963000
H	2.93569000	0.94712100	0.30613300
O	-2.92484300	-0.78636600	-0.49086000
H	-3.38016700	-0.10454100	-1.00411000

1-F-6-F-substituted

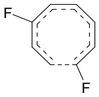

C	0.22814100	-1.11455500	1.20615400
C	-0.92709200	-1.33570700	0.49959900
C	-1.69467400	-0.29006600	-0.03929900
C	-1.47846000	1.06706200	-0.16812000
C	-0.39252700	1.95402900	0.06169400
C	0.98630400	1.86817700	0.19218700
C	1.89186300	0.80329100	0.00988300
C	1.57464800	-0.42574800	-0.50431200
H	0.38245500	-0.15943100	1.69165000
H	0.80498400	-1.95457600	1.58196600
H	-1.23948100	-2.34412300	0.23931600
H	-2.38637000	1.57897300	-0.48350100
H	-0.74333600	2.98393900	0.11084800
H	1.46017000	2.80715700	0.46956600
H	0.75338500	-0.61670100	-1.18117200
F	2.55832000	-1.34162100	-0.62300500
H	2.91425900	0.93444100	0.35823800
F	-2.90001800	-0.70155500	-0.52517600

1-F-6-CHO-substituted

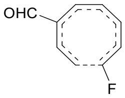

C	0.65853000	-1.11888300	1.30910400
C	-0.53754000	-1.30569100	0.67166600
C	-1.36483100	-0.26670600	0.17338100

C	-1.11067200	1.09510000	0.03752700
C	0.00283100	1.96590000	0.16185800
C	1.38755800	1.85027800	0.15878700
C	2.24110900	0.76305000	-0.11102000
C	1.82171000	-0.44255900	-0.60314000
H	0.89235100	-0.15826600	1.74978700
H	1.23912700	-1.97139700	1.65023800
H	-0.86161500	-2.32614800	0.46303700
H	-2.02404300	1.63887200	-0.20644800
H	-0.31880900	3.00454200	0.22751000
H	1.90933300	2.77851300	0.38260200
H	0.91009100	-0.59251400	-1.16546200
F	2.73499400	-1.40017000	-0.83160600
H	3.29772800	0.85787000	0.12802200
C	-2.73027000	-0.70672400	-0.23985300
H	-2.89911600	-1.80204300	-0.17878500
O	-3.62131800	0.02119000	-0.61448800

1-F-6-CN-substituted

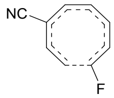

C	0.44889700	-1.02440300	1.32531300
C	-0.75822100	-1.13064800	0.68897600
C	-1.46026100	-0.02296100	0.14348000
C	-1.05274400	1.29801400	-0.04775700
C	0.16064700	2.02946700	0.05063900
C	1.52291900	1.75730100	0.07192600
C	2.25040100	0.56923700	-0.12641200
C	1.70294900	-0.60636300	-0.56719800
H	0.76219700	-0.07152500	1.73229200
H	0.94028800	-1.91176000	1.71367800
H	-1.18822200	-2.11486500	0.51640500
H	-1.88379500	1.94824500	-0.31643500
H	-0.03934300	3.09972900	0.06753800
H	2.14245700	2.63120700	0.26174400
H	0.79879600	-0.67696200	-1.15669800
F	2.50387400	-1.67231000	-0.72018900
H	3.30652200	0.55509000	0.13270100
C	-2.82002900	-0.30416100	-0.26364100
N	-3.90587300	-0.54303900	-0.58878200

1-F-6-NO_2-substituted

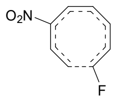

C	0.79106400	-1.03376000	1.31475300
C	-0.42817700	-1.10478000	0.69736800
C	-1.09631400	0.03895400	0.20868000
C	-0.68387400	1.34952000	0.04061300
C	0.54557100	2.05367900	0.12807300
C	1.90173800	1.75224800	0.11014000
C	2.59841300	0.55457500	-0.13254900
C	2.01559800	-0.59687700	-0.59270500
H	1.13082200	-0.10067500	1.74558000

H	1.27582300	-1.94182900	1.66107500
H	-0.88170400	-2.06871300	0.49408600
H	-1.51619400	2.00987900	-0.18565300
H	0.36830200	3.12654400	0.17802500
H	2.54416800	2.60802300	0.30652900
H	1.10242200	-0.63253300	-1.17135200
F	2.78692500	-1.67704400	-0.78181000
H	3.65854200	0.51002800	0.10555600
N	-2.51880800	-0.21029100	-0.18754400
O	-3.20895000	0.73417400	-0.52750500
O	-2.91567100	-1.36250400	-0.15136800

1-CHO-6-CH_3-substituted

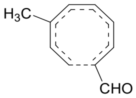

C	0.13375500	-0.96081800	1.21055400
C	-0.99669100	-1.34523500	0.53153800
C	-1.96527200	-0.46613100	-0.00612900
C	-1.89711300	0.92368700	-0.12787200
C	-0.94696400	1.96943700	0.03530200
C	0.43459800	2.13371100	0.13110600
C	1.48095300	1.21483000	-0.03199800
C	1.32355800	-0.06501400	-0.52722500
H	0.18807500	0.02423000	1.65615000
H	0.81055700	-1.70842100	1.61588000
H	-1.14487600	-2.40741500	0.33529200
H	-2.86337100	1.34721500	-0.40245200
H	-1.45931700	2.93156800	0.06464400
H	0.74038100	3.14978800	0.37318400
H	0.46824600	-0.29219400	-1.15175800
C	2.47884500	-0.98258800	-0.61322900
H	2.32007600	-1.89831000	-1.21696400
O	3.53986100	-0.79990600	-0.05292900
H	2.47446600	1.50095900	0.31074900
C	-3.23889200	-1.13612100	-0.48808900
H	-3.00945300	-1.88071600	-1.25930200
H	-3.74157300	-1.66296700	0.33020600
H	-3.94276100	-0.41903500	-0.91594400

1-CHO-6-NH_2-substituted

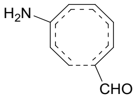

C	0.13127800	-0.97404600	1.16062000
C	-0.99013700	-1.39080900	0.48680400
C	-1.97927900	-0.51813200	-0.02129900
C	-1.94618400	0.88799400	-0.10120700
C	-1.00710600	1.93044800	0.03483400
C	0.37844100	2.11856200	0.13783700
C	1.44064900	1.22716000	-0.02717400
C	1.33054700	-0.06083800	-0.53048500
H	0.15128700	0.00237900	1.62861600
H	0.83288200	-1.70519200	1.55218700

H	-1.09887400	-2.44494100	0.23557600
H	-2.93653000	1.29314700	-0.31324800
H	-1.52952000	2.88708100	0.06795300
H	0.66021400	3.13789500	0.39314000
H	0.49948600	-0.30836800	-1.17945700
C	2.51922100	-0.92667700	-0.61006500
H	2.41517600	-1.82793700	-1.24731500
O	3.55650500	-0.72644800	-0.00853700
H	2.42605900	1.54068900	0.31635600
N	-3.11803400	-1.14363700	-0.53253100
H	-3.42206600	-1.98083500	-0.05299200
H	-3.88850200	-0.53884300	-0.78398400

1-CHO-6-OH-substituted

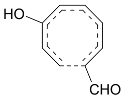

C	0.13643900	-1.00297600	1.16467600
C	-0.98332800	-1.42503800	0.49148200
C	-1.95816700	-0.54839900	-0.02212400
C	-1.96021000	0.84831700	-0.11736900
C	-1.04687500	1.91474700	0.04233300
C	0.33172600	2.11871900	0.15065200
C	1.40718500	1.23889000	-0.01601800
C	1.31559400	-0.04373200	-0.52949200
H	0.15175500	-0.02754100	1.63400600
H	0.84325800	-1.73101500	1.55237100
H	-1.12269400	-2.47683000	0.25468000
H	-2.95834800	1.22720600	-0.34738900
H	-1.58857200	2.85998000	0.08023900
H	0.60170200	3.14007900	0.41041000
H	0.48601800	-0.30211400	-1.17590000
C	2.51946600	-0.89311000	-0.61544900
H	2.42794700	-1.79474200	-1.25323200
O	3.55374600	-0.67340700	-0.01804000
H	2.38649100	1.56166300	0.33588900
O	-3.07116100	-1.21318300	-0.46275100
H	-3.65921800	-0.60847100	-0.93688600

1-CHO-6-F-substituted

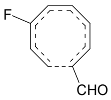

C	0.13563900	-1.06234100	1.16664000
C	-0.98687200	-1.44834900	0.47777300
C	-1.92684300	-0.53786100	-0.03052400
C	-1.96341200	0.84031100	-0.15941100
C	-1.07219600	1.92365400	0.04097700
C	0.29877400	2.12408200	0.18223900
C	1.38025100	1.24442400	0.01652000
C	1.30034000	-0.02113300	-0.52940400
H	0.16944000	-0.09765300	1.65543100
H	0.83417700	-1.80992500	1.53123100
H	-1.15093900	-2.49190000	0.22101400
H	-2.95932900	1.16608300	-0.45497000

H	-1.62422000	2.86248400	0.07396800
H	0.56764500	3.14138700	0.45912900
H	0.46869700	-0.27593100	-1.17523100
C	2.51067000	-0.86530900	-0.62650600
H	2.42590100	-1.75532200	-1.28063000
O	3.54057000	-0.64733000	-0.02327400
H	2.35314500	1.56133200	0.39060400
F	-3.05190900	-1.14519800	-0.49601900

1-CHO-6-CHO-substituted

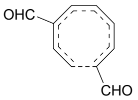

C	0.47548500	-1.00174800	1.30604200
C	-0.68915700	-1.31240900	0.65822400
C	-1.63047600	-0.37655900	0.15842700
C	-1.55980200	1.00491300	0.00208200
C	-0.59142300	2.03743200	0.11761000
C	0.79455200	2.14790400	0.15544400
C	1.79955500	1.18636100	-0.05667100
C	1.57749700	-0.07160000	-0.56643800
H	0.61815600	-0.01896700	1.73540100
H	1.13462300	-1.78798200	1.66402600
H	-0.90443600	-2.36295700	0.45982900
H	-2.53965100	1.40877800	-0.25556500
H	-1.07673400	3.01245800	0.15729100
H	1.15309700	3.15115000	0.37867000
H	0.67022200	-0.27515100	-1.12306200
C	2.69455200	-1.03477400	-0.71333100
H	2.47008000	-1.94600600	-1.30096300
O	3.78973500	-0.87976700	-0.21734900
H	2.81647300	1.43232100	0.24583000
C	-2.93581500	-0.98889700	-0.24900800
H	-2.96332500	-2.09646500	-0.19231600
O	-3.91327400	-0.37784900	-0.61308000

1-CHO-6-CN-substituted

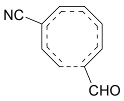

C	0.30462700	-0.89333700	1.31562500
C	-0.87312400	-1.17080100	0.67519900
C	-1.74058700	-0.18236900	0.13941300
C	-1.56794400	1.18919900	-0.06080700
C	-0.51366000	2.13781900	0.02507200
C	0.87659100	2.14146500	0.06672100
C	1.80461700	1.09822600	-0.09602100
C	1.48873900	-0.16022200	-0.55690800
H	0.48848500	0.09542500	1.71518300
H	0.91443600	-1.69940800	1.71381100
H	-1.15642700	-2.20724100	0.50594100
H	-2.50346100	1.67658500	-0.33062200
H	-0.92039400	3.14851300	0.02721100
H	1.30946900	3.12236300	0.25318400

H	0.58130300	-0.31113700	-1.12997600
C	2.53627500	-1.20642100	-0.65869100
H	2.26180300	-2.11125100	-1.23410600
O	3.62636500	-1.11675400	-0.13734100
H	2.83510700	1.27686300	0.20804700
C	-3.03816300	-0.68317700	-0.26476000
N	-4.06935400	-1.09699700	-0.59128600

1-CHO-6-NO_2-substituted

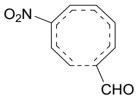

C	0.63116600	-0.82633800	1.34196900
C	-0.56334600	-1.06706400	0.71733600
C	-1.37873600	-0.03707700	0.20345800
C	-1.17463300	1.31415500	-0.01425100
C	-0.08331400	2.22295700	0.03933000
C	1.30562000	2.17091000	0.05391700
C	2.18755300	1.08848200	-0.11206700
C	1.81190600	-0.15936400	-0.55544900
H	0.86075000	0.15502600	1.73617300
H	1.21599200	-1.65459400	1.73195600
H	-0.89154100	-2.08634800	0.54563000
H	-2.10393300	1.82030100	-0.26008300
H	-0.45090200	3.24809100	0.03938400
H	1.78061000	3.13560700	0.22138200
H	0.89251800	-0.27697200	-1.11723400
C	2.81387900	-1.25110800	-0.65820800
H	2.49388300	-2.15138100	-1.21651200
O	3.91377000	-1.19788000	-0.15387800
H	3.22852700	1.22615100	0.17705000
N	-2.75374400	-0.50445400	-0.18803900
O	-2.91535100	-1.70383800	-0.32631800
O	-3.62970100	0.32446700	-0.34951400

1-CN-6-CH_3-substituted

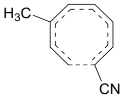

C	0.22417100	-0.80330900	1.32676500
C	-0.82908500	-1.32771100	0.61567300
C	-1.83119100	-0.56499100	-0.02440800
C	-1.86085700	0.81875600	-0.21703200
C	-0.99871300	1.93744800	-0.05636700
C	0.36064800	2.20730900	0.09947200
C	1.48177800	1.36908600	0.02743200
C	1.42846200	0.05062600	-0.39231100
H	0.15345600	0.20239400	1.72260200
H	0.94388500	-1.46402200	1.80223500
H	-0.87081500	-2.40730500	0.47210300
H	-2.83720700	1.15443000	-0.56633400
H	-1.57722000	2.86027700	-0.10305600
H	0.58247300	3.25277200	0.30247800
H	0.62460600	-0.26456300	-1.04501800
C	2.62240500	-0.74556200	-0.44749200

N	3.57273700	-1.40871200	-0.46874300
H	2.43090400	1.76077200	0.38654000
C	-3.01564800	-1.35939000	-0.54170000
H	-3.52296500	-1.88611400	0.27363800
H	-3.74681200	-0.72331000	-1.04531700
H	-2.68126500	-2.11792200	-1.25885900

1-CN-6-NH_2-substituted

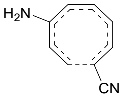

C	0.22417100	-0.80330900	1.32676500
C	-0.82908500	-1.32771100	0.61567300
C	-1.83119100	-0.56499100	-0.02440800
C	-1.86085700	0.81875600	-0.21703200
C	-0.99871300	1.93744800	-0.05636700
C	0.36064800	2.20730900	0.09947200
C	1.48177800	1.36908600	0.02743200
C	1.42846200	0.05062600	-0.39231100
H	0.15345600	0.20239400	1.72260200
H	0.94388500	-1.46402200	1.80223500
H	-0.87081500	-2.40730500	0.47210300
H	-2.83720700	1.15443000	-0.56633400
H	-1.57722000	2.86027700	-0.10305600
H	0.58247300	3.25277200	0.30247800
H	0.62460600	-0.26456300	-1.04501800
C	2.62240500	-0.74556200	-0.44749200
N	3.57273700	-1.40871200	-0.46874300
H	2.43090400	1.76077200	0.38654000
C	-3.01564800	-1.35939000	-0.54170000
H	-3.52296500	-1.88611400	0.27363800
H	-3.74681200	-0.72331000	-1.04531700
H	-2.68126500	-2.11792200	-1.25885900

1-CN-6-OH-substituted

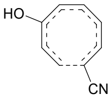

C	0.22899600	-0.84998800	1.28313100
C	-0.80295900	-1.41585300	0.57309900
C	-1.81615100	-0.66065700	-0.04453600
C	-1.92873900	0.72593400	-0.20957000
C	-1.11505800	1.86852000	-0.04610200
C	0.23434000	2.18980600	0.12837200
C	1.38996300	1.40445700	0.05344400
C	1.41751200	0.08802300	-0.38468200
H	0.10945200	0.14024400	1.70538600
H	0.97996400	-1.48580300	1.74290000
H	-0.82417900	-2.48596100	0.38425900
H	-2.93737400	1.00932700	-0.51689900
H	-1.72978700	2.76767200	-0.08312900
H	0.40835000	3.23947000	0.35282800
H	0.64836600	-0.26278200	-1.06002800
C	2.65944300	-0.62980800	-0.43983700
N	3.64821900	-1.23464700	-0.45776900

H	2.31552700	1.83996000	0.42442100
O	-2.83455500	-1.44058000	-0.51954700
H	-3.43550300	-0.91756500	-1.06888200

1-CN-6-F-substituted

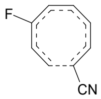

C	0.23006700	-0.90370600	1.28780400
C	-0.80088700	-1.44219500	0.55652800
C	-1.78540800	-0.65457700	-0.05663100
C	-1.93241200	0.70913700	-0.25471600
C	-1.14703400	1.86960300	-0.05078000
C	0.19210000	2.19212100	0.15923900
C	1.35638600	1.41194300	0.08847100
C	1.40224200	0.11164600	-0.38386300
H	0.11848600	0.07815800	1.72987500
H	0.97907200	-1.55468400	1.72866200
H	-0.83865300	-2.50760400	0.34491600
H	-2.93094900	0.93572000	-0.62484200
H	-1.77316500	2.76006200	-0.09294000
H	0.36003500	3.23868300	0.40288300
H	0.63412400	-0.23850600	-1.06152000
C	2.65116300	-0.59446800	-0.44413400
N	3.64659600	-1.18731500	-0.46945000
H	2.27249200	1.84506700	0.48442600
F	-2.81610300	-1.38252300	-0.55964700

1-CN-6-CHO-substituted

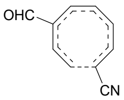

C	0.56438100	-0.81136200	1.43070800
C	-0.55518100	-1.24248400	0.76908400
C	-1.52266600	-0.39822600	0.17240400
C	-1.51049700	0.97360200	-0.06810600
C	-0.58948100	2.04958000	0.01806000
C	0.78941100	2.22131800	0.09413900
C	1.84580100	1.30259000	-0.03163500
C	1.68629900	-0.00302500	-0.44913500
H	0.61493100	0.20295900	1.80554300
H	1.26096900	-1.52740700	1.85718400
H	-0.69630400	-2.31501400	0.63285600
H	-2.49549600	1.31646500	-0.38655300
H	-1.11218500	3.00485600	-0.02175400
H	1.09941900	3.25035700	0.26415600
H	0.81106700	-0.28054200	-1.02293000
C	2.81806900	-0.87876800	-0.56588500
N	3.72129900	-1.60047800	-0.64133500
H	2.83804000	1.63154000	0.26869300
C	-2.77676200	-1.10363500	-0.24721300
H	-2.75200300	-2.20483100	-0.11809900
O	-3.76422100	-0.56657200	-0.69053500

1-CN-6-CN-substituted

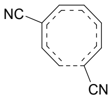

C	0.39061600	-0.73035400	1.42129200
C	-0.74877000	-1.11538700	0.76331400
C	-1.63902100	-0.20880500	0.13463000
C	-1.51513100	1.15618400	-0.14110200
C	-0.50049200	2.14612700	-0.07425100
C	0.88707100	2.20638300	0.01732700
C	1.86488200	1.20152800	-0.05701100
C	1.60440400	-0.10175300	-0.43424800
H	0.49328900	0.28808100	1.77381600
H	1.03114900	-1.47609900	1.88284600
H	-0.96781100	-2.17441200	0.64901400
H	-2.45734300	1.58826600	-0.47392800
H	-0.94088600	3.13933200	-0.15184900
H	1.27715000	3.21188800	0.15882700
H	0.72869400	-0.32370500	-1.03113900
C	2.66518100	-1.06792800	-0.50041500
N	3.50910800	-1.86083900	-0.53299200
H	2.87582800	1.45777100	0.25159300
C	-2.89621100	-0.79088200	-0.28759200
N	-3.89699900	-1.26513200	-0.62570000

1-CN-6-NO_2-substituted

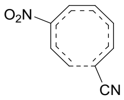

C	0.71281600	-0.67963800	1.43802800
C	-0.45693900	-1.00304600	0.79935100
C	-1.28712800	-0.03228800	0.20630800
C	-1.11066000	1.31357400	-0.06991300
C	-0.04229100	2.24684200	-0.02159400
C	1.34750400	2.22876700	0.03704400
C	2.26473700	1.17011700	-0.06279100
C	1.91845600	-0.11314900	-0.43939700
H	0.87806800	0.32779300	1.79846300
H	1.32158100	-1.46351700	1.87875500
H	-0.73871900	-2.04235200	0.67190000
H	-2.04180300	1.78423600	-0.37262400
H	-0.42964900	3.26275800	-0.08154300
H	1.79622100	3.21019700	0.17380300
H	1.01990800	-0.27768500	-1.02093500
C	2.91838100	-1.14066200	-0.53303300
N	3.71415100	-1.98041800	-0.58901600
H	3.29498800	1.36499500	0.22599800
N	-2.63374200	-0.56170800	-0.20728800
O	-3.50036500	0.23074100	-0.52601600
O	-2.78122400	-1.77007200	-0.20194700

1-NO_2-6-CH_3-substituted

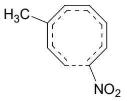

C	-0.06500300	-0.62310100	1.43684300
C	-1.06533000	-1.24931100	0.74226200
C	-2.10465200	-0.58723700	0.04009200
C	-2.21094100	0.77099100	-0.25388900
C	-1.41473500	1.95043500	-0.17067900
C	-0.07652600	2.30799900	-0.04162800
C	1.09117100	1.52570700	-0.04137200
C	1.09985100	0.19357900	-0.36204800
H	-0.18464500	0.40671500	1.75136900
H	0.69386500	-1.20434500	1.95483000
H	-1.03820600	-2.33551200	0.66082200
H	-3.19954900	1.02327800	-0.63712800
H	-2.04727000	2.83185400	-0.27852700
H	0.08853300	3.37645500	0.07571500
H	0.38413000	-0.27715800	-1.01719600
N	2.35163300	-0.54537500	-0.30953300
O	2.38404700	-1.60034900	-0.92550800
O	3.26984600	-0.09886200	0.36097900
H	2.02280100	1.97182900	0.29796100
C	-3.23140800	-1.48706200	-0.42850900
H	-3.70001100	-2.00315800	0.41621600
H	-4.00462500	-0.93245700	-0.96419000
H	-2.84215200	-2.25819100	-1.10334200

1-NO_2-6-NH_2-substituted

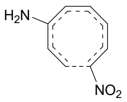

C	-0.07805800	-0.66703300	1.37387900
C	-1.06685400	-1.31195500	0.68328200
C	-2.12952000	-0.64395400	0.02133800
C	-2.27052600	0.73689800	-0.21856600
C	-1.47481000	1.89945900	-0.16136600
C	-0.13279700	2.27648100	-0.02521100
C	1.04682000	1.52548300	-0.03538500
C	1.11171600	0.19059200	-0.36692400
H	-0.22574200	0.35054700	1.71628800
H	0.70352400	-1.23730500	1.86895200
H	-1.00148600	-2.38883800	0.53854400
H	-3.28737500	0.98341500	-0.52543700
H	-2.11099700	2.77924800	-0.26146800
H	0.00830700	3.34573500	0.11088600
H	0.43157500	-0.29931000	-1.04519200
N	2.38539300	-0.49304600	-0.29838700
O	2.46996600	-1.55438200	-0.90310400
O	3.27973700	-0.01006200	0.38342400
H	1.96583100	1.99989400	0.30141400
N	-3.13337800	-1.46535800	-0.46695700
H	-3.32425700	-2.32064300	0.03635100
H	-3.95693600	-1.02418800	-0.85176100

1-NO_2-6-OH-substituted

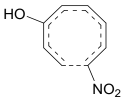

C	-0.07226100	-0.69372500	1.38632500
C	-1.05682900	-1.34592800	0.69499400
C	-2.10238600	-0.67572200	0.02185600
C	-2.27531200	0.68652900	-0.24120500
C	-1.51312200	1.87583200	-0.15940000
C	-0.18246400	2.26960500	-0.01562900
C	1.00945100	1.53113700	-0.02223400
C	1.09221300	0.20245400	-0.36175400
H	-0.22837100	0.32234400	1.72831500
H	0.71176700	-1.25847100	1.88360200
H	-1.02227500	-2.42413300	0.56422500
H	-3.29290600	0.90206400	-0.57274600
H	-2.16925700	2.74072100	-0.25620800
H	-0.05429100	3.34007300	0.12378600
H	0.41840000	-0.29711200	-1.03940200
N	2.38164200	-0.46282000	-0.30215400
O	2.48166400	-1.51150300	-0.92284000
O	3.26619000	0.02889700	0.38354900
H	1.91954100	2.01436400	0.32567500
O	-3.07831300	-1.53271700	-0.39617100
H	-3.70617600	-1.07861300	-0.97618100

1-NO_2-6-F-substituted

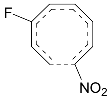

C	-0.06383500	-0.75198600	1.39341200
C	-1.05137700	-1.37580800	0.68030100
C	-2.06856700	-0.66991900	0.01095800
C	-2.27287700	0.66504600	-0.28465000
C	-1.54091200	1.87522200	-0.16328500
C	-0.22104000	2.27236000	0.01464300
C	0.97818200	1.53698100	0.01252600
C	1.07436900	0.22241300	-0.36075700
H	-0.21087300	0.25627200	1.76010400
H	0.71709600	-1.33604200	1.87267400
H	-1.03493600	-2.45139700	0.52571600
H	-3.27651800	0.81961600	-0.67692800
H	-2.20926600	2.72972400	-0.26333200
H	-0.09764300	3.34083800	0.17351900
H	0.40199900	-0.27296900	-1.04366500
N	2.37064700	-0.43803300	-0.30719500
O	2.47532800	-1.47552000	-0.94331200
O	3.24987100	0.05076100	0.38550100
H	1.87924200	2.01619500	0.38813900
F	-3.06321000	-1.47597700	-0.43803000

1-NO_2-6-CHO-substituted

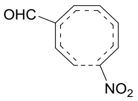

C	0.26736800	-0.58786600	1.55926800
C	-0.80437000	-1.12212800	0.90171700
C	-1.80483100	-0.37381600	0.22669900
C	-1.86208300	0.96924200	-0.12238900
C	-1.00368900	2.10495800	-0.10769700
C	0.35881300	2.35834600	-0.04252400
C	1.46326300	1.48245400	-0.07874600
C	1.35689100	0.15801300	-0.39159100
H	0.26660100	0.45480000	1.85227000
H	0.99671000	-1.23263700	2.04284000
H	-0.88113100	-2.20718000	0.83155200
H	-2.85687400	1.22971200	-0.48591600
H	-1.58122800	3.02241200	-0.21762500
H	0.61506800	3.41215800	0.03981500
H	0.55880900	-0.27438400	-0.97518700
N	2.54996100	-0.68088000	-0.39302500
O	2.45504400	-1.74814700	-0.97757700
O	3.54151500	-0.28957600	0.20054600
H	2.44052000	1.85817000	0.21422600
C	-3.01403000	-1.17730300	-0.15586300
H	-2.92827700	-2.26447100	0.04359600
O	-4.02454800	-0.72525400	-0.63892300

1-NO_2-6-CN-substituted

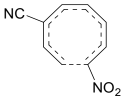

C	0.11418800	-0.51087500	1.51725300
C	-0.97470500	-1.01309700	0.86198400
C	-1.92208600	-0.20770300	0.17118300
C	-1.89174500	1.13683700	-0.19499300
C	-0.95048900	2.20528500	-0.18760100
C	0.42465100	2.37167500	-0.10675300
C	1.47202900	1.42978900	-0.10169500
C	1.29277700	0.10686800	-0.39293100
H	0.14801600	0.53412000	1.80016800
H	0.80662000	-1.17908000	2.02236300
H	-1.11184100	-2.08987400	0.79884800
H	-2.85703100	1.47785500	-0.56490500
H	-1.46376700	3.15666500	-0.32091000
H	0.74406600	3.40894600	-0.03902000
H	0.49543300	-0.28611000	-1.00520000
N	2.43644000	-0.79922000	-0.34162100
O	2.30117700	-1.86736800	-0.91474300
O	3.42526200	-0.45456500	0.28417700
H	2.46305800	1.75010300	0.21005600
C	-3.13377600	-0.90344300	-0.21126300
N	-4.09660100	-1.47066100	-0.51380400

1-NO_2-6-NO_2-substituted

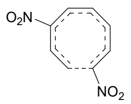

C	0.43355500	-0.45591000	1.52476600
C	-0.69058500	-0.89260700	0.88131900
C	-1.58264700	-0.01075000	0.23057800
C	-1.49989100	1.31968400	-0.12841000
C	-0.50129100	2.33588000	-0.13150400
C	0.88120900	2.42230000	-0.07618000
C	1.87320400	1.42139400	-0.09571800
C	1.61552200	0.11405000	-0.39542700
H	0.53194900	0.58025200	1.82404000
H	1.09795100	-1.16980700	2.00417400
H	-0.89198800	-1.95452600	0.79352600
H	-2.45837100	1.70111000	-0.46928800
H	-0.96421600	3.31470600	-0.24616900
H	1.26114000	3.43887300	-0.00668900
H	0.79271700	-0.22963400	-1.00366400
N	2.71017200	-0.85433300	-0.36538400
O	2.51172700	-1.90672400	-0.94776700
O	3.72158000	-0.56823200	0.25315500
H	2.88506800	1.68172900	0.20478300
N	-2.88908700	-0.65630500	-0.15380500
O	-2.93378800	-1.87166200	-0.12541500
O	-3.82155600	0.05755700	-0.47033800

5.3.7 1,7-substituted

1-CH_3-7-CH_3-substituted

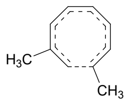

C	0.73381300	0.33319500	1.18533800
C	1.50764300	-0.40983400	0.32829600
C	1.00513900	-1.58863000	-0.28157400
C	-0.28554200	-2.08368000	-0.37842200
C	-1.61003400	-1.64293100	-0.07396900
C	-2.25364800	-0.44192800	0.19044600
C	-1.79697200	0.89290400	0.14044500
C	-0.60036000	1.29322000	-0.40974700
H	-0.13130900	-0.11973800	1.65192400
H	1.14159900	1.21788000	1.67017400
H	1.75893300	-2.21695400	-0.75762700
H	-0.30200600	-3.10051400	-0.77217100
H	-2.30480200	-2.48279800	-0.08454700
H	-3.29511300	-0.55701400	0.48800600
H	-0.13583900	0.61890000	-1.12276700
C	-0.18770700	2.73625100	-0.49458500
H	-0.63305300	3.32390400	0.31432100
H	0.90051700	2.84398800	-0.44157100
H	-0.51203800	3.17083400	-1.44855300
H	-2.41671300	1.64474800	0.63086700
C	2.86573200	0.08356100	-0.11083500
H	3.58932600	-0.73836900	-0.14296000
H	2.82003400	0.51019600	-1.12180600
H	3.25208100	0.85216000	0.56435300

1-CH_3-7-NH_2-substituted

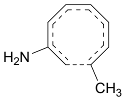

C	0.76302400	0.39422700	1.20951800
C	1.54788400	-0.28058500	0.31369800
C	1.13273400	-1.50907200	-0.28607900
C	-0.11462700	-2.08855800	-0.37590200
C	-1.47183800	-1.75155200	-0.05654200
C	-2.20267000	-0.60438000	0.19618200
C	-1.85536200	0.76779100	0.13557200
C	-0.70453000	1.25712700	-0.42662900
H	-0.04870700	-0.12841200	1.69589600
H	1.10350200	1.32509400	1.65707800
H	1.93414100	-2.08621100	-0.74884500
H	-0.05704000	-3.10399700	-0.76949800
H	-2.09445400	-2.64575500	-0.04654200
H	-3.23254800	-0.79878900	0.49419800
H	-0.16729600	0.61648100	-1.11922900
C	-0.38047800	2.72099200	-0.50254600
H	-0.87517800	3.28028300	0.29768400
H	0.69953300	2.88538300	-0.43438000
H	-0.71117400	3.13538000	-1.46343200
H	-2.53207400	1.46861000	0.62573700
N	2.72964200	0.30717000	-0.17188100
H	3.29866700	0.76105000	0.53256900
H	3.29032000	-0.29524900	-0.76169900

1-CH_3-7-OH-substituted

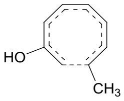

C	0.71550200	0.52583100	1.15588500
C	1.57886300	-0.09067200	0.28841900
C	1.32834100	-1.34070600	-0.32780500
C	0.16300300	-2.08072200	-0.39440700
C	-1.21269600	-1.92358800	-0.04136200
C	-2.08446800	-0.87955100	0.23589500
C	-1.92764900	0.52126900	0.16033700
C	-0.85173900	1.15738500	-0.41566700
H	-0.02412800	-0.08082900	1.65905000
H	0.96866100	1.48027000	1.61360900
H	2.19811900	-1.77627300	-0.81468200
H	0.34395000	-3.07798100	-0.79628700
H	-1.71562600	-2.89019100	-0.02233600
H	-3.06874400	-1.20947100	0.56608000
H	-0.26292200	0.59037000	-1.13010300
C	-0.75616800	2.65318000	-0.52443100
H	-1.28285300	3.14699700	0.29805200
H	0.28622700	2.98688400	-0.52326700
H	-1.20216000	2.99462500	-1.46705700
H	-2.68594000	1.12940500	0.65465100
O	2.71997600	0.52686900	-0.16308600
H	2.96767400	1.23669300	0.44579700

1-CH_3-7-F-substituted

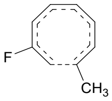

C	-0.57522000	-0.72102700	1.15460000
C	-1.53677200	-0.26878500	0.29968600
C	-1.60946000	0.98301900	-0.33585800
C	-0.65907200	1.98788300	-0.40149400
C	0.71032000	2.15359700	-0.04451200
C	1.79675300	1.33614200	0.24459700
C	1.97508600	-0.06059100	0.16850700
C	1.09539500	-0.94263600	-0.41984400
H	0.02462300	0.00954600	1.67743000
H	-0.66068800	-1.71649300	1.57880500
H	-2.55959000	1.18313000	-0.82566200
H	-1.07306300	2.91240900	-0.80346700
H	0.97950600	3.20932200	-0.02931600
H	2.67443700	1.88502200	0.58373700
H	0.39272700	-0.53618500	-1.14035500
C	1.37644900	-2.41469000	-0.52871200
H	2.00133300	-2.76175100	0.29986800
H	0.45090300	-2.99768900	-0.53714200
H	1.90440500	-2.62986600	-1.46627900
H	2.85338900	-0.46948200	0.66930700
F	-2.49209600	-1.15616000	-0.09208200

1-CH_3-7-CHO-substituted

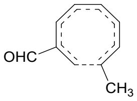

C	0.58897200	0.08039300	1.19732600
C	1.03100800	-0.85858200	0.30469800
C	0.20707700	-1.83981300	-0.32114000
C	-1.16291300	-1.93375700	-0.41292900
C	-2.28995800	-1.12559100	-0.03733000
C	-2.52753100	0.20197100	0.25176700
C	-1.69107500	1.35081200	0.17895600
C	-0.48543600	1.41707100	-0.46383400
H	-0.35823800	-0.05017300	1.70248800
H	1.29531100	0.79748100	1.60827900
H	0.75096300	-2.64625900	-0.81453600
H	-1.48939700	-2.87811500	-0.84846000
H	-3.20093800	-1.72338600	-0.01270800
H	-3.54169100	0.40552200	0.59254500
H	-0.24278000	0.61735200	-1.15735900
C	0.34195800	2.66436500	-0.56359000
H	0.07180800	3.38443900	0.21481500
H	1.40907200	2.43560700	-0.47674200
H	0.18989700	3.14201600	-1.53950300
H	-2.03648700	2.23733000	0.71266300
C	2.44772700	-0.79852900	-0.13464600
H	2.80187600	-1.67537300	-0.71542800
O	3.19895300	0.12544000	0.09228500

1-CH_3-7-CN-substituted

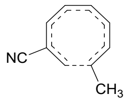

C	0.51975800	0.26139900	1.23600400
C	1.21452000	-0.56659300	0.38790400
C	0.64497600	-1.68511100	-0.28611100
C	-0.67792400	-2.05306000	-0.41910600
C	-1.96173100	-1.50175200	-0.10170100
C	-2.49197600	-0.25451400	0.17471800
C	-1.91599300	1.03978000	0.13747700
C	-0.70346400	1.34868900	-0.42610900
H	-0.39811000	-0.10055500	1.67836500
H	1.01502100	1.09801600	1.71948900
H	1.36181900	-2.35505900	-0.75550400
H	-0.78134300	-3.04626300	-0.85533200
H	-2.72715900	-2.27725900	-0.12347200
H	-3.54054700	-0.27566000	0.46732300
H	-0.27824300	0.62745400	-1.11764500
C	-0.16404400	2.74725700	-0.50652800
H	-0.56814500	3.37769800	0.29126500
H	0.92854000	2.75704100	-0.44364400
H	-0.43457200	3.19949600	-1.46875900
H	-2.46920800	1.83591200	0.63710200
C	2.57544000	-0.21991600	0.05332500
N	3.66493900	0.06601400	-0.21834600

1-CH_3-7-NO_2-substituted

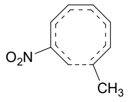

C	0.19533200	0.34104100	1.22242700
C	0.90544200	-0.49726800	0.41429500
C	0.42941100	-1.67215100	-0.21599900
C	-0.87245700	-2.10637900	-0.34139700
C	-2.18185300	-1.60236700	-0.04412800
C	-2.77019000	-0.37253400	0.18224000
C	-2.25925700	0.94885000	0.09545600
C	-1.07170900	1.30507300	-0.49027300
H	-0.69749500	-0.05085900	1.68917000
H	0.65634900	1.22692800	1.64360700
H	1.19326500	-2.31366000	-0.64324900
H	-0.92795500	-3.11920000	-0.73889100
H	-2.90930300	-2.41361100	-0.03013000
H	-3.81664500	-0.43222000	0.47711000
H	-0.60932900	0.58244600	-1.15701400
C	-0.59538400	2.72273300	-0.60925300
H	-1.06369800	3.36483900	0.14279800
H	0.49193200	2.78300300	-0.49840500
H	-0.84347100	3.12008400	-1.60125500
H	-2.85026700	1.73138300	0.57300400
N	2.27337600	-0.06838100	0.04767100
O	3.01931900	-0.89457700	-0.44693000
O	2.57905300	1.09427000	0.26309800

1- NH_2-7-CH_3-substituted

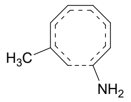

C	0.68516200	0.35026100	1.16363000
C	1.49395200	-0.40285800	0.33185700
C	1.01069800	-1.56922500	-0.29228400
C	-0.28712700	-2.07764800	-0.36746800
C	-1.59599000	-1.63281600	-0.06222000
C	-2.22792900	-0.41086700	0.20030400
C	-1.78410300	0.90861800	0.11686100
C	-0.55388300	1.30369900	-0.41391000
H	-0.15874600	-0.12687100	1.64494600
H	1.09156700	1.22863600	1.66254500
H	1.76333100	-2.18452900	-0.78569900
H	-0.30167200	-3.10098200	-0.74479100
H	-2.30583800	-2.45928400	-0.07742000
H	-3.26639500	-0.51535700	0.51099800
H	-0.08041900	0.65221400	-1.14001700
N	-0.23982000	2.64809400	-0.57464400
H	-0.59345600	3.26304000	0.14901200
H	0.73099600	2.84127400	-0.78587700
H	-2.42940000	1.68482300	0.52818000
C	2.85702500	0.10835300	-0.07238800
H	3.57161100	-0.71513500	-0.17361500
H	2.81446800	0.61125400	-1.04910600
H	3.25586700	0.81916200	0.65706300

1- NH_2-7-NH_2-substituted

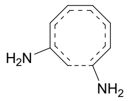

C	0.72847900	0.30790500	1.20803200
C	1.47617400	-0.44406200	0.33052100
C	0.95527000	-1.60598400	-0.29340800
C	-0.35874500	-2.04763400	-0.37243100
C	-1.65343600	-1.55108100	-0.05755000
C	-2.23283300	-0.30683200	0.20184500
C	-1.73427700	0.99745300	0.11658700
C	-0.49454400	1.31731600	-0.43068100
H	-0.11001700	-0.15811500	1.70764000
H	1.15407400	1.19802700	1.66660600
H	1.68965100	-2.25396500	-0.77212400
H	-0.41784400	-3.06720600	-0.75595300
H	-2.39195400	-2.35172900	-0.06399700
H	-3.27529900	-0.36888200	0.51184400
H	-0.04416400	0.62018100	-1.12773800
N	-0.06393100	2.61923800	-0.59695100
H	-0.37315000	3.28114000	0.10461900
H	0.91907900	2.71759200	-0.81875300
H	-2.33858700	1.80394200	0.53037100
N	2.71216600	0.05989200	-0.12648100
H	3.32438200	0.39101300	0.61085200
H	3.20965300	-0.56839200	-0.74682800

1- NH_2-7-OH-substituted

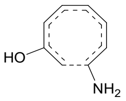

C	0.67017400	0.49628400	1.18781900
C	1.54241500	-0.12325400	0.32779400
C	1.29275900	-1.36360400	-0.29645600
C	0.10494000	-2.08316800	-0.36998800
C	-1.26093600	-1.87050100	-0.05695200
C	-2.09157500	-0.77495900	0.20440900
C	-1.89463800	0.60432400	0.11644100
C	-0.76268700	1.21084200	-0.42817100
H	-0.06853500	-0.11066600	1.69199600
H	0.95546800	1.44109600	1.64127800
H	2.15575700	-1.84070500	-0.76238500
H	0.26838700	-3.09460100	-0.74374900
H	-1.81508400	-2.80793000	-0.06100300
H	-3.09384800	-1.06083300	0.52078500
H	-0.16978000	0.64911400	-1.14038600
N	-0.67217600	2.58156800	-0.58402900
H	-1.13460000	3.13516100	0.12729600
H	0.25168000	2.93348700	-0.80101100
H	-2.66576300	1.25271100	0.53119800
O	2.66642400	0.58650600	-0.02919500
H	3.08744300	0.16435700	-0.79162600

1- NH_2-7-F-substituted

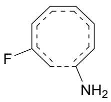

C	-0.58266000	-0.64337900	1.14166900
C	-1.53547600	-0.11195400	0.30689600
C	-1.50520100	1.12120800	-0.34296700
C	-0.45683100	2.04240200	-0.38655900
C	0.91072200	2.05675300	-0.04037900
C	1.90685700	1.10833400	0.24313000
C	1.95026400	-0.27850900	0.13836800
C	0.94808400	-1.07874300	-0.42475300
H	0.05034900	0.04912100	1.67865500
H	-0.75756900	-1.61951500	1.58424900
H	-2.42687700	1.40341400	-0.84538100
H	-0.78310300	3.01200900	-0.76294600
H	1.30656700	3.07131700	-0.03266800
H	2.83729700	1.55422700	0.59159500
H	0.28637400	-0.63166100	-1.15768800
N	1.13213300	-2.43833800	-0.59676700
H	1.67548700	-2.90731500	0.11780600
H	0.31080700	-2.96451700	-0.86519600
H	2.81828700	-0.78594100	0.55863900
F	-2.56190000	-0.93438200	-0.05579200

1- NH_2-7-CHO-substituted

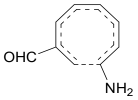

C	0.27220300	0.50696400	1.20867100
C	1.20417300	-0.18699500	0.44494300
C	0.93631800	-1.39809700	-0.21604900
C	-0.26839900	-2.08318400	-0.35653800
C	-1.63435200	-1.82805900	-0.08261800
C	-2.43308600	-0.70596600	0.16294600
C	-2.17145400	0.66186200	0.07482500
C	-0.99986100	1.22232200	-0.44177400
H	-0.51857100	-0.04333900	1.69976200
H	0.57288800	1.43984600	1.68619400
H	1.80584600	-1.87327100	-0.66589400
H	-0.12715300	-3.08779800	-0.75547800
H	-2.22279600	-2.74429500	-0.11715500
H	-3.45369500	-0.94922100	0.45277300
H	-0.41151600	0.62117400	-1.12598100
N	-0.88349300	2.58726700	-0.64705100
H	-1.35275200	3.18110500	0.02601500
H	0.04329100	2.92275700	-0.87544500
H	-2.92738100	1.34430700	0.46338600
C	2.52191500	0.45463100	0.22711000
H	2.62821700	1.45865600	0.69459500
O	3.44791600	-0.03020900	-0.38531300

1-NH_2-7-CN-substituted

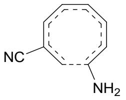

C	0.46383100	0.26502000	1.20836900
C	1.17315000	-0.60422500	0.39040500
C	0.59128600	-1.69229600	-0.29114100
C	-0.75659900	-2.02885000	-0.40069900
C	-2.00025200	-1.42330400	-0.09937600
C	-2.46812000	-0.13424000	0.18157200
C	-1.85253600	1.11604900	0.11908300
C	-0.58482300	1.35414500	-0.42128700
H	-0.43476600	-0.10861400	1.68034700
H	0.98318200	1.07753300	1.70833400
H	1.28491900	-2.37392500	-0.77749000
H	-0.89329500	-3.02978700	-0.80960500
H	-2.81181600	-2.14862300	-0.14120800
H	-3.51256700	-0.10503300	0.48667600
H	-0.19097100	0.63159500	-1.12722000
N	-0.08942900	2.62921700	-0.58918200
H	-0.36823800	3.32106500	0.09551000
H	0.88632400	2.70076300	-0.84761300
H	-2.39058300	1.96554000	0.53937900
C	2.54206400	-0.27211000	0.07686400
N	3.63225900	0.02626100	-0.18079600

1-NH_2-7-NO_2-substituted

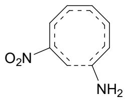

C	0.13484900	0.31655900	1.22933700
C	0.85840900	-0.54846600	0.44081800
C	0.37870200	-1.69581700	-0.20775700
C	-0.94797400	-2.09018800	-0.34218000
C	-2.22087000	-1.51602400	-0.09494500
C	-2.73903000	-0.23816300	0.13482500
C	-2.17145800	1.03823200	0.06914200
C	-0.90977200	1.33217600	-0.45328300
H	-0.73660000	-0.08913200	1.72515700
H	0.61879200	1.18116300	1.66937600
H	1.12971200	-2.34772400	-0.64302900
H	-1.03709200	-3.10723100	-0.72273400
H	-3.00602600	-2.26983200	-0.13494600
H	-3.79271500	-0.24040400	0.40836600
H	-0.46400800	0.61854700	-1.13752700
N	-0.44079100	2.61480200	-0.60241700
H	-0.78395900	3.30079900	0.05923100
H	0.55698400	2.69167100	-0.76620400
H	-2.75428100	1.86628300	0.47255800
N	2.20828700	-0.10598900	0.05063800
O	3.03307200	-0.94738600	-0.25019600
O	2.41687500	1.10292500	0.03475400

1-OH-7-CH_3-substituted

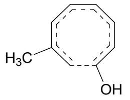

C	-0.68748600	-0.41058600	1.16076900
C	-1.52775900	0.29473100	0.32672700
C	-1.11427100	1.50692000	-0.27162500
C	0.14420400	2.09296500	-0.34207600
C	1.48344200	1.72358800	-0.04001200
C	2.18946300	0.54430700	0.18817600
C	1.82750000	-0.80642000	0.08524400
C	0.63030100	-1.27412200	-0.43395700
H	0.13234500	0.10490100	1.64505400
H	-1.03300600	-1.33092100	1.62709500
H	-1.90511500	2.08296900	-0.75239100
H	0.09806300	3.11867100	-0.70972300
H	2.13732600	2.59491700	-0.03111300
H	3.22421600	0.70414000	0.48650000
H	0.07917600	-0.69674900	-1.16449600
O	0.34395700	-2.59900100	-0.53634800
H	0.94541500	-3.10872200	0.02814700
H	2.52077300	-1.54217500	0.49556000
C	-2.84438300	-0.30022300	-0.11101200
H	-3.60385600	0.47821000	-0.23803200
H	-2.73615600	-0.80768900	-1.07917100
H	-3.21689200	-1.03251000	0.60995300

1-OH-7-NH_2-substituted

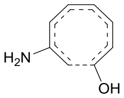

C	-0.67243500	-0.53319000	1.17654100
C	-1.57179200	0.03470800	0.30747600
C	-1.36092800	1.32361200	-0.26321500
C	-0.21408700	2.08910900	-0.32778100
C	1.17177700	1.93286900	-0.02199100
C	2.05370700	0.88047800	0.18011900
C	1.92625600	-0.51661300	0.06777200
C	0.83593700	-1.19021800	-0.44707200
H	0.03527100	0.10842600	1.68366700
H	-0.86275600	-1.51541500	1.60225700
H	-2.24142400	1.77862300	-0.71755100
H	-0.42002500	3.09927600	-0.68390000
H	1.67371300	2.89880800	0.01479600
H	3.05191800	1.20359900	0.47210800
H	0.15578900	-0.73815900	-1.15620300
O	0.78344300	-2.54333400	-0.51860600
H	1.48113900	-2.92557000	0.03601600
H	2.74200500	-1.11972900	0.46915300
N	-2.64151000	-0.71799000	-0.19091200
H	-3.08562200	-1.33943800	0.47316300
H	-3.31758600	-0.20234600	-0.73937400

1-OH-7-OH-substituted

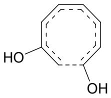

C	-0.61789800	-0.63938500	1.16048500
C	-1.56531700	-0.13571300	0.30865000
C	-1.49788300	1.14744800	-0.28645200
C	-0.43766600	2.03591900	-0.35207100
C	0.94775600	2.03450800	-0.02726100
C	1.93581800	1.08472600	0.21269200
C	1.96422300	-0.31433600	0.09899200
C	0.96122500	-1.09375500	-0.44961300
H	0.03275900	0.05552100	1.67296400
H	-0.75770500	-1.63024900	1.58149100
H	-2.42607600	1.50132600	-0.73671300
H	-0.75166800	3.01383700	-0.71792400
H	1.34308700	3.04887100	0.00020100
H	2.88286600	1.51669100	0.53259700
H	0.26648500	-0.69146900	-1.17410200
O	1.05497900	-2.44046100	-0.55625600
H	1.76362100	-2.76786500	0.01919100
H	2.82699300	-0.82989700	0.52255100
O	-2.59136700	-0.97209600	-0.05003700
H	-3.03080800	-0.63278800	-0.84244700

1-OH-7-F-substituted

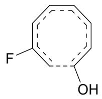

C	-0.54748200	-0.73887100	1.11933700
C	-1.53190900	-0.26841200	0.29180200
C	-1.60583700	0.98627900	-0.32495800
C	-0.64717600	1.99114200	-0.35934200
C	0.71914100	2.12114200	-0.00778300
C	1.79404600	1.26567000	0.24058700
C	1.96692200	-0.11703100	0.10889400
C	1.05333400	-1.00297300	-0.44773800
H	0.03982600	-0.01259100	1.66365200
H	-0.63367900	-1.74285900	1.52273000
H	-2.55333000	1.19861800	-0.81392100
H	-1.05419000	2.93308700	-0.72653800
H	1.01781900	3.16770100	0.03165800
H	2.68735500	1.78579100	0.58258500
H	0.33686700	-0.67661300	-1.18990400
O	1.30947900	-2.32843100	-0.56441500
H	2.04951700	-2.57554400	0.01174300
H	2.87972800	-0.54210800	0.52845700
F	-2.49466400	-1.14774500	-0.09110500

1-OH-7-CHO-substituted

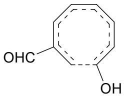

C	-0.28854900	-0.54545100	1.19946400
C	-1.22000200	0.14124100	0.44033000
C	-0.97645000	1.37992300	-0.18702600
C	0.20871300	2.08858100	-0.32225600
C	1.58615400	1.85726800	-0.05629000
C	2.41130200	0.75503200	0.14863200
C	2.18419700	-0.62505200	0.03675800
C	1.03281500	-1.21110100	-0.46537100
H	0.50345700	0.00245900	1.69222000
H	-0.56428000	-1.50413900	1.63759100
H	-1.85874100	1.84698300	-0.62039700
H	0.04736700	3.09614500	-0.70555900
H	2.14992000	2.78947200	-0.06339400
H	3.43117000	1.01439400	0.42593900
H	0.39566300	-0.67230300	-1.15450100
O	0.89009300	-2.55369400	-0.60336700
H	1.57379100	-3.01658300	-0.09455900
H	2.95962700	-1.28893200	0.42214900
C	-2.52410000	-0.52388100	0.18937700
H	-2.60983700	-1.54896700	0.60746900
O	-3.45416900	-0.02354300	-0.40271600

1-OH-7-CN-substituted

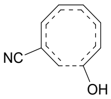

C	0.49236400	0.34945900	1.18233400
C	1.24349400	-0.47934000	0.37002800
C	0.73535000	-1.63037300	-0.27711800
C	-0.57507700	-2.07511800	-0.37547400
C	-1.86822200	-1.57422600	-0.06420900
C	-2.44573200	-0.33277800	0.18492500
C	-1.94844600	0.97608800	0.09106900
C	-0.72275800	1.33507500	-0.44581800
H	-0.38471300	-0.06528600	1.66057300
H	0.95405300	1.21752800	1.64314400
H	1.47902400	-2.26930000	-0.74734100
H	-0.63218000	-3.08855300	-0.77190800
H	-2.60878600	-2.37303200	-0.06869700
H	-3.48896900	-0.38760400	0.48986700
H	-0.21692100	0.69731900	-1.15910500
O	-0.31539900	2.61973300	-0.55682500
H	-0.87350700	3.19953800	-0.01521600
H	-2.56484100	1.77162000	0.51216500
C	2.59790200	-0.09254300	0.05282200
N	3.68668300	0.21177700	-0.20003200

1-OH-7-NO_2-substituted

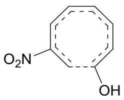

C	0.17056600	0.42132600	1.15107600
C	0.92131400	-0.44030300	0.38846900
C	0.47382400	-1.62792100	-0.21211700
C	-0.82516800	-2.11080400	-0.28407900
C	-2.12770500	-1.63070500	0.01892000
C	-2.73727500	-0.39480300	0.21640200
C	-2.27767000	0.92083200	0.05257200
C	-1.06902100	1.28516400	-0.51938900
H	-0.68849500	0.00422800	1.65824100
H	0.62290000	1.32235700	1.54924000
H	1.24359400	-2.25619700	-0.64714700
H	-0.85720600	-3.14228000	-0.63302600
H	-2.84613800	-2.44826700	0.06143200
H	-3.77485800	-0.46332100	0.53710300
H	-0.55754300	0.62186800	-1.20571500
O	-0.69588700	2.56922100	-0.70620800
H	-1.25010000	3.16612200	-0.17932000
H	-2.91131400	1.71953800	0.44061500
N	2.30122700	-0.01035800	0.05162200
O	3.02074100	-0.80405600	-0.53032100
O	2.64231800	1.11130400	0.38479300

1-F-7-CH_3-substituted

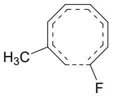

C	0.72284000	0.44195800	1.16995600
C	1.55327400	-0.24775900	0.32256300
C	1.14539900	-1.46918200	-0.27524900
C	-0.09852100	-2.07364500	-0.34880000

C	-1.44994000	-1.74089700	-0.02747500
C	-2.18860900	-0.58995500	0.20035700
C	-1.86181400	0.77839000	0.09587100
C	-0.69544400	1.25335600	-0.43806500
H	-0.10391300	-0.07103600	1.64506900
H	1.05007100	1.37367800	1.62632200
H	1.94367300	-2.03424200	-0.75726700
H	-0.03512000	-3.09210000	-0.73301000
H	-2.07305900	-2.63434400	-0.00378500
H	-3.21694900	-0.77780300	0.50290000
H	-0.10214900	0.72896800	-1.17548100
F	-0.49980100	2.58762000	-0.47860400
H	-2.54445600	1.51036500	0.52132100
C	2.86571600	0.34878500	-0.12622400
H	3.64515200	-0.41878400	-0.17994400
H	2.77383700	0.78522400	-1.12958900
H	3.20370800	1.13518700	0.55328300

1-F-7-NH_2-substituted

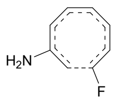

C	-0.72976800	-0.52617000	1.19295500
C	-1.58379300	0.07326000	0.30741600
C	-1.31312900	1.35763100	-0.26170200
C	-0.14293400	2.07791800	-0.32967000
C	1.24199800	1.88084200	-0.01219000
C	2.09377100	0.81155300	0.18407100
C	1.92134400	-0.59002000	0.07364300
C	0.82393900	-1.20522400	-0.45004500
H	0.01732300	0.07516500	1.69275400
H	-0.95256000	-1.50582200	1.60854000
H	-2.17422800	1.84699400	-0.71788100
H	-0.31064400	3.08990900	-0.69955200
H	1.76464300	2.83527000	0.03772100
H	3.10123700	1.10413500	0.47456600
H	0.13243100	-0.77797400	-1.16348200
F	0.78555200	-2.55216000	-0.45718900
H	2.68920800	-1.23624100	0.49231100
N	-2.67544900	-0.63105200	-0.21584800
H	-3.15164400	-1.24278200	0.43511800
H	-3.32615900	-0.08058400	-0.76133400

1-F-7-OH-substituted

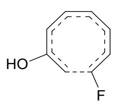

C	-0.71030000	-0.59166100	1.13522000
C	-1.58841400	0.02313300	0.28276900
C	-1.36627200	1.29328700	-0.30620800
C	-0.21960900	2.06039600	-0.34909300
C	1.15760200	1.91769900	0.00548600
C	2.04896900	0.88013100	0.22931300
C	1.93661500	-0.52054100	0.09922800
C	0.86145700	-1.16380800	-0.44921700

H	0.01683800	0.02038300	1.65109300
H	-0.92976500	-1.56923700	1.55930900
H	-2.24421300	1.71804200	-0.78777200
H	-0.41784300	3.06337500	-0.72751300
H	1.64348500	2.89081100	0.06492800
H	3.03206300	1.21274700	0.55710900
H	0.19873600	-0.74132900	-1.19272100
F	0.87194800	-2.51210400	-0.48696200
H	2.71753100	-1.14490000	0.52673800
O	-2.70984800	-0.60649700	-0.18889500
H	-2.90586400	-1.38079100	0.35764700

1-F-7-F-substituted

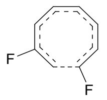

C	-0.64282000	-0.71376400	1.12943900
C	-1.56214900	-0.15534500	0.29172900
C	-1.51350200	1.10914400	-0.32242000
C	-0.47279500	2.02016900	-0.36233300
C	0.90357800	2.04129300	0.00400600
C	1.90559000	1.11168000	0.24743700
C	1.96563300	-0.28997100	0.11453300
C	0.99299800	-1.06953600	-0.45297800
H	0.02757800	-0.05362900	1.66153400
H	-0.80789100	-1.71194000	1.52230000
H	-2.43774700	1.40810300	-0.81118900
H	-0.79293000	2.98920800	-0.74430000
H	1.27588300	3.06334400	0.05940400
H	2.83718900	1.55645600	0.59201800
H	0.29079700	-0.73208900	-1.20374800
F	1.18953200	-2.39989400	-0.50042300
H	2.81428500	-0.81136000	0.55131500
F	-2.59690600	-0.93678600	-0.11333300

1-F-7-CHO-substituted

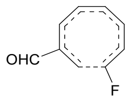

C	0.62304000	0.21685800	1.14018200
C	1.14651900	-0.70552800	0.27090400
C	0.41457200	-1.76384000	-0.33463700
C	-0.94081200	-2.01413800	-0.37929900
C	-2.13263200	-1.33599400	0.03226500
C	-2.50944800	-0.03359600	0.29395800
C	-1.83248800	1.20263300	0.14443000
C	-0.63899800	1.37747800	-0.48975200
H	-0.29863400	0.00942200	1.66652200
H	1.26684800	1.00602400	1.52173600
H	1.02714900	-2.50926300	-0.84316600
H	-1.17162700	-2.99465100	-0.79541700
H	-2.96967500	-2.02941700	0.10783900
H	-3.52624200	0.06756300	0.66747500
H	-0.21042800	0.69785700	-1.21363600
F	-0.12335000	2.61379000	-0.57063600

H	-2.27239400	2.08533500	0.60354000
C	2.56480500	-0.54877300	-0.15559200
H	2.96499800	-1.37538900	-0.77906400
O	3.26660100	0.39347600	0.13314500

1-F-7-CN-substituted

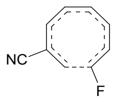

C	0.52933600	0.36385800	1.20223100
C	1.26954400	-0.44944400	0.37681000
C	0.76336300	-1.60972800	-0.27227300
C	-0.53532800	-2.06654200	-0.37914600
C	-1.84385300	-1.59251400	-0.05348100
C	-2.44986900	-0.37159200	0.18467700
C	-1.97814000	0.95718100	0.08786800
C	-0.77900900	1.32580100	-0.45305600
H	-0.36399800	-0.03831900	1.66072900
H	0.97602400	1.24465400	1.65347800
H	1.51485800	-2.24104500	-0.74069200
H	-0.57961800	-3.07365100	-0.79266800
H	-2.55985000	-2.41332500	-0.03850900
H	-3.49232500	-0.44679700	0.48659200
H	-0.21678800	0.74294400	-1.17095000
F	-0.46198700	2.63026400	-0.48655100
H	-2.58490100	1.75145700	0.51717300
C	2.61764500	-0.05395700	0.04188600
N	3.70033600	0.25476200	-0.22990000

1-F-7-NO_2-substituted

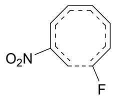

C	0.20918600	0.42773500	1.17473200
C	0.94257900	-0.42267800	0.39736700
C	0.49428000	-1.61969900	-0.20323400
C	-0.79383700	-2.10867000	-0.28687500
C	-2.10980900	-1.64652800	0.02636400
C	-2.74088000	-0.42898000	0.20858100
C	-2.30173800	0.90527700	0.04529900
C	-1.11882500	1.28317500	-0.52351500
H	-0.67060700	0.02826200	1.66023900
H	0.64974900	1.34046600	1.55987300
H	1.27061200	-2.24352500	-0.63350900
H	-0.81791800	-3.13438400	-0.65265200
H	-2.80812900	-2.48028500	0.08753200
H	-3.77958100	-0.51247000	0.52090000
H	-0.54350300	0.68326900	-1.21694000
F	-0.83760300	2.59105600	-0.61679800
H	-2.92667700	1.70266700	0.44174500
N	2.31624600	0.01315500	0.03860400
O	3.04144100	-0.79242300	-0.51822600
O	2.64168700	1.15075200	0.32840700

1-CHO-7-CH_3-substituted

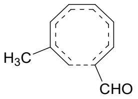

C	-0.16116700	-0.84688600	1.11795000
C	-1.20377900	-1.24400400	0.31563300
C	-2.12808400	-0.30904300	-0.21320700
C	-2.11330400	1.07772000	-0.28763600
C	-1.20968200	2.14948200	-0.03040600
C	0.15657900	2.32770400	0.16407300
C	1.22691200	1.42445800	0.04908300
C	1.12612900	0.16663900	-0.50645200
H	-0.19077500	0.12138800	1.60141500
H	0.51901700	-1.57970200	1.54653400
H	-3.02404200	-0.75576900	-0.64602100
H	-3.07651700	1.46808200	-0.61636100
H	-1.74573800	3.09830100	-0.00526700
H	0.43269000	3.33966800	0.45502700
H	0.31812700	-0.04965800	-1.19597500
C	2.29425800	-0.73974300	-0.54026000
H	2.18166600	-1.64194500	-1.17469400
O	3.31565900	-0.56227200	0.08963000
H	2.18722200	1.70890500	0.47712100
C	-1.32702400	-2.68112000	-0.13327700
H	-2.36599500	-3.02209600	-0.07024000
H	-1.01408000	-2.79389200	-1.17942000
H	-0.71187200	-3.34634800	0.47785200

1-CHO-7-NH_2-substituted

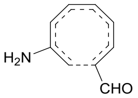

C	-0.14849200	-0.90821700	1.11510900
C	-1.18531000	-1.28515600	0.29671100
C	-2.13461600	-0.35220900	-0.21085800
C	-2.12267700	1.02757000	-0.29088600
C	-1.23750000	2.11883300	-0.02210300
C	0.12077600	2.32202700	0.17587800
C	1.21281700	1.43981800	0.05856500
C	1.13320300	0.19230600	-0.51359100
H	-0.20097800	0.04267100	1.62804100
H	0.54059100	-1.64631800	1.51988300
H	-3.03817200	-0.81620000	-0.60546800
H	-3.09101100	1.40642300	-0.61797600
H	-1.79599800	3.05375500	0.01638900
H	0.37566700	3.33778100	0.47384600
H	0.32581700	-0.03318700	-1.20129900
C	2.30601700	-0.70673500	-0.54798600
H	2.19672300	-1.60930800	-1.18354500
O	3.32882800	-0.52609200	0.07860700
H	2.16356000	1.73518200	0.49946000
N	-1.36194200	-2.61821500	-0.09621100
H	-0.64709700	-3.26014800	0.22208500
H	-1.59143500	-2.76383400	-1.07182100

1-CHO-7-OH-substituted

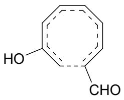

C	-0.14410400	-0.94385300	1.12409600
C	-1.17952300	-1.28397000	0.29967000
C	-2.13162100	-0.36873600	-0.22166800
C	-2.13477300	1.01185700	-0.29302600
C	-1.25121100	2.10034500	-0.01803600
C	0.10745900	2.29770000	0.18141900
C	1.20247800	1.41810800	0.06066300
C	1.14190700	0.17504200	-0.52019500
H	-0.16944300	0.00716700	1.63670100
H	0.51760000	-1.71600300	1.50501400
H	-3.02947000	-0.83740700	-0.62748500
H	-3.10411900	1.38633300	-0.62139600
H	-1.80753400	3.03628700	0.02300300
H	0.36354600	3.31159200	0.48465400
H	0.33803700	-0.06443600	-1.20649300
C	2.32971700	-0.70979000	-0.54344600
H	2.23224300	-1.62824300	-1.15450900
O	3.35083200	-0.49064500	0.07278300
H	2.14928700	1.71720100	0.50791700
O	-1.26096200	-2.59758800	-0.08722200
H	-1.85107300	-2.68683700	-0.84877500

1-CHO-7-F-substituted

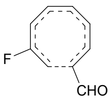

C	-0.08953500	-0.97279300	1.07216600
C	-1.16376200	-1.28022600	0.28709300
C	-2.12463400	-0.41295900	-0.25757600
C	-2.16531300	0.97228500	-0.30494700
C	-1.30449300	2.06303800	-0.00207000
C	0.05450500	2.27370500	0.21402200
C	1.16226000	1.42084400	0.07596000
C	1.13361900	0.17691900	-0.51885000
H	-0.09641600	-0.04178500	1.61990800
H	0.55837200	-1.76798900	1.42832900
H	-2.97590900	-0.92517300	-0.70044700
H	-3.13806900	1.33030800	-0.64017000
H	-1.87008600	2.99311800	0.04584500
H	0.29258300	3.28364100	0.54358600
H	0.34905600	-0.06415600	-1.22696200
C	2.36184300	-0.65272100	-0.56770200
H	2.32203000	-1.53581100	-1.23415300
O	3.35604200	-0.42350800	0.08725200
H	2.10504000	1.73714800	0.52016100
F	-1.28687600	-2.57219900	-0.11585600

1-CHO-4-CHO-substituted

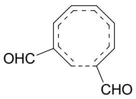

C	-0.27890600	-0.54313000	1.18037400
C	-1.39915000	-0.24054300	0.44079400
C	-1.67821400	1.02345400	-0.13557300
C	-0.90064100	2.15797000	-0.27956100
C	0.45396300	2.54630200	-0.04526100
C	1.68448700	1.92774000	0.12573300
C	2.06250400	0.57603500	0.00368500
C	1.27439800	-0.41842300	-0.53040200
H	0.29554200	0.24073500	1.65470800
H	-0.14924000	-1.54602700	1.58542100
H	-2.68922400	1.10642900	-0.53111200
H	-1.48410200	3.00631600	-0.63647000
H	0.54156400	3.63270100	-0.02933300
H	2.48996800	2.60866200	0.39390400
H	0.44921100	-0.15677400	-1.18306400
C	1.76650100	-1.81497100	-0.58263800
H	1.18510200	-2.50746200	-1.22237500
O	2.72443600	-2.21710400	0.04245000
H	3.03089000	0.28056100	0.40617600
C	-2.35338300	-1.34719300	0.16634600
H	-2.04298800	-2.33645800	0.56342900
O	-3.40144600	-1.22441200	-0.42523500

1-CHO-7-CN-substituted

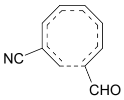

C	-0.49020500	-0.41908200	1.17473100
C	-1.52614300	0.01915300	0.38637700
C	-1.60728300	1.31110700	-0.20678800
C	-0.66433900	2.31345000	-0.32279800
C	0.72879400	2.49864700	-0.05667900
C	1.84980200	1.70839800	0.13799400
C	2.02959100	0.31209200	0.03157300
C	1.12434600	-0.56010000	-0.52273100
H	0.16717700	0.30715200	1.63330300
H	-0.50003200	-1.41956900	1.59725000
H	-2.57968600	1.55325700	-0.63030300
H	-1.10739500	3.23736100	-0.69280700
H	0.97237200	3.56065100	-0.03700800
H	2.74109200	2.26622900	0.41834300
H	0.35506600	-0.19112400	-1.19163000
C	1.40639000	-2.01725800	-0.54886700
H	0.73711400	-2.62845400	-1.18349600
O	2.29182700	-2.53956400	0.09297000
H	2.93408000	-0.11673500	0.46149900
C	-2.56556800	-0.92059200	0.03580300
N	-3.39524400	-1.67530700	-0.25294400

1-CHO-7-NO_2-substituted

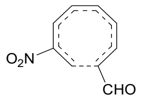

C	-0.23992600	-0.30140100	1.18179000
C	-1.19544600	0.29701200	0.41774000
C	-1.16070100	1.59456100	-0.14790800
C	-0.10418500	2.47153400	-0.26820000
C	1.30738000	2.47064100	-0.02269700
C	2.31863700	1.54047000	0.13593300
C	2.32194000	0.13105100	0.00813100
C	1.32491000	-0.62339500	-0.55548800
H	0.50080900	0.32612500	1.65829200
H	-0.37672800	-1.31117900	1.55299800
H	-2.11159200	1.94727500	-0.53480600
H	-0.43233900	3.45045800	-0.61480100
H	1.68503000	3.49208200	0.01684800
H	3.27833000	1.97411300	0.41072200
H	0.59478900	-0.16353300	-1.21233700
C	1.43073600	-2.10589500	-0.58538100
H	0.68085500	-2.63345300	-1.20300500
O	2.26285000	-2.72534300	0.04065500
H	3.16755300	-0.40921600	0.43331800
N	-2.35203400	-0.54483600	0.02579000
O	-3.34979100	0.01467600	-0.39116100
O	-2.23087600	-1.75262000	0.14159800

1-CN-7-CH_3-substituted

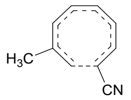

C	-0.15991600	0.74166400	1.19615900
C	0.51970100	1.55784400	0.32285300
C	1.70738100	1.11996200	-0.31223000
C	2.28941700	-0.13688500	-0.41611600
C	1.95794800	-1.48725300	-0.10352400
C	0.82021200	-2.23221900	0.19021900
C	-0.54228600	-1.88842600	0.16754300
C	-1.02390300	-0.70308500	-0.35441800
H	0.34778700	-0.10192900	1.64732300
H	-1.05821400	1.09577000	1.69664700
H	2.27316000	1.90659000	-0.81220500
H	3.29515200	-0.07985800	-0.83233600
H	2.84867300	-2.11429000	-0.13586900
H	1.02680100	-3.26051000	0.47987400
H	-0.43941700	-0.17251200	-1.09612200
C	-2.43138700	-0.41672800	-0.35626100
N	-3.56014700	-0.15400600	-0.34290300
H	-1.24449000	-2.56954000	0.64295800
C	-0.05645200	2.88973100	-0.09362900
H	0.72978000	3.64746400	-0.17563700
H	-0.53801200	2.81455400	-1.07710400
H	-0.80449100	3.24465900	0.61922500

1-CN-7-NH_2-substituted

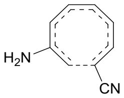

C	-0.17138700	0.79000300	1.20322700
C	0.52510600	1.56585100	0.30893200
C	1.73106800	1.11215900	-0.30130600
C	2.27604100	-0.15167100	-0.41180800
C	1.92784900	-1.50481600	-0.09650000
C	0.78620800	-2.23788100	0.18886900
C	-0.57839800	-1.88253500	0.16605800
C	-1.03947500	-0.69941600	-0.36599500
H	0.33340900	-0.03818300	1.68298400
H	-1.07646800	1.16222900	1.67714100
H	2.32304300	1.90375300	-0.75972800
H	3.28460600	-0.11099200	-0.82311700
H	2.81639800	-2.13490900	-0.11467300
H	0.98370300	-3.26870900	0.47684900
H	-0.44271700	-0.17431700	-1.10186500
C	-2.43766700	-0.37177100	-0.36147400
N	-3.55664300	-0.06954100	-0.34617900
H	-1.28618300	-2.54984500	0.65184300
N	0.07380100	2.83200500	-0.07028500
H	-0.83035400	3.09756800	0.29983600
H	0.15838400	3.05661800	-1.05404700

1-CN-7-OH-substituted

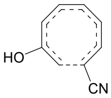

C	-0.07865400	0.85142300	1.19582200
C	0.74132600	1.48940200	0.30524200
C	1.85719100	0.88298200	-0.32763000
C	2.23535900	-0.44299000	-0.42973600
C	1.71207000	-1.72766100	-0.09109200
C	0.48130600	-2.28925100	0.21612000
C	-0.82355200	-1.75787300	0.18591300
C	-1.14448900	-0.53726600	-0.36580500
H	0.27541400	-0.05038700	1.67548500
H	-0.90186200	1.39295900	1.65149100
H	2.54526400	1.58414200	-0.80179600
H	3.23555000	-0.53936900	-0.85130600
H	2.50449700	-2.47523400	-0.10778900
H	0.53787100	-3.33099100	0.52602700
H	-0.49356500	-0.09081200	-1.10691500
C	-2.50318500	-0.07140000	-0.36742800
N	-3.59543600	0.31504800	-0.35820900
H	-1.60876600	-2.32352700	0.68181000
O	0.41808400	2.77597400	-0.02998800
H	0.86474600	3.03589600	-0.84807200

1-CN-7-F-substituted

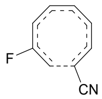

C	-0.11418100	0.86939200	1.15147900
C	0.76792200	1.46219400	0.29148000
C	1.86462800	0.87465800	-0.35760800
C	2.24724000	-0.45627500	-0.43474900
C	1.71159300	-1.72318600	-0.07681000
C	0.46946200	-2.26527300	0.24191400
C	-0.82754000	-1.72897100	0.19698400
C	-1.14892300	-0.50474300	-0.36108200
H	0.20119400	-0.02444600	1.67086600
H	-0.92036700	1.45673500	1.58005300
H	2.51431800	1.58630200	-0.86203000
H	3.24596400	-0.56285400	-0.85634300
H	2.49112800	-2.48437300	-0.08722300
H	0.51649900	-3.30080500	0.57309900
H	-0.50939300	-0.07597600	-1.12284700
C	-2.51480600	-0.05765100	-0.37278700
N	-3.61148900	0.31554200	-0.36312400
H	-1.61781100	-2.29004700	0.69036900
F	0.51405700	2.74064500	-0.07966600

1-CN-7-CHO-substituted

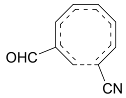

C	0.29301400	0.40338600	1.25133000
C	1.33320800	-0.00765800	0.44687600
C	1.38799500	-1.25129200	-0.22407300
C	0.42825500	-2.23407600	-0.39855100
C	-0.96041200	-2.40583700	-0.12149700
C	-2.06449800	-1.60515300	0.14245100
C	-2.22682900	-0.20762800	0.11004600
C	-1.28228900	0.67253100	-0.38091300
H	-0.36497200	-0.32720200	1.70326700
H	0.32822900	1.38468900	1.72260800
H	2.35579200	-1.46347100	-0.67546500
H	0.84772900	-3.14094700	-0.83291100
H	-1.23107100	-3.46060000	-0.16630700
H	-2.96202900	-2.15995500	0.40671200
H	-0.53830200	0.31839900	-1.08414700
C	-1.53761400	2.08580700	-0.39730000
N	-1.71621200	3.23053800	-0.38750100
H	-3.13259400	0.20203900	0.55194800
C	2.44087300	0.95741200	0.20535000
H	2.29570900	1.95780700	0.66201900
O	3.44309800	0.70381500	-0.42219200

1-CN-7-CN-substituted

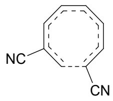

C	0.54415400	0.12854300	1.21652400
C	1.29061200	-0.65617100	0.36669100
C	0.79444900	-1.80083800	-0.31240600
C	-0.49645600	-2.28057700	-0.44367100
C	-1.82010400	-1.85867300	-0.11689800
C	-2.47679300	-0.67417700	0.18325000
C	-2.04002900	0.66508700	0.15897500
C	-0.83765300	1.08566600	-0.37002300
H	-0.34784300	-0.29155500	1.66175500
H	0.98825800	0.99367300	1.69961700
H	1.55540200	-2.40675200	-0.79917200
H	-0.51041300	-3.27456000	-0.88919400
H	-2.50722400	-2.70361700	-0.14970900
H	-3.51577700	-0.80560100	0.47757000
H	-0.32175400	0.47199100	-1.09874100
C	-0.49216700	2.48021100	-0.36433200
N	-0.20140100	3.60120500	-0.34045100
H	-2.67748300	1.40602000	0.63668800
C	2.64171600	-0.25313200	0.05459200
N	3.72861100	0.05518800	-0.19883900

1-CN-7-NO_2-substituted

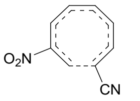

C	0.22932200	0.21702600	1.20547700
C	1.03232900	-0.52744200	0.38695300
C	0.70044200	-1.73255400	-0.26467900
C	-0.52752700	-2.35769900	-0.38048200
C	-1.88814000	-2.07412400	-0.05606600
C	-2.66988900	-0.96015300	0.21112600
C	-2.38795600	0.41927100	0.14226200
C	-1.24750700	0.96459100	-0.40896300
H	-0.60712300	-0.27897200	1.67861300
H	0.59492100	1.14457700	1.63258200
H	1.53626300	-2.24736700	-0.72753600
H	-0.43119000	-3.35826500	-0.79929400
H	-2.47869200	-2.98969400	-0.05705200
H	-3.68711500	-1.19635200	0.51563400
H	-0.66372500	0.39439500	-1.12179800
C	-1.07521100	2.39015900	-0.44819300
N	-0.93276700	3.53936900	-0.46252000
H	-3.10570100	1.09584900	0.60140000
N	2.36096200	0.04917500	0.05123200
O	3.16104000	-0.66727100	-0.52363000
O	2.57018500	1.20246800	0.37761300

1-NO_2-7-CH_3-substituted

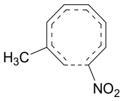

C	0.23720800	0.71945700	1.27043700
C	1.00705500	1.46465300	0.41527700
C	2.12824700	0.89549800	-0.24681200
C	2.53400400	-0.41782300	-0.42054700

C	2.02836000	-1.73070700	-0.17069500
C	0.80571700	-2.33405800	0.08862100
C	-0.49988100	-1.80567600	0.10432900
C	-0.80252200	-0.55265000	-0.35286300
H	0.62108600	-0.20778700	1.67862300
H	-0.61136900	1.16629700	1.78463700
H	2.79618300	1.62215200	-0.71056800
H	3.53470200	-0.47604000	-0.84834600
H	2.83108200	-2.46474300	-0.23835200
H	0.87151200	-3.39504300	0.31897800
H	-0.23527300	-0.02220200	-1.10202200
N	-2.17353600	-0.06859500	-0.27239900
O	-2.44172400	0.91106500	-0.95229500
O	-2.95345800	-0.63080600	0.47966600
H	-1.29656800	-2.38485900	0.56410300
C	0.61497300	2.87480900	0.04696100
H	0.18266200	2.90687300	-0.96052500
H	-0.12567800	3.27731000	0.74174200
H	1.48889000	3.53511100	0.05130300

1-NO_2-7-NH_2-substituted

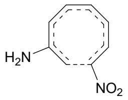

C	0.20045700	0.76124600	1.28846100
C	0.94969100	1.49464200	0.40661200
C	2.11337000	0.95346300	-0.22567700
C	2.53366700	-0.34594800	-0.40513000
C	2.05808500	-1.67943600	-0.16092500
C	0.85405500	-2.31684700	0.07796800
C	-0.47109600	-1.82392500	0.09030600
C	-0.79129400	-0.58206500	-0.37024200
H	0.62180300	-0.13491800	1.72546500
H	-0.67124800	1.19185400	1.77672300
H	2.77927700	1.70722700	-0.64463100
H	3.54023600	-0.38188200	-0.82152800
H	2.88564400	-2.38633500	-0.21045900
H	0.94825600	-3.37746000	0.30111400
H	-0.22262300	-0.03312300	-1.10441200
N	-2.15843400	-0.09243300	-0.27682700
O	-2.40651100	0.93407600	-0.89565200
O	-2.95568300	-0.69039600	0.42632000
H	-1.25301700	-2.41867200	0.55461100
N	0.61093700	2.80289500	0.05895900
H	-0.28185200	3.11794800	0.41990600
H	0.70195000	3.02590400	-0.92530500

1-NO_2-7-OH-substituted

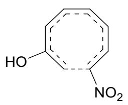

C	0.26097800	0.79425700	1.28623800
C	1.08109200	1.43841600	0.40490600
C	2.16437500	0.81922800	-0.28011600
C	2.49373900	-0.50883200	-0.45874900

C	1.93762100	-1.79660000	-0.17095700
C	0.69608000	-2.33448200	0.11885600
C	-0.58978000	-1.74948700	0.13825000
C	-0.84932200	-0.50437000	-0.35204200
H	0.58057000	-0.14664900	1.71248500
H	-0.54005300	1.34015000	1.77624100
H	2.86878800	1.52012100	-0.72954300
H	3.47988100	-0.61622200	-0.90949800
H	2.70905600	-2.56353600	-0.23092000
H	0.71708000	-3.39249000	0.37102100
H	-0.26433700	-0.00460400	-1.10862300
N	-2.20470200	0.02776400	-0.28074600
O	-2.46165000	0.95181500	-1.03616300
O	-2.98142000	-0.45436300	0.52773600
H	-1.40126700	-2.28280400	0.62620900
O	0.80960400	2.75132100	0.14010100
H	1.18223100	3.01271400	-0.71386700

1-NO_2-7-F-substituted

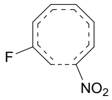

C	0.21964600	0.81872200	1.23582100
C	1.10798600	1.41339400	0.38926100
C	2.17924000	0.81531400	-0.30150000
C	2.51589500	-0.51770900	-0.45631700
C	1.94643800	-1.78938000	-0.15664700
C	0.69293100	-2.30933900	0.13655500
C	-0.58672800	-1.72225400	0.13928700
C	-0.85520200	-0.47236900	-0.34964700
H	0.49665900	-0.11565300	1.70332400
H	-0.56671600	1.41088000	1.69500400
H	2.84890000	1.53037200	-0.77419000
H	3.50330400	-0.63301400	-0.90140000
H	2.70524900	-2.56939400	-0.20887700
H	0.70474100	-3.36323900	0.40579300
H	-0.28998200	0.01994200	-1.12667600
N	-2.22390000	0.03253500	-0.27879100
O	-2.50373400	0.94247500	-1.04146900
O	-2.98329700	-0.45481000	0.54319100
H	-1.40132900	-2.25708600	0.62088100
F	0.90461100	2.71442800	0.07811500

1-NO_2-7-CHO-substituted

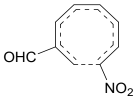

C	-0.34476600	-0.30440100	1.31381900
C	-1.45434700	-0.37352200	0.50955500
C	-2.01722800	0.72655300	-0.18801800
C	-1.54032000	2.00259600	-0.41460800
C	-0.33460100	2.73623400	-0.17906200
C	1.00285800	2.46382900	0.05862500
C	1.71452000	1.24512200	0.06390900
C	1.19212300	0.06066700	-0.37277300

H	-0.02372500	0.64222500	1.72944200
H	0.03396800	-1.20416200	1.79850500
H	-2.99435500	0.51566000	-0.61974500
H	-2.29459100	2.64621700	-0.86619400
H	-0.52131100	3.80780300	-0.24763600
H	1.60608200	3.34176700	0.27729700
H	0.39134000	-0.03107500	-1.09041200
N	1.99397400	-1.15408600	-0.29613600
O	1.57252900	-2.11428400	-0.92194900
O	2.99307200	-1.15639000	0.40435200
H	2.71025500	1.22602400	0.50089000
C	-2.06794300	-1.71446400	0.29168900
H	-1.51351500	-2.56070000	0.74503300
O	-3.09731800	-1.89943100	-0.31403400

1-NO_2-7-CN-substituted

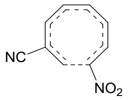

C	0.45610500	0.38361400	1.27786900
C	1.51105500	0.60061600	0.42758400
C	2.19620800	-0.42466700	-0.28728700
C	1.89054000	-1.75805600	-0.46954600
C	0.81281600	-2.65237800	-0.17335500
C	-0.53795400	-2.56766200	0.11295700
C	-1.41734600	-1.46191200	0.12826200
C	-1.09784900	-0.22736900	-0.35714000
H	0.30413900	-0.60706200	1.68580900
H	-0.02995900	1.21419300	1.78215400
H	3.12165000	-0.10407900	-0.76061300
H	2.71236200	-2.29572000	-0.94062500
H	1.15073400	-3.68657500	-0.23186200
H	-1.00220700	-3.51816600	0.36520500
H	-0.34623500	-0.03153200	-1.10758100
N	-2.08574600	0.84553600	-0.27871300
O	-1.90375400	1.79705400	-1.01818000
O	-2.99728200	0.73830100	0.52589300
H	-2.38708100	-1.57714000	0.60655200
C	1.92017900	1.95943800	0.15684700
N	2.26894100	3.04210600	-0.05812900

1-NO_2-7-NO_2-substituted

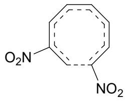

C	-0.31808200	-0.05430500	1.26642900
C	-1.32127000	0.33715300	0.43026900
C	-1.41873200	1.56224300	-0.26779700
C	-0.48369000	2.56672500	-0.41670400
C	0.89292100	2.78726900	-0.09476600
C	2.01455600	2.02410300	0.17884500
C	2.21838700	0.62630700	0.14381400
C	1.32295400	-0.26111100	-0.37818100
H	0.31440900	0.70864800	1.69960400
H	-0.33929800	-1.03617800	1.72853900

H	-2.37995400	1.74258400	-0.73823300
H	-0.91889400	3.45775800	-0.86674000
H	1.12255500	3.85216300	-0.11491600
H	2.89110100	2.60204700	0.46135900
H	0.57823900	-0.02238000	-1.12332300
N	1.63679900	-1.68759700	-0.36141000
O	1.00156300	-2.38274500	-1.13454300
O	2.47878300	-2.08866900	0.42516800
H	3.11302300	0.21999900	0.61008600
N	-2.37771100	-0.67013300	0.13429800
O	-2.25602600	-1.77264100	0.63173400
O	-3.30395300	-0.32480000	-0.57711500

5.3.8 1,8-substituted

1-CH_3-8-CH_3-substituted

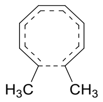

C	0.72663600	0.94155000	-0.63162000
C	0.07153300	1.73965800	0.28731600
C	-1.28420700	1.61392900	0.63708800
C	-2.22412000	0.63689200	0.31511700
C	-2.22467000	-0.63582400	-0.31456800
C	-1.28527600	-1.61289800	-0.63789400
C	0.07027000	-1.73976000	-0.28782600
C	0.72548300	-0.94198200	0.63129900
H	0.10820300	0.42027400	-1.35399900
H	0.65368900	2.48729000	0.82835100
H	-1.67830800	2.42250800	1.25102400
H	-3.23563700	0.93699700	0.58984700
H	-3.23656700	-0.93554800	-0.58831200
H	-1.67988600	-2.42077400	-1.25243000
H	0.10703400	-0.42000700	1.35314700
C	2.14585100	-1.20249700	1.04462000
H	2.71112700	-1.69611400	0.24725000
H	2.66457300	-0.27666400	1.31464000
H	2.16945000	-1.85484800	1.92656200
H	0.65210200	-2.48766600	-0.82882500
C	2.14747600	1.20119200	-1.04385100
H	2.71242800	1.69430900	-0.24591600
H	2.66578400	0.27512100	-1.31368800
H	2.17214100	1.85357200	-1.92574600

1-CH_3-8-NH_2-substituted

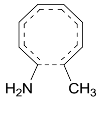

C	-0.71893300	0.88446200	0.64282300
C	-0.06104100	1.73674200	-0.23850400
C	1.28509100	1.62425900	-0.59359900
C	2.22406900	0.63306200	-0.26992700
C	2.20190100	-0.64827300	0.31431300
C	1.23172300	-1.62465000	0.59841900
C	-0.10178300	-1.73747300	0.22288500

C	-0.75715200	-0.87813000	-0.66949400
H	-0.09822700	0.38067200	1.37591200
H	-0.64844200	2.50061000	-0.75202200
H	1.68399300	2.44012100	-1.19399300
H	3.24223900	0.94063100	-0.51079400
H	3.20412900	-0.98339800	0.58034600
H	1.60439900	-2.45173800	1.20052200
H	-0.15318100	-0.35267600	-1.39924000
N	-2.04706800	-1.16114900	-1.10236000
H	-2.64996900	-1.60360400	-0.41819100
H	-2.52017500	-0.40511100	-1.58158400
H	-0.69147500	-2.52927700	0.68592200
C	-2.14853800	1.11780900	1.03940700
H	-2.65574500	0.17807800	1.28873100
H	-2.20848000	1.75986700	1.92675400
H	-2.71160300	1.60701500	0.23622200

1-CH_3-8-OH-substituted

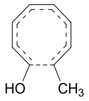

C	-0.76199900	-0.87559000	-0.69385100
C	-0.12244500	-1.73748900	0.18888800
C	1.21439900	-1.63355200	0.58547300
C	2.18554500	-0.65983600	0.33672300
C	2.22546400	0.63080300	-0.24242800
C	1.30232000	1.62696100	-0.56788500
C	-0.05692300	1.74247900	-0.24406700
C	-0.72738400	0.90739000	0.63425100
H	-0.19852700	-0.33754700	-1.44321400
H	-0.72250400	-2.52335800	0.65117700
H	1.57333700	-2.47559500	1.17450900
H	3.18080100	-0.99596800	0.62637300
H	3.24934200	0.92826300	-0.47057700
H	1.71582900	2.44647300	-1.15339900
H	-0.11892700	0.38946600	1.36878200
C	-2.17185000	1.11637700	0.98690900
H	-2.73829500	1.48813300	0.12767700
H	-2.63906400	0.18390700	1.32267700
H	-2.26973600	1.83968300	1.80597600
H	-0.63494200	2.49400800	-0.78416200
O	-2.05340600	-1.05733000	-1.08055500
H	-2.49281900	-1.68408100	-0.48545600

1-CH_3-8-F-substituted

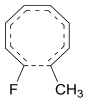

C	-0.69687600	-0.93769300	-0.69403000
C	-0.04838800	-1.76048200	0.19007800
C	1.28979500	-1.59246800	0.59244400
C	2.20396500	-0.57339300	0.35244900
C	2.18575800	0.71989400	-0.23997300
C	1.22869400	1.67643300	-0.55937700
C	-0.14328200	1.74740500	-0.24775200

C	-0.79519400	0.90946100	0.63172500
H	-0.20408300	-0.36298800	-1.46545300
H	-0.63629300	-2.55028700	0.65300700
H	1.68728200	-2.41784900	1.17964400
H	3.21268600	-0.85051200	0.65796600
H	3.19591800	1.05417000	-0.47707100
H	1.61379100	2.51457700	-1.13817600
H	-0.18321900	0.38930700	1.36211000
C	-2.25492800	1.04412500	0.95233600
H	-2.79313000	1.54674700	0.14329600
H	-2.71726900	0.06477400	1.11174400
H	-2.39169000	1.62590600	1.87226700
H	-0.73785700	2.47589600	-0.80075000
F	-1.98482200	-1.20993600	-0.99622100

1-CH_3-8-CHO-substituted

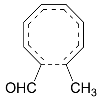

C	-0.65184400	-0.67861300	-0.63905800
C	-0.23725500	-1.63216000	0.27664500
C	1.10135500	-1.84727300	0.61994900
C	2.24479300	-1.10964700	0.29528200
C	2.55087000	0.13538100	-0.30643800
C	1.88647500	1.32528400	-0.61852500
C	0.60887300	1.79714100	-0.28367300
C	-0.24073600	1.18838800	0.62404200
H	0.05591000	-0.32205700	-1.37773400
H	-1.00572100	-2.20965000	0.78863500
H	1.29917100	-2.73382600	1.21952000
H	3.15745300	-1.64853900	0.55077000
H	3.60711900	0.18646900	-0.57003000
H	2.48416100	2.01176800	-1.21643400
H	0.21206200	0.49650100	1.32665600
C	-1.54956300	1.78591100	1.04619700
H	-1.98003300	2.41200400	0.25812200
H	-2.27530700	1.01082900	1.31238100
H	-1.39721500	2.41170500	1.93478000
H	0.25328600	2.68422500	-0.80868000
C	-2.08101900	-0.50629800	-0.95281900
H	-2.30710600	0.03901200	-1.89056100
O	-2.98693300	-0.88589200	-0.23713000

1-CH_3-8-CN-substituted

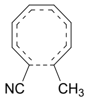

C	-0.67102800	-0.81302200	-0.49145900
C	-0.07968200	-1.70001500	0.39588300
C	1.29816200	-1.74909500	0.63803400
C	2.32706700	-0.90810800	0.20859600
C	2.45045500	0.33966700	-0.45104400
C	1.63808900	1.43951500	-0.73595600
C	0.34719700	1.77994500	-0.30512100
C	-0.35468900	1.11617300	0.68555300

H	-0.07796700	-0.39910300	-1.29708100
H	-0.72221500	-2.35432700	0.98114200
H	1.63087800	-2.59079900	1.24173400
H	3.30840000	-1.33942000	0.40673600
H	3.46982000	0.48973000	-0.80585700
H	2.10270600	2.16394700	-1.40305100
H	0.22572000	0.51161000	1.37587700
C	-1.69931300	1.57573100	1.16543200
H	-2.29114900	0.74519300	1.56150900
H	-1.57312800	2.30571600	1.97498300
H	-2.27088800	2.04875800	0.36152500
H	-0.15114800	2.59548100	-0.82949900
C	-2.08759800	-0.83624600	-0.70858000
N	-3.23757100	-0.80629400	-0.85372000

1-CH_3-8-NO_2-substituted

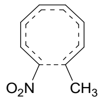

C	-0.48960000	-0.66796400	-0.44032200
C	0.02626000	-1.65602300	0.36134800
C	1.40248200	-1.86081500	0.54019000
C	2.49372700	-1.09625900	0.13289800
C	2.72028300	0.17477300	-0.45817400
C	2.01602800	1.36156700	-0.64925100
C	0.77597500	1.81181800	-0.15997700
C	0.04144900	1.19233400	0.82915200
H	0.02714900	-0.23649600	-1.28256400
H	-0.67308100	-2.26343900	0.93022900
H	1.66639300	-2.77147000	1.07289800
H	3.43450800	-1.62779300	0.27635100
H	3.73706900	0.24336300	-0.84365300
H	2.53301300	2.07290100	-1.29167500
H	0.56200000	0.48789600	1.47186000
C	-1.25219200	1.74588300	1.34466700
H	-1.76554900	2.33478600	0.57884600
H	-1.92261300	0.94861300	1.68229700
H	-1.06029200	2.39370100	2.20940900
H	0.34724200	2.69060200	-0.64134800
N	-1.92719300	-0.51070000	-0.54773300
O	-2.33025800	0.21469900	-1.44563400
O	-2.64498700	-1.05590500	0.27917000

1- NH_2-8-NH_2-substituted

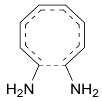

C	0.77926000	-0.78351500	0.65656100
C	0.14401400	-1.71895100	-0.18019800
C	-1.19124600	-1.66672200	-0.54553500
C	-2.18264400	-0.70371400	-0.25469200
C	-2.21613200	0.59067700	0.27911700
C	-1.27779800	1.61238000	0.55427000
C	0.04318100	1.74919300	0.16864400
C	0.71459200	0.85220900	-0.68727000

H	0.16744500	-0.29653900	1.40621600
H	0.76139800	-2.49861600	-0.63109500
H	-1.55039900	-2.51905800	-1.11897000
H	-3.18407200	-1.07328200	-0.47587700
H	-3.23379500	0.90042700	0.51699800
H	-1.68041000	2.43726500	1.13947400
H	0.11069300	0.34540900	-1.43178400
N	2.02265300	1.12079400	-1.09439300
H	2.63427200	1.33386400	-0.31076300
H	2.43380900	0.35135500	-1.61246200
H	0.62365100	2.56445800	0.60170200
N	2.14026700	-0.92093300	0.99280200
H	2.54865100	-1.80838200	0.71819500
H	2.34896100	-0.72526300	1.96412600

1- NH_2-8-OH-substituted

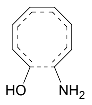

C	0.74382600	-0.81578700	0.69433900
C	0.10180800	-1.73202100	-0.14078300
C	-1.22885200	-1.64381600	-0.53772100
C	-2.19129000	-0.64949200	-0.29235200
C	-2.19776600	0.65093000	0.24124400
C	-1.23953200	1.64050300	0.53079000
C	0.09915200	1.74346100	0.17883900
C	0.76737600	0.84958200	-0.67133400
H	0.18028700	-0.29151300	1.45404300
H	0.70648800	-2.53025900	-0.57762600
H	-1.59746700	-2.49537100	-1.10592100
H	-3.19654100	-0.98339200	-0.54896800
H	-3.21063400	0.98871700	0.46003900
H	-1.62931700	2.47919800	1.10486300
H	0.17591900	0.34161900	-1.42509000
N	2.09397400	1.07468400	-1.03278700
H	2.69552400	1.31819600	-0.25228800
H	2.50984100	0.32726600	-1.57514000
H	0.68915400	2.53411300	0.64263100
O	2.05383300	-0.97411500	1.06244700
H	2.45992900	-1.67859800	0.53525600

1- NH_2-8-F-substituted

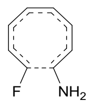

C	0.68961700	-0.88070400	0.70495900
C	0.04329400	-1.75342800	-0.14257900
C	-1.28878700	-1.60651200	-0.54835400
C	-2.20261600	-0.57551600	-0.31051400
C	-2.16235900	0.72576600	0.23777500
C	-1.17686000	1.67928400	0.52523000
C	0.17467500	1.74415800	0.18996900
C	0.82278300	0.85387900	-0.66883500
H	0.19423000	-0.32621700	1.48981100
H	0.64099700	-2.54992700	-0.58273900

H	-1.68925300	-2.44180800	-1.11855800
H	-3.21862000	-0.85917700	-0.58519200
H	-3.16297900	1.09339200	0.46335100
H	-1.54241100	2.53180500	1.09526700
H	0.22545500	0.33014900	-1.40672200
N	2.15354500	1.01157900	-1.01089500
H	2.74818800	1.37045600	-0.27250100
H	2.57746700	0.21172400	-1.46424200
H	0.77951700	2.51212000	0.67227500
F	1.99712400	-1.11945900	0.98440100

1- NH_2-8-CHO-substituted

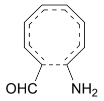

C	-0.59412900	-0.63880700	-0.67701600
C	-0.15168000	-1.65502700	0.19794300
C	1.16841600	-1.82395800	0.55111800
C	2.29418300	-1.01434400	0.23023700
C	2.52564300	0.26365400	-0.27065900
C	1.76495600	1.43863400	-0.53737700
C	0.48872600	1.80549300	-0.18540800
C	-0.36074900	1.03370400	0.65389600
H	0.10062700	-0.30953600	-1.44329700
H	-0.90641600	-2.29469200	0.65416400
H	1.40794600	-2.71148500	1.13298300
H	3.23111600	-1.53767900	0.42329600
H	3.58002000	0.42042300	-0.49483100
H	2.31530200	2.18739000	-1.10440200
H	0.12231500	0.36946900	1.36128200
N	-1.55890700	1.53646800	1.09989900
H	-2.04988400	2.14870700	0.45749700
H	-2.18888900	0.85016500	1.50427600
H	0.08732200	2.73266100	-0.59333300
C	-2.01437700	-0.50403800	-0.98533200
H	-2.26942800	-0.08418500	-1.97865900
O	-2.90545100	-0.74479800	-0.18533500

1-NH_2-8-CN-substituted

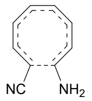

C	-0.61918300	-0.78459200	-0.53704800
C	-0.00204400	-1.72847600	0.30198300
C	1.35554800	-1.73739800	0.56550200
C	2.36272000	-0.83211700	0.15424900
C	2.42466400	0.44357700	-0.41109700
C	1.53368400	1.51986100	-0.65240900
C	0.25408400	1.78268400	-0.21132300
C	-0.45611200	1.00242900	0.73033000
H	-0.04027700	-0.36569900	-1.35139300
H	-0.63227100	-2.44121300	0.83082800
H	1.72036100	-2.58027000	1.14829900
H	3.35969700	-1.24624800	0.30593200
H	3.43744600	0.69076000	-0.72664600

H	1.95839200	2.29361000	-1.28954100
H	0.11839700	0.41153300	1.43522200
N	-1.68123900	1.41249900	1.19081200
H	-2.28691000	1.86469700	0.51413300
H	-2.19200300	0.74669400	1.75754900
H	-0.26416200	2.64084600	-0.63762300
C	-2.03382600	-0.81017400	-0.72927900
N	-3.18960100	-0.71956600	-0.79827000

1-NH_2-8-NO_2-substituted

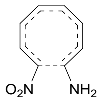

C	-0.42807300	-0.68681700	-0.53243500
C	0.13174100	-1.70202000	0.23419100
C	1.49541300	-1.83457200	0.44249100
C	2.55312800	-0.97264800	0.07981000
C	2.69555700	0.33874600	-0.38710800
C	1.88250100	1.48711000	-0.52856700
C	0.63119700	1.82733900	-0.04831200
C	-0.11940100	1.08411500	0.88196400
H	0.06763300	-0.22137300	-1.37001900
H	-0.54861500	-2.37732200	0.74777400
H	1.81038800	-2.74595600	0.94514400
H	3.52210300	-1.46251300	0.17837000
H	3.71602400	0.53523200	-0.71281800
H	2.35139800	2.26674300	-1.12689300
H	0.38770500	0.37455300	1.52685700
N	-1.32454700	1.51778300	1.33225400
H	-1.87770100	2.09249500	0.70478800
H	-1.88132100	0.86764200	1.87501600
H	0.17491100	2.73649500	-0.43674400
N	-1.84329400	-0.51913400	-0.56118400
O	-2.52146000	-1.04776500	0.31996600
O	-2.30354300	0.25975800	-1.39261300

1-OH-8-OH-substituted

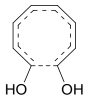

C	-0.75381200	0.83051100	0.70116000
C	-0.11740600	1.73893500	-0.13754200
C	1.22900100	1.65587900	-0.49914500
C	2.18092900	0.65952400	-0.25465300
C	2.18113400	-0.65909800	0.25450100
C	1.22944500	-1.65558200	0.49936400
C	-0.11695200	-1.73895200	0.13776700
C	-0.75340300	-0.83077200	-0.70116000
H	-0.18975900	0.31086300	1.46459900
H	-0.72490000	2.51685600	-0.60224400
H	1.60997800	2.51489400	-1.04783800
H	3.19109700	0.99389200	-0.48994600
H	3.19143500	-0.99321800	0.48958500
H	1.61064000	-2.51443400	1.04815800
H	-0.18925500	-0.31105400	-1.46450000

O	-2.07139000	-0.92611700	-1.03265500
H	-2.55143600	-1.37879300	-0.32149200
H	-0.72430600	-2.51698700	0.60244300
O	-2.07180900	0.92559900	1.03237200
H	-2.55151700	1.37946000	0.32174200

1-OH-8-F-substituted

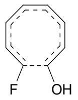

C	0.69127500	-0.89517000	0.68850100
C	0.04487400	-1.75891100	-0.16332600
C	-1.29634600	-1.60013600	-0.54287900
C	-2.19235600	-0.56253900	-0.28807100
C	-2.13010800	0.73992600	0.26845100
C	-1.13943100	1.68781300	0.53224900
C	0.21146800	1.74069300	0.16865900
C	0.82112000	0.83903900	-0.69214200
H	0.18366500	-0.33372700	1.46182200
H	0.63777900	-2.54528900	-0.62542700
H	-1.71413300	-2.42849800	-1.11069100
H	-3.21574600	-0.83120300	-0.54924300
H	-3.12370700	1.10911100	0.52076700
H	-1.48775600	2.54797300	1.10072900
H	0.23824100	0.32174000	-1.44329800
O	2.12865900	0.90088100	-1.03550000
H	2.63051200	1.39124600	-0.36624200
H	0.83756500	2.49663600	0.64424300
F	1.99125900	-1.11992400	0.98030000

1-OH-8-CHO-substituted

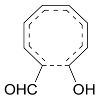

C	-0.66815700	-0.61708600	-0.62513100
C	-0.26247800	-1.62665800	0.24669900
C	1.06724400	-1.87161700	0.56838300
C	2.22007300	-1.14052900	0.22516600
C	2.51925700	0.11425400	-0.33418600
C	1.84477700	1.32346500	-0.58762800
C	0.59814500	1.79823000	-0.19925100
C	-0.26531400	1.13385800	0.67835500
H	0.03321300	-0.27799700	-1.37900600
H	-1.03950200	-2.20538300	0.74410700
H	1.26364500	-2.76990000	1.15012500
H	3.13119800	-1.70296000	0.43071700
H	3.57059900	0.18183800	-0.61107200
H	2.42875900	2.02951100	-1.17512400
H	0.13160900	0.43946400	1.40674200
O	-1.42961500	1.66837700	1.10070800
H	-1.71678600	2.38313400	0.51098000
H	0.25333900	2.73679000	-0.63458200
C	-2.09728600	-0.40701300	-0.91260700
H	-2.32019800	0.21793500	-1.80248300

O	-3.00456500	-0.82760800	-0.22560800

1-OH-8-CN-substituted

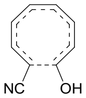

C	-0.63150300	-0.80382800	-0.50726100
C	-0.01726800	-1.73350200	0.32946600
C	1.35817700	-1.74292900	0.54810400
C	2.33764000	-0.83132400	0.12150100
C	2.37836600	0.44880000	-0.46754700
C	1.49567500	1.52256300	-0.67695300
C	0.21854400	1.78587700	-0.19076600
C	-0.41738000	1.00453800	0.77530800
H	-0.04507900	-0.37347800	-1.31056400
H	-0.63804500	-2.42790300	0.89118200
H	1.74336200	-2.58885700	1.11287800
H	3.34354600	-1.22699300	0.26199200
H	3.37966500	0.68206600	-0.82730300
H	1.90236800	2.30085400	-1.31976200
H	0.17401300	0.42010000	1.46938100
O	-1.63041100	1.28709500	1.28185100
H	-2.17576900	1.77290800	0.64163800
H	-0.33142200	2.62504300	-0.61585200
C	-2.04763900	-0.78664900	-0.70958400
N	-3.19386100	-0.66596900	-0.84171400

1-OH-8-NO_2-substituted

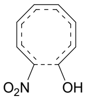

C	-0.46768600	-0.66348800	-0.47719400
C	0.06616700	-1.70252700	0.25346500
C	1.44093100	-1.88127700	0.42381900
C	2.50762100	-1.05148900	0.06127700
C	2.68666500	0.26124200	-0.42991700
C	1.93154300	1.43422400	-0.54968700
C	0.68945500	1.82215700	-0.04348200
C	-0.04407100	1.11045900	0.89703600
H	0.03913300	-0.17367200	-1.29267300
H	-0.62234000	-2.35913000	0.77976400
H	1.73585500	-2.81125600	0.90383500
H	3.46610300	-1.56095000	0.16119400
H	3.70368900	0.40502200	-0.79200100
H	2.42238900	2.20228600	-1.14480400
H	0.43647600	0.41606100	1.57637000
O	-1.24290700	1.50588800	1.34855500
H	-1.70359500	2.04080800	0.67842300
H	0.24131700	2.72885800	-0.44783600
N	-1.89906400	-0.47042500	-0.52247200
O	-2.62997700	-1.21358200	0.10834700
O	-2.28828200	0.51133800	-1.15401200

1-F-8-F-substituted

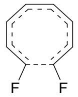

C	0.76788300	-0.90832400	0.67696400
C	0.13167500	-1.76313600	-0.18608700
C	-1.21853500	-1.63834700	-0.56010000
C	-2.15674500	-0.64723200	-0.29416800
C	-2.15688000	0.64685000	0.29416800
C	-1.21887500	1.63814400	0.56012100
C	0.13131800	1.76318700	0.18609500
C	0.76752500	0.90852000	-0.67693700
H	0.26386900	-0.34499000	1.45133400
H	0.73891600	-2.53033900	-0.66142700
H	-1.60422000	-2.47481300	-1.13899400
H	-3.16550600	-0.95413800	-0.56891200
H	-3.16567800	0.95347700	0.56909000
H	-1.60468600	2.47443600	1.13918500
H	0.26350600	0.34500100	-1.45117300
F	2.06929300	1.11098500	-0.94644100
H	0.73848500	2.53032000	0.66163200
F	2.06972000	-1.11064400	0.94632200

1-F-8-CHO-substituted

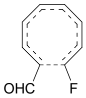

C	-0.73230700	-0.60750400	-0.62930600
C	-0.34105500	-1.61078900	0.23742300
C	0.99081500	-1.91131300	0.55106200
C	2.16828200	-1.23420000	0.23102400
C	2.53127100	0.01224800	-0.33752300
C	1.93205100	1.24940100	-0.57119400
C	0.69744000	1.79594200	-0.18865100
C	-0.19498900	1.19978000	0.66962900
H	-0.03441700	-0.24717800	-1.37605000
H	-1.12848600	-2.15893600	0.75305400
H	1.14394400	-2.82672200	1.11946400
H	3.05354500	-1.82913300	0.45596100
H	3.58012000	0.01436600	-0.63197400
H	2.55568000	1.93242400	-1.14449200
H	0.07156400	0.48536300	1.43666600
F	-1.33683900	1.84991900	0.94781600
H	0.38557100	2.73620700	-0.63802900
C	-2.17052700	-0.34289500	-0.86184700
H	-2.40707700	0.33714200	-1.70293100
O	-3.05934700	-0.79960200	-0.17571500

1-F-8-CN-substituted

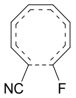

C	-0.73700800	-0.76614600	-0.47860500

C	-0.15972200	-1.69911100	0.36940600
C	1.21991200	-1.80075300	0.58263800
C	2.26765500	-0.98968300	0.14225000
C	2.41534300	0.26607000	-0.49549800
C	1.63875400	1.40568500	-0.70913100
C	0.38552200	1.79466800	-0.21753200
C	-0.31771200	1.10144100	0.74068100
H	-0.14604800	-0.35373600	-1.28778800
H	-0.81348100	-2.34252500	0.95403900
H	1.53575900	-2.66479800	1.16303600
H	3.23809400	-1.45836500	0.30470800
H	3.42488500	0.38916700	-0.88546300
H	2.10637700	2.13851100	-1.36330700
H	0.13989100	0.46116500	1.48400200
F	-1.50524200	1.57993900	1.13080900
H	-0.10272100	2.66187700	-0.65556000
C	-2.15907700	-0.72345900	-0.65930100
N	-3.30822700	-0.65471700	-0.79148500

1-F-8-NO_2-substituted

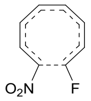

C	0.53456700	-0.63163200	0.44247600
C	0.03522300	-1.65938300	-0.31609300
C	-1.33858600	-1.90512400	-0.46822800
C	-2.44005300	-1.15698200	-0.06190300
C	-2.68197100	0.12585400	0.49788500
C	-2.00831600	1.33792400	0.61414400
C	-0.80270000	1.81278000	0.06773800
C	-0.06907700	1.14736200	-0.87938800
H	0.02160400	-0.19091000	1.28355900
H	0.74301100	-2.25962000	-0.88258900
H	-1.58756800	-2.83671100	-0.97083300
H	-3.37119200	-1.71283100	-0.17096700
H	-3.68737100	0.18393700	0.91270500
H	-2.52650700	2.06638000	1.23433000
H	-0.47085000	0.41811000	-1.57126100
F	1.07412400	1.69186200	-1.30862500
H	-0.38161400	2.73590300	0.45810100
N	1.97327200	-0.42239600	0.50914600
O	2.68268400	-0.95773700	-0.32699500
O	2.36806000	0.32035700	1.39459200

1-CHO-8-CHO-substituted

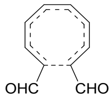

C	0.32464300	0.95730200	-0.61447200
C	-0.37219700	1.79195100	0.24353900
C	-1.72840500	1.66266700	0.55771600
C	-2.65407400	0.65347800	0.27113900
C	-2.65437100	-0.65262700	-0.27118400
C	-1.72915000	-1.66225000	-0.55764000
C	-0.37295600	-1.79193800	-0.24358400

C	0.32406600	-0.95740100	0.61438100
H	-0.21575400	0.38913600	-1.36232500
H	0.19441300	2.57706200	0.74196100
H	-2.15271800	2.49596100	1.11514100
H	-3.66988600	0.96666400	0.51167700
H	-3.67032000	-0.96533100	-0.51177300
H	-2.15381400	-2.49542800	-1.11497100
H	-0.21622600	-0.38920700	1.36228700
C	1.76641000	-1.20755300	0.87595600
H	2.20526700	-0.66836800	1.73568300
O	2.45294800	-1.93003500	0.18585200
H	0.19339600	-2.57726000	-0.74195800
C	1.76703400	1.20719900	-0.87602400
H	2.20563300	0.66830300	-1.73607700
O	2.45380200	1.92922200	-0.18567800

1-CHO-8-CN-substituted

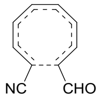

C	0.28874500	1.09094000	-0.46349800
C	-0.51537800	1.80678600	0.41488500
C	-1.86208800	1.52212200	0.65553500
C	-2.67948500	0.46619600	0.23737100
C	-2.53859900	-0.77843700	-0.41774100
C	-1.51225700	-1.67654900	-0.73257800
C	-0.17301000	-1.71724200	-0.33658500
C	0.38598400	-0.89259400	0.62618600
H	-0.17042600	0.54031200	-1.27532500
H	-0.05841500	2.60844300	0.99067800
H	-2.38111800	2.26414400	1.25881900
H	-3.72843000	0.67045700	0.45025100
H	-3.50880000	-1.15051400	-0.74592800
H	-1.82605800	-2.48668600	-1.38816500
H	-0.25448300	-0.43753400	1.37291600
C	1.82928900	-1.02568400	0.96525100
H	2.18166800	-0.42798100	1.82698400
O	2.59814000	-1.71770100	0.33820400
H	0.49665800	-2.40322300	-0.85303500
C	1.64652200	1.50591200	-0.68560000
N	2.74942300	1.82221300	-0.84602600

1-CHO-8-NO_2-substituted

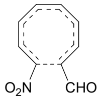

C	0.21834700	-0.90363900	0.40250700
C	-0.46185700	-1.73805100	-0.44799400
C	-1.85061200	-1.67500500	-0.64182200
C	-2.79708600	-0.74702000	-0.21272300
C	-2.81694400	0.51409600	0.44020200
C	-1.92952900	1.55493800	0.71001100
C	-0.61913000	1.79372200	0.26646600
C	0.01084000	1.07649200	-0.72934100
H	-0.21885800	-0.43386200	1.27091200

H	0.11972800	-2.43595500	-1.04537000
H	-2.26743300	-2.49805500	-1.21809700
H	-3.81209200	-1.09798800	-0.39720200
H	-3.81776800	0.73429500	0.81088500
H	-2.33554500	2.31414400	1.37582600
H	-0.58210600	0.52176400	-1.44822200
C	1.41391300	1.40176500	-1.11171900
H	1.78506100	0.94621500	-2.04740600
O	2.13197600	2.10101600	-0.43324800
H	-0.03718600	2.56014200	0.77596800
N	1.66191500	-1.06137900	0.54253600
O	2.14653900	-0.69048300	1.59629000
O	2.28712800	-1.51613800	-0.40411300

1-CN-8-CN-substituted

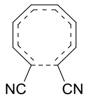

C	0.36885900	1.01666300	-0.47832000
C	-0.29388900	1.73357900	0.51196400
C	-1.63840500	1.54008400	0.83346900
C	-2.56732500	0.59279700	0.38393200
C	-2.56731000	-0.59270600	-0.38407300
C	-1.63843300	-1.54015600	-0.83336300
C	-0.29392200	-1.73367600	-0.51183100
C	0.36882900	-1.01672000	0.47842300
H	-0.20897300	0.58031700	-1.28462700
H	0.27730300	2.44043200	1.10888200
H	-2.04986900	2.26084100	1.53670300
H	-3.58121800	0.85762000	0.68228500
H	-3.58117500	-0.85739000	-0.68264200
H	-2.04994300	-2.26104400	-1.53643700
H	-0.20892800	-0.58051100	1.28485800
C	1.73750300	-1.31435500	0.79336100
N	2.84622400	-1.53403900	1.04625100
H	0.27724500	-2.44061400	-1.10867700
C	1.73737200	1.31439500	-0.79341100
N	2.84604600	1.53417000	-1.04643000

1-CN-8-NO_2-substituted

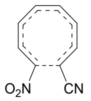

C	0.26441000	-0.84266900	0.40374800
C	-0.31694000	-1.67015700	-0.52742700
C	-1.69094300	-1.66300300	-0.79800000
C	-2.70959200	-0.81958900	-0.34985800
C	-2.82859900	0.38328500	0.38947900
C	-2.01444700	1.45004500	0.77674600
C	-0.71197200	1.80578500	0.40562900
C	0.00073100	1.16871700	-0.59778500
H	-0.25360400	-0.45237200	1.26703100
H	0.33650800	-2.30306800	-1.12206200
H	-2.03231700	-2.45940800	-1.45521400
H	-3.69359500	-1.21016800	-0.60758000

H	-3.85521500	0.52440200	0.72600800
H	-2.48959600	2.13303500	1.47771100
H	-0.53455100	0.64003500	-1.37807600
C	1.32396800	1.60633800	-0.94156700
N	2.40312200	1.92863200	-1.21145300
H	-0.21340300	2.58243300	0.98014800
N	1.70537400	-0.92559800	0.63284300
O	2.11360900	-0.38942300	1.65002800
O	2.39596600	-1.48415700	-0.20046300

1-NO_2-8-NO_2-substituted

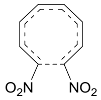

C	-0.02183900	1.06388000	-0.39967000
C	-0.64206200	1.67903800	0.65650800
C	-1.99627100	1.46339000	0.96307200
C	-2.91654700	0.55679300	0.44223300
C	-2.91649200	-0.55704400	-0.44217600
C	-1.99611000	-1.46351700	-0.96305500
C	-0.64187900	-1.67905100	-0.65650800
C	-0.02164800	-1.06387000	0.39966100
H	-0.53898400	0.71730500	-1.28319500
H	-0.03850000	2.29902300	1.31453000
H	-2.40460200	2.12493100	1.72345600
H	-3.92984400	0.77864700	0.77539600
H	-3.92978600	-0.77911100	-0.77520700
H	-2.40440900	-2.12514300	-1.72338300
H	-0.53878400	-0.71735800	1.28321400
N	1.38392800	-1.35378700	0.67786200
O	1.74628200	-1.18443800	1.82904300
O	2.08935700	-1.71700600	-0.24710900
H	-0.03823700	-2.29886100	-1.31462800
N	1.38372600	1.35384500	-0.67788500
O	2.08919000	1.71684300	0.24715800
O	1.74600200	1.18490700	-1.82914300

5.3.9 2,3-substituted

2-CH_3-3-CH_3-substituted

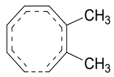

C	-1.37849100	-1.37811700	0.84968400
C	0.02726100	-1.25439100	-0.97982000
C	-2.44767300	-0.74654300	0.26969300
C	-2.46027700	0.62047800	-0.09427100
C	-1.43548400	1.54945200	-0.17420900
C	-0.00980000	1.60023700	-0.04385700
C	1.08187400	0.73980300	-0.04173500
C	1.09424000	-0.65534900	-0.36243300
H	-0.63161800	-0.79727200	1.37598100
H	-1.42869800	-2.43549100	1.09576300
H	-0.71462900	-0.65013600	-1.48615400
H	0.07365500	-2.30392800	-1.25955500
H	-3.32207000	-1.33538800	-0.00316600

H	-3.43774700	1.01741400	-0.36425200
H	-1.82240200	2.55188700	-0.35949300
C	2.26561900	-1.50030300	0.08198800
H	2.11916400	-2.54419300	-0.20763500
H	3.20496800	-1.15769100	-0.36655100
H	2.40064000	-1.46531800	1.17087200
H	0.30212700	2.63939500	0.06430500
C	2.41928000	1.35111300	0.33341900
H	2.80731000	0.91571800	1.26252100
H	3.17579200	1.17759300	-0.44074600
H	2.33421200	2.42912400	0.48736300

2-CH_3-3-NH_2-substituted

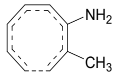

C	1.34482700	-1.42348300	-0.83438800
C	-0.07219200	-1.24453400	0.96458700
C	2.41837700	-0.79436300	-0.25454800
C	2.45558000	0.57271000	0.09966100
C	1.44873500	1.52794700	0.16464900
C	0.03248600	1.61528000	0.05451200
C	-1.07596000	0.76471700	0.03156000
C	-1.13255400	-0.62544600	0.35479100
H	0.60961900	-0.84320200	-1.37683500
H	1.39446700	-2.48200100	-1.07596500
H	0.67821200	-0.65687200	1.47795200
H	-0.14156500	-2.29303500	1.24218100
H	3.28469000	-1.39274800	0.02523700
H	3.43795800	0.95137900	0.37626300
H	1.86054600	2.52157300	0.34390000
C	-2.33349400	-1.42377800	-0.09085300
H	-2.14583900	-2.49360300	0.03365400
H	-3.22754800	-1.18513200	0.50115800
H	-2.57992000	-1.22413800	-1.13957900
H	-0.27178400	2.66057500	-0.02385400
N	-2.29007800	1.32288300	-0.41331200
H	-2.26919800	2.32810700	-0.53311200
H	-3.11392600	1.03461700	0.10235800

2-CH_3-3-OH-substituted

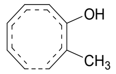

C	-1.30589600	-1.43765900	0.84004100
C	0.11175600	-1.26318300	-0.94772800
C	-2.39078200	-0.83385100	0.25340400
C	-2.45562100	0.52991900	-0.11091900
C	-1.47143100	1.50645700	-0.17478300
C	-0.05733400	1.61074400	-0.04220000
C	1.05037100	0.77001700	-0.03195200
C	1.16140900	-0.61012000	-0.35331100
H	-0.58549100	-0.84010300	1.38354800
H	-1.33720600	-2.49453100	1.09105500
H	-0.65264600	-0.69969600	-1.46695800
H	0.21173600	-2.31071200	-1.21918600
H	-3.24338800	-1.45149200	-0.02575100

H	-3.44429800	0.88492900	-0.39596900
H	-1.89727000	2.49230800	-0.36184500
C	2.40618300	-1.34088600	0.07964600
H	2.33156400	-2.40216100	-0.17164200
H	3.29173300	-0.92788800	-0.41394300
H	2.57314300	-1.24297100	1.15854700
H	0.24009000	2.65863100	0.04661700
O	2.26127900	1.31622500	0.33010700
H	2.12988000	2.21525200	0.66148700

2-CH_3-3-F-substituted

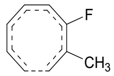

C	1.27492100	-1.45456500	-0.83884300
C	-0.17685800	-1.28593400	0.95187200
C	2.36211400	-0.87985200	-0.23202600
C	2.46204300	0.48574900	0.12598700
C	1.51080200	1.49177200	0.16406000
C	0.09934000	1.61609500	0.00506600
C	-0.99712000	0.78269800	0.03217900
C	-1.18902300	-0.58363600	0.34991700
H	0.57041800	-0.83802600	-1.38277000
H	1.28344100	-2.51137500	-1.09109400
H	0.60619800	-0.76001900	1.48193600
H	-0.32047700	-2.33275100	1.20542900
H	3.19340500	-1.51924800	0.06128900
H	3.45856000	0.81158000	0.41902800
H	1.95612300	2.47045100	0.33987800
C	-2.46635900	-1.24338900	-0.10402700
H	-2.45097300	-2.30772600	0.14342500
H	-3.33430200	-0.78849600	0.38338100
H	-2.61345600	-1.13535700	-1.18457800
H	-0.21344200	2.64906800	-0.14137800
F	-2.15718400	1.40980800	-0.32885100

2-CH_3-3-CHO-substituted

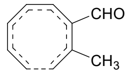

C	1.91385900	0.97859600	0.94182000
C	0.59721800	1.41637600	-0.97340300
C	2.77279400	0.10915200	0.32781100
C	2.40396200	-1.18732200	-0.10402300
C	1.15700900	-1.77446700	-0.24063400
C	-0.21344500	-1.38581700	-0.14244900
C	-0.98496200	-0.22979200	-0.13033900
C	-0.59798500	1.13328000	-0.37547200
H	1.00786100	0.60440500	1.40274600
H	2.24516800	1.96668800	1.24873400
H	1.14299600	0.64176800	-1.49732200
H	0.88323000	2.44480100	-1.17924800
H	3.78533700	0.43699600	0.09951400
H	3.23384900	-1.83905300	-0.37309700
H	1.23163700	-2.84059900	-0.45300100
C	-1.45574800	2.27993200	0.12245300
H	-0.88319400	3.21056900	0.08148000

H	-2.34984500	2.42462500	-0.49409800
H	-1.78725700	2.13267800	1.15612200
H	-0.85504600	-2.26484800	-0.07946800
C	-2.43719300	-0.47729600	0.12950900
H	-3.08665500	0.41619700	0.11798400
O	-2.93764200	-1.56376000	0.32975300

2-CH_3-3-CN-substituted

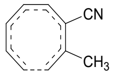

C	1.72966100	1.15671500	0.94565800
C	0.39343500	1.38265000	-0.98097100
C	2.70741300	0.40458800	0.35275400
C	2.53340900	-0.93418500	-0.07150900
C	1.38933900	-1.70013700	-0.21798800
C	-0.02434700	-1.52159400	-0.12903000
C	-0.93605900	-0.47017200	-0.14266200
C	-0.76004400	0.92970700	-0.40563600
H	0.87890900	0.67224600	1.40880200
H	1.92403400	2.18338800	1.24314000
H	1.06007600	0.69473600	-1.48485000
H	0.51663100	2.43892200	-1.20402700
H	3.66741800	0.86848600	0.13368400
H	3.45149600	-1.46002800	-0.32840600
H	1.62068400	-2.74425800	-0.42481200
C	-1.83030100	1.89048900	0.05068400
H	-1.54113400	2.92011300	-0.17290500
H	-2.78194800	1.68537200	-0.45144700
H	-2.01439200	1.80462400	1.12801400
H	-0.52507200	-2.48595700	-0.05370100
C	-2.29459500	-0.86942800	0.15956900
N	-3.38631100	-1.16992100	0.40589800

2-CH_3-3-NO_2-substituted

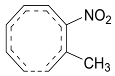

C	2.12614400	1.02707100	0.95311700
C	0.78038100	1.46502300	-0.91051500
C	3.02092600	0.22517000	0.29844500
C	2.71837600	-1.07650500	-0.16751000
C	1.50490200	-1.72457900	-0.31074000
C	0.11640700	-1.41153000	-0.18836300
C	-0.68140200	-0.28524500	-0.15817200
C	-0.40570100	1.10653000	-0.33364200
H	1.25475400	0.59037400	1.42551700
H	2.41108500	2.02124800	1.28588400
H	1.34643300	0.75145700	-1.49601800
H	1.02457300	2.51544600	-1.04461300
H	4.00915300	0.61667500	0.06438200
H	3.57800200	-1.67432000	-0.46553500
H	1.62842100	-2.77666500	-0.56273200
C	-1.30015200	2.17506700	0.25708800
H	-0.69852800	3.06207400	0.47740200
H	-2.09187500	2.45877800	-0.44027500

H	-1.78199800	1.84945000	1.18371800
H	-0.48007900	-2.31919700	-0.14114800
N	-2.11566800	-0.61478600	0.07695100
O	-2.94486300	0.09416200	-0.46842900
O	-2.38883100	-1.56889200	0.78549300

2- NH_2-3-CH_3-substituted

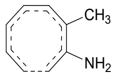

C	-1.42628500	-1.35299600	0.86553200
C	0.03270700	-1.27269800	-1.01793200
C	-2.46492300	-0.68048500	0.28878700
C	-2.42495300	0.68840400	-0.08807400
C	-1.37487800	1.57892700	-0.19296800
C	0.05678900	1.58660900	-0.05404300
C	1.11941100	0.70347300	-0.04233000
C	1.08336000	-0.70269200	-0.35585700
H	-0.62889500	-0.81794800	1.36562600
H	-1.51327400	-2.41039000	1.10155500
H	-0.68023700	-0.64228100	-1.53005000
H	0.04606300	-2.32855000	-1.27568400
H	-3.36592700	-1.23177700	0.02465400
H	-3.39064800	1.11792300	-0.35198900
H	-1.72588600	2.58896300	-0.40498900
H	0.39852700	2.61708200	0.04927600
C	2.47388000	1.26921000	0.33942100
N	2.09745400	-1.52201400	0.16836100
H	2.28523900	-2.35437200	-0.37655600
H	2.95562200	-1.05688400	0.42875100
H	2.78744900	0.92448400	1.33425100
H	3.25460100	0.98111000	-0.37570000
H	2.44454200	2.36022100	0.37710000

2- NH_2-3-NH_2-substituted

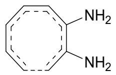

C	-1.38943900	-1.40303000	0.80210800
C	0.07111600	-1.27042400	-1.01569600
C	-2.43497900	-0.71761600	0.24704900
C	-2.41770900	0.66565700	-0.06516500
C	-1.38092400	1.57763900	-0.13728500
C	0.04917800	1.60366400	-0.04772800
C	1.11574000	0.72185700	-0.04751200
C	1.10824100	-0.68674500	-0.34026800
H	-0.61527800	-0.88077100	1.35010100
H	-1.47054900	-2.46924500	0.99736500
H	-0.61396100	-0.64888000	-1.57659400
H	0.09430800	-2.33126900	-1.24850300
H	-3.32531800	-1.27106200	-0.04816500
H	-3.39011200	1.09168500	-0.30860200
H	-1.75046300	2.58862300	-0.30838100
H	0.39629500	2.63616700	0.02322200
N	2.11870300	-1.46427300	0.23079000

H	2.46666300	-2.23664300	-0.31979200
H	2.83475900	-0.95046800	0.72614800
N	2.39220800	1.21407000	0.35127100
H	2.36528700	2.18823100	0.63078800
H	3.10464600	1.08904100	-0.36503200

2- NH_2-3-OH-substituted

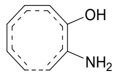

C	-1.34704300	-1.41115500	0.85208800
C	0.11456300	-1.29128800	-0.97013100
C	-2.40826200	-0.77288300	0.26921900
C	-2.42705800	0.59499400	-0.10524700
C	-1.41869900	1.53736400	-0.19239900
C	0.00411700	1.59937300	-0.05232000
C	1.08174500	0.73752800	-0.03570300
C	1.15601400	-0.65850300	-0.34685500
H	-0.58409100	-0.85114100	1.37767000
H	-1.40771200	-2.46913500	1.09348600
H	-0.61875900	-0.70331800	-1.50378500
H	0.18298600	-2.34800100	-1.21271900
H	-3.28361200	-1.36012600	-0.00412600
H	-3.40693400	0.97925200	-0.38437900
H	-1.81051800	2.53179400	-0.40483400
H	0.33280400	2.63841000	0.03219000
N	2.24818900	-1.36569600	0.16565400
H	2.54709200	-2.14540100	-0.40602300
H	3.02467500	-0.77723600	0.44019900
O	2.31759800	1.24112100	0.32431800
H	2.21369900	2.12322200	0.70629400

2- NH_2-3-F-substituted

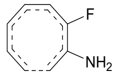

C	1.31708800	-1.43009000	-0.85277300
C	-0.18788200	-1.31405200	0.97198200
C	2.38104100	-0.82525300	-0.24462400
C	2.43779800	0.54535800	0.12406100
C	1.46260600	1.52063600	0.18193800
C	0.04302900	1.60520400	0.01466300
C	-1.02852000	0.75432100	0.03278500
C	-1.19043300	-0.63058300	0.34006000
H	0.57184200	-0.84623500	-1.37852500
H	1.35135300	-2.48834300	-1.09700400
H	0.56692100	-0.76410600	1.51570600
H	-0.30128000	-2.37069100	1.19672600
H	3.23370700	-1.43747900	0.04435600
H	3.42642300	0.89866500	0.41328900
H	1.87544000	2.50859700	0.38150200
H	-0.29695800	2.63056400	-0.12669400
N	-2.31414900	-1.27417700	-0.18728400
H	-2.66972200	-2.02972400	0.38441700
H	-3.05282700	-0.64847000	-0.48217000

F	-2.21269200	1.34591300	-0.32768400

2- NH_2-3-CHO-substituted

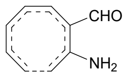

C	2.02679700	0.95979600	0.85561100
C	0.57427600	1.42451000	-1.08404500
C	2.78553500	0.00380400	0.25593000
C	2.32460800	-1.30385500	-0.06305500
C	1.05430600	-1.83463000	-0.14266300
C	-0.30160800	-1.37192200	-0.07178500
C	-1.00977100	-0.18699700	-0.10853700
C	-0.53270200	1.16505400	-0.33769300
H	1.10351400	0.70446400	1.36180000
H	2.42824400	1.94903900	1.05819300
H	1.02307700	0.63571400	-1.67363900
H	0.91121100	2.44378600	-1.25304000
H	3.80345600	0.24825900	-0.04232600
H	3.11292900	-2.01973500	-0.29375800
H	1.07202500	-2.91198100	-0.30105300
H	-0.98877800	-2.21597800	-0.00867200
C	-2.48830200	-0.34278900	-0.00735700
N	-1.16823300	2.19773000	0.35828300
H	-1.25364500	3.08435900	-0.12119000
H	-1.98610000	1.96440700	0.90296500
H	-3.07816000	0.55403000	-0.28463800
O	-3.07112200	-1.36228600	0.29611700

2-NH_2-3-CN-substituted

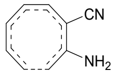

C	1.77958400	1.14297500	0.96079700
C	0.35560500	1.38982500	-1.05352800
C	2.71726000	0.36385200	0.35699600
C	2.50278100	-0.97754900	-0.06773200
C	1.34950300	-1.71674200	-0.22053400
C	-0.06607300	-1.51067100	-0.11548300
C	-0.95931100	-0.45496200	-0.13300200
C	-0.75482600	0.95674900	-0.39646500
H	0.88531900	0.71232600	1.39455300
H	2.00164800	2.16976000	1.23780900
H	0.99249400	0.68020300	-1.56284500
H	0.50480500	2.44591600	-1.25849300
H	3.69144600	0.79357200	0.13139000
H	3.40935500	-1.52601400	-0.31945700
H	1.55624700	-2.76229400	-0.44395500
H	-0.58176100	-2.46675600	-0.03386500
C	-2.32748300	-0.82017100	0.15130200
N	-3.43578300	-1.06509000	0.38817400
N	-1.66405500	1.85612400	0.16235400
H	-1.79591900	2.71020800	-0.36416900
H	-2.54700900	1.46599900	0.47123000

2-NH_2-3-NO_2-substituted

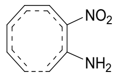

C	2.23418600	1.08721500	0.81819500
C	0.77395100	1.26478500	-1.20705400
C	3.04029400	0.12781100	0.29561700
C	2.63747600	-1.21632700	0.04823200
C	1.39410800	-1.80043900	-0.03549900
C	0.01358600	-1.40182600	-0.01975900
C	-0.73252600	-0.25317900	-0.10697500
C	-0.33617100	1.10849500	-0.43432100
H	1.30402600	0.83733500	1.31439000
H	2.59056500	2.10456900	0.95531200
H	1.23587600	0.41379100	-1.68907500
H	1.11636700	2.25674300	-1.48709200
H	4.05482300	0.39190500	0.00320300
H	3.45706500	-1.91437900	-0.11709600
H	1.45692900	-2.88247800	-0.13502000
H	-0.62902200	-2.27443200	0.05548200
N	-0.95251900	2.21168500	0.14269300
H	-0.87925900	3.06189700	-0.40076300
H	-1.87390100	2.05662400	0.52789800
N	-2.18365200	-0.50334100	0.10497500
O	-2.85269600	0.38441100	0.62199500
O	-2.65101500	-1.57306000	-0.23593600

2-OH-3-CH_3-substituted

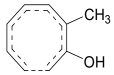

C	-1.42795400	-1.32275800	0.88158400
C	0.01421000	-1.32110000	-0.94240600
C	-2.46496200	-0.65318800	0.28611200
C	-2.40899500	0.70021300	-0.12259800
C	-1.34894600	1.58747500	-0.21823700
C	0.07432100	1.58755100	-0.05707300
C	1.13019600	0.69081200	-0.02874100
C	1.07507400	-0.69932100	-0.34455300
H	-0.65024600	-0.77253900	1.39631600
H	-1.53491700	-2.36917700	1.15563700
H	-0.70569300	-0.71997900	-1.47939000
H	0.05814000	-2.37932100	-1.19015500
H	-3.36832500	-1.20430900	0.03032200
H	-3.36532900	1.12909800	-0.41893100
H	-1.69294600	2.59675300	-0.44469500
H	0.42878900	2.61394700	0.04246200
C	2.49567800	1.21052100	0.36834000
O	2.16272200	-1.41066600	0.10280300
H	2.14577300	-2.29916100	-0.27920100
H	2.80629800	0.78620200	1.32958000
H	3.25941700	0.92377600	-0.36151300
H	2.48553500	2.29881600	0.46257400

2-OH-3-NH_2-substituted

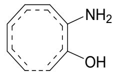

C	-1.38862000	-1.37555800	0.86636100
C	0.04990300	-1.30419100	-0.91736000
C	-2.43414500	-0.70333600	0.27971100
C	-2.40481500	0.64995700	-0.12060100
C	-1.36190600	1.56671200	-0.20976100
C	0.05207600	1.60714500	-0.07573500
C	1.12779300	0.72175500	-0.01821000
C	1.11359000	-0.66389200	-0.34504000
H	-0.62274700	-0.82845500	1.40111100
H	-1.49969600	-2.42266800	1.13688500
H	-0.67515700	-0.71635100	-1.46278300
H	0.11248000	-2.36166200	-1.16234500
H	-3.33275100	-1.26340900	0.02426300
H	-3.36821500	1.05941200	-0.41969500
H	-1.73514100	2.56684100	-0.43109500
H	0.40750500	2.63768300	-0.02902500
O	2.22770000	-1.34800600	0.09647600
H	2.27415700	-2.20722800	-0.34417900
N	2.37960000	1.25418400	0.31763000
H	3.08140700	0.55621400	0.52768900
H	2.35609600	1.99283800	1.00777300

2-OH-3-OH-substituted

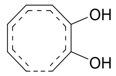

C	1.35638800	-1.38539900	-0.84734100
C	-0.08726900	-1.29956900	0.92052600
C	2.41124600	-0.73137300	-0.25862400
C	2.40081900	0.62882900	0.12296400
C	1.37034500	1.55563600	0.19678400
C	-0.04695300	1.59854600	0.05148200
C	-1.12201200	0.72271700	0.01391800
C	-1.14738300	-0.66479800	0.33076900
H	0.60989500	-0.82656400	-1.39746700
H	1.44334100	-2.43808300	-1.10424500
H	0.62953400	-0.70892400	1.47393500
H	-0.15291300	-2.35550100	1.17088200
H	3.29496000	-1.30595600	0.01443200
H	3.36817500	1.03024500	0.42015400
H	1.74644100	2.55588800	0.41025900
H	-0.39059000	2.63351000	-0.01776500
O	-2.24876400	-1.33111300	-0.12664600
H	-2.27797000	-2.20890600	0.27857000
O	-2.35380200	1.20299900	-0.34146300
H	-2.26143800	2.10168300	-0.68675600

2-OH-3-F-substituted

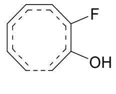

C	1.32064200	-1.40562400	-0.84791200

C	-0.16307300	-1.32714800	0.92142500
C	2.38133700	-0.79053500	-0.23359000
C	2.41326100	0.57327700	0.14250000
C	1.41948300	1.53696000	0.18639200
C	0.00498600	1.60653200	0.01259000
C	-1.06568800	0.74472800	0.01167900
C	-1.18145000	-0.63575100	0.32392100
H	0.59393800	-0.81861100	-1.39594500
H	1.37588200	-2.45908900	-1.10914500
H	0.57599800	-0.77769000	1.48725200
H	-0.27674900	-2.38380300	1.14973700
H	3.23993500	-1.39422600	0.05541300
H	3.39095400	0.94051900	0.45020200
H	1.82006600	2.52983500	0.38660100
H	-0.34676000	2.62960000	-0.11497400
O	-2.31534800	-1.22679200	-0.15246900
H	-2.42237300	-2.09458900	0.26150900
F	-2.24501100	1.31419500	-0.33921400

2-OH-3-CHO-substituted

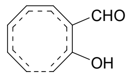

C	1.95954600	0.91946900	0.95528100
C	0.62796200	1.47246500	-0.95157000
C	2.77073600	0.01570000	0.32838600
C	2.34001800	-1.25574500	-0.12388100
C	1.07299900	-1.79649500	-0.26337000
C	-0.28223500	-1.36177400	-0.14655200
C	-1.00850700	-0.18274700	-0.11834900
C	-0.55347900	1.15746300	-0.35147800
H	1.03340800	0.59479100	1.41461900
H	2.34369400	1.88481500	1.27328100
H	1.15257200	0.71221800	-1.51298400
H	0.91361500	2.50811200	-1.11938300
H	3.79863400	0.29428000	0.10404300
H	3.14152800	-1.93584000	-0.40936200
H	1.10825600	-2.85937200	-0.49925400
H	-0.95944800	-2.21469600	-0.09356300
C	-2.47727300	-0.34635100	0.12083400
O	-1.36260600	2.12483700	0.18980400
H	-1.12579000	2.98939700	-0.17489800
H	-3.07472200	0.57774900	0.06750900
O	-3.01618800	-1.41025700	0.34197000

2-OH-3-CN-substituted

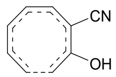

C	1.78434300	1.09603500	0.96110100
C	0.41171900	1.43100200	-0.96174400
C	2.71574600	0.30375000	0.34999300
C	2.46932700	-1.01802900	-0.09511300
C	1.29267700	-1.73190700	-0.24025900
C	-0.11165400	-1.49722100	-0.12634800

C	-0.98342900	-0.41720600	-0.12176100
C	-0.73031300	0.97078500	-0.37884400
H	0.90657400	0.65731900	1.42009200
H	2.03405700	2.10752200	1.26929900
H	1.04398200	0.74190900	-1.50345700
H	0.55105900	2.49227400	-1.15002100
H	3.69779300	0.71874600	0.13149400
H	3.35894100	-1.58188100	-0.37131400
H	1.47664300	-2.78020200	-0.47064800
H	-0.65220800	-2.44019400	-0.05922200
C	-2.36034800	-0.74769300	0.16738100
N	-3.45496000	-1.04813000	0.39666300
O	-1.69390000	1.80058600	0.11407100
H	-1.60932400	2.67963700	-0.28185900

2-OH-3-NO_2-substituted

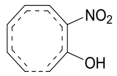

C	2.18815500	1.06420400	0.91243800
C	0.79524000	1.33590800	-1.16339100
C	3.02107600	0.15451300	0.34360700
C	2.64830200	-1.17783600	0.00584000
C	1.41574100	-1.78078700	-0.11960200
C	0.03289600	-1.40381700	-0.07653400
C	-0.72133400	-0.25579200	-0.13302100
C	-0.33752000	1.10885300	-0.45476100
H	1.25513700	0.75854000	1.37035300
H	2.51796000	2.07785900	1.12101800
H	1.32701200	0.51936900	-1.63115200
H	1.07039800	2.35571200	-1.40923200
H	4.03431100	0.45550600	0.08489800
H	3.48313500	-1.84726400	-0.19667300
H	1.49761500	-2.85509300	-0.27374900
H	-0.60153300	-2.28342800	-0.01163300
O	-1.01364100	2.18525200	0.01082300
H	-1.83874000	1.88696100	0.43597000
N	-2.16350200	-0.50163100	0.11951200
O	-2.83542900	0.43517900	0.55501600
O	-2.63294500	-1.59895900	-0.09256900

2-F-3-CH_3-substituted

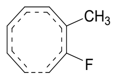

C	-1.44102100	-1.30632300	0.89134200
C	-0.00754900	-1.36197500	-0.93358000
C	-2.45795200	-0.61676400	0.28245900
C	-2.38066300	0.73373600	-0.12935000
C	-1.31100400	1.61222100	-0.21733800
C	0.10853500	1.59285600	-0.05357600
C	1.14136800	0.66704000	-0.02537800
C	1.03596700	-0.70487000	-0.35856300
H	-0.65659300	-0.76900700	1.40892000
H	-1.57273400	-2.34837200	1.16967600

H	-0.74076000	-0.78690200	-1.48005300
H	0.08304500	-2.42157400	-1.14795300
H	-3.36893800	-1.15293100	0.02162900
H	-3.32991300	1.17211900	-0.43394400
H	-1.64383800	2.62570700	-0.44113500
H	0.48546400	2.61094400	0.04652900
F	2.09157900	-1.46619400	0.04673500
C	2.52441400	1.13599900	0.37459700
H	2.82177000	0.69289600	1.33127200
H	3.27782700	0.83869100	-0.36145800
H	2.54789000	2.22265300	0.48222400

2-F-3-NH_2-substituted

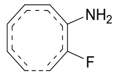

C	1.41186100	-1.35115200	-0.87899200
C	-0.02020000	-1.34106700	0.90987500
C	2.43325200	-0.65494900	-0.27667000
C	2.37219100	0.69303000	0.13627300
C	1.31357700	1.59356600	0.21857900
C	-0.09708600	1.60950000	0.06711400
C	-1.14473800	0.68913900	0.00191900
C	-1.07217700	-0.68011500	0.35456600
H	0.63508300	-0.82029900	-1.41401600
H	1.55413400	-2.39210200	-1.15697400
H	0.71636300	-0.76839700	1.45491000
H	-0.12426900	-2.39610800	1.13938400
H	3.34312400	-1.19523000	-0.01824000
H	3.32442400	1.11652400	0.45067500
H	1.66813300	2.59871400	0.44731400
H	-0.46828900	2.63327200	-0.00276800
F	-2.15798000	-1.41407700	-0.02285700
N	-2.38589700	1.12131000	-0.46912800
H	-2.51233500	2.12435500	-0.49775400
H	-3.19334400	0.62907900	-0.10890400

2-F-3-OH-substituted

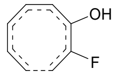

C	-1.37923900	-1.36745300	0.85726200
C	0.06199600	-1.33885600	-0.91863600
C	-2.40855300	-0.68456300	0.25587100
C	-2.36883000	0.67417900	-0.12800400
C	-1.32402400	1.58655800	-0.19538200
C	0.09110600	1.60492200	-0.04801600
C	1.13886600	0.69515600	-0.00982100
C	1.10699500	-0.67944000	-0.34614100
H	-0.61938300	-0.82848900	1.40831700
H	-1.49868600	-2.41611100	1.11585400
H	-0.66360500	-0.76734700	-1.47973000
H	0.16393300	-2.39642000	-1.13683800
H	-3.30399500	-1.23813800	-0.02274800
H	-3.32724100	1.09055100	-0.43299200

H	-1.68430100	2.59256600	-0.40858500
H	0.46150900	2.63033200	0.01956600
F	2.16943200	-1.40102200	0.07265200
O	2.38804700	1.12294000	0.34580400
H	2.33261300	2.01572300	0.71406400

2-F-3-F-substituted

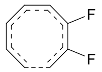

C	1.33747400	-1.38852100	-0.85382000
C	-0.13890900	-1.36852300	0.91744700
C	2.37696000	-0.74567100	-0.22990600
C	2.38238100	0.61785700	0.14565900
C	1.37476600	1.56910200	0.18262400
C	-0.03797300	1.61494800	0.00929600
C	-1.08263500	0.71998400	0.00908200
C	-1.14161800	-0.65187500	0.33963400
H	0.60017200	-0.81928800	-1.40597500
H	1.42295300	-2.43936100	-1.11621200
H	0.60639400	-0.83970800	1.49385000
H	-0.28934000	-2.42458700	1.11388100
H	3.24686900	-1.33050300	0.06429600
H	3.35240900	0.99963300	0.45938700
H	1.76121300	2.56807200	0.37970400
H	-0.41474800	2.62917600	-0.11592700
F	-2.24017000	-1.29939400	-0.10127700
F	-2.28300800	1.23858900	-0.34240100

2-F-3-CHO-substituted

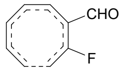

C	1.95207800	0.90553900	0.97730600
C	0.65678100	1.51227800	-0.91659500
C	2.75710100	0.00491900	0.33388900
C	2.32485100	-1.25689300	-0.13766700
C	1.05681300	-1.80047200	-0.27892900
C	-0.29394000	-1.36627400	-0.15694200
C	-1.00518500	-0.17549000	-0.12047900
C	-0.52602700	1.14134200	-0.36706700
H	1.02774500	0.57577400	1.43613700
H	2.34382600	1.86150200	1.31281800
H	1.21888500	0.78374200	-1.48258800
H	0.88132400	2.56503400	-1.05034000
H	3.78586000	0.28320400	0.11300600
H	3.12602900	-1.93092500	-0.43760200
H	1.09361200	-2.86156500	-0.52220800
H	-0.97908100	-2.21295500	-0.10752600
F	-1.32153700	2.13862100	0.10753800
C	-2.47408500	-0.30691900	0.13728000
H	-3.05375000	0.63030700	0.10787500
O	-3.03011800	-1.36123600	0.35472700

2-F-3-CN-substituted

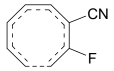

C	1.77579700	1.08895800	0.97344000
C	0.43078100	1.46549200	-0.93807700
C	2.70061700	0.29351600	0.35201500
C	2.45192500	-1.02338600	-0.10022200
C	1.27489600	-1.74124300	-0.24362000
C	-0.12559100	-1.50633600	-0.13172600
C	-0.98504300	-0.41332700	-0.12328400
C	-0.70225800	0.95506400	-0.39502700
H	0.90171800	0.64901800	1.43782100
H	2.03462900	2.09512400	1.29046100
H	1.09144700	0.80816900	-1.48422500
H	0.50987500	2.53512700	-1.09897700
H	3.68338700	0.70666000	0.13300500
H	3.34097100	-1.58421100	-0.38387900
H	1.46112800	-2.78937700	-0.47259700
H	-0.67461800	-2.44446500	-0.06922500
C	-2.36687000	-0.71007900	0.17341500
N	-3.46780700	-0.97340500	0.41603900
F	-1.64437800	1.82064800	0.03709500

2-F-3-NO_2-substituted

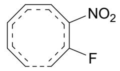

C	2.15968400	0.98592800	0.94145500
C	0.81460600	1.53188300	-0.86462400
C	3.00687900	0.14121900	0.27264700
C	2.64791000	-1.14605800	-0.18612400
C	1.41465700	-1.76571400	-0.30873900
C	0.04000700	-1.41357900	-0.18000500
C	-0.71268300	-0.25966500	-0.13157500
C	-0.34591500	1.10104400	-0.30868000
H	1.28606000	0.59028000	1.44584200
H	2.50008700	1.96330900	1.27133600
H	1.36400600	0.86362100	-1.51296900
H	1.00905500	2.59703300	-0.92871200
H	4.00698900	0.48965000	0.02250000
H	3.48310400	-1.76947000	-0.50127600
H	1.50815900	-2.82009700	-0.56210700
H	-0.59543600	-2.29569800	-0.15324900
F	-1.12374500	2.01186700	0.30767300
N	-2.16477700	-0.49059200	0.07397100
O	-2.49973400	-1.43389600	0.76994500
O	-2.93098500	0.26619300	-0.49174000

2-CHO-3-CH_3-substituted

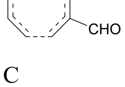

C	1.09881000	-1.59889500	-0.89205500
C	-0.21225500	-1.01921800	1.01517600
C	2.27457900	-1.32759600	-0.25953300
C	2.72837900	-0.02429900	0.09703400

C	2.06401500	1.17944900	0.16807000
C	0.72417800	1.69084600	0.01904100
C	-0.57565100	1.22535300	0.03461200
C	-1.00344000	-0.10936600	0.37052500
H	0.55876000	-0.81127400	-1.40215100
H	0.80825600	-2.61977000	-1.12256700
H	0.68606900	-0.70131700	1.52796300
H	-0.61502900	-2.00186600	1.24404400
H	2.90425200	-2.16045900	0.05069800
H	3.78054000	0.03266100	0.37089800
H	2.74903600	2.00427100	0.36548900
H	0.76645100	2.77184900	-0.11306600
C	-1.65564500	2.23713100	-0.31573800
C	-2.33666600	-0.60008000	-0.07842200
H	-3.03439300	0.14775400	-0.49565900
O	-2.67975100	-1.76265000	-0.01781900
H	-2.07915100	2.05009400	-1.30959600
H	-2.48172600	2.22105100	0.40257100
H	-1.24288200	3.24825400	-0.32834300

2-CHO-3-NH_2-substituted

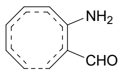

C	1.03948100	-1.68731700	-0.80850900
C	-0.27525700	-0.98157300	1.04686400
C	2.21966900	-1.39262500	-0.19345300
C	2.71407700	-0.08186800	0.06547900
C	2.08792000	1.14657500	0.07501500
C	0.76738400	1.69918900	-0.01105800
C	-0.54459400	1.25682400	0.04101600
C	-1.02107200	-0.06533300	0.36104400
H	0.51348900	-0.92767800	-1.37262300
H	0.73631200	-2.71694800	-0.97491900
H	0.60928200	-0.68083700	1.59447100
H	-0.70649200	-1.95576900	1.25898900
H	2.83293300	-2.21661300	0.17061800
H	3.77366400	-0.03773500	0.31064900
H	2.80735100	1.95589400	0.20005200
H	0.82093100	2.78365300	-0.11491000
C	-2.30801900	-0.53292200	-0.22407200
H	-2.82095400	0.17032500	-0.90331700
O	-2.77118800	-1.63683900	-0.02067100
N	-1.55948600	2.17633500	-0.28374900
H	-1.22720800	3.10712600	-0.50436500
H	-2.33093300	2.21325200	0.37301400

2-CHO-3-OH-substituted

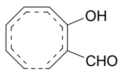

C	1.01623400	-1.66435200	-0.87362900
C	-0.30445200	-1.00650700	0.99796600
C	2.19530900	-1.42571300	-0.23200200
C	2.71095300	-0.14168300	0.10904500
C	2.11344400	1.09921000	0.15189300
C	0.80602100	1.67006800	0.00608900

C	-0.50551900	1.24067000	0.02956200
C	-1.03916200	-0.04883600	0.35825500
H	0.50728000	-0.86886300	-1.40272800
H	0.69922900	-2.67856100	-1.09822400
H	0.60227200	-0.74792000	1.52861000
H	-0.77025200	-1.96497200	1.20872000
H	2.78740300	-2.28025300	0.09377800
H	3.76202100	-0.13477100	0.39117300
H	2.83950500	1.89072000	0.33657800
H	0.87273900	2.75416300	-0.10888300
C	-2.40101100	-0.41868500	-0.12189900
H	-2.98742900	0.37796500	-0.60686500
O	-2.85491300	-1.53815100	-0.00597600
O	-1.49068200	2.14756500	-0.27535100
H	-1.09889900	2.95215100	-0.64322100

2-CHO-3-F-substituted

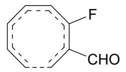

C	1.00983800	-1.65637200	-0.88173600
C	-0.35138300	-1.02983100	0.99408600
C	2.18259800	-1.44018700	-0.22246900
C	2.71614900	-0.16498800	0.12951900
C	2.14296700	1.08568900	0.15744100
C	0.83846000	1.65653100	-0.02035500
C	-0.45434300	1.21229400	0.02655000
C	-1.04830200	-0.04210200	0.35768400
H	0.51502400	-0.84812800	-1.40564700
H	0.68321600	-2.66416800	-1.12061000
H	0.56045400	-0.80624800	1.53108900
H	-0.84878000	-1.97526500	1.19148400
H	2.75820400	-2.30518900	0.10437100
H	3.76339700	-0.17841400	0.42574600
H	2.87313000	1.87194500	0.34524100
H	0.87100100	2.73220000	-0.18597100
C	-2.42726200	-0.34153900	-0.12530300
H	-2.97424800	0.48505800	-0.60783400
O	-2.93599700	-1.43565500	-0.00997600
F	-1.37397300	2.16627700	-0.29895200

2-CHO-3-CHO-substituted

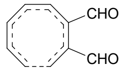

C	1.77125400	-1.19347300	-0.89514600
C	0.39524300	-1.28000600	1.03192400
C	2.76820900	-0.50068200	-0.27278200
C	2.69816100	0.87822000	0.06467700
C	1.62087300	1.73596000	0.14798800
C	0.19131500	1.65509000	0.07549100
C	-0.79715500	0.69005000	0.13333500
C	-0.68115100	-0.72025700	0.40474800
H	0.97803500	-0.65922000	-1.40382800
H	1.88078000	-2.24797500	-1.13135400
H	1.08698900	-0.66397200	1.59261400

H	0.42075300	-2.35493000	1.18737400
H	3.66902300	-1.03325800	0.02773700
H	3.65440100	1.34000200	0.30571600
H	1.93125100	2.76794900	0.30714900
H	-0.24746300	2.64882100	-0.01249400
C	-2.19036500	1.22960700	-0.00160600
C	-1.67100800	-1.66571100	-0.19469800
H	-2.47360300	-1.22524300	-0.81583100
O	-1.60078500	-2.86925600	-0.07513300
H	-3.00425200	0.53202800	0.27411600
O	-2.46523700	2.35963200	-0.33671600

2-CHO-3-CN-substituted

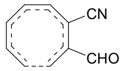

C	1.61552700	-1.30617600	-0.96618500
C	0.24071200	-1.22785100	1.01678200
C	2.68987400	-0.76801300	-0.32479300
C	2.80222600	0.59494100	0.07383500
C	1.85582700	1.58856500	0.19343600
C	0.43038200	1.71669800	0.08567000
C	-0.67244600	0.88085400	0.12710700
C	-0.76177100	-0.52723500	0.41469400
H	0.87768000	-0.66563200	-1.43404400
H	1.59244400	-2.35730400	-1.23840700
H	1.04344700	-0.71745800	1.53169800
H	0.10400800	-2.29067100	1.19602300
H	3.51892500	-1.42003300	-0.05507100
H	3.81121100	0.91193300	0.33189800
H	2.30256200	2.56164000	0.39297300
H	0.14680300	2.76240500	-0.02232400
C	-1.92535700	1.55565700	-0.14584200
N	-2.92481500	2.09985400	-0.36199300
C	-1.95604200	-1.29615700	-0.05239100
H	-2.80998700	-0.70593000	-0.43039200
O	-2.00337300	-2.50570400	-0.03403500

2-CHO-3-NO_2-substituted

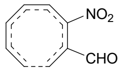

C	-2.07423800	1.10908500	-0.89029400
C	-0.68546100	1.30999800	0.97283600
C	-3.03040900	0.38152200	-0.23871200
C	-2.88912800	-0.98283200	0.12312000
C	-1.77046600	-1.78757000	0.19983100
C	-0.35048200	-1.63847300	0.09969900
C	0.55961200	-0.60752000	0.12962800
C	0.42279300	0.79664000	0.36027800
H	-1.27760300	0.60062000	-1.41970600
H	-2.24111100	2.15116200	-1.14713700
H	-1.32498300	0.67830100	1.57582700
H	-0.76118900	2.38697700	1.09413500
H	-3.94885800	0.87806800	0.06944800
H	-3.81550800	-1.48487800	0.39685600

H	-2.02327600	-2.82964700	0.38714700
H	0.14260700	-2.60404600	0.02156400
C	1.35429900	1.77988800	-0.28871900
H	2.11549600	1.38138800	-0.97869200
O	1.24435200	2.97214000	-0.11776200
N	1.96943200	-1.06324400	-0.04313400
O	2.18703000	-2.09093300	-0.65853900
O	2.83477900	-0.36616400	0.46336300

2-CN-3-CH_3-substituted

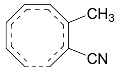

C	1.26352200	-1.55656400	-0.86999700
C	-0.05720600	-1.15430800	1.02987800
C	2.42767000	-1.14111400	-0.28737900
C	2.72006800	0.20243200	0.06931200
C	1.91197600	1.31924400	0.14895500
C	0.52297000	1.65862100	0.00988000
C	-0.71170900	1.03745900	0.04871600
C	-0.96806000	-0.32254700	0.43099500
H	0.62047800	-0.84253900	-1.36910800
H	1.10593900	-2.60394400	-1.11138400
H	0.81252900	-0.72491900	1.50839000
H	-0.33651500	-2.16550500	1.30750500
H	3.17233100	-1.88803100	-0.01578500
H	3.76050900	0.39070100	0.32814600
H	2.49369600	2.22304300	0.33051800
C	-2.24808100	-0.88791900	0.07276400
H	0.42458600	2.73337300	-0.14120100
N	-3.27283200	-1.34460700	-0.21713300
C	-1.92794300	1.86005500	-0.32541100
H	-2.39729900	1.46339300	-1.23312200
H	-2.68769100	1.83340800	0.46279300
H	-1.65798300	2.90111400	-0.51308900

2-CN-3-NH_2-substituted

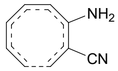

C	1.26716500	-1.61194300	-0.86341000
C	-0.08962400	-1.12129800	1.05739900
C	2.41266200	-1.16521400	-0.27188100
C	2.70294600	0.18616600	0.06555800
C	1.90147900	1.31106400	0.12255800
C	0.52369700	1.66962600	0.00542000
C	-0.72067300	1.05295100	0.03827300
C	-0.99493300	-0.30576800	0.43837800
H	0.59834000	-0.92098200	-1.36084000
H	1.13540500	-2.66593600	-1.09082600
H	0.79141400	-0.69114500	1.51443600
H	-0.36543700	-2.13134800	1.34029000
H	3.16544000	-1.89968700	0.01331500
H	3.74319800	0.37573900	0.32232600
H	2.49416100	2.21090600	0.28868800
C	-2.27225300	-0.85638300	0.06382900

H	0.41640500	2.74882900	-0.10749600
N	-3.31133400	-1.26369700	-0.24972100
N	-1.82667200	1.84402400	-0.25748600
H	-1.68035600	2.58237700	-0.93183400
H	-2.71532700	1.37375800	-0.37435800

2-CN-3-OH-substituted

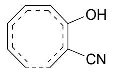

C	1.18160200	-1.62541800	-0.83819500
C	-0.14222100	-1.12010700	1.04015800
C	2.35159500	-1.22610600	-0.25606000
C	2.69778600	0.11496900	0.06324100
C	1.93992600	1.26862100	0.11103800
C	0.56923600	1.65980200	-0.01030600
C	-0.67841500	1.06713300	0.04242700
C	-1.02341100	-0.26694200	0.43077800
H	0.55852500	-0.91177300	-1.36220600
H	1.00099500	-2.67454800	-1.05399700
H	0.73920200	-0.71738100	1.52088500
H	-0.45692900	-2.11962300	1.31998000
H	3.06877600	-1.98988800	0.04225500
H	3.74472500	0.26704900	0.31796000
H	2.55911800	2.15150200	0.26838500
C	-2.31932100	-0.76544500	0.04469300
H	0.48846600	2.74036600	-0.14365900
N	-3.34383900	-1.21263500	-0.25728100
O	-1.77988100	1.80766700	-0.27354900
H	-1.51761800	2.64236700	-0.68688600

2-CN-3-F-substituted

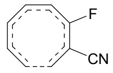

C	1.17066200	-1.61655900	-0.84369700
C	-0.18078900	-1.14631800	1.04030400
C	2.33803200	-1.23551300	-0.24598900
C	2.69949700	0.10158800	0.07913200
C	1.95962200	1.26499500	0.11064200
C	0.58918600	1.65101900	-0.04114100
C	-0.63668800	1.04513500	0.04148000
C	-1.03663200	-0.26630300	0.43220200
H	0.55827900	-0.89055600	-1.36405000
H	0.98049900	-2.66172100	-1.06983100
H	0.70646300	-0.76763300	1.52929500
H	-0.51991700	-2.14135200	1.30725400
H	3.04266400	-2.00909200	0.05590300
H	3.74587400	0.23754900	0.34564000
H	2.58076300	2.14596300	0.26752000
C	-2.34929800	-0.71031800	0.03861200
H	0.47265300	2.71756700	-0.22707600
N	-3.39397400	-1.10222500	-0.26957800
F	-1.68122300	1.83983300	-0.29187600

2-CN-3-CHO-substituted

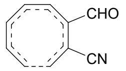

C	-1.74461000	-1.32771000	0.94243500
C	-0.40698000	-1.39391800	-1.02883000
C	-2.78469500	-0.67634300	0.34415200
C	-2.75863400	0.68525200	-0.05885200
C	-1.70911200	1.57310700	-0.19372900
C	-0.28143200	1.54267700	-0.10714000
C	0.74497300	0.61750800	-0.15078300
C	0.67849500	-0.78387900	-0.46168700
H	-0.93755400	-0.76641900	1.39799900
H	-1.83001600	-2.37310600	1.22493300
H	-1.15793800	-0.79040800	-1.52070200
H	-0.39280600	-2.45757900	-1.24313400
H	-3.68918400	-1.23548800	0.11132500
H	-3.73182000	1.10907200	-0.30115800
H	-2.05420300	2.58887700	-0.38315400
C	1.76828700	-1.64259400	-0.05294300
H	0.12576300	2.54865500	-0.00166100
N	2.61752600	-2.35778200	0.27726200
C	2.11224500	1.17985200	0.11059300
H	2.94226400	0.45051000	0.08813800
O	2.33644900	2.34833100	0.32841100

2-CN-3-CN-substituted

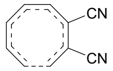

C	1.65206500	-1.36722700	-0.93093800
C	0.32961000	-1.29723700	1.03679800
C	2.73372500	-0.77516400	-0.34369800
C	2.80164000	0.59186400	0.03438200
C	1.81834300	1.55332500	0.15817600
C	0.39138700	1.63067400	0.07569500
C	-0.68981400	0.76527100	0.13879300
C	-0.72256600	-0.63394200	0.46776300
H	0.88518100	-0.76466100	-1.40286500
H	1.66919000	-2.42109200	-1.19349200
H	1.11590000	-0.73259800	1.51915200
H	0.24802600	-2.35331200	1.27130800
H	3.59813600	-1.38952000	-0.09839200
H	3.80167400	0.95179900	0.26947600
H	2.23436700	2.54447700	0.33253100
C	-1.87987200	-1.40364200	0.08104800
H	0.06934100	2.66424500	-0.03854600
C	-1.96900600	1.37927000	-0.13983800
N	-2.98562400	1.88635600	-0.36221800
N	-2.78793200	-2.05185500	-0.22753400

2-CN-3-NO_2-substituted

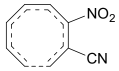

C	2.03944900	1.26023800	0.86449500
C	0.72029300	1.35509200	-1.03911200
C	3.03719100	0.52928400	0.27965000
C	2.92898400	-0.84281800	-0.05956300
C	1.82556400	-1.66762400	-0.15602400
C	0.40193000	-1.54471900	-0.08718300
C	-0.53820700	-0.54404300	-0.16232900
C	-0.41394800	0.84922500	-0.46063400
H	1.22768400	0.75864800	1.37776000
H	2.18622200	2.30951500	1.10427200
H	1.38185500	0.69025800	-1.57952100
H	0.80249600	2.41895900	-1.23444800
H	3.96468800	1.02984600	0.00763600
H	3.87156100	-1.33613800	-0.28988400
H	2.10147000	-2.70897400	-0.31190500
C	-1.36584300	1.80383500	0.05573500
H	-0.07152400	-2.51691900	0.02339300
N	-2.03784700	2.63892300	0.49233100
N	-1.93614300	-1.02050500	0.04241200
O	-2.81835500	-0.36211000	-0.47621900
O	-2.11402000	-2.03350800	0.69488000

2-NO_2-3-CH_3-substituted

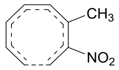

C	-1.42777800	-1.57239000	0.83057600
C	-0.11177100	-0.99099400	-1.06479700
C	-2.59695400	-1.22940700	0.22055200
C	-2.98041700	0.10141300	-0.11303200
C	-2.25563900	1.27250400	-0.15249900
C	-0.89847100	1.71545000	0.03591800
C	0.38999300	1.21292900	0.00020300
C	0.71279800	-0.11589600	-0.43101000
H	-0.83854900	-0.82581700	1.34879800
H	-1.18410100	-2.61023100	1.03801200
H	-0.99530600	-0.60696800	-1.55576900
H	0.23500200	-1.98439500	-1.32561600
H	-3.27653000	-2.02109600	-0.09165300
H	-4.02683200	0.21948000	-0.38884800
H	-2.89719100	2.13542700	-0.33126900
H	-0.89730500	2.78702000	0.23104900
N	2.02468200	-0.68871000	-0.05672700
O	3.02449700	-0.01899100	-0.24275700
O	2.02433400	-1.82260500	0.39586700
C	1.49673600	2.15318400	0.43715000
H	1.06697600	3.10056500	0.76791400
H	2.07077200	1.73506300	1.27023900
H	2.20865100	2.34393700	-0.36901000

2-NO_2-3-NH_2-substituted

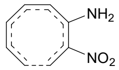

C	1.36442300	-1.66495200	-0.83758100
C	0.07611900	-0.89632700	1.13794600
C	2.52260700	-1.29642800	-0.23300300
C	2.94962500	0.04457700	0.02817900
C	2.26346600	1.23714900	0.05637300
C	0.92089500	1.73699900	-0.02961700
C	-0.38830300	1.28097300	0.04673400
C	-0.74856900	-0.06572200	0.46013200
H	0.74906000	-0.93687900	-1.35296600
H	1.12094600	-2.71104700	-0.99759700
H	0.97095400	-0.49017000	1.59157400
H	-0.23804000	-1.89846000	1.40027200
H	3.19639500	-2.08240500	0.10786100
H	4.01134600	0.14326400	0.24505500
H	2.94507300	2.07939600	0.17548000
H	0.93403300	2.82058300	-0.14842300
N	-2.01438900	-0.67615700	0.01302400
O	-2.97139000	0.04596300	-0.24264800
O	-2.04997800	-1.89002600	-0.07688600
N	-1.38631100	2.20791100	-0.18165500
H	-1.14516000	2.98464000	-0.78026200
H	-2.33035000	1.86370200	-0.27928300

2-NO_2-3-OH-substituted

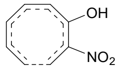

C	1.37071400	-1.64431500	-0.76142800
C	0.04604300	-0.98118800	1.08665100
C	2.54527000	-1.29011900	-0.16437500
C	2.96338700	0.04038900	0.12127500
C	2.27899300	1.23832900	0.11355900
C	0.93916800	1.70754300	-0.06886600
C	-0.34709400	1.20343700	-0.02110700
C	-0.74558000	-0.08529000	0.44308400
H	0.78529800	-0.91945200	-1.31296700
H	1.12713700	-2.68802300	-0.93753400
H	0.93695800	-0.62615200	1.58680900
H	-0.34320100	-1.95659300	1.35770600
H	3.21244200	-2.08194800	0.17401900
H	4.01173000	0.13697300	0.39678900
H	2.94703300	2.08682100	0.25908700
H	0.93040800	2.78177100	-0.26368900
N	-2.08021600	-0.57414300	0.04301800
O	-3.05228800	0.05151700	0.40721100
O	-2.10467300	-1.60953000	-0.60639500
O	-1.38232100	1.98361800	-0.44196700
H	-1.03744400	2.74804000	-0.92489700

2-NO_2-3-F-substituted

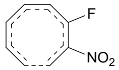

C	1.18819600	-1.64499900	-0.84777300
C	-0.00987500	-1.07754100	1.03257400
C	2.41648000	-1.40072700	-0.29326400
C	2.94481300	-0.12023300	0.01199800
C	2.34779100	1.12271000	0.09433500
C	1.02954300	1.67276800	0.05594800
C	-0.25777000	1.20644000	0.14335400
C	-0.75227700	-0.08192500	0.47135000
H	0.66320000	-0.86124300	-1.38173900
H	0.87846500	-2.66364300	-1.06382700
H	0.89097700	-0.81580600	1.57107900
H	-0.47284500	-2.03642900	1.23726400
H	3.03342700	-2.25313900	-0.01252800
H	4.01143700	-0.10961900	0.22775600
H	3.07850800	1.91869600	0.22836600
H	1.03148800	2.75834700	-0.02490400
N	-2.07831300	-0.46498400	-0.05241800
O	-2.45184200	0.06770600	-1.08056300
O	-2.68938100	-1.32890700	0.55339400
F	-1.20867600	2.14981900	-0.02314700

2-NO_2-3-CHO-substituted

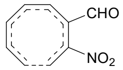

C	-1.65662900	-1.45044000	0.90623200
C	-0.43378700	-1.29654500	-1.02941200
C	-2.81271200	-0.95766100	0.36011400
C	-3.01915000	0.39799900	0.00829800
C	-2.12935700	1.44739600	-0.13842000
C	-0.71391800	1.64721200	-0.13842300
C	0.44156200	0.88957000	-0.21529100
C	0.53949400	-0.50357100	-0.49943200
H	-0.96084900	-0.77643300	1.39344800
H	-1.56996600	-2.50277700	1.16204700
H	-1.24570800	-0.82356200	-1.56553900
H	-0.24371100	-2.34995300	-1.20070800
H	-3.61915500	-1.65151400	0.12882800
H	-4.05535600	0.66604000	-0.19082600
H	-2.64182200	2.39660100	-0.28799700
H	-0.46960500	2.70787400	-0.09187100
N	1.71440300	-1.22549700	0.04574900
O	2.32307500	-0.69255100	0.96045600
O	1.98298200	-2.31848700	-0.42014300
C	1.71955500	1.68577100	-0.14412800
H	2.62968000	1.18352600	-0.51191800
O	1.76460800	2.83232500	0.23307000

2-NO_2-3-CN-substituted

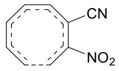

C	1.66290100	-1.41378900	-0.89077300
C	0.42291900	-1.17885800	1.03488300
C	2.81851800	-0.92720100	-0.33870400
C	3.04130500	0.43075200	-0.00649700
C	2.16733400	1.49604300	0.11769000
C	0.75767000	1.72888500	0.09366600
C	-0.41239700	0.98265300	0.15696700
C	-0.54272400	-0.40250100	0.47116200
H	0.98148400	-0.74276700	-1.40148700
H	1.56268300	-2.47039400	-1.12283500
H	1.23833400	-0.69507300	1.55532500
H	0.21736800	-2.22173100	1.24729400
H	3.61156600	-1.62813800	-0.08402600
H	4.07855900	0.68682300	0.20141500
H	2.69308500	2.43818300	0.26344600
H	0.54638700	2.79477700	0.03543400
C	-1.61780700	1.76811500	0.00271200
N	-2.53755500	2.46581000	-0.07719000
N	-1.73599600	-1.12240500	-0.03242800
O	-2.32879200	-0.62865500	-0.97305600
O	-2.02132400	-2.18010900	0.50132100

2-NO_2-3-NO_2-substituted

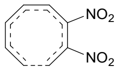

C	2.00552400	1.32479800	0.80037100
C	0.75051700	1.28452700	-1.03093200
C	3.09137400	0.67486400	0.25948500
C	3.13270400	-0.70381900	-0.03558600
C	2.12432100	-1.65121800	-0.12109200
C	0.70033400	-1.67991700	-0.10422000
C	-0.32612500	-0.76509700	-0.18780800
C	-0.30627200	0.61816800	-0.48019300
H	1.26833600	0.76179300	1.36266500
H	2.05921800	2.38763000	1.01982200
H	1.46066100	0.72382400	-1.62433000
H	0.67033600	2.35284000	-1.20155300
H	3.96596100	1.26114800	-0.01617800
H	4.12496900	-1.09889000	-0.24599400
H	2.51821400	-2.65845100	-0.24174900
H	0.31860000	-2.69565600	-0.03840900
N	-1.33552300	1.47508100	0.15697600
O	-1.60211300	2.52940600	-0.38591100
O	-1.78550000	1.09045800	1.22098800
N	-1.68705800	-1.36189700	-0.07614900
O	-1.81775000	-2.38020900	0.57461500
O	-2.57744900	-0.79469700	-0.68221800

5.3.10 2,4-substituted

2-CH_3-4-CH_3-substituted

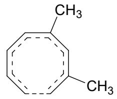

C	0.67745400	1.84386700	-0.81987500
C	1.41269500	0.61297300	0.95737000
C	-0.44437600	2.35948300	-0.21913300
C	-1.57355900	1.59644500	0.15533900
C	-1.79656900	0.22935500	0.20986300
C	-1.07604400	-0.99632800	0.00309200
C	0.27213100	-1.32658200	-0.01283600
C	1.44737800	-0.59645600	0.30946300
H	0.60959300	0.91748100	-1.37644200
H	1.51270200	2.49302000	-1.06971100
H	0.52844400	0.90076600	1.51108400
H	2.33813800	1.11939100	1.22111100
H	-0.44562100	3.40739300	0.07646900
H	-2.43338700	2.18630700	0.47047900
H	-2.84452600	0.01546000	0.42856400
H	0.47192300	-2.34980500	-0.33266000
C	2.75350400	-1.13004100	-0.23150000
H	3.61213200	-0.65011800	0.24558300
H	2.83257400	-2.21076400	-0.07127700
H	2.82736800	-0.95872800	-1.31380800
C	-2.03128500	-2.16965300	-0.19664100
H	-2.72737100	-1.98114100	-1.02085000
H	-1.49760900	-3.09932500	-0.40641200
H	-2.63234300	-2.32831300	0.70702500

2-CH_3-4-NH_2-substituted

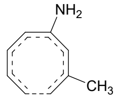

C	0.33744900	1.90299900	-0.83093900
C	1.28563900	0.83244200	0.95982700
C	-0.86147900	2.23048500	-0.24632300
C	-1.83365500	1.28901900	0.16132300
C	-1.81875600	-0.09126600	0.25533100
C	-0.90082800	-1.17192200	0.00600500
C	0.48743400	-1.26537600	-0.04132300
C	1.51566500	-0.35920900	0.31603000
H	0.43420000	0.96685700	-1.36733100
H	1.04678800	2.68340300	-1.09467500
H	0.36694000	0.98014600	1.51260300
H	2.11904800	1.48076800	1.21789600
H	-1.04654800	3.26734700	0.02793300
H	-2.77169000	1.72643000	0.50095500
H	-2.78657800	-0.49475300	0.55360300
H	0.85106800	-2.22508800	-0.41213700
C	2.89565200	-0.66815600	-0.21868400
H	3.66057400	-0.05804700	0.26858800
H	3.14932700	-1.72250200	-0.06373100
H	2.94740600	-0.47873600	-1.29926100
N	-1.59875300	-2.39620700	-0.13094100
H	-2.40742400	-2.35479100	-0.73973300
H	-1.01457500	-3.19167900	-0.35560600

2-CH_3-4-OH-substituted

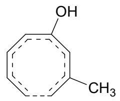

C	-0.14254600	-1.91375500	-0.84267500
C	-1.22279300	-0.92621900	0.95901800
C	1.06549800	-2.13380200	-0.23171500
C	1.95051800	-1.10962800	0.17790000
C	1.80800500	0.26398800	0.24920500
C	0.78261900	1.23737200	0.00137600
C	-0.60043000	1.23624200	-0.02358900
C	-1.55059400	0.23823300	0.31247100
H	-0.31949200	-0.98381500	-1.36892800
H	-0.77805300	-2.75155500	-1.11659500
H	-0.29601100	-0.99763500	1.51346200
H	-1.99489600	-1.65092700	1.20479200
H	1.33610100	-3.14981700	0.04869800
H	2.92142700	-1.45843200	0.52632500
H	2.73584100	0.76017400	0.54035000
H	-1.00802100	2.18437800	-0.37062600
C	-2.94884800	0.43936800	-0.22455800
H	-3.65350900	-0.27049700	0.21608500
H	-3.30464700	1.45337700	-0.01219500
H	-2.97181000	0.31170000	-1.31460600
O	1.28587700	2.51298200	-0.20164700
H	2.19747400	2.45841100	-0.51817200

2-CH_3-4-F-substituted

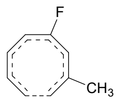

C	0.01483500	-1.93305300	-0.80986800
C	-1.15159600	-0.98728400	0.97047400
C	1.23163500	-2.04482600	-0.19085200
C	2.05719900	-0.94719500	0.15242800
C	1.81065700	0.41164400	0.17797700
C	0.68484300	1.26646100	-0.00329400
C	-0.68910700	1.20447600	-0.00954400
C	-1.56923800	0.13465600	0.30479200
H	-0.22742800	-1.03536900	-1.36569700
H	-0.56423900	-2.82024600	-1.05041200
H	-0.22580200	-0.97375700	1.53141600
H	-1.85999100	-1.77372000	1.21810600
H	1.57261700	-3.02858900	0.12597800
H	3.06811300	-1.20947500	0.45941200
H	2.69611400	1.01714500	0.36305000
H	-1.15187500	2.13910800	-0.32242400
C	-2.97004200	0.22265900	-0.25224300
H	-3.62858900	-0.52291600	0.20047500
H	-3.39900000	1.21436100	-0.07243400
H	-2.97002800	0.06405300	-1.33841600
F	1.13055500	2.55157400	-0.19869700

2-CH_3-4-CHO-substituted

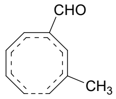

C	1.69357700	1.42628300	-0.83277000
C	1.82610600	-0.03117800	0.95142800
C	0.91227400	2.35928300	-0.20250100
C	-0.44657100	2.17408300	0.15478500
C	-1.24176400	1.04539800	0.20053400
C	-1.09009700	-0.36828600	0.01499300
C	-0.03549500	-1.27351800	-0.00274900
C	1.34225000	-1.13820100	0.29933300
H	1.23196700	0.62431000	-1.39473600
H	2.72907200	1.65031400	-1.07461300
H	1.14867700	0.60772700	1.50293700
H	2.87940500	0.03456300	1.21363200
H	1.37239600	3.29442100	0.11351800
H	-0.96354300	3.08366700	0.45679500
H	-2.28806200	1.27753400	0.39558900
H	-0.32477400	-2.28063100	-0.31115900
C	2.28208300	-2.19332700	-0.23458900
H	3.26225800	-2.13748200	0.24578300
H	1.87681300	-3.19720900	-0.06885900
H	2.42774400	-2.07820600	-1.31651900
C	-2.41182600	-1.06312600	-0.15934800
H	-2.33616800	-2.14631800	-0.38634600
O	-3.49987700	-0.54464300	-0.06384000

2-CH_3-4-CN-substituted

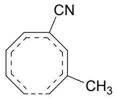

C	-1.87585000	1.07365400	0.87845900
C	-1.71732500	-0.37626900	-0.94736000
C	-1.34575700	2.15708300	0.23165100
C	0.00916500	2.28661100	-0.16405300
C	1.04150500	1.37321000	-0.24108400
C	1.21193900	-0.04713600	-0.06340000
C	0.39663500	-1.17363200	-0.03052000
C	-0.98520100	-1.34065500	-0.30522000
H	-1.23112500	0.38748600	1.41308800
H	-2.92834600	1.05541700	1.14783800
H	-1.21605900	0.40843300	-1.49880600
H	-2.76448300	-0.54754800	-1.18405400
H	-2.01324900	2.96464900	-0.06414200
H	0.29799800	3.28924600	-0.47410500
H	1.99484500	1.84756900	-0.46686800
H	0.91669500	-2.08506800	0.26218100
C	-1.64927500	-2.58186800	0.24198800
H	-2.64598200	-2.72166400	-0.18367000
H	-1.05394900	-3.47315000	0.01719500
H	-1.74898800	-2.52571400	1.33331300

C	2.61756400	-0.38419900	0.09305000
N	3.73889200	-0.64577900	0.21956600

2-CH_3-4-NO_2-substituted

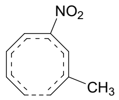

C	2.25641100	-0.99905800	0.87715800
C	2.02807200	0.46130900	-0.93241200
C	1.79660600	-2.09566800	0.19973700
C	0.45764000	-2.29776300	-0.21990400
C	-0.63179800	-1.45341300	-0.29016200
C	-0.87472100	-0.05930000	-0.09778100
C	-0.15043100	1.11386800	-0.04384500
C	1.22487000	1.36397100	-0.28528700
H	1.56916500	-0.36067500	1.41783700
H	3.30314400	-0.92776100	1.15999500
H	1.58866100	-0.34217800	-1.50921400
H	3.06568900	0.70441800	-1.14720800
H	2.51485200	-2.85499800	-0.10527400
H	0.24031500	-3.30647600	-0.56588600
H	-1.54923600	-1.97675600	-0.53829500
H	-0.73598300	1.98102800	0.24729200
C	1.80282400	2.62802600	0.30548600
H	2.79952200	2.83430500	-0.09229300
H	1.16114400	3.48780700	0.08592100
H	1.88043800	2.55241000	1.39741900
N	-2.36025800	0.20285100	0.06478300
O	-2.76835500	1.34137300	-0.08489700
O	-3.07823700	-0.73923800	0.34968100

2- NH_2-4-CH_3-substituted

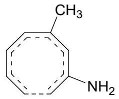

C	0.82274900	1.80020600	-0.83804400
C	1.50590600	0.46699400	0.97265700
C	-0.24839300	2.39112400	-0.22698100
C	-1.43050300	1.70954300	0.16119200
C	-1.75608100	0.36892300	0.22981200
C	-1.13299200	-0.91393400	0.00810000
C	0.17369700	-1.35618500	-0.01480800
C	1.41561800	-0.71315100	0.28533700
H	0.70601100	0.86716300	-1.37474100
H	1.70950300	2.37994000	-1.08056600
H	0.65685200	0.80728200	1.54883000
H	2.47511500	0.90583300	1.19529400
H	-0.17316300	3.43568400	0.07023700
H	-2.24117700	2.36361600	0.48114900
H	-2.81079900	0.23304300	0.47492300
H	0.28996100	-2.39721300	-0.31832000
C	-2.18383100	-2.00158000	-0.19309100
H	-2.84129100	-1.76182700	-1.03557800

H	-1.73338700	-2.97972700	-0.37627600
H	-2.81642800	-2.08688400	0.69854300
N	2.56269700	-1.26768200	-0.30738100
H	3.40414400	-1.20797600	0.25281500
H	2.43875200	-2.19679800	-0.68967700

2- NH_2-4-NH_2-substituted

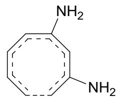

C	-0.41114500	-1.90314100	-0.85111700
C	-1.36674000	-0.75647900	0.97669200
C	0.76568300	-2.26647000	-0.25618700
C	1.76714800	-1.35547500	0.16898800
C	1.80126500	0.01840100	0.27693200
C	0.92641400	1.13863500	0.00786300
C	-0.44748400	1.29538000	-0.04997300
C	-1.52017900	0.42017500	0.29166400
H	-0.50100800	-0.95638300	-1.36909500
H	-1.15338000	-2.65582600	-1.10477300
H	-0.46775300	-0.92317000	1.55328200
H	-2.22767000	-1.38311900	1.19290900
H	0.91568700	-3.30840400	0.01979900
H	2.68553600	-1.82822900	0.51589500
H	2.77232900	0.38664500	0.60709600
H	-0.77077900	2.27303200	-0.41087800
N	1.68227600	2.32700700	-0.12675800
H	2.50656600	2.23544700	-0.70850900
H	1.14435300	3.14580900	-0.38149500
N	-2.76519200	0.72003200	-0.29054400
H	-3.56569800	0.48157200	0.28191500
H	-2.84754600	1.65719800	-0.66420200

2- NH_2-4-OH-substituted

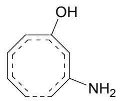

C	0.15499200	1.92368400	-0.86252000
C	1.29412300	0.90378500	0.95736300
C	-1.03850900	2.15291300	-0.23649600
C	-1.92502500	1.13092600	0.19025500
C	-1.79260500	-0.23889800	0.26752600
C	-0.77714100	-1.22767400	0.00056900
C	0.59818200	-1.25755200	-0.01971600
C	1.57063100	-0.26213600	0.29307000
H	0.33130000	0.98717300	-1.37739900
H	0.80002000	2.75380000	-1.13795700
H	0.38824700	0.98103000	1.54282700
H	2.08503800	1.61859100	1.16996600
H	-1.29858700	3.17010700	0.04853100
H	-2.88868500	1.48775500	0.55119000
H	-2.71322100	-0.72796300	0.59096600
H	1.00060700	-2.21996000	-0.32949200

O	-1.30725500	-2.49032400	-0.20734000
H	-2.22538600	-2.41298200	-0.49922600
N	2.86222600	-0.53544500	-0.18090100
H	3.52641200	0.21931100	-0.06475900
H	2.90883600	-0.93645200	-1.10992500

2- NH_2-4-F-substituted

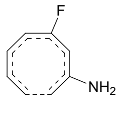

C	0.01675200	-1.95530400	-0.82535600
C	-1.21582600	-0.96637400	1.00000800
C	1.22267600	-2.05402700	-0.19704900
C	2.04349200	-0.94682700	0.15347300
C	1.79639200	0.40612400	0.19324100
C	0.66749500	1.26507000	-0.00214700
C	-0.69896300	1.21391000	-0.01484000
C	-1.58957000	0.13427500	0.28293600
H	-0.25995500	-1.05598600	-1.36194900
H	-0.56540200	-2.84441700	-1.05109500
H	-0.30541500	-0.94268300	1.58255900
H	-1.92735400	-1.76072300	1.20823300
H	1.57082000	-3.03364900	0.12437900
H	3.05542000	-1.20777700	0.45942600
H	2.67463100	1.01269500	0.40499300
H	-1.15905100	2.15524500	-0.31033500
F	1.12145900	2.54763700	-0.19021300
N	-2.84678400	0.18521600	-0.33639100
H	-3.60695100	-0.21075900	0.20227600
H	-3.09707700	1.08172800	-0.73343800

2- NH_2-4-CHO-substituted

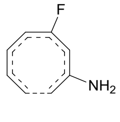

C	-1.73830000	1.38708900	0.84785900
C	-1.86900500	-0.13366400	-0.94142800
C	-0.99408000	2.33436700	0.20143500
C	0.36815300	2.18065700	-0.16756100
C	1.19502400	1.07782400	-0.21190200
C	1.08790100	-0.34175500	-0.01660900
C	0.07705200	-1.28727400	-0.00327300
C	-1.31777600	-1.19726400	-0.27480500
H	-1.25017000	0.59570000	1.40277800
H	-2.77941400	1.57723100	1.09487200
H	-1.22742500	0.51360500	-1.52342300
H	-2.93127800	-0.11545400	-1.17330900
H	-1.48273900	3.25296400	-0.11909100
H	0.85778000	3.10203900	-0.48026200
H	2.23147400	1.33775400	-0.42148900
H	0.39773300	-2.29534400	0.26540900
C	2.43530000	-0.99300500	0.15234400
H	2.39798200	-2.08074400	0.36609300

O	3.50310000	-0.43390500	0.06773100
N	-2.10024300	-2.26554000	0.19287900
H	-3.09498300	-2.16049800	0.03651700
H	-1.90767200	-2.55907900	1.14354300

2-NH_2-4-CN-substituted

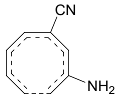

C	1.93175400	-1.01042000	0.90251700
C	1.71180900	0.51255800	-0.97541500
C	1.44591700	-2.09811500	0.24136200
C	0.09555400	-2.27238600	-0.17364000
C	-0.96820900	-1.40446300	-0.26527300
C	-1.20101700	0.00994800	-0.06582900
C	-0.44853900	1.16920900	-0.02306800
C	0.93789000	1.40071500	-0.28371700
H	1.27108800	-0.31918700	1.41051600
H	2.98351700	-0.94911100	1.16841900
H	1.23940100	-0.27949200	-1.53929000
H	2.75627600	0.72767800	-1.18427200
H	2.14075900	-2.88295200	-0.05216700
H	-0.15129100	-3.28472200	-0.48957400
H	-1.89694800	-1.91032500	-0.52154300
H	-1.01222600	2.05750400	0.25879900
C	-2.62147000	0.28023700	0.09114600
N	-3.75346200	0.48904200	0.21961400
N	1.50857400	2.52968200	0.32078000
H	2.23251000	2.98698000	-0.21945300
H	0.84899200	3.19886500	0.69731500

2-NH_2-4-NO_2-substituted

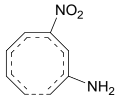

C	2.27474200	-0.96709100	0.90191900
C	2.04854000	0.56663100	-0.92260900
C	1.84856500	-2.06143000	0.20709200
C	0.51695400	-2.27774400	-0.23880700
C	-0.58518800	-1.45539300	-0.31619400
C	-0.86009400	-0.06617800	-0.10282700
C	-0.17992500	1.12551600	-0.05254100
C	1.20088900	1.41463900	-0.26272200
H	1.56784400	-0.33385800	1.42342900
H	3.31561400	-0.87257000	1.19940100
H	1.64003400	-0.23217000	-1.52593900
H	3.08245800	0.84774900	-1.10667500
H	2.58290500	-2.80641800	-0.09372400
H	0.32200000	-3.28559400	-0.60104500
H	-1.48760400	-1.98968400	-0.59324900
H	-0.78962300	1.98736300	0.20120800
N	-2.35581600	0.15487900	0.06458900
O	-2.79748300	1.28161600	-0.07411900

O	-3.04241700	-0.80993100	0.34806700
N	1.63840800	2.63832200	0.25912300
H	2.63283600	2.80859800	0.18054000
H	1.28768200	2.87699800	1.17861800

2-OH-4-CH_3-substituted

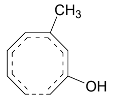

C	0.98841400	1.70073900	-0.84628200
C	1.57843900	0.33185000	0.89876200
C	-0.01128400	2.39715900	-0.21369900
C	-1.23939300	1.83305400	0.20036300
C	-1.69965700	0.52684400	0.25293000
C	-1.21479200	-0.80274300	0.00089200
C	0.04563900	-1.37200600	-0.04757300
C	1.32841700	-0.85564800	0.26268600
H	0.75364000	0.80254300	-1.40364300
H	1.91397100	2.20168100	-1.11860300
H	0.79326000	0.77359100	1.49549100
H	2.59865100	0.63651100	1.12190500
H	0.17397300	3.42835500	0.08157400
H	-1.97117800	2.55854000	0.55414400
H	-2.75945600	0.49612000	0.51163900
H	0.08534500	-2.40824000	-0.37636000
C	-2.36570000	-1.78316100	-0.19984900
H	-3.01762400	-1.46320800	-1.01952000
H	-2.00959300	-2.79213700	-0.41793200
H	-2.98203000	-1.83398200	0.70566300
O	2.34665500	-1.61333200	-0.26480000
H	3.18730300	-1.34964200	0.13465900

2-OH-4-NH_2-substituted

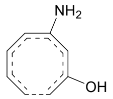

C	0.56535800	1.85471900	-0.85138300
C	1.45545600	0.66276600	0.90872100
C	-0.57305700	2.31635500	-0.23902400
C	-1.63440800	1.49011900	0.19872700
C	-1.78476400	0.11908500	0.29111900
C	-1.01348200	-1.06468600	0.00254300
C	0.34640200	-1.33213800	-0.07496100
C	1.47203700	-0.55097100	0.27023200
H	0.54902600	0.91683800	-1.39310600
H	1.35337100	2.55006800	-1.12993100
H	0.59495800	0.92233400	1.50895100
H	2.38463800	1.18518600	1.12374800
H	-0.63099700	3.36637500	0.04039600
H	-2.50385900	2.03394600	0.56675800
H	-2.78244400	-0.16740800	0.62312700
H	0.61419500	-2.31794900	-0.45063800
N	-1.85592800	-2.18884100	-0.13144300

H	-2.68691200	-2.03405100	-0.68902100
H	-1.38601300	-3.04514700	-0.39607800
O	2.63642100	-1.05685400	-0.25766300
H	3.39291100	-0.60495800	0.14135700

2-OH-4-OH-substituted

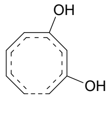

C	0.27262400	1.90543100	-0.86352700
C	1.35822700	0.83957300	0.91090900
C	-0.90439400	2.20120100	-0.22617500
C	-1.83680700	1.23308700	0.21649500
C	-1.78634300	-0.14597100	0.28765800
C	-0.84094500	-1.18895600	-0.00463100
C	0.53320100	-1.29063700	-0.06059100
C	1.54343800	-0.35566500	0.26834300
H	0.38632300	0.96737800	-1.39292600
H	0.95259600	2.70249300	-1.15269300
H	0.46729900	0.97988400	1.50638100
H	2.20229800	1.49213900	1.12102700
H	-1.10628500	3.23227400	0.05627300
H	-2.77084000	1.64381900	0.59736100
H	-2.73136700	-0.57829600	0.62104700
H	0.89647400	-2.25049700	-0.41678100
O	-1.43974700	-2.41824400	-0.20739600
H	-2.35869400	-2.29303700	-0.47988100
O	2.76163900	-0.69869100	-0.26291900
H	3.45305000	-0.14905100	0.13182900

2-OH-4-F-substituted

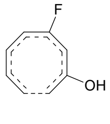

C	0.04173400	1.94040100	-0.83062900
C	1.26048200	0.96176800	0.92223200
C	-1.15604400	2.08311900	-0.18332900
C	-1.99635700	1.00611100	0.19085500
C	-1.79094400	-0.35886900	0.21539600
C	-0.69924200	-1.24938100	-0.00849300
C	0.67200400	-1.23682000	-0.04621900
C	1.57894400	-0.19072500	0.25876700
H	0.25770000	1.03764200	-1.38921200
H	0.63493900	2.81406800	-1.08636300
H	0.36366800	0.98724500	1.52491000
H	2.01964300	1.71184800	1.13009100
H	-1.46734800	3.07574700	0.13554500
H	-2.98956700	1.29676700	0.52913800
H	-2.68606200	-0.93686100	0.43635000
H	1.12535500	-2.17025500	-0.36813400
F	-1.19008300	-2.51537800	-0.19719800
O	2.81707300	-0.39185800	-0.29352700
H	3.45237000	0.22344100	0.09919300

2-OH-4-CHO-substituted

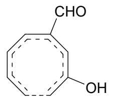

C	-1.78986300	1.32531400	0.87017500
C	-1.89211500	-0.21284800	-0.93890100
C	-1.08510500	2.28581800	0.20137900
C	0.27741300	2.17829500	-0.18513900
C	1.14709500	1.10847100	-0.22820900
C	1.09393800	-0.30968900	-0.01570200
C	0.11064600	-1.28452200	0.01464500
C	-1.27798000	-1.23322100	-0.27281800
H	-1.27341900	0.54690700	1.41687500
H	-2.83447300	1.48075400	1.12479800
H	-1.29008000	0.47733900	-1.51160400
H	-2.95490100	-0.27422900	-1.15033600
H	-1.60844300	3.18447400	-0.12090200
H	0.72704800	3.11454000	-0.51306400
H	2.17112700	1.40573900	-0.44900400
H	0.46668200	-2.27747500	0.30015900
C	2.46206100	-0.91331800	0.15846600
H	2.46197400	-1.99886600	0.38798100
O	3.50941800	-0.31960400	0.06350900
O	-2.07580600	-2.25069100	0.18968100
H	-1.61095600	-2.76262400	0.86619100

2-OH-4-CN-substituted

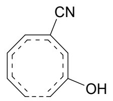

C	2.01196200	0.99228300	-0.88021400
C	1.77173200	-0.60301600	0.87681300
C	1.50194700	2.07481200	-0.20675700
C	0.16127700	2.25886900	0.20799900
C	-0.94017200	1.41985300	0.27204900
C	-1.21868300	0.02429800	0.05608900
C	-0.48179400	-1.15849800	-0.01500800
C	0.88162200	-1.44022400	0.24545400
H	1.37025300	0.33380900	-1.44651300
H	3.06625800	0.96249800	-1.13884700
H	1.39757100	0.20383500	1.48574300
H	2.78978500	-0.92973000	1.07195100
H	2.19768300	2.85310900	0.10302700
H	-0.05805000	3.26697000	0.55665500
H	-1.85135600	1.95690600	0.52655000
H	-1.04408100	-2.03488000	-0.32454400
C	-2.64109400	-0.20929700	-0.09119200
N	-3.78508300	-0.38540000	-0.21220200
O	1.27686500	-2.64721800	-0.27976200
H	2.13181400	-2.89145200	0.09808700

2-OH-4-NO_2-substituted

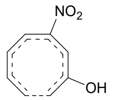

C	-2.37630200	0.91855000	0.91209400
C	-2.07986900	-0.60327000	-0.90610500
C	-1.89609500	2.03469000	0.27239200
C	-0.56368500	2.27063100	-0.14357600
C	0.56401300	1.47040800	-0.24491800
C	0.86802200	0.09219100	-0.07074900
C	0.19228600	-1.11286000	-0.03257700
C	-1.16072500	-1.42939600	-0.30227100
H	-1.71798300	0.25599700	1.45368400
H	-3.42886100	0.85434400	1.17175700
H	-1.73507200	0.23832200	-1.48459100
H	-3.08503900	-0.95976000	-1.11545700
H	-2.61397200	2.80403700	-0.00819700
H	-0.37955600	3.29451000	-0.46511700
H	1.46018200	2.02637400	-0.49636900
H	0.79625000	-1.96329100	0.26087400
N	2.37828900	-0.12412900	0.06857300
O	2.77641500	-1.21952600	0.45742800
O	3.13105600	0.80740300	-0.20860000
O	-1.51530400	-2.66368400	0.18703900
H	-2.36717500	-2.92083400	-0.18927000

2-F-4-CH_3-substituted

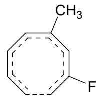

C	1.26388700	1.51914200	-0.85518200
C	1.65745100	0.07692200	0.88400000
C	0.38761500	2.35343300	-0.20495800
C	-0.91402800	1.99482800	0.21037300
C	-1.58918000	0.78358100	0.25315600
C	-1.33078500	-0.60315900	-0.00130700
C	-0.16973700	-1.35901600	-0.05534300
C	1.16016800	-1.03549200	0.27223000
H	0.88922000	0.67455100	-1.41953200
H	2.25348500	1.87385800	-1.13060100
H	0.99258800	0.66980000	1.49460700
H	2.72443800	0.14811500	1.06786000
H	0.73635600	3.33851800	0.09997500
H	-1.51374300	2.82982000	0.57083800
H	-2.64049300	0.93059000	0.50663200
H	-0.28176000	-2.38765700	-0.39051500
C	-2.61994800	-1.39170800	-0.19954400
H	-3.23145100	-0.95541500	-0.99593600
H	-2.42374800	-2.43639200	-0.44995900
H	-3.21895300	-1.37572500	0.71873900
F	2.07126800	-1.92680500	-0.21029500

2-F-4-NH_2-substituted

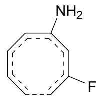

C	0.83716900	1.75718700	-0.85376400
C	1.57495200	0.46854900	0.89606100
C	-0.22620400	2.36298200	-0.22945600
C	-1.39395000	1.69460300	0.20208900
C	-1.74657700	0.35864400	0.28455600
C	-1.15366000	-0.92018300	0.00058000
C	0.16267300	-1.36383300	-0.08067200
C	1.36176900	-0.73134700	0.28157400
H	0.68643400	0.83677500	-1.40416300
H	1.71053900	2.34091700	-1.13191600
H	0.78873600	0.88167700	1.51113100
H	2.59372300	0.79508300	1.07473200
H	-0.13674000	3.40833200	0.05918200
H	-2.17460300	2.35687900	0.57449600
H	-2.77835700	0.22192500	0.60785300
H	0.30573500	-2.37319000	-0.46082400
N	-2.13515700	-1.92144400	-0.12876200
H	-2.94678200	-1.66211700	-0.67550000
H	-1.78844000	-2.83740100	-0.38249600
F	2.46542500	-1.36426500	-0.20855200

2-F-4-OH-substituted

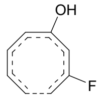

C	0.41089000	1.88785900	-0.86990100
C	1.44832900	0.77423800	0.89135100
C	-0.73942200	2.24959800	-0.21565700
C	-1.73316700	1.34597600	0.22545500
C	-1.78501400	-0.03542000	0.28436000
C	-0.91679200	-1.13817200	-0.00697400
C	0.45227400	-1.31924100	-0.06620600
C	1.49485000	-0.44126500	0.27708600
H	0.45774700	0.95098500	-1.41097600
H	1.13548900	2.64378900	-1.16023800
H	0.58902200	1.01691200	1.49891000
H	2.37250600	1.31487400	1.06485000
H	-0.86981000	3.28901900	0.07829300
H	-2.63341900	1.81952900	0.61444700
H	-2.76143300	-0.39924900	0.60869100
H	0.76658400	-2.29642000	-0.42123400
O	-1.58590400	-2.32872200	-0.20592800
H	-2.49874500	-2.15267500	-0.47161300
F	2.70417800	-0.83315900	-0.20786600

2-F-4-F-substituted

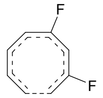

C	-0.09283500	-1.93936000	-0.84214300
C	-1.30793400	-0.96437700	0.90370800
C	1.09429300	-2.10201300	-0.17736600
C	1.95527500	-1.04597200	0.20377300
C	1.78726100	0.32606800	0.21986500
C	0.72177200	1.24026800	-0.01083900
C	-0.65223300	1.24627000	-0.05582500
C	-1.55271700	0.21320900	0.26773300
H	-0.28170800	-1.03421000	-1.40634500
H	-0.69785200	-2.80229300	-1.10522500
H	-0.41988700	-1.04712100	1.51346100
H	-2.12382100	-1.65667200	1.08088500
H	1.38039500	-3.10061300	0.14662100
H	2.93606300	-1.36147800	0.55531100
H	2.69720100	0.88071100	0.44044000
H	-1.09785300	2.18129400	-0.38320700
F	1.23271600	2.49573700	-0.19904200
F	-2.80047500	0.40379900	-0.23377900

2-F-4-CHO-substituted

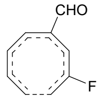

C	-1.81754300	1.27225600	0.87375300
C	-1.92179700	-0.24492000	-0.88456800
C	-1.13387200	2.24723700	0.19432100
C	0.22009500	2.16580100	-0.21330700
C	1.12002000	1.11608700	-0.24764100
C	1.10508100	-0.29342700	-0.00698900
C	0.13701800	-1.28598600	0.05024400
C	-1.23122800	-1.24416300	-0.26574900
H	-1.27986900	0.51811700	1.43392200
H	-2.85759700	1.42341500	1.14941800
H	-1.37027100	0.46802600	-1.47922500
H	-2.98252300	-0.36812300	-1.07731500
H	-1.67862600	3.13554300	-0.12128400
H	0.64005700	3.10642600	-0.56626700
H	2.13418100	1.43801500	-0.48072800
H	0.47617300	-2.26724900	0.38113600
C	2.48557100	-0.86559800	0.17667300
H	2.50637400	-1.94107200	0.44542800
O	3.51878900	-0.25373600	0.04937200
F	-1.94647600	-2.29854700	0.20661300

2-F-4-CN-substituted

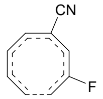

C	1.97110600	-0.90135100	0.91710000
C	1.76965100	0.60761900	-0.87823700
C	1.54413200	-2.00136400	0.22293000
C	0.21845000	-2.22933300	-0.22164300
C	-0.89782400	-1.41758600	-0.29243400
C	-1.20580500	-0.03205400	-0.06028200
C	-0.49511200	1.15810600	0.01496800
C	0.85888700	1.41572300	-0.27015800
H	1.26472800	-0.28003800	1.45275700
H	3.01137400	-0.81211300	1.21743000
H	1.41585300	-0.21822000	-1.47734600
H	2.77916000	0.97019700	-1.04157600
H	2.28216900	-2.74414800	-0.07476900
H	0.03272800	-3.23986500	-0.58141800
H	-1.79526200	-1.97120200	-0.56100500
H	-1.05744800	2.03355200	0.32955000
C	-2.63635800	0.17075600	0.09642400
N	-3.77738300	0.32093300	0.22439700
F	1.30507000	2.59913400	0.22151100

2-F-4-NO_2-substituted

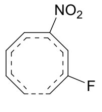

C	-2.30586000	0.82912500	0.96255600
C	-2.08109800	-0.58191900	-0.91663000
C	-1.90759800	1.96895700	0.31727900
C	-0.59220700	2.24563900	-0.13015100
C	0.54253300	1.46537100	-0.25210000
C	0.86073500	0.08957200	-0.07516900
C	0.19991700	-1.11852800	-0.04982500
C	-1.14679200	-1.39371800	-0.35115600
H	-1.58185800	0.19430500	1.45688300
H	-3.34071400	0.70486800	1.26901300
H	-1.75486400	0.28367000	-1.47379800
H	-3.08035600	-0.96537300	-1.09511800
H	-2.66332200	2.71067900	0.06464700
H	-0.43370000	3.27446500	-0.44811200
H	1.42592000	2.03576700	-0.51887300
H	0.78898300	-1.98062200	0.24497200
N	2.36041500	-0.11145700	0.07727300
O	2.74213500	-1.11806100	0.64339800
O	3.10282900	0.74113500	-0.37116700
F	-1.56227600	-2.60990500	0.08364400

2-CHO-4-CH_3-substituted

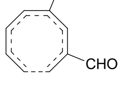

C	0.19258700	1.90079300	-0.90138400
C	1.10664600	0.77249200	0.96323100
C	-0.92719900	2.30543600	-0.23331900

C	-1.98526600	1.45525800	0.19365400
C	-2.10322700	0.08323300	0.25668900
C	-1.28911900	-1.08176700	0.00450700
C	0.07529400	-1.28748600	0.00706700
C	1.17638100	-0.44552400	0.34029400
H	0.21154600	0.94408200	-1.40753000
H	0.95964200	2.61632300	-1.18369300
H	0.22551800	1.05413600	1.52243900
H	2.02747400	1.31919200	1.14991500
H	-1.00374700	3.34984900	0.06549600
H	-2.87198700	1.98461800	0.53939200
H	-3.12014500	-0.21604900	0.51769500
H	0.37705200	-2.28828000	-0.30504600
C	-2.14668000	-2.31500300	-0.24964900
H	-2.84989900	-2.14702200	-1.07210100
H	-1.53938300	-3.18948900	-0.49322300
H	-2.73936800	-2.55739100	0.64073600
C	2.51011900	-0.90405100	-0.13179900
H	2.54330700	-1.94398800	-0.51919800
O	3.51534500	-0.22828300	-0.11882800

2-CHO-4-NH_2-substituted

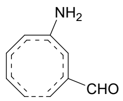

C	0.08386600	1.88152100	-0.89991000
C	1.06555100	0.82016300	0.95384700
C	-1.06643300	2.24368900	-0.25616900
C	-2.06885000	1.34360800	0.19639400
C	-2.11429900	-0.02982000	0.29212200
C	-1.24440100	-1.14676300	0.00410600
C	0.13450200	-1.28301000	-0.02477300
C	1.18579700	-0.40004200	0.33847900
H	0.15178700	0.91978300	-1.39269900
H	0.81178100	2.63188900	-1.19534700
H	0.17745800	1.06784000	1.51804800
H	1.96552800	1.40092200	1.13712600
H	-1.20254400	3.28759300	0.02071300
H	-2.97217800	1.82746900	0.56486600
H	-3.08717600	-0.39597000	0.62007100
H	0.47805000	-2.25621400	-0.37999800
N	-1.99408100	-2.33057600	-0.17239800
H	-2.80017800	-2.23677800	-0.77872200
H	-1.44856200	-3.14844100	-0.41360900
C	2.54099800	-0.80064100	-0.13069900
H	2.61585600	-1.83319400	-0.53285200
O	3.52104500	-0.09038600	-0.10014900

2-CHO-4-OH-substituted

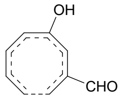

C	0.00460700	1.87865500	-0.90440000

C	1.05178300	0.83078100	0.96203300
C	-1.14362400	2.20234700	-0.24028100
C	-2.12010600	1.27076500	0.20800600
C	-2.12054800	-0.10437000	0.27970800
C	-1.19898400	-1.17370800	-0.00260900
C	0.17296700	-1.29608800	-0.00769000
C	1.20254800	-0.38024300	0.34019400
H	0.10254800	0.91629600	-1.39082600
H	0.70796800	2.65017000	-1.20399700
H	0.15648100	1.05812700	1.52359500
H	1.93392600	1.44062800	1.13820900
H	-1.30855200	3.24025800	0.04284100
H	-3.03802900	1.72205500	0.58087400
H	-3.08346600	-0.50801300	0.59809600
H	0.50525600	-2.27753200	-0.34396600
O	-1.82619000	-2.38276500	-0.24281700
H	-2.72203400	-2.23084800	-0.57273200
C	2.56718400	-0.74750900	-0.13437300
H	2.66844300	-1.77982400	-0.52863100
O	3.52400300	-0.00662300	-0.11305800

2-CHO-4-F-substituted

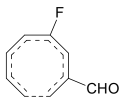

C	-0.04329500	1.89905000	-0.87379800
C	1.03093000	0.81954500	0.98957700
C	-1.19370800	2.17639500	-0.19761500
C	-2.17705900	1.21613600	0.17784400
C	-2.15010500	-0.15766600	0.20467800
C	-1.16658900	-1.17612700	-0.00668000
C	0.19489300	-1.31040700	0.01261800
C	1.21347400	-0.37030200	0.34190900
H	0.07592500	0.95501000	-1.39124000
H	0.65525300	2.68920600	-1.13268000
H	0.12765800	1.01221300	1.55235500
H	1.89109900	1.45942600	1.16767600
H	-1.37411500	3.19956400	0.12740000
H	-3.12576800	1.63934600	0.50272300
H	-3.11420300	-0.62033300	0.40860400
H	0.53074600	-2.30141200	-0.29003500
F	-1.80206000	-2.36531100	-0.24983700
C	2.58050800	-0.69694300	-0.15283700
H	2.70603100	-1.72375600	-0.55419800
O	3.51395200	0.07255600	-0.13953100

2-CHO-4-CHO-substituted

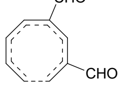

C	0.63267200	2.15321200	-0.90968200
C	1.42641300	1.03896700	0.96041100
C	-0.48082000	2.64709200	-0.28611000

C	-1.60904800	1.87969200	0.11210300
C	-1.80727400	0.51937700	0.20643400
C	-1.02667200	-0.67749900	0.05188400
C	0.31890600	-0.99636900	0.08919400
C	1.46083500	-0.21916900	0.40552000
H	0.59410000	1.19391500	-1.41022400
H	1.44322900	2.81792100	-1.19517200
H	0.55130600	1.37349000	1.49996000
H	2.35854000	1.55959800	1.17430100
H	-0.49776700	3.70194100	-0.01549500
H	-2.47899100	2.47048700	0.39336300
H	-2.84498500	0.25632100	0.40913800
H	0.56223400	-2.02738300	-0.17122300
C	-1.88410400	-1.90017100	-0.13467900
H	-1.32024300	-2.83182600	-0.33918700
O	-3.09042400	-1.92195700	-0.06797400
C	2.78980700	-0.77573300	0.04443500
H	3.64608800	-0.09502400	0.23388200
O	2.97319900	-1.88252300	-0.40907700

2-CHO-4-CN-substituted

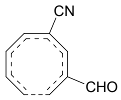

C	0.93431100	1.84691300	-0.97338200
C	1.49573500	0.48800200	0.96436700
C	0.04358500	2.58757600	-0.25906100
C	-1.23368200	2.14367200	0.20086000
C	-1.79551500	0.89516800	0.30004200
C	-1.36412200	-0.47092700	0.08211200
C	-0.15400300	-1.13794300	0.06738000
C	1.16645800	-0.68925400	0.35168000
H	0.63288300	0.92072400	-1.44662500
H	1.88450400	2.26867600	-1.28780400
H	0.75721600	1.05407400	1.51471200
H	2.54531400	0.72380400	1.12111400
H	0.32595300	3.59898700	0.02872700
H	-1.89180800	2.94298200	0.53643900
H	-2.84836800	0.92761900	0.57482900
H	-0.22444900	-2.18750700	-0.21792300
C	-2.49912100	-1.35412800	-0.12194800
N	-3.41039800	-2.04937400	-0.28733500
C	2.27541700	-1.56023600	-0.13275300
H	1.97007600	-2.56339400	-0.49427200
O	3.43838500	-1.22917500	-0.14920500

2-CHO-4-NO_2-substituted

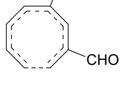

C	1.53142600	1.74861700	-1.02382900
C	1.85438100	0.38764100	0.97823900

C	0.76633600	2.64417000	-0.34255100
C	-0.56705500	2.42863000	0.12268500
C	-1.32671800	1.29450100	0.26883800
C	-1.10346600	-0.11204800	0.09562800
C	-0.03911600	-0.97515600	0.11500800
C	1.33471600	-0.73768600	0.40251000
H	1.09318200	0.86195500	-1.46411600
H	2.53746900	2.00552200	-1.34214600
H	1.22229400	1.09168300	1.50146100
H	2.92861800	0.44804400	1.13422400
H	1.20481200	3.60807200	-0.08874200
H	-1.08626500	3.33595300	0.42516500
H	-2.35682900	1.49439700	0.54582600
H	-0.29016900	-1.99949800	-0.15038000
N	-2.41380400	-0.85273800	-0.08972000
O	-2.38613300	-1.92039300	-0.67319500
O	-3.42597300	-0.34494500	0.35495300
C	2.28625800	-1.79783700	-0.04063300
H	1.82070000	-2.74978400	-0.36679800
O	3.48738700	-1.66118400	-0.05948500

2-CN-4-CH_3-substituted

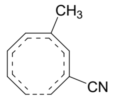

C	0.11089200	-1.97264900	-0.86408200
C	-0.93205500	-1.06969100	0.99182000
C	1.32845500	-2.13864000	-0.26015100
C	2.19196300	-1.08408200	0.13076700
C	2.01089700	0.28388100	0.20542200
C	0.96161900	1.25045300	0.00655100
C	-0.41863600	1.17523300	0.03655500
C	-1.29891000	0.11477800	0.39940500
H	-0.11768300	-1.04749200	-1.37830800
H	-0.49454100	-2.83503300	-1.12851200
H	0.00904700	-1.12086800	1.52131700
H	-1.68384300	-1.81425300	1.23404600
H	1.64054100	-3.14438800	0.01630000
H	3.18617400	-1.40660400	0.43592100
H	2.95107600	0.79262400	0.42529200
H	-0.93398600	2.08785300	-0.25413000
C	1.52990000	2.64381900	-0.23733800
H	0.74364200	3.37972100	-0.41676100
H	2.10638800	2.97590400	0.63412100
H	2.20700400	2.64842500	-1.09776300
C	-2.67605700	0.26437800	-0.01037800
N	-3.78031900	0.38274700	-0.34042200

2-CN-4-NH_2-substituted

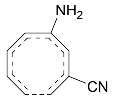

C	0.19122100	-1.93818900	-0.85844800

C	-0.88755200	-1.09318000	0.99173600
C	1.42817400	-2.06520700	-0.28192500
C	2.24230300	-0.98069300	0.12871700
C	2.00764900	0.37524500	0.23751700
C	0.92208800	1.29619400	0.00380400
C	-0.46108800	1.16111600	0.00121700
C	-1.29212600	0.08273200	0.40133000
H	-0.07753200	-1.01764600	-1.36228000
H	-0.38060900	-2.82113800	-1.12996800
H	0.05185100	-1.11684800	1.52633700
H	-1.61971400	-1.85654600	1.23388500
H	1.78660200	-3.05991100	-0.02410000
H	3.24250600	-1.26614500	0.45074600
H	2.90084100	0.92972000	0.52453500
H	-1.00443800	2.04070400	-0.33817800
N	1.40561900	2.60901300	-0.16223200
H	2.20784600	2.69919700	-0.77356400
H	0.70166900	3.30629400	-0.36776700
C	-2.67585900	0.17974900	-0.00566800
N	-3.78531600	0.25609100	-0.33053000

2-CN-4-OH-substituted

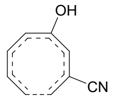

C	0.24315500	-1.92364100	-0.86654800
C	-0.88157400	-1.08528800	1.00101100
C	1.46892400	-2.02921300	-0.26701100
C	2.26669000	-0.92992300	0.14010400
C	2.00507000	0.42207300	0.22557200
C	0.89120400	1.30218700	-0.00205300
C	-0.48366000	1.17504900	0.02063200
C	-1.30172800	0.07812300	0.40265600
H	-0.04195200	-1.00417100	-1.36322800
H	-0.31377500	-2.81468700	-1.14177300
H	0.06001600	-1.09684600	1.53217300
H	-1.59815500	-1.86577900	1.23511000
H	1.83996800	-3.01714700	-0.00152100
H	3.27079900	-1.19456100	0.46683400
H	2.88840800	0.99945800	0.50318100
H	-1.00494200	2.07396700	-0.29664300
O	1.26905500	2.61085900	-0.22690200
H	2.16754400	2.64236400	-0.58238600
C	-2.68502500	0.15240900	-0.01172200
N	-3.79409800	0.20283900	-0.34176800

2-CN-4-F-substituted

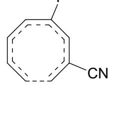

C	0.30715100	-1.93357500	-0.83752600
C	-0.85412000	-1.07697700	1.02236500
C	1.52814000	-1.98957200	-0.22646200

C	2.31704200	-0.85792300	0.11131800
C	2.01958700	0.48639400	0.15075100
C	0.85453700	1.29859000	-0.00683700
C	-0.51124300	1.17975300	0.03952800
C	-1.30988400	0.05789000	0.40172800
H	0.00147700	-1.03677100	-1.36312700
H	-0.23461100	-2.84393300	-1.07691700
H	0.08748500	-1.04714600	1.55352900
H	-1.53889800	-1.88447800	1.25970500
H	1.91823700	-2.95877100	0.07859000
H	3.34360100	-1.08356300	0.39353000
H	2.88291400	1.12746200	0.31962300
H	-1.04073900	2.08549100	-0.24281100
F	1.24095700	2.59107900	-0.23197300
C	-2.68869900	0.09117800	-0.02807600
N	-3.79473500	0.11249300	-0.37072800

2-CN-4-CHO-substituted

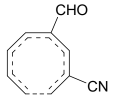

C	0.89251300	2.00124000	-0.88911600
C	1.53204900	0.74210500	0.99726200
C	-0.15092100	2.60748000	-0.24951000
C	-1.36506700	1.97091800	0.13334500
C	-1.72283400	0.64387400	0.20876300
C	-1.09125700	-0.63654700	0.03374800
C	0.20235300	-1.12263100	0.05798800
C	1.43076400	-0.48837600	0.39672700
H	0.74887100	1.05634300	-1.39795300
H	1.78222500	2.56519900	-1.15398300
H	0.67356800	1.14431000	1.51710200
H	2.50554300	1.16011700	1.23366500
H	-0.04401100	3.65123700	0.04213400
H	-2.16064300	2.65579000	0.42167600
H	-2.78433800	0.50204800	0.40835000
H	0.30397100	-2.17134900	-0.22073200
C	-2.09195900	-1.74355900	-0.17341400
H	-1.64689500	-2.73597800	-0.38651000
O	-3.29098400	-1.61566800	-0.11580100
C	2.64190500	-1.15467700	-0.01995600
N	3.61204400	-1.68876300	-0.35890900

2-CN-4-CN-substituted

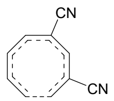

C	0.86254900	1.96454200	-0.92946400
C	1.47222100	0.71343800	0.99525200
C	-0.14294200	2.62457200	-0.28420400
C	-1.37509100	2.04778400	0.13265400
C	-1.79326600	0.74101500	0.24069800
C	-1.20952400	-0.57142900	0.07658200

C	0.06303400	-1.11658800	0.08952700
C	1.31400900	-0.51524500	0.40666700
H	0.67777300	1.01386400	-1.41387300
H	1.77042000	2.48714500	-1.21676000
H	0.63793900	1.16325600	1.51551900
H	2.46499400	1.09476700	1.21201700
H	0.01099100	3.66841600	-0.01604700
H	-2.13471800	2.76971200	0.42644500
H	-2.85369500	0.66107400	0.47243800
H	0.11439100	-2.16932000	-0.17700000
C	-2.23974900	-1.57866600	-0.11259300
N	-3.06931800	-2.37169800	-0.26480900
C	2.49583700	-1.22865100	-0.01817000
N	3.44495100	-1.79595100	-0.36153800

2-CN-4-NO_2-substituted

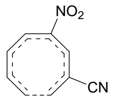

C	1.63888200	1.76639300	-0.97106000
C	1.89084200	0.46747200	1.01158700
C	0.82712100	2.67817200	-0.36070200
C	-0.51289100	2.44586200	0.05779300
C	-1.25171600	1.29411700	0.20818400
C	-1.00261600	-0.10938000	0.08548200
C	0.06703700	-0.96918800	0.12967800
C	1.42618700	-0.69709200	0.45669400
H	1.22637200	0.87446900	-1.42572400
H	2.65083000	2.03438900	-1.26094900
H	1.19735700	1.13714600	1.50127500
H	2.94654600	0.58576000	1.23411400
H	1.23651900	3.65899600	-0.12458000
H	-1.06492800	3.34621000	0.31934400
H	-2.29433100	1.48041900	0.44536900
H	-0.16149500	-1.99924000	-0.12458700
N	-2.30431400	-0.87402900	-0.08668600
O	-2.26346200	-1.93380200	-0.68026800
O	-3.31641000	-0.38675900	0.37982600
C	2.39028600	-1.70150900	0.07135200
N	3.17050100	-2.49778900	-0.24113900

2-NO_2-4-CH_3-substituted

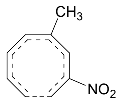

C	0.45349800	-1.98383300	-0.89573800
C	-0.59661800	-1.12384200	0.98230700
C	1.66735500	-2.13524500	-0.28418900
C	2.51901000	-1.07296600	0.12080200
C	2.32169100	0.28982100	0.21624700
C	1.25937800	1.24771100	0.03085700
C	-0.11777500	1.15554100	0.07092700
C	-0.95313700	0.06625200	0.41917200

H	0.21093100	-1.05557500	-1.39855200
H	-0.14325400	-2.85061600	-1.16477900
H	0.34008000	-1.16792000	1.51950800
H	-1.34624100	-1.88012600	1.18229000
H	1.99101100	-3.13887300	-0.01292400
H	3.51689700	-1.38848400	0.42104700
H	3.25468200	0.80704400	0.44664800
H	-0.66127900	2.05681200	-0.19184700
C	1.80924500	2.65020500	-0.19793200
H	2.49266400	2.67096500	-1.05311400
H	1.01249800	3.37434900	-0.37810500
H	2.37433900	2.98448200	0.68028100
N	-2.36943700	0.18767700	-0.00994500
O	-2.78277200	1.29120200	-0.32148700
O	-3.04624600	-0.82716000	-0.02295700

2-NO_2-4-NH_2-substituted

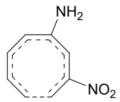

C	0.52387200	-1.94194800	-0.88946300
C	-0.55971300	-1.13888300	0.97694600
C	1.75686100	-2.06232800	-0.30642400
C	2.56493800	-0.97587700	0.11821500
C	2.32149200	0.37524300	0.24689100
C	1.22711600	1.29259900	0.03006400
C	-0.15314100	1.14897100	0.04015200
C	-0.94675300	0.04758700	0.42068100
H	0.24716800	-1.01713400	-1.38186000
H	-0.04357100	-2.82558200	-1.16729000
H	0.37269800	-1.16083800	1.52309100
H	-1.29294300	-1.91165500	1.17228100
H	2.12164200	-3.05666300	-0.05569200
H	3.56739000	-1.25957500	0.43454100
H	3.21095700	0.93192600	0.54118200
H	-0.72038800	2.02343700	-0.26196900
N	1.69976900	2.61015500	-0.12016700
H	2.50306400	2.71488800	-0.72767600
H	0.98931400	3.30273900	-0.31964000
N	-2.36969100	0.13117500	-0.00758100
O	-2.81202200	1.22346200	-0.32070300
O	-3.02221700	-0.89884100	-0.01493500

2-NO_2-4-OH-substituted

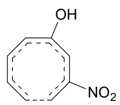

C	0.57075500	-1.92498000	-0.89827600
C	-0.55463800	-1.13059200	0.98501900
C	1.79342000	-2.02700300	-0.29420400
C	2.58670400	-0.92779200	0.12748600
C	2.31888400	0.42023000	0.23557900
C	1.19906500	1.29948900	0.02606700
C	-0.17316700	1.16409000	0.05989500

C	-0.95403000	0.04602900	0.42251000
H	0.27948200	-1.00115800	-1.38384600
H	0.01685700	-2.81568700	-1.18020900
H	0.37961100	-1.14512300	1.52822500
H	-1.27578400	-1.91708700	1.17134200
H	2.16944600	-3.01544000	-0.03730200
H	3.59279700	-1.19271400	0.44766800
H	3.19995300	0.99722600	0.52102200
H	-0.71805400	2.05791700	-0.22285500
O	1.56931000	2.61242600	-0.17880000
H	2.46780400	2.65558600	-0.53297100
N	-2.37725500	0.11244200	-0.01189200
O	-3.01694900	-0.92576700	-0.01450500
O	-2.82652200	1.19741100	-0.33323100

2-NO_2-4-F-substituted

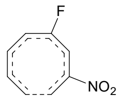

C	0.60739900	-1.93022300	-0.87689100
C	-0.53920300	-1.11575600	1.00665800
C	1.82616900	-1.99809800	-0.26450600
C	2.62441300	-0.87617000	0.09011800
C	2.33698200	0.46802800	0.16064000
C	1.17567400	1.29258400	0.03020200
C	-0.18831200	1.17885600	0.08928700
C	-0.95952000	0.04228400	0.42699900
H	0.30430100	-1.02402400	-1.38828000
H	0.05902900	-2.83400300	-1.12558400
H	0.39421400	-1.10723000	1.55203700
H	-1.24144600	-1.92006200	1.19064000
H	2.21112800	-2.97352800	0.02735500
H	3.65123400	-1.11458000	0.36049300
H	3.20598600	1.09890900	0.33853000
H	-0.73277100	2.08447800	-0.15524300
F	1.56926200	2.58597300	-0.16607800
N	-2.37509700	0.08313800	-0.02473500
O	-2.81997100	1.15137000	-0.40346600
O	-3.01140000	-0.95570900	0.01507200

2-NO_2-4-CHO-substituted

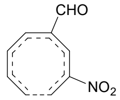

C	0.38174800	2.16636400	-0.92244700
C	1.14764200	1.00742300	0.99274100
C	-0.71172600	2.66310800	-0.27516900
C	-1.85696600	1.91026600	0.11909000
C	-2.08102100	0.55679900	0.21311100
C	-1.32481500	-0.65778900	0.04985700
C	0.00917800	-1.00983800	0.08579900
C	1.14042300	-0.22814900	0.41820200
H	0.33444300	1.20623700	-1.42182400
H	1.21193900	2.81318800	-1.19087200
H	0.25929800	1.32887100	1.51790100

H	2.08484600	1.51434200	1.19156300
H	-0.70860400	3.71332500	0.01318200
H	-2.71581800	2.51654000	0.40132400
H	-3.12192600	0.31226800	0.42141500
H	0.23671700	-2.04174900	-0.17102200
C	-2.20762700	-1.86237600	-0.14523700
H	-1.66385000	-2.80426100	-0.35741600
O	-3.41299700	-1.85586800	-0.07873600
N	2.45143300	-0.76957300	-0.01536600
O	2.50150300	-1.94003900	-0.34952000
O	3.40423200	-0.00991800	-0.01079200

2-NO_2-4-CN-substituted

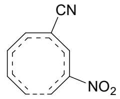

C	0.27932300	2.15133600	-0.95291900
C	1.05734100	1.02901500	0.99272700
C	-0.80571400	2.66155200	-0.30297900
C	-1.94599400	1.91755700	0.11799900
C	-2.17626700	0.56738000	0.24091100
C	-1.41356900	-0.65398900	0.09280600
C	-0.07815200	-1.01075900	0.12000800
C	1.04638300	-0.21035300	0.42975000
H	0.22800700	1.18054100	-1.43095500
H	1.10830700	2.79128700	-1.24063100
H	0.17403000	1.35951300	1.52096300
H	1.99613900	1.54011500	1.17224300
H	-0.80004900	3.71665300	-0.03445600
H	-2.80053700	2.52796500	0.40307500
H	-3.21481500	0.34201200	0.47568000
H	0.14531700	-2.04456400	-0.12334500
C	-2.29260500	-1.79743100	-0.08466800
N	-3.00829500	-2.69634000	-0.22560900
N	2.35712200	-0.74897100	-0.01688500
O	3.30284200	0.01898700	-0.03363300
O	2.40932600	-1.92176000	-0.33723300

2-NO_2-4-NO_2-substituted

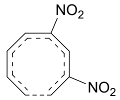

C	0.99793200	2.18058700	-0.96188900
C	1.54700100	0.92184400	0.98532600
C	0.05990400	2.90405700	-0.28640200
C	-1.20425300	2.42302800	0.16438000
C	-1.72735900	1.15772000	0.28685500
C	-1.23691600	-0.18028500	0.13101100
C	-0.02707600	-0.82514900	0.15047000
C	1.24877800	-0.28612200	0.43310700
H	0.73893000	1.24227500	-1.43740900
H	1.93542400	2.63597200	-1.26795500
H	0.77147400	1.44538900	1.52595300
H	2.57962900	1.21116600	1.14304200
H	0.29762600	3.93260200	-0.01969300

H	-1.89267300	3.20468000	0.47910500
H	-2.78246400	1.15787700	0.53987200
H	-0.04926900	-1.88344200	-0.08414500
N	-2.38451700	-1.15743500	-0.06181000
O	-2.14924500	-2.34697100	0.02316400
O	-3.48368700	-0.69392600	-0.30443700
N	2.39981300	-1.10334600	-0.03501400
O	2.18485400	-2.25805600	-0.35007200
O	3.49135200	-0.56294000	-0.07092500

5.3.11 2,5-substituted

2-CH_3-5-CH_3-substituted

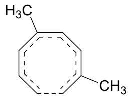

C	0.47946100	1.81018100	-0.72194500
C	1.39798200	0.64614600	1.00966800
C	-0.75801200	2.00369100	-0.15977200
C	-1.72479100	0.99933200	0.07641500
C	-1.67464500	-0.38712400	-0.00599400
C	-0.61429900	-1.32544400	-0.24577900
C	0.76780900	-1.38791700	-0.17383300
C	1.74615700	-0.47185600	0.29194600
H	0.65819200	0.94507700	-1.34783400
H	1.14904600	2.65396500	-0.86636300
H	0.44049300	0.68115700	1.51292700
H	2.16452400	1.32385600	1.37849600
H	-1.00263300	2.99564600	0.21795900
H	-2.69539100	1.38983400	0.37879900
H	-1.02987400	-2.29458100	-0.53025600
H	1.17631700	-2.32327700	-0.55875700
C	3.16921100	-0.67737800	-0.17069100
H	3.87181400	-0.07513400	0.41145800
H	3.46261600	-1.72877700	-0.07859600
H	3.28246600	-0.40279200	-1.22790100
C	-3.01918900	-1.09452600	0.13577500
H	-3.82869500	-0.39211100	0.34675100
H	-3.27181800	-1.62467600	-0.79054100
H	-2.99515800	-1.83881600	0.93912600

2-CH_3-5-NH_2-substituted

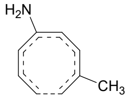

C	0.40730500	1.82339400	-0.73067600
C	1.35478600	0.67949100	0.99858000
C	-0.83877300	1.96935900	-0.16890300
C	-1.76353500	0.94081600	0.10318300
C	-1.67157600	-0.44580600	-0.00829000
C	-0.58756100	-1.34793400	-0.28867200
C	0.79212000	-1.37190100	-0.18683800
C	1.74256900	-0.43884200	0.30019200
H	0.62201200	0.96758300	-1.35802000
H	1.03882600	2.69563700	-0.87528000

H	0.39127000	0.69307000	1.49194100
H	2.10066200	1.37611700	1.37464600
H	-1.11493200	2.95288800	0.20958500
H	-2.73374700	1.29274500	0.45310100
H	-0.98776900	-2.29880600	-0.64158600
H	1.22848800	-2.28559800	-0.59271700
C	3.17666400	-0.61224000	-0.14230100
H	3.85869100	-0.01052600	0.46446500
H	3.48736300	-1.65999300	-0.06651400
H	3.30526800	-0.31282200	-1.19119600
N	-2.89995200	-1.14828000	0.07303800
H	-3.69740000	-0.57152200	0.31003900
H	-2.87106100	-1.97883100	0.65262600

2-CH_3-5-OH-substituted

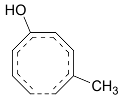

C	0.35454800	1.83147700	-0.72966300
C	1.34461700	0.69086400	1.00008800
C	-0.88681300	1.94963700	-0.15699600
C	-1.79359600	0.89707200	0.09334600
C	-1.65325200	-0.48102400	0.00132700
C	-0.57070700	-1.38764100	-0.23292400
C	0.80945100	-1.37962200	-0.16241000
C	1.74497100	-0.41233100	0.28814000
H	0.58402600	0.98095100	-1.35903600
H	0.97324100	2.71371100	-0.86765600
H	0.38686400	0.68353800	1.50500800
H	2.07830000	1.40702500	1.36258300
H	-1.18288700	2.92272600	0.23236000
H	-2.78061000	1.21974500	0.42708400
H	-0.97869000	-2.35938800	-0.50634300
H	1.25808000	-2.29825200	-0.54219500
C	3.17416900	-0.55397700	-0.17942700
H	3.85018400	0.07983900	0.40061600
H	3.51565000	-1.59071900	-0.08714100
H	3.27277900	-0.27489700	-1.23691500
O	-2.81643400	-1.22111100	0.13656200
H	-3.58579700	-0.64212300	0.05024900

2-CH_3-5-F-substituted

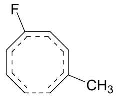

C	0.30397100	1.84967700	-0.71490900
C	1.32750500	0.68654800	1.01404400
C	-0.93707700	1.94946100	-0.14291400
C	-1.82979700	0.87454000	0.07089600
C	-1.62182300	-0.48476400	-0.00360000
C	-0.55764600	-1.40756600	-0.21264000
C	0.82181100	-1.38631200	-0.15969700
C	1.74035800	-0.39493000	0.27976600
H	0.54482200	1.00446000	-1.34744500
H	0.92132500	2.73516000	-0.83708600

H	0.37040600	0.66144800	1.51984500
H	2.04883700	1.41522600	1.37554200
H	-1.25260100	2.91320100	0.25274700
H	-2.84833000	1.13128200	0.35203500
H	-0.97239400	-2.38282500	-0.46266200
H	1.28146400	-2.30262400	-0.53066200
C	3.16715800	-0.50616300	-0.20211000
H	3.83068600	0.16177700	0.35323200
H	3.53977900	-1.52983700	-0.08746300
H	3.24359300	-0.25238600	-1.26739700
F	-2.79937200	-1.18198000	0.12292300

2-CH_3-5-CHO-substituted

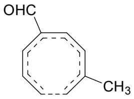

C	0.84141000	1.83273200	-0.73918700
C	1.70973900	0.58108600	1.07339500
C	-0.39121500	2.05977800	-0.18678900
C	-1.38781300	1.07633100	0.00001200
C	-1.34595000	-0.30759500	-0.09782300
C	-0.32450500	-1.29815300	-0.30228900
C	1.04513100	-1.40526900	-0.18619700
C	2.03557900	-0.50822400	0.31416100
H	1.01481400	0.94961900	-1.34072800
H	1.55134400	2.64683200	-0.85728200
H	0.73150900	0.65546100	1.52986900
H	2.48475900	1.23323600	1.46856200
H	-0.61870700	3.05583500	0.18866500
H	-2.37514000	1.45136000	0.26669400
H	-0.76939900	-2.24759700	-0.60700000
H	1.44206100	-2.35255000	-0.55226600
C	3.46135900	-0.75092500	-0.11938700
H	4.16013900	-0.10852600	0.42235300
H	3.74806500	-1.79348300	0.05761400
H	3.58311100	-0.56113500	-1.19331000
C	-2.70628700	-0.94074600	-0.00066200
H	-2.72871900	-2.02999300	-0.21113700
O	-3.73106800	-0.36664300	0.28707000

2-CH_3-5-CN-substituted

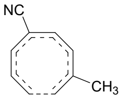

C	0.91401500	1.80759100	-0.71862300
C	1.68601600	0.49246100	1.05503600
C	-0.28138200	2.15299000	-0.14480400
C	-1.36694200	1.27214900	0.05485800
C	-1.44720900	-0.11374200	-0.04749700
C	-0.53172400	-1.20599000	-0.27218800
C	0.82584700	-1.42733300	-0.18354300
C	1.90185200	-0.62380300	0.29328600
H	0.98121000	0.92410800	-1.34062500
H	1.69756400	2.54951800	-0.84357700
H	0.73088900	0.64075800	1.54180800

H	2.52287400	1.07326600	1.43532900
H	-0.40300900	3.16032700	0.24797900
H	-2.30174100	1.75019700	0.33992000
H	-1.07485000	-2.10334100	-0.56394700
H	1.12727100	-2.40507700	-0.55982100
C	3.29083000	-0.99054300	-0.17014300
H	4.05534700	-0.41766600	0.36040900
H	3.48536800	-2.05567900	-0.00356800
H	3.40852600	-0.80589100	-1.24541200
C	-2.81322800	-0.59601200	0.06237900
N	-3.89970000	-0.98816000	0.15127700

2-CH_3-5-NO_2-substituted

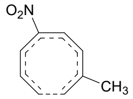

C	1.35019900	1.76568700	-0.83776900
C	2.04735700	0.54469100	1.03963700
C	0.16336100	2.19414500	-0.30479100
C	-0.96255700	1.37962100	-0.05165600
C	-1.08538700	0.00461900	-0.06569200
C	-0.24112900	-1.13742600	-0.21560600
C	1.10430400	-1.40881200	-0.08727700
C	2.21234200	-0.62677500	0.35186900
H	1.39578200	0.84089000	-1.39860400
H	2.16114000	2.47035800	-0.99946400
H	1.09825100	0.77507400	1.50568400
H	2.91024900	1.10665500	1.38870600
H	0.07962800	3.23027400	0.01605900
H	-1.87589400	1.90471500	0.20744100
H	-0.82528700	-2.01438800	-0.47483600
H	1.36571800	-2.42054700	-0.39709700
C	3.58551800	-1.08614300	-0.07509300
H	3.72241800	-2.15116400	0.14121700
H	3.72277500	-0.95702100	-1.15625900
H	4.37350900	-0.52955800	0.43795400
N	-2.53751300	-0.41416800	0.06239700
O	-3.29978500	0.33692800	0.64368500
O	-2.87643300	-1.47864800	-0.42234900

2- NH_2-5-CH_3-substituted

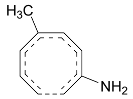

C	0.48378200	1.81935600	-0.73236700
C	1.46023400	0.64039000	1.00072100
C	-0.74555300	2.01634300	-0.16070300
C	-1.71262200	1.00870700	0.07677000
C	-1.66514300	-0.37454900	-0.00555800
C	-0.60452300	-1.31757900	-0.25043800
C	0.77030800	-1.40259400	-0.17020800
C	1.76393800	-0.48176600	0.27154100
H	0.65880800	0.95255400	-1.35750100
H	1.16075800	2.65753800	-0.87546100
H	0.52272600	0.67726900	1.53831700

H	2.24725800	1.31083300	1.33876900
H	-0.98477300	3.00625400	0.22486400
H	-2.68291800	1.39914600	0.38130900
H	-1.02665500	-2.28498800	-0.53099300
H	1.17890400	-2.35215400	-0.51563800
N	3.08119000	-0.76711200	-0.12675000
H	3.76020100	-0.06219000	0.13226500
H	3.18763000	-1.03911700	-1.09725800
C	-3.00602900	-1.08565300	0.15195600
H	-3.81603000	-0.38323100	0.36152200
H	-3.26505500	-1.62664400	-0.76650400
H	-2.97552900	-1.82141800	0.96327700

2- NH_2-5-NH_2-substituted

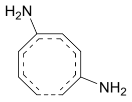

C	0.86703200	-1.43070100	-0.78356000
C	-0.86703200	-1.43070000	0.78356100
C	1.79721400	-0.69875900	-0.07235500
C	1.72534900	0.67464100	0.24671800
C	0.69526600	1.61135500	0.14928300
C	-0.69526700	1.61135500	-0.14928300
C	-1.72534900	0.67464000	-0.24671900
C	-1.79721400	-0.69875900	0.07235500
H	0.17248900	-0.93671800	-1.44663400
H	1.04703800	-2.48370300	-0.98023800
H	-0.17248900	-0.93671700	1.44663500
H	-1.04703800	-2.48370300	0.98023900
H	2.65368700	-1.23319000	0.33812400
H	2.65132500	1.08930500	0.64370700
H	-1.05171800	2.62509500	-0.33428900
H	-2.65132500	1.08930400	-0.64370900
N	-2.95249005	-1.41964026	-0.48133035
H	-2.80993343	-2.20461004	-1.08424001
H	-3.88094874	-1.12506589	-0.25507740
N	1.17581171	2.97801803	0.39869826
H	2.05072198	2.94079948	0.88155102
H	0.50757158	3.46961864	0.95707446

2- NH_2-5-OH-substituted

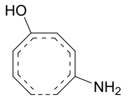

C	0.36260300	1.83783100	-0.73597500
C	1.40551800	0.69170800	0.98505500
C	-0.87170300	1.96221100	-0.15451000
C	-1.78002400	0.90738100	0.09377900
C	-1.64324400	-0.46780800	0.00252700
C	-0.56228500	-1.38239500	-0.22456700
C	0.81317800	-1.39783300	-0.14824900
C	1.76222200	-0.42161400	0.26650400
H	0.58351500	0.98787000	-1.36985800
H	0.99000700	2.71422700	-0.87271100
H	0.47266200	0.68460000	1.53278600

H	2.15984800	1.40400300	1.31163300
H	-1.16046600	2.93353300	0.24379400
H	-2.76806600	1.23074100	0.42475200
H	-0.97818000	-2.35567700	-0.48005100
H	1.26235000	-2.33282200	-0.48196400
N	3.08983800	-0.64571800	-0.14323600
H	3.73488600	0.09114900	0.11402800
H	3.19878100	-0.89828300	-1.11891800
O	-2.80808400	-1.21047400	0.14274200
H	-3.57712500	-0.63240800	0.04983800

2- NH_2-5-F-substituted

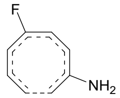

C	0.30612300	1.86238800	-0.72224000
C	1.38902400	0.68485500	1.00668000
C	-0.92750700	1.96207000	-0.14137900
C	-1.81846600	0.88157300	0.07055300
C	-1.61072600	-0.47441600	-0.00288700
C	-0.54568700	-1.40295300	-0.20834300
C	0.82832600	-1.40296600	-0.14866800
C	1.75964200	-0.40142300	0.25883900
H	0.54606500	1.01667800	-1.35499400
H	0.92919800	2.74443700	-0.84094200
H	0.45436200	0.65724800	1.55071100
H	2.12831100	1.41138000	1.33524200
H	-1.24045900	2.92280000	0.26274400
H	-2.83855800	1.13597000	0.34921400
H	-0.96654100	-2.37924400	-0.44356100
H	1.29083700	-2.33416600	-0.47332800
N	3.08366000	-0.59175200	-0.16929400
H	3.71863600	0.15637900	0.07979300
H	3.18890000	-0.85383100	-1.14250800
F	-2.78674800	-1.17668400	0.13192900

2- NH_2-5-CHO-substituted

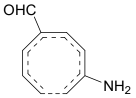

C	0.84686000	1.86558200	-0.75917500
C	1.75504000	0.52620000	1.13088800
C	-0.37790600	2.07450600	-0.20149100
C	-1.37364700	1.07981200	-0.01322300
C	-1.33501400	-0.29880500	-0.10500200
C	-0.30934800	-1.29122100	-0.33409300
C	1.04859500	-1.41142800	-0.21119200
C	2.05167000	-0.51708800	0.30686600
H	1.06235400	0.96968000	-1.32696000
H	1.55763200	2.68133800	-0.85923400
H	0.77366400	0.59505700	1.57810400
H	2.53511400	1.17530200	1.51951900
H	-0.61758500	3.06742800	0.17420700
H	-2.36196400	1.45446900	0.25195300
H	-0.75767400	-2.23218100	-0.65926300

H	1.44372700	-2.36202200	-0.57076200
N	3.35138400	-0.70168200	-0.18158500
H	4.09447900	-0.46533400	0.46387000
H	3.52122300	-1.58787100	-0.63965300
C	-2.68903600	-0.93474400	0.01691100
H	-2.71141300	-2.02509300	-0.18974000
O	-3.71281600	-0.36448600	0.31826500

2-NH_2-5-CN-substituted

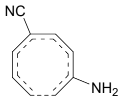

C	0.93277500	1.83401000	-0.73238600
C	1.72956600	0.42546200	1.11047800
C	-0.25538100	2.16946100	-0.15592500
C	-1.34717500	1.28389700	0.04169900
C	-1.43643200	-0.09529700	-0.05617800
C	-0.52305200	-1.19503300	-0.30745100
C	0.82027900	-1.43710600	-0.21076400
C	1.91356200	-0.64377200	0.28446800
H	1.03338000	0.93409800	-1.32567300
H	1.72518000	2.56983500	-0.83564300
H	0.77220100	0.57081100	1.59045100
H	2.57426300	0.99821100	1.48390000
H	-0.38194100	3.17464500	0.24030400
H	-2.28076900	1.76666700	0.32394100
H	-1.07424000	-2.07973800	-0.62206000
H	1.11451700	-2.41858700	-0.58293200
N	3.17811700	-0.94539900	-0.23176000
H	3.95613500	-0.78332100	0.39498000
H	3.25584100	-1.83505900	-0.70742500
C	-2.79775600	-0.57953600	0.08076900
N	-3.87995900	-0.97889900	0.19060200

2-NH_2-5-NO_2-substituted

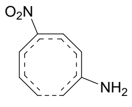

C	1.36246400	1.86613400	-0.69654800
C	2.07007900	0.38665100	1.13878700
C	0.17593300	2.23092400	-0.13555000
C	-0.95522100	1.38807600	0.02323300
C	-1.08212500	0.02436500	-0.09170400
C	-0.22794900	-1.10187100	-0.36662000
C	1.10456700	-1.38901400	-0.26641400
C	2.22120700	-0.65915500	0.27736300
H	1.44517000	0.97684300	-1.30798300
H	2.18185200	2.57607900	-0.76604500
H	1.11136800	0.56113400	1.60613600
H	2.93217300	0.90809200	1.54589800
H	0.07973200	3.22946300	0.28457700
H	-1.87595000	1.89188000	0.29949800
H	-0.80818000	-1.94844700	-0.71954300
H	1.37110900	-2.36046400	-0.68286600

N	3.47820800	-0.99635200	-0.23144500
H	4.25628500	-0.87533700	0.40425900
H	3.52926900	-1.87477800	-0.73076400
N	-2.51456800	-0.42451900	0.05921100
O	-3.39938500	0.41375800	0.02654000
O	-2.72337000	-1.61563600	0.22060800

2-OH-5-CH_3-substituted

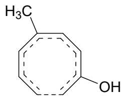

C	0.51291900	1.81560900	-0.73576200
C	1.48189100	0.63454500	0.96676400
C	-0.71507800	2.01755400	-0.15572700
C	-1.68335100	1.01855600	0.09662200
C	-1.65470900	-0.36783400	0.00454400
C	-0.61255500	-1.31674500	-0.26983300
C	0.76523900	-1.40672600	-0.21766700
C	1.74932200	-0.50367300	0.25062800
H	0.68023200	0.95503000	-1.37121200
H	1.18552400	2.65592500	-0.88725600
H	0.54914500	0.69174600	1.50930600
H	2.28926800	1.27955000	1.30715000
H	-0.94711000	3.01002600	0.22778100
H	-2.64376700	1.41679600	0.42135600
H	-1.04530200	-2.27602500	-0.56101300
H	1.18291500	-2.33205300	-0.60906900
O	3.01239100	-0.79346600	-0.20627200
H	3.66503800	-0.29192800	0.30176800
C	-3.00362000	-1.06099000	0.17380100
H	-3.80075900	-0.34949100	0.40097900
H	-3.28197700	-1.58826200	-0.74681000
H	-2.97267600	-1.80536200	0.97698400

2-OH-5-NH_2-substituted

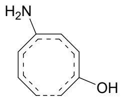

C	0.43593600	1.83849600	-0.76322300
C	1.44517700	0.66615200	0.96167500
C	-0.79218500	1.98397400	-0.16546300
C	-1.71236200	0.95944100	0.13699400
C	-1.65155000	-0.42776900	0.01616900
C	-0.59059400	-1.34337400	-0.29915200
C	0.78800500	-1.38930700	-0.22846100
C	1.74618800	-0.46906500	0.25653800
H	0.64711400	0.98118000	-1.38966800
H	1.06474100	2.71055500	-0.92113900
H	0.50122600	0.71134200	1.48618000
H	2.23393500	1.33072700	1.30783000
H	-1.05578600	2.96836300	0.21924000
H	-2.66509500	1.30852300	0.53394900
H	-0.99485400	-2.29628800	-0.64294500
H	1.23349500	-2.29060600	-0.64468100
O	3.02234300	-0.73481800	-0.18832800

H	3.65890000	-0.22480600	0.33114900
N	-2.89116100	-1.07298100	0.25856100
H	-3.68922400	-0.64591500	-0.19530300
H	-2.88675000	-2.07494200	0.12162000

2-OH-5-OH-substituted

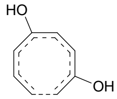

C	0.38776800	1.83608700	-0.74247000
C	1.42447700	0.68754300	0.95465400
C	-0.84523200	1.96373100	-0.15279300
C	-1.75259800	0.91567800	0.11364100
C	-1.63278400	-0.46342100	0.01326100
C	-0.56794000	-1.38199000	-0.24925500
C	0.81018400	-1.39917500	-0.20052200
C	1.74933500	-0.44068200	0.24751500
H	0.60397200	0.99017700	-1.38296500
H	1.00883700	2.71548000	-0.88912900
H	0.49248600	0.69930300	1.50228600
H	2.19752600	1.37727600	1.28647700
H	-1.12921800	2.93808900	0.24162900
H	-2.73066400	1.24590900	0.46645400
H	-0.99229500	-2.34676700	-0.52169500
H	1.26894200	-2.30708100	-0.58620700
O	3.02223500	-0.66798800	-0.21992700
H	3.65430300	-0.13729600	0.28438400
O	-2.80291000	-1.19133900	0.17151000
H	-3.56774700	-0.60709500	0.08192500

2-OH-5-F-substituted

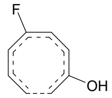

C	0.33335100	1.85761800	-0.72775600
C	1.40341600	0.68241800	0.97331300
C	-0.90048700	1.96363200	-0.14117400
C	-1.79170900	0.89089500	0.08917600
C	-1.59965600	-0.47012400	0.00787900
C	-0.55108300	-1.40259900	-0.23415900
C	0.82575700	-1.40391800	-0.20354400
C	1.74612000	-0.42069800	0.23947100
H	0.56588000	1.01614300	-1.36852200
H	0.95131400	2.74182100	-0.85752100
H	0.46840200	0.67303100	1.51582800
H	2.16075100	1.38670300	1.30977500
H	-1.20809300	2.92848400	0.25726600
H	-2.80316500	1.15319300	0.39087200
H	-0.98049000	-2.37099200	-0.48549300
H	1.29743100	-2.30748900	-0.58281000
O	3.01685100	-0.61546100	-0.24067400
H	3.64122300	-0.06253700	0.24960500
F	-2.78025800	-1.15755700	0.16412800

2-OH-5-CHO-substituted

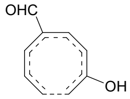

C	0.86631000	1.84268000	-0.75772100
C	1.78307300	0.55967300	1.05228900
C	-0.35890000	2.06990400	-0.19249900
C	-1.35393400	1.08609500	0.00760300
C	-1.32624400	-0.29673300	-0.10230800
C	-0.31530700	-1.29176800	-0.34449200
C	1.04799600	-1.42083200	-0.23812500
C	2.03859600	-0.53768300	0.28339200
H	1.04203000	0.95889800	-1.35771700
H	1.57631800	2.65628200	-0.87932200
H	0.81728000	0.65564000	1.52630400
H	2.59093100	1.17757100	1.43727100
H	-0.58265400	3.06557900	0.18551400
H	-2.33464100	1.46352800	0.29540700
H	-0.77113600	-2.22981800	-0.66698500
H	1.46098700	-2.35558300	-0.61059700
O	3.30066500	-0.85218100	-0.14456100
H	3.95575800	-0.35836600	0.36878700
C	-2.68692200	-0.92120600	0.01862700
H	-2.72234600	-2.00726700	-0.20607900
O	-3.70073100	-0.34372600	0.33791400

2-OH-5-CN-substituted

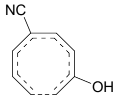

C	0.95639300	1.80891300	-0.72944300
C	1.76109200	0.44900200	1.03489200
C	-0.23006500	2.16143800	-0.14447700
C	-1.32078000	1.28798200	0.06591100
C	-1.42610800	-0.09435300	-0.04949900
C	-0.53079300	-1.19635200	-0.31618900
C	0.81672200	-1.44842100	-0.23937800
C	1.89795600	-0.66708000	0.26041400
H	1.01985700	0.92668700	-1.35375100
H	1.74559300	2.54496200	-0.85445500
H	0.82390600	0.62059200	1.54414300
H	2.63166400	0.98724900	1.40252500
H	-0.34077100	3.16801700	0.25276200
H	-2.24463700	1.77479200	0.37183600
H	-1.09063300	-2.07648300	-0.62766900
H	1.12934600	-2.41497200	-0.62782600
C	-2.79437100	-0.55977300	0.08783600
N	-3.88327000	-0.93947800	0.19936700
O	3.11288200	-1.09075600	-0.20346800
H	3.82523400	-0.67659100	0.30420800

2-OH-5-NO_2-substituted

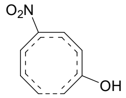

C	1.38404100	1.83648800	-0.69627600
C	2.11123000	0.41526300	1.05910300
C	0.20020100	2.21845100	-0.12529000
C	-0.93062500	1.38880900	0.04660200
C	-1.07283100	0.02246000	-0.08203200
C	-0.23645600	-1.10653800	-0.36329500
C	1.10025400	-1.40369300	-0.28400300
C	2.20694400	-0.68199200	0.25264100
H	1.43033200	0.96668500	-1.33897600
H	2.20032000	2.54753400	-0.78904700
H	1.17730300	0.61741500	1.56291500
H	3.00093800	0.90321600	1.45015600
H	0.12103200	3.21813800	0.29564000
H	-1.84211200	1.89801100	0.34236300
H	-0.82651900	-1.95150000	-0.70325400
H	1.38361200	-2.36438700	-0.70761200
O	3.40641400	-1.14328700	-0.21232200
H	4.13202600	-0.76708300	0.30607300
N	-2.51411900	-0.40773100	0.06302800
O	-3.38407300	0.44579500	0.03629400
O	-2.74167200	-1.59618300	0.21300800

2-F-5-CH_3-substituted

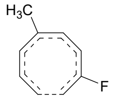

C	0.54246900	1.81488700	-0.74860600
C	1.52390100	0.64635200	0.96085200
C	-0.67741600	2.01528300	-0.15010800
C	-1.65349800	1.02642000	0.10788400
C	-1.64843900	-0.36097700	0.00543400
C	-0.62164200	-1.32015700	-0.27374200
C	0.75825400	-1.41020400	-0.22340300
C	1.72171900	-0.50869900	0.26232800
H	0.70050600	0.95512200	-1.38715300
H	1.21461700	2.65426700	-0.90478600
H	0.60473000	0.75600800	1.51707400
H	2.38095900	1.23900800	1.26394700
H	-0.89815900	3.00746100	0.24104500
H	-2.60459100	1.43623900	0.44489500
H	-1.05998000	-2.27617800	-0.56573800
H	1.18211800	-2.33128900	-0.61755200
C	-3.00839700	-1.03241700	0.17167100
F	2.99279400	-0.78037300	-0.14297000
H	-3.79502100	-0.30835200	0.39486600
H	-3.29103700	-1.55592800	-0.74952000
H	-2.99099800	-1.77593000	0.97578600

2-F-5-NH_2-substituted

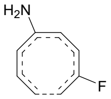

C	0.47243200	1.82801400	-0.75800900
C	1.47463600	0.68516000	0.95159500
C	-0.75713500	1.98185200	-0.16100500
C	-1.68926700	0.96776000	0.13561600
C	-1.64488200	-0.42099300	0.00406400
C	-0.59527700	-1.34451800	-0.31614900
C	0.78362800	-1.39446400	-0.23394800
C	1.71670200	-0.47466500	0.27325000
H	0.66670500	0.97672900	-1.39787600
H	1.10574100	2.69652700	-0.91543800
H	0.54673300	0.77185500	1.49808000
H	2.31165500	1.30168300	1.26333000
H	-1.00966300	2.96674500	0.22980700
H	-2.63889100	1.33924600	0.52025100
H	-1.01880900	-2.28177100	-0.67613200
H	1.23778900	-2.29065000	-0.65108400
F	3.00165600	-0.71415900	-0.11417600
N	-2.89031500	-1.08792900	0.10687000
H	-3.66972600	-0.48877900	0.34770400
H	-2.87925400	-1.91752400	0.68837400

2-F-5-OH-substituted

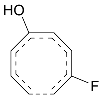

C	0.41189600	1.83941800	-0.75749700
C	1.46332500	0.70130800	0.95202400
C	-0.81224000	1.96271100	-0.14870100
C	-1.72497400	0.92199200	0.12589600
C	-1.62652900	-0.45925000	0.01426300
C	-0.57422300	-1.38593600	-0.25904300
C	0.80520800	-1.40064300	-0.21100300
C	1.72243400	-0.44416900	0.25958100
H	0.62272800	0.99284900	-1.39844500
H	1.03061700	2.71923000	-0.90897900
H	0.53951700	0.76506400	1.50861400
H	2.28691600	1.34217600	1.24938800
H	-1.08720900	2.93696700	0.25263300
H	-2.69403800	1.26207100	0.49338500
H	-1.00258300	-2.34669500	-0.53810200
H	1.27219400	-2.30142900	-0.60279200
F	3.00359200	-0.64891000	-0.15366100
O	-2.80345800	-1.17072000	0.17261900
H	-3.56218800	-0.57686000	0.09317600

2-F-5-F-substituted

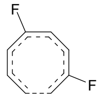

C	0.35447100	1.86109800	-0.74234200

C	1.44206600	0.70107600	0.96683400
C	-0.87050800	1.96273200	-0.13620800
C	-1.76664400	0.89630400	0.10153300
C	-1.59311200	-0.46768700	0.00940000
C	-0.55590800	-1.40660500	-0.24260700
C	0.82265400	-1.40392400	-0.21412300
C	1.72028100	-0.42234300	0.24988300
H	0.58006400	1.01951700	-1.38499300
H	0.96998500	2.74577200	-0.87777800
H	0.51552700	0.74362200	1.52092600
H	2.25119800	1.35871700	1.26659500
H	-1.16965700	2.92727700	0.26967200
H	-2.77115500	1.16809500	0.41749300
H	-0.98807000	-2.37225100	-0.49849500
H	1.30287200	-2.30013700	-0.59935400
F	3.00036500	-0.59453600	-0.17442600
F	-2.77931700	-1.14041000	0.16683900

2-F-5-CHO-substituted

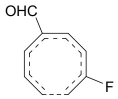

C	0.89007800	1.83283400	-0.76768700
C	1.82628100	0.58519200	1.03503100
C	-0.32899300	2.06089200	-0.18584600
C	-1.32979500	1.08608300	0.01891200
C	-1.31950400	-0.29805800	-0.09905700
C	-0.32177900	-1.30072000	-0.33687400
C	1.04526300	-1.42624200	-0.23278300
C	2.01196200	-0.53713900	0.29316100
H	1.05129200	0.95278400	-1.37709900
H	1.60000100	2.64498800	-0.89676000
H	0.88089100	0.73138600	1.53524200
H	2.68596800	1.15464400	1.37241700
H	-0.54202900	3.05624300	0.19939400
H	-2.30441600	1.47267500	0.31504500
H	-0.78038100	-2.23977100	-0.65152400
H	1.46614200	-2.35863500	-0.60175000
C	-2.69011700	-0.90658200	0.01335500
F	3.28268800	-0.83071400	-0.08444800
H	-2.73888900	-1.99067000	-0.21733200
O	-3.69539300	-0.31559800	0.33164100

2-F-5-CN-substituted

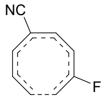

C	0.98408000	1.79859900	-0.74124900
C	1.80584900	0.46398100	1.01665400
C	-0.19533900	2.15118600	-0.13871500
C	-1.29156700	1.28884100	0.07887200
C	-1.41829000	-0.09285200	-0.04587400
C	-0.53964100	-1.20161600	-0.31385100
C	0.81133800	-1.45369700	-0.24083600

C	1.87036700	-0.66953600	0.26928400
H	1.03383900	0.92114800	-1.37321600
H	1.77266300	2.53357600	-0.87390600
H	0.89266900	0.68725200	1.54773800
H	2.72267200	0.94502300	1.34128900
H	-0.29458900	3.15660800	0.26477100
H	-2.20598500	1.78533600	0.39708700
H	-1.10409100	-2.08002100	-0.62082300
H	1.13015200	-2.41765600	-0.62997400
C	-2.79439500	-0.53795900	0.08666200
N	-3.88937200	-0.90032000	0.19327200
F	3.09820700	-1.07896700	-0.13683900

2-F-5-NO_2-substituted

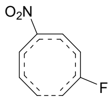

C	1.41673500	1.75358700	-0.84998100
C	2.15869400	0.50143800	1.00666500
C	0.24280500	2.18479300	-0.28989000
C	-0.89213200	1.38652200	-0.02901300
C	-1.05880600	0.01569300	-0.07312700
C	-0.24711000	-1.13906100	-0.27381000
C	1.09116100	-1.43879900	-0.15877900
C	2.17743800	-0.67517500	0.32585600
H	1.44866600	0.83928400	-1.42838900
H	2.23186700	2.45322900	-1.01154900
H	1.25111700	0.80176100	1.50836700
H	3.09410300	0.95524500	1.31727900
H	0.17882600	3.21633400	0.04931200
H	-1.78653000	1.92791500	0.26119500
H	-0.84851100	-1.99700900	-0.55527700
H	1.37488600	-2.43445200	-0.49069000
F	3.38963000	-1.16406400	-0.03479500
N	-2.51913800	-0.36955900	0.07753100
O	-2.87656100	-1.45342000	-0.34629600
O	-3.26716900	0.42431700	0.61787800

2-CHO-5-CH_3-substituted

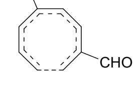

C	-0.11714200	1.93259500	-0.80414600
C	0.95121800	1.05116200	1.02787900
C	-1.36989300	1.92012100	-0.24916600
C	-2.15587900	0.77256800	0.02837500
C	-1.87283200	-0.58376700	-0.00348600
C	-0.65999400	-1.33211000	-0.21493000
C	0.70020600	-1.14973500	-0.08457700
C	1.46836400	-0.05812300	0.39841900
H	0.23520100	1.07827300	-1.36799300
H	0.38602800	2.87773800	-0.98832600
H	-0.00858700	0.99779100	1.52237400
H	1.61983700	1.84715500	1.35257000

H	-1.79124500	2.86807100	0.08361200
H	-3.18148000	1.00633600	0.30897100
H	-0.89454400	-2.35726300	-0.50714000
H	1.30701700	-1.99094800	-0.41554400
C	2.92340200	-0.06287600	0.11507700
H	3.45794300	0.85948100	0.42729300
O	3.53056100	-0.97664400	-0.39861300
C	-3.07342500	-1.50803000	0.15507100
H	-3.98834500	-0.95398600	0.37541500
H	-3.23930800	-2.07379800	-0.76966800
H	-2.91115000	-2.23652300	0.95625100

2-CHO-5-NH_2-substituted

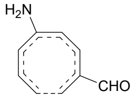

C	-0.20457200	1.95008800	-0.83843900
C	0.91024300	1.05166000	1.04361500
C	-1.44529300	1.86944900	-0.26483700
C	-2.17836200	0.70490000	0.06598800
C	-1.86167600	-0.64754500	0.00376600
C	-0.63016200	-1.35487300	-0.27919800
C	0.71496500	-1.13164100	-0.12279100
C	1.45483100	-0.03567000	0.40593600
H	0.21137200	1.11363000	-1.38504100
H	0.24261000	2.92251200	-1.02251200
H	-0.06938500	0.99118100	1.49702900
H	1.56057800	1.85369100	1.38951300
H	-1.90399500	2.80077400	0.06661600
H	-3.18807700	0.89571800	0.42741900
H	-0.83781100	-2.35660500	-0.65710700
H	1.35065200	-1.93459600	-0.49292400
C	2.91162300	-0.01463900	0.14552400
H	3.42986000	0.90249500	0.49753300
O	3.53895400	-0.90308100	-0.39014600
N	-2.93312500	-1.51304700	0.25535800
H	-3.84303100	-1.22245000	-0.07561800
H	-2.76211400	-2.50074900	0.13137600

2-CHO-5-OH-substituted

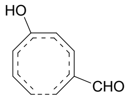

C	-0.22919900	1.92654200	-0.81710500
C	0.89307400	1.05933700	1.03209500
C	-1.47253100	1.84595600	-0.25053400
C	-2.20154700	0.66718900	0.04129900
C	-1.84421900	-0.67010800	0.00233100
C	-0.61850600	-1.39406400	-0.20699600
C	0.72695600	-1.15297800	-0.07413500
C	1.45216600	-0.02557800	0.40103100
H	0.17403100	1.09467200	-1.38054600
H	0.22393900	2.89786400	-0.99182400

H	-0.07090800	0.97428400	1.51489600
H	1.52817700	1.88264300	1.35507900
H	-1.93627300	2.77005200	0.09245400
H	-3.23246800	0.84263000	0.35062700
H	-0.85253500	-2.41715400	-0.49511600
H	1.36648200	-1.97054100	-0.40251400
C	2.90456400	0.02452800	0.11322600
H	3.40718200	0.96156300	0.43411000
O	3.54115800	-0.86159200	-0.41324800
O	-2.86024900	-1.58547500	0.15964100
H	-3.71945300	-1.14442600	0.10442400

2-CHO-5-F-substituted

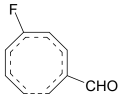

C	-0.27429000	1.92839900	-0.79920100
C	0.87337000	1.05191100	1.04326600
C	-1.51867000	1.83623200	-0.23909200
C	-2.23260800	0.63818600	0.01514500
C	-1.81373900	-0.67026200	-0.00278200
C	-0.60634700	-1.41529200	-0.17450600
C	0.73964800	-1.16343200	-0.06011300
C	1.44451800	-0.01698500	0.39787800
H	0.13380400	1.10267100	-1.36867700
H	0.17986800	2.90167300	-0.96031100
H	-0.08701200	0.94991100	1.53012700
H	1.49493500	1.88625500	1.36353500
H	-1.99795000	2.74999500	0.10834200
H	-3.28343700	0.74346600	0.27347000
H	-0.84735800	-2.44477200	-0.43326000
H	1.38693400	-1.97873100	-0.37768000
C	2.89571900	0.06031800	0.09634500
H	3.38512900	1.00580800	0.41142300
O	3.53932500	-0.81636400	-0.43497700
F	-2.85834600	-1.54220200	0.14124500

2-CHO-5-CHO-substituted

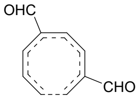

C	0.33063000	1.99577900	-0.80846400
C	1.31102300	0.98652500	1.07988500
C	-0.92689500	2.07443700	-0.27277500
C	-1.79241900	0.97727700	-0.05023900
C	-1.57607400	-0.39047500	-0.09612000
C	-0.43422100	-1.24786900	-0.26029800
C	0.92995900	-1.16850200	-0.09536000
C	1.75467500	-0.12890300	0.41576100
H	0.63392400	1.11412500	-1.35865500
H	0.91730300	2.89648300	-0.96705000
H	0.33120600	1.00719100	1.53570000
H	2.02712000	1.72668800	1.43337600
H	-1.29104100	3.04599000	0.05664900
H	-2.82232800	1.23505000	0.19301700

H	-0.74326700	-2.25072500	-0.56023900
H	1.48638400	-2.04887400	-0.41136000
C	3.21626600	-0.22418600	0.15364600
H	3.80751100	0.64984000	0.49817800
O	3.76189200	-1.16443700	-0.37650200
C	-2.84988600	-1.18971300	0.01298300
H	-2.73545300	-2.27709700	-0.17114900
O	-3.93560600	-0.73617600	0.28617800

2-CHO-5-CN-substituted

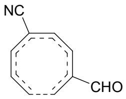

C	0.35986100	2.01246900	-0.78841600
C	1.28698600	0.96401100	1.06891500
C	-0.88390600	2.17784000	-0.23911300
C	-1.82147300	1.14477700	-0.00443600
C	-1.68651100	-0.23782100	-0.05097900
C	-0.60714000	-1.17845500	-0.22969100
C	0.76112200	-1.17117900	-0.08421600
C	1.65440200	-0.18229900	0.40807800
H	0.59008500	1.11903700	-1.35462400
H	1.00378600	2.87199000	-0.95213100
H	0.32350900	1.03771400	1.55349000
H	2.05179200	1.66223600	1.40559100
H	-1.17581300	3.16952300	0.10093200
H	-2.82202800	1.48481200	0.25307900
H	-0.98980700	-2.15523600	-0.52040400
H	1.25827100	-2.08315100	-0.40967300
C	3.10283500	-0.36638400	0.11985900
H	3.75149300	0.47328800	0.44566400
O	3.58004000	-1.34209800	-0.41100200
C	-2.96320400	-0.92175600	0.06784600
N	-3.97849300	-1.47009200	0.16557300

2-CHO-5-NO_2-substituted

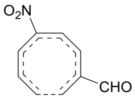

C	0.83717100	2.08722100	-0.73847800
C	1.68056000	0.94909600	1.09576000
C	-0.40281000	2.30798200	-0.19921600
C	-1.40818900	1.33144500	-0.00964500
C	-1.34123300	-0.04406400	-0.07301300
C	-0.34067500	-1.04770300	-0.25768600
C	1.02644700	-1.11868900	-0.11624500
C	1.97634500	-0.19945100	0.40300000
H	1.02341100	1.20197900	-1.33303300
H	1.53010500	2.91386600	-0.86781200
H	0.72347700	1.06969700	1.58341500
H	2.48753800	1.58809700	1.45114500
H	-0.63729300	3.30332700	0.17267500
H	-2.39279000	1.71638200	0.23431300
H	-0.78140900	-1.99018300	-0.56626600
H	1.47033300	-2.04529900	-0.47475100

C	3.41222600	-0.46071900	0.10548900
H	4.11057000	0.32751100	0.45622600
O	3.82836200	-1.44565500	-0.45793300
N	-2.71823200	-0.67474600	0.04170700
O	-3.69522600	0.04137300	-0.08279100
O	-2.77630700	-1.86982400	0.26476600

2-CN-5-CH_3-substituted

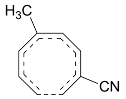

C	0.11146200	1.88144800	-0.75533500
C	1.09686000	0.80063100	1.05320300
C	-1.12730000	1.97320100	-0.18202800
C	-2.02411900	0.89959900	0.06065800
C	-1.87906800	-0.47498200	-0.01944700
C	-0.75013500	-1.33377300	-0.27650600
C	0.62321200	-1.30425700	-0.16677100
C	1.50271900	-0.31312600	0.35861600
H	0.37650000	1.01604800	-1.34911500
H	0.71416400	2.77330100	-0.90214900
H	0.12659100	0.79255300	1.53011600
H	1.82587200	1.51906600	1.41464000
H	-1.44874000	2.94486200	0.19067000
H	-3.01903400	1.22438000	0.36016400
H	-1.09112700	-2.31777200	-0.60245700
H	1.12251900	-2.19459200	-0.54305800
C	2.89828100	-0.43544000	0.01218500
N	4.01874600	-0.53292600	-0.26629700
C	-3.16271100	-1.28199000	0.12963400
H	-4.01779100	-0.64671900	0.36949300
H	-3.38919700	-1.80977500	-0.80460000
H	-3.06619400	-2.03873100	0.91511400

2-CN-5-NH_2-substituted

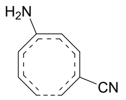

C	0.03083900	1.89719700	-0.77519800
C	1.05746700	0.81149300	1.05030400
C	-1.20571600	1.93492700	-0.19052100
C	-2.05713200	0.83980200	0.09686200
C	-1.87396700	-0.53216400	-0.01708000
C	-0.72436000	-1.35081900	-0.34021800
C	0.64042500	-1.28731800	-0.20312200
C	1.49536500	-0.29009700	0.35820200
H	0.33832600	1.04281200	-1.36443300
H	0.59075800	2.81587800	-0.92212000
H	0.07535300	0.79052400	1.50240700
H	1.76796500	1.54052000	1.42693900
H	-1.55702600	2.89511100	0.18551300
H	-3.04552500	1.12637100	0.45413800
H	-1.05432800	-2.30546200	-0.74902300
H	1.16552400	-2.14704000	-0.61402500
C	2.89701100	-0.38771000	0.03344500

N	4.02353000	-0.46857200	-0.22624000
N	-3.03011500	-1.32389500	0.09011400
H	-3.87282700	-0.83186400	0.35708800
H	-2.92171200	-2.19144100	0.60036000

2-CN-5-OH-substituted

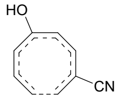

C	0.00946200	1.88766500	-0.76617300
C	1.04998900	0.80794700	1.05983700
C	-1.22448700	1.91767900	-0.18019500
C	-2.07468900	0.81075900	0.07283800
C	-1.86043800	-0.55247700	-0.01483700
C	-0.71991700	-1.39435100	-0.26926200
C	0.64558000	-1.31283100	-0.15664300
C	1.49247500	-0.28611300	0.36125700
H	0.31906100	1.03704400	-1.36007900
H	0.57110100	2.80679100	-0.90302000
H	0.07489000	0.77265000	1.52661300
H	1.75241000	1.55310600	1.41929700
H	-1.58338600	2.87073800	0.20568500
H	-3.08004700	1.08201700	0.39620200
H	-1.06597000	-2.37485500	-0.59002000
H	1.17421300	-2.18753400	-0.52907600
C	2.88908200	-0.35903100	0.00688300
N	4.01012900	-0.41576300	-0.28025800
O	-2.96265800	-1.36559000	0.12800900
H	-3.77425000	-0.84036500	0.08990000

2-CN-5-F-substituted

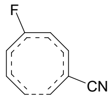

C	-0.02608300	1.89554500	-0.74733600
C	1.03677000	0.79628200	1.07507100
C	-1.26016800	1.91746100	-0.16456100
C	-2.10260800	0.79414700	0.04767200
C	-1.83159200	-0.54902300	-0.02134300
C	-0.71450500	-1.41760700	-0.23447500
C	0.65204000	-1.33073800	-0.13727600
C	1.48591500	-0.28328600	0.36087700
H	0.28626000	1.04972100	-1.34728500
H	0.54064200	2.81381200	-0.86909600
H	0.06444500	0.74845700	1.54671900
H	1.73050700	1.55193400	1.42906100
H	-1.63101600	2.86222400	0.22876200
H	-3.13526600	1.00416900	0.31546800
H	-1.07188700	-2.40363700	-0.52500200
H	1.18575700	-2.20881900	-0.49319500
C	2.87983400	-0.33238400	-0.01135600
N	3.99710100	-0.36988900	-0.31452100
F	-2.96297400	-1.30789100	0.10139800

2-CN-5-CHO-substituted

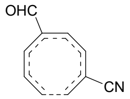

C	0.51086700	1.92170200	-0.76148600
C	1.42447200	0.74017300	1.10469600
C	-0.72875500	2.06661700	-0.20442500
C	-1.67173600	1.02548900	-0.01778000
C	-1.55607300	-0.35052300	-0.11110200
C	-0.48165100	-1.28439500	-0.31754300
C	0.88533800	-1.32064700	-0.17337300
C	1.78999800	-0.35894600	0.37428200
H	0.75369200	1.04131400	-1.34292000
H	1.16692100	2.77947100	-0.88039500
H	0.43795900	0.78022600	1.54505400
H	2.17485800	1.42355300	1.48916000
H	-1.01996700	3.04751500	0.16689000
H	-2.67965000	1.34777200	0.24098200
H	-0.86767400	-2.25060500	-0.64644700
H	1.35676900	-2.23428000	-0.52810800
C	3.18607000	-0.52635000	0.04179200
N	4.30383600	-0.66102800	-0.22933100
C	-2.88439600	-1.05866300	-0.01602300
H	-2.85291200	-2.14384800	-0.24167300
O	-3.93020700	-0.53633300	0.28606900

2-CN-5-CN-substituted

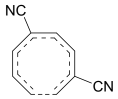

C	0.54649500	1.92742400	-0.73996400
C	1.38930600	0.70777800	1.09540000
C	-0.67218800	2.16622400	-0.16769400
C	-1.68947400	1.20032200	0.03035100
C	-1.66300300	-0.18456500	-0.06409700
C	-0.66158000	-1.20170900	-0.28627900
C	0.70233000	-1.31807000	-0.16255500
C	1.67553600	-0.41733100	0.36642400
H	0.70735000	1.03961100	-1.33865900
H	1.26452100	2.73283000	-0.86402700
H	0.41855500	0.80105200	1.56238400
H	2.18734200	1.34322900	1.46616000
H	-0.88139600	3.16210300	0.21781000
H	-2.66022400	1.60763700	0.30427100
H	-1.12395200	-2.13421900	-0.60460100
H	1.10620200	-2.25939500	-0.52739600
C	3.05183500	-0.67166300	0.00972200
N	4.15425500	-0.87529900	-0.28040100
C	-2.98616400	-0.77460500	0.04663200
N	-4.03667800	-1.25265500	0.13988900

2-CN-5-NO_2-substituted

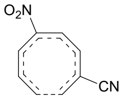

C	0.99564700	1.91324200	-0.86661100
C	1.77133800	0.78563400	1.05866900
C	-0.21429100	2.24372600	-0.32269200
C	-1.27847500	1.34426800	-0.06590800
C	-1.29825500	-0.03248000	-0.07133900
C	-0.37168100	-1.11072300	-0.21953100
C	0.98419200	-1.28285700	-0.06907800
C	1.99882500	-0.39852700	0.40619500
H	1.12152900	0.98142600	-1.40364300
H	1.74842500	2.67748600	-1.03653900
H	0.80708800	0.96245700	1.51451100
H	2.60209600	1.40112500	1.38903100
H	-0.38063500	3.27161500	-0.00719100
H	-2.23034300	1.80125200	0.18529700
H	-0.88568800	-2.02650700	-0.49399800
H	1.34362600	-2.26460900	-0.36749100
C	3.36181800	-0.74395400	0.07444300
N	4.45427000	-1.01629300	-0.19598300
N	-2.71688700	-0.55961800	0.05648000
O	-3.52323400	0.12346600	0.65865500
O	-2.97457800	-1.63382000	-0.45219900

2-NO_2-5-CH_3-substituted

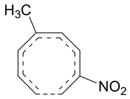

C	-0.28093200	1.89170400	-0.84352000
C	0.73391000	0.91223100	1.01824000
C	-1.51211100	1.97063700	-0.25606500
C	-2.38301300	0.88443600	0.03519900
C	-2.20698500	-0.48707600	0.00821000
C	-1.05832500	-1.32962700	-0.22673500
C	0.31147400	-1.26395000	-0.11361900
C	1.13676400	-0.22132200	0.37579500
H	0.00212100	1.00896500	-1.40327500
H	0.30174300	2.79021200	-1.02479800
H	-0.23163500	0.90025100	1.50362100
H	1.45817000	1.65947100	1.32121000
H	-1.85272500	2.94638800	0.08798900
H	-3.38283800	1.19842100	0.32969500
H	-1.37687300	-2.33108900	-0.51951800
H	0.84851100	-2.14684200	-0.44483200
N	2.57073400	-0.30909500	0.01733000
O	3.25347500	0.69177300	0.16054600
O	2.99380600	-1.37555500	-0.39536100
C	-3.46857500	-1.31831400	0.19986200
H	-4.33499700	-0.69618400	0.43312800
H	-3.69479400	-1.87848500	-0.71536600
H	-3.34331400	-2.04950800	1.00514400

2-NO_2-5-NH_2-substituted

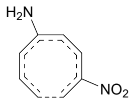

C	-0.43450500	1.96620000	-0.89253500
C	0.74499500	0.96138900	0.99376500
C	-1.65521000	1.92176400	-0.27053200
C	-2.43785000	0.79696600	0.08903300
C	-2.20924800	-0.57827000	0.02491700
C	-1.02896200	-1.36395700	-0.27150000
C	0.33329500	-1.23150300	-0.14752700
C	1.14584900	-0.17671400	0.35110100
H	-0.05406300	1.12094400	-1.44733500
H	0.04255000	2.92211300	-1.08121800
H	-0.22600200	0.98069600	1.46131700
H	1.48047000	1.69745900	1.28962000
H	-2.06562000	2.87158500	0.07191200
H	-3.42827400	1.05623400	0.46221200
H	-1.31681400	-2.35237800	-0.62757600
H	0.90626200	-2.07815900	-0.50901000
N	2.58935200	-0.27670700	0.02594500
N	-3.33306100	-1.39794700	0.18782300
H	-4.19097300	-0.93378000	0.44886600
H	-3.18876200	-2.26633900	0.68338400
O	3.00735100	-1.32977300	-0.46123400
O	3.30477400	0.69589200	0.27262400

2-NO_2-5-OH-substituted

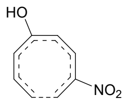

C	-0.37358300	1.88432200	-0.86857200
C	0.69274500	0.91106200	1.02258900
C	-1.59690200	1.91038700	-0.26457100
C	-2.42535600	0.79819200	0.04600300
C	-2.18804700	-0.56233500	0.01959600
C	-1.03181700	-1.39521600	-0.20788700
C	0.32920400	-1.28256600	-0.09607400
C	1.12592300	-0.20723800	0.37902600
H	-0.04800700	1.01262700	-1.42279800
H	0.17029500	2.80683500	-1.04657500
H	-0.27918800	0.88060700	1.49516900
H	1.39395000	1.68176500	1.32029500
H	-1.97000200	2.87108600	0.08766500
H	-3.43291500	1.06683800	0.36453900
H	-1.35979000	-2.39382300	-0.48850800
H	0.89137400	-2.15221300	-0.42013000
N	2.55911600	-0.25408300	0.01272900
O	3.22321300	0.75343400	0.19093700
O	3.00007500	-1.29605100	-0.44200500
O	-3.27067600	-1.38902100	0.21028900
H	-4.09344100	-0.88168100	0.16680200

2-NO_2-5-F-substituted

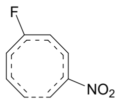

C	-0.40176700	1.89139300	-0.84283900
C	0.67954000	0.89606500	1.04015300
C	-1.62798800	1.90956600	-0.24684900
C	-2.44994100	0.77973400	0.01980800
C	-2.15809300	-0.55957600	0.01050600
C	-1.02628900	-1.41955700	-0.17140800
C	0.33632300	-1.30351900	-0.07413400
C	1.11857500	-0.20984300	0.38153400
H	-0.07427200	1.02607700	-1.40645000
H	0.14848100	2.81369400	-1.00248100
H	-0.28862200	0.85380100	1.51984100
H	1.37305500	1.67571500	1.33250200
H	-2.01291500	2.86158900	0.11437600
H	-3.48423800	0.98498000	0.28491900
H	-1.36658400	-2.42262600	-0.42031500
H	0.90322600	-2.17591600	-0.38149300
N	2.55068500	-0.23505100	-0.00155900
O	3.19682300	0.78597600	0.15897600
O	3.00170100	-1.27370600	-0.45156300
F	-3.27369800	-1.33063200	0.17889800

2-NO_2-5-CHO-substituted

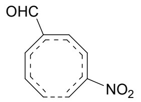

C	0.10769900	1.94972500	-0.84762700
C	1.05551000	0.86913200	1.06406300
C	-1.12713400	2.07927200	-0.27913200
C	-2.04393400	1.02098700	-0.04930800
C	-1.89646200	-0.35346400	-0.09274700
C	-0.80113600	-1.27000600	-0.27136600
C	0.56518400	-1.26826000	-0.12449500
C	1.41527400	-0.25196400	0.38447300
H	0.36627300	1.05495300	-1.40035400
H	0.74451600	2.81685600	-0.99819000
H	0.07533800	0.90144400	1.51811700
H	1.80416100	1.58237600	1.38909000
H	-1.43873500	3.06335100	0.06643000
H	-3.05771600	1.32894400	0.20411700
H	-1.16471500	-2.25606600	-0.56411200
H	1.07421500	-2.17536100	-0.43319100
N	2.85535400	-0.38412300	0.03969300
O	3.56156200	0.59887600	0.18081800
O	3.24715300	-1.46543900	-0.36005400
C	-3.20799900	-1.08900700	0.03731400
H	-3.15182400	-2.18100600	-0.14586100
O	-4.26384000	-0.57907700	0.32411700

2-NO_2-5-CN-substituted

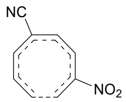

C	0.13725100	1.97947600	-0.81229200
C	1.02113800	0.85711400	1.06808200
C	-1.08292000	2.18845800	-0.23461400
C	-2.06822200	1.19446300	-0.00248700
C	-1.99884000	-0.19026500	-0.05085900
C	-0.96606700	-1.18332200	-0.24322400
C	0.39950200	-1.24957400	-0.11158300
C	1.31097500	-0.28462600	0.38674900
H	0.32620000	1.07933700	-1.38424600
H	0.82994700	2.80297400	-0.95915000
H	0.05599300	0.93600600	1.54824800
H	1.81345600	1.52718700	1.38144500
H	-1.32392900	3.18632700	0.12663900
H	-3.05060600	1.57994700	0.26129600
H	-1.39786800	-2.13959400	-0.53184700
H	0.85261600	-2.18148000	-0.43393600
C	-3.30300200	-0.81668100	0.08415400
N	-4.33909300	-1.32130100	0.19568000
N	2.73459100	-0.49077700	0.01523400
O	3.48829700	0.45993900	0.12892300
O	3.06505600	-1.59449100	-0.37747400

2-NO_2-5-NO_2-substituted

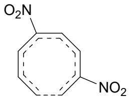

C	0.54887800	2.09979300	-0.88865200
C	1.47515100	0.96588800	1.02017000
C	-0.68520100	2.30993900	-0.32921700
C	-1.68812700	1.34664400	-0.06525700
C	-1.65379400	-0.03548400	-0.06492500
C	-0.67488800	-1.06279700	-0.19901800
C	0.69389900	-1.16256300	-0.05864200
C	1.66390200	-0.23041400	0.38616400
H	0.76589000	1.19531200	-1.43804000
H	1.22272900	2.93479800	-1.05109600
H	0.53688800	1.15555200	1.51573500
H	2.33051900	1.57373100	1.28420700
H	-0.92933100	3.32146900	-0.00995300
H	-2.66212000	1.74931800	0.19029900
H	-1.14602200	-2.00875800	-0.44285000
H	1.11128600	-2.12282800	-0.34004700
N	3.07866800	-0.56841400	0.04051900
N	-3.05671800	-0.62910500	0.07576700
O	3.32221700	-1.71157500	-0.33962100
O	3.92144500	0.31871900	0.16373900
O	-3.95364100	0.09841000	0.49368400
O	-3.22282100	-1.80580300	-0.23355100

5.3.12 2,6-substituted

2-CH_3-6-CH_3-substituted

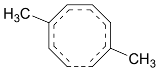

C	0.09858800	-1.51247900	-0.82509600
C	-1.15511800	-0.87990900	0.97564800
C	1.34197400	-1.28541200	-0.29348400
C	1.89876100	-0.00595200	-0.03720500
C	1.25374400	1.22521100	-0.06108900
C	-0.07548700	1.73739300	-0.19863000
C	-1.38366300	1.29207500	-0.09883000
C	-1.92600200	0.05007900	0.32301700
H	-0.39260800	-0.73275200	-1.39393700
H	-0.23918900	-2.52892000	-1.00813400
H	-0.24035400	-0.56705700	1.46223900
H	-1.59005500	-1.81675200	1.31629600
H	-0.03721100	2.80448100	-0.42068000
H	-2.12363100	2.03162300	-0.40727300
C	-3.33920100	-0.27458700	-0.09889400
H	-3.72539200	-1.15059200	0.42890600
H	-4.00903200	0.56852600	0.10315600
H	-3.39157800	-0.47608700	-1.17680300
H	1.95921500	2.05292700	0.01682600
H	1.93462300	-2.14449400	0.02267300
C	3.37968400	-0.00958300	0.29664900
H	3.96685900	-0.46164100	-0.51025300
H	3.76147800	0.99897300	0.47223600
H	3.56718800	-0.59924500	1.20223400

2-CH_3-6-NH_2-substituted

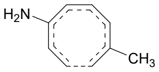

C	0.11238500	-1.50293400	-0.80236600
C	-1.16407400	-0.89859800	0.96459400
C	1.35698100	-1.30067200	-0.26371500
C	1.91782100	-0.02099000	-0.02338800
C	1.28230000	1.22215300	-0.06774200
C	-0.04191000	1.73295600	-0.17308100
C	-1.35686500	1.29453000	-0.08714200
C	-1.91245900	0.05326500	0.31291600
H	-0.35406000	-0.72019400	-1.38867000
H	-0.24277300	-2.51382700	-0.98058100
H	-0.25920300	-0.60518800	1.48048600
H	-1.62313000	-1.82989200	1.28889000
H	-0.00068000	2.80196800	-0.38519100
H	-2.08691300	2.04335800	-0.39523000
C	-3.32399500	-0.25121300	-0.13029200
H	-3.73018500	-1.12428900	0.38730800
H	-3.98476500	0.60043900	0.06566200
H	-3.36269500	-0.44608200	-1.21005800
H	2.00211200	2.04146800	-0.03170100
H	1.92628300	-2.15362100	0.10330300
N	3.27617700	-0.03802000	0.35033800
H	3.85355800	-0.67397800	-0.18638900
H	3.70809700	0.87499200	0.42109000

2-CH_3-6-OH-substituted

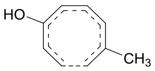

C	0.15398500	-1.52597600	-0.80245000
C	-1.15355600	-0.88834500	0.98120100
C	1.38968900	-1.29135400	-0.26141900
C	1.90801400	0.00381300	-0.01601000
C	1.27968700	1.23945100	-0.03683900
C	-0.04555500	1.74430500	-0.17573600
C	-1.35225800	1.28918700	-0.10578500
C	-1.89962900	0.04390000	0.30563600
H	-0.33438200	-0.75455300	-1.38479400
H	-0.18364400	-2.54483800	-0.96670000
H	-0.24193700	-0.58687100	1.48088200
H	-1.60641700	-1.81843600	1.31687200
H	-0.00858200	2.81221400	-0.39221300
H	-2.08972400	2.02576000	-0.42562800
C	-3.30768400	-0.27308300	-0.13956400
H	-3.69883400	-1.16126400	0.36350100
H	-3.98017500	0.56493200	0.07459200
H	-3.34723500	-0.44816300	-1.22242800
H	2.01591600	2.03803500	0.03996800
H	1.97863500	-2.13264600	0.10537300
O	3.24564300	0.08533600	0.30909000
H	3.69508000	-0.72825000	0.04365400

2-CH_3-6-F-substituted

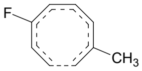

C	0.15621800	-1.56433900	-0.78711900
C	-1.13207900	-0.86975900	0.99018600
C	1.39735500	-1.31452800	-0.26288200
C	1.87667900	-0.01244100	-0.02565200
C	1.28896500	1.23369300	-0.03066200
C	-0.02861200	1.75765500	-0.18446300
C	-1.33256600	1.30044300	-0.11288100
C	-1.87817200	0.05597200	0.30553400
H	-0.34272800	-0.80559400	-1.37636300
H	-0.17314600	-2.58794900	-0.93790900
H	-0.22052500	-0.56483300	1.48809000
H	-1.58579900	-1.79566400	1.33553900
H	0.01691700	2.82400600	-0.40449200
H	-2.07158600	2.03341500	-0.43747500
C	-3.28319800	-0.26787300	-0.14357600
H	-3.67788300	-1.14724700	0.37174000
H	-3.95720200	0.57358500	0.05088300
H	-3.31462700	-0.46228300	-1.22343000
H	2.04200400	2.01413700	0.06563700
H	2.03090200	-2.12702700	0.08408800
F	3.20734900	0.01472400	0.27697600

2-CH_3-6-CHO-substituted

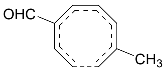

C	0.35222700	1.66914300	-0.82492300
C	1.49072300	0.77120600	1.04216500
C	-0.90071700	1.49079600	-0.31110200
C	-1.53751800	0.23467600	-0.11353800
C	-1.03029800	-1.05703400	-0.16075800
C	0.23935000	-1.69096900	-0.29488300
C	1.57311000	-1.34867700	-0.15045100
C	2.19772400	-0.17194200	0.34493700
H	0.82597600	0.88010900	-1.39479700
H	0.76066600	2.66861300	-0.94394300
H	0.52551000	0.52128500	1.46450000
H	1.98514700	1.65380300	1.44042400
H	0.11482400	-2.74270900	-0.55244100
H	2.26475600	-2.12800000	-0.47302600
C	3.64654500	0.05234300	-0.01739700
H	4.07293400	0.89251000	0.53651300
H	4.24382700	-0.83922000	0.20294900
H	3.75785200	0.25901700	-1.08925400
H	-1.83673700	-1.78943400	-0.10860300
H	-1.44936400	2.36858300	0.03239400
C	-2.99892700	0.33223900	0.18124900
H	-3.37825300	1.37022900	0.28647900
O	-3.76005500	-0.60068400	0.30337700

2-CH_3-6-CN-substituted

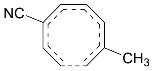

C	-0.16701200	-1.55421600	-0.86686500
C	-1.35436700	-0.84745900	1.01747500
C	1.07764000	-1.31999000	-0.35450500
C	1.61178300	-0.01924900	-0.12839600
C	1.00260200	1.23240700	-0.13498900
C	-0.32199600	1.75335800	-0.23824900
C	-1.62158100	1.29922700	-0.09589100
C	-2.14364400	0.05734700	0.35669300
H	-0.68304200	-0.77821500	-1.41760900
H	-0.51203500	-2.57259400	-1.01869100
H	-0.41819300	-0.52736500	1.45749400
H	-1.76937900	-1.78275700	1.38470500
H	-0.28692700	2.81851000	-0.46475100
H	-2.37581100	2.03106100	-0.38728300
C	-3.56756900	-0.27852100	-0.01647100
H	-3.92276600	-1.16481800	0.51505000
H	-4.23769400	0.55440100	0.22249200
H	-3.65816000	-0.46691900	-1.09372100
H	1.73136700	2.03914500	-0.07407200
H	1.69060700	-2.15939200	-0.03418400
C	3.03114100	0.00413000	0.15698600
N	4.16572200	0.00667800	0.39083600

2-CH_3-6-NO_2-substituted

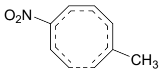

C	-0.53791200	-1.54638500	-0.88305600
C	-1.70789000	-0.84513600	1.01499800
C	0.71004200	-1.32213200	-0.37520500
C	1.22539300	-0.02036500	-0.16513900
C	0.63568700	1.22775100	-0.16157800
C	-0.68821800	1.75261100	-0.24910200
C	-1.98665300	1.30016300	-0.09425600
C	-2.50445100	0.05736100	0.36007900
H	-1.05503800	-0.77151800	-1.43448400
H	-0.89113000	-2.56347600	-1.02332100
H	-0.77119500	-0.52221100	1.45180400
H	-2.11602900	-1.78382300	1.38113800
H	-0.65264200	2.81732400	-0.47573100
H	-2.74286200	2.03332100	-0.37719500
C	-3.92951000	-0.28195500	-0.00512000
H	-4.28023500	-1.16732500	0.53079400
H	-4.59980400	0.55044300	0.23501700
H	-4.02530900	-0.47371100	-1.08123800
H	1.37657400	2.02053400	-0.11230800
H	1.32160900	-2.15510100	-0.04780600
N	2.69984200	0.00377900	0.11193400
O	3.26334400	1.07804500	0.22319600
O	3.26643500	-1.07084200	0.21681300

2- NH_2-6-CH_3-substituted

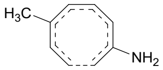

C	0.12319200	-1.52323700	-0.84998200
C	-1.21565700	-0.90558000	0.99464000
C	1.34883400	-1.28388200	-0.29957900
C	1.88992700	0.00724700	-0.03177900
C	1.23606600	1.22759500	-0.04212300
C	-0.10341400	1.72714600	-0.18915200
C	-1.40382300	1.28310100	-0.09475600
C	-1.94785600	0.02228400	0.30449400
H	-0.39686500	-0.74689700	-1.39733400
H	-0.21344500	-2.54123200	-1.02576200
H	-0.31626500	-0.59214700	1.50543700
H	-1.65586400	-1.85512600	1.28870500
H	-0.07222800	2.79575300	-0.40506600
H	-2.14927600	2.02651800	-0.37990600
H	1.92828300	2.06366200	0.05767100
H	1.94933200	-2.13515900	0.02213000
N	-3.22711200	-0.29941000	-0.17698100
H	-3.80732300	-0.82521900	0.46478800
H	-3.73549500	0.47364400	-0.58726100
C	3.37103000	0.01483300	0.29745100
H	3.95893800	-0.41323700	-0.52218900
H	3.74217800	1.02336400	0.49484600
H	3.56801400	-0.59509300	1.18752200

2- NH_2-6-NH_2-substituted

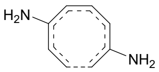

C	0.13885900	-1.51067100	-0.82985100
C	-1.23021900	-0.92542000	0.98421300
C	1.36280600	-1.29858400	-0.26785400
C	1.91051200	-0.00633500	-0.01868900
C	1.26630300	1.22644600	-0.05172500
C	-0.06913700	1.72335000	-0.16047800
C	-1.37588000	1.28704000	-0.07839200
C	-1.93501400	0.02476800	0.29331400
H	-0.35387900	-0.72765100	-1.39375500
H	-0.22134700	-2.52137000	-0.99763300
H	-0.34132400	-0.63295500	1.52487400
H	-1.69371800	-1.87079800	1.25541900
H	-0.03588900	2.79485300	-0.36126600
H	-2.11220000	2.04279500	-0.35288700
H	1.97400500	2.05476200	0.00179800
H	1.93456800	-2.14456800	0.11089400
N	-3.20923400	-0.27574400	-0.21266600
H	-3.80637400	-0.80352900	0.41168400
H	-3.70037400	0.50681800	-0.62571600
N	3.26670500	-0.01719100	0.34962600
H	3.84397100	-0.66637300	-0.17076100
H	3.70088200	0.89499900	0.41539800

2- NH_2-6-OH-substituted

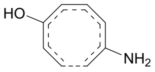

C	0.15068600	-1.53280700	-0.82957300
C	-1.21008200	-0.90920100	0.99666200
C	1.38054500	-1.31511100	-0.28331200
C	1.90100300	-0.01991800	-0.02469000
C	1.27668600	1.21672700	-0.04439500
C	-0.05349400	1.72639600	-0.17761700
C	-1.35788500	1.29124600	-0.09149800
C	-1.91650900	0.03174500	0.29649600
H	-0.35558200	-0.75237100	-1.38402500
H	-0.20415200	-2.54558500	-0.99551700
H	-0.31412000	-0.61347700	1.52357500
H	-1.67242000	-1.85029900	1.28367500
H	-0.01236400	2.79495300	-0.39041900
H	-2.09457500	2.04326500	-0.37478300
H	1.99531400	2.03620000	0.02254000
H	1.99041900	-2.14593500	0.06213900
N	-3.19115600	-0.27204600	-0.20318800
H	-3.78416300	-0.80480000	0.42073300
H	-3.68737700	0.50624900	-0.61795200
O	3.23935800	-0.06308800	0.27965100
H	3.53654700	0.80636900	0.58271100

2- NH_2-6-F-substituted

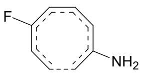

C	0.18163800	-1.57911000	-0.81040900
C	-1.19200300	-0.88569300	1.01982500
C	1.40356200	-1.31255700	-0.26866300
C	1.86546200	0.00216800	-0.02316800
C	1.26829300	1.23717200	-0.01518700
C	-0.06027000	1.75024500	-0.17517000
C	-1.35525100	1.29467600	-0.10632300
C	-1.89952000	0.02848800	0.28953800
H	-0.35327000	-0.82516300	-1.37377700
H	-0.14492400	-2.60517000	-0.94994400
H	-0.29708500	-0.57242400	1.53903400
H	-1.64424200	-1.82669700	1.32222400
H	-0.02155300	2.81787500	-0.39017400
H	-2.10169400	2.03122300	-0.40495000
H	2.00939900	2.02600500	0.09879900
H	2.04519300	-2.11450300	0.08728000
N	-3.15917500	-0.30070400	-0.22922900
H	-3.75021000	-0.84931500	0.38273700
H	-3.66653200	0.46619200	-0.65169500
F	3.19640800	0.03828600	0.27582400

2- NH_2-6-CHO-substituted

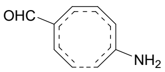

C	0.33998300	1.69093800	-0.84528600
C	1.54224300	0.76524600	1.08285800
C	-0.90087000	1.49885000	-0.32342000
C	-1.52648700	0.23155700	-0.11512100
C	-1.01446800	-1.05205300	-0.14684600
C	0.26357000	-1.68197600	-0.29676300
C	1.59016800	-1.34673600	-0.15492900
C	2.21817600	-0.15648700	0.33588800
H	0.84986300	0.90349100	-1.38531700
H	0.74500600	2.69275300	-0.95587200
H	0.58642600	0.50200200	1.51433300
H	2.03739000	1.65755000	1.45637900
H	0.13916000	-2.73254900	-0.55929700
H	2.28407400	-2.12892300	-0.46535100
H	-1.81206500	-1.79150700	-0.06975900
H	-1.46039700	2.37049000	0.01751300
N	3.53007200	0.09063200	-0.09447100
H	4.12353600	0.55673400	0.58041500
H	4.00022700	-0.69992900	-0.51681200
C	-2.99097700	0.32022100	0.17666700
H	-3.37985800	1.35604700	0.26525300
O	-3.74398500	-0.61724200	0.31268900

2-NH_2-6-CN-substituted

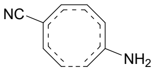

C	-0.14625600	-1.57386500	-0.89301100
C	-1.41115900	-0.85371700	1.05845000
C	1.08235500	-1.32511000	-0.36698600
C	1.60063600	-0.01286800	-0.12630600
C	0.98510400	1.22843700	-0.11740900

C	-0.34928800	1.74185200	-0.23784200
C	-1.64081100	1.29246000	-0.09857600
C	-2.16519000	0.03489800	0.34617500
H	-0.69915100	-0.80268800	-1.41399700
H	-0.48696500	-2.59482900	-1.03680300
H	-0.48485900	-0.52421600	1.50845800
H	-1.82744200	-1.79822300	1.39825900
H	-0.31686700	2.80643700	-0.46749000
H	-2.39911100	2.02596500	-0.37463500
H	1.70258300	2.04227800	-0.02899800
H	1.70649500	-2.15596700	-0.04669100
C	3.02056700	0.02158800	0.15829200
N	4.15510300	0.03094200	0.39211000
N	-3.44971300	-0.30585000	-0.09599000
H	-4.00404500	-0.84750400	0.55511600
H	-3.98410800	0.45106400	-0.50278700

2-NH_2-6-NO_2-substituted

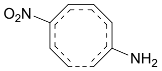

C	-0.51786000	-1.57205700	-0.89990300
C	-1.76606000	-0.84536800	1.06210200
C	0.71464200	-1.33403000	-0.37870600
C	1.21541900	-0.02050900	-0.16238700
C	0.61993900	1.21759400	-0.15157100
C	-0.71339900	1.73645900	-0.25865000
C	-2.00449800	1.29215100	-0.10406900
C	-2.52665200	0.03681700	0.34913800
H	-1.06783800	-0.80262500	-1.42686200
H	-0.87131700	-2.59096000	-1.02526000
H	-0.83860200	-0.51063600	1.50578800
H	-2.17611400	-1.79048500	1.40761700
H	-0.67849700	2.79918800	-0.49457000
H	-2.76335500	2.02693400	-0.37528000
H	1.35120700	2.01725300	-0.08086700
H	1.33330700	-2.15859200	-0.04461900
N	-3.81261600	-0.30730300	-0.08352700
H	-4.36364100	-0.84706900	0.57183000
H	-4.34981900	0.44464100	-0.49564400
N	2.69298300	0.01823200	0.11273900
O	3.24271400	1.09682000	0.24945500
O	3.27390000	-1.05063100	0.19025100

2-OH-6-CH_3-substituted

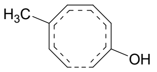

C	0.10965200	-1.51642500	-0.85857800
C	-1.21300600	-0.93387300	0.93157600
C	1.34178100	-1.27914100	-0.30741900
C	1.87374700	0.00540700	-0.02254000
C	1.21809700	1.23072300	-0.04449100
C	-0.11301700	1.73089800	-0.20814200
C	-1.41732500	1.28297700	-0.12672600
C	-1.94010300	0.03391100	0.28901900
H	-0.39046100	-0.73974400	-1.42399800
H	-0.21002700	-2.53551400	-1.05915300

H	-0.31560000	-0.63925800	1.45624400
H	-1.67763700	-1.86917800	1.23664300
H	-0.08113700	2.79859900	-0.42693100
H	-2.17980700	1.99675400	-0.43097300
H	1.91271000	2.06447500	0.05750400
H	1.94330300	-2.13312200	0.00466200
O	-3.23070700	-0.18258500	-0.12513800
H	-3.60275700	-0.93664600	0.35307500
C	3.34859800	0.01692300	0.33634300
H	3.95545300	-0.39676100	-0.47663200
H	3.70952900	1.02492500	0.55362800
H	3.53153700	-0.60223900	1.22278500

2-OH-6-NH_2-substituted

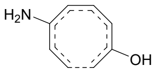

C	0.12713200	-1.50413600	-0.84146600
C	-1.22636900	-0.95229800	0.91855200
C	1.35730400	-1.29446300	-0.27796100
C	1.89504800	-0.00899000	-0.00722000
C	1.24887000	1.22887300	-0.05319000
C	-0.07789700	1.72611400	-0.18432500
C	-1.38909200	1.28604800	-0.11614700
C	-1.92740100	0.03790800	0.27688600
H	-0.34747900	-0.72037500	-1.41996300
H	-0.21262300	-2.51676500	-1.03895000
H	-0.33893300	-0.67998400	1.47137500
H	-1.71620400	-1.88118000	1.20363900
H	-0.04357600	2.79603000	-0.39167400
H	-2.14150100	2.01143300	-0.41631600
H	1.95882400	2.05488700	0.00524300
H	1.93083300	-2.14486800	0.08780900
O	-3.21616500	-0.15851300	-0.15306600
H	-3.60056100	-0.91347300	0.31394000
N	3.24132500	-0.01292000	0.39366700
H	3.83505600	-0.66201100	-0.10757200
H	3.67064000	0.90052300	0.47054100

2-OH-6-OH-substituted

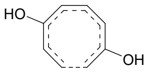

C	0.13900800	-1.52536400	-0.84005400
C	-1.20689900	-0.93877700	0.92958300
C	1.37427600	-1.31024100	-0.29015900
C	1.88547400	-0.02189800	-0.01256600
C	1.25945900	1.21976700	-0.04598800
C	-0.06214300	1.72928200	-0.19905400
C	-1.37078800	1.28984600	-0.12732200
C	-1.90872600	0.04374400	0.27926600
H	-0.34624100	-0.74472400	-1.41305300
H	-0.19748500	-2.53975500	-1.03331900
H	-0.31315100	-0.66327200	1.47034000
H	-1.69545600	-1.86377800	1.22851000

H	-0.02036500	2.79648400	-0.41700900
H	-2.12404700	2.01160000	-0.43394000
H	1.98070900	2.03674300	0.02265600
H	1.98426500	-2.14432000	0.04694000
O	-3.19716600	-0.15385600	-0.14723200
H	-3.58176400	-0.90845800	0.32036200
O	3.21663200	-0.05924200	0.32190600
H	3.49984400	0.80611000	0.64888900

2-OH-6-F-substituted

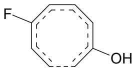

C	0.17064800	-1.56850300	-0.82195600
C	-1.18939800	-0.92090100	0.94949800
C	1.39772200	-1.30643900	-0.27478100
C	1.85144600	0.00101700	-0.01056800
C	1.25137800	1.24054700	-0.01368000
C	-0.06879400	1.75190200	-0.19407300
C	-1.36751100	1.29128800	-0.14132500
C	-1.89250000	0.03815800	0.27091400
H	-0.33892000	-0.81344300	-1.40707600
H	-0.13992100	-2.59560900	-0.98885200
H	-0.29616200	-0.62930400	1.48330800
H	-1.66885400	-1.84629100	1.26090600
H	-0.03027200	2.81879100	-0.41153500
H	-2.12994900	1.99763700	-0.46155300
H	1.99301900	2.02800800	0.10655300
H	2.03821600	-2.11254800	0.07349100
O	-3.16973400	-0.18085600	-0.17538300
H	-3.55318200	-0.93859400	0.28830500
F	3.17399400	0.04175400	0.31948300

2-OH-6-CHO-substituted

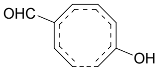

C	0.35789700	1.67790600	-0.85078700
C	1.54290000	0.80656300	1.01417400
C	-0.89286600	1.49339500	-0.33392200
C	-1.51362900	0.23269100	-0.11546800
C	-1.00242100	-1.05651100	-0.15864700
C	0.26881600	-1.68529700	-0.31559800
C	1.59993500	-1.34680000	-0.18115400
C	2.20743400	-0.16716600	0.32214600
H	0.84120900	0.89190700	-1.41692300
H	0.75532100	2.68064400	-0.98029700
H	0.58772800	0.56818500	1.46034700
H	2.06448200	1.68186400	1.39428000
H	0.14554700	-2.73570900	-0.57826300
H	2.31441600	-2.10215100	-0.50178100
H	-1.80293900	-1.79308400	-0.08523100
H	-1.44977500	2.36893200	0.00100800
O	3.52317200	-0.04005100	-0.04036800
H	3.94448000	0.65368600	0.48625700
C	-2.97411900	0.32281200	0.19806700

H	-3.36160100	1.35887200	0.28937200
O	-3.72349200	-0.61478700	0.34766400

2-OH-6-CN-substituted

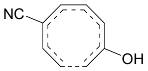

C	-0.16358300	-1.56208800	-0.90052500
C	-1.40634600	-0.89624500	0.98638200
C	1.07524100	-1.31786800	-0.38009400
C	1.58753600	-0.01292700	-0.12688300
C	0.97001800	1.23352200	-0.12582100
C	-0.35802200	1.74523900	-0.25162800
C	-1.65333000	1.29049000	-0.12115300
C	-2.15593000	0.04282400	0.33324800
H	-0.69333700	-0.79017300	-1.44408300
H	-0.49189300	-2.58376600	-1.06749400
H	-0.48046600	-0.58961200	1.45236200
H	-1.84777100	-1.82846100	1.33096900
H	-0.32802200	2.81007100	-0.47938000
H	-2.43074300	1.99535400	-0.40806800
H	1.68994400	2.04546600	-0.04029700
H	1.69904900	-2.15248900	-0.06931000
C	3.00308300	0.02169600	0.17986700
N	4.13361400	0.03219800	0.43160200
O	-3.45367500	-0.18344300	-0.03909000
H	-3.81466300	-0.93209800	0.45645800

2-OH-6-NO_2-substituted

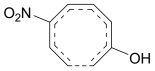

C	-0.53626600	-1.55078300	-0.92596200
C	-1.75637100	-0.89885500	0.97959000
C	0.70790300	-1.32166100	-0.41170000
C	1.20356600	-0.01804300	-0.16989400
C	0.60758200	1.22547400	-0.15218700
C	-0.71991200	1.74346300	-0.25681200
C	-2.01449700	1.29193900	-0.11299200
C	-2.51391900	0.04201200	0.33863000
H	-1.06639000	-0.77581000	-1.46492600
H	-0.87483600	-2.56991200	-1.08707600
H	-0.82800300	-0.59266100	1.44085000
H	-2.19140100	-1.83519600	1.32101300
H	-0.68814800	2.80880200	-0.48016900
H	-2.79328300	2.00059200	-0.38680000
H	1.34145600	2.02237600	-0.07761100
H	1.32908200	-2.15260300	-0.09875000
O	-3.81396500	-0.18302200	-0.02431700
H	-4.17195400	-0.93349700	0.47073600
N	2.67775300	0.01547900	0.12895900
O	3.24441000	-1.05526000	0.26198700
O	3.23589100	1.09306600	0.22832800

2-F-6-CH_3-substituted

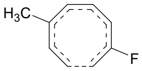

C	0.10703300	-1.52183000	-0.87122700
C	-1.22235000	-0.97959300	0.92277500
C	1.33248200	-1.26985600	-0.30922400
C	1.85716200	0.01566100	-0.02204400
C	1.19966400	1.24197200	-0.05310200
C	-0.12955500	1.73596200	-0.21587800
C	-1.42939900	1.26781700	-0.13170800
C	-1.91193700	0.02025700	0.30252300
H	-0.39640900	-0.74941300	-1.43917900
H	-0.19812700	-2.54407800	-1.07659700
H	-0.31907600	-0.72226100	1.45571600
H	-1.73883200	-1.89335600	1.19792200
H	-0.10919200	2.80330100	-0.43587600
H	-2.20749800	1.96378800	-0.43643700
H	1.89358300	2.07691000	0.04247000
H	1.93887400	-2.11944100	0.00573100
F	-3.19863800	-0.23827400	-0.05600500
C	3.32875100	0.03187300	0.35044800
H	3.94255000	-0.40145800	-0.44660400
H	3.68986000	1.04362900	0.54856400
H	3.50089500	-0.56671700	1.25296200

2-F-6-NH_2-substituted

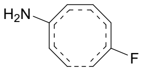

C	0.12240000	-1.50738600	-0.85177000
C	-1.23886000	-1.00311700	0.90306100
C	1.34785400	-1.28512000	-0.27879600
C	1.88035900	-0.00011300	-0.00578400
C	1.23260800	1.24017200	-0.06227900
C	-0.09147100	1.73074700	-0.19008200
C	-1.39954500	1.27055800	-0.11867300
C	-1.89958000	0.02380200	0.28990500
H	-0.35278300	-0.72738000	-1.43453600
H	-0.20275500	-2.52309500	-1.05642700
H	-0.34869600	-0.77332400	1.46935200
H	-1.78374300	-1.90712800	1.15481400
H	-0.06909600	2.80061400	-0.39734300
H	-2.16692200	1.97967200	-0.41858300
H	1.94232300	2.06674900	-0.01227000
H	1.92527200	-2.13281200	0.08743900
F	-3.18739000	-0.20902800	-0.08490300
N	3.22102700	0.00253200	0.40611000
H	3.82392500	-0.65254100	-0.07543000
H	3.64920600	0.91551200	0.49084400

2-F-6-OH-substituted

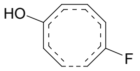

C	0.16472100	-1.53195900	-0.84911000
C	-1.22357700	-0.98840300	0.92606900
C	1.38026200	-1.27468500	-0.27466700
C	1.86791200	0.02607000	0.00212900
C	1.22580800	1.25714300	-0.02638500
C	-0.09915900	1.74266800	-0.19101200
C	-1.39752000	1.26474700	-0.13901900
C	-1.88654300	0.01366900	0.28285900
H	-0.33408900	-0.76613100	-1.42980700
H	-0.14180400	-2.55636900	-1.03693300
H	-0.32536800	-0.74213100	1.47290100
H	-1.75728700	-1.89423400	1.19390300
H	-0.08051500	2.81138800	-0.40261600
H	-2.17253400	1.95917200	-0.45359000
H	1.95051300	2.06354800	0.06962600
H	1.97967000	-2.10866200	0.09148400
F	-3.16652500	-0.23858700	-0.10182700
O	3.19142100	0.12684800	0.36141600
H	3.65734500	-0.68957600	0.13495300

2-F-6-F-substituted

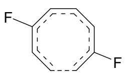

C	0.16954800	-1.57351900	-0.83431800
C	-1.19718700	-0.96705800	0.93787900
C	1.38983400	-1.29618300	-0.27652000
C	1.83474600	0.01182300	-0.00773100
C	1.23188700	1.25260000	-0.01868900
C	-0.08596500	1.75678900	-0.20240000
C	-1.38017200	1.27476800	-0.15009200
C	-1.86427000	0.02406700	0.28255400
H	-0.34247000	-0.82349500	-1.42326900
H	-0.12570400	-2.60395600	-1.00673500
H	-0.29864500	-0.71218000	1.48041300
H	-1.72800100	-1.87071800	1.21844600
H	-0.05914700	2.82354400	-0.42051700
H	-2.15794900	1.96207000	-0.47379600
H	1.97211300	2.04156500	0.10012900
H	2.03634600	-2.09716300	0.07285900
F	-3.13999200	-0.23841800	-0.10502500
F	3.15254000	0.05848500	0.33484400

2-F-6-CHO-substituted

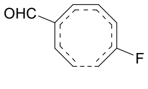

C	0.36474000	1.68483000	-0.86327800
C	1.56211500	0.85005200	1.00902000
C	-0.87987000	1.48838400	-0.33411900
C	-1.49824100	0.22772700	-0.11446000
C	-0.99134100	-1.06414700	-0.16633800
C	0.27707100	-1.69147400	-0.32306100
C	1.60586500	-1.33730100	-0.18445100
C	2.17772700	-0.15860600	0.33538800

H	0.84912000	0.90233700	-1.43297300
H	0.75229300	2.69068100	-0.99603900
H	0.60264900	0.65884500	1.46696200
H	2.14243300	1.69840400	1.35626000
H	0.16161500	-2.74180100	-0.58819500
H	2.33501000	-2.07830900	-0.50482700
H	-1.79432400	-1.79866200	-0.09935200
H	-1.43813000	2.36123500	0.00594900
F	3.49156600	0.00236400	0.03115300
C	-2.95848900	0.31789600	0.20697300
H	-3.34493800	1.35303600	0.30870600
O	-3.70591000	-0.62140100	0.35113500

2-F-6-CN-substituted

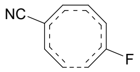

C	-0.16856000	-1.57009100	-0.91050100
C	-1.42023100	-0.94404600	0.97788000
C	1.06382000	-1.31154600	-0.37898400
C	1.57181200	-0.00590000	-0.12694500
C	0.95539300	1.24287400	-0.13413600
C	-0.37027100	1.75101800	-0.25664900
C	-1.66151700	1.27832600	-0.12318000
C	-2.12608200	0.03311000	0.34573700
H	-0.70030100	-0.80395400	-1.45996400
H	-0.48398700	-2.59521800	-1.07962600
H	-0.48843000	-0.68352400	1.45849300
H	-1.92242600	-1.85460800	1.28775700
H	-0.35016800	2.81564700	-0.48478100
H	-2.45329400	1.96663400	-0.40983200
H	1.67654900	2.05450300	-0.05698900
H	1.69076400	-2.14211800	-0.06315600
C	2.98636200	0.03069700	0.18493500
N	4.11605000	0.04451100	0.43951400
F	-3.41838000	-0.23284400	0.02917300

2-F-6-NO_2-substituted

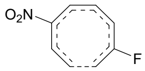

C	0.54134400	1.54962700	-0.95569400
C	1.76840400	0.95679300	0.96041400
C	-0.69647900	1.31229300	-0.42728500
C	-1.18911200	0.01118600	-0.17252100
C	-0.59676400	-1.23531700	-0.14917400
C	0.72802700	-1.75223600	-0.24451300
C	2.01857900	-1.28236300	-0.10139400
C	2.48076600	-0.03047400	0.35219800
H	1.07406900	0.77405200	-1.49088400
H	0.86683100	2.57042000	-1.13120800
H	0.83211300	0.70533600	1.43705700
H	2.26655400	1.87369500	1.25794800
H	0.70522900	-2.81966800	-0.45715400
H	2.81155300	-1.97804300	-0.36641600
H	-1.33321500	-2.02999100	-0.07501100

H	-1.32085900	2.14256700	-0.11791500
F	3.77609800	0.22891000	0.04492900
N	-2.66309100	-0.01885500	0.13360000
O	-3.23130100	-1.09394200	0.18523600
O	-3.21546200	1.05098900	0.31874400

2-CHO-6-CH_3-substituted

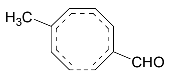

C	0.26585800	-1.42614700	-0.92933500
C	-0.97821400	-0.71119900	0.96309000
C	1.48310400	-1.33407500	-0.32334900
C	2.18024900	-0.12345300	-0.02054500
C	1.68698900	1.16740000	-0.03860200
C	0.42246900	1.82727200	-0.21963400
C	-0.91558100	1.52050700	-0.11326700
C	-1.57902400	0.33417300	0.31425900
H	-0.15090400	-0.57596400	-1.45498900
H	-0.17782500	-2.39563800	-1.13609600
H	-0.02882500	-0.57096500	1.46096000
H	-1.57134000	-1.58300400	1.22669100
H	0.58062600	2.87704300	-0.46724300
H	-1.58336500	2.32783900	-0.41648200
C	-3.00895000	0.19839300	-0.06734500
H	2.47531100	1.91069600	0.08263000
H	1.96499400	-2.25609000	0.00438700
C	3.63807800	-0.31409700	0.35256100
H	-3.47775100	1.11709500	-0.47793100
O	-3.65580900	-0.82008700	0.03874900
H	4.18866400	-0.81824100	-0.44910500
H	4.13297900	0.63685400	0.56221300
H	3.72404800	-0.94157800	1.24797200

2-CHO-6-NH_2-substituted

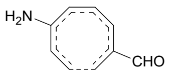

C	0.29141000	-1.42054400	-0.90750000
C	-0.99606700	-0.73406700	0.96450800
C	1.51119700	-1.35186300	-0.30634000
C	2.20632300	-0.13879000	-0.01363600
C	1.71404100	1.16197300	-0.05495100
C	0.45244900	1.81219300	-0.19857000
C	-0.89241300	1.50887000	-0.10089600
C	-1.56830900	0.32762700	0.31471200
H	-0.10948100	-0.56319600	-1.43416400
H	-0.16924300	-2.38223000	-1.11102900
H	-0.05022800	-0.61772600	1.47457000
H	-1.61152400	-1.59280000	1.21968100
H	0.60729500	2.86501400	-0.43536200
H	-1.55117600	2.32337900	-0.40279600
C	-2.99881300	0.21843100	-0.08021500
H	2.52223500	1.89237400	0.00052600
H	1.97752200	-2.27508800	0.03777900
H	-3.44662700	1.14689900	-0.49218900

O	-3.66533000	-0.78834100	0.01437200
N	3.57009400	-0.27809800	0.27718200
H	3.89265200	-1.22266900	0.43790300
H	3.95165200	0.37648100	0.94715100

2-CHO-6-OH-substituted

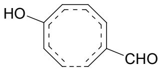

C	0.27705200	-1.44534400	-0.88438000
C	-0.97219000	-0.71800500	0.96327600
C	1.50648700	-1.37707500	-0.29928000
C	2.18706900	-0.16184800	-0.01355500
C	1.72862600	1.14427400	-0.04200300
C	0.47769900	1.81564800	-0.20648100
C	-0.86668800	1.52009000	-0.10928100
C	-1.54641300	0.34300400	0.31153200
H	-0.11994700	-0.59479900	-1.42430500
H	-0.18436200	-2.40815800	-1.08064800
H	-0.03157600	-0.59636600	1.48183800
H	-1.58927700	-1.57382700	1.22431600
H	0.64695700	2.86419300	-0.45044800
H	-1.52162800	2.33519000	-0.41797300
C	-2.97615700	0.23240300	-0.08433800
H	2.54516400	1.86475300	0.04223100
H	2.00440800	-2.28117000	0.04359200
H	-3.42605300	1.16084900	-0.49383300
O	-3.63923900	-0.77654100	0.00818200
O	3.49837500	-0.38826100	0.32026700
H	3.91031700	0.42887000	0.63468800

2-CHO-6-F-substituted

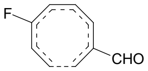

C	0.29466200	-1.49763900	-0.87042300
C	-0.95913300	-0.68948700	0.98455400
C	1.52032500	-1.38474500	-0.28548500
C	2.15181800	-0.14611400	-0.01199200
C	1.72741200	1.15808200	-0.01500100
C	0.48334900	1.83858400	-0.20982000
C	-0.85606800	1.53107200	-0.12741800
C	-1.53068900	0.35194000	0.30419500
H	-0.13080900	-0.66861200	-1.42093200
H	-0.14540900	-2.47523500	-1.03994100
H	-0.01310600	-0.56022700	1.49213900
H	-1.57428100	-1.54212300	1.25992600
H	0.65692500	2.88499400	-0.45753200
H	-1.51705300	2.33597500	-0.45036700
C	-2.95806700	0.22433800	-0.10015300
H	2.56503000	1.84123500	0.11671500
H	2.04782800	-2.26698000	0.06977900
H	-3.41464700	1.14317100	-0.52315100
O	-3.60981800	-0.79043800	0.00078900
F	3.46249000	-0.29832100	0.32625700

2-CHO-6-CHO-substituted

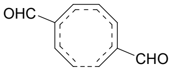

C	-0.13367700	-1.57340100	-0.96417700
C	-1.28338100	-0.63427700	1.01832700
C	1.08246100	-1.47943500	-0.36780300
C	1.81159500	-0.27276100	-0.11303400
C	1.41654400	1.04893100	-0.14905200
C	0.19807400	1.78070100	-0.33232400
C	-1.14611800	1.53114600	-0.18462800
C	-1.84592600	0.39172700	0.31596500
H	-0.56706500	-0.72966300	-1.48593300
H	-0.60262600	-2.54053400	-1.11830000
H	-0.29901600	-0.53437400	1.45557300
H	-1.90802400	-1.46384900	1.33941700
H	0.40410200	2.81233800	-0.61561400
H	-1.79094600	2.35201300	-0.50151000
C	-3.29538200	0.30068800	-0.01753700
H	2.27309600	1.71678000	-0.04614000
H	1.55302900	-2.39911600	-0.01736800
C	3.25221800	-0.49453300	0.22951900
H	-3.73883500	1.21537100	-0.46254500
O	-3.97596400	-0.68264200	0.16421700
H	3.54578100	-1.56047100	0.32230200
O	4.07497100	0.37499000	0.40060700

2-CHO-6-CN-substituted

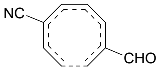

C	0.02666800	-1.43556200	-0.99257200
C	-1.16301200	-0.69263200	0.99173100
C	1.24401400	-1.31279200	-0.40202200
C	1.89592700	-0.06627600	-0.11991800
C	1.41543100	1.23192400	-0.10863100
C	0.14251400	1.87590400	-0.25049600
C	-1.18128400	1.52983800	-0.10755100
C	-1.79924700	0.32485700	0.33788200
H	-0.43307100	-0.59625700	-1.49865300
H	-0.40004800	-2.41647100	-1.17757500
H	-0.19767800	-0.53732400	1.45451300
H	-1.72814200	-1.57804600	1.27133300
H	0.27506900	2.92950300	-0.49241300
H	-1.88026000	2.31830100	-0.38945300
C	-3.23717400	0.14455900	-0.01010700
H	2.21176100	1.96636300	0.00138500
H	1.76814900	-2.20881700	-0.07625100
C	3.30249900	-0.19336100	0.19895400
N	4.42530400	-0.31303200	0.45646800
H	-3.74681900	1.04653500	-0.40708500
O	-3.84051200	-0.89641400	0.11191300

2-CHO-6-NO_2-substituted

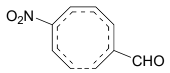

C	-0.33899800	-1.39924500	-1.03029700
C	-1.51668600	-0.69170100	0.98055600
C	0.88013800	-1.28362000	-0.44271800
C	1.50338700	-0.03064600	-0.16803400
C	1.03242800	1.25759900	-0.13167000
C	-0.24506400	1.89790500	-0.24894800
C	-1.56391300	1.54174000	-0.09352000
C	-2.16778500	0.32597100	0.34284400
H	-0.80534800	-0.55817000	-1.52769800
H	-0.76806200	-2.37933500	-1.21377200
H	-0.54979800	-0.53343100	1.43918600
H	-2.06978200	-1.58757000	1.25097700
H	-0.12007300	2.95444700	-0.48014100
H	-2.27156400	2.32932600	-0.35573600
C	-3.60683400	0.13563500	0.00384900
H	1.83698700	1.98039800	-0.02738700
H	1.40992800	-2.17211200	-0.11723800
H	-4.12916200	1.03762100	-0.37594100
O	-4.19748500	-0.91345800	0.11635200
N	2.96867000	-0.14857700	0.14281100
O	3.63710900	0.86566200	0.22188800
O	3.41364400	-1.27132500	0.30372300

2-CN-6-CH_3-substituted

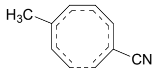

C	0.29120100	-1.50218300	-0.85119900
C	-0.92663600	-0.83561000	1.01237800
C	1.52807400	-1.30773700	-0.30365500
C	2.13348400	-0.04395500	-0.04621900
C	1.53650900	1.20607400	-0.07084900
C	0.22380400	1.76502200	-0.22153500
C	-1.09249700	1.37183400	-0.09528200
C	-1.64583500	0.14675800	0.37645700
H	-0.19558500	-0.70305500	-1.39648700
H	-0.07764800	-2.50775600	-1.03196100
H	0.01188100	-0.57261600	1.48049600
H	-1.41183700	-1.74938000	1.34078000
H	0.29702400	2.82227500	-0.47591800
H	-1.82365200	2.11813700	-0.39776500
C	-3.02789300	-0.11503700	0.05153100
H	2.26900500	2.00919100	0.01080300
H	2.09436800	-2.18390300	0.01388000
C	3.61300000	-0.10734600	0.28314900
N	-4.13620400	-0.32797700	-0.21014100
H	3.77785100	-0.70565700	1.18723400
H	4.17703600	-0.58280600	-0.52645900
H	4.03572100	0.88449100	0.45772900

2-CN-6-NH_2-substituted

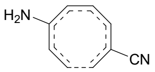

C	0.30473400	-1.49069100	-0.82258000
C	-0.93459100	-0.86339900	0.99995500
C	1.54678200	-1.32208200	-0.27716400
C	2.15512600	-0.05864600	-0.03702000
C	1.56276900	1.20371100	-0.08281100
C	0.25542300	1.75718500	-0.19098300
C	-1.06874500	1.36702500	-0.07760200
C	-1.63280900	0.14293100	0.37132100
H	-0.15739300	-0.68744900	-1.38392400
H	-0.08152900	-2.48985800	-1.00005500
H	-0.00775900	-0.61947900	1.50012600
H	-1.44397600	-1.76986200	1.31166000
H	0.32724100	2.81852500	-0.42817500
H	-1.79086800	2.12248000	-0.37733700
C	-3.01629900	-0.09892200	0.03178900
H	2.30862200	1.99899800	-0.05116800
H	2.09380100	-2.19297100	0.08182600
N	-4.12468000	-0.29774600	-0.24076900
N	3.50665500	-0.12530200	0.33069100
H	4.06058200	-0.80652400	-0.17334300
H	3.98311400	0.76480000	0.40150900

2-CN-6-OH-substituted

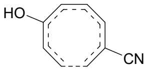

C	0.33742400	-1.51792600	-0.81950800
C	-0.92614900	-0.84275200	1.02405600
C	1.57302400	-1.31915600	-0.27395600
C	2.14380600	-0.04067000	-0.03088100
C	1.56335400	1.21612700	-0.05187300
C	0.25702500	1.76965600	-0.19570500
C	-1.06128800	1.36843000	-0.09653900
C	-1.62056600	0.14373400	0.36788600
H	-0.14643200	-0.72552700	-1.37697900
H	-0.03394100	-2.52503000	-0.98159300
H	0.01057600	-0.59356200	1.50335400
H	-1.43176500	-1.74570900	1.35138000
H	0.32983500	2.82900300	-0.43998100
H	-1.78877000	2.11304900	-0.41030400
C	-3.00114200	-0.11026500	0.02452100
H	2.32828300	1.98729700	0.02193200
H	2.13892500	-2.18014200	0.08338200
N	-4.10637300	-0.31846200	-0.25279400
O	3.48036300	-0.01125400	0.28725200
H	3.90206200	-0.84317100	0.03234700

2-CN-6-F-substituted

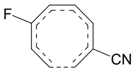

C	0.33550100	-1.55809900	-0.80367300
C	-0.90291700	-0.82558600	1.03139200
C	1.57734900	-1.34289800	-0.27563900
C	2.10912000	-0.05552800	-0.04013200
C	1.57180700	1.21014300	-0.04492400
C	0.27337000	1.78288300	-0.20626800
C	-1.04158000	1.37965700	-0.10346300
C	-1.59883800	0.15559200	0.36849200
H	-0.15955300	-0.77834800	-1.36830600
H	-0.02600300	-2.57069200	-0.95326500
H	0.03347700	-0.57324700	1.50987700
H	-1.40890500	-1.72505600	1.36755200
H	0.35493200	2.83958200	-0.45705900
H	-1.77138300	2.12027100	-0.42178300
C	-2.97783600	-0.10258600	0.02391200
H	2.35289400	1.96249000	0.05032900
H	2.18912600	-2.17511400	0.06428100
N	-4.08200700	-0.31327500	-0.25525500
F	3.43706700	-0.08538000	0.25522000

2-CN-6-CHO-substituted

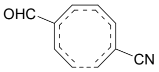

C	0.06980800	-1.44491400	-0.92379900
C	-1.12091300	-0.83182300	1.00983900
C	1.28881400	-1.15068600	-0.38683700
C	1.77531700	0.16478400	-0.13845600
C	1.11993900	1.38496600	-0.11676300
C	-0.22112500	1.87312400	-0.21187900
C	-1.49529800	1.36831400	-0.06043200
C	-1.93169100	0.09098400	0.39664800
H	-0.49993900	-0.68704100	-1.44725400
H	-0.21954400	-2.47739900	-1.09786300
H	-0.19238500	-0.49954800	1.45389600
H	-1.51749400	-1.78792400	1.33612300
H	-0.22960600	2.93949000	-0.43309600
H	-2.29504600	2.05746100	-0.32288200
C	-3.29380500	-0.27844900	0.09307900
H	1.81884000	2.22001600	-0.03404000
H	1.95245500	-1.95703500	-0.07990200
C	3.23507700	0.25035200	0.17184600
N	-4.38656600	-0.57081400	-0.15540800
H	3.63411300	1.27844200	0.29173600
O	3.96222700	-0.70633500	0.30270800

2-CN-6-CN-substituted

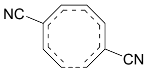

C	0.03125100	-1.53127300	-0.89685500
C	-1.12237500	-0.80580000	1.04414100
C	1.26917600	-1.32484800	-0.36592600
C	1.84439900	-0.03687900	-0.13428300
C	1.27781000	1.22975100	-0.14111100
C	-0.03325800	1.79045400	-0.26000500

C	-1.33877800	1.38022900	-0.09662000
C	-1.86394900	0.15070500	0.39962400
H	-0.48006800	-0.73779100	-1.42712800
H	-0.33755500	-2.54055800	-1.05192300
H	-0.16737200	-0.53168500	1.47212500
H	-1.58471100	-1.72260900	1.39588500
H	0.03060000	2.84698100	-0.51595700
H	-2.08794900	2.11503100	-0.38303200
C	-3.24913100	-0.13226400	0.10597800
H	2.02880600	2.01506100	-0.07263600
H	1.86013900	-2.17989500	-0.04514700
C	3.26343100	-0.06184600	0.15345000
N	4.39646200	-0.09873600	0.38948700
N	-4.35836900	-0.36039500	-0.13556400

2-CN-6-NO_2-substituted

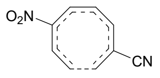

C	-0.34451200	-1.50618000	-0.93572200
C	-1.48447400	-0.81505300	1.02805800
C	0.89571900	-1.31315600	-0.40548400
C	1.44748800	-0.02498200	-0.17259700
C	0.89909700	1.23600300	-0.15026000
C	-0.41239300	1.80058800	-0.24699900
C	-1.71508700	1.38686000	-0.07702800
C	-2.23378100	0.14774000	0.40282400
H	-0.85899900	-0.70773300	-1.45547900
H	-0.71772800	-2.51315500	-1.09472400
H	-0.52893100	-0.54406100	1.45684600
H	-1.93978400	-1.74087900	1.36511800
H	-0.34961200	2.86046800	-0.48738500
H	-2.46781400	2.12579000	-0.34300500
C	-3.61924800	-0.13583600	0.11157900
H	1.66223900	2.00626800	-0.08049100
H	1.48909900	-2.16374200	-0.08901200
N	-4.72876500	-0.36390100	-0.12852500
N	2.92429600	-0.04922300	0.11076400
O	3.53056300	1.00448400	0.15853300
O	3.43768100	-1.14035900	0.28224500

2-NO_2-6-CH_3-substituted

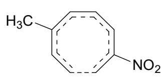

C	0.64060700	-1.45148900	-0.92375100
C	-0.57631400	-0.86410400	0.97445300
C	1.86967900	-1.28322700	-0.35421100
C	2.48306300	-0.03224000	-0.04549000
C	1.89503300	1.22044200	-0.02037200
C	0.58523700	1.79504700	-0.15018800
C	-0.73121900	1.40581800	-0.03117900
C	-1.25859200	0.16141900	0.38992500
H	0.16071900	-0.62742300	-1.43806800
H	0.26330600	-2.44663300	-1.14071800
H	0.35780100	-0.63313200	1.46650000

H	-1.08412100	-1.78688900	1.22935500
H	0.66528600	2.86049800	-0.36375000
H	-1.47191200	2.15613700	-0.28742000
H	2.63235000	2.01462600	0.09641200
H	2.42737000	-2.17354500	-0.06165500
C	3.96128300	-0.11975900	0.28333100
N	-2.67375800	-0.09882700	0.03430700
O	-3.07084800	-1.25067000	0.09456400
O	-3.36426000	0.85029500	-0.29371700
H	4.39134900	0.86182800	0.49401200
H	4.11958000	-0.75178200	1.16540600
H	4.52278600	-0.57034000	-0.54211300

2-NO_2-6-NH_2-substituted

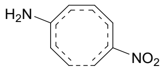

C	0.65037600	-1.44841100	-0.87772700
C	-0.58000200	-0.88133000	0.97246600
C	1.88747000	-1.30197600	-0.31826100
C	2.50346400	-0.04825700	-0.03843500
C	1.91829700	1.21714800	-0.04166400
C	0.61365200	1.78342200	-0.13000100
C	-0.71095400	1.39837300	-0.01974500
C	-1.24724100	0.15865900	0.38894700
H	0.19465400	-0.62371000	-1.41329800
H	0.25350700	-2.43829700	-1.08175600
H	0.34195000	-0.65789200	1.49035900
H	-1.10341600	-1.79677000	1.22145200
H	0.69179000	2.85122000	-0.33220800
H	-1.44328200	2.15620600	-0.27599900
H	2.66862100	2.00648800	0.01818000
H	2.42777400	-2.18575300	0.01886400
N	-2.66354400	-0.08766800	0.01999800
O	-3.06356400	-1.24005700	0.04653200
O	-3.35108600	0.87086500	-0.28511700
N	3.85415900	-0.13619300	0.32565300
H	4.40444400	-0.80411000	-0.19970200
H	4.33647000	0.74740800	0.42974800

2-NO_2-6-OH-substituted

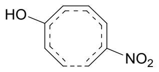

C	0.65905700	-1.47026400	-0.87885500
C	-0.56579900	-0.87464600	0.97494000
C	1.90176300	-1.31836400	-0.33335800
C	2.49088300	-0.06042200	-0.04076000
C	1.92691200	1.20686200	-0.02630600
C	0.62770800	1.78564100	-0.14169400
C	-0.69446200	1.40068600	-0.03156800
C	-1.23136300	0.16283800	0.38667600
H	0.19309500	-0.64775900	-1.40798800
H	0.26882400	-2.46239800	-1.08413900
H	0.36047500	-0.65362200	1.48578300
H	-1.09203000	-1.78631800	1.23203900

H	0.71343200	2.85104700	-0.35168400
H	-1.42738800	2.15589600	-0.29413400
H	2.68700200	1.98653300	0.05519100
H	2.48005000	-2.18708800	-0.02771200
N	-2.64969600	-0.08232500	0.02317100
O	-3.05230100	-1.23297300	0.06085800
O	-3.33399900	0.87640800	-0.28805300
O	3.82315900	-0.18490600	0.25335500
H	4.18134900	0.65777000	0.56671700

2-NO_2-6-F-substituted

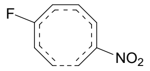

C	0.67823900	-1.51766700	-0.86697800
C	-0.55473500	-0.85083700	1.00094700
C	1.91461100	-1.32102800	-0.32272000
C	2.45262400	-0.04326900	-0.04139400
C	1.92312700	1.22381100	0.00138000
C	0.62754700	1.81162100	-0.13735500
C	-0.68771200	1.41255200	-0.03952200
C	-1.21648800	0.17043500	0.38677600
H	0.18512900	-0.71789800	-1.40519700
H	0.31062900	-2.52327400	-1.04575400
H	0.37718600	-0.62528700	1.50016500
H	-1.07978700	-1.76007800	1.26915000
H	0.71492300	2.87591800	-0.34981000
H	-1.42855600	2.15647700	-0.31303500
H	2.70845600	1.96741000	0.12564100
H	2.52173900	-2.16576700	-0.00582100
N	-2.63098200	-0.08964800	0.01371200
O	-3.02386200	-1.24277100	0.06055900
O	-3.31807600	0.86083600	-0.31415000
F	3.77993100	-0.09313200	0.25228800

2-NO_2-6-CHO-substituted

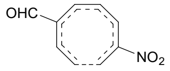

C	-0.23876000	1.62358000	-0.95201500
C	0.89083200	0.81828600	1.02399600
C	-1.47029300	1.46862100	-0.39429300
C	-2.12516900	0.22620800	-0.12952400
C	-1.64421100	-1.06940700	-0.11704600
C	-0.38157700	-1.73172700	-0.24610000
C	0.94710200	-1.40775100	-0.09233100
C	1.52304900	-0.20496800	0.39118800
H	0.24439200	0.80168500	-1.46567100
H	0.17500700	2.61400300	-1.11643300
H	-0.08229900	0.63233100	1.45689600
H	1.44030200	1.70082300	1.33010600
H	-0.52035800	-2.78406100	-0.48974000
H	1.66019000	-2.18061000	-0.36128500
H	-2.45988600	-1.78639900	-0.01558200
H	-2.00770800	2.36450900	-0.08058300
C	-3.58763700	0.36141100	0.17178000

N	2.96044900	-0.00310100	0.07549800
O	3.41250900	1.12144000	0.20133500
O	3.60351100	-0.97093300	-0.28865500
H	-3.95294900	1.40713900	0.22975400
O	-4.35350100	-0.55716100	0.34358500

2-NO_2-6-CN-substituted

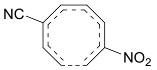

C	0.39095600	-1.47810200	-0.97492100
C	-0.76913300	-0.83855600	0.99623200
C	1.62023800	-1.29458000	-0.41913000
C	2.20238600	-0.01839100	-0.13344100
C	1.64584000	1.25083300	-0.08935400
C	0.33863800	1.82672600	-0.18830100
C	-0.96733700	1.41735400	-0.03813800
C	-1.46719000	0.16708300	0.40135300
H	-0.11377300	-0.66281900	-1.47853200
H	0.01667400	-2.47911600	-1.16623500
H	0.18034700	-0.60246300	1.45664700
H	-1.26324400	-1.76408700	1.26887700
H	0.40950200	2.89213700	-0.40069800
H	-1.72579800	2.15493200	-0.28047100
H	2.40224900	2.02676000	0.01534800
H	2.20295200	-2.16347600	-0.12098100
C	3.62034800	-0.06546000	0.15710700
N	4.75245900	-0.12015600	0.39391500
N	-2.88781300	-0.11419400	0.07217300
O	-3.26322700	-1.27103500	0.14311800
O	-3.59301200	0.82567800	-0.24624500

2-NO_2-6-NO_2-substituted

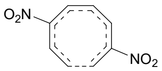

C	0.01588600	-1.45446500	-1.01034200
C	-1.13033300	-0.84298700	0.98125900
C	1.24730800	-1.28399000	-0.45538900
C	1.80644600	-0.00675300	-0.17327600
C	1.26914300	1.25604000	-0.10433700
C	-0.03803900	1.83600400	-0.18221900
C	-1.34137000	1.42396700	-0.02469800
C	-1.83539700	0.16668500	0.40239500
H	-0.49091400	-0.63501400	-1.50523400
H	-0.36379700	-2.45306300	-1.20327900
H	-0.17941200	-0.61047500	1.44054900
H	-1.61875200	-1.77486200	1.24260200
H	0.03266400	2.90343600	-0.38236900
H	-2.10280300	2.16400700	-0.24980800
H	2.03870900	2.01630600	-0.00283200
H	1.83168600	-2.14774900	-0.15788600
N	-3.25659900	-0.11528700	0.07707400
O	-3.62826300	-1.27367000	0.13886700
O	-3.96508800	0.82599100	-0.22939600
N	3.28253200	-0.05261500	0.11432200

O	3.90207700	0.99265900	0.16973400
O	3.77992600	-1.15176400	0.28056100

5.3.13 2,7-substituted

2-CH_3-7-CH_3-substituted

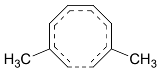

C	0.71268100	-0.99031200	-0.90480900
C	-0.71267800	-0.99031300	0.90480300
C	1.77579800	-0.37900100	-0.29067100
C	1.74992400	1.00544200	0.02934100
C	0.71300600	1.92096900	0.06738100
C	-0.71300400	1.92096900	-0.06738100
C	-1.74992300	1.00544200	-0.02934000
C	-1.77579700	-0.37900200	0.29066800
H	-0.01735000	-0.39177500	-1.43577600
H	0.75921400	-2.04468900	-1.16770500
H	0.01735200	-0.39177600	1.43577100
H	-0.75921100	-2.04469000	1.16769900
H	2.71867100	1.42720800	0.30085300
H	-1.07839900	2.93740000	-0.21599100
H	-2.71866900	1.42720900	-0.30085200
C	-2.95914000	-1.18416800	-0.19168500
H	-2.98372000	-2.17643700	0.26671800
H	-3.89979700	-0.67538400	0.04658000
H	-2.93019300	-1.31388200	-1.28133300
H	1.07840100	2.93740000	0.21599500
C	2.95913500	-1.18417100	0.19169000
H	3.89979900	-0.67542500	-0.04663200
H	2.93021700	-1.31382400	1.28134600
H	2.98367300	-2.17646600	-0.26665800

2-CH_3-7-NH_2-substituted

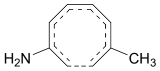

C	-0.74587600	-1.04120700	0.92016400
C	0.74637300	-0.97979900	-0.92469100
C	-1.78394400	-0.43357600	0.26845300
C	-1.78370000	0.96473100	-0.03154100
C	-0.76432800	1.88989100	-0.06054900
C	0.66491800	1.92221400	0.08881100
C	1.71903600	1.03340100	0.04021000
C	1.78288900	-0.35106800	-0.29528000
H	-0.04270400	-0.43381900	1.47363300
H	-0.77519000	-2.10478400	1.14315200
H	-0.01546400	-0.40491700	-1.43644900
H	0.80904800	-2.03429300	-1.18344500
H	-2.76292300	1.37246900	-0.28615600
H	1.00160300	2.94440100	0.26172900
H	2.67747800	1.47621400	0.31646600
C	2.98735800	-1.12544400	0.18644500
H	3.02398500	-2.12407300	-0.25735100
H	3.91609300	-0.60469500	-0.07339900

H	2.97632900	-1.23949500	1.27809800
H	-1.14954700	2.89993100	-0.20262700
N	-2.83268700	-1.20111200	-0.26791800
H	-3.60577000	-0.66319900	-0.63893600
H	-3.16048900	-1.95081200	0.32858100

2-CH_3-7-OH-substituted

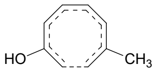

C	0.72491500	-1.10158100	-0.88866800
C	-0.76121200	-0.96457800	0.93932800
C	1.76447300	-0.47311800	-0.26819300
C	1.80856600	0.91727600	0.02864100
C	0.81145800	1.86986000	0.05929700
C	-0.61350000	1.93101600	-0.08719600
C	-1.68535400	1.05985100	-0.04728200
C	-1.77979000	-0.31902900	0.29389200
H	0.01590700	-0.51003500	-1.45070000
H	0.78732000	-2.16551100	-1.09434600
H	-0.00197200	-0.39383400	1.45935900
H	-0.84996100	-2.01363200	1.21101100
H	2.80415800	1.29788400	0.26238100
H	-0.93088900	2.95967200	-0.25723600
H	-2.63196900	1.51983800	-0.33545100
C	-2.99123600	-1.07501400	-0.19865200
H	-3.05849300	-2.06556300	0.25897800
H	-3.91269600	-0.52971700	0.03449200
H	-2.96055000	-1.20606300	-1.28796500
H	1.22211000	2.87059200	0.19328500
O	2.81439100	-1.25600100	0.15025500
H	3.33199400	-0.78373100	0.81714600

2-CH_3-7-F-substituted

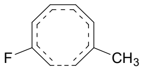

C	-0.70407900	-1.13500300	0.83847500
C	0.76291300	-0.95553900	-0.94329500
C	-1.74316600	-0.47191100	0.25790600
C	-1.82780600	0.89471800	-0.06987600
C	-0.84507400	1.86707500	-0.07321700
C	0.57106300	1.93612100	0.10604700
C	1.65280400	1.07103300	0.07756800
C	1.77053600	-0.29685700	-0.28489300
H	0.00844700	-0.56479400	1.41730500
H	-0.79039900	-2.19894100	1.03363300
H	0.00634300	-0.39075000	-1.47363800
H	0.88403600	-1.99523200	-1.23794300
H	-2.82572600	1.22234600	-0.35243200
H	0.88037600	2.96489000	0.29004800
H	2.58796500	1.53509600	0.39436900
C	2.99214100	-1.04177400	0.19990200
H	3.08029500	-2.02119000	-0.27735100
H	3.90485900	-0.47532400	-0.01526500
H	2.95644700	-1.19556000	1.28614800

H	-1.26478000	2.86221800	-0.21733000
F	-2.80042900	-1.21666000	-0.16658200

2-CH_3-7-CHO-substituted

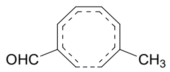

C	-0.69410100	-0.69807700	0.92725600
C	0.75108600	-0.99850200	-0.99082300
C	-1.51828700	0.19783100	0.30673300
C	-1.16875500	1.53996200	-0.03872800
C	0.04594700	2.17803000	-0.09440100
C	1.43051600	1.82202400	0.09054100
C	2.19533700	0.68134900	0.05155700
C	1.87907900	-0.67313900	-0.30077700
H	0.18982400	-0.36537400	1.45514900
H	-1.05872200	-1.70354200	1.12253100
H	0.16710000	-0.22867100	-1.47998300
H	0.53937600	-2.02848000	-1.26624100
H	-2.01708400	2.16815600	-0.31375500
H	2.02968600	2.71308000	0.27934000
H	3.23421800	0.83551100	0.34577400
C	2.81455600	-1.74214800	0.20969700
H	2.56162500	-2.72381200	-0.19838500
H	3.85089600	-1.51358400	-0.06337500
H	2.77641000	-1.80564900	1.30448200
H	-0.05377900	3.24592000	-0.28766100
C	-2.86158000	-0.26906500	-0.12520400
H	-3.53351500	0.52254600	-0.51811000
O	-3.24110400	-1.41821100	-0.07436000

2-CH_3-7-CN-substituted

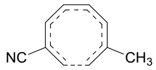

C	-0.60725800	-0.80403900	0.96878300
C	0.77478700	-1.03927600	-0.92464800
C	-1.55603300	-0.01578800	0.36977100
C	-1.35123900	1.34339900	-0.01789700
C	-0.19137900	2.07923700	-0.10279300
C	1.22222600	1.85768000	0.03907000
C	2.09739000	0.79434400	0.00210700
C	1.90568200	-0.58810500	-0.30418200
H	0.23095200	-0.33096400	1.46282500
H	-0.83025500	-1.83313900	1.23224100
H	0.11772200	-0.34461700	-1.43381100
H	0.65896600	-2.09149600	-1.17247900
H	-2.25735000	1.88351100	-0.28320100
H	1.73931800	2.80645400	0.18167100
H	3.12348600	1.05947100	0.25889300
C	2.96427300	-1.55008500	0.17837200
H	2.79066100	-2.55977200	-0.20169700
H	3.95827400	-1.22750200	-0.15086900
H	2.98665500	-1.59447100	1.27441300
H	-0.39452800	3.13163000	-0.29828000
C	-2.81526700	-0.61589100	-0.00119400

N	-3.82614200	-1.09542400	-0.30200500

2-CH_3-7-NO_2-substituted

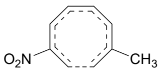

C	-0.29582200	-0.73848000	0.95566200
C	1.05905000	-0.97530600	-0.96938000
C	-1.19184800	0.11638300	0.39145700
C	-0.96761800	1.47206700	0.03394900
C	0.21989700	2.15954000	-0.04392500
C	1.62487100	1.87256300	0.08936300
C	2.45275000	0.77587600	0.01633700
C	2.20120100	-0.59095600	-0.32873600
H	0.56092600	-0.31418400	1.46155600
H	-0.56882300	-1.76576400	1.16666900
H	0.43182400	-0.23618400	-1.45361600
H	0.89201800	-2.01438500	-1.24161400
H	-1.86149700	2.03943900	-0.20299600
H	2.18175600	2.79335700	0.26252400
H	3.49008800	0.98863700	0.27648500
C	3.21291400	-1.60948300	0.13655600
H	2.99453000	-2.60254300	-0.26344100
H	4.22180500	-1.32587100	-0.18309100
H	3.22889500	-1.67558100	1.23163100
H	0.06176400	3.22388900	-0.21318900
N	-2.49901100	-0.45510700	-0.00508300
O	-2.62746700	-1.66732100	0.04384100
O	-3.37660600	0.31503700	-0.35546900

2- NH_2-7-NH_2-substituted

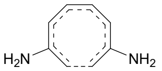

C	0.76683900	-1.02477200	-0.92993200
C	-0.76355500	-1.03650000	0.90935300
C	1.78931200	-0.40293100	-0.27009500
C	1.75574000	0.99167700	0.03932500
C	0.71692000	1.89517200	0.06720600
C	-0.71351200	1.89657300	-0.08129200
C	-1.75564900	0.99796500	-0.02982900
C	-1.79072800	-0.39696100	0.27473800
H	0.05406000	-0.42727600	-1.48220900
H	0.81446000	-2.08676300	-1.15745800
H	-0.03051700	-0.46050800	1.45843300
H	-0.82071400	-2.09841800	1.13736500
H	2.72381300	1.42011200	0.30361000
H	-1.07185400	2.91409600	-0.23676100
H	-2.73224300	1.42174100	-0.26424400
N	-2.95421600	-1.08063400	-0.13941400
H	-2.92771200	-2.07699800	0.04194100
H	-3.22404800	-0.90195000	-1.10068000
H	1.07763400	2.91257600	0.21863300
N	2.85207000	-1.15712800	0.26816700
H	3.60538100	-0.60121000	0.65400500
H	3.21456000	-1.87240500	-0.35074000

2- NH_2-7-OH-substituted

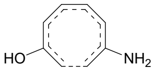

C	-0.75044900	-1.06534300	0.86352100
C	0.76623100	-1.02204700	-0.90810800
C	-1.78516800	-0.41421900	0.24982800
C	-1.78034000	0.96637400	-0.06890400
C	-0.75180000	1.88662000	-0.07629000
C	0.67110200	1.90178700	0.09578200
C	1.72795300	1.01356600	0.05731000
C	1.78856800	-0.37176700	-0.26448600
H	-0.04094500	-0.48396300	1.43608800
H	-0.81659400	-2.12640700	1.09483100
H	0.04956500	-0.44461000	-1.47740000
H	0.85456500	-2.07618800	-1.16212700
H	-2.75667100	1.35129400	-0.35690500
H	1.01775600	2.92193100	0.26040400
H	2.69295700	1.44790800	0.31836600
N	2.95489000	-1.04783200	0.14552500
H	2.94918400	-2.04124100	-0.05095000
H	3.23432100	-0.87082200	1.10391000
H	-1.12372800	2.89920700	-0.23055300
O	-2.88811200	-1.08324200	-0.23082000
H	-2.95633600	-1.94618500	0.20030100

2- NH_2-7-F-substituted

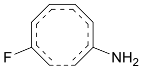

C	0.73605100	-1.13196800	-0.85192700
C	-0.79126800	-0.99526200	0.96371400
C	1.74753600	-0.44277100	-0.26476800
C	1.79267100	0.92727400	0.07474800
C	0.79062100	1.87408200	0.09098500
C	-0.63001100	1.91076400	-0.09883300
C	-1.69227400	1.03230200	-0.07900300
C	-1.77768400	-0.35122100	0.26600600
H	-0.01490900	-0.59344000	-1.41180300
H	0.84526500	-2.19541000	-1.03796600
H	-0.06732500	-0.41145600	1.51568200
H	-0.88953500	-2.04581000	1.22467400
H	2.78250000	1.27785600	0.35937200
H	-0.96111000	2.93406300	-0.27556100
H	-2.64009400	1.47930700	-0.38204000
N	-2.84986900	-1.07936400	-0.27762100
H	-3.22710800	-1.79886800	0.32690400
H	-3.58755200	-0.51579300	-0.68125800
H	1.18238800	2.87678400	0.25746500
F	2.83030100	-1.15454200	0.16026000

2- NH_2-7-CHO-substituted

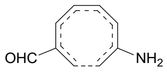

C	-0.73320400	-0.69713200	0.94453600
C	0.81516600	-1.03702600	-1.06069400
C	-1.51637500	0.21047700	0.30394200
C	-1.12506900	1.54538500	-0.06111300
C	0.09631300	2.15479000	-0.12009200
C	1.47620400	1.77390000	0.11263800
C	2.22237100	0.63256700	0.08160600
C	1.88743600	-0.72669500	-0.28886600
H	0.18786000	-0.41328100	1.43625100
H	-1.12444300	-1.69335200	1.13575800
H	0.25785100	-0.24902600	-1.54828900
H	0.58791800	-2.06415900	-1.33198200
H	-1.95764400	2.18830000	-0.35089000
H	2.08141500	2.65453500	0.32886000
H	3.26088600	0.76668800	0.38695300
N	2.67974000	-1.72183900	0.29638400
H	2.76143400	-2.57887800	-0.23621800
H	3.57616600	-1.41244100	0.64965300
H	0.02687800	3.21748400	-0.35073400
C	-2.87256000	-0.21773300	-0.12753900
H	-3.50840600	0.58652400	-0.55457400
O	-3.30122300	-1.34759000	-0.04324700

2-NH_2-7-CN-substituted

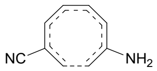

C	-0.63738700	-0.80292600	0.99936800
C	0.82240000	-1.08259400	-0.98110600
C	-1.55373200	-0.00424400	0.38276400
C	-1.31706300	1.35469200	-0.02308400
C	-0.15127800	2.06442200	-0.12596200
C	1.26538700	1.82035200	0.03982500
C	2.12384800	0.75663900	0.00707700
C	1.91043000	-0.63836100	-0.29389300
H	0.24027500	-0.36762700	1.45858900
H	-0.87443500	-1.83142100	1.25247500
H	0.18736600	-0.37461100	-1.49641100
H	0.68965100	-2.13720500	-1.20670500
H	-2.21456800	1.90870200	-0.28978700
H	1.79252600	2.76230000	0.19086700
H	3.15627300	1.00715700	0.25295800
N	2.82445800	-1.53346700	0.27053000
H	2.98568100	-2.38358600	-0.25460400
H	3.68763300	-1.12652800	0.60657800
H	-0.33051600	3.11411200	-0.35530200
C	-2.82434200	-0.57542300	0.00569200
N	-3.84581000	-1.02748200	-0.30235200

2-NH_2-7-NO_2-substituted

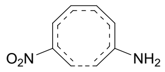

C	-0.32964600	-0.75289300	0.97963200
C	1.11426200	-1.00772700	-1.05183400
C	-1.18946100	0.11703800	0.40298300
C	-0.92981500	1.47645600	0.04041600
C	0.26282500	2.13760700	-0.04311400
C	1.66947800	1.82483600	0.11997300
C	2.47954700	0.72928800	0.03725400
C	2.20755900	-0.64359300	-0.33057500
H	0.56917900	-0.37428800	1.44710200
H	-0.61972700	-1.77904600	1.17221200
H	0.50850900	-0.24683700	-1.52564200
H	0.93307300	-2.04352300	-1.32508900
H	-1.81236600	2.06135800	-0.19575100
H	2.23461300	2.73455000	0.32237900
H	3.52133100	0.92227500	0.29536300
N	3.07741000	-1.59629400	0.20475800
H	3.19051700	-2.44278900	-0.33839900
H	3.96055700	-1.24388100	0.55025400
H	0.13052800	3.20128700	-0.23600800
N	-2.50892200	-0.41795800	-0.00182100
O	-3.32851100	0.36626000	-0.45121400
O	-2.70950400	-1.61318700	0.13679200

2-OH-7-OH-substituted

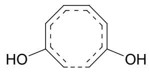

C	0.75817600	-1.05271800	-0.87300800
C	-0.75816500	-1.05260100	0.87315100
C	1.77956000	-0.39000200	-0.24459900
C	1.74821900	0.98249700	0.09945500
C	0.70762300	1.89219000	0.10310400
C	-0.70781000	1.89215100	-0.10311600
C	-1.74833500	0.98237700	-0.09952200
C	-1.77961800	-0.39011000	0.24460200
H	0.04484900	-0.47955500	-1.44921700
H	0.85096900	-2.10790300	-1.12188900
H	-0.04483500	-0.47925800	1.44917200
H	-0.85082300	-2.10774000	1.12227300
H	2.71401200	1.37509400	0.41136500
H	-1.06621700	2.90658800	-0.27615200
H	-2.71413100	1.37489700	-0.41152800
O	-2.88977100	-1.04861800	-0.23235300
H	-2.98319700	-1.89758700	0.22129000
H	1.06598100	2.90664500	0.27611700
O	2.88993500	-1.04849700	0.23237700
H	2.98417500	-1.89696500	-0.22203300

2-OH-7-F-substituted

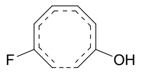

C	0.74014300	-1.11189900	-0.86060800
C	-0.77568800	-1.04284300	0.89433600
C	1.74540900	-0.41815000	-0.25621000
C	1.76750000	0.94162200	0.10513200
C	0.75228200	1.88086900	0.10729700
C	-0.66017400	1.90358000	-0.10593200
C	-1.71267000	1.00788300	-0.10782400
C	-1.77202800	-0.36275000	0.24448500
H	0.01101400	-0.56636800	-1.44273200
H	0.87506300	-2.16737900	-1.07427100
H	-0.05309400	-0.47885500	1.46770900
H	-0.89485300	-2.09247500	1.15375200
H	2.74587000	1.29943900	0.41850900
H	-1.00281400	2.92270100	-0.28295200
H	-2.66964300	1.41273000	-0.43077300
O	-2.88770400	-0.99886900	-0.24218500
H	-2.99662300	-1.85290400	0.19894600
H	1.13296200	2.88628500	0.28286800
F	2.82723300	-1.12912200	0.16915200

2-OH-7-CHO-substituted

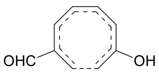

C	0.72593800	-0.67192700	-0.95204500
C	-0.76059000	-1.09140100	0.95641500
C	1.50753700	0.23695400	-0.29935900
C	1.09846800	1.55357100	0.08606100
C	-0.13593600	2.14859400	0.13738300
C	-1.50364100	1.75384300	-0.10608300
C	-2.23594300	0.59774000	-0.09907400
C	-1.87444500	-0.74240900	0.26137700
H	-0.17109400	-0.36810500	-1.47545000
H	1.13243000	-1.65695200	-1.16829600
H	-0.19849800	-0.32365900	1.46911600
H	-0.54807300	-2.12625900	1.21253800
H	1.91802000	2.20190100	0.39987900
H	-2.12289900	2.62781200	-0.30802900
H	-3.27326800	0.69070600	-0.41353500
O	-2.74926900	-1.66594300	-0.24210800
H	-2.55834000	-2.53818100	0.13188200
H	-0.07965300	3.21050700	0.37466900
C	2.86212400	-0.18994800	0.13448200
H	3.49890400	0.61474400	0.55844400
O	3.28694500	-1.32188300	0.05508800

2-OH-7-CN-substituted

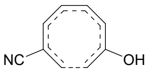

C	-0.63429000	-0.78296400	0.99964300
C	0.78728200	-1.13169400	-0.88349800
C	-1.54864000	0.01991300	0.37125800
C	-1.29128600	1.36155100	-0.05378100
C	-0.11186500	2.06125300	-0.14158700

C	1.29348200	1.80440300	0.04215000
C	2.14193500	0.72606300	0.03306100
C	1.90443400	-0.65001000	-0.26790600
H	0.22062300	-0.33275000	1.48687600
H	-0.89396700	-1.79771600	1.28441700
H	0.14896700	-0.44752800	-1.42538300
H	0.67542700	-2.19139600	-1.10030900
H	-2.17644400	1.92150600	-0.34783500
H	1.83251300	2.74033200	0.18686300
H	3.17618400	0.93599200	0.29689900
O	2.88692500	-1.46923000	0.21512700
H	2.78953700	-2.35828200	-0.15524600
H	-0.27949500	3.11184600	-0.37526600
C	-2.82171900	-0.54664100	-0.00247100
N	-3.84353500	-0.99991600	-0.30760700

2-OH-7-NO_2-substituted

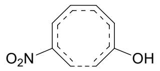

C	-0.32156300	-0.73404200	0.98390600
C	1.07447200	-1.06448100	-0.94421700
C	-1.18404000	0.13550400	0.39533500
C	-0.91060600	1.47749500	0.00770300
C	0.29279500	2.13160100	-0.06666200
C	1.68883900	1.81125600	0.11245900
C	2.49267000	0.70323100	0.05724400
C	2.20154400	-0.65268600	-0.30092600
H	0.55197200	-0.33534300	1.48220400
H	-0.62867700	-1.74839700	1.20999000
H	0.46125900	-0.33001400	-1.44820100
H	0.91575300	-2.10876300	-1.20163600
H	-1.78439500	2.06414000	-0.25598900
H	2.26278200	2.71803700	0.30171100
H	3.53423900	0.86014900	0.32906000
O	3.14961700	-1.52483200	0.15317100
H	3.00606200	-2.40135400	-0.23233800
H	0.16820300	3.19439000	-0.26895800
N	-2.50207000	-0.40153900	-0.00639300
O	-2.68737700	-1.60227600	0.10796200
O	-3.33441000	0.38344000	-0.42865200

2-F-7-F-substituted

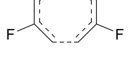

C	0.75984900	-1.10471000	-0.87980300
C	-0.75983300	-1.10469300	0.87980900
C	1.73842100	-0.39078500	-0.25474300
C	1.73184600	0.96745900	0.11483900
C	0.70443700	1.89384800	0.11048600
C	-0.70445900	1.89384400	-0.11049600
C	-1.73186300	0.96745100	-0.11484900
C	-1.73841700	-0.39079200	0.25474000
H	0.02232800	-0.57163100	-1.46244300
H	0.92365600	-2.15435800	-1.10094700

H	-0.02231800	-0.57158800	1.46243300
H	-0.92362300	-2.15433800	1.10097700
H	2.70150600	1.33753200	0.44052100
H	-1.06998900	2.90419300	-0.28954900
H	-2.70152600	1.33751000	-0.44053600
H	1.06995100	2.90418900	0.28958700
F	-2.82906600	-1.07897100	-0.17898800
F	2.82907900	-1.07894500	0.17899400

2-F-7-CHO-substituted

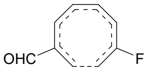

C	-0.73051300	-0.66714500	0.96120800
C	0.72150300	-1.15353500	-0.92148800
C	-1.49155200	0.25403900	0.29695100
C	-1.06273900	1.55936000	-0.09236600
C	0.18055100	2.14363900	-0.13676700
C	1.53816900	1.73167100	0.10081700
C	2.24350400	0.55426200	0.09856400
C	1.83837400	-0.75831500	-0.26741900
H	0.16275900	-0.37132000	1.49512300
H	-1.15798400	-1.64134000	1.18540400
H	0.13860800	-0.41594100	-1.45380600
H	0.55541500	-2.20405700	-1.13399000
H	-1.87190000	2.21662100	-0.41435700
H	2.17787300	2.59298700	0.29138500
H	3.28395000	0.61707200	0.40903000
H	0.13458200	3.20739300	-0.36721400
F	2.67843900	-1.72458900	0.18262700
C	-2.85425400	-0.15185400	-0.14061900
H	-3.47715200	0.66217100	-0.56668900
O	-3.29379400	-1.27687700	-0.06022600

2-F-7-CN-substituted

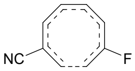

C	-0.63888300	-0.78396600	1.00952300
C	0.75997400	-1.18925600	-0.85534500
C	-1.53491100	0.03353100	0.36932000
C	-1.25892800	1.36753500	-0.05867900
C	-0.07134700	2.05983400	-0.13940200
C	1.32641100	1.78807900	0.03940200
C	2.15234700	0.68780800	0.03448100
C	1.87022000	-0.66523300	-0.27560600
H	0.21582400	-0.34261300	1.50481400
H	-0.92055700	-1.79038300	1.30231600
H	0.10260400	-0.53507500	-1.41043600
H	0.69821100	-2.25738900	-1.03526800
H	-2.13632400	1.93555500	-0.36026300
H	1.88370200	2.71404700	0.17642800
H	3.19202400	0.86862600	0.29746700
H	-0.23086400	3.11309400	-0.36617200
C	-2.81676900	-0.51278600	-0.00766500
N	-3.84499200	-0.94809900	-0.31611200

F	2.82018300	-1.53138300	0.15641400

2-F-7-NO_2-substituted

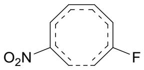

C	-0.32455900	-0.74257300	0.98960500
C	1.04720400	-1.12944500	-0.90937400
C	-1.16988500	0.14150800	0.39209400
C	-0.88236900	1.47574700	0.00273800
C	0.32704200	2.12678500	-0.06531200
C	1.71539000	1.79629500	0.10389000
C	2.49933400	0.66864800	0.05350800
C	2.16944100	-0.66399700	-0.30631500
H	0.54527900	-0.35285000	1.50091100
H	-0.65306700	-1.74880000	1.22220500
H	0.41042800	-0.43198400	-1.43529500
H	0.94457200	-2.18807300	-1.12309100
H	-1.75070100	2.06796200	-0.26689000
H	2.30581000	2.69480500	0.27971500
H	3.54520000	0.80087600	0.32086500
H	0.20624600	3.19159300	-0.25836500
F	3.08703500	-1.57503300	0.09876200
N	-2.49886100	-0.37792800	-0.01049200
O	-3.32515100	0.42142100	-0.41509500
O	-2.69168000	-1.57773800	0.08753600

2-CHO-7-CHO-substituted

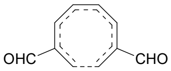

C	0.76616400	-0.66749300	-0.98792800
C	-0.76613200	-0.66732800	0.98830500
C	1.74322500	-0.01426700	-0.30139300
C	1.72415900	1.36781500	0.08324200
C	0.71114400	2.28872800	0.12267600
C	-0.71139200	2.28864100	-0.12298200
C	-1.72433000	1.36765400	-0.08325100
C	-1.74323600	-0.01437200	0.30158800
H	-0.02814900	-0.12536400	-1.48352700
H	0.88713800	-1.72398000	-1.21357100
H	0.02807000	-0.12496000	1.48382600
H	-0.88694800	-1.72378600	1.21416300
H	2.69344600	1.75503900	0.39968900
H	-1.06567000	3.29387100	-0.35034000
H	-2.69367000	1.75471900	-0.39974600
H	1.06531000	3.29405400	0.34978900
C	-2.93212300	-0.80197800	-0.14070600
H	-3.76069800	-0.20592200	-0.57626100
O	-3.02605600	-2.00396400	-0.05186700
C	2.93216000	-0.80180300	0.14090300
H	3.76045800	-0.20580300	0.57705000
O	3.02641700	-2.00371800	0.05139200

2-CHO-7-CN-substituted

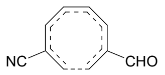

C	0.68692200	-0.74804000	-1.03429000
C	-0.74879600	-0.73351100	0.93760000
C	1.74438900	-0.18602500	-0.37996200
C	1.83079700	1.18579700	0.03536500
C	0.86452700	2.15548400	0.11550400
C	-0.56314800	2.24636700	-0.06247100
C	-1.63284000	1.38864300	-0.00922300
C	-1.72148700	0.00007400	0.32360400
H	-0.05590400	-0.10846900	-1.49229800
H	0.69440600	-1.79671900	-1.31345900
H	0.07705300	-0.24986400	1.44259600
H	-0.92143300	-1.78891600	1.13280100
H	2.82970000	1.50694300	0.32161600
H	-0.86338100	3.27861300	-0.23962500
H	-2.58679600	1.84799500	-0.27116300
H	1.28744700	3.13558500	0.33417700
C	2.84775300	-1.03724800	0.00423500
N	3.73707700	-1.70857600	0.31897200
C	-2.96626500	-0.70343200	-0.10309800
H	-3.78491200	-0.04538900	-0.46061000
O	-3.11085400	-1.90355200	-0.08130300

2-CHO-7-NO_2-substituted

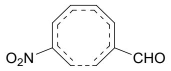

C	0.40980100	-0.62999600	-1.01381700
C	-1.01291000	-0.66857200	0.96487200
C	1.40797200	0.04678500	-0.39552500
C	1.43016000	1.41885600	-0.01223400
C	0.39701800	2.31758600	0.05689800
C	-1.03416000	2.29883900	-0.11354600
C	-2.03881900	1.36780300	-0.03368300
C	-2.02946200	-0.01590000	0.33217700
H	-0.37763600	-0.06412200	-1.49240500
H	0.50755300	-1.68596000	-1.23776500
H	-0.22234400	-0.12006700	1.46070800
H	-1.11255300	-1.72977300	1.17930800
H	2.41119400	1.80176000	0.24830600
H	-1.41028300	3.30160100	-0.31363800
H	-3.02381700	1.75136700	-0.30235000
H	0.74903600	3.33049900	0.24852000
C	-3.22402900	-0.81319000	-0.07855600
H	-4.08506300	-0.22147900	-0.45166200
O	-3.28517700	-2.01904300	-0.02755700
N	2.59854700	-0.75187600	0.00923000
O	2.52301400	-1.96368000	-0.07959400
O	3.57974500	-0.14652300	0.40175800

2-CN-7-CN-substituted

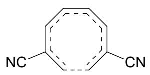

C	0.68375300	-0.81963200	-0.97646100
C	-0.68376600	-0.81963700	0.97648000
C	1.73354500	-0.17306100	-0.38304100
C	1.74340500	1.20957200	-0.01215500
C	0.71630500	2.11867200	0.06834900
C	-0.71628500	2.11866700	-0.06835000
C	-1.74339000	1.20957000	0.01215500
C	-1.73354600	-0.17306000	0.38304800
H	-0.09527000	-0.23991700	-1.45418900
H	0.75170100	-1.87325800	-1.22829700
H	0.09525700	-0.23992900	1.45421600
H	-0.75172100	-1.87326400	1.22831200
H	2.72558300	1.60388900	0.23841300
H	-1.08249800	3.12900000	-0.24647700
H	-2.72556100	1.60388900	-0.23843600
H	1.08250800	3.12899900	0.24648700
C	2.89535400	-0.94714000	-0.01073200
N	3.82637600	-1.56432000	0.29411700
C	-2.89536100	-0.94713200	0.01072400
N	-3.82638900	-1.56429800	-0.29413500

2-CN-7-NO_2-substituted

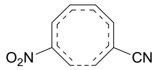

C	-0.39162600	-0.71238000	0.95852000
C	0.96239500	-0.74969900	-1.00138200
C	-1.38291600	0.02784700	0.39673600
C	-1.34898200	1.40553200	0.05105200
C	-0.27281900	2.25559400	-0.02554600
C	1.15814000	2.16816700	0.10334600
C	2.12987100	1.20242600	0.00232000
C	2.04210500	-0.17193900	-0.39235300
H	0.41838500	-0.19468900	1.45433000
H	-0.53684500	-1.76760500	1.15984000
H	0.21823800	-0.11747200	-1.46870400
H	0.96877800	-1.80175800	-1.26827700
H	-2.31611100	1.84274100	-0.17479900
H	1.58394100	3.15176100	0.29781400
H	3.13363200	1.53477800	0.25688900
H	-0.58057500	3.28818400	-0.18400700
C	3.16029800	-1.01240200	-0.02916500
N	4.06014600	-1.67667600	0.26977200
N	-2.61586200	-0.70481700	0.00160700
O	-3.59665300	-0.04618600	-0.29199100
O	-2.57062500	-1.92185900	-0.00224700

2-NO_2-7-NO_2-substituted

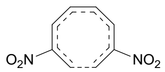

C	0.67610200	-0.63198200	-0.98092800
C	-0.67606100	-0.63195100	0.98110800
C	1.69971700	0.04603500	-0.40110900
C	1.74186000	1.41840400	-0.03281500
C	0.71690900	2.32765700	0.06166600
C	-0.71712300	2.32754900	-0.06226000
C	-1.74196700	1.41821500	0.03256000
C	-1.69968500	0.04593600	0.40117700
H	-0.10379700	-0.06439600	-1.47102500
H	0.76103400	-1.69178700	-1.19314300
H	0.10375500	-0.06427600	1.47124100
H	-0.76092700	-1.69176100	1.19333300
H	2.73254800	1.79581600	0.19865400
H	-1.08592300	3.33703400	-0.23813100
H	-2.73273400	1.79545100	-0.19890300
H	1.08559400	3.33723700	0.23722600
N	-2.89138500	-0.75877300	0.01310300
O	-2.77320000	-1.97040000	0.00754700
O	-3.91058200	-0.15787500	-0.27233000
N	2.89144800	-0.75863800	-0.01293500
O	2.77369800	-1.97031300	-0.00863500
O	3.91027100	-0.15773900	0.27381500

5.3.14 3,4-substituted

3-CH_3-4-CH_3-substituted

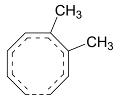

C	-2.10132900	-0.69862700	0.93918200
C	-1.02231000	-1.56488200	-0.92217500
C	-2.51544400	0.45177400	0.31818400
C	-1.65465500	1.46734200	-0.15392700
C	-0.27493600	1.54729100	-0.28754900
C	0.90343900	0.74717700	-0.09016100
C	1.12092800	-0.63523600	-0.02742300
C	0.21300100	-1.68256800	-0.34234800
H	-1.13693800	-0.73202000	1.42737400
H	-2.82641000	-1.44564300	1.25178100
H	-1.29258800	-0.66822400	-1.46367700
H	-1.60069000	-2.45795000	-1.14112600
H	-3.57208100	0.56500900	0.07998900
H	-2.16636200	2.36722700	-0.49237200
H	0.00729900	2.55605200	-0.58643700
H	0.51449400	-2.67857200	-0.01947900
C	2.50601800	-1.11496400	0.37462200
H	2.87087900	-0.62596400	1.28365000
H	2.50370400	-2.19322300	0.55126600
H	3.24405600	-0.91941400	-0.41328900
C	2.15748800	1.60544500	0.05810200
H	1.95257600	2.65327500	-0.16876200
H	2.53761500	1.57024600	1.08676200
H	2.97124200	1.27268400	-0.59473100

3-CH_3-4-NH_2-substituted

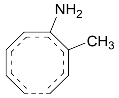

C	2.01515400	0.77231700	0.93399400
C	0.95148500	1.60413000	-0.90749300
C	2.51116900	-0.36647200	0.34688800
C	1.70227900	-1.41208600	-0.14793400
C	0.33373100	-1.53545900	-0.32008500
C	-0.87389500	-0.78248400	-0.08978000
C	-1.16432600	0.58965700	-0.02627000
C	-0.30548900	1.66570000	-0.35843400
H	1.04294500	0.74550500	1.40894900
H	2.69002100	1.55805500	1.26354600
H	1.26368100	0.72794300	-1.46033600
H	1.48844600	2.52520500	-1.11421500
H	3.57636700	-0.43105900	0.13299000
H	2.25204500	-2.28534300	-0.49633200
H	0.06594700	-2.53150100	-0.67434200
H	-0.65570900	2.64788800	-0.04100200
C	-2.57013700	0.98968500	0.38411500
H	-2.88007500	0.51647400	1.32453800
H	-2.63360800	2.07103900	0.52548800
H	-3.31257000	0.73009600	-0.38341900
N	-1.97497500	-1.66317000	-0.01375500
H	-1.80486400	-2.52138900	0.49385700
H	-2.86761600	-1.26064400	0.22656200

3-CH_3-4-OH-substituted

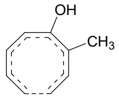

C	2.00559700	0.77749000	0.94892400
C	0.89449600	1.65297700	-0.89069900
C	2.51182300	-0.33027300	0.31812100
C	1.71962000	-1.38865400	-0.18006300
C	0.34999500	-1.53434300	-0.31829400
C	-0.84691800	-0.77961500	-0.08205400
C	-1.18914900	0.57058000	-0.02823900
C	-0.35566600	1.67726900	-0.32988400
H	1.03168500	0.72940900	1.41841000
H	2.66898300	1.56722700	1.29086800
H	1.22760100	0.79061400	-1.45302600
H	1.41829300	2.58602900	-1.07645700
H	3.57522400	-0.36892100	0.08975000
H	2.27987900	-2.24762700	-0.54576400
H	0.08778800	-2.53564800	-0.66738400
H	-0.73400500	2.64212100	0.00692800
C	-2.61980900	0.88254600	0.36058800
H	-2.90525900	0.37674400	1.28764300
H	-2.75452400	1.96007100	0.48544200
H	-3.31857400	0.53952000	-0.41042700
O	-1.96363200	-1.59030100	0.06051300
H	-1.68796700	-2.48497900	0.29950600

3-CH_3-4-F-substituted

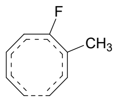

C	2.02431700	0.80341000	0.92162300
C	0.82887100	1.67604900	-0.88439700
C	2.52601300	-0.28846300	0.26456900
C	1.74365600	-1.38388700	-0.17065800
C	0.37475400	-1.55600400	-0.25613000
C	-0.80497900	-0.77928900	-0.07239200
C	-1.20972100	0.54355800	-0.01527200
C	-0.40319600	1.67959500	-0.28918900
H	1.07004600	0.73496600	1.42775200
H	2.67997200	1.61254000	1.23076900
H	1.15452800	0.82120800	-1.46332300
H	1.34648900	2.61308500	-1.06646500
H	3.57949700	-0.30142200	-0.00927600
H	2.30935600	-2.25129200	-0.50558100
H	0.08439200	-2.57512000	-0.50633100
H	-0.79218100	2.62923100	0.07625800
C	-2.65894800	0.79163100	0.35043600
H	-2.94996300	0.24789900	1.25336200
H	-2.83008400	1.85910500	0.50975900
H	-3.32547300	0.45923500	-0.45259200
F	-1.87235400	-1.64100200	0.04601400

3-CH_3-4-CHO-substituted

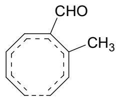

C	2.48075500	0.09980400	0.89822400
C	1.67220300	1.21554700	-0.98761400
C	2.43854100	-1.13009000	0.29921600
C	1.25654800	-1.81210100	-0.08304800
C	-0.06071700	-1.40748100	-0.19706100
C	-0.87186600	-0.22950600	-0.04781200
C	-0.61725300	1.15190600	-0.01122100
C	0.59664300	1.79535700	-0.36801700
H	1.61258500	0.48171200	1.41729900
H	3.43372600	0.56139000	1.14293400
H	1.56844800	0.26482900	-1.49378400
H	2.53245900	1.82118400	-1.25733900
H	3.37897100	-1.60429700	0.02099000
H	1.41133600	-2.85240800	-0.36447300
H	-0.70965500	-2.25139300	-0.42409000
H	0.69532200	2.83682600	-0.06632400
C	-1.70774700	2.12308000	0.43326100
H	-2.25091600	1.78076700	1.31724100
H	-1.25550400	3.08652000	0.68038300
H	-2.43879800	2.31446300	-0.36044900
C	-2.32833900	-0.60358500	0.00600200
H	-3.04647700	0.22909000	0.06327900
O	-2.76051200	-1.73578300	-0.04090600

3-CH_3-4-CN-substituted

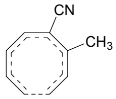

C	2.39381900	0.28238200	0.95233400
C	1.51826900	1.40843100	-0.89376900
C	2.55121600	-0.90379500	0.28796000
C	1.49123100	-1.72747200	-0.16485600
C	0.13254500	-1.50687700	-0.28186800
C	-0.80837400	-0.42803700	-0.09924400
C	-0.77004700	0.97109900	-0.02932900
C	0.34654500	1.80116800	-0.30367100
H	1.46265300	0.50484400	1.45586200
H	3.26085000	0.86026600	1.26003600
H	1.56639300	0.48508800	-1.45597100
H	2.29687900	2.13964300	-1.09055500
H	3.55755700	-1.22100000	0.01913000
H	1.79465000	-2.71913400	-0.49469900
H	-0.39695900	-2.41824100	-0.55475800
H	0.27010600	2.83116600	0.04003200
C	-2.05115000	1.69317600	0.34522400
C	-2.15303300	-0.96750900	0.00381900
H	-2.48651000	1.30086100	1.26923800
H	-1.86552200	2.76167100	0.47390300
H	-2.80745000	1.57642300	-0.43896900
N	-3.22268200	-1.40528400	0.08816500

3-CH_3-4-NO_2-substituted

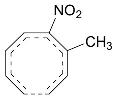

C	2.64103400	0.13084300	0.95909200
C	1.93902700	1.29348200	-0.92544400
C	2.68841900	-1.09158100	0.34218900
C	1.55452400	-1.80303500	-0.11408300
C	0.23364200	-1.42325000	-0.26138600
C	-0.54269500	-0.23605700	-0.09886600
C	-0.39804100	1.14427800	-0.08461500
C	0.80671100	1.83662700	-0.38032400
H	1.72678400	0.46235300	1.43348900
H	3.55594800	0.62442600	1.27475700
H	1.89196100	0.35197700	-1.45664600
H	2.79578800	1.92699900	-1.13526300
H	3.65976000	-1.52657600	0.11329400
H	1.74473900	-2.82887000	-0.42256200
H	-0.40712900	-2.25568200	-0.54634900
H	0.83266400	2.88330700	-0.08192100
C	-1.59161100	2.02333900	0.24867600
H	-1.85921200	1.95063800	1.30652700
H	-1.35946800	3.06358300	0.01344600
H	-2.47725800	1.74443300	-0.33107500
N	-1.97959600	-0.65507400	0.03794000
O	-2.51011800	-0.48867800	1.11952100
O	-2.51906600	-1.14369000	-0.93761100

3- NH_2-4-CH_3-substituted

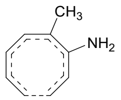

C	-2.14077300	-0.66225100	0.89062400
C	-1.05381700	-1.52320100	-0.88081600
C	-2.49348800	0.52133900	0.28124900
C	-1.59187000	1.51085600	-0.15439000
C	-0.20135700	1.55089600	-0.25188000
C	0.95062000	0.72597700	-0.07885700
C	1.12017500	-0.67104600	-0.01643700
C	0.18365800	-1.69272000	-0.31387300
H	-1.20862400	-0.72780600	1.43507100
H	-2.91074500	-1.37438000	1.17708700
H	-1.27074700	-0.63062700	-1.45278200
H	-1.66060900	-2.39643300	-1.10164300
H	-3.53950200	0.67678100	0.01889700
H	-2.06372900	2.43078800	-0.49552800
H	0.11199500	2.55897800	-0.52206900
H	0.45172100	-2.68646000	0.04563400
N	2.35919200	-1.17443300	0.42316800
H	3.13857600	-0.53474300	0.43860300
H	2.62039200	-2.06377500	0.01793000
C	2.24032600	1.52857800	0.05551200
H	2.66092300	1.44585200	1.06708800
H	3.01080100	1.20159300	-0.65547600
H	2.06435200	2.59069500	-0.12178400

3- NH_2-4-NH_2-substituted

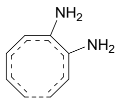

C	-2.07677800	-0.71435500	0.92141900
C	-0.96247200	-1.59385400	-0.90450100
C	-2.49466600	0.44126600	0.30864800
C	-1.64046900	1.46167600	-0.16156400
C	-0.26053600	1.55018100	-0.28947300
C	0.91109400	0.75863200	-0.07908600
C	1.15170300	-0.62579500	-0.01248900
C	0.28174900	-1.69247400	-0.33930000
H	-1.11384800	-0.74561400	1.41354600
H	-2.80396800	-1.45657100	1.24058900
H	-1.24439600	-0.70754700	-1.45761800
H	-1.52929400	-2.49650400	-1.11073700
H	-3.55148800	0.55264400	0.07081400
H	-2.15423600	2.35312600	-0.51739300
H	0.03215400	2.55265600	-0.60666300
H	0.60910400	-2.67632800	-0.00071300
N	2.47948600	-0.97946300	0.32243600
H	2.60027200	-1.97189000	0.48541500
N	2.09224200	1.52221200	0.12598400
H	1.95657500	2.51934300	0.01685700

H	2.88295500	1.19477400	-0.42543500
H	2.85632900	-0.42899100	1.09046900

3- NH_2-4-OH-substituted

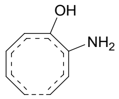

C	2.04612800	0.74350200	0.91233100
C	0.91617800	1.62039200	-0.85023600
C	2.49764700	-0.39020500	0.27649200
C	1.66822300	-1.42727900	-0.19376000
C	0.28591200	-1.53772600	-0.29306300
C	-0.88484200	-0.76256400	-0.06954700
C	-1.19567000	0.60368200	-0.01664900
C	-0.33422000	1.69265600	-0.29197500
H	1.09684500	0.72350000	1.43160200
H	2.75220100	1.50313500	1.23747100
H	1.19874700	0.75920300	-1.44183100
H	1.47198900	2.53512400	-1.03260400
H	3.55398900	-0.46250000	0.02225500
H	2.19357100	-2.30273100	-0.57058600
H	-0.01039700	-2.53740400	-0.61858700
H	-0.68018500	2.65341100	0.08887100
N	-2.48976500	0.94935000	0.40310900
H	-3.14016600	0.17454800	0.40425000
H	-2.87025200	1.76200700	-0.06452600
O	-2.04004900	-1.53986500	0.05346200
H	-1.81374000	-2.38957400	0.45266500

3- NH_2-4-F-substituted

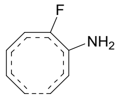

C	2.05725300	0.78304000	0.87960600
C	0.84631400	1.64032100	-0.85085300
C	2.51172500	-0.33956500	0.23031500
C	1.69738600	-1.41786100	-0.17442100
C	0.31786000	-1.55859300	-0.22594400
C	-0.84194000	-0.76885600	-0.05950500
C	-1.22079700	0.57237500	-0.00428400
C	-0.38769500	1.69014200	-0.25823300
H	1.12930100	0.74254500	1.43505700
H	2.75232600	1.56783200	1.16553900
H	1.12449800	0.78583700	-1.45495000
H	1.39229500	2.56043300	-1.03448900
H	3.55863900	-0.38432100	-0.06636400
H	2.23259800	-2.30125900	-0.51609300
H	0.00337300	-2.57576000	-0.45629900
H	-0.74608400	2.63646200	0.14520800
N	-2.53128900	0.86114500	0.39863000
H	-3.15209400	0.06301900	0.41644500
H	-2.94716300	1.64965800	-0.08028100
F	-1.94547800	-1.59761000	0.04841400

3- NH_2-4-CHO-substituted

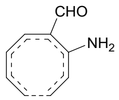

C	2.53379000	0.04633700	0.80234100
C	1.67940300	1.14651900	-0.99804800
C	2.39003700	-1.21174700	0.26907800
C	1.16749600	-1.86050900	-0.01702700
C	-0.14096000	-1.40707900	-0.10899000
C	-0.89903200	-0.19904000	-0.04154600
C	-0.56499000	1.17668000	0.02714000
C	0.64713200	1.79169700	-0.36454400
H	1.73468200	0.48193000	1.38521600
H	3.52718500	0.45235400	0.97498200
H	1.49978500	0.20804300	-1.50759400
H	2.55910300	1.70499100	-1.30279800
H	3.29109300	-1.74211800	-0.03859700
H	1.26837900	-2.91773200	-0.25495400
H	-0.82870100	-2.23245200	-0.28714900
H	0.79912800	2.82022900	-0.03851600
N	-1.56448900	2.03974200	0.48330500
H	-2.08775000	1.75047200	1.29850100
H	-1.32627400	3.02202500	0.50182800
C	-2.36881500	-0.45989200	-0.15003000
H	-2.98292500	0.41678000	-0.41805600
O	-2.89583100	-1.54756400	-0.02577900

3-NH_2-4-CN-substituted

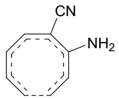

C	2.42865200	0.29517600	0.85890600
C	1.51976200	1.33291400	-0.87144300
C	2.53450400	-0.93842900	0.25494900
C	1.44912300	-1.75539000	-0.12094200
C	0.08469900	-1.51158000	-0.20519100
C	-0.84242000	-0.42757400	-0.07903100
C	-0.77093900	0.98819700	-0.00746200
C	0.35813600	1.78844700	-0.29356400
H	1.54943600	0.53007000	1.44305200
H	3.32711600	0.85259400	1.11020900
H	1.50602900	0.42407400	-1.45967200
H	2.30969600	2.04090000	-1.10392400
H	3.52312700	-1.28720500	-0.04110800
H	1.71993200	-2.76378400	-0.42598800
H	-0.45994800	-2.42739700	-0.42980700
H	0.31750300	2.81318400	0.07196600
C	-2.20126100	-0.91682100	-0.02003600
N	-3.30437200	-1.27192800	0.03639200
N	-1.89239500	1.68658900	0.42021700
H	-2.76123800	1.17511100	0.51332600
H	-2.01582600	2.61019000	0.02855300

3-NH_2-4-NO_2-substituted

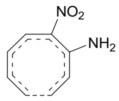

C	2.72197700	0.15718300	0.87131500
C	1.93340800	1.16221200	-0.95521800
C	2.68038000	-1.12005700	0.35775100
C	1.50709700	-1.81728800	0.01036000
C	0.18581300	-1.41083000	-0.12913100
C	-0.59482400	-0.22793800	-0.05554900
C	-0.38994600	1.16934200	-0.06945700
C	0.83545600	1.79027300	-0.42041200
H	1.87283300	0.54260200	1.41854800
H	3.68001400	0.61889900	1.09564300
H	1.81870700	0.21569800	-1.46801100
H	2.80022300	1.75523300	-1.23006800
H	3.62103800	-1.60919800	0.10795000
H	1.65252800	-2.86692900	-0.23387100
H	-0.46290600	-2.25498400	-0.34898200
H	0.92451300	2.83941000	-0.14199600
N	-1.44309700	2.03286200	0.17566700
H	-1.21371200	3.01588400	0.16358100
N	-2.03819600	-0.60991700	0.01265200
H	-2.14342400	1.76004500	0.85062100
O	-2.70698100	-0.15929200	0.93385800
O	-2.47513400	-1.36504100	-0.83255800

3-OH-4-CH_3-substituted

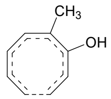

C	-2.12870200	-0.61579800	0.92048000
C	-1.04394500	-1.56856000	-0.86611600
C	-2.47591300	0.54167600	0.26826500
C	-1.57113100	1.52113900	-0.19581600
C	-0.18412300	1.55966800	-0.28064700
C	0.95598700	0.72400500	-0.06953700
C	1.10362100	-0.66363300	-0.01591700
C	0.19413000	-1.71066900	-0.29774000
H	-1.18189500	-0.67967600	1.43975700
H	-2.89824600	-1.31546700	1.23620200
H	-1.27684300	-0.68492300	-1.44485400
H	-1.65092700	-2.44866100	-1.05524100
H	-3.52182700	0.69249400	0.00358000
H	-2.04340800	2.43160400	-0.56090600
H	0.14530300	2.55755000	-0.56913100
H	0.47874100	-2.69609100	0.07540300
O	2.37023500	-1.06273000	0.36297400
H	2.47759800	-2.00703300	0.18734700
C	2.26542700	1.48393700	0.09437600
H	2.11857200	2.55371300	-0.06720800
H	2.66719600	1.34727100	1.10422600

H	3.03175200	1.12046100	-0.59705300

3-OH-4-NH_2-substituted

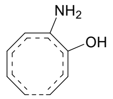

C	-2.04671900	-0.68475800	0.92998100
C	-0.96844800	-1.62677400	-0.84997000
C	-2.47566800	0.45456100	0.29315600
C	-1.62283000	1.46406700	-0.20482400
C	-0.24568600	1.55077100	-0.32790600
C	0.92605400	0.76540500	-0.06974900
C	1.14495100	-0.61567600	-0.01724600
C	0.28836100	-1.69740600	-0.30262800
H	-1.08474100	-0.69027700	1.42649600
H	-2.76781700	-1.42231000	1.27262100
H	-1.25434000	-0.76927600	-1.44414300
H	-1.52877900	-2.54164500	-1.01507700
H	-3.53267600	0.55358600	0.05188800
H	-2.13747200	2.34275300	-0.59023500
H	0.06893800	2.53326700	-0.68154400
H	0.62748300	-2.66160100	0.08015400
O	2.43692700	-0.94626300	0.37704900
H	2.65346500	-1.83065500	0.05437500
N	2.08379600	1.56456600	0.03208500
H	1.96153400	2.39362000	0.59961100
H	2.91233600	1.04954700	0.29998600

3-OH-4-OH-substituted

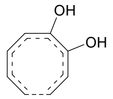

C	-2.03017900	-0.72298200	0.93271100
C	-0.90039800	-1.64127300	-0.85778600
C	-2.48001100	0.39667600	0.27838400
C	-1.64835100	1.42641800	-0.21304000
C	-0.27036800	1.54004200	-0.31427300
C	0.90779200	0.77488900	-0.05708400
C	1.19278900	-0.59125100	-0.01558900
C	0.34873700	-1.68946700	-0.29751500
H	-1.06775600	-0.70601200	1.42768500
H	-2.73424400	-1.47673000	1.27485000
H	-1.20266100	-0.78552400	-1.44652700
H	-1.44669600	-2.56432500	-1.02402500
H	-3.53717600	0.46904800	0.02871500
H	-2.17649300	2.29376300	-0.60469600
H	0.02469200	2.53236600	-0.66228600
H	0.70197300	-2.64782700	0.08687600
O	2.48157400	-0.87479000	0.36409000
H	2.67721200	-1.79637400	0.14787400
O	2.04835000	1.53779400	0.09056300
H	1.80168800	2.41926800	0.39946200

3-OH-4-F-substituted

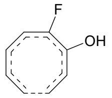

C	2.04369400	0.76600100	0.89937400
C	0.82495400	1.66176800	-0.85888500
C	2.49542600	-0.34064800	0.22845300
C	1.68030600	-1.41608100	-0.19345100
C	0.30507000	-1.56339100	-0.24478000
C	-0.86334700	-0.78332400	-0.04848700
C	-1.21800100	0.55920000	-0.00268800
C	-0.40535700	1.68877700	-0.26121300
H	1.10324200	0.72599400	1.43376400
H	2.73657900	1.54699100	1.20012200
H	1.12024200	0.81166300	-1.46027200
H	1.36428800	2.58850700	-1.02623400
H	3.54261900	-0.38206900	-0.06705800
H	2.21974800	-2.29224700	-0.54716800
H	-0.00754300	-2.57693100	-0.49248800
H	-0.77005000	2.63121000	0.14928200
O	-2.52012900	0.77745000	0.36276300
H	-2.77087100	1.68018400	0.12412900
F	-1.95041000	-1.59852300	0.07487700

3-OH-4-CHO-substituted

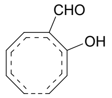

C	2.48609200	-0.03866300	0.86476000
C	1.73655800	1.17162400	-0.91916600
C	2.35033600	-1.25628900	0.24652600
C	1.12710900	-1.86638600	-0.11943600
C	-0.17130400	-1.39109900	-0.19366700
C	-0.91075800	-0.17645400	-0.03911000
C	-0.55068000	1.17928900	0.00770200
C	0.67834800	1.79822700	-0.31219400
H	1.66394600	0.37703000	1.43110100
H	3.47506600	0.34348600	1.10384700
H	1.57420900	0.25961300	-1.47860100
H	2.63201900	1.73669300	-1.15823500
H	3.25523800	-1.78018200	-0.06008100
H	1.21922400	-2.90802000	-0.42132500
H	-0.87520500	-2.19280600	-0.41215400
H	0.81371400	2.81565800	0.05607100
O	-1.54159600	2.02461600	0.44647400
H	-1.33084700	2.93401100	0.19358700
C	-2.39324800	-0.42447800	0.00313700
H	-3.03210300	0.46933200	0.02132000
O	-2.89715200	-1.52829500	-0.00982900

3-OH-4-CN-substituted

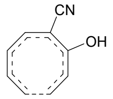

C	2.41186100	0.19272600	0.90761600
C	1.55634300	1.35432500	-0.86265700
C	2.48333500	-1.00810500	0.24789300
C	1.37523400	-1.78159400	-0.17054000
C	0.02190700	-1.50349500	-0.25049800
C	-0.86868300	-0.39077800	-0.07714000
C	-0.73240900	1.00296300	-0.01253400
C	0.40040000	1.80325000	-0.27805500
H	1.51754900	0.45643500	1.45601300
H	3.32189000	0.71250600	1.19456000
H	1.54711400	0.44937200	-1.45582400
H	2.36746500	2.05046600	-1.05207800
H	3.46402700	-1.37808400	-0.04818700
H	1.62390800	-2.78643000	-0.50520100
H	-0.55290900	-2.39203900	-0.50488700
H	0.36696900	2.82133600	0.10965300
C	-2.24565100	-0.83263700	0.01511400
N	-3.32589600	-1.24550400	0.07714900
O	-1.87154300	1.64726500	0.37486200
H	-1.81642200	2.58692500	0.15182100

3-OH-4-NO_2-substituted

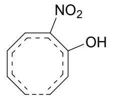

C	-2.72625000	0.00763200	-0.85644200
C	-1.90252300	1.37176600	0.83637300
C	-2.67315000	-1.14988400	-0.12627000
C	-1.48829700	-1.81790000	0.26553400
C	-0.16348700	-1.42233300	0.27574900
C	0.58360200	-0.23129500	0.06585000
C	0.36819100	1.13220800	-0.08168300
C	-0.81964500	1.86059800	0.15871600
H	-1.87470700	0.31677900	-1.44794400
H	-3.68480100	0.44201800	-1.12656000
H	-1.79902600	0.50753200	1.48066700
H	-2.77073400	2.00168600	1.00240500
H	-3.60703600	-1.57836200	0.23450300
H	-1.63632200	-2.82740800	0.64261800
H	0.50524900	-2.24439000	0.52494400
H	-0.88300100	2.83551100	-0.32291200
N	2.04615200	-0.56269700	0.02945200
O	2.74159400	-0.12049000	0.92257400
O	2.42902900	-1.29159300	-0.86591500
O	1.43910100	1.82112900	-0.58420200
H	1.47887000	2.70839500	-0.20050300

3-F-4-CH_3-substituted

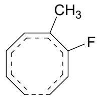

C	-2.10823700	-0.55373400	0.93408400
C	-1.08676900	-1.58800700	-0.85151400
C	-2.43738700	0.60182200	0.26962200
C	-1.51764800	1.56140200	-0.20934000
C	-0.13266200	1.57451600	-0.30030800
C	0.97528600	0.69882900	-0.07412300
C	1.05123300	-0.68493700	-0.03015100
C	0.15811500	-1.73807000	-0.29805000
H	-1.15809800	-0.63259200	1.44504800
H	-2.88897600	-1.23330700	1.26504200
H	-1.31945000	-0.70988400	-1.43781100
H	-1.70601500	-2.46347400	-1.02173000
H	-3.48151200	0.76880200	0.00890100
H	-1.97622400	2.47638500	-0.58072500
H	0.22610100	2.55911700	-0.59846400
H	0.47976300	-2.71174000	0.06457100
F	2.28077300	-1.15529700	0.34458200
C	2.31652000	1.39789500	0.10505400
H	2.69550800	1.24940500	1.12155600
H	3.07456400	1.00441900	-0.57832800
H	2.21667700	2.47224500	-0.06094600

3-F-4-NH_2-substituted

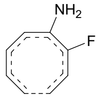

C	-2.03710700	-0.62598100	0.93460600
C	-1.01896600	-1.63145200	-0.84119200
C	-2.43660700	0.52447600	0.29710300
C	-1.56179200	1.51235400	-0.20657200
C	-0.18456200	1.56821200	-0.33786200
C	0.95490600	0.73852200	-0.07485100
C	1.09369400	-0.64613300	-0.03048700
C	0.24522800	-1.72460000	-0.31202100
H	-1.07529900	-0.65860600	1.43006800
H	-2.77864100	-1.33987900	1.28315700
H	-1.29589000	-0.77279900	-1.43717700
H	-1.59522900	-2.53757400	-0.99950600
H	-3.49138600	0.65062200	0.05984900
H	-2.05820100	2.40233300	-0.59003700
H	0.15962100	2.53956900	-0.69361700
H	0.61077200	-2.68373500	0.04902200
F	2.34927500	-1.05416700	0.35397600
N	2.14734700	1.47827700	0.03467900
H	2.06331400	2.31426000	0.59904100
H	2.95726900	0.93299000	0.29832600

3-F-4-OH-substituted

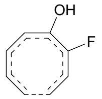

C	-2.01868800	-0.66265300	0.93873500
C	-0.94871900	-1.65157100	-0.85161000
C	-2.44190500	0.46619200	0.28203900
C	-1.58890800	1.47656500	-0.21487200
C	-0.21101800	1.56062700	-0.32534300
C	0.93619800	0.74975900	-0.06328800
C	1.14094700	-0.62352600	-0.02682400
C	0.30576600	-1.71894700	-0.30332900
H	-1.05518900	-0.67123500	1.43155800
H	-2.74159800	-1.39562600	1.28615800
H	-1.24183900	-0.79481900	-1.44281100
H	-1.51127500	-2.56624600	-1.00966800
H	-3.49748000	0.56295500	0.03508800
H	-2.10087000	2.35434900	-0.60499900
H	0.11399200	2.54131200	-0.67830800
H	0.68536400	-2.66902100	0.06669400
F	2.39017600	-0.98872700	0.35367200
O	2.11071000	1.45162500	0.09022100
H	1.90960000	2.34519800	0.39842100

3-F-4-F-substituted

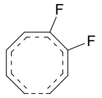

C	2.02588000	0.70892700	0.91368200
C	0.86337100	1.68267700	-0.85157900
C	2.46071700	-0.39901100	0.23429700
C	1.63023900	-1.45773000	-0.20268900
C	0.25480300	-1.58305900	-0.26411300
C	-0.88586900	-0.76496800	-0.05365000
C	-1.17103000	0.58772800	-0.01556300
C	-0.37465000	1.71929600	-0.26950300
H	1.07956900	0.68570700	1.43863400
H	2.73219900	1.47190900	1.22854800
H	1.15912400	0.83416500	-1.45409500
H	1.41309900	2.60533000	-1.00723800
H	3.50863900	-0.45643300	-0.05481400
H	2.15882900	-2.33849200	-0.56161900
H	-0.08303500	-2.58527200	-0.52356000
H	-0.77218500	2.65242500	0.12315900
F	-2.44154500	0.87905000	0.34663500
F	-2.00478800	-1.52710500	0.08288500

3-F-4-CHO-substituted

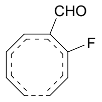

C	2.43880200	-0.09759500	0.90255600
C	1.78700800	1.18464000	-0.89297600
C	2.30564100	-1.29688900	0.25099200
C	1.08321000	-1.88373000	-0.15878400
C	-0.20654100	-1.39240300	-0.23790300
C	-0.90845600	-0.15987500	-0.04543800
C	-0.49812700	1.17019700	-0.01409600
C	0.71651700	1.81094500	-0.30730000
H	1.60363400	0.32276000	1.44637400
H	3.42427900	0.26575300	1.18098800
H	1.63706900	0.27716300	-1.46226800
H	2.69213900	1.74629100	-1.10188400
H	3.21130700	-1.82569600	-0.04331500
H	1.16949000	-2.91860100	-0.48481500
H	-0.92820300	-2.17136600	-0.47924700
H	0.80143700	2.83457800	0.04853800
F	-1.44734300	2.06189700	0.38918400
C	-2.39819100	-0.35627100	0.05268800
H	-3.00455500	0.55304500	0.17268000
O	-2.93746100	-1.43938900	-0.00976700

3-F-4-CN-substituted

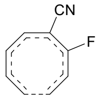

C	2.37352700	0.14320200	0.94202200
C	1.59275500	1.38177000	-0.83897500
C	2.44681500	-1.03984400	0.25340100
C	1.34045800	-1.79837500	-0.20090100
C	-0.00827200	-1.51290900	-0.29156700
C	-0.87330900	-0.38315700	-0.08976100
C	-0.68832800	0.99494900	-0.03326600
C	0.42342500	1.81851800	-0.27250700
H	1.46880000	0.41078100	1.47142600
H	3.28221700	0.64740900	1.25896400
H	1.60357500	0.48375400	-1.44165800
H	2.40696400	2.08211600	-0.99655000
H	3.42969500	-1.41064600	-0.03324300
H	1.59000500	-2.79710000	-0.55314300
H	-0.59559400	-2.38620400	-0.56802400
H	0.33681500	2.83320200	0.10736400
C	-2.26167300	-0.77887000	0.02738100
N	-3.35579600	-1.14668300	0.11498200
F	-1.78936600	1.69019600	0.33055800

3-F-4-NO_2-substituted

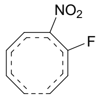

C	-2.69371800	-0.03794000	-0.89265600
C	-1.93974600	1.38562000	0.80960600
C	-2.64308600	-1.17143500	-0.12784800
C	-1.46019800	-1.81823700	0.30942800

C	-0.13887400	-1.41882600	0.33411600
C	0.60017500	-0.22701700	0.08594100
C	0.33618800	1.12266400	-0.05778400
C	-0.84008200	1.86512000	0.15374200
H	-1.83120600	0.27348700	-1.46707800
H	-3.65079600	0.37666300	-1.19645900
H	-1.85903700	0.52694300	1.46332700
H	-2.80866300	2.02169200	0.94543200
H	-3.58005800	-1.60003500	0.22419000
H	-1.61139700	-2.81721500	0.71258300
H	0.53306800	-2.22700500	0.61464600
H	-0.85961300	2.84312200	-0.32060200
N	2.06426900	-0.53390000	0.01638100
O	2.40454100	-1.41282400	-0.75464000
O	2.80659100	0.07874200	0.75665400
F	1.35608900	1.86806900	-0.53267600

3-CHO-4-CH_3-substituted

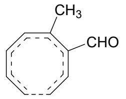

C	-1.95986400	-1.23162600	0.93285300
C	-0.79672600	-1.63749400	-1.06345300
C	-2.73434500	-0.22717000	0.41097300
C	-2.25407400	1.03228400	-0.00608100
C	-0.97769900	1.54648300	-0.20435200
C	0.39048000	1.12746700	-0.12048300
C	0.97776900	-0.14787500	-0.13060700
C	0.42268900	-1.40653900	-0.49260200
H	-1.00584400	-1.00579500	1.38887100
H	-2.40508200	-2.18707200	1.19745600
H	-1.35663300	-0.83858300	-1.53203000
H	-1.08606200	-2.65206400	-1.32260500
H	-3.78555400	-0.43057700	0.21446600
H	-3.03769400	1.74918600	-0.24780900
H	-1.03808000	2.60676700	-0.44370000
H	1.03553900	-2.26371100	-0.22229200
C	2.39463100	-0.26104100	0.33564000
H	2.85418000	0.64555200	0.76334100
O	3.03691900	-1.29067400	0.31802400
C	1.33893600	2.32871800	-0.04215000
H	1.60864000	2.56181200	0.99499800
H	2.26067900	2.18268800	-0.60848700
H	0.84977300	3.21794200	-0.44483000

3-CHO-4-NH_2-substituted

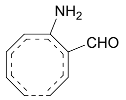

C	-1.88154100	-1.29892900	0.88383700
C	-0.74588300	-1.62203500	-1.12045600
C	-2.70412000	-0.28827600	0.44879200
C	-2.26812800	1.00046800	0.07955600
C	-1.01712400	1.54946600	-0.16551600

C	0.35824500	1.14320800	-0.13665000
C	0.98884500	-0.12217700	-0.13108200
C	0.47609600	-1.37343100	-0.55604400
H	-0.92800400	-1.06511000	1.33739900
H	-2.29443200	-2.27772400	1.11339200
H	-1.32841900	-0.82547500	-1.56705100
H	-1.01024600	-2.63601300	-1.40593200
H	-3.75503400	-0.50879900	0.27281600
H	-3.07502000	1.70698700	-0.11058000
H	-1.09497500	2.60864300	-0.41108900
H	1.11106700	-2.22072500	-0.30348700
C	2.35363500	-0.18569700	0.45087300
H	2.65949300	0.69204900	1.04715500
O	3.09184900	-1.14851000	0.36343900
N	1.24652200	2.22368000	-0.09510100
H	0.84158400	3.14704700	-0.03174200
H	2.05338600	2.18585600	-0.70254100

3-CHO-4-OH-substituted

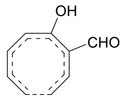

C	-1.86972500	-1.26920100	0.93319800
C	-0.69238000	-1.69147900	-1.01386500
C	-2.71442200	-0.32234300	0.40680400
C	-2.30041100	0.95220700	-0.03018100
C	-1.04691700	1.51037500	-0.23105600
C	0.32629200	1.12356800	-0.12074000
C	1.01259000	-0.09729800	-0.12861600
C	0.52362300	-1.38523500	-0.46701000
H	-0.93790100	-0.96477700	1.39155200
H	-2.24992000	-2.24478500	1.22326600
H	-1.28308800	-0.93548300	-1.51514700
H	-0.93407900	-2.72572900	-1.24095600
H	-3.75145900	-0.58894200	0.21427300
H	-3.11161100	1.62680700	-0.29935800
H	-1.11556200	2.55970600	-0.52332800
H	1.18611500	-2.19917700	-0.17902100
C	2.43833600	-0.07764100	0.31557200
H	2.82449200	0.88131300	0.69049300
O	3.14791300	-1.06334900	0.30832300
O	1.17806500	2.20704000	-0.05983900
H	0.70327100	2.98382400	0.26572300

3-CHO-4-F-substituted

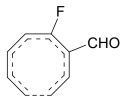

C	-1.91291500	-1.24897600	0.92802200
C	-0.65596200	-1.71395300	-0.98599900
C	-2.73327800	-0.31595600	0.34806000
C	-2.31199500	0.97027100	-0.05371200
C	-1.05127400	1.52503400	-0.19456600

C	0.29811500	1.09073600	-0.10655000
C	1.02016700	-0.09751600	-0.11878300
C	0.54487300	-1.40329900	-0.41432500
H	-0.99672500	-0.93701100	1.41290500
H	-2.29765900	-2.22513300	1.20918500
H	-1.23042400	-0.96232700	-1.51248300
H	-0.90520300	-2.75034900	-1.19366500
H	-3.75964500	-0.58993200	0.11251800
H	-3.11447300	1.65632800	-0.31855600
H	-1.06670900	2.59244100	-0.40590800
H	1.20658600	-2.20605700	-0.09594500
C	2.46203300	-0.02501700	0.27105800
H	2.85560700	0.96378700	0.54995000
O	3.17895700	-1.00246400	0.30906800
F	1.10204300	2.19889100	-0.02997600

3-CHO-4-CHO-substituted

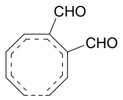

C	-2.37220800	-0.90256100	0.92864800
C	-1.28231800	-1.57317200	-1.11734800
C	-2.83490800	0.26262000	0.38578900
C	-2.03707300	1.37684200	0.02129900
C	-0.67360200	1.54052900	-0.14532100
C	0.51736600	0.74802000	-0.08694500
C	0.78277800	-0.63064800	-0.11768800
C	-0.06754200	-1.69998700	-0.50871500
H	-1.39316200	-0.95015500	1.38495200
H	-3.05703000	-1.71233200	1.16493600
H	-1.59648600	-0.63431600	-1.55527100
H	-1.84358400	-2.45850300	-1.40171600
H	-3.89660400	0.33936600	0.15654900
H	-2.60537400	2.27979300	-0.19589200
H	-0.41366200	2.57906700	-0.33974500
H	0.28606900	-2.69388300	-0.24468100
C	2.10305900	-1.10632200	0.42125100
H	2.69729700	-0.38340500	1.00710300
O	2.49344200	-2.24743800	0.31610500
C	1.74806000	1.62691000	-0.12870400
H	2.70391800	1.12016500	-0.34696900
O	1.73367700	2.83004000	-0.00896300

3-CHO-4-CN-substituted

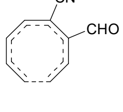

C	-2.34466900	-0.80289000	0.96254500
C	-1.30248100	-1.56309800	-1.01595100
C	-2.85464700	0.32130300	0.37136200
C	-2.08611500	1.43224300	-0.05041500
C	-0.72476100	1.61411800	-0.21338700
C	0.47457200	0.82317300	-0.11874600
C	0.78133700	-0.54389000	-0.13741900

C	-0.06351000	-1.64038800	-0.44574800
H	-1.36712300	-0.78115900	1.42578500
H	-2.99728300	-1.62245400	1.24960300
H	-1.63150200	-0.65877600	-1.51111600
H	-1.85237400	-2.47144300	-1.24460900
H	-3.91881200	0.35559800	0.14548100
H	-2.66760300	2.31476200	-0.31011000
H	-0.48029200	2.65060600	-0.43698100
H	0.32874300	-2.61263200	-0.15626400
C	1.62008000	1.71816300	-0.02754200
N	2.49174100	2.47821300	0.03454600
C	2.17829000	-0.94926100	0.24512800
H	2.89356600	-0.13728200	0.45692800
O	2.52274000	-2.10519400	0.34006200

3-CHO-4-NO_2-substituted

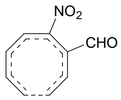

C	-1.70495100	1.50946700	1.03390900
C	-2.99720900	-0.65589900	-0.27989600
C	-2.03225000	-1.61437400	0.12029100
C	-0.65629700	-1.56185900	0.22844400
C	0.36012300	-0.56957100	0.10565900
C	0.46979200	0.81452300	0.06948200
C	-0.53571400	1.76932000	0.38364900
H	-1.77090400	0.66487300	-1.41824600
H	-3.52173200	1.19963800	-1.19571700
H	-1.86358500	0.57239700	1.55253500
H	-2.39196400	2.31866700	1.26342100
H	-4.02838600	-0.86809100	-0.00369300
H	-2.44561700	-2.57691100	0.41518600
H	-0.22377100	-2.53389100	0.45019300
H	-0.33473200	2.77931300	0.03531700
N	1.69967100	-1.25876000	0.07297400
O	2.57723800	-0.80958100	0.78924900
O	1.81975300	-2.22962500	-0.65089500
C	1.73790600	1.43199200	-0.46739800
H	2.45239800	0.76677600	-0.97836900
O	1.94269400	2.62132700	-0.41816800

3-CN-4-CH_3-substituted

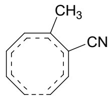

C	-2.10126400	-0.94783000	0.99007700
C	-1.03091800	-1.67219700	-0.94796700
C	-2.72171400	0.12190200	0.39691900
C	-2.05758400	1.26988800	-0.08634200
C	-0.71497000	1.57545100	-0.26455500
C	0.56598500	0.94843100	-0.13774900
C	0.94884100	-0.40021900	-0.13945600
C	0.22984200	-1.59649300	-0.42523700

H	-1.12249000	-0.82723000	1.43545800
H	-2.67987800	-1.80625700	1.31978200
H	-1.46566500	-0.82780900	-1.46486200
H	-1.47369900	-2.64470100	-1.14096100
H	-3.79111400	0.06340600	0.20255700
H	-2.72029400	2.08122700	-0.38352000
H	-0.59476700	2.62350900	-0.53357700
H	0.72282500	-2.51975600	-0.12717400
C	2.33567700	-0.65908800	0.18691500
N	3.43986500	-0.89118900	0.45121600
C	1.71356100	1.94417500	-0.00417600
H	2.18985500	1.84806200	0.97780600
H	2.49181800	1.77214700	-0.75413700
H	1.35961100	2.97160900	-0.10046800

3-CN-4-NH_2-substituted

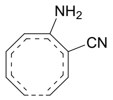

C	-2.02766400	-1.00128900	0.95395300
C	-1.01745800	-1.66284200	-0.94787100
C	-2.69637400	0.08926800	0.44360300
C	-2.05941500	1.25167000	-0.03149100
C	-0.73056100	1.57017000	-0.26791800
C	0.56419000	0.96038600	-0.15043300
C	0.97235600	-0.39460200	-0.14361500
C	0.26395600	-1.58019900	-0.46396400
H	-1.05462700	-0.86306700	1.40841100
H	-2.58207700	-1.87995100	1.27253900
H	-1.45814900	-0.82433500	-1.47023500
H	-1.44430000	-2.64007400	-1.15124300
H	-3.76955200	0.02017200	0.27944100
H	-2.73708400	2.05875700	-0.30577600
H	-0.61255200	2.60761700	-0.57931100
H	0.76814300	-2.50337600	-0.18421900
C	2.34970800	-0.61850100	0.21434800
N	3.46228800	-0.77496600	0.50377000
N	1.57722000	1.91428300	-0.11795700
H	1.37376800	2.77248500	0.37510700
H	2.52744500	1.59218700	0.01493300

3-CN-4-OH-substituted

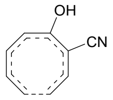

C	-2.01258300	-1.02343000	0.97518900
C	-0.92814200	-1.70073100	-0.94550200
C	-2.69952900	0.02517300	0.41429500
C	-2.09382500	1.20552000	-0.06392100
C	-0.76906200	1.55695700	-0.26680100
C	0.52523600	0.96325600	-0.13783400
C	1.00396800	-0.35210800	-0.14248000
C	0.33562200	-1.57113500	-0.43692500
H	-1.04081500	-0.85529300	1.42161300

H	-2.54312800	-1.91353200	1.30173500
H	-1.39780500	-0.88005100	-1.47096500
H	-1.32999200	-2.69178500	-1.13196400
H	-3.76655900	-0.08225500	0.23158000
H	-2.78991800	1.98908900	-0.35831200
H	-0.66707000	2.59825400	-0.57637100
H	0.86987300	-2.47051000	-0.13790700
C	2.39782500	-0.50947000	0.19966300
N	3.50396200	-0.71124600	0.47741900
O	1.54600400	1.87413900	-0.05612500
H	1.21259100	2.72750500	0.25355400

3-CN-4-F-substituted

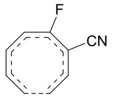

C	-2.05025700	-1.00127200	0.96615700
C	-0.89050600	-1.72667200	-0.92151500
C	-2.71425100	0.02950400	0.35305200
C	-2.09896400	1.22220100	-0.08724700
C	-0.76779700	1.57041100	-0.23107600
C	0.49664500	0.93414400	-0.12434800
C	1.01406900	-0.35661300	-0.12438000
C	0.35654100	-1.59284000	-0.38042100
H	-1.09326300	-0.82361100	1.44049900
H	-2.58641200	-1.89177900	1.28135600
H	-1.34210500	-0.91215600	-1.47247300
H	-1.30191600	-2.71693000	-1.08979900
H	-3.77206300	-0.08726200	0.12662000
H	-2.78462300	2.01669500	-0.37535300
H	-0.61188200	2.62270900	-0.46072800
H	0.88688500	-2.48154100	-0.04581300
C	2.41855600	-0.46548700	0.18903200
N	3.53880400	-0.61450700	0.44015700
F	1.47217000	1.87724100	-0.03565900

3-CN-4-CHO-substituted

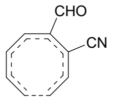

C	-2.43629100	-0.69505000	0.96575900
C	-1.47923000	-1.55731400	-1.01070700
C	-2.79004700	0.49096600	0.38213200
C	-1.88246700	1.49774600	-0.02955400
C	-0.50893500	1.50361100	-0.18796500
C	0.59196600	0.59483800	-0.09567000
C	0.70826800	-0.80103900	-0.13193800
C	-0.25451000	-1.79930800	-0.45510600
H	-1.46597200	-0.80779400	1.43092600
H	-3.19458700	-1.42109800	1.24576200
H	-1.68888000	-0.61217400	-1.49288900
H	-2.13681100	-2.38758700	-1.24944700
H	-3.84070600	0.66664900	0.15714500
H	-2.34851100	2.44994400	-0.27718700

H	-0.12661400	2.50224600	-0.39291000
H	0.00617400	-2.81923900	-0.18223000
C	1.98918000	-1.39435000	0.20688200
N	2.96924800	-1.94666400	0.48274500
C	1.90922300	1.33493500	-0.00503900
H	2.80900300	0.71026600	0.12038800
O	2.01490300	2.53690400	-0.07144200

3-CN-4-CN-substituted

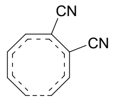

C	-2.40849300	-0.64779400	0.99817200
C	-1.45081700	-1.55889000	-0.96266900
C	-2.81102500	0.50263500	0.37671300
C	-1.94462000	1.52657600	-0.07742700
C	-0.57379700	1.57753500	-0.24537400
C	0.54777000	0.68426200	-0.13539600
C	0.71941400	-0.70664400	-0.13393300
C	-0.21494000	-1.74298300	-0.41138400
H	-1.42930100	-0.71111800	1.45460300
H	-3.13745100	-1.38889300	1.31381800
H	-1.69443500	-0.63896500	-1.47706200
H	-2.08678900	-2.41563300	-1.16304700
H	-3.86897600	0.63345500	0.15692300
H	-2.44345700	2.45282400	-0.35587400
H	-0.23097300	2.58057300	-0.49064000
H	0.08397400	-2.74268800	-0.10589200
C	1.78406100	1.43918100	-0.04426200
N	2.74228300	2.08517200	0.02278900
C	2.03910800	-1.20110000	0.19120900
N	3.07020800	-1.65749000	0.45339500

3-CN-4-NO_2-substituted

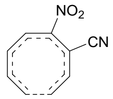

C	-2.67250000	0.51697400	-0.99321100
C	-1.84393500	1.47874200	0.99190600
C	-2.94890800	-0.69271400	-0.41412900
C	-1.97265500	-1.61817300	0.02194800
C	-0.60707300	-1.50084500	0.20487900
C	0.37083200	-0.47126200	0.10878600
C	0.42077900	0.91722200	0.15374200
C	-0.63319800	1.82590900	0.46533300
H	-1.70438500	0.70007700	-1.44131900
H	-3.47737200	1.18152100	-1.29394700
H	-1.98171500	0.52186700	1.47692800
H	-2.57735900	2.24898900	1.20933000
H	-3.98645500	-0.95065900	-0.21112100
H	-2.35522100	-2.60379200	0.27867500
H	-0.13267000	-2.44682400	0.45513700
H	-0.44816900	2.86378900	0.19994700
C	1.67440900	1.59073400	-0.10456000

N	2.62582500	2.22718300	-0.27437100
N	1.72556800	-1.10753500	-0.02239200
O	2.35099600	-0.86700000	-1.03310000
O	2.08364000	-1.83700400	0.88254300

3-NO_2-4-CH_3-substituted

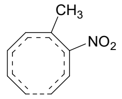

C	-2.24816400	-1.04380200	1.04149600
C	-1.17910000	-1.72176700	-0.91321600
C	-2.97390000	-0.04565900	0.44267200
C	-2.42983400	1.13583700	-0.10693200
C	-1.12987800	1.54852300	-0.36384800
C	0.20873300	1.05231000	-0.23840500
C	0.67256100	-0.25745300	-0.18742200
C	0.08811600	-1.52010100	-0.43976800
H	-1.26916100	-0.83459800	1.45134700
H	-2.74677500	-1.93301500	1.41775200
H	-1.70371000	-0.93543600	-1.43785800
H	-1.53676500	-2.73531800	-1.06584500
H	-4.04112500	-0.19835000	0.29204700
H	-3.17353200	1.87111900	-0.41030000
H	-1.11654800	2.58254700	-0.70425800
H	0.67769300	-2.38238800	-0.13926900
N	2.10053100	-0.41687000	0.20707600
O	2.79501100	-1.14972400	-0.47588000
O	2.48192300	0.16386600	1.20831000
C	1.24183300	2.17595200	-0.23595000
H	1.39940600	2.55467600	0.77824500
H	2.21228100	1.87075900	-0.63134800
H	0.87684200	3.00191700	-0.85124100

3-NO_2-4-NH_2-substituted

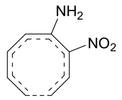

C	2.18522000	-1.21239500	-0.82218100
C	1.20574500	-1.48428800	1.15932600
C	2.94985000	-0.11111000	-0.49496900
C	2.41589400	1.14207900	-0.14981100
C	1.13293600	1.59340600	0.12843900
C	-0.23213300	1.12730700	0.13671900
C	-0.71722000	-0.20408800	0.23230500
C	-0.06854400	-1.38058200	0.65951600
H	1.22070200	-1.07049400	-1.29261700
H	2.66602100	-2.16784300	-1.01607200
H	1.71018300	-0.62196100	1.57490700
H	1.55926000	-2.45685400	1.48639400
H	4.02096200	-0.23779100	-0.35387000
H	3.16300100	1.92044100	-0.00225500
H	1.13506900	2.65896300	0.35386300
H	-0.60930300	-2.29775000	0.45036000

N	-2.10840000	-0.47599800	-0.17480500
O	-2.34520700	-1.55061500	-0.70107600
O	-2.98235200	0.36342800	0.02822000
N	-1.11417500	2.18030300	0.08358000
H	-0.81122800	3.02141600	-0.38333000
H	-2.10666600	1.99726800	0.10797700

3-NO_2-4-OH-substituted

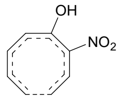

C	-2.23046800	-1.17096400	0.85884000
C	-1.16912900	-1.55129000	-1.06823200
C	-2.99062900	-0.10635200	0.42491300
C	-2.44955000	1.14103500	0.07050700
C	-1.14998600	1.58043000	-0.13697300
C	0.19905100	1.09853700	-0.12190600
C	0.72611200	-0.21601700	-0.19041000
C	0.08929100	-1.42841200	-0.54065400
H	-1.28997400	-0.98146500	1.36069600
H	-2.70128900	-2.12472700	1.08174800
H	-1.65213700	-0.71100700	-1.54956700
H	-1.52999500	-2.53855400	-1.33808200
H	-4.05142900	-0.25555800	0.23633300
H	-3.18639900	1.91998700	-0.11766100
H	-1.10018400	2.65218300	-0.31680600
H	0.62682400	-2.32995700	-0.26824300
N	2.14469600	-0.41713900	0.15354100
O	2.50988900	-1.52396800	0.49504900
O	2.93423100	0.53190100	0.07861200
O	1.02652000	2.16092700	-0.05344200
H	1.95844500	1.85638500	-0.10145900

3-NO_2-4-F-substituted

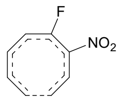

C	2.22897300	1.04624300	1.04695100
C	1.06912300	1.78012500	-0.86689000
C	2.97904000	0.09988900	0.40051700
C	2.46366000	-1.10415500	-0.13200200
C	1.16882100	-1.53617300	-0.35254800
C	-0.14482300	-1.01021200	-0.22986600
C	-0.72175400	0.24577800	-0.16397500
C	-0.17813800	1.53437500	-0.36813300
H	1.26766300	0.78308800	1.46988400
H	2.69227800	1.95129200	1.42922300
H	1.59675700	1.02208000	-1.43041400
H	1.41291500	2.80326800	-0.97900400
H	4.03291200	0.29952900	0.21798400
H	3.21472200	-1.82536300	-0.44829700
H	1.10079000	-2.57081200	-0.68286500
H	-0.78613300	2.36310900	-0.01433600

N	-2.15981700	0.27715700	0.20027200
O	-2.86849100	1.02832000	-0.44787200
O	-2.52747100	-0.39402500	1.14395100
F	-1.04832200	-2.01954200	-0.28189300C

3-NO_2-4-CHO-substituted

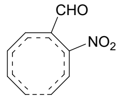

C	-2.49090900	-0.94551600	1.03757700
C	-1.46891400	-1.70784000	-1.01421200
C	-3.04391700	0.14927500	0.43653000
C	-2.32800700	1.27785000	-0.03900300
C	-0.98396800	1.50730200	-0.26663700
C	0.25044800	0.78734400	-0.19208600
C	0.54672800	-0.57094700	-0.19117000
C	-0.21189000	-1.72839400	-0.48502700
H	-1.49008300	-0.90348900	1.44658100
H	-3.11662700	-1.77027400	1.36682700
H	-1.85968700	-0.81826600	-1.48987700
H	-1.98991200	-2.64057000	-1.20599700
H	-4.11735100	0.15251800	0.25472800
H	-2.95489000	2.13171400	-0.28966000
H	-0.78602300	2.54400900	-0.52949300
H	0.22788000	-2.67744000	-0.19227500
N	1.92921800	-0.92666100	0.23871200
O	2.47332600	-1.85898800	-0.32558300
O	2.41786800	-0.28767200	1.15626200
C	1.44118900	1.72906300	-0.29097700
H	2.40331000	1.28520300	-0.59122400
O	1.34809200	2.92096000	-0.13199900

3-NO_2-4-CN-substituted

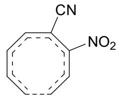

C	-2.52540100	-0.82214700	1.03191000
C	-1.50492400	-1.62191500	-0.97378300
C	-3.07255600	0.26546200	0.40996000
C	-2.34619900	1.37847800	-0.08182700
C	-0.99823400	1.59528800	-0.29171600
C	0.23002000	0.85414100	-0.17025100
C	0.53117300	-0.50393900	-0.16415700
C	-0.24546400	-1.64943600	-0.44732100
H	-1.53186600	-0.77068600	1.45778900
H	-3.15606400	-1.63951100	1.36979200
H	-1.87907200	-0.74072200	-1.47701200
H	-2.03251000	-2.55314100	-1.15451800
H	-4.14360100	0.26905700	0.21639300
H	-2.96116200	2.22984700	-0.36622500
H	-0.78981200	2.62149900	-0.58545600
H	0.18841900	-2.60055900	-0.15258400
C	1.35704400	1.77064900	-0.17505700
N	2.18314300	2.57794500	-0.24269700

N	1.92444500	-0.86804200	0.22150600
O	2.44505300	-1.77754300	-0.39941100
O	2.42992200	-0.27053100	1.15111300

3-NO_2-4-NO_2-substituted

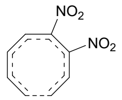

C	-2.80010900	-0.67479700	1.01078100
C	-1.79718800	-1.62231100	-1.02210600
C	-3.19601300	0.45237300	0.35339900
C	-2.33535500	1.47863800	-0.11812700
C	-0.96814400	1.54350200	-0.29120000
C	0.13601400	0.65118200	-0.17058500
C	0.31371300	-0.71692600	-0.14523000
C	-0.58031400	-1.77694000	-0.43171100
H	-1.81128200	-0.74940900	1.44391500
H	-3.53089700	-1.40825600	1.33949100
H	-2.06394300	-0.70432500	-1.52924900
H	-2.42296200	-2.48928000	-1.20846200
H	-4.25396000	0.57798400	0.13086500
H	-2.84524600	2.39280000	-0.41511000
H	-0.62979600	2.53803600	-0.56865900
H	-0.27172300	-2.76078400	-0.09046600
N	1.42087300	1.43856300	-0.20466600
O	2.29552600	1.03285500	-0.94494700
O	1.46968500	2.44798900	0.46646000
N	1.63279200	-1.21758900	0.34003300
O	2.13273700	-0.64279000	1.28831500
O	2.07936900	-2.20704100	-0.20498200

5.3.15 3,5-substituted

3-CH_3-5-CH_3-substituted

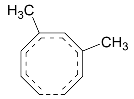

C	-0.53850200	2.03092900	-0.84205200
C	0.73503000	1.69945900	0.99192100
C	-1.70826400	1.53112500	-0.32065100
C	-1.95311100	0.17895200	0.00109700
C	-1.12364900	-0.93839900	0.04601900
C	0.28830800	-1.14675400	-0.09886800
C	1.48429100	-0.44036200	-0.00379500
C	1.69082500	0.89395800	0.42223500
H	0.12808100	1.37441200	-1.38525700
H	-0.45029700	3.09210000	-1.05917900
H	-0.12055900	1.24944600	1.47825000
H	0.99105000	2.70864600	1.30291600
H	-2.49159300	2.23519600	-0.04204000
H	-2.98999900	-0.02911400	0.25958100
H	2.66525600	1.32819400	0.19627000
C	2.75140000	-1.17996000	-0.40020500
H	2.54458800	-2.20411000	-0.71965500

H	3.25158700	-0.66667200	-1.23010400
H	3.46323600	-1.21904400	0.43159500
H	0.47891000	-2.20301100	-0.29892100
C	-1.85357400	-2.26589500	0.24014600
H	-2.92815900	-2.12470500	0.37557700
H	-1.71043900	-2.91176900	-0.63456400
H	-1.46817300	-2.80789300	1.11044900

3-CH_3-5-NH_2-substituted

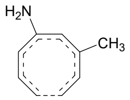

C	0.70311500	1.96527900	0.85188100
C	-0.57877400	1.73035300	-0.99585300
C	1.82975900	1.36422100	0.33866000
C	1.96450700	0.01040600	-0.02067800
C	1.04583100	-1.04117300	-0.04466800
C	-0.37750200	-1.13629600	0.13496100
C	-1.50767400	-0.33982900	-0.00299700
C	-1.60888600	0.99837900	-0.45584200
H	-0.02667500	1.37182300	1.38626900
H	0.71302400	3.02885900	1.07298700
H	0.25208700	1.21822600	-1.46380000
H	-0.75423900	2.75406500	-1.31575100
H	2.67070100	2.00183700	0.06783100
H	2.97137700	-0.27587200	-0.32283000
H	-2.55342000	1.50373900	-0.25445100
C	-2.82977600	-0.96627100	0.40815300
H	-2.70700700	-1.99474300	0.75520200
H	-3.28944000	-0.39243600	1.22156400
H	-3.53903200	-0.96832600	-0.42675200
H	-0.62794600	-2.16078200	0.41091600
N	1.61525200	-2.33101100	-0.17704600
H	2.61172700	-2.33587100	-0.35364900
H	1.12848000	-2.94385900	-0.81991300

3-CH_3-5-OH-substituted

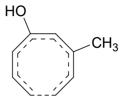

C	0.82146000	1.91718000	0.85997100
C	-0.51833800	1.75446900	-0.97466900
C	1.90410500	1.26888100	0.31674900
C	1.96608600	-0.09582700	-0.02915600
C	0.98682600	-1.08312100	-0.06157500
C	-0.43458600	-1.13969300	0.08556300
C	-1.52674600	-0.28713800	-0.00404600
C	-1.56555200	1.06952800	-0.41100800
H	0.07267300	1.35540000	1.40234400
H	0.88062300	2.97929700	1.07957900
H	0.27646200	1.20753100	-1.46567100
H	-0.64600200	2.79126400	-1.27346400
H	2.76859800	1.86451100	0.02652800

H	2.95397900	-0.44309600	-0.33355600
H	-2.47761800	1.61781100	-0.17512200
C	-2.87132700	-0.86632000	0.39953000
H	-3.25933300	-0.34830800	1.28499500
H	-3.60977500	-0.74083500	-0.39917300
H	-2.80316700	-1.92969600	0.63922300
H	-0.71325600	-2.17379500	0.28043400
O	1.44777100	-2.37506600	-0.25545600
H	2.40307700	-2.40730400	-0.11061900

3-CH_3-5-F-substituted

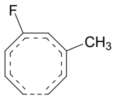

C	0.98188000	1.85174400	0.84959000
C	-0.41088200	1.76949500	-0.97627100
C	2.01463000	1.13426800	0.30056600
C	1.97536600	-0.24100900	-0.01286500
C	0.90802000	-1.11409000	-0.05946100
C	-0.50887600	-1.11815600	0.06484900
C	-1.54693000	-0.19977500	0.00333800
C	-1.48900000	1.16286300	-0.38571400
H	0.20293500	1.34028700	1.39948900
H	1.10306700	2.91128200	1.05577500
H	0.33863200	1.16823600	-1.47535600
H	-0.46568300	2.81387000	-1.26986000
H	2.91689600	1.66483800	0.00110400
H	2.91903500	-0.71394500	-0.27300400
H	-2.35425100	1.77448800	-0.12985500
C	-2.92413500	-0.69622800	0.40251000
H	-3.27909800	-0.15983100	1.29070700
H	-3.65303100	-0.52146700	-0.39561600
H	-2.92135200	-1.76286800	0.63735400
H	-0.83849000	-2.14223500	0.23306300
F	1.33676700	-2.40748000	-0.24367200

3-CH_3-5-CHO-substituted

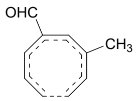

C	-0.62463900	2.15578300	0.84460800
C	-1.62453400	1.27792100	-1.07218800
C	0.65357900	2.16788000	0.34595100
C	1.42803000	1.01933400	0.08409300
C	1.09540000	-0.33010600	0.05578300
C	-0.10228100	-1.11496100	0.15253100
C	-1.47153900	-0.95745600	0.00214000
C	-2.17865700	0.18595200	-0.46242600
H	-0.99290700	1.28204400	1.36538500
H	-1.15263400	3.08787700	1.02658900
H	-0.63564000	1.21701400	-1.50673000
H	-2.25689200	2.09192000	-1.41512500
H	1.09681600	3.12329200	0.07008500

H	2.47951900	1.19944300	-0.13434000
H	-3.25026900	0.19957700	-0.26001500
C	-2.35307900	-2.13823100	0.36872400
H	-3.03622000	-1.86779900	1.18283400
H	-2.96943500	-2.45052700	-0.48106900
H	-1.76706200	-2.99893100	0.69906100
H	0.15150900	-2.15217400	0.37925500
C	2.30639100	-1.21664700	-0.07438900
H	2.10556300	-2.30056000	0.05114900
O	3.43195300	-0.84099900	-0.30575500

3-CH_3-5-CN-substituted

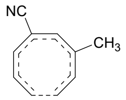

C	0.91884500	2.06110100	-0.82540900
C	1.75743800	1.01831100	1.04905900
C	-0.32612200	2.31885300	-0.30780700
C	-1.30221900	1.33935500	-0.03597800
C	-1.21651000	-0.05265700	-0.01613400
C	-0.19382100	-1.05926200	-0.13628100
C	1.18462200	-1.14941300	-0.01505100
C	2.09677900	-0.15769200	0.43491600
H	1.09570700	1.14195400	-1.36772600
H	1.61399700	2.87470700	-1.01247600
H	0.78550400	1.12748500	1.51192300
H	2.53162200	1.70351600	1.38257400
H	-0.57265700	3.33770500	-0.01553400
H	-2.29110000	1.72527800	0.20109100
H	3.14880800	-0.33835200	0.21294400
C	1.82302400	-2.47249700	-0.39707000
H	2.56713100	-2.32340100	-1.18815700
H	2.34308500	-2.91865700	0.45739000
H	1.08729900	-3.19193300	-0.76242600
H	-0.64954100	-2.02451400	-0.35075200
C	-2.52540700	-0.67041600	0.12447000
N	-3.56566000	-1.16684000	0.24040700

3-CH_3-5-NO_2-substituted

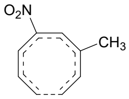

C	1.43595200	2.03939700	-0.77361800
C	2.14994500	0.88751100	1.08490600
C	0.20838600	2.38902700	-0.26820900
C	-0.86167100	1.50000800	-0.04448000
C	-0.88713100	0.11684500	-0.04438400
C	0.02261800	-0.97422800	-0.17444300
C	1.38687200	-1.18574500	-0.05199900
C	2.38126300	-0.29553000	0.43506800
H	1.54316300	1.12698600	-1.34479000
H	2.20072600	2.79633800	-0.92260400
H	1.18984400	1.07538700	1.54715300

H	2.98367800	1.48549500	1.44189600
H	0.05113400	3.41469200	0.05928500
H	-1.81698300	1.96142600	0.18087100
H	3.41402300	-0.56392600	0.21076600
C	1.91119400	-2.54065600	-0.49245000
H	2.61381600	-2.42702800	-1.32667000
H	2.45045900	-3.03620700	0.32152500
H	1.10674400	-3.20187700	-0.82084600
H	-0.51744900	-1.88357800	-0.41719400
N	-2.30838800	-0.40060300	0.09251600
O	-2.46368000	-1.56193500	0.42694300
O	-3.22944600	0.36652600	-0.12686200

3- NH_2-5-CH_3-substituted

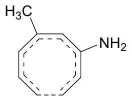

C	-0.40828300	2.07852500	-0.82354700
C	0.86271500	1.63403300	0.96716300
C	-1.59607300	1.63010100	-0.29122700
C	-1.91747000	0.29284100	0.01734600
C	-1.15750900	-0.87813600	0.04205500
C	0.23050300	-1.18185600	-0.07982900
C	1.47127400	-0.53859800	-0.02053500
C	1.76989900	0.78411800	0.38362100
H	0.21353700	1.40157100	-1.39370200
H	-0.27619300	3.13710700	-1.03169300
H	-0.00016800	1.22584500	1.47825500
H	1.17400900	2.62965800	1.26972200
H	-2.33648500	2.37274500	0.00587200
H	-2.96096500	0.14526000	0.28862800
H	2.74765300	1.16563200	0.09260500
N	2.58986400	-1.27606300	-0.46350600
H	2.40680200	-2.25587500	-0.64125000
H	3.42060700	-1.15936200	0.10417500
H	0.36520700	-2.25539100	-0.23356700
C	-1.97737100	-2.15481500	0.22386900
H	-3.04312300	-1.94331100	0.33307200
H	-1.85713700	-2.81177400	-0.64588700
H	-1.64889500	-2.71694300	1.10480600

3- NH_2-5-NH_2-substituted

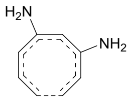

C	-0.60529200	2.01018300	-0.83567400
C	0.68255800	1.68726500	0.97334800
C	-1.74882300	1.44488600	-0.31324900
C	-1.94010000	0.09792300	0.03773100
C	-1.07034600	-1.00028900	0.04069300
C	0.33879700	-1.17125800	-0.11863500
C	1.50803600	-0.41549300	-0.01338600
C	1.68070300	0.92067300	0.42248200
H	0.09094400	1.39903000	-1.39434400

H	-0.58627400	3.07473200	-1.05249300
H	-0.15356700	1.20490600	1.46407600
H	0.89987000	2.70518300	1.28436200
H	-2.56008800	2.11435000	-0.02743400
H	-2.95248100	-0.14326300	0.35864400
H	2.62658100	1.39005600	0.15684400
N	2.69097200	-1.03061000	-0.47074000
H	2.61316300	-2.02269700	-0.65584800
H	3.51293700	-0.82373800	0.08326500
H	0.55112600	-2.21361000	-0.36172700
N	-1.71054500	-2.25885800	0.16295200
H	-2.70845400	-2.20716300	0.32266500
H	-1.26994500	-2.89484500	0.81664900

3- NH_2-5-OH-substituted

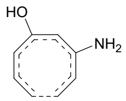

C	-0.76301200	1.95597600	-0.84229500
C	0.59406600	1.72452000	0.94709100
C	-1.85225600	1.32263200	-0.28800500
C	-1.94585300	-0.03873800	0.05250600
C	-0.99364300	-1.05980100	0.05706400
C	0.41580700	-1.16749800	-0.07411300
C	1.53688400	-0.33725500	-0.01514200
C	1.62457600	1.02160000	0.37509000
H	-0.03889100	1.38686600	-1.40988900
H	-0.81246500	3.01939200	-1.05875100
H	-0.19734300	1.19282200	1.46055600
H	0.74603100	2.75977000	1.23821800
H	-2.69931300	1.93617400	0.01754800
H	-2.93354500	-0.35948100	0.38392100
H	2.53139000	1.54792700	0.08143300
N	2.74841600	-0.89657300	-0.45695700
H	2.73061000	-1.89792700	-0.60351000
H	3.56419600	-0.61362300	0.07174700
H	0.67213700	-2.21410300	-0.23410700
O	-1.50529300	-2.33555900	0.23743900
H	-2.45880500	-2.32595100	0.07884900

3- NH_2-5-F-substituted

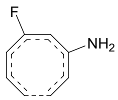

C	-0.94272300	1.88947800	-0.82475300
C	0.46113100	1.74588600	0.95003400
C	-1.97851500	1.16931500	-0.27678100
C	-1.95765300	-0.20564300	0.02619900
C	-0.90352300	-1.10246800	0.05121200
C	0.50491200	-1.14210900	-0.05399800
C	1.56206800	-0.23144800	-0.02151800
C	1.53512900	1.13428200	0.35536500
H	-0.18164000	1.38399900	-1.40414700

H	-1.06747700	2.95071900	-1.02082200
H	-0.27813700	1.14962300	1.47064600
H	0.52721000	2.79010000	1.24032700
H	-2.87301600	1.70661800	0.03536800
H	-2.90323300	-0.66313700	0.30467700
H	2.39090300	1.73425400	0.05003800
N	2.80997300	-0.69852800	-0.46230000
H	2.86976200	-1.69668600	-0.61826400
H	3.60592700	-0.35313800	0.05921400
H	0.82462900	-2.17526100	-0.18361300
F	-1.36329900	-2.38679500	0.22979100

3- NH_2-5-CHO-substituted

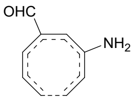

C	0.71860900	2.14491600	-0.82015900
C	1.68745400	1.19445800	1.03434200
C	-0.55915200	2.18297500	-0.31428100
C	-1.37268800	1.06159200	-0.06439800
C	-1.09174100	-0.30300200	-0.05122400
C	0.06153300	-1.14034800	-0.12599100
C	1.44700100	-1.02751600	-0.01649000
C	2.20909200	0.08877500	0.41923200
H	1.05446900	1.27947600	-1.37523300
H	1.26435100	3.06872800	-0.99277100
H	0.71204900	1.14907700	1.50112100
H	2.34498500	1.99354000	1.36349900
H	-0.96920500	3.14763100	-0.01826200
H	-2.41441600	1.27792100	0.16448000
H	3.26574200	0.07653100	0.15489700
N	2.21983500	-2.12903600	-0.43165000
H	1.69064100	-2.96252900	-0.65597700
H	3.00563400	-2.34682100	0.16926000
H	-0.23038600	-2.17823000	-0.30212500
C	-2.34073800	-1.13964200	0.07541300
H	-2.18053400	-2.23319500	-0.02660700
O	-3.45479900	-0.71776700	0.27757600

3-NH_2-5-CN-substituted

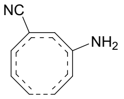

C	1.05005100	2.01343500	-0.80074600
C	1.81985300	0.89712300	1.01164500
C	-0.18618400	2.32260400	-0.28189200
C	-1.21289800	1.39658400	-0.02216900
C	-1.20473800	-0.00230900	-0.01546500
C	-0.25566100	-1.06794400	-0.10789900
C	1.12845200	-1.23173400	-0.02561800
C	2.10491100	-0.29459300	0.39735800
H	1.17884800	1.10534000	-1.37417600
H	1.77670000	2.80102200	-0.97987400

H	0.86688300	1.03665600	1.50575000
H	2.62844500	1.54698900	1.33261800
H	-0.38087100	3.34901600	0.02454800
H	-2.17842400	1.83213200	0.22298400
H	3.13372500	-0.51698300	0.11806700
N	1.64870900	-2.46227000	-0.45879400
H	0.96031800	-3.17256200	-0.67416700
H	2.39966900	-2.83456100	0.10930500
H	-0.76394200	-2.01715900	-0.27167400
C	-2.55031300	-0.54076600	0.11733300
N	-3.61759200	-0.97834200	0.22327200

3-NH_2-5-NO_2-substituted

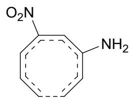

C	1.51785500	1.99411400	-0.75411500
C	2.19164300	0.79811600	1.03779300
C	0.29742300	2.36921900	-0.24016100
C	-0.79729700	1.51498800	-0.01756100
C	-0.87009400	0.12971100	-0.04091500
C	-0.01312600	-0.99204600	-0.15140900
C	1.35536800	-1.24837300	-0.06364200
C	2.39153600	-0.39528000	0.39342900
H	1.59475200	1.09828400	-1.35560200
H	2.29421300	2.73949500	-0.90264200
H	1.25072400	0.99544800	1.53485900
H	3.04530200	1.37632400	1.37881900
H	0.17278700	3.39474600	0.10270500
H	-1.73213400	2.00424500	0.23039300
H	3.40404400	-0.68283200	0.11363500
N	1.79476100	-2.49428900	-0.54186700
H	1.05920300	-3.15423100	-0.76211600
H	2.52583600	-2.92874100	0.00787200
H	-0.58585400	-1.89272400	-0.34921500
N	-2.31635900	-0.33456100	0.08752100
O	-3.20264800	0.47240100	-0.12670200
O	-2.52454500	-1.49374500	0.40185100

3-OH-5-CH_3-substituted

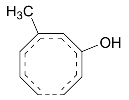

C	-0.23649900	2.09359300	-0.83561000
C	1.04696300	1.54783800	0.97004100
C	-1.44193200	1.75374300	-0.27220300
C	-1.87839800	0.44803900	0.04543500
C	-1.23078500	-0.78460400	0.04386300
C	0.12548700	-1.20123400	-0.11817600
C	1.40384700	-0.66651800	-0.03402600
C	1.85249200	0.60976300	0.37728000
H	0.32594700	1.35511800	-1.39117500
H	-0.01467400	3.13441300	-1.05536900
H	0.14644400	1.23475800	1.48208800

H	1.45888000	2.51000400	1.25946100
H	-2.10862600	2.55970300	0.03362200
H	-2.92636600	0.39590600	0.33434700
H	2.86551600	0.88871100	0.08582900
O	2.37570700	-1.56009100	-0.43796200
H	3.23989800	-1.26837000	-0.11783000
H	0.19873800	-2.27104300	-0.31719500
C	-2.14958900	-1.98989700	0.22386700
H	-3.18904800	-1.69132200	0.37582400
H	-2.11232300	-2.62935900	-0.66596500
H	-1.83955200	-2.60211200	1.07722400

3-OH-5-NH_2-substituted

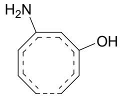

C	-0.47633100	2.03908600	-0.84837300
C	0.84269100	1.63155200	0.97848000
C	-1.63738000	1.55616000	-0.29217800
C	-1.91945100	0.22411400	0.06743400
C	-1.13681100	-0.93434800	0.04091700
C	0.25605400	-1.19475000	-0.15878100
C	1.46089900	-0.52418200	-0.02813500
C	1.76266500	0.78634100	0.41337400
H	0.17965100	1.37648100	-1.39748600
H	-0.38716800	3.09853900	-1.07142500
H	-0.03382900	1.22203800	1.46418200
H	1.14300400	2.63031500	1.28078600
H	-2.39402200	2.28158700	0.00668300
H	-2.94145300	0.05700300	0.40502900
H	2.74575100	1.17447500	0.14614900
O	2.52841000	-1.29296300	-0.44091000
H	3.35448200	-0.87990700	-0.15570100
H	0.42885700	-2.23335300	-0.43780600
N	-1.85402700	-2.14316900	0.16207000
H	-2.83860400	-2.03895000	0.36920600
H	-1.41976800	-2.84618100	0.74674600

3-OH-5-OH-substituted

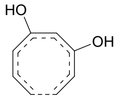

C	-0.63327700	1.99669300	-0.85137500
C	0.75563900	1.67721500	0.95626500
C	-1.74568300	1.44097200	-0.26931800
C	-1.93337700	0.08606600	0.07238800
C	-1.06382500	-1.00168900	0.05533500
C	0.33503800	-1.20156200	-0.10942500
C	1.49518200	-0.44943300	-0.02835600
C	1.71175600	0.89248000	0.36765700
H	0.05405900	1.37795700	-1.41314800
H	-0.61285900	3.06060700	-1.06907000
H	-0.07890600	1.21435400	1.46740000

H	0.99084500	2.69970000	1.23543600
H	-2.54226000	2.11213200	0.05044500
H	-2.93966300	-0.16122700	0.41081400
H	2.65797800	1.34232500	0.06649400
O	2.60365100	-1.15813400	-0.43157800
H	3.40543400	-0.70422100	-0.13862300
H	0.55116800	-2.24909600	-0.30657100
O	-1.65420200	-2.23846200	0.23506200
H	-2.61012100	-2.16421300	0.10991400

3-OH-5-F-substituted

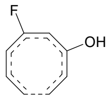

C	-0.82432800	1.93336500	-0.83681500
C	0.61590600	1.71530400	0.95890500
C	-1.88817800	1.28825500	-0.25928000
C	-1.95629600	-0.08762500	0.04938400
C	-0.97099100	-1.05471500	0.05068100
C	0.43176700	-1.17962300	-0.09371000
C	1.53000700	-0.33790700	-0.03574300
C	1.62691000	1.02102600	0.35045700
H	-0.09217800	1.37311500	-1.40344400
H	-0.88051400	2.99873700	-1.04070200
H	-0.17164800	1.18148900	1.47520400
H	0.76288300	2.75395100	1.23812100
H	-2.74026600	1.88450500	0.06369900
H	-2.92798900	-0.47721700	0.34119100
H	2.52706800	1.55283600	0.04197100
O	2.68979000	-0.95624700	-0.43809300
H	3.45480700	-0.44470200	-0.14116100
H	0.71531200	-2.21487700	-0.26959400
F	-1.50606300	-2.30514900	0.23290900

3-OH-5-CHO-substituted

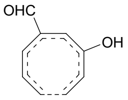

C	0.75925400	2.12043400	-0.83060600
C	1.75193500	1.13308300	1.04277600
C	-0.50363800	2.18860900	-0.29988000
C	-1.34533000	1.08501300	-0.04524500
C	-1.10190500	-0.28348800	-0.05700700
C	0.03387100	-1.14172400	-0.16754800
C	1.40885700	-1.05725000	-0.02963800
C	2.22507700	0.01173600	0.42046700
H	1.07335200	1.23753400	-1.37130600
H	1.32635200	3.02895400	-1.01413200
H	0.77261100	1.12866900	1.50252300
H	2.43973900	1.90785200	1.36729400
H	-0.88589100	3.16232100	0.00288500
H	-2.37870700	1.32719400	0.19623700
H	3.28309400	-0.04348600	0.16214500

O	2.05711400	-2.21061700	-0.41505700
H	2.94860600	-2.22934200	-0.04168800
H	-0.24704900	-2.17212100	-0.38872100
C	-2.36679600	-1.09743500	0.06465800
H	-2.23133300	-2.18940600	-0.07320600
O	-3.46570400	-0.65338800	0.29882000

3-OH-5-CN-substituted

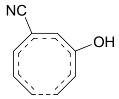

C	1.07551600	1.99044200	-0.81251700
C	1.87760700	0.83403600	1.01664800
C	-0.13875500	2.31312400	-0.26076700
C	-1.18471900	1.40453300	0.00335400
C	-1.21159600	0.00963300	-0.01679800
C	-0.27838000	-1.06780000	-0.14763200
C	1.09138700	-1.24721500	-0.03948800
C	2.10916800	-0.35976800	0.38897400
H	1.18616500	1.07350900	-1.37607200
H	1.81372200	2.76536900	-0.99847000
H	0.93355400	1.01256000	1.51417200
H	2.71252400	1.45707200	1.32205700
H	-0.30830000	3.33944500	0.06027300
H	-2.13644900	1.86062200	0.26497800
H	3.12909800	-0.61819400	0.10357200
O	1.49105600	-2.50067700	-0.44021000
H	2.36791100	-2.69978200	-0.08467300
H	-0.76969800	-2.01574700	-0.35626200
C	-2.56575600	-0.50472800	0.11721100
N	-3.64340200	-0.91471100	0.22545500

3-OH-5-NO_2-substituted

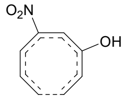

C	1.54302000	1.90567200	-0.90008200
C	2.23404500	0.79563200	1.00008900
C	0.34387100	2.33402400	-0.38859900
C	-0.76527400	1.51620100	-0.08755100
C	-0.86771600	0.13697000	-0.04950900
C	-0.03576000	-1.00698400	-0.12240300
C	1.31510200	-1.27593200	0.03043600
C	2.38577000	-0.44332800	0.43844200
H	1.60640900	0.95739700	-1.41717400
H	2.33037500	2.62402900	-1.11059700
H	1.29997900	1.07009500	1.47189300
H	3.10890900	1.36926900	1.29012300
H	0.23923400	3.38437400	-0.12316700
H	-1.68372400	2.03643600	0.16107600
H	3.38898000	-0.78937500	0.18870100
O	1.63106100	-2.57143700	-0.30342000
H	2.48976600	-2.81177100	0.07029100
H	-0.59722000	-1.91343100	-0.32696400
N	-2.32205000	-0.28820700	0.08363000

O	-2.66275800	-1.33472200	-0.43390500
O	-3.07414100	0.44577100	0.69800900

3-F-5-CH_3-substituted

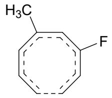

C	0.01592200	2.08725100	-0.83815000
C	1.25121100	1.43657900	0.96320900
C	-1.21944900	1.88838000	-0.27116200
C	-1.80721700	0.64331100	0.04751100
C	-1.31221500	-0.65621900	0.04667100
C	-0.00681800	-1.21044300	-0.12981700
C	1.30441100	-0.79474400	-0.02372100
C	1.93130000	0.39495000	0.38314000
H	0.48955200	1.29175200	-1.39782200
H	0.35288100	3.09622200	-1.05945200
H	0.32132400	1.24765500	1.48225900
H	1.78508300	2.34030400	1.24102400
H	-1.78668600	2.76651500	0.03545400
H	-2.85394000	0.71595400	0.33735000
H	2.97752100	0.49782300	0.10645700
F	2.19965400	-1.75221400	-0.40952200
H	-0.03588300	-2.27772000	-0.35225500
C	-2.35544500	-1.75257400	0.23377200
H	-2.37848800	-2.40708100	-0.64545700
H	-2.12075800	-2.37961600	1.10031600
H	-3.35769500	-1.34083700	0.36911300

3-F-5-NH_2-substituted

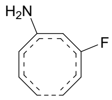

C	-0.26791600	2.05493700	-0.85343500
C	1.02499100	1.56548500	0.97507200
C	-1.46791600	1.68846200	-0.29175400
C	-1.87975900	0.38977200	0.06970000
C	-1.21733000	-0.83834300	0.04580300
C	0.14827700	-1.21569600	-0.16682600
C	1.38733900	-0.63366800	-0.01831000
C	1.84939500	0.62086200	0.41687800
H	0.32041600	1.33096100	-1.40132500
H	-0.07699100	3.09993300	-1.07966600
H	0.11288900	1.25872800	1.47008800
H	1.43203500	2.52893500	1.26720400
H	-2.14830200	2.48466300	0.00908000
H	-2.91305200	0.32487800	0.40810700
H	2.87843200	0.85841800	0.16085000
F	2.40396600	-1.45270900	-0.41876100
H	0.24035600	-2.25893800	-0.46555700
N	-2.03720100	-1.97830700	0.17144900
H	-3.01081200	-1.78598400	0.36823600
H	-1.67274600	-2.70992800	0.76890800

3-F-5-OH-substituted

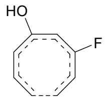

C	-0.46562500	2.02131300	-0.85603000
C	0.91232300	1.63754800	0.95061000
C	-1.61505300	1.55694300	-0.26693500
C	-1.91146300	0.22007800	0.07515800
C	-1.13440700	-0.93223700	0.05898000
C	0.24864900	-1.22411300	-0.12058300
C	1.43727300	-0.53747800	-0.02019800
C	1.79608600	0.76544600	0.36967000
H	0.16896000	1.35090400	-1.42059700
H	-0.36145000	3.08002600	-1.07447100
H	0.04227700	1.25731600	1.46992700
H	1.23795600	2.63746800	1.22040600
H	-2.35231100	2.28992500	0.05847300
H	-2.93487700	0.05505200	0.41287700
H	2.79312400	1.08791200	0.08180200
F	2.51154200	-1.28326600	-0.40398000
H	0.40340800	-2.27870700	-0.33692200
O	-1.80635900	-2.12369700	0.24381200
H	-2.75679600	-1.98592700	0.12979200

3-F-5-F-substituted

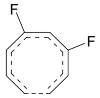

C	-0.69058500	1.96359700	-0.84221200
C	0.74914900	1.69933600	0.95286500
C	-1.78899100	1.39005300	-0.25479300
C	-1.94681100	0.02040100	0.05391600
C	-1.02933700	-1.00797800	0.05478200
C	0.36575900	-1.20495700	-0.10434100
C	1.48575000	-0.40896000	-0.02815600
C	1.70783500	0.92799700	0.35064600
H	0.00074000	1.35809700	-1.41379200
H	-0.67980000	3.02996000	-1.04749500
H	-0.07286600	1.22843000	1.47605000
H	0.97067700	2.72726000	1.22221200
H	-2.59677700	2.04095300	0.07563900
H	-2.94182300	-0.30421700	0.34730800
H	2.66260200	1.35303100	0.05185000
F	2.62426800	-1.04930300	-0.40842500
H	0.60395200	-2.24812700	-0.29935100
F	-1.63130400	-2.22428800	0.24079500

3-F-5-CHO-substituted

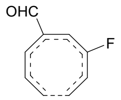

C	0.84266700	2.06444900	-0.83809200
C	1.84390000	1.07640700	1.03146200
C	-0.41112000	2.18815200	-0.29521200
C	-1.29947100	1.12247000	-0.03647800
C	-1.11763400	-0.25423500	-0.05372100
C	-0.00664100	-1.14398200	-0.18007000
C	1.35786000	-1.08009700	-0.02167500
C	2.24748500	-0.07905600	0.42089800
H	1.11369200	1.16899000	-1.38153800
H	1.44619100	2.94742900	-1.02909900
H	0.87305100	1.13517100	1.50379900
H	2.58020500	1.81183700	1.34105500
H	-0.74709100	3.17720900	0.01153700
H	-2.32015000	1.40901100	0.21178800
H	3.29431700	-0.23901800	0.17380300
F	2.00058300	-2.23085600	-0.37163600
H	-0.30760100	-2.16435300	-0.42106300
C	-2.40870500	-1.02152500	0.06858600
H	-2.31357300	-2.11767000	-0.07093200
O	-3.48929000	-0.53579900	0.30389700

3-F-5-CN-substituted

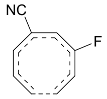

C	1.16532900	1.91591800	-0.81980900
C	1.95532700	0.74914500	1.01370100
C	-0.02896400	2.29535300	-0.26038100
C	-1.11919200	1.44070700	0.00797100
C	-1.21901400	0.05100300	-0.01282400
C	-0.32287100	-1.05746100	-0.16059400
C	1.03151300	-1.26271500	-0.03023000
C	2.11020200	-0.46274100	0.39596900
H	1.23264700	0.99616100	-1.38565900
H	1.93666400	2.65685300	-1.00924700
H	1.02921200	0.99014800	1.51760800
H	2.82951900	1.32142900	1.30825500
H	-0.14552300	3.32793400	0.06328700
H	-2.04505300	1.94546300	0.27356200
H	3.09865900	-0.82400100	0.12302100
F	1.42295600	-2.51023700	-0.40728300
H	-0.83664300	-1.98734400	-0.39540500
C	-2.59142600	-0.40649000	0.12354800
N	-3.68450200	-0.77297500	0.23229000

3-F-5-NO_2-substituted

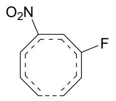

C	1.59635900	1.84751400	-0.88982800
C	2.29197700	0.73556700	1.00867500
C	0.40976200	2.30604300	-0.37381500
C	-0.72543600	1.52151000	-0.08014900
C	-0.87500400	0.14795900	-0.04865100
C	-0.05586500	-1.00615300	-0.14574400
C	1.28313300	-1.27032800	0.02810200
C	2.39210800	-0.50984600	0.44780400
H	1.63511800	0.90271700	-1.41560400
H	2.40200900	2.54623800	-1.09681400
H	1.37184600	1.04852500	1.48281700
H	3.19082500	1.27078400	1.29894300
H	0.33775200	3.35651200	-0.09918800
H	-1.62725600	2.07005100	0.16863900
H	3.36549800	-0.93527300	0.21578100
F	1.62192800	-2.54734500	-0.29620000
H	-0.61816000	-1.90555800	-0.37750200
N	-2.33703700	-0.24799300	0.08714800
O	-2.66969200	-1.35390700	-0.29446500
O	-3.10504800	0.56321500	0.56950800

3-CHO-5-CH_3-substituted

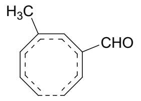

C	0.95387800	-1.96163100	-0.88792700
C	-0.29493900	-1.84096300	1.02592600
C	2.09161000	-1.39057800	-0.37165100
C	2.25141700	-0.02849500	-0.03877100
C	1.34795100	1.02666800	0.04793700
C	-0.07765100	1.09658900	-0.02798800
C	-1.18817800	0.26635000	0.10205700
C	-1.30651100	-1.09229800	0.48451000
H	0.22204700	-1.34574700	-1.39402900
H	0.93339400	-3.02234100	-1.12155400
H	0.54499300	-1.35459300	1.50371400
H	-0.47220700	-2.87798500	1.29793700
H	2.92782400	-2.04210400	-0.12244500
H	3.28003400	0.24974300	0.18532200
H	-2.26190800	-1.56005700	0.25483600
C	-2.47747200	0.93143300	-0.25259100
H	-2.40050100	2.01336000	-0.48834000
O	-3.55079400	0.37484900	-0.30383200
H	-0.38853900	2.13108700	-0.20124700
C	1.97276700	2.40903300	0.21319200
H	3.06070800	2.35637300	0.29250700
H	1.73290200	3.04136600	-0.64981600
H	1.59037300	2.91545300	1.10560700

3-CHO-5-NH_2-substituted

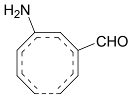

C	1.02747800	-1.91601400	-0.89928100
C	-0.22069500	-1.82333100	1.02808200
C	2.14320000	-1.29995700	-0.38476400
C	2.25618100	0.05350500	-0.01284600
C	1.31427300	1.08099400	0.04858700
C	-0.11359600	1.10614000	-0.07161600
C	-1.19148900	0.24531600	0.09816300
C	-1.26819900	-1.10719500	0.51026600
H	0.26523600	-1.33251000	-1.39885200
H	1.05272100	-2.97611100	-1.13358700
H	0.61483700	-1.31082700	1.48638300
H	-0.36279700	-2.86311300	1.31026100
H	3.00170600	-1.92284700	-0.13754700
H	3.26508700	0.36175800	0.25958500
H	-2.21437400	-1.60298100	0.30471600
C	-2.50171300	0.86173500	-0.27652700
H	-2.45278000	1.93005100	-0.57276700
O	-3.56183800	0.27952700	-0.27871200
H	-0.43479500	2.11928600	-0.32406500
N	1.83809900	2.39094700	0.15670700
H	2.84396900	2.43790800	0.25824100
H	1.37656200	2.97939000	0.84000600

3-CHO-5-OH-substituted

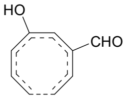

C	-1.09959800	-1.88448300	0.90217700
C	0.19903900	-1.82754500	-1.01260000
C	-2.18728900	-1.24455200	0.36371900
C	-2.25977800	0.11972100	0.01331300
C	-1.28144200	1.10289700	-0.06048000
C	0.14309600	1.12182500	0.02012200
C	1.20141800	0.23241700	-0.10274700
C	1.24772400	-1.13529600	-0.46997400
H	-0.33076700	-1.31916700	1.41244100
H	-1.14973500	-2.94493200	1.13027300
H	-0.61332300	-1.29724100	-1.49253300
H	0.31600000	-2.87502700	-1.27641600
H	-3.05659800	-1.84327800	0.09679500
H	-3.25875400	0.46920200	-0.24977000
H	2.17461100	-1.65237100	-0.23134400
C	2.52360300	0.83719000	0.24911000
H	2.50105000	1.92349000	0.47246200
O	3.56458600	0.22431600	0.30638900
H	0.46752500	2.14982500	0.18836200
O	-1.72676000	2.40064900	-0.23406300
H	-2.67325300	2.45673100	-0.04471100

3-CHO-5-F-substituted

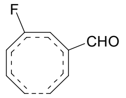

C	-1.18520300	-1.84768600	0.89427100
C	0.15844300	-1.81855300	-1.02010800
C	-2.25118700	-1.17956000	0.35100600
C	-2.27531900	0.19591100	0.03266700
C	-1.24391300	1.10625600	-0.05830200
C	0.17342000	1.12688000	-0.00305900
C	1.21172400	0.21246600	-0.10009300
C	1.21374800	-1.16227300	-0.45096200
H	-0.40404600	-1.30270400	1.40826300
H	-1.25641800	-2.90931500	1.11120600
H	-0.63437600	-1.26427800	-1.50612900
H	0.24377600	-2.86997900	-1.27945700
H	-3.13863400	-1.74736000	0.07848700
H	-3.24666300	0.63351900	-0.18572900
H	2.11843600	-1.70903500	-0.19374700
C	2.54776500	0.78600600	0.24586100
H	2.55613300	1.87712700	0.44573900
O	3.56859700	0.14257200	0.32174500
H	0.50921100	2.15528400	0.13928700
F	-1.71034100	2.38660800	-0.21550700

3-CHO-5-CHO-substituted

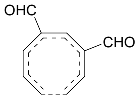

C	-0.12810900	2.27253900	-0.89505400
C	1.04776400	1.75842700	1.09786100
C	-1.37771200	2.02809200	-0.38590300
C	-1.89569300	0.74832000	-0.10327900
C	-1.28374800	-0.49881200	-0.03907700
C	0.06223800	-0.97696100	-0.08097100
C	1.34701400	-0.48391500	0.08855300
C	1.81125400	0.79591000	0.50461700
H	0.42496200	1.48201700	-1.38526100
H	0.19607200	3.28913000	-1.09894700
H	0.08756500	1.51635400	1.53405900
H	1.49505100	2.70117600	1.40060700
H	-2.01289100	2.87530000	-0.13491200
H	-2.96610500	0.70364400	0.09444000
H	2.85776300	1.00039600	0.28579200
C	2.42257800	-1.46342800	-0.24686900
H	2.06926900	-2.48457000	-0.49928400
O	3.60412900	-1.20545800	-0.26609300
H	0.07654900	-2.05368000	-0.27488600
C	-2.26932000	-1.63039800	0.07776500
H	-1.82866800	-2.64441400	-0.01286700
O	-3.45627500	-1.50504200	0.26426800

3-CHO-5-CN-substituted

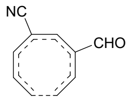

C	-0.14623900	2.29783200	-0.87771300
C	1.01081300	1.75222300	1.08587500
C	-1.40203900	2.10648600	-0.36043500
C	-1.97412900	0.85106100	-0.07240800
C	-1.40181600	-0.41874300	-0.00747300
C	-0.07397200	-0.96052900	-0.06072100
C	1.22514300	-0.50360900	0.09053000
C	1.73780600	0.75940900	0.49369000
H	0.36309600	1.48857200	-1.38435400
H	0.21733700	3.30078000	-1.08169100
H	0.05416900	1.54059900	1.54548400
H	1.49280000	2.68148500	1.37671500
H	-1.99744300	2.97786300	-0.09657700
H	-3.04214400	0.86590900	0.13411400
H	2.78751500	0.92778500	0.26053200
C	2.26384400	-1.52249600	-0.25804600
H	1.87460000	-2.53314100	-0.49569700
O	3.45061600	-1.29752600	-0.29872100
H	-0.11030200	-2.03465700	-0.24888300
C	-2.39664900	-1.47028900	0.11373100
N	-3.18587600	-2.31186500	0.21399300

3-CHO-5-NO_2-substituted

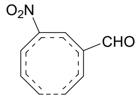

C	0.55220400	2.38506500	-0.93953200
C	1.56169300	1.71040700	1.06767200
C	-0.72786900	2.41408000	-0.44756800
C	-1.50576100	1.28021000	-0.13765600
C	-1.13596900	-0.04884800	-0.03806000
C	0.06249700	-0.81122200	-0.06142100
C	1.41535000	-0.57010300	0.11697500
C	2.12418900	0.59779300	0.51012800
H	0.93408800	1.49044300	-1.41389100
H	1.07630000	3.31050400	-1.15992800
H	0.57298500	1.66912500	1.50559300
H	2.18413800	2.55331000	1.35423400
H	-1.17866800	3.37765800	-0.22060500
H	-2.55630100	1.46455500	0.06192400
H	3.19177800	0.58259600	0.29877800
C	2.27629700	-1.75428600	-0.19275100
H	1.72864900	-2.69007200	-0.42343800
O	3.48455700	-1.72935600	-0.21023100
H	-0.15802600	-1.85999900	-0.24925100
N	-2.35098500	-0.95072500	0.08380800
O	-2.24898000	-2.10552600	-0.28666500
O	-3.36980600	-0.47282100	0.54604700

3-CN-5-CH_3-substituted

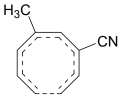

C	1.23670900	-1.80815200	-0.93086700
C	0.00256400	-1.93077100	1.02227800
C	2.24380200	-1.03623000	-0.41022400
C	2.13913200	0.32649900	-0.05098800
C	1.05148900	1.18529700	0.06070300
C	-0.36483200	0.99576600	-0.01817800
C	-1.28954000	-0.03913200	0.11294900
C	-1.14380400	-1.40038300	0.49653300
H	0.38569400	-1.34002600	-1.40792100
H	1.42021400	-2.84879500	-1.18269400
H	0.73812600	-1.28419600	1.48154500
H	0.03964100	-2.98172300	1.29315900
H	3.19520400	-1.51534300	-0.18409200
H	3.09637300	0.79527000	0.17058000
H	-1.98232700	-2.05655000	0.27454200
C	-2.65212500	0.34160200	-0.20051900
N	-3.74581500	0.62887900	-0.45206300
H	-0.86796100	1.94929900	-0.18177400
C	1.40055200	2.65802200	0.25361100
H	2.47709700	2.80871800	0.35714900
H	1.06387200	3.24478000	-0.60911400
H	0.91109100	3.07130200	1.14127500

3-CN-5-NH_2-substituted

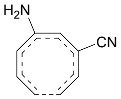

C	1.27945500	-1.76255100	-0.94659300
C	0.06137100	-1.89769400	1.03290800
C	2.26653200	-0.95962200	-0.43231300
C	2.13364200	0.38508100	-0.03060400
C	1.02229400	1.22237300	0.06037900
C	-0.39154500	1.00085700	-0.05958500
C	-1.28595800	-0.04871900	0.11928100
C	-1.11095700	-1.39716300	0.53538100
H	0.40471200	-1.32505200	-1.40988500
H	1.49711800	-2.79537500	-1.20177200
H	0.79765300	-1.23037800	1.46053700
H	0.12461600	-2.94324200	1.31958200
H	3.23172800	-1.41519600	-0.21535100
H	3.07217100	0.86864700	0.23778700
H	-1.94275200	-2.07099700	0.34551300
C	-2.65672700	0.29248700	-0.20655100
N	-3.75689700	0.55016800	-0.46099900
H	-0.89481300	1.93567000	-0.30212400
N	1.29973400	2.59846300	0.19786300
H	2.27808900	2.83138900	0.30728900
H	0.72296900	3.09382000	0.86657400

3-CN-5-OH-substituted

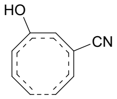

C	1.33640300	-1.72595900	-0.94893400
C	0.07013300	-1.89937200	1.01178900
C	2.29663000	-0.91322900	-0.40615000
C	2.12941100	0.43612900	-0.02630700
C	0.99573800	1.23056500	0.07494700
C	-0.41464800	1.01328500	-0.00955100
C	-1.29287800	-0.05535100	0.11496100
C	-1.09429400	-1.41477100	0.48504100
H	0.46440300	-1.29886500	-1.42713000
H	1.57085900	-2.75623700	-1.19902900
H	0.78193200	-1.22458100	1.46905400
H	0.15175500	-2.94929300	1.27631500
H	3.26413800	-1.35046200	-0.16457800
H	3.05346200	0.95181400	0.23629600
H	-1.90585200	-2.10118200	0.25629700
C	-2.66803100	0.27590800	-0.20073900
N	-3.77154600	0.51989600	-0.45339000
H	-0.91463000	1.96608800	-0.16910000
O	1.20698700	2.57920500	0.27921100
H	2.12807100	2.80657500	0.09157300

3-CN-5-F-substituted

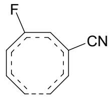

C	1.42160900	-1.67826300	-0.93953000
C	0.11116700	-1.88315000	1.01491500
C	2.35239800	-0.83940200	-0.38928700
C	2.13204000	0.51286500	-0.04255300
C	0.95861400	1.22544300	0.07206000
C	-0.44453800	1.01060600	0.01058500
C	-1.29881400	-0.07937300	0.10599800
C	-1.05353300	-1.43740900	0.45925100
H	0.54284600	-1.27625300	-1.42755400
H	1.67961300	-2.70614600	-1.17625100
H	0.79608600	-1.18695700	1.48152400
H	0.22584900	-2.93095300	1.27477400
H	3.33157400	-1.24112800	-0.13579300
H	3.01122400	1.11120300	0.18354000
H	-1.83615600	-2.15024600	0.21063300
C	-2.68103900	0.21903700	-0.20480900
N	-3.79034500	0.43872700	-0.45399200
H	-0.95527900	1.96200200	-0.12289500
F	1.19435900	2.56058400	0.26335500

3-CN-5-CHO-substituted

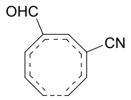

C	-0.20538300	2.29445300	-0.92970700
C	0.97280000	1.86824800	1.10099300
C	-1.43415400	1.95351300	-0.42950900
C	-1.84786600	0.63874700	-0.12840900
C	-1.13966400	-0.55293700	-0.03558100
C	0.23635900	-0.93866900	-0.07110100
C	1.47578900	-0.33714000	0.10560200
C	1.83341600	0.97877500	0.52956200
H	0.42476700	1.54578600	-1.39204100
H	0.03381400	3.33158100	-1.14699100
H	0.02821900	1.53576600	1.50989000
H	1.32513400	2.84710700	1.41138700
H	-2.14368500	2.74746300	-0.20457300
H	-2.91299100	0.51011300	0.06009800
H	2.85819600	1.28425300	0.32930100
C	2.60601500	-1.19367000	-0.18644800
N	3.52037300	-1.86411800	-0.42289000
H	0.33728300	-2.00921900	-0.25417500
C	-2.03714500	-1.75770500	0.09975700
H	-1.52184300	-2.73637700	0.02296100
O	-3.22906300	-1.71616700	0.28667800

3-CN-5-CN-substituted

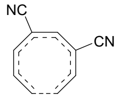

C	-0.28540600	2.30694000	-0.91285900
C	0.88940600	1.88059200	1.08832400
C	-1.51568100	1.98774800	-0.40136300
C	-1.95150000	0.68149200	-0.09508200
C	-1.24978800	-0.51903300	-0.00454700
C	0.12404400	-0.93382600	-0.05384600
C	1.36608700	-0.33432600	0.10449500
C	1.73900300	0.97977400	0.51527500
H	0.31919400	1.54952000	-1.39477200
H	-0.03164400	3.34034400	-1.13031500
H	-0.04567000	1.55423000	1.52392900
H	1.25249600	2.85932600	1.38661800
H	-2.20690000	2.79265300	-0.16055400
H	-3.01652100	0.58540600	0.10407300
H	2.76269200	1.27686100	0.29872900
C	2.48834000	-1.19706600	-0.20355400
N	3.40096700	-1.86506400	-0.45134800
H	0.20246700	-2.00410500	-0.23362700
C	-2.13363500	-1.66396400	0.13443800
N	-2.83830100	-2.57553700	0.24823900

3-CN-5-NO_2-substituted

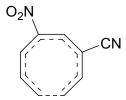

C	0.62152500	2.40516500	-0.96832500
C	1.60290900	1.74695700	1.07284500
C	-0.66117100	2.41755600	-0.48660300
C	-1.42127700	1.27360500	-0.16401100
C	-1.03208000	-0.04670800	-0.04101200
C	0.16902600	-0.80587100	-0.05688900
C	1.51772100	-0.53802800	0.13320300
C	2.20428200	0.64515000	0.53693100
H	1.02741500	1.50919700	-1.42008200
H	1.13091800	3.33673300	-1.19724500
H	0.60667400	1.68295800	1.48974500
H	2.20011700	2.60545300	1.36440200
H	-1.13270600	3.37574800	-0.28006700
H	-2.47585300	1.44297600	0.02757800
H	3.27469800	0.66572700	0.34482500
C	2.39086700	-1.66256400	-0.13317300
N	3.11191300	-2.54295100	-0.34625400
H	-0.03626200	-1.85651500	-0.23882600
N	-2.23834400	-0.96212300	0.09429900
O	-3.25240400	-0.49306200	0.57508000
O	-2.13019600	-2.11173000	-0.28563500

3-NO_2-5-CH_3-substituted

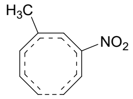

C	1.59738400	-1.80693500	-0.92068100
C	0.40415400	-1.94654600	1.05589400
C	2.59019000	-1.00170400	-0.42378000
C	2.44905800	0.36001300	-0.07406200
C	1.33634200	1.18228400	0.05722700
C	-0.07419400	0.94414100	0.01213300
C	-0.94320400	-0.11881300	0.16504000
C	-0.76924500	-1.47003700	0.53846200
H	0.72637600	-1.36846400	-1.38991500
H	1.80501000	-2.84503900	-1.16398800
H	1.12164300	-1.26974900	1.50006400
H	0.48651500	-2.99396300	1.32994900
H	3.55940400	-1.44998900	-0.21090800
H	3.39465800	0.86222200	0.12279900
H	-1.58370500	-2.15098600	0.31953000
N	-2.37364300	0.23188300	-0.14082300
O	-3.14952100	-0.69142600	-0.31761300
O	-2.69436900	1.40520100	-0.20047600
H	-0.62520700	1.87079100	-0.13257900
C	1.63689300	2.66649900	0.24102300
H	2.71055100	2.86210000	0.27635100
H	1.21848300	3.24455300	-0.59095200
H	1.18861700	3.05174400	1.16259300

3-NO_2-5-NH_2-substituted

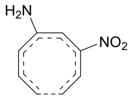

C	-1.63798200	-1.73876300	1.00970200
C	-0.42883800	-1.94475300	-1.02666700
C	-2.60442100	-0.93097100	0.47097400
C	-2.44509900	0.39797800	0.02565500
C	-1.31972700	1.20781600	-0.11077300
C	0.08978300	0.95632400	-0.00385400
C	0.93976500	-0.11688300	-0.16198800
C	0.75244800	-1.46884600	-0.53459500
H	-0.73899200	-1.31573700	1.43857600
H	-1.87449900	-2.76074100	1.29015500
H	-1.16417500	-1.27309700	-1.44894400
H	-0.52124300	-2.99624200	-1.28102000
H	-3.58253600	-1.36924300	0.27785700
H	-3.36957700	0.89885700	-0.25832300
H	1.56682600	-2.15201200	-0.32517000
N	2.37554000	0.21763000	0.15201400
H	0.64536300	1.86934200	0.19063900
N	-1.61090300	2.55208300	-0.41375600
H	-2.39479300	2.95657100	0.08162800
H	-0.82058100	3.18109900	-0.45269600
O	3.19159000	-0.68236400	0.06367400
O	2.66168200	1.35759000	0.47492100

3-NO_2-5-OH-substituted

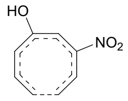

C	-1.67922600	-1.70301100	0.98982400
C	-0.44059200	-1.94616100	-0.98287100
C	-2.63287400	-0.88979800	0.43712800
C	-2.44590400	0.44664600	0.02251500
C	-1.29768600	1.21280800	-0.11675500
C	0.10755900	0.96581300	-0.03959400
C	0.94614700	-0.12028900	-0.15170500
C	0.74081200	-1.48050300	-0.47819100
H	-0.79679300	-1.27743200	1.44987700
H	-1.92396800	-2.72466900	1.26401500
H	-1.14195300	-1.27013000	-1.45439700
H	-0.55224300	-3.00206500	-1.20932500
H	-3.60992400	-1.31681200	0.21720400
H	-3.36344900	0.97448500	-0.23850300
H	1.53814000	-2.17200100	-0.23195600
N	2.38502700	0.21036000	0.14518100
O	3.19764300	-0.69053500	0.02981100
O	2.67169200	1.34364300	0.48327600
H	0.64242300	1.90245900	0.08851500
O	-1.47953800	2.55787800	-0.36305100
H	-2.39521800	2.81272400	-0.18410300

3-NO_2-5-F-substituted

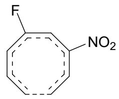

C	-1.75157200	-1.66441300	0.97399000
C	-0.46650900	-1.92449300	-0.99860100
C	-2.68022400	-0.82980400	0.41508200
C	-2.44946000	0.51319600	0.03967400
C	-1.26898600	1.20746400	-0.10566900
C	0.13029400	0.97209300	-0.05821300
C	0.95075700	-0.12956200	-0.14611600
C	0.70955200	-1.48872500	-0.46056000
H	-0.86404900	-1.26038700	1.44416400
H	-2.01352500	-2.68633300	1.23108200
H	-1.14748300	-1.23197100	-1.47650600
H	-0.60228200	-2.97761700	-1.22401900
H	-3.66569400	-1.22721100	0.18031500
H	-3.32454600	1.11834100	-0.18446600
H	1.48374100	-2.19946400	-0.19447300
N	2.39214700	0.17600900	0.14674000
O	3.17830000	-0.75341700	0.09569600
O	2.70573700	1.31968600	0.41960800
H	0.67172200	1.90852400	0.04642600
F	-1.48870300	2.53993100	-0.32551800

3-NO_2-5-CHO-substituted

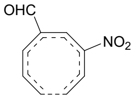

C	0.78001000	2.34059900	0.94768100
C	-0.42881600	2.08563500	-1.09848900
C	1.96237900	1.86583900	0.44593000
C	2.21839900	0.51418600	0.13198200
C	1.37138800	-0.58072500	0.01915000
C	-0.04140600	-0.79481600	0.03433700
C	-1.17947500	-0.04163100	-0.14467300
C	-1.39538800	1.30520600	-0.53812000
H	0.06611600	1.66408100	1.39949000
H	0.65823800	3.39619800	1.17332000
H	0.47011100	1.65104000	-1.51458400
H	-0.65666400	3.10817000	-1.38347600
H	2.76044300	2.57384600	0.23155500
H	3.26182300	0.25970200	-0.05037800
H	-2.37211300	1.72499700	-0.32444600
N	-2.45114900	-0.79265700	0.13066600
O	-3.48509000	-0.14824500	0.14748100
O	-2.39645000	-1.99330700	0.32526300
H	-0.28922500	-1.84295700	0.19335600
C	2.11368600	-1.88621100	-0.12324100
H	1.48016000	-2.79337800	-0.06095900
O	3.30335000	-1.98864600	-0.30098000

3-NO_2-5-CN-substituted

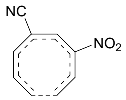

C	-0.95413300	-2.31056600	0.92940500
C	0.25218800	-2.10318000	-1.09434300
C	-2.12082100	-1.80176700	0.42372500
C	-2.34020200	-0.44459000	0.10607600
C	-1.45135400	0.62163500	-0.00794200
C	-0.02754900	0.80025600	0.01937000
C	1.07751100	-0.00160100	-0.14900000
C	1.24665400	-1.35481100	-0.53586100
H	-0.22934700	-1.65660900	1.39695800
H	-0.86612700	-3.36970500	1.15317300
H	-0.62141700	-1.63569400	-1.52874600
H	0.44438000	-3.13466100	-1.37336200
H	-2.93605300	-2.48558100	0.19738600
H	-3.37730800	-0.17646200	-0.08308400
H	2.20580200	-1.80914400	-0.31377800
N	2.37594300	0.70146300	0.14479900
O	3.38283000	0.01643400	0.16950200
O	2.36162900	1.90091400	0.34254100
H	0.24741100	1.83935200	0.17981300
C	-2.13303300	1.89627400	-0.15591600
N	-2.67859600	2.90993900	-0.27876900

3-NO_2-5-NO_2-substituted

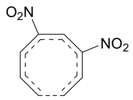

C	0.14243100	2.60309400	0.97903300
C	-0.96287400	2.16480900	-1.07035900
C	1.39790700	2.36554700	0.48711600
C	1.91854800	1.09487200	0.16135400
C	1.27614800	-0.12031100	0.02875900
C	-0.05198800	-0.62194600	0.03025400
C	-1.30279000	-0.08369400	-0.15843200
C	-1.76651700	1.20288700	-0.53257500
H	-0.43026400	1.80422200	1.43246900
H	-0.17516800	3.61619700	1.20801900
H	-0.00112600	1.91662800	-1.49957400
H	-1.38162400	3.12991100	-1.33896400
H	2.04589700	3.21334600	0.27638900
H	2.98701100	1.05224500	-0.02363700
H	-2.80577900	1.42598100	-0.31817500
N	-2.41490500	-1.06607700	0.09899000
O	-3.55167100	-0.62736100	0.09950000
O	-2.13259000	-2.23179000	0.29291500
H	-0.07872400	-1.69580200	0.19029400
N	2.27454300	-1.26006100	-0.10853500
O	1.91917200	-2.37323600	0.22326700
O	3.37972800	-0.99402500	-0.54204600

5.2.16 3,6-substituted

3-CH_3-6-CH_3-substituted

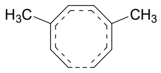

C	0.65065400	1.62853500	0.92727000
C	-0.65054200	1.62846800	-0.92730800
C	1.74104100	0.98485700	0.39703400
C	1.78036900	-0.38493200	0.03835100
C	0.71362900	-1.27559600	-0.04225700
C	-0.71371600	-1.27557500	0.04222500
C	-1.78042600	-0.38486800	-0.03833300
C	-1.74098900	0.98491300	-0.39703900
H	-0.11847200	1.05366000	1.42658100
H	0.71376600	2.68067900	1.19115000
H	0.11852500	1.05349700	-1.42660100
H	-0.71355300	2.68060800	-1.19122200
H	2.62707600	1.57469500	0.15939600
H	1.06230300	-2.29992900	-0.17914800
H	-2.62694300	1.57485600	-0.15939100
C	-3.16296000	-0.92559700	0.28090900
H	-3.13016100	-1.97264200	0.59153900
H	-3.62251800	-0.35149800	1.09439200
H	-3.82995500	-0.84843300	-0.58507500
H	-1.06241800	-2.29990500	0.17908900
C	3.16291300	-0.92566200	-0.28085500
H	3.83014100	-0.84766100	0.58487700
H	3.13022000	-1.97298300	-0.59058900
H	3.62214100	-0.35220200	-1.09497900

3-CH_3-6-NH_2-substituted

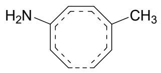

C	0.68428100	1.60387700	0.91372300
C	-0.63361400	1.65828300	-0.90464400
C	1.77413800	0.96879100	0.37197400
C	1.79472700	-0.40273000	0.02407100
C	0.71971600	-1.29433900	-0.03431200
C	-0.70191200	-1.27337900	0.02579600
C	-1.76428100	-0.37187600	-0.03832600
C	-1.71904900	1.00122000	-0.37516900
H	-0.07124100	1.02467200	1.42954000
H	0.74608900	2.65590200	1.17659500
H	0.12697200	1.09759600	-1.43193200
H	-0.70785500	2.71372800	-1.15256900
H	2.64707800	1.55327800	0.08439800
H	1.06866700	-2.32383500	-0.13244600
H	-2.60250900	1.58894500	-0.12117200
C	-3.14851400	-0.90981300	0.28281400
H	-3.60189700	-0.34612200	1.10725100
H	-3.82196200	-0.82048500	-0.57717700
H	-3.11888800	-1.96113600	0.57989400
H	-1.06190100	-2.29465400	0.15559800
N	3.06403900	-0.89545400	-0.35144000
H	3.82082500	-0.60476600	0.25630400
H	3.09540900	-1.89514700	-0.50976900

3-CH_3-6-OH-substituted

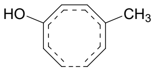

C	0.74705200	1.59745100	0.91990100
C	-0.60536400	1.65577300	-0.91995900
C	1.81177000	0.92627000	0.37621000
C	1.77408000	-0.44018000	0.01555800
C	0.70015100	-1.31473800	-0.07634300
C	-0.72072300	-1.27716500	0.01036000
C	-1.76086400	-0.35353200	-0.03221800
C	-1.69086400	1.02136900	-0.37173800
H	-0.03235200	1.04486300	1.42796800
H	0.84034000	2.64894600	1.17416900
H	0.15380800	1.07891600	-1.43212200
H	-0.66292900	2.71063200	-1.17396800
H	2.70620900	1.48371700	0.09624400
H	1.06465800	-2.33117300	-0.21871200
H	-2.56221900	1.62466400	-0.11279800
C	-3.15226300	-0.85798200	0.30902000
H	-3.55793600	-0.31684800	1.17263400
H	-3.84820500	-0.70556000	-0.52334300
H	-3.14984900	-1.92191600	0.55806800
H	-1.09511000	-2.29369600	0.13360400
O	2.97728300	-1.03227300	-0.31572600
H	3.70747200	-0.50795100	0.03931600

3-CH_3-6-F-substituted

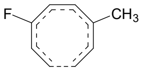

C	0.79304400	1.61858800	0.90761700
C	-0.55998200	1.64333100	-0.92832400
C	1.84422100	0.90975100	0.38234400
C	1.74425000	-0.44608600	0.02785600
C	0.68770100	-1.32657800	-0.08406400
C	-0.73275900	-1.28468200	0.01324800
C	-1.75308500	-0.34096200	-0.03013200
C	-1.65622400	1.03128000	-0.37502200
H	-0.00137100	1.09644700	1.42335800
H	0.91683400	2.66995200	1.14893900
H	0.18752600	1.05069000	-1.43968400
H	-0.60140100	2.69655700	-1.19135700
H	2.77664500	1.40456300	0.12171600
H	1.05770500	-2.33840400	-0.24445700
H	-2.51543700	1.65174200	-0.11720300
C	-3.15259500	-0.81613600	0.31791800
H	-3.53379700	-0.27768400	1.19399700
H	-3.85180900	-0.63224100	-0.50484900
H	-3.17409700	-1.88334600	0.55077800
H	-1.11807200	-2.29660000	0.13656200
F	2.95220500	-1.00807900	-0.27405000

3-CH_3-6-CHO-substituted

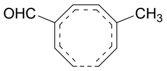

C	-0.07007600	1.94264300	-0.90311600
C	1.16301200	1.52629000	1.03967900
C	-1.24510000	1.44520300	-0.41141900
C	-1.50215300	0.07732400	-0.13029000
C	-0.64099800	-1.01407900	-0.06581700
C	0.75408100	-1.28484100	-0.11581900
C	1.95259400	-0.59082700	0.03056100
C	2.13320300	0.74499600	0.47114800
H	0.62996800	1.28626100	-1.40230200
H	0.04000900	3.00676500	-1.09148500
H	0.27712600	1.06918400	1.46179700
H	1.39459500	2.53485100	1.36973200
H	-2.03754700	2.14832900	-0.15127800
H	-1.21270300	-1.93936100	0.01805200
H	3.11339900	1.18703300	0.29099300
C	3.23190300	-1.33596700	-0.29713800
H	3.77382700	-0.82475700	-1.10164100
H	3.89999800	-1.37326700	0.57009700
H	3.03607300	-2.35954300	-0.62392900
H	0.91906000	-2.34841900	-0.28776200
C	-2.93930100	-0.23412300	0.12212500
H	-3.60475400	0.65431900	0.13995500
O	-3.40650400	-1.33763900	0.29603500

3-CH_3-6-CN-substituted

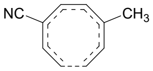

C	0.25098800	1.79808800	0.93293300
C	-0.99058800	1.55969800	-0.99946800
C	1.39632900	1.23895000	0.43593200
C	1.53711200	-0.14262500	0.13180100
C	0.58811000	-1.16145400	0.04344500
C	-0.82825800	-1.30238800	0.08396400
C	-1.96307200	-0.50756100	-0.05055800
C	-2.02800200	0.84984100	-0.45384200
H	-0.48426500	1.17722700	1.42706000
H	0.21343000	2.86201800	1.14673900
H	-0.15480800	1.03735800	-1.44743200
H	-1.13361000	2.59281800	-1.30285100
H	2.23893800	1.87934700	0.18442000
H	1.06085600	-2.13769400	-0.05443300
H	-2.96585900	1.37007200	-0.25843200
C	-3.30167100	-1.15295900	0.25159900
H	-3.81635700	-0.60666400	1.05069800
H	-3.95562900	-1.13366400	-0.62687500
H	-3.19218200	-2.19061000	0.57408500
H	-1.08286800	-2.35105400	0.23473800
C	2.89668200	-0.56382200	-0.13034300
N	3.98950900	-0.88482500	-0.34292700

3-CH_3-6-NO_2-substituted

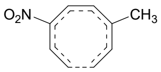

C	0.16593700	1.87502600	-0.90674500
C	1.38430800	1.55894200	1.02542000
C	-1.00308100	1.35486000	-0.42351000
C	-1.17089400	-0.02362700	-0.15987100
C	-0.27527900	-1.07590200	-0.08520900
C	1.13425800	-1.26739800	-0.11973000
C	2.29546200	-0.51559200	0.03814300
C	2.40290200	0.82979400	0.46949000
H	0.87756900	1.23993400	-1.41730000
H	0.24832900	2.94245700	-1.08750400
H	0.53349300	1.05249500	1.46319000
H	1.55482000	2.58217800	1.34744400
H	-1.82005300	2.01357800	-0.15105300
H	-0.79530800	-2.02722800	-0.01548400
H	3.35701200	1.32475200	0.28842700
C	3.61210200	-1.19961300	-0.27284300
H	3.46941800	-2.23078500	-0.60280800
H	4.14028200	-0.66262800	-1.06945000
H	4.26856600	-1.20634900	0.60376300
H	1.35232800	-2.32038300	-0.29275000
N	-2.59392400	-0.41919600	0.08751500
O	-2.85568300	-1.59092400	0.29770700
O	-3.43222700	0.46685100	0.06854800

3- NH_2-6-NH_2-substituted

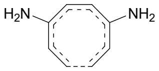

C	0.67016600	1.63416200	0.89247200
C	-0.67016900	1.63406900	-0.89252800
C	1.75217900	0.98640600	0.34553000
C	1.77812000	-0.38943500	0.02365400
C	0.70980100	-1.28959600	-0.01977900
C	-0.70980300	-1.28962300	0.01981800
C	-1.77814300	-0.38948800	-0.02367100
C	-1.75218100	0.98636000	-0.34553700
H	-0.07441800	1.06881800	1.43843500
H	0.74102400	2.69034500	1.13626600
H	0.07438000	1.06868000	-1.43849200
H	-0.74098400	2.69025300	-1.13633000
H	2.62133300	1.56618600	0.03673400
H	1.06814300	-2.31592200	-0.11708400
H	-2.62128900	1.56618700	-0.03667800
N	-3.05593200	-0.87916600	0.35371300
H	-3.07638900	-1.87587700	0.53375300
H	-3.79504500	-0.62760900	-0.29310900
H	-1.06811000	-2.31596600	0.11714800
N	3.05598800	-0.87915200	-0.35366700
H	3.79488600	-0.62825200	0.29366000
H	3.07624900	-1.87574900	-0.53437600

3- NH_2-6-OH-substituted

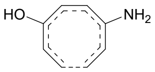

C	0.73731100	1.62919900	0.89631400
C	-0.63937200	1.63171300	-0.90674700
C	1.79374200	0.94228200	0.35074300
C	1.75748600	-0.42831700	0.01626300
C	0.68716900	-1.31101700	-0.05696800
C	-0.73064200	-1.29273400	0.00713900
C	-1.77628800	-0.36838400	-0.01499100
C	-1.72286900	1.00881900	-0.34233600
H	-0.03262000	1.09192400	1.43410200
H	0.84261000	2.68403000	1.13186600
H	0.10345200	1.04821800	-1.43617500
H	-0.69185700	2.68706700	-1.15789800
H	2.68643900	1.49326500	0.05177500
H	1.06070900	-2.32454800	-0.19674900
H	-2.57941600	1.60655100	-0.03239600
N	-3.05763800	-0.82628800	0.37695100
H	-3.10785200	-1.82418100	0.54322100
H	-3.80654400	-0.53408200	-0.24054400
H	-1.10560800	-2.31345300	0.09635100
O	2.96287300	-1.02281700	-0.32356400
H	3.69195400	-0.51760500	0.05980600

3- NH_2-6-F-substituted

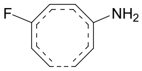

C	0.78420100	1.65131600	0.88088000
C	-0.59096100	1.61972000	-0.91326900
C	1.82754800	0.92405600	0.35872100
C	1.72739900	-0.43540200	0.02893100
C	0.67220300	-1.32360000	-0.06561400
C	-0.74345300	-1.30074500	0.00803800
C	-1.77016700	-0.35421800	-0.01213100
C	-1.68741500	1.01997500	-0.34686000
H	-0.00052600	1.14667200	1.42769200
H	0.92304500	2.70506400	1.10338400
H	0.13953800	1.01998900	-1.44172800
H	-0.62813000	2.67270600	-1.17615700
H	2.76002000	1.41135000	0.08271700
H	1.05104100	-2.33280800	-0.22193100
H	-2.53136600	1.63699800	-0.04071700
N	-3.05336500	-0.78527600	0.38512000
H	-3.13610800	-1.78278600	0.53779400
H	-3.81032000	-0.44498300	-0.19586100
H	-1.13046800	-2.31663300	0.09424800
F	2.93563300	-1.00280500	-0.27749600

3- NH_2-6-CHO-substituted

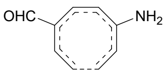

C	-0.03692200	1.97092200	-0.87104900
C	1.18863100	1.49285400	1.00923000
C	-1.21507900	1.46193500	-0.38867300
C	-1.48462300	0.09560700	-0.12928700
C	-0.63045200	-1.01365000	-0.08944700
C	0.74444900	-1.31332400	-0.10366000
C	1.96293000	-0.62128900	0.01870400
C	2.16546100	0.71342400	0.44645100
H	0.65132900	1.33003600	-1.40521000
H	0.06344100	3.03861400	-1.04505700
H	0.31581700	1.03055700	1.45305800
H	1.42216700	2.50091800	1.33793500
H	-2.00396900	2.16446600	-0.11422700
H	-1.22085800	-1.92881300	-0.02713100
H	3.13564600	1.15405300	0.22160200
N	3.11656900	-1.30953600	-0.35126000
H	3.00959300	-2.29549300	-0.54895500
H	3.95416800	-1.10367500	0.17699100
H	0.90452600	-2.38552100	-0.22354600
C	-2.91515400	-0.20873300	0.12800500
H	-3.57419500	0.68454200	0.15928900
O	-3.39363800	-1.31117600	0.29405400

3-NH_2-6-CN-substituted

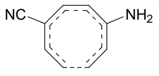

C	-0.22121200	1.82794700	-0.89978200
C	1.01336400	1.52931100	0.97352100
C	-1.36993900	1.25503000	-0.41577800
C	-1.52090600	-0.12676000	-0.13514100
C	-0.57551200	-1.16060700	-0.06451800
C	0.82520300	-1.32762800	-0.06888900
C	1.97744800	-0.53131300	0.04080700
C	2.06025300	0.82559900	0.43391300
H	0.50244700	1.22238300	-1.42844700
H	-0.19416400	2.89530800	-1.09827700
H	0.19239900	1.00059000	1.44164700
H	1.15561200	2.56158900	1.27861200
H	-2.21043400	1.89408100	-0.15204100
H	-1.06381000	-2.13085400	0.01552900
H	2.98686400	1.34536500	0.19554400
N	3.19021200	-1.12459400	-0.31341300
H	3.16686500	-2.11961000	-0.49332100
H	3.99829200	-0.84773400	0.22867500
H	1.07769400	-2.38353800	-0.16870400
C	-2.87879300	-0.54358900	0.12966900
N	-3.97181200	-0.86476600	0.34455200

3-NH_2-6-NO_2-substituted

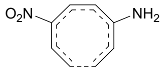

C	0.19236500	1.90305500	-0.86301400
C	1.39833800	1.52375900	1.00027600
C	-0.98060300	1.36612700	-0.39630900

C	-1.15833400	-0.01287600	-0.16105800
C	-0.26195900	-1.07870500	-0.11165900
C	1.12867700	-1.29370500	-0.10885500
C	2.30875900	-0.53797600	0.02524400
C	2.43126300	0.80466300	0.45180800
H	0.89124600	1.28792400	-1.41381000
H	0.26258600	2.97462000	-1.02426500
H	0.56559100	1.00536600	1.45896700
H	1.56539800	2.54496100	1.32907600
H	-1.79623600	2.02075800	-0.10827900
H	-0.79518400	-2.02408700	-0.06671700
H	3.37291800	1.30436600	0.23082000
N	3.49730500	-1.16385200	-0.33672900
H	3.44835900	-2.15218600	-0.54427100
H	4.32393700	-0.90940000	0.18724800
H	1.34669300	-2.35428000	-0.23343100
N	-2.57189400	-0.40409100	0.08608400
O	-2.83415700	-1.57233500	0.32721600
O	-3.41762100	0.47627300	0.03785600

3-OH-6-OH-substituted

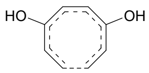

C	-0.67825500	1.63501400	-0.90231400
C	0.70001600	1.62591000	0.90698300
C	-1.75888800	0.98886600	-0.35890700
C	-1.76722200	-0.38143200	-0.02059400
C	-0.72469800	-1.30094100	0.01751700
C	0.69239500	-1.31124200	-0.06288500
C	1.74896000	-0.41374100	0.01280000
C	1.76499100	0.95554900	0.35874300
H	0.08297700	1.06887100	-1.42324400
H	-0.75097100	2.68902100	-1.15345400
H	-0.06792200	1.07690900	1.43551100
H	0.79361300	2.67947700	1.15248800
H	-2.65191100	1.54540300	-0.08382600
H	-1.09955100	-2.32201600	0.11693000
H	2.65137000	1.52006200	0.06673700
O	2.96083200	-0.98850900	-0.33027800
H	3.68465700	-0.46859500	0.04347200
H	1.07471500	-2.32051100	-0.20752100
O	-3.03496400	-0.82976200	0.28780600
H	-2.98771000	-1.71036200	0.68461800

3-OH-6-F-substituted

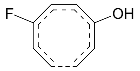

C	0.75342400	1.65049700	0.89611000
C	-0.65772000	1.61675500	-0.91751100
C	1.79877700	0.94895000	0.35228700
C	1.72558800	-0.41473800	0.01770300
C	0.69326200	-1.32733200	-0.05342700
C	-0.72164900	-1.32207000	0.05024100
C	-1.75038500	-0.39288100	-0.00187700

C	-1.72903800	0.97903900	-0.34855300
H	-0.03038500	1.12681700	1.42649800
H	0.87362800	2.70455200	1.12712800
H	0.09787200	1.04394100	-1.43924600
H	-0.72724300	2.67002900	-1.17132600
H	2.71899000	1.45563900	0.07046000
H	1.08670900	-2.33144700	-0.20441900
H	-2.59641100	1.56852800	-0.04942800
O	-2.97185900	-0.92917100	0.35055500
H	-3.68606400	-0.37333900	0.01083900
H	-1.12252900	-2.32533200	0.18189900
F	2.94297300	-0.94837200	-0.30296500

3-OH-6-CHO-substituted

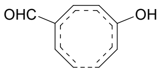

C	-0.01154400	1.96070500	-0.88979200
C	1.25009500	1.46912900	1.02808600
C	-1.18385800	1.47175700	-0.38409700
C	-1.47530400	0.10605400	-0.12350500
C	-0.64696900	-1.01623000	-0.10597000
C	0.72950100	-1.32881700	-0.15452100
C	1.93755200	-0.65458500	0.00513900
C	2.19344200	0.66444800	0.45096800
H	0.67615800	1.30422800	-1.40595100
H	0.10507100	3.02579200	-1.06850400
H	0.35659900	1.03608300	1.45884000
H	1.51238100	2.47142800	1.35173000
H	-1.95877500	2.18643500	-0.10189100
H	-1.24754700	-1.92471700	-0.04616000
H	3.17946400	1.07493800	0.23187900
O	3.02144800	-1.42383700	-0.32461900
H	3.83469200	-1.01403400	0.00125900
H	0.90806700	-2.38849200	-0.32826200
C	-2.91317700	-0.17136100	0.14529400
H	-3.55495200	0.73377300	0.18596400
O	-3.40764700	-1.26516800	0.31105500

3-OH-6-CN-substituted

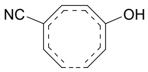

C	-0.20256700	1.81698000	-0.92173300
C	1.06975300	1.51768100	0.98823600
C	-1.34471500	1.26150400	-0.41378600
C	-1.51354700	-0.12213900	-0.13097000
C	-0.58877500	-1.16873700	-0.08271200
C	0.81456200	-1.34364100	-0.11816300
C	1.95579000	-0.56090800	0.03043700
C	2.08867200	0.78688400	0.43823400
H	0.52331900	1.19830600	-1.43241400
H	-0.16300900	2.88281700	-1.12548600
H	0.22781400	1.01395800	1.44534000
H	1.23761800	2.54841000	1.28455900
H	-2.17451800	1.91118600	-0.14278400

H	-1.08459400	-2.13529600	-0.00715100
H	3.03256300	1.28058300	0.20662400
O	3.10581400	-1.23915200	-0.27869000
H	3.87690900	-0.76170500	0.05728200
H	1.08944600	-2.38585100	-0.26936100
C	-2.87743600	-0.51449600	0.14545000
N	-3.97464000	-0.81113700	0.37185100

3-OH-6-NO_2-substituted

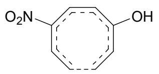

C	0.20847500	1.88935000	-0.89041500
C	1.45439600	1.50979400	1.01545400
C	-0.95621700	1.36857600	-0.39802600
C	-1.14996100	-0.01196500	-0.15953000
C	-0.27547100	-1.08850100	-0.13014300
C	1.11929700	-1.31011200	-0.15920700
C	2.28562900	-0.56834800	0.01486300
C	2.45688000	0.76466600	0.45389300
H	0.91090300	1.26039000	-1.42122100
H	0.29134600	2.95910200	-1.05679100
H	0.60003500	1.01833900	1.46268100
H	1.64647900	2.52996300	1.33281100
H	-1.76124300	2.03288600	-0.10292700
H	-0.81594900	-2.02988400	-0.08737500
H	3.41465800	1.23743000	0.23677300
O	3.41238500	-1.27608700	-0.30188800
H	4.19910100	-0.82455400	0.03415300
H	1.36035800	-2.35646100	-0.33491500
N	-2.57213600	-0.38227000	0.09813500
O	-2.84991700	-1.54762800	0.32756100
O	-3.39983100	0.51470500	0.07039400

3-F-6-F-substituted

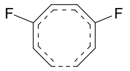

C	0.70132700	1.63847500	0.90700500
C	-0.70129900	1.63847300	-0.90697700
C	1.76077200	0.96246500	0.35733000
C	1.71842600	-0.40092500	0.01385500
C	0.70712600	-1.33580700	-0.05892200
C	-0.70713800	-1.33580600	0.05889900
C	-1.71843400	-0.40091500	-0.01385700
C	-1.76076600	0.96247100	-0.35733000
H	-0.07153200	1.09606500	1.43474900
H	0.80233200	2.69193600	1.14918100
H	0.07156000	1.09607100	-1.43472600
H	-0.80229400	2.69194400	-1.14911800
H	2.66886900	1.49095900	0.07710400
H	1.11245600	-2.33444900	-0.21137800
H	-2.66898000	1.49090700	-0.07741500
F	-2.94433500	-0.90330200	0.31228100
H	-1.11246800	-2.33443400	0.21140000
F	2.94433200	-0.90331800	-0.31226000

3-F-6-CHO-substituted

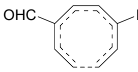

C	0.03954300	1.93192300	-0.90386000
C	1.30281600	1.47346400	1.02764800
C	-1.14244100	1.47292200	-0.39301200
C	-1.46326700	0.11477100	-0.12322300
C	-0.66277800	-1.02241100	-0.09696600
C	0.71598900	-1.34101500	-0.16185300
C	1.90524800	-0.66124600	0.01645400
C	2.22413500	0.63534300	0.46077400
H	0.71433300	1.25710400	-1.41342400
H	0.17902900	2.99246800	-1.09197700
H	0.39594000	1.07073800	1.45912200
H	1.59383600	2.46999600	1.34436600
H	-1.90119600	2.20606500	-0.11531500
H	-1.27347800	-1.92302800	-0.02656200
H	3.23623800	0.97448000	0.25503000
F	3.00369900	-1.39893000	-0.29292600
H	0.89858200	-2.39656200	-0.35593600
C	-2.91108800	-0.12926000	0.14825100
H	-3.53355700	0.78888100	0.18389600
O	-3.42399500	-1.21208900	0.31898300

3-F-6-CN-substituted

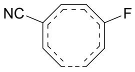

C	-0.16015200	1.79231500	-0.93724700
C	1.11612200	1.53467600	0.98357400
C	-1.31029400	1.26101700	-0.42194500
C	-1.50373400	-0.11835300	-0.12933700
C	-0.60078300	-1.17916600	-0.07525900
C	0.80540800	-1.35481900	-0.12515000
C	1.92487300	-0.56364800	0.04266700
C	2.11758200	0.76934900	0.44646200
H	0.55643700	1.15780900	-1.44175900
H	-0.10359400	2.85518200	-1.15164000
H	0.25928300	1.06085700	1.44409200
H	1.31157900	2.56403200	1.26750700
H	-2.12798100	1.92692000	-0.15439600
H	-1.10402100	-2.14077800	0.00820900
H	3.09310900	1.19763100	0.23049900
F	3.08757200	-1.20642400	-0.24110600
H	1.08828000	-2.39180200	-0.29624900
C	-2.87566400	-0.48398300	0.15072600
N	-3.97734100	-0.75948000	0.37953400

3-F-6-NO_2-substituted

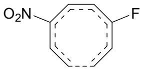

C	0.24756100	1.85906000	-0.91354800
C	1.50042900	1.52567000	1.00968300
C	-0.92321600	1.36195500	-0.41184300
C	-1.13885800	-0.01361500	-0.15987300
C	-0.28721500	-1.10259600	-0.11957500
C	1.11166800	-1.32389300	-0.16216000
C	2.25466600	-0.57212800	0.02918500
C	2.48402400	0.74608300	0.46043900
H	0.94285600	1.21401700	-1.43446600
H	0.34557800	2.92510400	-1.09412600
H	0.63105900	1.06497900	1.45997500
H	1.71898800	2.54508800	1.31190400
H	-1.71743600	2.04147800	-0.12270200
H	-0.83486800	-2.03894300	-0.06441700
H	3.47104200	1.15267000	0.25545700
F	3.39759800	-1.24364300	-0.26043400
H	1.36154700	-2.36538000	-0.35392400
N	-2.57234400	-0.35874300	0.10288900
O	-3.37772700	0.55671700	0.09727500
O	-2.87041100	-1.52149700	0.31174200

3-CHO-6-CHO-substituted

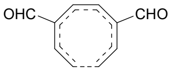

C	-0.43795200	1.92267600	-0.94686000
C	0.79350100	1.67986800	1.06787300
C	-1.59525300	1.39594200	-0.44559700
C	-1.80630800	0.02569900	-0.13947200
C	-0.90895500	-1.02927800	-0.03706600
C	0.50045300	-1.20794600	-0.05308600
C	1.63997800	-0.42844600	0.10974600
C	1.78652400	0.92418700	0.51231000
H	0.29777000	1.28094600	-1.41358800
H	-0.36172900	2.98519700	-1.15809000
H	-0.08571800	1.20844200	1.48824500
H	0.98530000	2.70726200	1.36401200
H	-2.41505900	2.07540900	-0.21036800
H	-1.43408600	-1.98067400	0.05179700
H	2.76072300	1.36843100	0.31773000
C	2.91652800	-1.13987500	-0.20508400
H	2.81144900	-2.21885100	-0.43934600
O	4.00446200	-0.61261400	-0.22722900
H	0.74694200	-2.26000100	-0.21091800
C	-3.24108900	-0.33542300	0.09387800
H	-3.94062100	0.52556900	0.08670100
O	-3.66065200	-1.45440600	0.27522600

3-CHO-6-CN-substituted

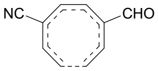

C	0.58064900	1.76407400	0.97495400
C	-0.64651900	1.67425100	-1.02337100
C	1.71723400	1.20118900	0.46463800
C	1.84188800	-0.17667300	0.13625900
C	0.88010200	-1.17771600	0.01585600
C	-0.53924400	-1.25525600	0.02306700
C	-1.62653300	-0.40209400	-0.12445600
C	-1.68671800	0.96758700	-0.48745900
H	-0.17585200	1.14371200	1.43759100
H	0.55440900	2.82321100	1.21183700
H	0.19311200	1.15987200	-1.47329600
H	-0.77265200	2.72024900	-1.28793700
H	2.57100600	1.83440300	0.23473800
H	1.32638300	-2.16553600	-0.08321200
H	-2.62915500	1.46831600	-0.27427200
C	-2.94649000	-1.04033700	0.17027700
H	-2.91095000	-2.12960200	0.37623100
O	-3.99800300	-0.44520500	0.20359900
H	-0.85324000	-2.29214400	0.15781400
C	3.20001800	-0.61452800	-0.11015300
N	4.29066400	-0.94911700	-0.30942300

3-CHO-6-NO_2-substituted

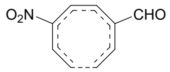

C	-0.16110900	1.82928100	-1.00721100
C	1.05053800	1.71754700	1.00395300
C	-1.31809500	1.31539400	-0.49154800
C	-1.46447300	-0.05221400	-0.16759200
C	-0.55818700	-1.08495600	-0.03282200
C	0.85833500	-1.21170900	-0.02474100
C	1.97049700	-0.39375400	0.12649600
C	2.07161000	0.97624100	0.47858800
H	0.57545400	1.18101000	-1.46388800
H	-0.09528200	2.88629200	-1.24551300
H	0.19365000	1.23274900	1.45419500
H	1.20517200	2.76223800	1.25819100
H	-2.14894000	1.97085700	-0.25543400
H	-1.05245200	-2.04769700	0.05968000
H	3.02995900	1.44646300	0.26727300
C	3.27253800	-1.07748000	-0.15204700
H	3.20366300	-2.16637900	-0.35007300
O	4.34177700	-0.51548900	-0.18078600
H	1.13738400	-2.25921700	-0.15106800
N	-2.88852300	-0.46233300	0.08346700
O	-3.15943200	-1.64822200	0.11221900
O	-3.70220300	0.42870000	0.24905400

3-CN-6-CN-substituted

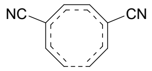

C	0.61280400	1.77351200	1.01388400
C	-0.61278500	1.77350400	-1.01387900

C	1.70584200	1.14453200	0.49084400
C	1.73706000	-0.23402700	0.13610800
C	0.71107900	-1.16440700	-0.00492700
C	-0.71108500	-1.16440500	0.00498100
C	-1.73706000	-0.23402500	-0.13610100
C	-1.70582600	1.14453200	-0.49084000
H	-0.19121100	1.19960700	1.45649900
H	0.65758100	2.82795800	1.26832400
H	0.19122400	1.19959500	-1.45650100
H	-0.65755500	2.82795200	-1.26831200
H	2.60244800	1.72098700	0.27508300
H	1.09203400	-2.17687300	-0.12452800
H	-2.60245600	1.72097800	-0.27515800
C	-3.06291400	-0.75858900	0.11490600
N	-4.12909900	-1.16252800	0.31732800
H	-1.09204500	-2.17686300	0.12460000
C	3.06290300	-0.75859900	-0.11492700
N	4.12908100	-1.16254400	-0.31737100

3-CN-6-NO_2-substituted

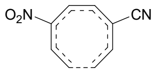

C	-0.18182300	1.86540100	-1.04023100
C	1.02895200	1.82203200	1.00002000
C	-1.30325300	1.29080300	-0.51502800
C	-1.36923600	-0.08000700	-0.17103300
C	-0.40607700	-1.05523300	-0.01753200
C	1.01535300	-1.11850600	-0.01319000
C	2.07668900	-0.23177300	0.13585700
C	2.09885700	1.14848800	0.48534300
H	0.59916600	1.25832500	-1.48004700
H	-0.17909300	2.92147700	-1.29140500
H	0.20048500	1.28343000	1.44184300
H	1.11301300	2.87570700	1.24766100
H	-2.17349600	1.89742800	-0.29011000
H	-0.84671000	-2.04187800	0.09335600
H	3.01906700	1.68798900	0.27369100
C	3.38259800	-0.81160300	-0.09962300
N	4.43353700	-1.25930300	-0.28849700
H	1.35239200	-2.14670800	-0.12801700
N	-2.76753900	-0.56848400	0.08627800
O	-2.97060400	-1.76718600	0.12491600
O	-3.62929100	0.27732700	0.24521700

3-NO_2-6-NO_2-substituted

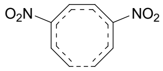

C	-0.59996800	1.92877000	-1.02693500
C	0.60004700	1.92866500	1.02724200
C	-1.69895000	1.30779400	-0.50798900
C	-1.71106800	-0.06606800	-0.16926600
C	-0.71178000	-1.00130800	-0.00978400
C	0.71179000	-1.00133000	0.01000100
C	1.71109800	-0.06608900	0.16934800

C	1.69902100	1.30773100	0.50822000
H	0.20723200	1.35694500	-1.46679000
H	-0.63702300	2.98592400	-1.27073800
H	-0.20716600	1.35679400	1.46701000
H	0.63712500	2.98578300	1.27119300
H	-2.59377100	1.87784400	-0.28397200
H	-1.11082400	-2.00595600	0.09572800
H	2.59387500	1.87777500	0.28431600
N	3.09171200	-0.61175800	-0.07450000
O	3.98438100	0.19898900	-0.24306100
O	3.24784200	-1.81733200	-0.09416700
H	1.11080500	-2.00598800	-0.09552400
N	-3.09174700	-0.61175700	0.07431000
O	-3.24782000	-1.81733700	0.09444400
O	-3.98454700	0.19899200	0.24217000

5.3.17 4,5-substituted

4-CH_3-5-CH_3-substituted

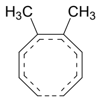

C	-2.09303800	-0.64873700	-0.92772900
C	-2.09326000	0.64862100	0.92757800
C	-1.39468300	-1.69423500	-0.36865800
C	-0.04995400	-1.68421800	0.05759400
C	0.94699200	-0.71036700	0.13063500
C	0.94695900	0.71045600	-0.13052300
C	-0.05003700	1.68425100	-0.05736100
C	-1.39484600	1.69415500	0.36865300
H	-1.56572200	0.13997700	-1.44537900
H	-3.13711200	-0.78531900	-1.19773900
H	-1.56604300	-0.14005700	1.44538200
H	-3.13742100	0.78513200	1.19728700
H	0.28354200	-2.66282500	0.39847800
H	0.28343000	2.66291800	-0.39809600
H	-1.95231800	2.59396200	0.11162000
H	-1.95216200	-2.59405900	-0.11169800
C	2.31570600	1.28228800	-0.49427700
H	2.80967800	0.69748500	-1.27693300
H	2.22132100	2.30807000	-0.85581100
H	2.99189200	1.30397500	0.36839700
C	2.31578500	-1.28220500	0.49420200
H	2.99161300	-1.30443000	-0.36874600
H	2.81019800	-0.69706300	1.27635100
H	2.22135700	-2.30782000	0.85620300

4-CH_3-5-NH_2-substituted

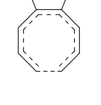

C	-2.10147700	-0.59976300	-0.94596600
C	-2.05348300	0.74054700	0.91587300

C	-1.44009600	-1.65281200	-0.35846600
C	-0.10963900	-1.68606300	0.10054700
C	0.92672600	-0.74807200	0.14797800
C	0.99283700	0.66978400	-0.13352400
C	0.03880600	1.68018200	-0.05633700
C	-1.30820600	1.75369800	0.36137100
H	-1.55035600	0.17533700	-1.45965700
H	-3.14560500	-0.71320700	-1.22457300
H	-1.56446500	-0.07569300	1.42922500
H	-3.09337500	0.92008900	1.17651000
H	0.18681500	-2.66425500	0.47951900
H	0.41811000	2.63963800	-0.40528400
H	-1.82277700	2.67603100	0.09631900
H	-2.03025700	-2.52945100	-0.09358900
C	2.38884100	1.16263100	-0.50111800
H	2.89303100	0.46919000	-1.17905100
H	2.33165600	2.14506400	-0.97467300
H	3.03349100	1.27727900	0.38200000
N	2.19709400	-1.31126300	0.41034600
H	2.16900500	-2.28527600	0.68327100
H	2.78922400	-0.77669800	1.03541200

4-CH_3-5-OH-substituted

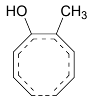

C	2.11555100	-0.59033600	0.92146600
C	2.01932100	0.81116100	-0.89509900
C	1.47588400	-1.63989100	0.30855400
C	0.13075100	-1.68812100	-0.10992300
C	-0.90110700	-0.75430200	-0.14013200
C	-1.02212900	0.65639800	0.12162000
C	-0.08905500	1.68687400	0.09444800
C	1.26423000	1.78984700	-0.29655900
H	1.54903500	0.15106100	1.46761500
H	3.16740500	-0.67892700	1.17851300
H	1.53646900	0.00968400	-1.43773300
H	3.05709500	1.00958200	-1.14932100
H	-0.16847000	-2.66506300	-0.49345800
H	-0.49721600	2.63134800	0.45053500
H	1.76606600	2.70781200	0.00559000
H	2.07929500	-2.49402900	0.00442800
C	-2.45183500	1.09137900	0.41568200
H	-2.92387500	0.44514800	1.16091800
H	-2.46834600	2.12132100	0.77840000
H	-3.07083400	1.03621100	-0.48575300
O	-2.14616000	-1.27629700	-0.44634400
H	-2.10700500	-2.24182000	-0.42932300

4-CH_3-5-F-substituted

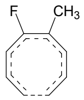

C	2.13359000	-0.56140100	0.89921000
C	1.96453600	0.89929600	-0.89288100
C	1.53231500	-1.62258000	0.27209300
C	0.17511700	-1.70528800	-0.10600000
C	-0.83953600	-0.76989500	-0.13053100
C	-1.04754200	0.62438700	0.11852700
C	-0.15986400	1.69242700	0.12604900
C	1.19462400	1.83656700	-0.25285600
H	1.54109400	0.14599500	1.46338100
H	3.19294500	-0.60107200	1.13665900
H	1.49634500	0.09588500	-1.44603900
H	2.99586400	1.12587000	-1.14843400
H	-0.16217800	-2.67676800	-0.45975500
H	-0.60595700	2.61606500	0.49079100
H	1.67537800	2.75729800	0.07422600
H	2.15807900	-2.45132300	-0.05435300
C	-2.50610400	0.97337900	0.38151400
H	-2.95923800	0.29898100	1.11318300
H	-2.58818400	1.99792400	0.75013400
H	-3.09720700	0.89457300	-0.53660800
F	-2.03664000	-1.37830900	-0.43043700

4-CH_3-5-CHO-substituted

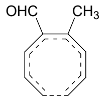

C	1.99339800	-1.20634900	0.92773800
C	2.34722000	-0.10091500	-1.03247000
C	0.93343400	-1.96633000	0.48452500
C	-0.33564200	-1.47980800	0.12412900
C	-0.87471400	-0.19964600	0.00095900
C	-0.35985100	1.14279500	0.15856000
C	0.91126200	1.67574300	-0.02278300
C	2.13166400	1.15042200	-0.50675900
H	1.81407300	-0.25231000	1.40326800
H	2.94206100	-1.68564500	1.15571500
H	1.53091700	-0.66065100	-1.46708000
H	3.34659300	-0.38270900	-1.35311400
H	-1.05933200	-2.25349600	-0.12603900
H	0.98866200	2.72154800	0.27094700
H	3.00327000	1.78058100	-0.33325800
H	1.10810600	-3.02211600	0.28527600
C	-1.39349000	2.18981600	0.58491700
H	-2.17091600	1.77279000	1.22987600
H	-0.89752800	2.98508600	1.14661200
H	-1.88247100	2.66456000	-0.27406700
C	-2.33349100	-0.25344200	-0.37727400
H	-2.78733900	0.70157400	-0.69248200
O	-3.00685500	-1.26036600	-0.41186300

4-CH_3-5-CN-substituted

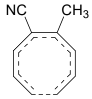

C	-2.14975300	-1.00726100	-0.89002300
C	-2.32155500	0.29444800	1.01709100
C	-1.25313500	-1.89943600	-0.35568400
C	0.08752800	-1.62293500	-0.02225600
C	0.83007200	-0.44013300	0.01873600
C	0.57901000	0.97334500	-0.17393800
C	-0.58224600	1.72412700	-0.05705600
C	-1.89178000	1.44066600	0.40037100
H	-1.79909800	-0.13113000	-1.41801400
H	-3.16777700	-1.32237800	-1.10242700
H	-1.61517300	-0.36983100	1.49561100
H	-3.35830500	0.20316300	1.32904100
H	0.66266700	-2.50017900	0.26607000
H	-0.46038900	2.75668500	-0.37786700
H	-2.63897800	2.19534500	0.15802700
H	-1.61176700	-2.88760700	-0.07463900
C	1.82685200	1.77896200	-0.51776300
H	2.39012200	1.32477200	-1.33848300
H	1.56187800	2.79850100	-0.80315200
H	2.50304300	1.83575100	0.34249700
C	2.23198200	-0.73607700	0.26968700
N	3.34170500	-0.99104600	0.48405100

4-CH_3-5-NO_2-substituted

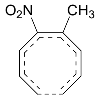

C	-2.36410100	-1.16587200	-0.79093700
C	-2.54704200	0.18529500	1.04362000
C	-1.36117800	-1.96208700	-0.29151800
C	-0.03588400	-1.55513500	-0.04137500
C	0.56154300	-0.30669800	-0.04228500
C	0.21871700	1.06531200	-0.28554300
C	-1.00059200	1.71820200	-0.16521200
C	-2.25394200	1.34677800	0.37223000
H	-2.11838200	-0.29170200	-1.37733100
H	-3.35915700	-1.57806800	-0.93412500
H	-1.75963700	-0.38203300	1.52069600
H	-3.55459300	0.01394800	1.41214500
H	0.64218100	-2.35937600	0.23094300
H	-0.98459100	2.73209800	-0.56135400
H	-3.07765700	2.01926300	0.13742200
H	-1.61245300	-2.96763200	0.03904800
C	1.36042100	1.95933000	-0.76880700
H	2.12754700	1.40393400	-1.31475500
H	0.95986900	2.71730500	-1.44605500
H	1.84570700	2.46861000	0.06804400
N	2.02872400	-0.45877100	0.26141200
O	2.48674300	0.26075100	1.13014800
O	2.66606200	-1.29521300	-0.35334800

4- NH_2-5-NH_2-substituted

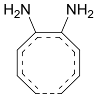

C	2.07182800	-0.70133300	0.91152400
C	2.07188400	0.70130400	-0.91148800
C	1.36426600	-1.71734100	0.31473300
C	0.02170500	-1.68661100	-0.11170700
C	-0.97368300	-0.71098900	-0.13836000
C	-0.97367600	0.71100700	0.13835700
C	0.02171800	1.68662500	0.11164200
C	1.36430400	1.71731600	-0.31472000
H	1.55453200	0.08186600	1.44875400
H	3.11573900	-0.85581800	1.17030900
H	1.55460700	-0.08188800	-1.44874800
H	3.11580700	0.85578400	-1.17022500
H	1.91452400	-2.60908500	0.01772900
H	-0.32803800	-2.64653600	-0.49242400
H	-0.32802700	2.64657500	0.49229300
N	-2.28620800	-1.20346800	-0.35360700
H	-2.30808700	-2.16941700	-0.65584700
H	-2.85040800	-0.60961600	-0.95526600
H	1.91455900	2.60907400	-0.01774200
N	-2.28619000	1.20349700	0.35362500
H	-2.85042300	0.60957000	0.95517600
H	-2.30806600	2.16941000	0.65597900

4- NH_2-5-OH-substituted

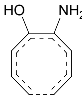

C	2.08901100	-0.69970100	0.89480400
C	2.04848600	0.77382300	-0.90165100
C	1.39488100	-1.70518200	0.27235400
C	0.03833100	-1.68215800	-0.11930300
C	-0.95721300	-0.71723000	-0.13434200
C	-1.01610500	0.70523600	0.11543200
C	-0.02466500	1.68721800	0.14945200
C	1.32370700	1.74364000	-0.25466200
H	1.56468700	0.06267800	1.45489100
H	3.13981600	-0.83902300	1.13276600
H	1.54768800	-0.00335800	-1.46331200
H	3.08992600	0.95654300	-1.15043200
H	1.95104100	-2.57979400	-0.06086100
H	-0.31354000	-2.64256200	-0.50102600
H	-0.39683300	2.63542100	0.53779300
H	1.85991300	2.63011600	0.08220400
N	-2.32545900	1.17524200	0.27845300
H	-2.99968600	0.51259000	0.63628800
H	-2.40419100	2.10108200	0.67532200
O	-2.23833100	-1.19210400	-0.38529800
H	-2.23256400	-2.15743200	-0.34293100

4- NH_2-5-F-substituted

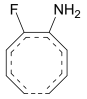

C	2.11144400	-0.64587600	0.87671400
C	1.98118800	0.86719600	-0.90801800
C	1.46256600	-1.67966300	0.25488500
C	0.09612900	-1.70428700	-0.10418600
C	-0.88907100	-0.74407700	-0.12899800
C	-1.04722000	0.66439400	0.11883100
C	-0.10857400	1.69334900	0.17675500
C	1.23782200	1.79890900	-0.23026800
H	1.55403100	0.08707900	1.44453300
H	3.17172800	-0.72645100	1.09926400
H	1.49728300	0.07971200	-1.47085100
H	3.01473500	1.08132300	-1.16337600
H	2.04787200	-2.53205900	-0.08393500
H	-0.28299900	-2.66264800	-0.45279000
H	-0.52019000	2.61795700	0.58062300
H	1.74972100	2.69457400	0.11968200
N	-2.38692100	1.04419800	0.26443500
H	-3.01353700	0.35809600	0.66291400
H	-2.52864100	1.97728900	0.62597500
F	-2.11636200	-1.30932500	-0.39415400

4- NH_2-5-CHO-substituted

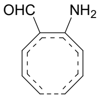

C	1.94136400	-1.24864000	0.91523400
C	2.28937300	-0.15990400	-1.07801100
C	0.84699200	-1.99344500	0.53284400
C	-0.40532200	-1.46721500	0.16648300
C	-0.88275300	-0.16994000	0.00708100
C	-0.30679300	1.14650500	0.21670900
C	0.96512700	1.66833900	-0.00946400
C	2.13283600	1.10779500	-0.56770600
H	1.80253100	-0.27146300	1.35722400
H	2.88189100	-1.74679900	1.13598900
H	1.43831000	-0.70505800	-1.46145400
H	3.26462700	-0.47351900	-1.43920200
H	0.97979000	-3.06060500	0.36727000
H	-1.15916300	-2.21037600	-0.08896400
H	1.08093100	2.69904100	0.32411600
H	3.02927300	1.71536200	-0.44852000
N	-1.28170700	2.06624800	0.63625400
H	-1.87154400	1.76529100	1.40231100
H	-0.96855600	3.02181400	0.74839700
C	-2.30640600	-0.12598000	-0.47343000
H	-2.62462700	0.83675000	-0.90711800
O	-3.06600100	-1.07240800	-0.46278400

4-NH_2-5-CN-substituted

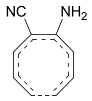

C	-2.10940200	-1.06820100	-0.89010100
C	-2.30412200	0.25106600	1.06976700
C	-1.17378000	-1.93296200	-0.38341900
C	0.15642400	-1.60883100	-0.04269000
C	0.86395100	-0.41017300	0.02840500
C	0.55837400	0.99404900	-0.20442400
C	-0.63460800	1.70613200	-0.10063700
C	-1.90686200	1.39488900	0.42889300
H	-1.80512800	-0.15647200	-1.38576200
H	-3.11592800	-1.42084000	-1.09810900
H	-1.58192800	-0.41954900	1.51465400
H	-3.33087300	0.15285600	1.40958200
H	-1.48781300	-2.94089000	-0.12145900
H	0.75746800	-2.46746800	0.24917300
H	-0.55618300	2.72461500	-0.47832800
H	-2.67692600	2.13269000	0.20579400
N	1.68846000	1.75410700	-0.49539500
H	2.42879800	1.33386100	-1.04177100
H	1.53886000	2.73323900	-0.69173900
C	2.26769400	-0.64399700	0.30812500
N	3.38634400	-0.86323100	0.51745800

4-NH_2-5-NO_2-substituted

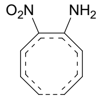

C	-2.32124000	-1.04839900	-1.02185000
C	-2.54734500	-0.00756700	1.10432300
C	-1.29964500	-1.88053000	-0.64174000
C	-0.00321700	-1.48783800	-0.24810600
C	0.60400700	-0.25565200	-0.08476000
C	0.21612900	1.13500800	-0.19530600
C	-1.03582100	1.70477600	0.05493100
C	-2.24687700	1.23395500	0.60401400
H	-2.11773800	-0.05593500	-1.39930300
H	-3.29505100	-1.46762700	-1.26004900
H	-1.77392900	-0.68256500	1.44278600
H	-3.55303600	-0.20813000	1.46234200
H	-1.51999900	-2.93579500	-0.49925100
H	0.65022500	-2.31676600	0.01160300
H	-1.06929100	2.75060900	-0.24584300
N	2.04976300	-0.46989600	0.24474400
H	-3.07434100	1.93545000	0.50319200
N	1.18833900	2.04816400	-0.57483400
H	2.10685800	1.72016200	-0.83223100
H	1.14846400	2.96508300	-0.15597600
O	2.30042400	-1.12883900	1.23398900
O	2.90397200	-0.01052000	-0.50219900

4-OH-5-OH-substituted

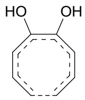

C	2.04781100	-0.71463300	0.89540600
C	2.04818700	0.71432500	-0.89533100
C	1.34272100	-1.72121300	0.28165600
C	-0.00847400	-1.68723900	-0.11841900
C	-0.99546300	-0.70926700	-0.12692100
C	-0.99527600	0.70941600	0.12687200
C	-0.00810600	1.68721300	0.11829100
C	1.34314400	1.72095800	-0.28160900
H	1.52858800	0.04973500	1.45773700
H	3.09507000	-0.86631800	1.14098000
H	1.52896300	-0.04991300	-1.45783100
H	3.09551100	0.86588600	-1.14070400
H	1.89292600	-2.60268500	-0.04347600
H	-0.36704300	-2.63819000	-0.51548200
H	-0.36651900	2.63827100	0.51524300
H	1.89343900	2.60234200	0.04360400
O	-2.27156500	1.13956800	0.40721800
H	-2.30027800	2.10394000	0.35145900
O	-2.27183500	-1.13918100	-0.40722200
H	-2.30072300	-2.10353200	-0.35117000

4-OH-5-F-substituted

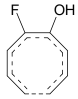

C	2.07215200	-0.66181600	0.87505100
C	1.98333500	0.81611400	-0.90146800
C	1.41441700	-1.69595700	0.25845300
C	0.05075700	-1.71158000	-0.10384700
C	-0.92767000	-0.74126000	-0.11918200
C	-1.02802900	0.66920200	0.12753600
C	-0.09405400	1.69537400	0.14944800
C	1.25674100	1.78022800	-0.24984500
H	1.51857400	0.07019400	1.44805600
H	3.13042300	-0.75258800	1.10285500
H	1.48153200	0.04153400	-1.46629400
H	3.02284900	1.00289200	-1.15426200
H	1.99545900	-2.55265500	-0.07696500
H	-0.33399300	-2.66275400	-0.46504700
H	-0.49660900	2.62277100	0.55858200
H	1.78134400	2.67110500	0.09167100
F	-2.15177800	-1.26416300	-0.40650900
O	-2.33327400	1.00845500	0.38576200
H	-2.43327000	1.96749500	0.31700700

4-OH-5-CHO-substituted

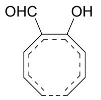

C	-1.89232300	-1.32408400	-0.89966000
C	-2.33655900	-0.15911300	1.04569600
C	-0.80022100	-2.01880100	-0.43392800
C	0.44301900	-1.45330000	-0.09425800
C	0.90817300	-0.14596700	-0.00616700
C	0.29122200	1.14488200	-0.19282700
C	-0.99183200	1.64759400	-0.02944000
C	-2.18291300	1.07894000	0.47376400
H	-1.75749300	-0.36781800	-1.38644000
H	-2.81743300	-1.85066900	-1.11845800
H	-1.49315900	-0.66930600	1.48984100
H	-3.32123100	-0.48073300	1.37227100
H	-0.91855600	-3.07636200	-0.20694800
H	1.21405600	-2.17289300	0.17654200
H	-1.10412400	2.67344200	-0.38198500
H	-3.08324800	1.65812500	0.27346200
O	1.24018100	2.05489900	-0.61043400
H	0.91779700	2.95758800	-0.47853700
C	2.36940700	-0.06912100	0.35675400
H	2.74442100	0.92661800	0.63702700
O	3.10621000	-1.03042000	0.39838600

4-OH-5-CN-substituted

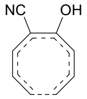

C	-2.06731400	-1.11185600	-0.86791300
C	-2.30821600	0.22378400	1.04150700
C	-1.12444200	-1.95521800	-0.33704700
C	0.20569800	-1.60969400	-0.02164800
C	0.88752800	-0.39549200	0.00668800
C	0.52809300	0.98943200	-0.20172100
C	-0.65160500	1.70674800	-0.08924700
C	-1.93571200	1.37602000	0.40171500
H	-1.76587800	-0.21793800	-1.39727200
H	-3.07085200	-1.47715500	-1.06755400
H	-1.57057700	-0.40888000	1.51610100
H	-3.33760800	0.09184800	1.36140800
H	-1.42984800	-2.95791000	-0.04624300
H	0.82873000	-2.45067000	0.27424500
H	-0.56752300	2.72709700	-0.46340800
H	-2.71715000	2.09254500	0.15273300
O	1.64472900	1.69750300	-0.56720700
H	1.50659100	2.64357000	-0.42045400
C	2.30520900	-0.57432100	0.26101500
N	3.41869300	-0.78842300	0.49543000

4-OH-5-NO_2-substituted

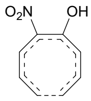

C	-2.27623300	-1.09416500	-1.03065900
C	-2.60224000	0.00103000	1.06762400
C	-1.26824700	-1.90509400	-0.57981900
C	0.02223700	-1.49624700	-0.18148000
C	0.61446800	-0.25264300	-0.05299400
C	0.18818500	1.12616000	-0.15953100
C	-1.05336800	1.70267600	0.06588900
C	-2.29502100	1.22987300	0.54784700
H	-2.06465800	-0.11137100	-1.42893400
H	-3.24076300	-1.52380900	-1.28645300
H	-1.83667600	-0.65829700	1.45156300
H	-3.62064300	-0.20818200	1.38230500
H	-1.48971000	-2.95549000	-0.40642400
H	0.68442900	-2.31167500	0.09550600
H	-1.03449300	2.75757600	-0.19707200
H	-3.12021500	1.92464300	0.39791600
O	1.12266400	2.06737500	-0.46473400
H	1.93612200	1.65136700	-0.80268300
N	2.07685000	-0.42408400	0.21873000
O	2.87931800	0.18628100	-0.48489400
O	2.40676500	-1.18687000	1.09986700

4-F-5-F-substituted

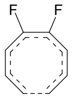

C	2.01161600	-0.76024800	0.87766100
C	2.01181800	0.76009700	-0.87766300
C	1.32853500	-1.75678700	0.22989900
C	-0.03675600	-1.72318000	-0.13018200
C	-0.96276700	-0.70513500	-0.11890700
C	-0.96267300	0.70519600	0.11892200
C	-0.03655400	1.72315100	0.13012700
C	1.32877400	1.75664800	-0.22986100
H	1.47554000	-0.01863700	1.45551700
H	3.06429900	-0.88724000	1.11256500
H	1.47574900	0.01857200	-1.45564400
H	3.06453600	0.88701900	-1.11245100
H	1.88431900	-2.62434900	-0.12017800
H	-0.46342800	-2.65303300	-0.49912500
H	-0.46316900	2.65308200	0.49894400
F	-2.22309700	-1.13865500	-0.38556400
H	1.88463000	2.62408300	0.12044800
F	-2.22295100	1.13888200	0.38555900

4-F-5-CHO-substituted

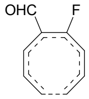

C	-1.84131000	-1.37416500	-0.89755000
C	-2.37252000	-0.15096700	1.01277500
C	-0.76066600	-2.03719100	-0.36902500
C	0.47861500	-1.45107500	-0.04384800
C	0.91900200	-0.13415200	-0.00519000
C	0.25100700	1.12425800	-0.17105500
C	-1.01403600	1.64915700	-0.03644700
C	-2.22458500	1.07282600	0.41432100
H	-1.70298800	-0.42970300	-1.40628400
H	-2.75651400	-1.91477600	-1.12223600
H	-1.53326100	-0.63458500	1.49357000
H	-3.36026500	-0.49307500	1.30789400
H	-0.87609100	-3.08596900	-0.10298600
H	1.26262000	-2.15093000	0.24055000
H	-1.06828200	2.68396200	-0.36556300
H	-3.12343300	1.63634100	0.17058500
F	1.14382600	2.09635000	-0.54785300
C	2.39268600	-0.00688200	0.29452600
H	2.77095900	1.01129600	0.46968700
O	3.14045700	-0.95507100	0.38180300

4-F-5-CN-substituted

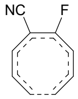

C	2.01934000	1.16420100	-0.86646300
C	2.34147000	-0.24056100	0.99570600
C	1.08980800	1.97846300	-0.27416500
C	-0.23973100	1.61870200	0.03427600
C	-0.89845400	0.39327400	0.02061300
C	-0.48883100	-0.97141500	-0.17668800
C	0.66347200	-1.71478400	-0.09110200
C	1.97067900	-1.38131000	0.33824200
H	1.70860800	0.28673800	-1.41797700
H	3.01763100	1.53838200	-1.07453000
H	1.60628500	0.36228300	1.51175100
H	3.37875300	-0.08724400	1.27865700
H	1.39838700	2.96953200	0.05113200
H	-0.87554100	2.44433500	0.34532700
H	0.51584300	-2.73538000	-0.43562500
H	2.74915300	-2.08264300	0.04342100
F	-1.56423900	-1.73785500	-0.50937900
C	-2.32699000	0.52050300	0.23632800
N	-3.45793500	0.67746500	0.42596900

4-F-5-NO_2-substituted

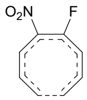

C	2.26014500	1.27384500	-0.74673100
C	2.55284600	-0.15631700	1.02306200
C	1.24260600	2.02018300	-0.20343800
C	-0.07157800	1.56119900	0.01858400
C	-0.61059300	0.29256800	-0.04120600
C	-0.14237300	-1.03266200	-0.29967700
C	1.05367100	-1.70394000	-0.20616300
C	2.30431600	-1.30549600	0.31568900
H	2.02862500	0.41747900	-1.36596500
H	3.24309400	1.71542400	-0.88307700
H	1.74727100	0.35194600	1.53623300
H	3.55818500	0.05585500	1.37486000
H	1.46544800	3.01607000	0.17237500
H	-0.78783300	2.31951300	0.32409800
H	1.00075400	-2.69806900	-0.64300000
H	3.14826000	-1.93335200	0.03719600
F	-1.14265600	-1.81613600	-0.78253300
N	-2.08476700	0.30804600	0.23405400
O	-2.50049700	-0.48820400	1.05172700
O	-2.75710000	1.14417400	-0.34035600

4-CHO-5-CHO-substituted

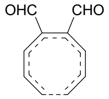

C	2.40384100	0.50883900	-1.00740400
C	2.40404900	-0.50850400	1.00731000
C	1.73534500	1.64268400	-0.60104100
C	0.38525200	1.70500300	-0.20358300
C	-0.57244100	0.72282900	0.01890000
C	-0.57232000	-0.72289900	-0.01882500
C	0.38550400	-1.70492800	0.20374000
C	1.73564100	-1.64242800	0.60102300
H	1.85299800	-0.32461000	-1.42089500
H	3.45424300	0.57012900	-1.27954700
H	1.85316700	0.32487900	1.42088300
H	3.45449900	-0.56966800	1.27929800
H	2.31022700	2.55750600	-0.46793000
H	0.01257500	2.70675700	0.00214100
H	0.01294600	-2.70674400	-0.00189900
H	2.31061000	-2.55719000	0.46787200
C	-1.89545500	-1.31366000	-0.45157300
H	-2.62115800	-0.59979000	-0.88532100
O	-2.16858500	-2.49111000	-0.42002000
C	-1.89567200	1.31339400	0.45166000
H	-2.62116900	0.59946900	0.88566600
O	-2.16909000	2.49077000	0.41983100

4-CHO-5-CN-substituted

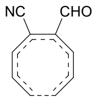

C	-2.46680100	-0.43068900	-0.99663200
C	-2.35033000	0.67863100	1.03605800
C	-1.90001000	-1.58755300	-0.52404200
C	-0.55213000	-1.76186200	-0.14201200
C	0.49746900	-0.86044700	0.02596200
C	0.64224000	0.57876900	-0.02882700
C	-0.23542600	1.63670400	0.15835200
C	-1.58659200	1.71409200	0.55676900
H	-1.85084600	0.34705600	-1.42616200
H	-3.52073800	-0.41223700	-1.25981000
H	-1.88122000	-0.19911300	1.45837700
H	-3.38680100	0.85170900	1.31298900
H	-2.54949300	-2.44203100	-0.34374300
H	-0.27998500	-2.79220000	0.07518900
H	0.23157200	2.59580100	-0.05921500
H	-2.07181600	2.67426600	0.39130300
C	2.04189300	1.05204500	-0.36347900
H	2.79810600	0.26650300	-0.52687900
O	2.34812100	2.21529000	-0.48201100
C	1.73248600	-1.57485300	0.31542200
N	2.68420900	-2.18301400	0.57265900

4-CHO-5-NO_2-substituted

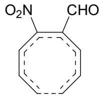

C	-2.61350500	-0.74414600	-0.91056000
C	-2.60197600	0.35678700	1.05696300
C	-1.82131200	-1.79205900	-0.49916900
C	-0.44922800	-1.71522900	-0.18833000
C	0.38468700	-0.62319300	-0.04023400
C	0.29471200	0.80802000	-0.10936900
C	-0.74822500	1.69758500	0.10111200
C	-2.06882900	1.52549800	0.56114000
H	-2.16678000	0.11344600	-1.39387300
H	-3.66690500	-0.91471900	-1.11479600
H	-1.95101600	-0.39660400	1.47911100
H	-3.63874800	0.33061200	1.38119300
H	-2.29666300	-2.74772400	-0.28874900
H	0.03448700	-2.66604100	0.01322000
H	-0.47988000	2.71816500	-0.16412100
H	-2.73636300	2.37239900	0.41352200
N	1.78299700	-1.07161000	0.29973900
O	2.41398200	-0.36192200	1.06523200
O	2.19737000	-2.11338500	-0.17415800
C	1.55482200	1.49909800	-0.59952200
H	2.32760500	0.85030000	-1.04676400
O	1.70195000	2.69621600	-0.59221100

4-CN-5-CN-substituted

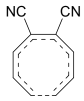

C	-2.41788100	0.62374100	0.98750000
C	-2.41788000	-0.62373100	-0.98750800
C	-1.75464300	1.68971300	0.43837800
C	-0.39275500	1.72014100	0.06387000
C	0.57079200	0.72355900	-0.05291700
C	0.57078200	-0.72355400	0.05290700
C	-0.39277900	-1.72012900	-0.06383500
C	-1.75465000	-1.68971300	-0.43839600
H	-1.87181600	-0.17783200	1.46582300
H	-3.46989500	0.71127500	1.24404400
H	-1.87180200	0.17783400	-1.46583100
H	-3.46989100	-0.71125700	-1.24406400
H	-2.32508900	2.58478100	0.19897500
H	-0.01979300	2.70933200	-0.19089700
H	-0.01984000	-2.70930200	0.19103400
C	1.88388500	1.28463300	-0.31711600
N	2.90720500	1.77651700	-0.54249900
H	-2.32510600	-2.58477700	-0.19899400
C	1.88387400	-1.28464300	0.31711100
N	2.90718800	-1.77654000	0.54249200

4-CN-5-NO_2-substituted

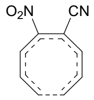

C	-2.58482400	-0.77663600	-0.93713900
C	-2.64368300	0.37655100	1.01531300
C	-1.75965700	-1.77624400	-0.47907800
C	-0.39336300	-1.63863100	-0.15825500
C	0.40437900	-0.52212200	-0.01497600
C	0.25151100	0.90610200	-0.10212800
C	-0.83171100	1.76713200	0.05295900
C	-2.15371500	1.54875700	0.48854700
H	-2.16612000	0.08682200	-1.43528300
H	-3.62782200	-0.99388400	-1.15002100
H	-1.96885800	-0.33889700	1.46538900
H	-3.68379600	0.31541400	1.32288100
H	-2.19876600	-2.74458400	-0.24886800
H	0.12640400	-2.56862200	0.05218800
H	-0.61043400	2.79288200	-0.23126800
H	-2.85144500	2.36189000	0.30003200
C	1.45165400	1.62353600	-0.49575400
N	2.35539900	2.24172100	-0.87022000
N	1.81644200	-0.92065600	0.32464500
O	2.37961400	-0.25673400	1.17315800
O	2.28718700	-1.89440900	-0.23227800

4-NO_2-5-NO_2-substituted

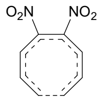

C	-2.78686600	-0.52414000	-0.96527000
C	-2.78697100	0.52311900	0.96568900
C	-2.11621800	-1.65363300	-0.54119800
C	-0.76090300	-1.71321000	-0.16822100
C	0.17121400	-0.71333800	0.02148900
C	0.17094900	0.71370800	-0.02132100
C	-0.76192200	1.71305300	0.16751800
C	-2.11705700	1.65281700	0.54100500
H	-2.23920700	0.27565600	-1.44511200
H	-3.84106300	-0.59414300	-1.21901400
H	-2.23868600	-0.27630500	1.44546100
H	-3.84108800	0.59266800	1.21988200
H	-2.69212300	-2.55797100	-0.35750900
H	-0.38133900	-2.70413200	0.06165400
H	-0.38333500	2.70409400	-0.06346000
H	-2.69350900	2.55679600	0.35726200
N	1.49819200	-1.27728000	0.46155600
O	2.12372000	-0.61339200	1.26765300
O	1.83097800	-2.36496200	0.03951900
N	1.49768500	1.27787700	-0.46135000
O	2.12401800	0.61285700	-1.26579800
O	1.82951500	2.36660900	-0.04121900
